# Design, modular synthesis and screening of 58 shape-diverse 3-D fragments

**DOI:** 10.1039/d5sc05819h

**Published:** 2025-09-19

**Authors:** Thomas D. Downes, S. Paul Jones, James D. Firth, John F. Darby, Amelia K. Gilio, Hanna F. Klein, Xinyu Wang, David C. Blakemore, Claudia De Fusco, Stephen D. Roughley, Lewis R. Vidler, Maria Ann Whatton, Alison J.-A. Woolford, Gail L. Wrigley, Roderick E. Hubbard, Liang Wu, Gideon J. Davies, Peter O'Brien

**Affiliations:** a Department of Chemistry, University, of York York YO10 5DD UK peter.obrien@york.ac.uk; b Medicine Design, Pfizer Inc 445 Eastern Point Road Groton CT 06340 USA; c Medicinal Chemistry, Oncology R&D, AstraZeneca Francis Crick Ave Cambridge CB2 0AA UK; d Vernalis (R&D) Ltd Granta Park, Abington Cambridge CB21 6GB UK; e Eli Lilly and Company UK 8 Arlington Square West, Downshire Way Bracknell Berkshire RG12 1PU UK; f Astex Pharmaceuticals 436 Cambridge Science Park, Milton Road Cambridge CB4 0QA UK

## Abstract

Fragment-based drug discovery is widely used in both academia and industry during the early stages of drug discovery. There is a growing interest in the design of 3-D fragments for inclusion in fragment libraries in order to increase chemical space coverage. We present herein the design and synthesis of 58 shape-diverse 3-D fragments that are prepared using just three modular synthetic methodologies. The 3-D fragments comprise a cyclic scaffold (cyclopentane, pyrrolidine, piperidine, tetrahydrofuran or tetrahydropyran) with one aromatic or heteroaromatic ring and possess properties within ‘rule-of-three’ fragment space. 3-D shape is assessed using principal moments of inertia analysis and conformational diversity is achieved by considering all conformations up to 1.5 kcal mol^−1^ above the energy of the global minimum energy conformer. Due to the modular nature of the fragment syntheses, these 3-D fragments are synthetically-enabled for fragment elaboration follow-on work, a key design feature. This modular, shape-diverse 3-D fragment collection has delivered privileged starting points across a spectrum of targets. Fragments from the set have been crystallographically validated in the SARS-CoV-2 main protease (M^pro^) and the nonstructural protein 3 (Nsp3) (Mac1) as well as human glycosyltransferase MGATV, a major enzyme in the mammalian *N*-glycosylation pathway and a promoter of aggressive metastatic cancers, underscoring the breadth of biological space that can be explored.

## Abstract 2

The design, modular synthesis and screening of 58 shape-diverse 3-D fragments that are synthetically enabled for fragment elaboration is described.

## Graphical abstract

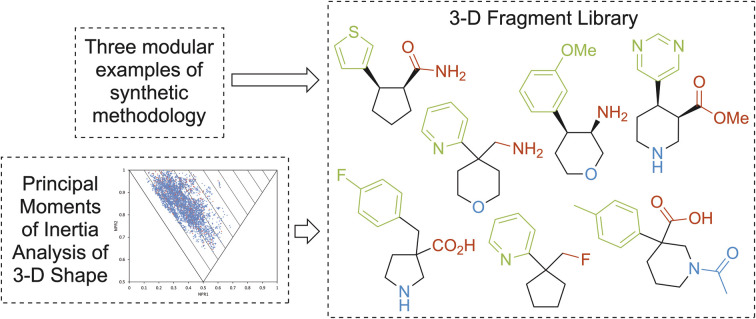

## Introduction

Fragment-based drug discovery (FBDD) continues to play a key role in hit identification during the early stages of drug discovery.^1–5^ This is highlighted by the fact that there are now eight drugs and over 59 clinical candidates that have their origins in FBDD programs.^6^ Due to the small size of fragment libraries (typically 1000–2000 compounds), it is necessary for the library to be carefully designed to generate high quality starting points for drug discovery.^6–11^ Typically, the physicochemical properties of the library components follow the widely accepted fragment ‘rule-of-three’ (*e.g.* MW < 300 and *c* log *P* < 3).^12,13^ In general, 3-D shape diversity was not an important consideration in early fragment libraries and sp^2^ rich compounds with planar aromatic systems predominated. However, there is an increasing recognition that inclusion of some 3-D fragments into fragment libraries is useful due to improvements in chemical space and pharmacophore coverage as well as overall library diversity.^9,14–16^ In addition, a more shape-diverse library could display a broader range of biological activities and be more successful in finding hits for non-traditional targets.^14,17^ Finally, 3-D fragments may have greater solubility and may be less promiscuous binders then planar aromatic counterparts.^4,18^

The growing interest in 3-D fragments has led to developments in the synthesis of 3-D fragments over the last 10 years. Different methodologies have been adopted to access 3-D fragments and we have reviewed both the physicochemical/3-D properties^19^ and the synthetic strategies used.^20,21^ As well as our contributions to 3-D fragments,^22–24^ recent synthetic methods to access 3-D fragments include Wijtmans *et al.*'s approach from biomass-derived dihydrolevoglucosenone (CyreneTM),^25^ Foley *et al.*'s arylation of the 7-oxabicyclo[2.2.1]heptane scaffold^26^ and a heterocycle assembly strategy developed by researchers at Merck.^27^ Furthermore, several vendors provide commercial 3-D fragment libraries: Life Chemicals 3D Fragment Library,^28^ ChemDiv 3D FL Fragment Library,^29^ Enamine 3D Shape Diverse Fragment Library.^30^

Two commonly used methods for assessing the 3-D shape of fragments are principal moments of inertia (PMI)^31^ and plane-of-best-fit;^32^ both methods are preferred to using the fraction of sp^3^ carbons (Fsp^3^)^33^ as a 3-D shape metric. For example, we have recently shown that there is little to no correlation between Fsp^3^ and the three-dimensionality of fragments, as measured by PMI analysis,^22^ and a similar lack of correlation between plane-of-best-fit and Fsp^3^ for medicinally-relevant compounds has been noted.^16,32^

**Fig. 1:**
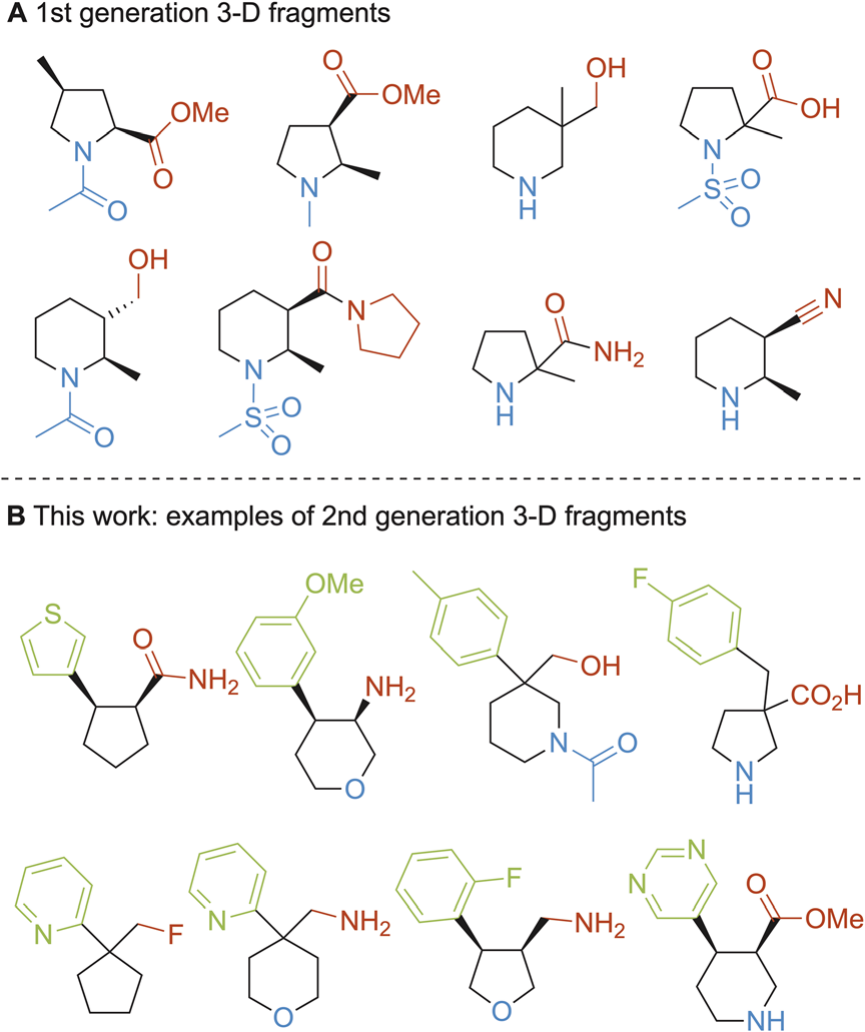
(A) 1st generation 3-D fragments; (B) this work: exemplar 2nd generation 3-D fragments.

To address the increasing interest in 3-D fragments for incorporation into fragment libraries, we have previously reported the use of PMI analysis to select the most 3-D compounds from a pool of virtually enumerated pyrrolidine and piperidine based fragments before carrying out any synthesis.^22^ Uniquely, this approach also considered the 3-D shape of all conformations up to 1.5 kcal mol^−1^ above the energy of the global minimum energy conformer for each fragment. As a result, a collection of 56, 1st generation, shape-diverse 3-D fragments were synthesised (Fig. 1A, selected examples) and formed part of the York 3-D fragment collection which is also available at the Diamond XChem facility.^34^ Unfortunately, this set of 3-D fragments has had a rather low hit rate in several screening campaigns (by both NMR spectroscopy and X-ray crystallography). In retrospect, these 3-D fragments, which contained amines, amides or sulfonamides as part of a pyrrolidine or piperidine scaffold with one other functional group (*e.g.* ester, alcohol, nitrile, ether, amide and carboxylic acid) (Fig. 1A) were arguably too simple to make productive interactions with most of the proteins investigated. Part of this can be attributed to the fact that these fragments lacked aromatic or heteroaromatic functionality and so productive π–π interactions with the proteins were not available. The absence of aromatic rings also meant that ligand-observed NMR screening^35^ of these 3-D fragments was challenging.^36^ There were additional synthetic limitations with this set of 56 fragments. First, whilst we attempted to develop general synthetic routes, several of the selected fragments required bespoke multistep syntheses. Second, with this set of 3-D fragments, elaboration from a fragment hit to a lead series was likely to be synthetically challenging at positions other than the functional groups that were likely interacting with the proteins. Researchers from Astex have identified that fragment elaboration is a bottleneck in the fragment-to-lead optimisation stage.^37^ Indeed, they introduced the term “fragment sociability” whereby fragments for which fragment elaboration is synthetically enabled are referred to as “sociable” and those where it is not as “unsociable”.^38^ The original set of 56 fragments could be deemed to be “unsociable fragments”.

To address all these limitations, we set out to create a new collection of *ca.* 50 3-D fragments that was based on some of our original design features but also contained at least one aromatic or heteroaromatic ring to increase the potential for protein interactions. A key design element for these 2nd generation 3-D fragments would be that a limited set of modular and robust methodologies to introduce the aryl/heteroaryl functionality would be utilised. This would ensure that the 3-D fragments could initially be rapidly synthesised and, crucially, that they were synthetically enabled for the required, and currently rate-limiting, follow-on work involving fragment-to-lead elaboration. It was thus envisaged that the 2nd generation 3-D fragments could be deemed “sociable”. The following design criteria were used – 3-D fragments would: (i) be built around a cyclic scaffold comprising a cyclopentane, pyrrolidine, piperidine, tetrahydrofuran or tetrahydropyran ring and would contain one aromatic or heteroaromatic ring; (ii) possess properties within ‘rule-of-three’ fragment space (MW < 300 Da, *c* log *P* < 3);^12^ (iii) be accessible using robust, modular methods to expedite both their synthesis and their subsequent elaboration to a hit series; (iv) be derived from a virtually enumerated library of potential 3-D fragments that had been evaluated by PMI analysis to ensure that they explored new areas of 3-D space; (v) possess conformational diversity by assessing the 3-D shape of all conformations up to 1.5 kcal mol^−1^ above the energy of the global minimum energy conformer for each fragment, in line with our previously disclosed approach.^22^ Selected examples of the 2nd generation 3-D fragments are shown in Fig. 1B. Most, but not all, the 3-D fragments are chiral. For a screening collection, we decided to work with racemic compounds for the chiral molecules. However, since only one enantiomer is likely to be biologically active, follow-up work on fragment hits with single enantiomers should be considered and others have indeed included that in related examples.^39^ However, the work in this paper only focuses on the use of racemic compounds.

In this paper, using design criteria (i)–(v) outlined above, focusing on specific scaffolds and three types of methodology for the introduction of aryl and heteroaryl groups, we report the design and modular synthesis of 58 shape-diverse 3-D fragments. Of note, by basing the synthetic routes around only three methods, we ensure that the 3-D fragments are synthetically enabled for fragment-to-lead elaboration and can thus be classed as “sociable” fragments.^38^ As highlighted herein, this modular, shape-diverse 3-D fragment collection has delivered privileged starting points across a spectrum of targets. Fragments from the set have been crystallographically validated in the SARS-CoV-2 main protease (M^pro^) and the nonstructural protein 3 (Nsp3) (Mac1) as well as human glycosyltransferase MGATV, an enzyme in the mammalian *N*-glycosylation pathway and a promoter of metastatic cancers. This highlights the range of biological space that can be investigated with these 3-D fragments. Furthermore, since every fragment is purposely ‘sociable’ and can be readily elaborated through the same chemistry that built the collection, we anticipate that this resource has the potential to accelerate fragment-to-lead campaigns in virology, oncology and beyond. Herein, we describe our results.

## Results and discussion

**Fig. 2:**
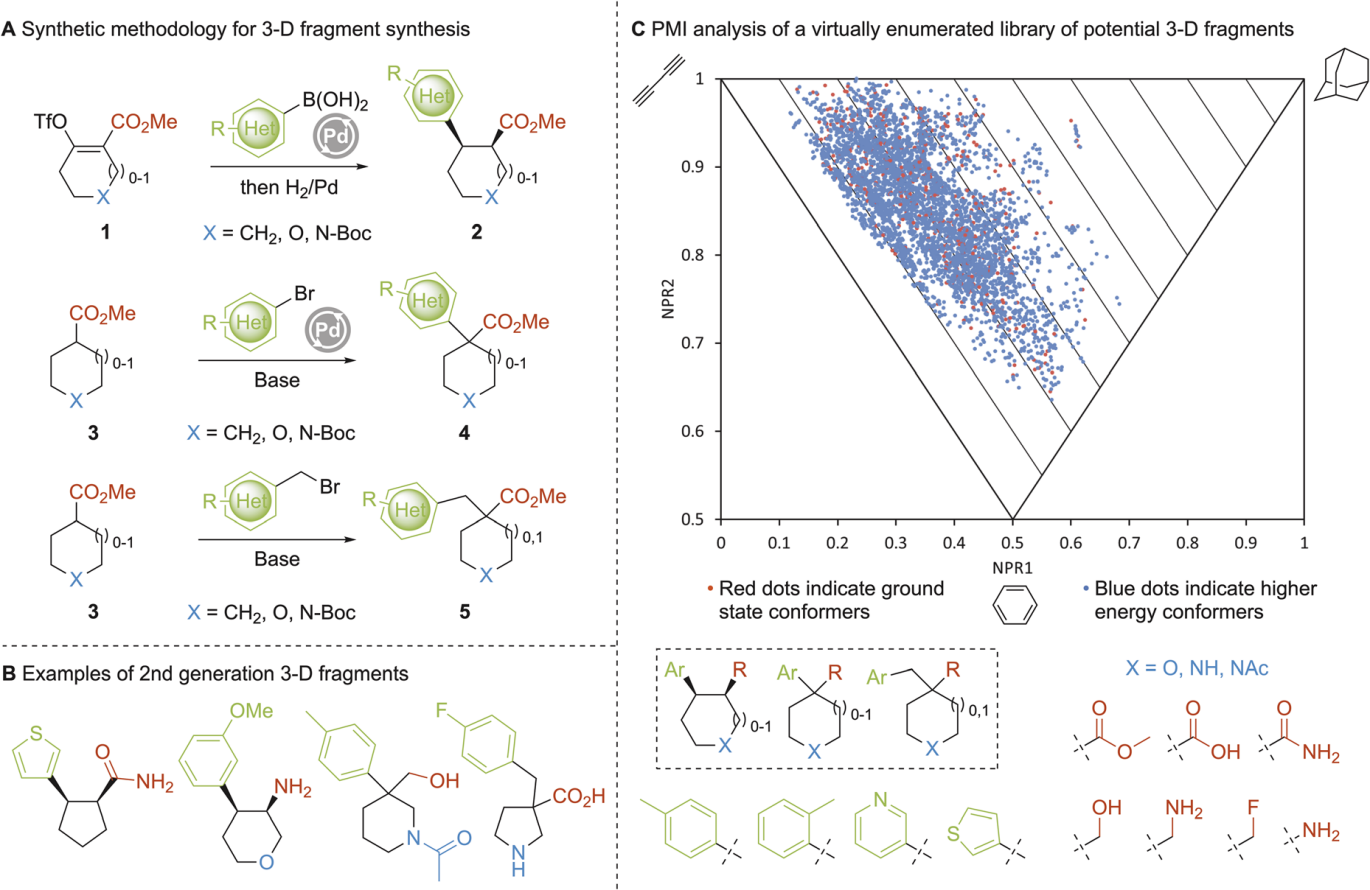
(A) Methodology for 3-D fragment synthesis; (B) exemplar 2nd generation 3-D fragments; (C) principal moments of inertia (PMI) analysis of potential fragments (red dots indicate ground state conformers and blue dots indicate higher energy conformers).

For this new set of 3-D fragments, the selection of both the scaffolds and suitable synthetic methodology was key. The plan was to design 3-D fragment building blocks with five- and six-membered ring scaffolds containing one aromatic or heteroaromatic group and an ester group (which could also be transformed into a range of other functionalities). Thus, we set out to identify modular, predictable and robust synthetic methods that would allow the introduction of aryl/heteroaryl groups to aliphatic cyclic esters. Three such methodologies were identified for our purposes (Fig. 2A). First, it was envisaged that Suzuki–Miyaura cross-coupling of enol triflates 1 with aryl/heteroaryl boronic acids, followed by alkene hydrogenation, would give 1,2-*cis*-disubstituted building blocks 2. Second, Pd-catalysed enolate α-(hetero)arylation of (hetero)cyclic esters 3 would access building blocks 4 equipped with a quaternary centre. Third, benzylation of (hetero)cyclic esters 3 to give 5 would increase the structural diversity of the quaternary centre-containing building blocks. It was envisioned that several of building blocks 2, 4 and 5 would have suitable molecular properties to be used as 3-D fragments themselves and further modification of ester or amino functionalities would enable additional 3-D fragments to be readily accessed. Exemplar 3-D fragments that were ultimately synthesised using these synthetic methods are shown in Fig. 2B.

Before commencing any synthetic work, the potential of these three methods to generate 3-D fragments in less well explored 3-D space was validated. Using our previously disclosed approach,^22^ the 3-D shape of a virtual library of 504 compounds was assessed using PMI analysis (Fig. 2C). The enumerated virtual library comprised 12 cyclic scaffolds (5- and 6-membered rings including oxygen and amine functionality (blue)), four aryl substituents (green) and seven functional groups (an ester and ones that could be derived from an ester, red) (see SI for full details). Shape analysis of these virtual fragments was performed using PMI analysis of their conformations up to 1.5 kcal mol^−1^ above the energy of the global minimum energy conformer (Fig. 2C, red dots are ground state conformers and blue dots are higher energy conformers).

With triangular PMI plots of the normalized PMIs (NPR1 *versus* NPR2), the three apexes correspond to disc (bottom), rod (top-left) and spherical (top-right) shapes; lines parallel to the rod–disc axis correspond to ΣNPR values (where ΣNPR = NPR1 + NPR2, ranging from 1.00–2.00). Conformations that lie furthest from this rod–disc axis are of interest as they deviate the most from planarity. This analysis of potential fragments shows that their conformations cover a wide range of chemical space whilst avoiding the overpopulated rod–disc axis and the first 10% of the PMI plot (ΣNPR < 1.10). Indeed, only 11 of the 504 global minimum energy conformers, and 198 of the 4619 conformers, fall within this region. This analysis provided confidence that our synthesised 3-D fragments would target the most interesting parts of 3-D chemical space, where ΣNPR > 1.10. However, we did not limit our synthetic efforts to 3-D fragments defined by the virtual library of 504 compounds (some were synthesised) as we also desired a wider range of aryl/heteroaryl and other functional group diversity in the new 3-D fragment collection.

**Scheme 1:**
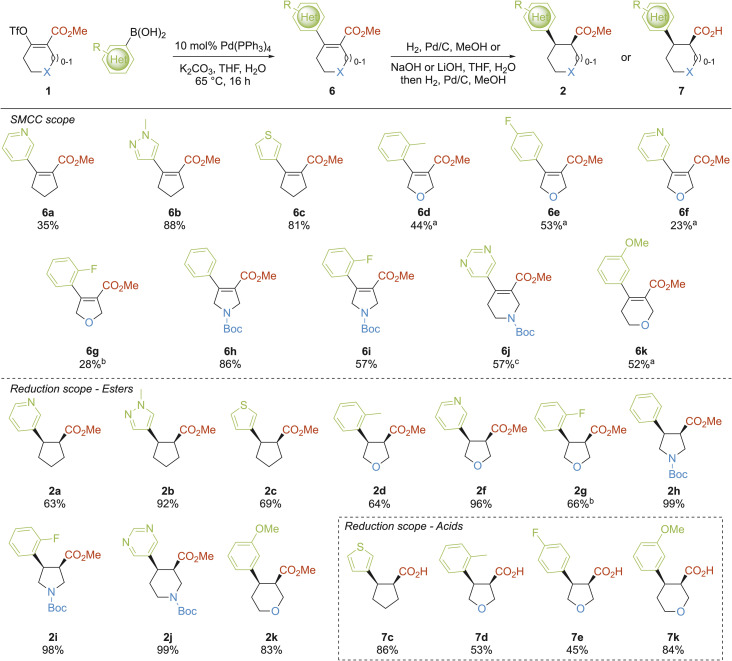
Synthesis of 1,2-*cis*-fragment building blocks 2 and 7. ^a^Yield over 2 steps from corresponding β-keto ester; ^b^isolated as a 60 : 40 mixture with the aryl boronic acid; ^c^using Pd(dppf)Cl_2_, KHCO_3_, THF, H_2_O, 65 °C, 2 h.

The synthetic investigations began with the synthesis of 1,2-*cis*-disubstituted building blocks 2 and 7 (Scheme 1). Such 1,2-*cis*-difunctionalised scaffolds have been studied previously^40–42^ and we included cyclopentane, tetrahydrofuran, pyrrolidine, piperidine and tetrahydropyran scaffolds. First, the scope of the Suzuki–Miyaura cross-coupling of enol triflates 1 was explored. Five structurally diverse enol triflates 1 were cross-coupled with a range of aryl and heteroaryl boronic acids in a non-exhaustive manner. Most cross-couplings proceeded readily using one set of unoptimised conditions (10 mol% Pd(PPh_3_)_4_, K_2_CO_3_, 4 : 1 THF/H_2_O (ref. 41)) giving 11 structurally-diverse arylated products 6a–k in 23–88% yields. Both five- and six-membered enol triflates worked well. Cyclopentyl enol triflate was coupled with three heteroaryl boronic acids to give 6a–c in 35–88% yields. For dihydrofurans 6d–g, the requisite enol triflate was partially unstable and so crude enol triflate was taken into the cross-coupling reaction to give dihydrofurans 6d–g in 23–53% yields over two steps. A similar two-step method was used to synthesise dihydropyran 6k in 52% yield. Boc-protected nitrogen-containing enol triflates worked very well, with dihydropyrroles 6h–i and tetrahydropyridine 6j isolated in 57–86% yields.

Subsequent hydrogenation of alkenes 6 over Pd/C gave 1,2-*cis*-substituted ester building blocks 2 in 63–99% yields. The expected *cis*-stereoselectivity of the hydrogenation was proven for 2h, by converting it into 8m (an *N*-acetyl analogue of 2h), whose synthesis is described later (see Scheme 4), and comparing NMR spectroscopic data with *trans*-8m which was independently synthesised *via* a *trans*-stereospecific route. Six of these compounds (2a–d, 2f and 2k) had the molecular properties (MW < 300 Da, *c* log *P* < 3) for direct inclusion in the fragment collection. These fragments, together with 2g–j were manipulated further to generate suitable 3-D fragments for the collection (*vide infra*). Tetrahydrofuran 2g could not be separated from residual boronic acid (a 60 : 40 mixture of 2g and aryl boronic acid was isolated) but this mixture could be used in subsequent steps (*vide infra*). It was also of interest to include some carboxylic acids 7 in the fragment collection and, additionally, carboxylic acids 7 would be useful intermediates for the generation of other 3-D fragments. However, with 8m (an *N*-acetyl analogue of 2h), typical ester hydrolysis conditions (LiOH, THF–water–MeOH, rt, 2 h) led to significant amounts of epimerisation such that the corresponding *trans*-acid was the major product. Therefore, a two-step protocol for accessing 1,2-*cis* acids 7 was developed in which the order of the steps was reversed. Thus, hydrolysis of esters 6 to the carboxylic acids prior to the hydrogenation step gave acids 7c–e and 7k in 45–86% yields, which all had suitable properties to be added to the collection. The *cis*-selectivity of the hydrogenation was established by X-ray crystallography of 7c and 7e (see SI).

**Scheme 2:**
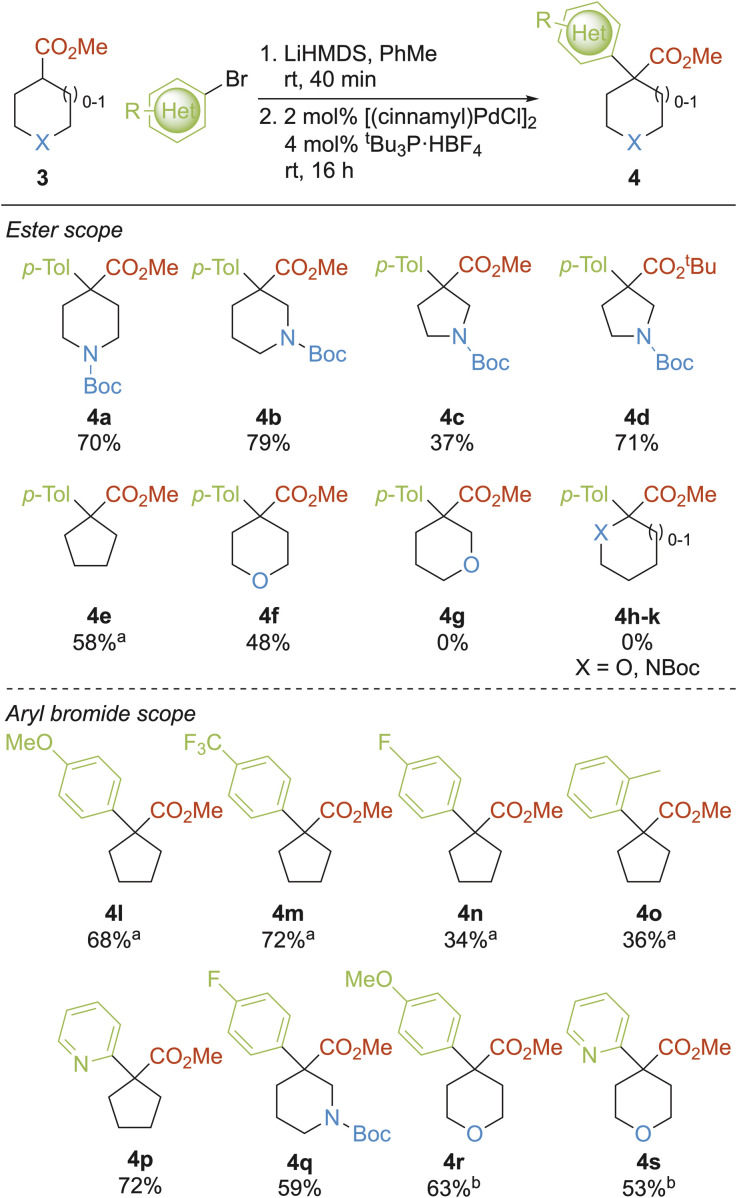
Arylation of cyclic esters with aryl/heteroaryl bromides. ^a^LDA used instead of LiHMDS; ^b^reaction performed at 50 °C.

The second modular approach identified for the synthesis of 3-D fragments was the Pd-catalysed α-arylation of enolates of cyclic esters 3. This would generate 3-D fragments and building blocks 4 with all-carbon quaternary centres (Scheme 2). There have been several reports on the α-arylation of substituted cyclohexyl esters;^43–46^ with more limited studies performed on heterocyclic or five-membered ring esters.^47,48^ Using the conditions developed by Zhou *et al.* (LiHMDS, then [(cinnamyl)PdCl]_2_ and *t*-Bu_3_P·HBF_4_ in toluene),^45^ the substrate scope of enolate α-arylation of a range of esters 3 was explored using *p*-tolyl bromide (Scheme 2). Gratifyingly, 4- and 3-substituted Boc protected piperidines were readily α-arylated to give 4a and 4b in 70% and 79% yield respectively. α-Arylation of an analogous *N*-Boc pyrrolidine gave 4c in a lower 37% yield, which may be a result of the poor solubility of the lithium enolate in toluene, a common issue for this chemistry in our hands. In support of this conjecture, α-arylation of the analogous *tert*-butyl ester resulted in a visibly more soluble lithium enolate and isolation of 4d in an improved 71% yield. α-Arylation of cyclopentyl methyl ester gave 4e in 58% yield when LDA was used as the base (34% yield of 4e with LiHMDS) and 4-substituted tetrahydropyran 4f was obtained in 48% yield. When using the 3-substitituted THP, none of 4g was obtained (which may be due to a β-alkoxy elimination in the lithium enolate^49^). Regardless of ring size or heteroatom, 2-substituted esters 4h–k were inaccessible using this Pd-catalysed α-arylation methodology.

With the scope and limitations of cyclic esters 3 determined, a range of aryl and heteroaryl bromides was assessed. Using the cyclopentyl ester with LDA, *para*-substituted aryl bromides gave 4l-o in 34–72% yields; of note, the sterically demanding *ortho*-methyl group was tolerated and 4o was generated in 36% yield. Furthermore, 2-pyridyl substituted cyclopentane 4p was isolated in 72% yield when using LiHMDS as the base (scale-up to 7.8 mmol scale gave 4p in 71% yield). Three further heteroatom-containing cyclic ester derivatives 4q–s were all accessed in good yields, with 50 °C required to bring about the cross-coupling to give 4r and 4s in satisfactory yields. Overall, 14 3-D fragment building-blocks 4 were accessed by expanding the scope of established enolate α-arylation.^43–48^ Furthermore, tetrahydropyran aryl esters 4f, 4r and 4s, as well as 2-pyridyl ester 4p, fulfilled the criteria for fragments and were added to the fragment collection.

**Scheme 3:**
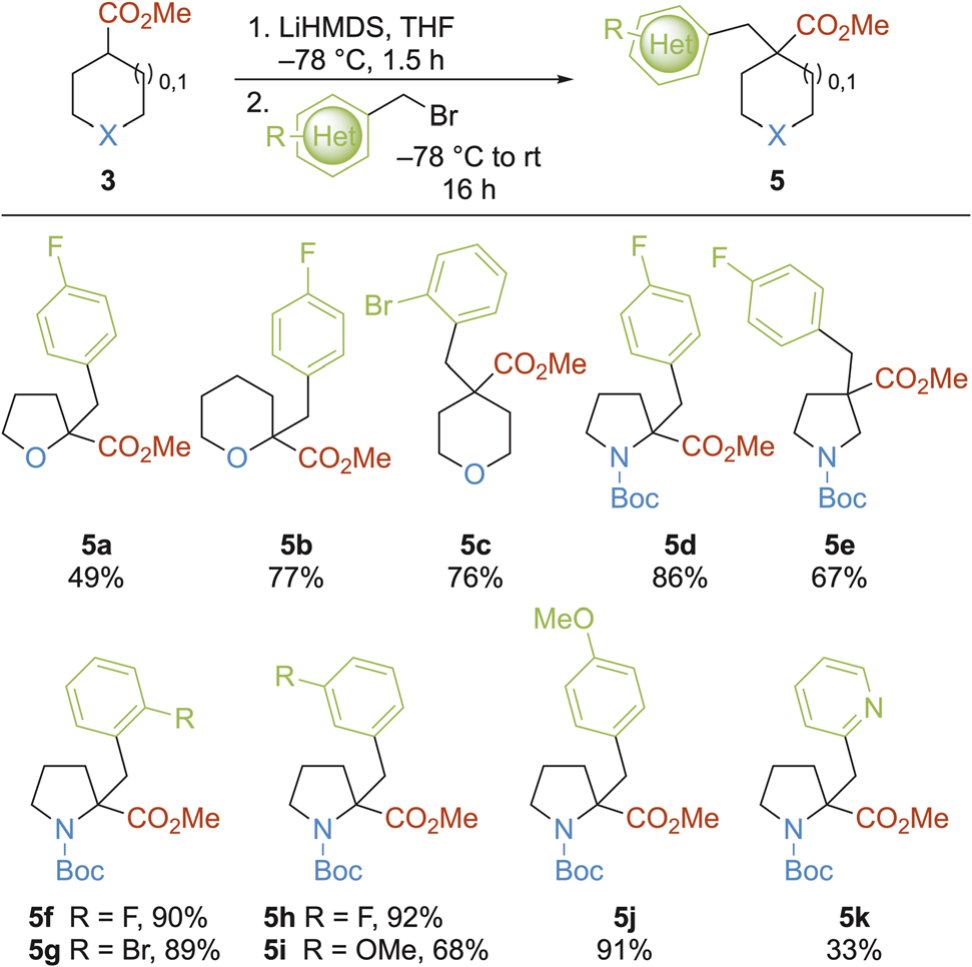
Alkylation of cyclic esters with aryl-/heteroaryl-containing bromomethanes.

The third modular approach to 3-D building blocks was the α-alkylation of enolates derived from cyclic esters 3 using aryl- and heteroaryl-containing bromomethanes to give 5, which also contained all-carbon quaternary centres (Scheme 3).^50^ To increase the pharmacophore coverage and structural diversity of the fragment collection, we focused primarily on using cyclic esters 3 that were unsuccessful substrates in the α-arylation methodology. Treatment of esters 3 with LiHMDS at −78 °C, followed by addition of substituted benzyl bromides gave substituted THF 5a, 2- and 4-substituted THPs 5b and 5c and 2- and 3-substituted pyrrolidines 5d and 5e in 49–86% yields. Next, 2-substituted *N*-Boc pyrrolidine fragment precursors 5f–k were synthesised in 33–92% yields; *ortho*- (5f–g), *meta*- (5h–i) and *para*- (5j) substituted benzyl bromides were well tolerated and 2-pyridyl benzyl bromide could be incorporated (5k), albeit in lower yield (33%). This method provided two 3-D fragments (THF 5a and THP 5b) and a range of building blocks (5c–k).

**Scheme 4:**
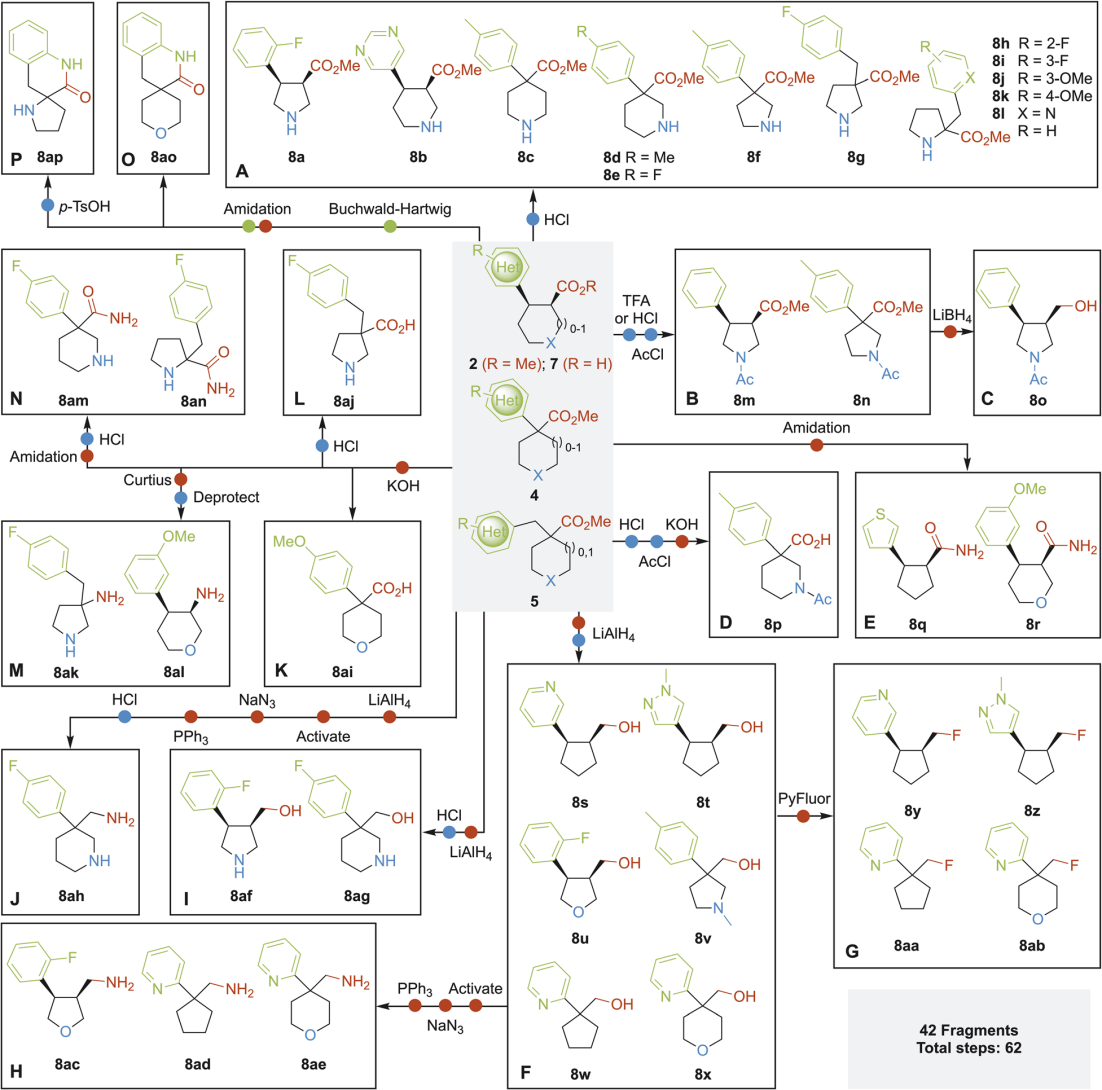
Synthesis of 42 fragments starting from 2, 4, 5 and 7.

These three synthetic methods delivered 16 ‘rule-of-three’ compliant cyclic fragments (MW < 300 Da, *c* log *P* < 3), together with many building blocks, from readily accessible starting materials in an expedient manner. Importantly, as outlined in Scheme 4, these building blocks 2, 4, 5 and 7 were decorated with ester, acid and amino functionalities that could be manipulated further to generate an additional 42 fragments (see SI for full experimental details). Removal of Boc groups from *N*-Boc protected building blocks gave 12 cyclic amine fragments 8a–l (Scheme 4A). Boc group removal followed by acetylation gave 8m and 8n (Scheme 4B); subsequent selective reduction of the ester of 8m using LiBH_4_ gave amino alcohol 8o (Scheme 4C). Similarly, amine deprotection, acetylation and ester hydrolysis of 4b gave acid 8p (Scheme 4D). Amidation of carboxylic acids 7c and 7k using aqueous ammonia and T3P® gave primary amines 8q and 8r respectively (Scheme 4E). Six primary alcohol fragments 8s–x were accessed through ester reduction with LiAlH_4_ (Scheme 4F). Fluorination of the heteroaryl-containing subset of these alcohols (8s, 8t, 8w and 8x) using PyFluor^51^ gave alkyl fluorides 8y–ab, further increasing the pharmacophore diversity of the collection (Scheme 4G). Furthermore, primary alcohols were converted into primary amines 8ac–ae through a three-step sequence involving activation (as a sulfonate), S_N_2 displacement with NaN_3_ and Staudinger reduction (Scheme 4H). Amino alcohols 8af and 8ag were accessed *via* ester reduction followed by Boc group removal (Scheme 4I). Diamine 8ah was synthesised from 4q*via* a five-step sequence: the ester in 4q was transformed into an amine in four steps and then the Boc group was removed (Scheme 4J). Carboxylic acid fragment 8ai was synthesised from 4r*via* ester hydrolysis (Scheme 4K) and 8aj was accessed from 5e through hydrolysis followed by amine deprotection (Scheme 4L). Amines 8ak and 8al were synthesised from their corresponding carboxylic acids through Curtius rearrangement and subsequent amine deprotection (Scheme 4M). Similarly, primary amides 8am and 8an were formed from the corresponding acids, followed by Boc group removal. Finally, spirocyclic lactam fragments 8ao and 8ap were synthesised from aryl bromide containing building blocks 5c and 5g respectively (Scheme 4O and P). Thus, amine functionality was introduced through Buchwald–Hartwig cross-coupling,^52^ followed by lactam formation (and Boc group removal in the case of 8ap). In all, as summarised in Scheme 4, 42 3-D fragments were synthesised in a total of 62 synthetic steps from building blocks 2, 4, 5 and 7, with an average of only 1.5 steps per fragment. The overall yields for each 3-D fragment 8 in Scheme 4, starting from the respective 3-D building blocks, are presented in the SI.

Overall, 58 cyclic 3-D fragments (see SI for structures) were prepared using simple modular approaches. Despite the apparent simplicity of the 3-D fragments, it is notable that 53 are novel. An analysis of the physicochemical properties showed that almost all of the 58 3-D fragments conformed to the ‘rule-of-three’ (Table 1). A comparison between the 1st and 2nd generation 3-D fragments is also provided in Table 1. The 2nd generation 3-D fragments have a higher mean MW and lipophilicity, which may account for more detectable binding interactions with proteins compared to the 1st generation 3-D fragments. The stability and solubility of the fragments was assessed to ensure that they were suitable for incorporation into a screening collection. Of the 58 fragments, 56 fragments were stable to prolonged storage on the bench and in DMSO stock solutions (>6 weeks). Of these, 50 3-D fragments were stable in aqueous buffer for >24 h. Crucially, all stable fragments were soluble at a concentration of >0.5 mM in aqueous buffer and are therefore suitable for biophysical screening.

**Table 1:** Mean physicochemical properties of the synthesised 3-D fragment collection

Property^a^	Idealised range^b^	Calculated values, 2nd generation fragments	Calculated values, 1st generation fragments
MW	≤300	216 ± 23	172 ± 38
clogP	≤3	1.4 ± 0.83	0.54 ± 0.55
HBA	≤3	2.5 ± 091	2.7 ± 0.73
HBD	≤3	0.72 ± 0.69	0.89 ± 0.70
RBC	≤3	2.2 ± 0.67	1.6 ± 0.77
TPSA	≤60	41 ± 10	47 ± 19

a MW = molecular weight, HBA = hydrogen bond acceptors, HBD = hydrogen bond donors, RBC = rotatable bond count, TPSA = topological polar surface area.

b Rule-of-three guidelines.^12^

**Fig. 3:**
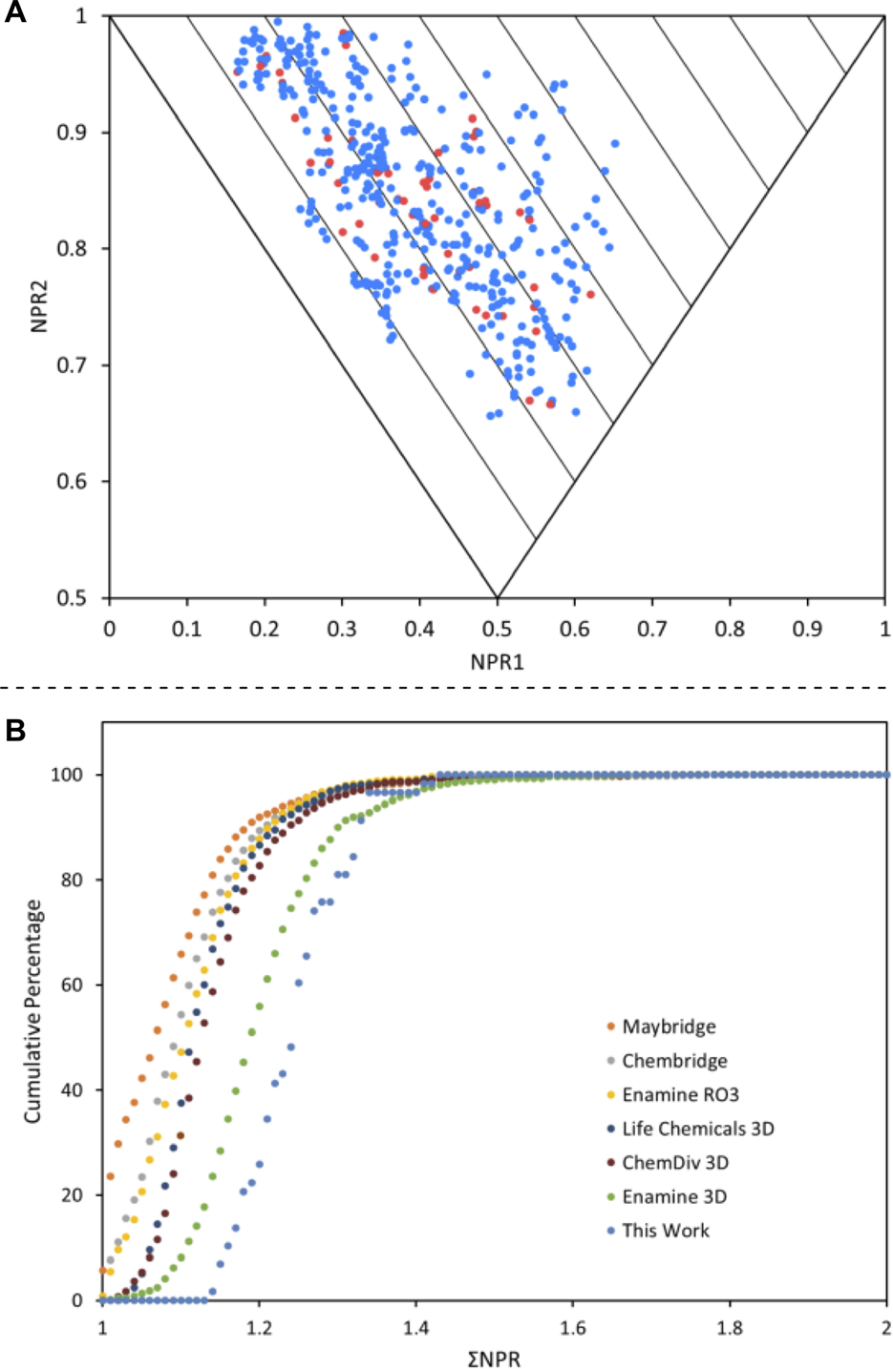
(A) PMI plot of the final fragment collection of 58 3-D fragments. Red dots indicate global minimum energy conformers and blue dots indicate higher energy conformers. (B) Cumulative PMI analysis of the fragment collection (light blue) along with six commercially available libraries.

The PMI plot of the 58 synthesised fragments is shown in Fig. 3A, clearly indicating that these new 3-D fragments cover a wide area of 3-D chemical space. Importantly, there are no conformers occupying the rod–disc axis and very few (<4%) within the first 10% of the PMI plot (ΣNPR <1.10); there are no global minimum energy conformers in the ΣNPR 1.00–1.10 region. Finally, to show that the 3-D fragments targeted under-represented areas of fragment space, we compared this collection of 3-D fragments with six commercial fragment libraries, including three that were designed to be 3-D in nature (Life Chemicals 3D Fragment Library, ChemDiv 3D FL Fragment Library, Enamine 3D Shape Diverse Fragment Library, accessed in 2017–2019). Using a random selection of 1000 compounds from each of the six commercial fragment libraries, all conformers (up to 1.5 kcal mol^−1^ above the energy of the global minimum energy conformer) were generated.^22^ Then, the mean distance from the rod–disc axis (ΣNPR) was determined for each fragment, based on its conformations. Fig. 3B shows the cumulative percentage of fragments within a defined mean distance from the rod–disc axis (ΣNPR). The fact that our 3-D fragments are the furthest to the right on this plot highlight that they are more three-dimensional than even commercially available 3-D fragment libraries.

**Fig. 4:**
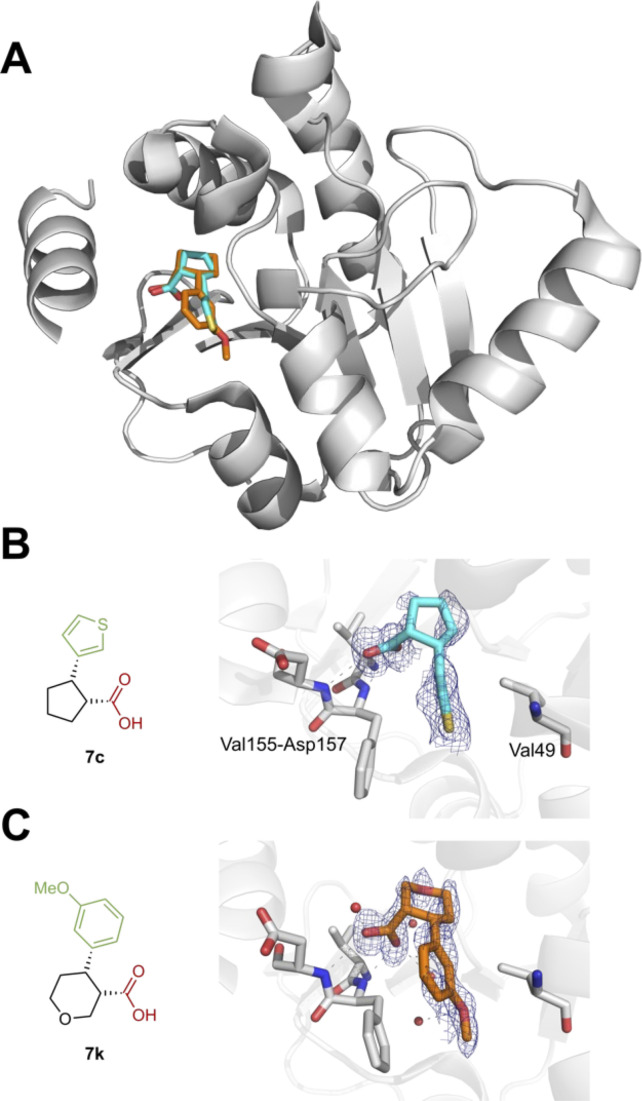
(A) Overview of SARS-CoV-2 Mac1 protein fold, showing binding site of 7c and 7k. (B) Molecular interactions of 7c with Mac1. (C) Molecular interactions of 7k with Mac1. Grey dashes denote H-bonds. Red spheres denote water molecules. Electron densities for ligands were calculated using the PanDDA method, and contoured to 0.5–1.0 *σ* (0.24–0.49 e^−^ Å^−3^).

The majority of these 3-D fragments were added to the York 3-D fragment collection at the Diamond XChem facility.^34^ During the early stages of the Covid-19 pandemic in March 2020, researchers at Diamond screened all of their in-house libraries against a number of the proteins found in SARS-CoV-2, including the main protease (M^pro^) and the nonstructural protein 3 (Nsp3) (also known as Mac1). From the high-throughput X-ray crystallographic screening against M^pro^, it was found that two of the new 3-D fragments, 8q and 8w, bound to the active site and dimer interface respectively.^53^ In addition, screening against Nsp3 (Mac1), revealed three structurally-related fragment hits, 7c, 7d and 7k.^54^ X-ray structures of 3-D fragments 7c (PDB: 5S3T) and 7k (PDB: 5S3X) are shown in Fig. 4. In each case, the carboxylic acid was hydrogen bonded to both Phe156 and Asp157 in the oxyanion subsite and there was evidence of weak π–π interactions with the phenyl ring of Phe156. These initial results highlight the usefulness of the 3-D fragments for identifying starting points for drug discovery. In addition, a key feature of these new 3-D fragments is that they are synthetically-enabled for fragment elaboration and fragment-to-lead development. This is a result of the modular nature of the synthetic methods used to create the 58 3-D fragment collection. A follow-on optimisation campaign starting from 3-D fragments 7c, 7d and 7k was readily accomplished as a result of our modular synthetic methodology and will be reported separately.

The 58 3-D fragments were also screened against *N*-acetylglucosaminyltransferase V (MGATV), a glycosyltransferase which is involved in the construction of complex-type tetra-antennary *N*-glycans.^55,56^ Upregulation of MGATV strongly drives cancer aggression and metastasis, due to the effects of excessive cell-surface tetra-antennary *N*-glycans in controlling growth-factor receptor turnover.^57^ Despite interest in MGATV inhibition as an anticancer strategy, few pharmacological inhibitors have been reported,^58–60^ and no molecules have been evaluated clinically. Very recently, Schumann and co-workers reported a complementary bio-orthogonal tool that selectively labels MGATV substrates *in vitro* and in live cells, further highlighting the emerging interest in chemical modulation of this transferase.^61^

A thermal shift assay (TSA) was used as an initial screen for potential binders. The 3-D fragments were screened against MGATV at both 4 mM and 8 mM fragment concentrations. Two compounds, 7d and 8p, were identified that produced a modest stabilising effect on the MGATV denaturation temperature, with 8p also stabilising in a dose dependent manner (see SI). As a result, 8p was progressed to an enzyme activity assay, to assess the ability to inhibit the MGATV catalyzed glycosyltransferase reaction. In accordance with TSA data, 8p showed selective MGATV inhibition, and also displayed dose dependent inhibition; we identified an IC_50_ of ∼4.4 mM (*n* = 1). Since racemic 8p was used, an even lower IC_50_ might be obtained if the appropriate enantiomerically pure compound was used.

**Fig. 5:**
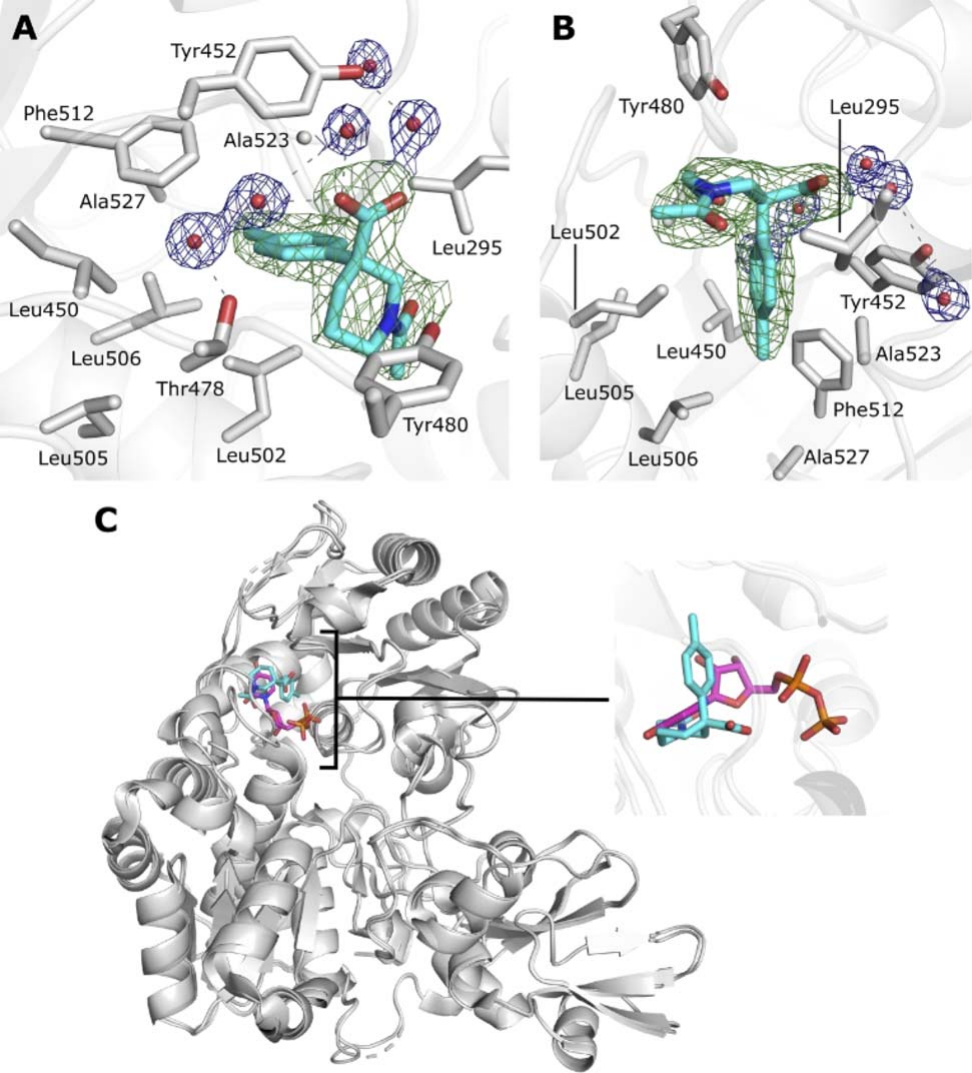
(A) Fragment 8p in the MGATV active site, showing water mediated H-bonding interactions to Tyr452 and Thr478; (B) hydrophobic ‘basket’ like motif involved in interacting with the tolyl moiety of 8p; (C) superposition of the 8p complex with a previously solved complex of MGATV bound to the donor subsite ligand UDP. 8p binds in the same subsite as UDP-GlcNAc substrates, and therefore acts as a competitive inhibitor of MGATV glycosyl donor substrate binding. Electron densities for waters are REFMAC *σ*_A_-weighted 2mFo-DFc, contoured to 1 *σ* (0.23 e^−^ Å^−3^). Electron density for ligand is REFMAC *σ*_A_-weighted mFo-DFc, contoured to 2.5 *σ* (0.175 e^−^ Å^−3^).

Encouraged by the biophysical results with 7d and 8p, we attempted to obtain a co-crystal complex X-ray structure between each of the fragments 7d and 8p and MGATV that might provide key information about their modes of binding. Although we were unable to obtain a structure of a complex with 7d, clear electron density in the MGATV crystal structure was observed after soaking with 8p, corresponding to a single molecule of the fragment 8p occupying the enzyme active site (Fig. 5; PDB code: 8CE3). Fragment 8p bound MGATV *via* a water-mediated H-bonding network from the carboxylate moiety to enzyme residues Tyr452 and Thr478 (Fig. 5A), as well as an unusual hydrophobic interaction between the tolyl moiety of 8p and a basket-like structure lined by residues Leu502, Leu505, Leu450, Leu506, Ala527, Phe512 and Ala523 of MGATV (Fig. 5B). These interactions place the bound fragment within the UDP-GlcNAc substrate binding pocket of MGATV (Fig. 5C), implicating 8p as a competitive inhibitor of MGATV glycosyl donor binding. Additionally, we confirmed the validity of 8p as an MGATV inhibitor by STD NMR (see SI) which was facilitated by the fact that the fragment contained an aryl group.

The co-crystal structure between 8p and MGATV (Fig. 5) also offers several clues towards further fragment development to obtain a more active inhibitor. Both the carboxylate and acetamide moieties of 8p offer vectors for fragment growth, which may lead to improvements in inhibitor potency and specificity. Whilst our co-crystal structure suggests that the shape of the MGATV ‘basket’ motif may sterically restrict growth of the tolyl motif in 8p to a sterically larger hydrophobic group, substitution to other aromatic or aliphatic groups may enable more optimal interactions that improve compound potency and selectivity.

## Conclusions

In summary, we have presented the design and synthesis of a collection of 58 3-D fragments that target under-represented areas of fragment chemical space. Five design criteria, that included 3-D shape analysis and conformational diversity, ‘rule-of-three’ compliance and synthetic tractability were utilised. The resulting 3-D fragments were built around cyclic scaffolds that also contained one aryl or heteroaryl ring. This fragment collection has proved extremely successful in a range of fragment screening studies, especially by X-ray crystallography. This shape-diverse 3-D fragment collection has delivered useful starting points across a range of targets. Fragments from the set have been crystallographically validated in the SARS-CoV-2 main protease (M^pro^) and the nonstructural protein 3 (Nsp3) (Mac1) as well as in the human glycosyltransferase MGATV, indicating the scope of biological space that can be tackled. Since every fragment is purposely ‘sociable’ and can be readily elaborated through the same chemistry that built the collection, we anticipate that this resource has the potential to accelerate fragment-to-lead campaigns in virology, oncology and beyond.

## Author contributions

All authors contributed to the conceptualization and writing (review & editing). TDD, SPJ, JDF, JFD, AKG, HFK, XW, LW and POB carried out the investigation, methodology, data curation and formal analysis. POB, REH, LW and GJD provided supervision and funding acquisition. POB, JDF and LW carried out the writing (original draft). DCB, CDF, SDR, LRV, MAW, AJAW and GLW provided invaluable insight and contributions from a pharmaceutical industry perspective.

## Conflicts of interest

There are no conflicts to declare.

## Supplementary Material

SC-016-D5SC05819H-s001

SC-016-D5SC05819H-s002

## Data Availability

Data for this article, including raw NMR data, are available at https://doi.org/10.15124/8be73bb1-2623-487e-a828-dd20e8d67131. CCDC 2096673 and 2096674 contain the supplementary crystallographic data for this paper.^62,63^ Supplementary information is available. This contains experimental details and characterisation data, library analysis and the MGATV analysis methods. See DOI: https://doi.org/10.1039/d5sc05819h.
