# Supplementary material for: Design, modular synthesis and screening of 58 shape-diverse 3-D fragments
Source: Chem Sci. 2025 Sep 19;16(42):20030–41. doi: 10.1039/d5sc05819h (PMC12486152; doi:10.1039/d5sc05819h)
Supplement: SC-016-D5SC05819H-s001 [file SC-016-D5SC05819H-s001.pdf]

Supplementary Information (SI) for Chemical Science.
This journal is © The Royal Society of Chemistry 2025

Design, Modular Synthesis and Screening of 58 Shape-Diverse 3-D Fragments

Thomas D. Downes+,[a] S. Paul Jones+,[a] James D. Firth,[a] John F. Darby,[a,b] Amelia K. Gilio,[a] Hanna F. Klein,[a] Xinyu Wang,[a] David C. Blakemore,[c] Claudia De Fusco,[d] Stephen D. Roughley,[e] Lewis R. Vidler,[f] Maria Ann Whatton,[g] Alison J.-A. Woolford,[h] Gail L. Wrigley,[i] Roderick E. Hubbard,[a,e] Liang Wu,[a,j,k] Gideon J. Davies,[a] and Peter O'Brien*[a]

[a] Department of Chemistry, University of York, Heslington, York YO10 5DD, U.K.

[b] Current address: Xyme AI, Inventa, Botley Road, Oxford, OX2 0HA.

[c] Medicine Design, Pfizer Inc, 445 Eastern Point Road, Groton, CT 06340, USA

[d] Current address: Amphista Therapeutics, The Cori Building, Granta Park, Great Abington, Cambridge, CB21 6GQ, U.K.

[e] Vernalis (R&D) Ltd, Granta Park, Abington, Cambridge, CB21 6GB, U.K.

[f] Eli Lilly and Company UK, 8 Arlington Square West, Downshire Way, Bracknell, Berkshire RG12 1PU, U.K.

[g] Current address: Evotec (UK) Ltd, Dorothy Crowfoot Hodgkin Campus, 114 Innovation Drive, Milton Park, Abingdon, Oxon, OX14 4RZ U.K.

[h] Astex Pharmaceuticals, 436 Cambridge Science Park, Milton Road, Cambridge, CB4 0QA, U.K.

[i] Medicinal Chemistry, Oncology R&D, AstraZeneca, Francis Crick Ave, Cambridge, CB2 0AA, U.K.

[j] Current address: The Rosalind Franklin Institute, Harwell Science & Innovation Campus, OX11 0QX, Didcot, U.K.

[k] Current address: Division of Structural Biology, Nuffield Department of Medicine, University of Oxford, The Wellcome Centre for Human Genetics, OX3 7BN, Oxford, U.K.

peter.obrien@york.ac.uk

The data supporting this paper can be found at:

https://doi.org/10.15124/8be73bb1-2623-487e-a828-dd20e8d67131

Index

1. Experimental Details S3

1.1 General S3

1.2 General Procedures S4

1.3 Experimental Procedures and Characterisation Data S7

2. Library Analysis S101

2.1 General S101

2.2 Virtual Library Enumeration S103

2.3 Shape Analysis of Virtual Library of *trans*-Isomers of **2** S104

2.4 Commercial Fragment Library Details S105

2.5 Structures and Smiles Files of the 58 3-D Fragments S106

2.6 Yields for the 42 3-D Fragments Synthesised from Building Blocks (Scheme 4) S109

3. MGAT4 Methods S110

3.1 Protein production, Crystallization and Complex Generation S110

3.2 Data Collection and Processing S110

3.3 Thermal Shift Assays S112

3.4 UDP-Glo Enzyme Inhibition Kinetics S113

3.5 STD NMR Studies S114

4. ^{1}H and ^{13}C NMR Spectra S116

5. References S246

1. Experimental Details

1.1. General

All-non aqueous reactions were carried out under oxygen free Ar or N_2 using flame-dried glassware. Et_2O and THF were freshly distilled from sodium and benzophenone. Alkyllithiums were titrated against *N*-benzylbenzamide before use. Brine refers to a saturated solution. Water is distilled water.

Flash column chromatography was carried out using Fluka Chemie GmbH silica (220-440 mesh). Thin layer chromatography was carried out using commercially available Merck F_{254} aluminium backed silica plates. Proton (400 MHz) and carbon (100.6 MHz) NMR spectra were recorded on a Jeol ECX-400 instrument using an internal deuterium lock. For samples recorded in $CDCl_3$, chemical shifts are quoted in parts per million relative to $CHCl_3$ (δ_H 7.26) and $CDCl_3$ (δ_C 77.0, central line of triplet). For samples recorded in CD_3OD, chemical shits are quoted in parts per million relative to CHD_2OD (δ_H 3.31, central line of quintet) and CD_3OD (δ_C 49.1, central line of septet). Carbon NMR spectra were recorded with broad band proton decoupling and assigned using DEPT experiments. Coupling constants (*J*) are quoted in Hertz. Melting points were carried out on a Gallenkamp melting point apparatus. Infrared spectra were recorded on a Perkin Elmer UATR Two FT-IR spectrometer. Electrospray high and low resonance mass spectra were recorded at room temperature on a Bruker Daltronics microOTOF spectrometer.

1.2 General Procedures

General Procedure A: Formation of Enol Triflates

iPr_2NEt (5.0 eq.) was added dropwise to a stirred solution of the *β*-ketoester (1.41-21.6 mmol, 1.0 eq.) in CH_2Cl_2 (25-200 mL) at –78 °C under Ar. The resulting solution was stirred at –78 °C for 10 min and then trifluoromethanesulfonic anhydride (1.2 eq.) was added dropwise over 15 min. The resulting solution was allowed to warm to rt and stirred at rt for 16 h. H_2O (25-100 mL) and then 5% citric acid$_{(aq)}$ (25-200 mL) were added and the aqueous layer was extracted with CH_2Cl_2 (3 × 25-100 mL). The combined organic extracts were dried (Na_2SO_4) and evaporated under reduced pressure to give the crude product.

General Procedure B: Suzuki-Miyaura Coupling

K_2CO_3 (2.5 eq.) was added portionwise to a stirred mixture of the enol triflate (0.37-7.29 mmol, 1.0 eq.) and aryl boronic acid (1.5 eq.) in THF (4-40 mL) and H_2O (1-10 mL) at rt. The reaction flask was evacuated under reduced pressure and back-filled with Ar three times. Then, $Pd(PPh_3)_4$ (0.05-0.1 eq.) was added and the resulting mixture was stirred and heated at 65 °C for 16 h. The mixture was allowed to cool to rt and H_2O (10-100 mL) was added. The aqueous layer was extracted with EtOAc (3 × 10-100 mL). The combined organic extracts were dried (Na_2SO_4) and evaporated under reduced pressure to give the crude product.

General Procedure C: Hydrogenation

10% Pd/C or 10% $Pd(OH)_2/C$ (0.01-0.03 eq.) was added to a stirred solution of the alkene (0.23-6.07 mmol, 1.0 eq.) in MeOH (5-50 mL) at rt. The reaction flask was evacuated under reduced pressure and back-filled with Ar three times. Then, the reaction flask was evacuated under reduced pressure and back-filled with H_2 three times. After the final evacuation, H_2 was charged and the reaction mixture was stirred vigorously under a H_2 balloon at rt for 2-112 h. Then, the solids were removed by filtration through Celite and the solvent was evaporated under reduced pressure to give the crude product.

General Procedure D: Enolate arylation using LiHMDS

A solution of cyclic ester (1.2 eq.) in toluene (1 mL) was added dropwise to a stirred solution of LiHMDS (1.0 M solution in toluene, 2.0 eq.) in toluene (2 mL) at rt under Ar. The resulting solution was stirred at rt for 40 min. In a separate reaction flask, [(cinnamyl)$PdCl_2$] (2 mol%), *t*-$Bu_3P·HBF_4$ (4 mol%) and aryl bromide (0.51-1.30 mmol, 1.0 eq.) were added, followed by dry toluene (4-5 mL). The reaction flask

was then evacuated under reduced pressure and back-filled with Ar three times. The LiHMDS/ester solution was then added dropwise to the reaction flask and the resulting solution was stirred at rt for 16 h. The solution was poured into 1 M $AcOH_{(aq)}$ (10 mL) and the aqueous layer was extracted with EtOAc (3 × 10 mL). The combined organics were washed with brine (20 mL) and sat $NaHCO_{3(aq)}$ (20 mL), dried ($MgSO_4$) and evaporated under reduced pressure to give the crude product.

General Procedure E: Enolate arylation of methyl cyclopentanecarboxylate using LDA

n-BuLi (2.3 M solution in hexane, 2.0 eq.) was added dropwise to a stirred solution of diisopropylamine (2.0 eq.) in dry toluene (1 mL) at 0 °C under Ar. The resulting solution was stirred at 0 °C for 30 min. A solution of methyl cyclopentanecarboxylate (1.2 eq.) in toluene (1 mL) was added dropwise to the LDA solution at 0 °C under Ar. The solution was stirred at 0 °C for 10 min and then allowed to warm to rt and stirred at rt for 10 min. In a separate reaction flask, [(cinnamyl)$PdCl_2$] (2 mol%), *t*-$Bu_3P{\cdot}HBF_4$ (4 mol%) and aryl bromide (0.79-1.56 mmol, 1.0 eq.) were added, followed by dry toluene (4-5 mL). The reaction flask was then evacuated under reduced pressure and back-filled with Ar three times. The LDA/ester solution was then added dropwise to the reaction flask and the resulting solution was stirred at rt for 16 h. The solution was poured into 1 M $AcOH_{(aq)}$ (10 mL) and the aqueous layer was extracted with EtOAc (3 × 10 mL). The combined organics were washed with brine (20 mL) and sat $NaHCO_{3(aq)}$ (20 mL), dried ($MgSO_4$) and evaporated under reduced pressure to give the crude product.

General Procedure F: Enolate alkylation using LiHMDS

LiHMDS (1.0 M solution in toluene, 1.4 eq.) was added dropwise to a stirred solution of cyclic ester (0.3-2.28 mmol, 1.0 eq.) in THF (4 mL) at –78 °C under Ar. The resulting solution was stirred at –78 °C for 1.5 h. Then, a solution of (substituted) benzyl bromide (1.4 eq.) in THF (1 mL) was added dropwise –78 °C under Ar. The resulting solution was stirred at –78 °C and slowly warmed up to rt over 16 h. The solution was poured into sat $NH_4Cl_{(aq)}$ (10 mL) and the two layers were separated. The aqueous layer was extracted with EtOAc (3 × 10 mL) and the combined organics were dried ($MgSO_4$) and evaporated under reduced pressure to give the crude product.

General Procedure G: Boc group removal using HCl solutions

HCl (4 M solution in dioxane or 2 M solution in Et_2O, 1-2.3 mL) was added to *N*-Boc heterocycle (0.06-0.41 mmol, 1.0 eq.) and the resulting mixture was stirred at rt for 16 h. Then, the volatiles were removed under reduced pressure to give the secondary amine as a hydrochloride salt.

General Procedure H: Ester Reduction with $LiAlH_4$

A solution of ester (0.49-3.88 mmol, 1.0 eq.) in THF (2-10 mL) at 0 °C was added dropwise to a stirred suspension of $LiAlH_4$ (2.0-5.0 eq.) in THF (2.5-10 mL) at 0 °C under Ar. The resulting mixture was stirred at 0 °C for 2 h. Water (1 mL for each mmol of LiAlH4), 20% $NaOH_{(aq)}$ (2 mL for each mmol of LiAlH4) and water (1 mL for each mmol of LiAlH4) were added sequentially (CARE – vigorous reaction). Then, $MgSO_4$ was added and the solids were removed by filtration. The filtrate was evaporated under reduced pressure to give the crude product.

1.3 Experimental Procedures and Characterisation

Methyl 2-(trifluoromethanesulfonyloxy)cyclopent-1-ene-1-carboxylate 1a

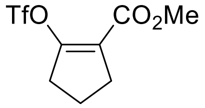

1a

Using general procedure A, *i*Pr_2NEt (12.9 mL, 73.9 mmol, 5.0 eq.), methyl 2-oxocyclopentane-1-carboxylate (2.01 g, 14.8 mmol, 1.0 eq.) and trifluoromethanesulfonic anhydride (2.98 mL, 17.7 mmol, 1.2 eq.) in CH_2Cl_2 (100 mL) gave the crude product. Purification by flash column chromatography with 70:30 hexane-Et_2O as eluent gave enol triflate **1a** (3.55 g, 92%) as an orange oil, R_F (70:30 hexane-Et_2O) 0.24; ^{1}H NMR (400 MHz, $CDCl_3$) δ 3.78 (s, 3H, OMe), 2.77-2.66 (m, 4H, CH_2), 2.01 (tt, J = 7.5, 7.5 Hz, 2H, CH_2); ^{13}C NMR (100.6 MHz, $CDCl_3$) δ 162.8 (C=O), 154.1 (=CO), 123.1 (=*C*CO_2Me), 118.4 (q, J = 320.0 Hz, CF_3), 52.0 (OMe), 32.9 (CH_2), 29.3 (CH_2), 18.9 (CH_2). Spectroscopic data are consistent with those reported in the literature.[1]

Lab Book Reference: TD 4/36

Methyl 4-oxooxolane-3-carboxylate S1

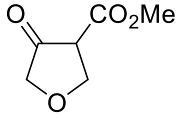

S1

Methyl 2-hydroxyacetate (7.72 mL, 100 mmol, 1,0 eq.) was added dropwise to a stirred suspension of NaH (60% in mineral oil, 4.40 g, 110 mmol, 1.1 eq.) in THF (50 mL) at 0 °C under N_2. The resulting mixture was warmed to rt and stirred at for 30 min before cooling to 0 °C. Then, a solution of methyl acrylate (9.91 mL, 110 mmol, 1.1 eq.) in anhydrous DMSO (25 mL) was added dropwise. The resulting mixture was warmed to rt and stirred at rt for 16 h. 1M $HCl_{(aq)}$ (100 mL) and Et_2O (100 mL) were added and the two layers were separated. The aqueous layer was extracted with Et_2O (3 × 100 mL) and the combined organic layers were washed with brine (4 × 50 mL), dried ($MgSO_4$) and concentrated under reduced pressure to give the crude product. Purification by flash column chromatography on silica with 50:50 hexane-Et_2O as eluent gave pyrrolidine β-keto ester **S1** (6.94 g, 48%) as a clear oil, R_F (50:50 hexane-Et_2O) 0.25; IR (ATR) 2886, 1772 (C=O, ketone), 1728 (C=O, ester), 1437, 1329, 1248, 1210, 1133, 1067 cm^{-1}; ^{1}H NMR (400 MHz, $CDCl_3$) δ 4.52–4.43 (m, 2H, OCH), 4.05 (d, J = 17.0 Hz, 1H,

OCH), 3.96 (d, J = 17.0 Hz, 1H, OCH), 3.79 (s, 3H, OMe), 3.53 (t, J = 8.5 Hz, 1H, CH); ^{13}C NMR (101 MHz, $CDCl_3$) δ 207.5 (C=O, ketone), 167.1 (C=O, ester), 70.8 (OCH_2), 69.5 (OCH_2), 53.3 (CH), 53.1 (Me); HRMS (ESI) *m/z* calcd for $C_6H_8O_4$ $(M + Na)^+$ 167.0315, found 167.0313 (+1.0 ppm error).

Lab Book Reference: JDF_B_294

1-*tert*-Butyl-4-methyl 3-oxopyrrolidine-1,4-dicarboxylate S2

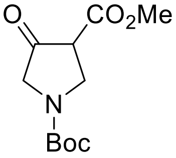

S2

Methyl acrylate (0.44 mL, 4.89 mmol, 1.0 eq.) was added dropwise to a stirred solution of dry *t*-BuOK (604 mg, 5.38 mmol, 1.1 eq.) and *N*-Boc glycine (926 mg, 4.89 mmol, 1.0 eq.) in THF at 0 °C. The resulting solution was stirred at rt for 16 h and then the solvent was evaporated under reduced pressure. The residue was dissolved in CH_2Cl_2 (50 mL) and 1 M $HCl_{(aq)}$ (10 mL) was added. The two layers were separated and the aqueous layer was extracted with CH_2Cl_2 (3 × 20 mL). The combined organic layers were dried (Na_2SO_4) and evaporated under reduced pressure to give the crude product. Purification by flash column chromatography on silica with 80:20 hexane-EtOAc as eluent gave oxopyrrolidine **S2** (830 mg, 90%) as a red oil, R_F (80:20 hexane-EtOAc) 0.35; ^{1}H NMR (400 MHz, $CDCl_3$) (50:50 mixture of keto/enol tautomers) (rotamers) δ 10.03-10.00 (m, 0.5 H, enol OH), 4.23-4.17 (m, 1H), 4.10-3.98 (m, 1.5H), 3.94-3.82 (m, 1.5H), 3.79 (s, 3H, OMe), 3.66-3.59 (m, 0.5H), 1.48 (s, 4.5H, CMe_3), 1.47 (s, 4.5H, CMe_3) ; ^{13}C NMR (100.6 MHz, $CDCl_3$) δ 172.0 (C=O, CO_2Me), 168.3 (C=O, CO_2Me), 154.1 (C=O, Boc), 97.2 (=C), 81.0 (O*C*Me_3), 80.2 (O*C*Me_3), 53.2 (OMe), 51.7 (OMe or CH_2), 51.6 (OMe or CH_2), 51.4 (CH_2), 51.1 (CH_2), 48.8 (CH_2), 48.4 (CH_2), 28.5 (C*Me*$_3$), 28.4 (C*Me*$_3$). Spectroscopic data are consistent with those reported in the literature.[2]

Lab Book Reference: TD 1/94

1-*tert*-Butyl 3-methyl 4-(trifluoromethanesulfonyloxy)-2,5-dihydro-1H-pyrrole-1,3-dicarboxylate 1b

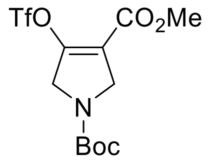

1b

Using general procedure A, *β*-ketoester **S1** (5.0 g, 19.4 mmol, 1.0 eq.), *i*Pr_2NEt (16.9 mL, 97.2 mmol, 5.0 eq.) and trifluoromethanesulfonic anhydride (3.92 mL, 23.3 mmol, 1.2 eq.) in CH_2Cl_2 (200 mL) gave the crude product. Purification by flash column chromatography on silica with 80:20 hexane-EtOAc as eluent gave enol triflate **1b** (5.18 g, 71%) as a brown solid, mp 59-62 °C; R_F (80:20 hexane-EtOAc) 0.30; IR (ATR) 1732 (C=O, CO_2Me), 1709 (C=O, Boc), 1682 (C=C), 1211, 1111 cm^{-1}; ^1H NMR (400 MHz, $CDCl_3$) δ 4.39-4.37 (m, 4H, NCH), 3.83 (s, 3H, OMe), 1.47 (s, 9H, CMe_3); ^{13}C NMR (100.6 MHz, $CDCl_3$) (rotamers) δ 160.5 (C=O, CO_2Me), 160.4 (C=O, CO_2Me), 153.5 (C=O, Boc or =CO), 153.4 (C=O, Boc or =CO), 147.6 (C=O, Boc or =CO), 147.1 (C=O, Boc or =CO), 120.0 (=C), 118.2 (q, J = 320 Hz, CF_3), 81.2 (O*C*Me_3), 52.5 (OMe), 51.5 (NCH_2), 51.3 (NCH_2), 50.7 (NCH_2), 50.3 (NCH_2), 28.5 (C*Me*$_3$); MS (ESI) *m/z* 398 [(M + Na)$^+$, 100]; HRMS (ESI) *m/z* calcd for $C_{12}H_{16}F_3NO_7S$ (M + Na)$^+$ 398.0492, found 398.0497 (–1.3 ppm error).

Lab Book Reference: TD 3/15

1-*tert*-Butyl-3-methyl 4-hydroxy-1,2,5,6-tetrahydropyridine-1,3-dicarboxylate S3

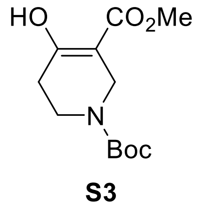

S3

Et_3N (6.33 mL, 45.5 mmol, 2.0 eq.) was added dropwise to a stirred solution of methyl 4-oxopiperidine-3-carboxylate hydrochloride (4.40 g, 22.7 mmol, 1.0 eq.) in THF (100 mL) at rt under Ar. The resulting solution was stirred at rt for 10 min. Then, di-*tert*-butyl dicarbonate (5.46 g, 25.0 mmol, 1.1 eq.) was added to the stirred solution at rt under Ar. The resulting solution was stirred at rt for 16 h and then the solvent was evaporated under reduced pressure. The residue was dissolved in CH_2Cl_2 (100 mL) and washed with H_2O (2 ×100 mL). The organic layer was dried (Na_2SO_4) and evaporated under reduced

pressure to give the crude product. Purification by flash column chromatography on silica with 80:20 hexane-EtOAc as eluent gave tetrahydropyridine **S3** (5.57 g, 95%) as a white solid, mp 46-50 °C; R_F (EtOAc) 0.67; ^{1}H NMR (400 MHz, $CDCl_3$) δ 11.96 (s, 1H, OH), 4.04 (br s, 2H, NCH_2), 3.76 (s, 3H, OMe), 3.55 (t, J = 6.0 Hz, 2H, NCH_2), 2.35 (t, J = 6.0 Hz, 2H, CH_2), 1.47 (s, 9H, CMe_3); ^{13}C NMR (100.6 Hz, $CDCl_3$) δ 171.2 (C=O, CO_2Me), 169.9 (=COH), 154.7 (C=O, Boc), 96.1 (=C), 80.2 (O*C*Me$_3$), 51.7 (OMe), 40.4 (NCH_2), 39.2 (NCH_2), 29.0 (CH_2), 28.4 (C*Me*$_3$). The crude material was sufficiently pure for use without further purification. Spectroscopic data are consistent with those reported in the literature.[3]

1-*tert*-Butyl-3-methyl 4-trifluoromethylsulfonyl-1,2,5,6-tetyrahydropyridine-1,3-dicarboxylate 1c

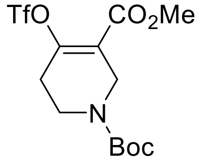

1c

Using general procedure A, *i*Pr_2NEt (18.9 mL, 108.2 mmol, 5.0 eq.), ester **S3** (5.57 g, 21.6 mmol, 1.0 eq.) and trifluoromethanesulfonic anhydride (4.37 mL, 26.0 mmol, 1.2 eq.) in CH_2Cl_2 (200 mL) gave the crude product. Purification by flash column chromatography with 80:20 hexane-EtOAc as eluent gave enol triflate **1c** (7.57 g, 90%) as a yellow solid, mp 42-45 °C; R_F (80:20 hexane-EtOAc) 0.27; IR (ATR) 1722 (C=O, CO_2Me), 1701 (C=O, Boc), 1609 (C=C), 1421, 1207 cm^{-1}; ^{1}H NMR (400 MHz, $CDCl_3$) δ 4.27 (br s, 2H, NCH_2), 3.83 (s, 3H, OMe), 3.62 (t, J = 5.5 Hz, 2H, NCH_2), 2.51 (tt, J = 5.5, 2.5 Hz, 2H, CH_2), 1.48 (s, 9H, CMe_3); ^{13}C NMR (100.6 MHz, $CDCl_3$) δ 163.0 (C=O, CO_2Me), 154.1 (C=O, Boc), 151.0 (=CO), 120.5 (=C), 118.4 (q, J = 320 Hz, CF_3), 81.2 (O*C*Me$_3$), 52.5 (OMe), 43.1 (NCH_2), 39.5 (NCH_2), 29.0 (CH_2), 28.4 (C*Me*$_3$); MS (ESI) *m/z* 412 [(M + Na)$^+$, 100]; HRMS (ESI) *m/z* calcd for $C_{13}H_{18}F_3NO_7S$ (M + Na)$^+$ 412.0648, found 412.0648 (+0.2 ppm error).

Lab Book Reference: TD 3/99

Methyl 1H-imidazole-1-carboxylate S4

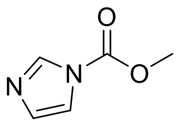

S4

Methyl chloroformate (8.50 mL, 110 mmol, 1.0 eq.) was added dropwise to a stirred solution of imidazole (15.0 g, 220 mmol, 2.0 eq.) in THF (200 mL) at 0 °C under N_2. The resulting mixture was warmed to rt and stirred at rt for 16 h. The resulting solid was removed by filtration, washed with Et_2O (200 mL) and the filtrate concentrated under reduced pressure to give the crude product. Purification by flash column chromatography on silica with EtOAc as eluent gave carbamate **S4** (11.6 g, 84%) as a white solid, R_F (EtOAc) 0.3; ^{1}H NMR (400 MHz, $CDCl_3$) δ 8.10 (br s, 1H, Ar), 7.38 (br s, 1H, Ar), 7.03 (br s, 1H, Ar), 3.99 (s, 3H, Me); ^{13}C NMR (101 MHz, $CDCl_3$) δ 149.3 (C=O), 137.2 (Ar), 130.7 (Ar), 117.2 (Ar), 54.8 (Me). Spectroscopic data consistent with those reported in the literature.[4]

Methyl 4-oxooxane-3-carboxylate S5

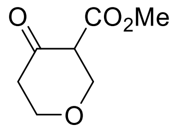

S5

LiHMDS (35.7 mL of a 1 M solution in toluene, 35.7 mmol, 2.2 eq.) was added to a stirred solution of oxan-4-one (1.50 mL, 16.2 mmol, 1.0 eq.) in toluene (70 mL) at –78 °C under N_2. The resulting solution was stirred at –78 °C for 90 mins then a solution of carbamate **S5** (2.46 g, 19.5 mmol, 1.2 eq.) in toluene (10 mL) was added dropwise. The resulting solution was stirred at 78 °C for 3 h then warmed to rt and $NH_4Cl_{(aq)}$ (20 mL) was added. EtOAc (50 mL) and 1M $HCl_{(aq)}$ (100 mL) were added and the two layers were separated. The aqueous layer was extracted with EtOAc (3 × 50 mL) and the combined organic layers were dried ($MgSO_4$) and concentrated under reduced pressure to give the crude product. Purification by flash column chromatography on silica with 80:20 hexane-Et_2O as eluent gave pyrrolidine beta-keto ester **S5** (1.47 g, 45%) as a clear oil that exists as 2:1 enol:keto forms, R_F (70:30 hexane-Et_2O) 0.26; IR (ATR) 2859, 1745 (C=O, ketone), 1719 (C=O, ester), 1666 (C=O, ester), 1630, 1443, 1311, 1242, 1221, 1195, 1101 cm^{-1}; ^{1}H NMR (400 MHz, $CDCl_3$) (enol) δ 11.77 (s, 1H, OH), 4.27 (t, J = 1.5 Hz, 2H, CH_2), 3.85 (t, J = 5.5 Hz, 2H, CH_2), 3.75 (s, 3H, Me), 2.59–2.39 (m, 2H, CH_2); (keto) δ 4.22 (dd, J = 11.5, 7.0 Hz, 1H, CH), 4.11 (dd, J = 11.5, 5.0 Hz, 1H, CH), 4.06–3.93 (m, 2H, CH), 3.77 (s, 3H, Me), 3.50 (dd, J = 6.5, 5.0 Hz, 1H, CH), 2.71–2.63 (m, 1H, CH), 2.60–2.52 (m, 1H, CH); ^{13}C NMR (101

MHz, $CDCl_3$) (mixture of keto and enol forms) δ 201.6 (C=O, ketone), 170.6 (C=O ester or C=*C*OH), 169.1 (C=O ester or C=*C*OH), 168.4 (C=O ester or C=*C*OH), 97.4 (*C*=COH), 69.7 (CH_2), 68.3 (CH_2), 64.1 (CH_2), 63.1 (CH_2), 57.8 (CH, keto), 52.7 (Me), 51.6 (Me), 42.1 (CH_2), 28.8 (CH_2); HRMS (ESI) m/z calcd for $C_7H_{10}NO_4$ $(M + Na)^+$ 181.0477, found 181.0471 (–3.2 ppm error).

Lab Book Reference: JDF_B_299

Methyl 2-(3-pyridyl)-cyclopent-1-enecarboxylate 6a

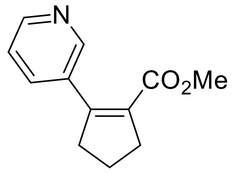

6a

Using general procedure B, enol triflate **1a** (100 mg, 0.37 mmol, 1.0 eq.), 3-pyridyl boronic acid (67 mg, 0.55 mmol, 1.5 eq.), $Pd(PPh_3)_4$ (42 mg, 0.037 mmol, 0.1 eq.) and K_2CO_3 (126 mg, 0.91 mmol, 2.5 eq.) in THF (4 mL) and H_2O (1 mL) gave the crude product. Purification by flash column chromatography with 50:50 hexane-EtOAc gave ester **6a** (26 mg, 35%) as a yellow oil, R_F (50:50 hexane-EtOAc) 0.18; IR (ATR) 2950, 1709 (C=O), 1632 (C=C), 1434, 1234 cm^{-1}; ^{1}H NMR (400 MHz, $CDCl_3$) δ 8.55 (br s, 1H, Ar), 8.51 (br d, J = 4.5 Hz, 1H, Ar), 7.67 (ddd, J = 8.0, 2.0, 2.0 Hz, 1H, Ar), 7.26 (dd, J = 8.0, 4.5 Hz, 1H, Ar), 3.62 (s, 3H, OMe), 2.88-2.82 (m, 4H, =CCH_2), 2.02 (tt, J = 8.0, 8.0 Hz, 2H, CH_2); ^{13}C NMR (400 MHz, $CDCl_3$) δ 166.1 (C=O), 150.4 (=*C*Ar), 149.0 (Ar), 148.7 (Ar), 135.4 (Ar), 132.9 (*ipso*-Ar), 130.9 (=*C*CO_2Me), 122.7 (Ar), 51.4 (OMe), 40.1 (=C*C*H_2), 35.1 (=C*C*H_2), 22.0 (CH_2); MS (ESI) *m/z* 204 [(M + H), 100]; HRMS (ESI) *m/z* calcd for $C_{12}H_{13}NO_2$ $(M + H)^+$ 204.1019, found 204.1022 (–1.3 ppm error).

Methyl 2-(3-*N*-methylpyrazole)-cyclopent-1-enecarboxylate 6b

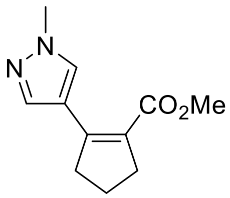

6b

Using general procedure B, enol triflate **1a** (100 mg, 0.37 mmol, 1.0 eq.), 3-(*N*-methylpyrazole)boronic acid (69 mg, 0.55 mmol, 1.5 eq.), $Pd(PPh_3)_4$ (42 mg, 0.037 mmol, 0.1 eq.) and K_2CO_3 (126 mg, 0.91 mmol, 2.5 eq.) in THF (4 mL) and H_2O (1 mL) gave the crude product. Purification by flash column chromatography with 50:50 hexane-EtOAc gave ester **6b** (66 mg, 88%) as an orange oil, R_F (50:50 hexane-EtOAc) 0.19; IR (ATR) 2950, 1708 (C=O), 1631 (C=C), 1234 cm^{-1}; ^{1}H NMR (400 MHz, $CDCl_3$) δ 8.36 (s, 1H, Ar), 7.73 (s, 1H, Ar), 3.90 (s, 3H, NMe), 3.74 (s, 3H, OMe), 2.88 (tt, J = 8.0 Hz, 2.0 Hz, 2H, =CCH_2), 2.76 (tt, J = 8.0 Hz, 2.0 Hz, 2H, =CCH_2), 1.90 (tt, J = 8.0, 8.0 Hz, 2H, CH_2); ^{13}C NMR (100.6 MHz, $CDCl_3$) δ 166.8 (C=O), 145.3 (=*C*Ar), 140.6 (Ar), 132.4 (Ar), 123.8 (=*C*CO_2Me), 117.3 (*ipso*-Ar), 51.2 (OMe), 39.2 (NMe), 38.5 (=CCH_2), 34.9 (=CCH_2), 21.6 (CH_2); MS (ESI) *m/z* 207 [(M + H), 100], 229 [(M + Na), 66]; HRMS (ESI) *m/z* calcd for $C_{11}H_{14}N_2O_2$ $(M + H)^+$ 207.1128, found 207.1126 (+0.8 ppm error).

Lab Book Reference: TD 4/42

Methyl 2-(3-thiophenyl)cyclopent-2-enecarboxylate 6c

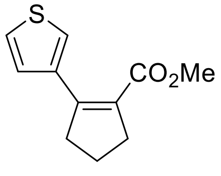

6c

Using general procedure B, enol triflate **1a** (2.00 g, 7.29 mmol, 1 eq.), 3-thiopheneboronic acid (1.40 g, 10.94 mmol, 1.5 eq.), $Pd(PPh_3)_4$ (842 mg, 0.73 mmol, 0.1 eq.) and K_2CO_3 (2.52 g, 18.23 mmol, 2.5 eq.) in THF (0 mL) and H_2O (10 mL) gave the crude product. Purification by flash column chromatography on silica with 90:10 hexane–Et_2O as eluent gave ester **6c** (1.23 g, 81%) as a yellow oil, R_F (90:10 hexane–Et_2O) 0.22; IR (ATR) 2948, 1711 (C=O), 1196 cm^{-1}; ^{1}H NMR (400 MHz, $CDCl_3$) δ 7.67 (dd, J = 3.0, 1.5 Hz, 1H, Ar), 7.37 (dd, J = 5.0, 1.5 Hz, 1H, Ar), 7.26 (dd, J = 5.0, 3.0 Hz, 1H, Ar), 3.73 (s, 3H, OMe), 2.90 (tt, J = 7.5, 2.0 Hz, 2H, =CCH_2), 2.82 (tt, J = 7.5, 2.0 Hz, 2H, =CCH_2), 1.96 (tt, J = 7.5, 7.5 Hz, 2H,

CH_2); ^{13}C NMR (100.6 MHz, $CDCl_3$) δ 167.0 (C=O), 146.6 (=*C*Ar), 136.8 (*ipso*-Ar), 128.5 (Ar), 127.5 (=*C*CO_2Me), 126.0 (Ar), 124.4 (Ar), 51.4 (OMe), 39.7 (=C*C*H_2), 35.7 (=C*C*H_2), 21.8 (CH_2); MS (ESI) *m/z* 231 [(M + Na)$^+$, 100]; HRMS (ESI) *m/z* calcd for $C_{11}H_{12}SO_2$ (M + Na)$^+$ 231.0450, found 231.0448 (+0.8 ppm error).

Lab Book Reference: TD 4/81

Methyl 3-(2-tolyl)-2,5-dihydrofuran-4-carboxylate 6d

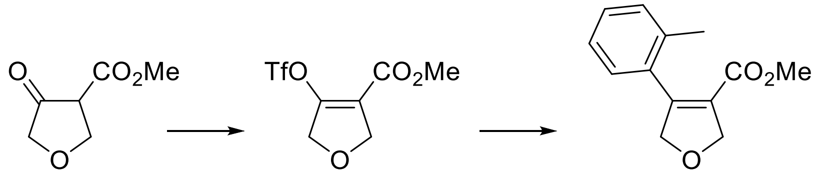

S1 **1d** **6d**

Using general procedure A, *β*-ketoester **S1** (522 mg, 3.62 mmol, 1 eq.), *i*Pr_2NEt (3.15 mL, 18.1 mmol, 5 eq.) and trifluoromethanesulfonic anhydride (0.731 mL, 4.34 mmol, 1.2 eq.) in CH_2Cl_2 (50 mL) gave the crude product. Purification by flash column chromatography with 80:20 hexane–EtOAc as eluent gave enol triflate **1d** (831 mg) as a brown oil, ^1H NMR (400 MHz, $CDCl_3$) for enol triflate **1d**: δ 4.89 (t, J = 5.0 Hz, 2H, OCH_2), 4.77 (t, J =5.0 Hz, 2H, OCH_2), 3.81 (s, 3H, OMe). Using general procedure B, crude enol triflate **1d** (831 mg, 3.0 mmol max., 1 eq.), 2-tolylboronic acid (613 mg, 4.51 mmol, 1.5 eq.), $Pd(PPh_3)_4$ (347 mg, 0.30 mmol, 0.1 eq.) and K_2CO_3 (1.04 g, 7.52 mmol, 2.5 eq.) in THF (40 mL) and H_2O (10 mL) gave the crude product. Purification by flash column chromatography on silica with 80:20 hexane-EtOAc as eluent gave a 60:40 mixture (by ^1H NMR spectroscopy) of ester **6d** and 4-fluorophenyl boronic acid (312 mg, i.e. 220 mg (28%) of ester **6d** over 2 steps) and ester **6d** (128 mg, 16% over 2 steps) as an orange oil, R_F (80:20 hexane-EtOAc) 0.10; IR (ATR) 1712 (C=O), 1648 (C=C), 1140 cm^{-1}; ^1H NMR (400 MHz, $CDCl_3$) δ 7.25-7.18 (m, 3H, Ar), 7.09-7.07 (m, 1H, Ar), 5.06 (d, J = 5.0 Hz, 1H, OCH), 5.05 (d, J = 5.0 Hz, 1H, OCH), 4.92 (d, J = 5.0 Hz, 1H, OCH), 4.91 (d, J = 5.0 Hz 1H, OCH), 3.61 (s, 3H, OMe), 2.27 (s, 3H, CMe); ^{13}C NMR (100.6 MHz, $CDCl_3$) δ 162.9 (C=O), 151.3 (=*C*Ar), 135.4 (*ipso*-Ar), 132.1 (*ipso*-Ar), 130.2 (Ar), 128.5 (Ar), 127.3 (Ar), 126.4 (=*C*CO_2Me), 125.7 (Ar), 80.6 (OCH_2), 77.4 (OCH_2), 51.6 (OMe), 19.6 (C*Me*); MS (ESI) *m/z* 219 [(M + H)$^+$, 3], 241 [(M + Na)$^+$, 100]; HRMS (ESI) *m/z* calcd for $C_{13}H_{14}O_3$ (M + Na)$^+$ 241.0835, found 241.0832 (+1.2 ppm error).

Lab Book Reference: TD 4/91

Methyl 3-(4-fluorophenyl)-2,5-dihydrofuran-4-carboxylate 6e

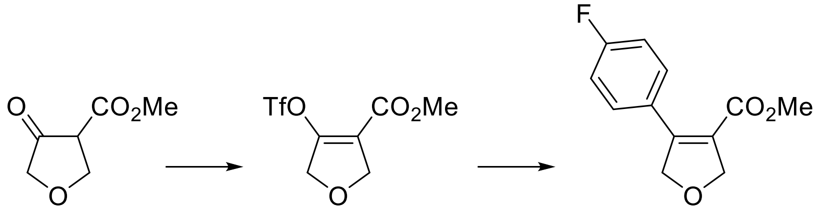

Using general procedure A, *β*-ketoester **S1** (203 mg, 1.41 mmol, 1 eq.), *i*Pr_2NEt (1.23 mL, 7.05 mmol, 5 eq.) and trifluoromethanesulfonic anhydride (0.285 mL, 1.69 mmol, 1.2 eq.) in CH_2Cl_2 (25 mL) gave the crude product. Purification by flash column chromatography with 80:20 hexane–EtOAc as eluent gave enol triflate **1d** (331 mg) as a brown oil. Using general procedure B, crude enol triflate **1d** (331 mg, 1.20 mmol max., 1 eq.), 4-fluorophenylboronic acid (252 mg, 1.80 mmol, 1.5 eq.), $Pd(PPh_3)_4$ (138 mg, 0.12 mmol, 0.1 eq.) and K_2CO_3 (414 mg, 3.00 mmol, 2.5 eq.) in THF (16 mL) and H_2O (4 mL) gave the crude product. Purification by flash column chromatography on silica with 90:10 hexane-EtOAc as eluent gave ester **6e** (168 mg, 53% over two steps) as a white solid, mp 68-70 °C, R_F (90:10 hexane-EtOAc) 0.18; IR (ATR) 1725 (C=O), 1645 (C=C), 830 cm^{-1}; ^1H NMR (400 MHz, $CDCl_3$) δ 7.51-7.47 (m, 2H, Ar), 7.09-7.05 (m, 2H, Ar), 5.06-5.00 (m, 4H OCH_2), 3.71 (s, 3H, OMe); ^{13}C NMR (100.6 MHz, $CDCl_3$) δ 164.5 (C=O), 162.7 (d, J = 250 Hz, CF), 148.4 (=*C*Ar), 130.6 (d, J = 8.5 Hz, Ar), 127.4 (d, J = 3.5 Hz, *ipso*-Ar), 124.3 (=*C*CO_2Me), 115.4 (d, J = 21.5 Hz, Ar), 79.8 (OCH_2), 77.5 (OCH_2), 51.7 (OMe); MS (ESI) *m/z* 245 [$(M + Na)^+$, 100]; HRMS (ESI) *m/z* calcd for $C_{12}H_{11}O_3F$ $(M + Na)^+$ 245.0584, found 245.0580 (+1.9 ppm error).

Lab Book Reference: TD 4/50

Methyl 3-(3-pyridyl)-2,5-dihydrofuran-4-carboxylate 6f

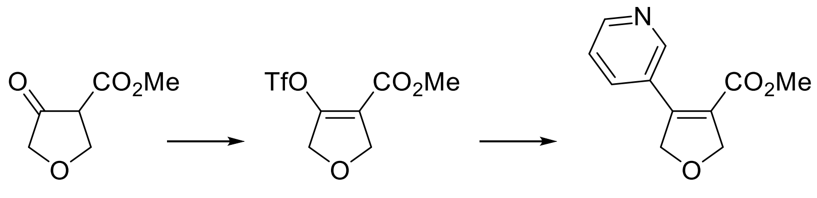

Using general procedure A, *β*-ketoester **S1** (522 mg, 3.62 mmol, 1 eq.), *i*Pr_2NEt (3.15 mL, 18.1 mmol, 5 eq.) and trifluoromethanesulfonic anhydride (0.731 mL, 4.34 mmol, 1.2 eq.) in CH_2Cl_2 (50 mL) gave the crude product. Purification by flash column chromatography with 80:20 hexane–EtOAc as eluent gave

enol triflate **1d** (847 mg) as a brown oil. Using general procedure B, crude enol triflate **1d** (847 mg, 3.07 mmol max., 1 eq.), 3-pyridylboronic acid (566 mg, 4.60 mmol, 1.5 eq.), $Pd(PPh_3)_4$ (354 mg, 0.30 mmol, 0.1 eq.) and K_2CO_3 (1.06 g, 0.30 mmol, 2.5 eq.) in THF (40 mL) and H_2O (10 mL) gave the crude product. Purification by flash column chromatography on silica with 50:50 hexane-EtOAc as eluent gave ester **6f** (184 mg, 23%) as an orange oil, R_F (50:50 hexane-EtOAc) 0.14; IR (ATR) 1718 (C=O), 1638 (C=C), 1223; ^{1}H NMR (400 MHz, $CDCl_3$) δ 8.62 (ddd, J = 2.0, 1.5, 1.5 Hz, 1H, Ar), 8.58 (ddd, J = 5.0, 1.5, 1.5 Hz, 1H, Ar), 7.87 (ddd, J = 8.0, 2.0, 1.5 Hz, Ar), 7.33 (ddd, J = 8.0, 5.0, 1.5 Hz, 1H, Ar), 5.09-5.07 (m, 2H, OCH_2), 5.05-5.03 (m, 2H, OCH_2), 3.70 (s, 3H, OMe); ^{13}C NMR (100.6 MHz, $CDCl_3$) δ 163.0 (C=O), 150.2 (Ar), 148.8 (Ar), 146.0 (=*C*Ar), 136.1 (Ar), 127.8 (*ipso*-Ar or =*C*CO_2Me), 126.6 (*ipso*-Ar or =*C*CO_2Me), 123.2 (Ar), 79.5 (OCH_2), 77.4 (OCH_2), 51.9 (OMe); MS (ESI) *m/z* 206 [(M + H)$^+$, 100]; HRMS (ESI) *m/z* calcd for $C_{11}H_{11}NO_3$ (M + H)$^+$ 206.0812, found 206.0813 (–0.7 ppm error).

Lab Book Reference: TD 4/92

Methyl 4-(2-fluorophenyl)-2,5-dihydrofuran-3-carboxylate 6g

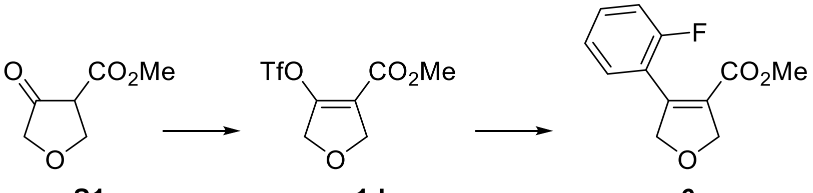

Using general procedure A, β-ketoester **S1** (3.00 g, 20.8 mmol, 1 eq.), *i*Pr_2NEt (18.1 mL, 104 mmol, 5 eq.) and trifluoromethanesulfonic anhydride (4.20 mL, 25.0 mmol, 1.2 eq.) in CH_2Cl_2 (200 mL) gave the crude product. Purification by flash column chromatography with 80:20 hexane–EtOAc as eluent gave enol triflate **1d** (3.10 g) as a brown oil. Using general procedure B, crude enol triflate **1d** (3.10 g, 11.2 mmol max., 1 eq.), 2-fluorophenylboronic acid (2.36 mg, 16.9 mmol, 1.5 eq.), $Pd(PPh_3)_4$ (1.30 mg, 1.12 mmol, 0.1 eq.) and K_2CO_3 (3.88 g, 28.1 mmol, 2.5 eq.) in THF (160 mL) and H_2O (40 mL) gave the crude product. Purification by flash column chromatography on silica with 80:20 hexane-EtOAc as eluent gave a 60:40 mixture (by ^{1}H NMR spectroscopy) of alkene **6g** and 2-fluorophenylboronic acid (2.13 g (i.e. 1.31 g (28%) over 2 steps) of alkene **6g**). ^{1}H NMR (400 MHz, $CDCl_3$) of alkene **6g**: δ 7.38-7.29 (m, 2H, Ar), 7.18-7.13 (m, 1H, Ar), 7.11-7.08 (m, 1H, Ar), 5.02-5.00 (m, 4H, OCH_2), 3.67 (s, 3H, OMe).

Lab Book Reference: TD 5/32

1-*tert*-Butyl 4-methyl 3-phenyl-2,5-dihydro-1H-pyrrole-1,4-dicarboxylate 6h

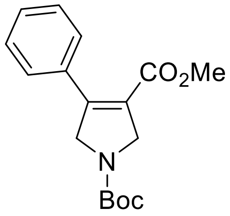

6h

Using general procedure B, enol triflate **1b** (1.00 g, 2.66 mmol, 1.0 eq.), phenylboronic acid (488 mg, 4.00 mmol, 1.5 eq.), $Pd(PPh_3)_4$ (308 mg, 0.266 mmol, 0.1 eq.) and K_2CO_3 (920 mg, 6.66 mmol, 2.5 eq.) in THF (80 mL) and H_2O (20 mL) gave the crude product. Purification by flash column chromatography with 90:10 hexane-EtOAc gave ester **6h** (666 mg, 83%) as a white solid, mp 119-122 °C; R_F (80:20 hexane-EtOAc) 0.28; IR (ATR) 1712 (C=O, CO_2Me), 1693 (C=O, Boc), 1646 (C=C), 1404, 1122 cm^{-1}; ^1H NMR (400 MHz, $CDCl_3$) (55:45 mixture of rotamers) δ 7.40-7.36 (m, 5H, Ph), 4.61-4.49 (m, 4H, NCH), 3.68 (s, 3H, OMe), 1.51 (s, 4.95H, CMe_3), 1.49 (s, 4.05H, CMe_3); ^{13}C NMR (100.6 MHz, $CDCl_3$) (rotamers) δ 174.1 (C=O, CO_2Me), 174.0 (C=O, CO_2Me), 153.9 (C=O, Boc), 148.5 (=*C*Ph), 133.0 (*ipso*-Ph), 132.9 (*ipso*-Ph), 129.3 (Ph), 129.2 (Ph), 128.3 (Ph), 128.1 (Ph), 123.6 (=*C*CO_2Me), 80.2 (O*C*Me_3), 80.1 (O*C*Me_3), 58.4 (NCH_2), 58.2 (NCH_2), 54.9 (NCH_2), 54.7 (NCH_2), 51.7 (OMe), 51.6 (OMe), 29.8 (C*Me*$_3$), 28.6 (C*Me*$_3$); MS (ESI) *m/z* 326 [(M + Na)$^+$, 100]; HRMS (ESI) *m/z* calcd for $C_{17}H_{21}NO_4$ (M + Na)$^+$ 326.1363, found 326.1369 (–1.2 ppm error).

Lab Book Reference: TD 3/86

1-*tert*-Butyl-4-methyl 3-(2-fluorophenyl)-2,5-dihydro-1H-pyrrole-1,4-dicarboxylate 6i

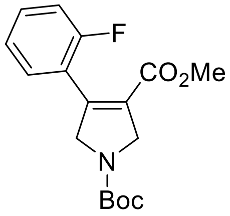

6i

Using general procedure B, enol triflate **1b** (709 g, 1.97 mmol, 1 eq.), 2-fluorophenylboronic acid (414 mg, 2.96 mmol, 1.5 eq.), $Pd(PPh_3)_4$ (228 mg, 0.20 mmol, 0.1 eq.) and K_2CO_3 (681 mg, 4.93 mmol, 2.5 eq.) in THF (16 mL) and H_2O (4 mL) gave the crude product. Purification by flash column chromatography on silica with 80:20 hexane-EtOAc as solvent gave ester **6i** (363 mg, 57%) as a yellow

solid, mp 121-124 °C; R_F (80:20 hexane-EtOAc) 0.24; IR (ATR) 1715 (C=O, CO_2Me), 1692 (C=O, Boc), 1653 (C=C), 1408, 1123 cm^{-1}; ^1H NMR (400 MHz, $CDCl_3$) (50:50 mixture of rotamers) δ 7.35-7.32 (m, 1H, Ar), 7.24-7.22 (m, 1H, Ar), 7.17-7.12 (m, 1H, Ar), 7.10-7.07 (m, 1H, Ar), 4.57-4.49 (m, 4H, NCH_2), 3.65 (s, 3H, OMe), 1.50 (s, 4.5H, CMe_3), 1.48 (s, 4.5H, CMe_3); ^{13}C NMR (100.6 MHz, $CDCl_3$) (rotamers) δ 163.3 (C=O, CO_2Me), 163.2 (C=O, CO_2Me), 159.4 (d, J = 249.0 Hz, CF), 154.0 (C=O, Boc), 153.8 (C=O, Boc), 142.7 (=CAr), 142.5 (=CAr), 130.8 (d, J = 8.5 Hz, Ar), 130.7 (d, J = 8.5 Hz, Ar), 129.8 (d, J = 2.5 Hz, Ar), 129.6 (d, J = 2.5 Hz, Ar), 127.0 (=*C*CO_2Me), 126.9 (=*C*CO_2Me), 124.0 (br s, Ar), 121.3 (d, J = 15.5 Hz, *ipso*-Ar), 115.9 (d, J = 22.0 Hz, Ar), 80.2 (O*C*Me_3), 80.1 (O*C*Me_3), 57.9 (d, J = 1.5 Hz, NCH_2), 57.7 (d, J = 1.5 Hz, NCH_2), 54.3 (NCH_2), 54.0 (NCH_2), 51.8 (OMe), 28.6 (CMe_3); MS (ESI) *m/z* 344 [(M + Na), 100]; HRMS (ESI) *m/z* calcd for $C_{17}H_{20}FNO_4$ $(M + Na)^+$ 344.1269, found 344.1271 (–0.8 ppm error).

Lab Book Reference: TD 5/23

1-*tert*-Butyl-4-methyl 3-(5-pyrimidyl)-1,2,3,6-tetrahydropyridine-1,4-dicarboxylate 6j

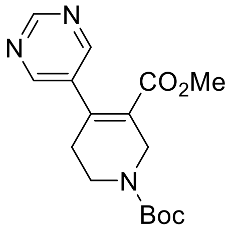

6j

$KHCO_3$ (1.07 g, 10.7 mmol, 2.5 eq.) was added portionwise to a stirred mixture of enol triflate **1c** (2.00 g, 5.36 mmol, 1.0 eq.). and 5-pyrimidylboronic acid (790 mg, 6.43 mmol, 1.5 eq.) in THF (75 mL) and H_2O (25 mL) at rt. The reaction flask was evacuated under reduced pressure and back-filled with Ar three times. Then, Pd(dppf)Cl_2 (198 mg, 0.27 mmol, 0.05 eq.) was added and the resulting mixture was stirred and heated at 65 °C for 2 h. The mixture was allowed to cool to rt and the solids were removed by filtration through Celite. H_2O (100 mL) was added and the aqueous layer was extracted with EtOAc (3 × 100 mL). The combined organic extracts were dried (Na_2SO_4) and evaporated under reduced pressure to give the crude product. Purification by flash column chromatography on silica that had been washed with 99:1 EtOAc–Et_3N with EtOAc as eluent gave ester **6j** (976 mg, 57%) as a brown oil, R_F (EtOAc) 0.26; IR (ATR) 1710 (C=O, CO_2Me), 1694 (C=O, Boc), 1625 (C=C); ^1H NMR (400 MHz, $CDCl_3$) (rotamers) δ 9.14 (s, 1H, Ar), 8.52 (s, 2H, Ar), 4.30 (br s, 2H, NCH_2), 3.63 (br t, J = 5.5 Hz, 2H, NCH_2CH_2), 3.55 (s, 3H, OMe), 2.51-2.48 (m, 2H, CH_2), 1.50 (m, 9H, CMe_3); ^{13}C NMR (100.6 MHz,

$CDCl_3$) (rotamers) δ 167.6 (C=O, CO_2Me), 158.8 (Ar), 158.5 (Ar), 157.8 (=*C*Ar), 155.6 (=*C*Ar), 155.3 (Ar), 154.6 (Ar), 140.1 (*ipso*-Ar), 139.4 (*ipso*-Ar), 125.3 (=*C*CO_2Me), 84.1 (O*C*Me_3), 53.1 (NCH_2), 52.8 (NCH_2), 51.9 (OMe), 37.8 (NCH_2), 28.5 (CH_2), 28.2 (C*Me_3*); MS (ESI) *m/z* 319 [$(M + H)^+$, 100]; HRMS (ESI) *m/z* calcd for $C_{16}H_{21}N_3O_4$ $(M + Na)^+$ 319.1532, found 319.1529 (–0.9 ppm error).

Lab Book Reference: TD 4/51

Methyl 4-(3-fluorophenyl)-5,6-dihydro-2*H*-tetrahydropyran-3-carboxylate 6k

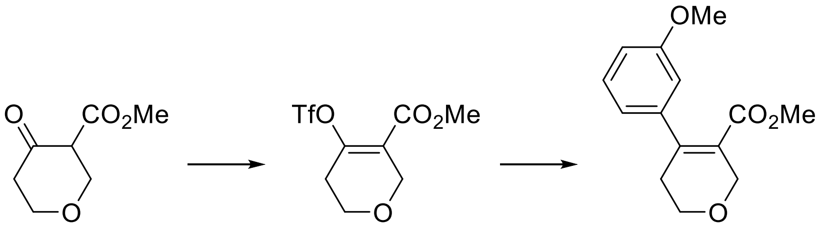

S5 **1e** **6k**

Using general procedure A, *β*-ketoester **S5** (546 mg, 3.45 mmol, 1.0 eq.), *i*Pr_2NEt (3.00 mL, 17.3 mmol, 5 eq.) and trifluoromethanesulfonic anhydride (696 μL, 4.14 mmol, 1.2 eq.) in CH_2Cl_2 (50 mL) gave the crude product. Purification by flash column chromatography with 80:20 hexane–EtOAc as eluent gave enol triflate **1e** (812 mg) as a brown oil, ^1H NMR (400 MHz, $CDCl_3$) for enol triflate **1e**: δ 4.43 (t, J = 2.5 Hz, 2H, OCH_2), 3.87 (t, J = 5.5 Hz, OCH_2), 3.80 (s, 3H, OMe), 2.52 (tt, J = 5.0, 2.5 Hz, 2H, CH_2). Using general procedure B, enol triflate **1e** (812 mg, 2.80 mmol, 1 eq.), 3-methoxyphenylboronic acid (637 mg, 4.20 mmol, 1.5 eq.), $Pd(PPh_3)_4$ (243 mg, 0.21 mmol, 0.05 eq.) and K_2CO_3 (966 mg, 7.00 mmol, 2.5 eq.) in THF (40 mL) and H_2O (10 mL) gave the crude product. Purification by flash column chromatography on silica with 80:20 hexane-EtOAc as eluent gave ester **6k** (447 mg, 52%) as a yellow oil, R_F (80:20 hexane-EtOAc) 0.15; IR (ATR) 1711 (C=O), 1642 (C=C), 1413 cm^{-1}; ^1H NMR (400 MHz, $CDCl_3$) δ 7.26 (dd, J = 8.0, 8.0 Hz, 1H, Ar), 6.84 (ddd, J = 8.0, 2.5, 1.0 Hz, 1H, Ar), 6.75-6.70 (m, 1H, Ar), 6.69 (dd, J = 2.5, 1.5 Hz, 1H, Ar), 4.44 (t, J = 2.5 Hz, 2H, OCH_2), 3.89 (t, J = 5.5 Hz, 2H, OC*H_2*CH_2), 3.80 (s, 3H, ArOMe), 3.50 (s, 3H, CO_2Me), 2.49 (tt, J = 5.5, 2.5 Hz, 2H, CH_2); ^{13}C NMR (100.6 MHz, $CDCl_3$) δ 166.7 (C=O), 159.5 (*ipso*-Ar), 146.0 (=*C*Ar), 143.0 (*ipso*-Ar), 129.3 (Ar), 126.1 (=*C*CO_2Me), 119.3 (Ar), 113.1 (Ar), 112.6 (Ar), 65.8 (O*C*H_2CH_2), 64.2 (OCH_2), 55.3 (ArO*Me*), 51.5 (CO_2*Me*), 32.4 (CH_2); MS (ESI) *m/z* 271 [$(M + Na)^+$, 100]; HRMS (ESI) *m/z* calcd for $C_{14}H_{16}O_4$ $(M + Na)^+$ 271.0941, found 271.0941 (+0.1 ppm error).

Lab Book Reference: TD 5/14

Methyl (1*R,2*S**)-2-(pyridin-3-yl)cyclopentane-1-carboxylate 2a**

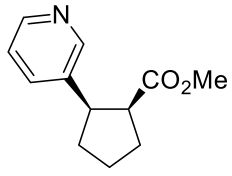

2a

Using general procedure C, cyclopentene **6a** (197 mg, 0.97 mmol, 1.0 eq.) and 10% Pd/C (20 mg, 0.02 mmol, 0.02 eq.) in MeOH (10 mL) for 40 h gave cyclopentane **2a** (130 mg, 63%) as a yellow oil, R_F (50:50 hexane-EtOAc) 0.33; IR (ATR) 2951, 1728 (C=O), 1197, 1169 cm^{-1}; ^1H NMR (400 MHz, $CDCl_3$) δ 8.45-8.41 (m, 2H, Ar), 7.50 (ddd, J = 8.0, 4.0, 2.0 Hz, 1H, Ar), 7.20-7.17 (m, 1H, Ar), 3.43-3.37 (m, 1H, C*H*Ar), 3.25 (s, 3H, OMe), 3.21-3.15 (m, 1H, C*H*CO_2Me), 2.14-1.97 (m, 5H, CH), 1.76-1.72 (m, 1H, CH); ^{13}C NMR (100.6 MHz, $CDCl_3$) δ 174.8 (C=O), 149.9 (Ar), 148.0 (Ar), 137.2 (*ipso*-Ar), 135.1 (Ar), 123.1 (Ar), 51.2 (OMe), 49.7 (*C*HCO_2Me), 46.5 (*C*HAr), 31.2 (CH_2), 28.7 (CH_2), 24.9 (CH_2); MS (ESI) *m/z* 206 [$(M + H)^+$, 100]; HRMS (ESI) *m/z* calcd for $C_{12}H_{15}NO_2$ $(M + H)^+$ 206.1176, found 206.1172 (+1.6 ppm error).

Lab Book Reference: TD 5/1

Methyl (1*R,2*S**)-2-(1-methyl-1H-pyrazol-4-yl)cyclopentane-1-carboxylate 2b**

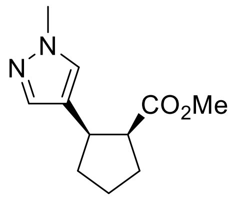

2b

Using general procedure C, cyclopentene **6b** (830 mg, 4.02 mmol, 1.0 eq.) and 10% Pd/C (83 mg, 0.08 mmol, 0.02 eq.) in MeOH (50 mL) for 112 h gave cyclopentane **2b** (775 mg, 92%) as an orange oil, IR (ATR) 2949, 1727 (C=O), 1167 cm^{-1}; ^1H NMR (400 MHz, $CDCl_3$) δ 7.26 (s, 1H, Ar), 7.12 (s, 1H, Ar), 3.82 (s, 3H, NMe), 3.42 (s, 3H, OMe), 3.33 (ddd, J = 7.5, 7.5, 7.5 Hz, 1H, C*H*Ar), 3.04 (ddd, J = 7.5, 7.5, 7.5 Hz, 1H, C*H*CO_2Me), 2.06-1.86 (m, 5H, CH), 1.71-1.64 (m, 1H, CH); ^{13}C NMR (100.6 MHz, $CDCl_3$) δ 175.3 (C=O), 138.6 (Ar), 128.2 (Ar), 121.9 (*ipso*-Ar), 51.2 (OMe), 49.6 (*C*HCO_2Me), 39.5 (*C*HAr), 38.9 (NMe), 32.8 (*C*H_2CH) , 28.0 (*C*H_2CH), 24.2 (CH_2*C*H_2CH_2); MS (ESI) *m/z* 209 [$(M + H)^+$, 100], 231 [$(M + Na)^+$, 46]; HRMS (ESI) *m/z* calcd for $C_{11}H_{16}N_2O_2$ $(M + H)^+$ 209.1285, found 209.1285 (–0.2 ppm error).

Lab Book Reference: TD 4/59

Methyl (1*R,2*S**)-2-(thiophen-3-yl)cyclopentane-1-carboxylate 2c**

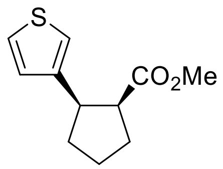

2c

Using general procedure C, cyclopentene **6c** (100 mg, 0.48 mmol, 1.0 eq.) and 10% Pd/C (10 mg, 0.009 mmol, 0.02 eq.) in MeOH (10 mL) for 40 h gave cyclopentane **2c** (70 mg, 69%) as an orange oil, IR (ATR) 2949, 1730 (C=O), 1196, 1169 cm^{-1}; ^{1}H NMR (400 MHz, $CDCl_3$) δ 7.22 (dd, J = 5.0, 3.0 Hz, 1H, Ar), 6.98-6.97 (m, 1H, Ar), 6.93 (dd, J = 5.0, 1.5 Hz, 1H, Ar), 3.49 (ddd, J = 8.0, 8.0, 8.0 Hz, 1H, C*H*Ar), 3.32 (s, 3H, OMe), 3.14-3.09 (m, 1H, C*H*CO_2Me), 2.13-1.91 (m, 5H, CH), 1.74-1.66 (m, 1H, CH), ^{13}C NMR (100.6 MHz, $CDCl_3$) 175.3 (C=O), 142.6 (*ipso*-Ar), 128.0 (Ar), 124.9 (Ar), 120.6 (Ar), 51.2 (OMe), 49.5 (*C*HCO_2Me), 44.7 (*C*HAr), 32.0 (CH_2), 28.4 (CH_2), 24.6 (CH_2)); MS (ESI) *m/z* 233 [(M + Na)$^+$, 100]; HRMS (ESI) *m/z* calcd for $C_{11}H_{14}O_2S$ (M + Na)$^+$ 233.0607, found 233.0604 (+1.0 ppm error).

Lab Book Reference: TD 4/94

Methyl (3*R,4*R**)-4-(2-methylphenyl)oxolane-3-carboxylate 2d**

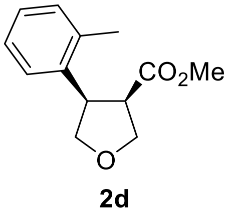

2d

Using general procedure C, dihydrofuran **6d** (69 mg, 0.32 mmol, 1.0 eq.) and 10% Pd/C (7 mg, 0.006 mmol, 0.02 eq.) in MeOH (5 mL) for 64 h gave tetrahydrofuran **2d** (45 mg, 64%) as an orange oil, IR (ATR) 2950, 1734 (C=O), 1200, 1173 cm^{-1}; ^{1}H NMR (400 MHz, $CDCl_3$) δ 7.20-7.11 (m, 4H, Ar), 4.33 (dd, J = 9.5, 6.0 Hz, 1H, OCH), 4.21-4.11 (m, 3H, OCH), 3.94-3.88 (m, 1H, C*H*Ar), 3.53 (ddd, J = 9.5, 8.0, 6.0, 1H, C*H*CO_2Me), 3.19 (s, 3H, OMe), 2.39 (s, 3H, CMe); ^{13}C NMR (100.6 MHz, $CDCl_3$) δ 172.5 (C=O), 136.9 (*ipso*-Ar), 136.4 (*ipso*-Ar), 130.3 (Ar), 127.1 (Ar), 126.6 (Ar), 126.2 (Ar), 72.6 (OCH_2), 70.0 (OCH_2), 51.4 (OMe), 48.6 (*C*HCO_2Me), 44.3 (*C*HAr), 20.0 (C*Me*); MS (ESI) *m/z* 243 [(M + Na)$^+$, 100]; HRMS (ESI) *m/z* calcd for $C_{13}H_{16}O_3$ (M + Na)$^+$ 243.0992, found 243.0991 (–0.1 ppm error).

Lab Book Reference: TD 5/5

Methyl (3*R,4*R**)-4-(pyridin-3-yl)oxolane-3-carboxylate 2f**

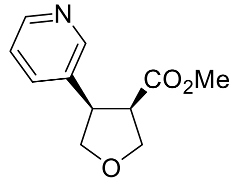

2f

Using general procedure C, dihydrofuran **6f** (184 mg, 0.89 mmol, 1.0 eq.) and 10% Pd/C (18 mg, 0.02 mmol, 0.02 eq.) in MeOH (10 mL) for 40 h gave tetrahydrofuran **2f** (177 mg, 96%) as an orange oil, IR (ATR) 2952, 2880, 1730 (C=O), 1200, 1173 cm^{-1}; ^{1}H NMR (400 MHz, $CDCl_3$) δ 8.46 (br s, 2H, Ar), 7.63-7.60 (m, 1H, Ar), 7.25-7.23 (m, 1H, Ar), 4.31 (dd, J = 9.0, 7.0 Hz, 1H, OCH), 4.18-4.09 (m, 3H, OCH), 3.75-3.70 (m, 1H, C*H*Ar), 3.55 (ddd, J = 8.5, 8.5, 7.0 Hz, 1H, C*H*CO_2Me), 3.32 (s, 3H, OMe); ^{13}C NMR (100.6 MHz, $CDCl_3$) δ 171.5 (C=O), 149.7 (Ar), 148.5 (Ar), 135.5 (Ar), 135.0 (*ipso*-Ar), 123.6 (Ar), 73.6 (OCH_2), 69.1 (OCH_2), 51.8 (OMe), 49.9 (*C*HCO_2Me), 45.7 (*C*HAr); MS (ESI) *m/z* 208 [(M + H)$^+$, 100], 230 [(M + Na)$^+$, 11]; HRMS (ESI) *m/z* calcd for $C_{11}H_{13}NO_3$ (M + H)$^+$ 208.0968, found 208.0969 (–0.5 ppm error).

Lab Book Reference: TD 5/2

Methyl (3*R,4*R**)-4-(2-fluorophenyl)oxolane-3-carboxylate 2g**

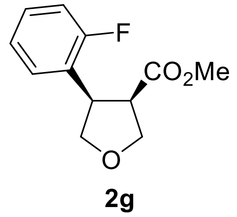

2g

Using general procedure C, a 60:40 mixture of alkene **6g** (2.13 g i.e. 1.31 g (5.90 mmol) of alkene **6g**) and 10% $Pd(OH)_2$/C (65 mg, 0.04 mmol, 0.01 eq.) in MeOH (50 mL) for 40 h gave the crude product. Purification by flash column chromatography with 80:20 hexane-EtOAc as eluent gave a 60:40 mixture (by ^{1}H NMR spectroscopy) of ester **2g** and 2-fluorophenylboronic acid (1.42 g (i.e. 870 mg (66%) of ester **2g**)). ^{1}H NMR (400 MHz, $CDCl_3$) of ester **2g**: δ 7.27-7.19 (m, 2H, Ar), 7.10-7.05 (m, 1H, Ar), 7.04-

7.00 (m, 1H, Ar), 4.31 (dd, *J* = 9.0, 6.0 Hz, 1H, OCH), 4.16-4.10 (m, 3H, OCH_2), 4.07-4.01 (m, 1H, C*H*Ar), 3.70-3.54 (m, 1H, C*H*CO_2Me), 3.30 (s, 3H, OMe).

Lab Book Reference: 5/32

1-*tert*-Butyl 3-methyl (3*R,4 *R**)-4-phenylpyrrolidine-1,3-dicarboxylate 2h**

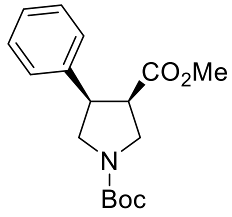

2h

Using general procedure C, dihydropyrrole **6h** (258 mg, 0.84 mmol, 1.0 eq.) and 10% Pd/C (26 mg, 0.02 mmol, 0.03 eq.) in MeOH (10 mL) for 16 h gave pyrrolidine **2h** (260 mg, 97%) as a yellow oil, R_F (80:20 hexane-EtOAc) 0.27; IR (ATR) 1735 (C=O, CO_2Me), 1691 (C=O, Boc), 1398, 1161 cm^{-1}; ^{1}H NMR (400 MHz, $CDCl_3$) (50:50 mixture of rotamers) δ 7.31-7.28 (m, 2H, Ph), 7.26-7.24 (m, 1H, Ph), 7.17-7.14 (m, 2H, Ph), 3.84-3.64 (m, 5H, NCH, C*H*Ph), 3.42-3.40 (m, 4H, OMe, C*H*CO_2Me), 1.51 (s, 4.5H, CMe_3), 1.50 (s, 4.5H, CMe_3); ^{13}C NMR (100.6 MHz, $CDCl_3$) (rotamers) δ 172.0 (C=O, CO_2Me), 171.8 (C=O, CO_2Me), 154.5 (C=O, Boc), 138.7 (*ipso*-Ph), 138.5 (*ipso*-Ph), 128.6 (Ph), 127.6 (Ph), 127.5 (Ph), 79.9 (O*C*Me_3), 51.6 (OMe), 50.5 (NCH_2), 50.4 (NCH_2), 48.9 (*C*HCO_2Me), 48.1 (*C*HCO_2Me), 47.2 (NCH_2), 47.1 (NCH_2), 46.5 (*C*HPh), 45.6 (*C*HPh), 28.7 (C*Me*$_3$); MS (ESI) *m/z* 328 [$(M + Na)^+$, 100]; HRMS (ESI) *m/z* calcd for $C_{17}H_{23}NO_4$ $(M + Na)^+$ 328.1519, found 328.1520 (–0.6 ppm error).

Lab Book Reference: TD 3/69

1-*tert*-Butyl 3-methyl (3*R,4 *R**)-4-(2-fluorophenyl)pyrrolidine-1,3-dicarboxylate 2i**

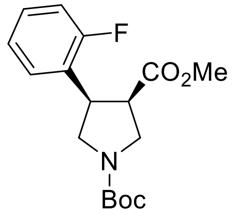

2i

Using general procedure C, ester **6i** (338 mg, 1.04 mmol, 1.0 eq.) and 10% Pd/C (34 mg, 0.03 mmol, 0.03 eq.) in MeOH (10 mL) for 40 h gave ester **2i** (339 mg, quant.) as a white solid, mp 96–100 °C; IR (ATR) 1736 (C=O, CO_2Me), 1693 (C=O, Boc), 1401, 1165 cm^{-1}; ^1H NMR (400 MHz, $CDCl_3$) δ 7.24-7.19 (m, 1H, Ar), 7.16-7.11 (m, 1H, Ar), 7.05 (ddd, *J* = 7.5, 7.5, 1.0 Hz, 1H, Ar), 7.00 (br dd, *J* = 9.5, 9.5 Hz, 1H, Ar), 3.93 (ddd, *J* = 7.5, 7.5, 7.5 Hz, 1H, C*H*Ar or C*H*CO_2Me), 3.82-3.62 (m, 4H, NCH, C*H*Ar or C*H*CO_2Me), 3.47-3.45 (m, 1H, NCH), 3.36 (s, 3H, OMe), 1.48 (br s, 9H, CMe_3); ^{13}C NMR (100.6 MHz, $CDCl_3$) (rotamers) δ 172.2 (C=O, *C*O_2Me) 172.1 (C=O, *C*O_2Me), 160.4 (d, *J* = 247 Hz, CF), 154.4 (C=O, Boc), 128.9 (d, *J* = 8.5 Hz, Ar), 127.7 (d, *J* = 4.0 Hz, Ar), 125.2 (d, *J* = 14.5 Hz, *ipso*-Ar), 125.0 (d, *J* = 14.5 Hz, *ipso*-Ar), 124.3 (d, *J* = 2.5 Hz, Ar), 124.2 (d, *J* = 2.5 Hz, Ar), 115.3 (d, *J* = 5.5 Hz, Ar), 115.1 (d, *J* = 5.5 Hz, Ar), 79.8 (O*C*Me_3) 51.6 (OMe), 48.8 (NCH_2), 48.7 (NCH_2), 47.7 (NCH_2), 47.6 (*C*HCO_2Me), 46.7 (*C*HCO_2Me), 39.2 (C*H*Ar), 38.3 (C*H*Ar), 28.5 (C*Me*$_3$); MS (ESI) *m/z* 346 [(M + Na), 100]; HRMS (ESI) *m/z* calcd for $C_{17}H_{22}FNO_4$ $(M + Na)^+$ 346.1425, found 346.1425 (–0.1 ppm error).

Lab Book Reference: TD 5/27

1-*tert*-Butyl 3-methyl (3*R,4 *R**)-4-(pyrimidin-5-yl)piperidine-1,3-dicarboxylate 2j**

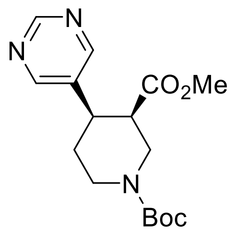

2j

Using general procedure C, tetrahydropyridine **6j** (1.94 g, 6.07 mmol, 1.0 eq.) and 10% Pd/C (46 mg, 0.04 mmol, 0.01 eq.) in MeOH (50 mL) for 40 h gave piperidine **2j** (1.93 g, 99%) as a yellow oil, IR (ATR) 1733 (C=O, CO_2Me), 1687 (C=O, Boc), 1415, 1156 cm^{-1}; ^1H NMR (400 MHz, $CDCl_3$) δ 9.09 (s, 1H, Ar), 8.66 (s, 2H, Ar) 4.60-4.28 (m, 2H, NCH), 3.53 (s, 3H, OMe), 3.21-3.13 (m, 1H, NCH), 3.02-

2.99 (m, 1H, NCH), 2.98 (ddd, *J* = 12.5, 4.0, 4.0 Hz, 1H, C*H*Ar), 2.97-2.87 (m, 2H, NCH, C*H*CO_2Me), 2.10 (m, 1H, CH), 1.73-1.62 (m, 1H, CH), 1.45 (s, 9H, CMe_3); ^{13}C NMR (100.6 MHz, $CDCl_3$) δ 171.4 (C=O, CO_2Me), 157.4 (Ar), 156.5 (Ar), 155.4 (C=O, Boc), 135.7 (*ipso*-Ar), 80.2 (O*C*Me_3), 51.8 (OMe), 46.9 (NCH_2), 45.0 (NCH_2 or *C*HCO_2Me) , 44.7 (NCH_2 or *C*HCO_2Me), 39.4 (*C*HAr), 28.5 (C*Me*$_3$), 24.9 (CH_2); MS (ESI) *m/z* 322 [(M + H)$^+$, 12], 344 [(M + Na)$^+$, 100]; HRMS (ESI) *m/z* calcd for $C_{16}H_{23}N_3O_4$ (M + Na)$^+$ 344.1581, found 344.1573 (+2.1 ppm error).

Lab Book Reference: TD 5/25

Methyl (3*R,4*R**)-4-(3-methoxyphenyl)oxane-3-carboxylate 2k**

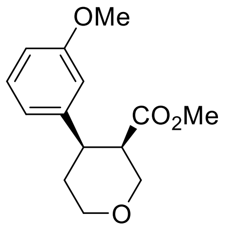

2k

Using general procedure C, dihydropyran **6k** (57 mg, 0.23 mmol, 1.0 eq.) and 10% Pd/C (6 mg, 0.006 mmol, 0.02 eq.) in MeOH (5 mL) for 16 h gave tetrahydropyran **2k** (47 mg, 82%) as an orange oil, IR (ATR) 2955, 2841, 1738 (C=O), 1165 cm^{-1}; ^{1}H NMR (400 MHz, $CDCl_3$) δ 7.22 (dd, *J* = 8.0, 8.0 Hz, 1H, Ar), 6.87-6.83 (m, 1H, Ar), 6.82-6.77 (m, 1H, Ar), 6.75 (dd, *J* = 8.0, 2.5 Hz, 1H, Ar), 4.29 (br d, *J* = 11.5 Hz, 1H, OCH), 4.19 (ddd, *J* = 11.5, 4.0, 1.5 Hz, 1H, OCH), 3.80 (s, 3H, ArO*Me*), 3.75 (dd, *J* = 11.5, 3.0 Hz, 1H, OCH), 3.55 (ddd, *J* = 11.5, 11.5, 2.5 Hz, 1H, OCH), 3.52 (s, 3H, CO_2Me), 3.05 (ddd, *J* = 12.5, 4.0, 4.0 Hz, 1H, C*H*Ar), 2.91 (br s, 1H, C*H*CO_2Me), 2.72 (dddd, 1H, *J* = 12.5, 12.5, 11.5, 4.0 Hz, 1H, OCH_2C*H*), 1.71 (br dd, 1H, *J* = 12.5, 1.5 Hz, OCH_2C*H*); ^{13}C NMR (100.6 MHz, $CDCl_3$) 172.4 (C=O), 159.6 (*ipso*-Ar), 144.4 (*ipso*-Ar), 129.4 (Ar), 119.8 (Ar), 113.6 (Ar), 111.7 (Ar), 69.9 (O*C*H_2CH), 68.6 (O*C*H_2CH_2), 55.3 (ArO*Me*), 51.5 (CO_2*Me*), 46.5 (*C*HCO_2Me), 41.8 (*C*HAr), 26.7 (OCH_2*C*H_2); MS (ESI) *m/z* 251 [(M + H)$^+$, 11] 273 [(M + Na)$^+$, 100]; HRMS (ESI) *m/z* calcd for $C_{14}H_{18}O_4$ (M + Na)$^+$ 273.1097, found 273.1084 (+4.7 ppm error).

Lab Book Reference: TD 5/18

2-(Thiophen-3-yl)cyclopent-1-ene-1-carboxylic acid S6

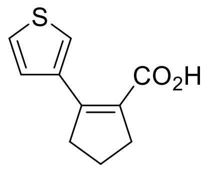

S6

2 M $NaOH_{(aq)}$ (3 mL) was added dropwise to a stirred solution of ester **6c** (150 mg, 0.72 mmol, 1 eq.) in MeOH (3mL) and THF (3 mL) at rt under Ar. The resulting mixture was stirred and heated at 70 °C for 1 h. The mixture was then allowed to cool to rt and H_2O (15 mL) was added. The mixture was washed with CH_2Cl_2 (10 mL) and 1 M $HCl_{(aq)}$ (10 mL) was added. The mixture was extracted with CH_2Cl_2 (2 × 50 mL) and the combined organic extracts were dried (Na_2SO_4) and evaporated under reduced pressure to give acid **S6** (115 mg, 84%) as a white solid, mp 116-119 °C; IR (ATR) 2942, 1669 (C=O), 1648 (C=C), 1594, 1268 cm^{-1}; ^1H NMR (400 MHz, $CDCl_3$) δ 7.71 (dd, J = 3.0, 1.0 Hz, 1H, Ar), 7.41 (dd, J = 5.0, 1.0 Hz), 7.25 (dd, J = 5.0, 3.0 Hz, 1H, Ar), 2.95 (tt, J = 7.5, 2.0 Hz, 2H, $=CCH_2$), 2.86 (tt, J = 7.5, 2.0 Hz, 2H, $=CCH_2$), 1.94 (tt, J = 7.5, 7.5 Hz, 2H, CH_2); ^{13}C NMR (100.6 MHz, $CDCl_3$) δ 171.4 (C=O), 149.7 (=*C*Ar), 136.4 (*ipso*-Ar), 128.8 (Ar), 126.8 (Ar), 126.7 (=*C*CO_2H), 124.5 (Ar), 40.4 (=C*C*H_2), 35.5 (=C*C*H_2), 21.7 (CH_2); HRMS (ESI) *m/z* calcd for $C_{10}H_{10}O_2S$ $(M + Na)^+$ 217.0294, found 217.0292 (+1.0 ppm error).

Lab Book Reference: TD 4/93

(1*R,2*S**)-2-(Thiophen-3-yl)cyclopentane-1-carboxylic acid 7c**

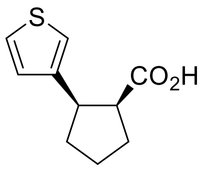

7c

Using general procedure C, cyclopentene **S6** (200 mg, 1.03 mmol, 1.0 eq.) and 10% Pd/C (20 mg, 0.014 mmol, 0.01 eq.) in MeOH (10 mL) for 40 h gave cyclopentane **7c** (188 mg, 93%) as a white solid, mp 75-77 °C; IR (ATR) 2953 (br, OH), 1699 (C=O), 1233, 787 cm^{-1}; ^{1}H NMR (400 MHz, $CDCl_3$) δ 7.21 (dd, J = 5.0, 3.0 Hz, 1H, Ar), 6.99-6.95 (m, 1H, Ar), 6.94 (dd, J = 5.0, 1.0 Hz, 1H, Ar), 3.48 (ddd, J = 8.0, 8.0, 8.0 Hz, 1H, C*H*Ar), 3.08 (ddd, J = 8.0, 8.0, 6.0 Hz, 1H, C*H*CO_2H), 2.08-1.92 (m, 5H, CH), 1.75-1.69 (m, 1H, CH); ^{13}C NMR (100.6 MHz, $CDCl_3$) δ 180.1 (C=O), 142.1 (*ipso*-Ar), 127.9 (Ar), 125.1 (Ar), 120.8 (Ar), 49.4 (*C*HCO_2H), 44.7 (*C*HAr), 31.9 (CH_2), 28.3 (CH_2), 24.2 (CH_2); MS (ESI) *m/z* 219 [(M + Na)$^+$, 100]; HRMS (ESI) *m/z* calcd for $C_{10}H_{12}O_2S$ (M + Na)$^+$ 219.0450, found 219.0449 (+0.6 ppm error). The structure was confirmed by X-Ray crystallography (CCDC 2096673):

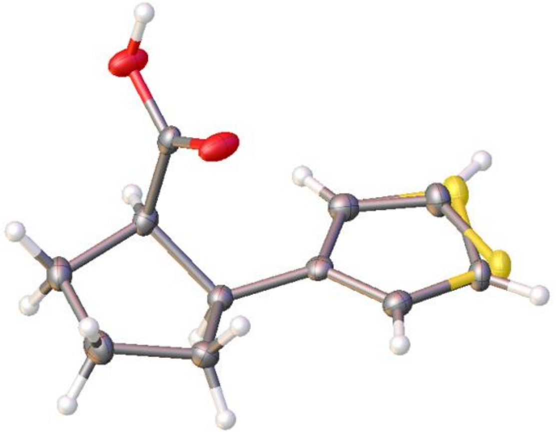

Lab Book Reference: TD 4/98

4-(2-Methylphenyl)-2,5-dihydrofuran-3-carboxylic acid S7

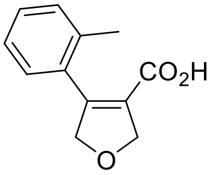

S7

2 M NaOH(aq) (1 mL) was added dropwise to a stirred solution of a 60:40 mixture of ester **6d** and 2-tolylboronic acid (72 mg, i.e. 50 mg (0.23 mmol, 1 eq.) of ester **6d**) in MeOH (1 mL) and THF (1 mL) at rt under Ar. The mixture was stirred and heated at 70 °C for 1 h. The mixture was then allowed to cool to rt and H_2O (10 mL) was added. The mixture was washed with CH_2Cl_2 (5 ml) and 1 M HCl(aq) (5 mL) was added. The mixture was extracted with CH_2Cl_2 (2 × 20 mL) and the combined organic extracts were dried (Na_2SO_4) and evaporated under reduced pressure to give acid **S7** (36 mg, 78%) as a yellow oil, IR (ATR) 2861 (br, OH), 1685 (C=O), 1272, 754 cm^{-1}; ^{1}H NMR (400 MHz, $CDCl_3$) δ 9.07 (br s, 1H, CO_2H) 7.26-7.16 (m, 3H, Ar), 7.06 (d, J = 7.5 Hz, 1H, Ar), 5.02 (t, J = 5.0 Hz, 2H, OCH_2), 4.92 (t, J = 5.0 Hz, 2H, OCH_2), 2.25 (s, 3H, C*Me*); ^{13}C NMR (100.6 MHz, $CDCl_3$) δ 167.5 (C=O), 153.5 (=*C*CO_2H), 135.3 (*ipso*-Ar or *C*Me), 131.7 (*ipso*-Ar or *C*Me), 130.3 (Ar), 128.6 (Ar), 127.1 (Ar), 126.1 (=*C*Ar), 125.7 (Ar), 80.9 (OCH_2), 76.5 (OCH_2), 19.6 (C*Me*); MS (ESI) *m/z* 195 [(M – H)$^-$, 100]; HRMS (ESI) *m/z* calcd for $C_{12}H_{12}O_3$ (M – H)$^-$ 203.0714, found 203.0709 (+2.3 ppm error).

Lab Book Reference: TD 5/3

(3*R,4*R**)-4-(2-Methylphenyl)oxolane-3-carboxylic acid 7d**

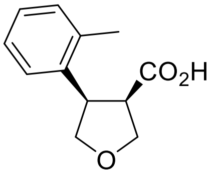

7d

Using general procedure C, dihydrofuran **S7** (189 mg, 0.925 mmol, 1.0 eq.) and 10% $Pd(OH)_2/C$ (19 mg, 0.02 mmol, 0.02 eq.) in MeOH (10 mL) for 112 h gave the crude product. Purification by flash column chromatography with 79:20:1 hexane-EtOAc-AcOH as eluent gave tetrahydrofuran **7d** (122 mg, 46%) as an orange oil, R_F (80:20:1 hexane-EtOAc-AcOH) 0.09; IR (ATR) 2980 (br, OH), 1736 (C=O), 1180 cm^{-1}; ^{1}H NMR (400 MHz, $CDCl_3$) δ 7.19-7.09 (m, 4H, Ar), 4.27 (dd, J = 9.0, 6.0 Hz, 1H, OCH), 4.19-4.10 (m, 3H, OCH), 3.94-3.88 (m, 1H, C*H*Ar), 3.54-3.49 (m, 1H, C*H*CO_2Me), 2.37 (s, 3H, CMe); ^{13}C

NMR (100.6 MHz, $CDCl_3$) δ 175.8 (C=O), 137.0 (*ipso*-Ar), 136.0 (*ipso*-Ar), 130.4 (Ar), 127.2 (Ar), 126.7 (Ar), 126.3 (Ar), 72.8 (OCH_2), 70.1 (OCH_2), 48.2 (*C*HCO_2Me), 44.3 (*C*HAr), 20.1 (C*Me*); MS (ESI) *m/z* 205 [$(M-H)^-$, 100]; HRMS (ESI) *m/z* calcd for $C_{12}H_{14}O_3$ $(M-H)^-$ 205.0870, found 205.0878 (–3.9 ppm error).

Lab Book Reference: TD 5/19

4-(4-Fluorophenyl)-2,5-dihydrofuran-3-carboxylic acid S8

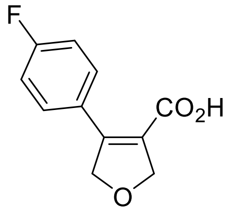

S8

LiOH (41 mg, 2.03 mmol, 3.0 eq.) was added portionwise to a stirred solution of ester **6e** (150 mg, 0.68 mmol, 1 eq.) in 4:1:1 THF–H_2O–MeOH (36 mL) at rt under Ar. The mixture stirred at rt for 2 h. Then, the solvent was evaporated under reduced pressure and H_2O (10 mL) was added. The mixture was washed with CH_2Cl_2 (10 mL) and 1 M $HCl_{(aq)}$ (10 mL) was added. The mixture was extracted with CH_2Cl_2 (2 × 20 mL) and the combined organic extracts were dried (Na_2SO_4) and evaporated under reduced pressure to give acid **S8** (110 mg, 78%) as a yellow oil, IR (ATR) 1678 (C=O), 1439 (C=C), 1220, 751 cm^{-1}; ^1H NMR (400 MHz, $CDCl_3$) δ 7.49-7.45 (m, 2H, Ar), 7.09-7.05 (m, 2H, Ar), 5.08-5.02 (m, 4H, OCH_2); ^{13}C NMR (100.6 MHz, $CDCl_3$) δ 167.8 (C=O), 163.4 (d, J = 250 Hz, CF), 151.1 (=*C*Ar), 130.6 (d, J = 8.5 Hz, Ar), 127.0 (d, J = 3.5 Hz, *ipso*-Ar), 123.7 (=*C*CO_2H), 115.5 (d, J = 22.0 Hz, Ar), 80.2 (OCH_2), 77.4 (OCH_2); MS (ESI) *m/z* 207 [$(M-H)^-$, 100]; HRMS (ESI) *m/z* calcd for $C_{11}H_8FO_3$ $(M-H)^-$ 207.0463, found 207.0466 (–1.4 ppm error).

Lab Book Reference: TD 4/57

(3*R,4*R**)-4-(4-Fluorophenyl)oxolane-3-carboxylic acid 7e**

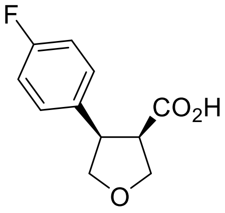

7e

Using general procedure C, dihydrofuran **S8** (102 mg, 0.49 mmol, 1.0 eq.) and 10% Pd/C (11 mg, 0.01 mmol, 0.02 eq.) in MeOH (10 mL) for 16 h gave the crude product. Purification by flash column chromatography on silica with 69:30:1 hexane-EtOAc-AcOH as eluent gave tetrahydrofuran **7e** (60 mg, 58%) as a white solid, mp 65-67 °C; R_F (69:30:1 hexane-EtOAc-AcOH) 0.11; IR (ATR) 2959 (br, OH), 2890, 1731 (C=O), 1709, 1511, 1224 cm^{-1}; ^{1}H NMR (400 MHz, $CDCl_3$) δ 7.20-7.17 (m, 2H, Ar), 6.97-6.93 (m, 2H, Ar), 4.20 (dd, J = 7.5, 7.5 Hz, 1H, OCH), 4.17-4.06 (m, 3H, OCH), 3.72-3.67 (m, 1H, C*H*Ar), 3.46 (ddd, J = 7.5, 7.5, 7.5 Hz, 1H, C*H*CO_2H); ^{13}C NMR (100.6 MHz, $CDCl_3$) δ 176.4 (C=O), 162.2 (d, J = 246.0 Hz, CF), 134.1 (d, J = 3.0 Hz, *ipso*-Ar), 129.8 (d, J = 8.0 Hz, Ar), 115.3 (d, J = 21.0 Hz, Ar), 73.7 (OCH_2), 68.9 (OCH_2), 49.8 (*C*HAr), 47.4 (*C*HCO_2H); MS (ESI) *m/z* 209 [(M – H)$^-$, 100]; HRMS (ESI) *m/z* calcd for $C_{11}H_{10}FO_3$ (M – H)$^-$ 209.0619, found 209.0618 (+0.7 ppm error). The structure was confirmed by X-Ray crystallography (CCDC 2096674):

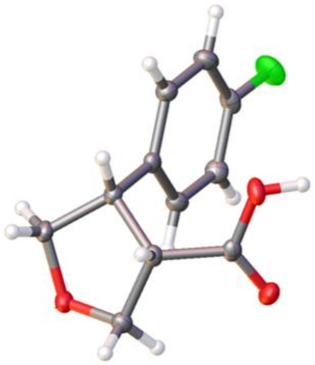

Lab Book Reference: TD 4/64

4-(3-Methoxyphenyl)-5,6-dihydro-2*H*-pyran-3-carboxylic acid S9

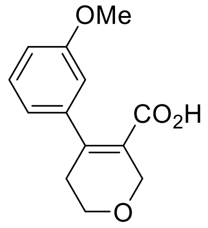

S9

2 M $NaOH_{(aq)}$ (12 mL) was added dropwise to a stirred solution of ester **6k** (590 mg, 2.38 mmol, 1 eq.) in MeOH (12 mL) and THF (12 mL) at rt under Ar. The resulting mixture was stirred and heated at 70 °C for 1 h. The mixture was then allowed to cool to rt and H_2O (25 mL) was added. The mixture was washed with CH_2Cl_2 (25 mL) and then 1 M $HCl_{(aq)}$ (50 mL) was added. The mixture was extracted with CH_2Cl_2 (2 × 50 mL) and the combined organic extracts were dried (Na_2SO_4) and evaporated under reduced pressure to give acid **S9** (481 mg, 86%) as a white solid, mp 101-103 °C; IR (ATR) 1675 (C=O), 1446 (C=C), 832 cm^{-1}; ^{1}H NMR (400 MHz, $CDCl_3$) δ 7.23 (dd, J = 8.0, 8.0 Hz, 1H, Ar), 6.82 (ddd, J = 8.0, 2.5, 1.0 Hz), 6.73 (ddd, J = 8.0, 1.5, 1.0 Hz, 1H, Ar), 6.68 (ddd, J = 2.5, 1.5 Hz), 4.41 (t, J = 2.5 Hz, 2H, OCH_2), 3.85 (t, J = 5.5 Hz, 2H, OCH_2CH_2), 3.79 (s, 3H, OMe), 2.49 (tt, J = 5.5, 2.5 Hz, 2H, CH_2); ^{13}C NMR (100.6 MHz, $CDCl_3$) δ 170.4 (C=O), 159.5 (*ipso*-Ar), 149.3 (=*C*Ar), 142.6 (*ipso*-Ar), 129.5 (Ar), 125.1 (=CCO_2H), 119.3 (Ar), 113.3 (Ar), 112.7 (Ar), 65.7 (OCH_2), 64.1 (OCH_2CH_2), 55.4 (OMe), 33.2 (CH_2); MS (ESI) *m/z* 257 [$(M + Na)^+$, 100]; HRMS (ESI) *m/z* calcd for $C_{13}H_{14}O_4$ $(M + Na)^+$ 257.0784, found 257.0783 (+0.3 ppm error).

Lab Book Reference: TD 5/17

(3*R,4*R**)-4-(3-Methoxyphenyl)oxane-3-carboxylic acid 7k**

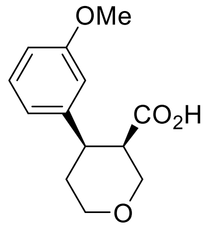

7k

Using general procedure C, dihydropyran **S9** (460 mg, 1.94 mmol, 1.0 eq.) and 10% $Pd(OH)_2/C$ (46 mg, 0.03 mmol, 0.02 eq.) in MeOH (50 mL) for 64 h gave tetrahydropyran **7k** (449 mg, 98%) as a white solid, mp 111-113 °C; IR (ATR) 2965 (br, OH), 2930, 1703 (C=O), 1165 cm^{-1}; ^{1}H NMR (400 MHz, $CDCl_3$) δ 9.47 (br s, 1H, CO_2H), 7.21 (dd, J = 8.0, 8.0 Hz, 1H, Ar), 6.86-6.82 (m, 1H, Ar), 6.82-6.77 (m,

1H, Ar), 6.77-6.75 (m, 1H, Ar), 4.32 (br dd, *J* = 12.0, 1.5 Hz, 1H, OCH), 4.17 (ddd, *J* = 11.5, 4.5, 1.5 Hz, 1H, OCH), 3.77-3.72 (m, 4H, OMe, OCH), 3.55 (ddd, *J* = 11.5, 11.5, 2.5 Hz, 1H, OCH), 3.04 (ddd, *J* = 12.0, 4.0, 4.0 Hz, 1H, C*H*Ar), 2.92-2.88 (m, 1H, C*H*CO_2H), 2.66 (dddd, 1H, *J* = 12.5, 12.0, 11.5, 4.0 Hz, 1H, OCH_2C*H*), 1.70 (m, 1H, OCH_2C*H*); ^{13}C NMR (100.6 MHz, $CDCl_3$) δ 177.6 (C=O), 159.6 (*ipso*-Ar), 143.9 (*ipso*-Ar), 129.3 (Ar), 119.8 (Ar), 113.4 (Ar), 111.8 (Ar), 69.8 (O*C*H_2CH), 68.6 (O*C*H_2CH_2), 55.2 (OMe), 46.3 (*C*HCO_2H), 41.4 (*C*HAr), 26.5 (OCH_2*C*H_2); MS (ESI) *m/z* 235 [(M – H)$^-$, 11]; HRMS (ESI) *m/z* calcd for $C_{13}H_{16}O_4$ (M – H)$^-$ 235.0976, found 235.0975 (+0.4 ppm error).

Lab Book Reference: TD 5/26

1-(*tert*-Butyl) 4-methyl piperidine-1,4-dicarboxylate 3a

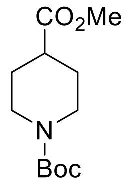

3a

Potassium carbonate (724 mg, 5.24 mmol, 3.0 eq.) was added to a stirred solution of piperidine-4-carboxylic acid (400 mg, 1.75 mmol, 1.0 eq.) in DMF (10 mL) at rt. The resulting suspension was stirred at rt for 30 min and methyl iodide (0.33 mL, 5.24 mmol, 3.0 eq.) was added. The mixture was then stirred at rt for 18 h. Water (3 mL) was added and the mixture was extracted with EtOAc (4 × 10 mL). The combined organic were washed with brine (4 × 80 mL), dried ($MgSO_4$) and evaporated under reduced pressure to give *N*-Boc piperidine-4-methyl ester **3a** (399 mg, 94%) as a colourless oil, IR (ATR) 2953, 1734 (C=O, CO_2Me), 1689 (C=O, Boc), 1419, 1158, 1038 cm^{-1}; ^{1}H NMR (400 MHz, $CDCl_3$) δ 4.02-3.98 (m, 2H, NCH), 3.67 (s, 3H, OMe), 2.84-2.78 (m, 2H, NCH), 2.43 (tt, *J* = 11.0, 4.0 Hz, 1H, CH), 1.87-1.79 (m, 2H, CH), 1.60 (dddd, *J* = 13.5, 11.0, 11.0, 4.0 Hz, 2H, CH), 1.43 (s, 9H, CMe_3); ^{13}C NMR (100.6 MHz, $CDCl_3$) δ 175.2 (C=O, CO_2Me), 154.8 (C=O, Boc), 79.7 (O*C*Me_3), 51.9 (OMe), 43.3 (NCH_2), 41.1 (CH), 28.5 (CH_2), 28.1 (C*Me_3*); MS (ESI) *m/z* 266 (M + Na)$^+$; HRMS *m/z* calcd for $C_{12}H_{21}NO_4$ (M + Na)$^+$ 266.1363, found 266.1359 (+1.3 ppm error). Spectroscopic data consistent with those reported in the literature.[5]

Lab Book Reference: PJ-06-86.

1-(*tert*-Butyl) 3-methyl piperidine-1,3-dicarboxylate 3b

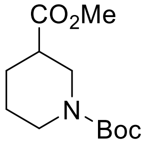

3b

Potassium carbonate (724 mg, 5.24 mmol, 3.0 eq.) was added to a stirred solution of *N*-Boc piperidine-3-carboxylic acid (400 mg, 1.75 mmol, 1.0 eq.) in DMF (10 mL) at rt. The resulting suspension was stirred at rt for 30 min and methyl iodide (0.33 mL, 5.24 mmol, 3.0 eq.) was added. The mixture was then stirred at rt for 18 h. Water (3 mL) was added and the mixture was extracted with EtOAc (4 × 10 mL). The combined organic were washed with brine (4 × 80 mL), dried ($MgSO_4$) and evaporated under reduced pressure to give *N*-Boc piperidine-3-methyl ester **3b** (383 mg, 90%) as a white solid, mp 38-40 °C; IR (ATR) 2952, 1734 (C=O, CO_2Me), 1690 (C=O, Boc), 1421, 1142 cm^{-1}; ^1H NMR (400 MHz, $CDCl_3$) δ 4.24-3.97 (m, 1H, NCH), 3.92-3.89 (m, 1H, NCH), 3.68 (s, 3H, OMe), 2.97-2.84 (m, 1H, NCH), 2.79 (ddd, J = 13.5, 11.0, 3.0 Hz, 1H, NCH), 2.48-2.41 (m, 1H, CH), 2.05-2.01 (m, 1H, CH), 1.71-1.67 (m, 1H, CH), 1.64-1.58 (m, 1H, CH), 1.45-1.37 (m, 10H, CMe_3 and CH); ^{13}C NMR (100.6 MHz, $CDCl_3$) δ 174.1 (C=O, CO_2Me), 154.8 (C=O, Boc), 79.8 (O*C*Me₃), 51.9 (OMe), 46.0 (NCH_2), 44.0 (NCH_2), 41.5 (CH), 28.5 (C*Me₃*), 27.5 (CH_2), 24.4 (CH_2); MS (ESI) *m/z* 266 $(M + Na)^+$; HRMS *m/z* calcd for $C_{12}H_{21}NO_4$ $(M + Na)^+$ 266.1363, found 266.1360 (0.8 ppm error). Spectroscopic data consistent with those reported in the literature.[6]

Lab Book Reference: PJ-06-90.

1-(*tert*-Butyl) 3-methyl pyrrolidine-1,3-dicarboxylate 3c

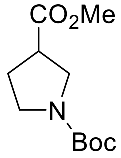

3c

A solution of Boc_2O (1.04 g, 4.8 mmol, 2.0 eq.) in CH_2Cl_2 (7.5 mL) was added dropwise to a stirred solution of pyrrolidine-3-methyl ester (400 mg, 2.4 mmol, 1.0 eq.), and Et_3N (1.0 mL, 4.8 mmol, 3.0 eq.) in CH_2Cl_2 (7.5 mL) at rt under Ar. The resulting solution was stirred at rt for 16 h. Then, the reaction

mixture was evaporated under reduced pressure to give the crude product as a white solid. Purification by flash column chromatography on silica with 80:20 hexane-EtOAc as eluent gave *N*-Boc pyrrolidine-3-methyl ester **3c** (445 mg, 81%) as a colourless oil, R_F (80:20 hexane-Et_2O) 0.14, IR (ATR) 2976, 1736 (C=O, CO_2Me), 1691 (C=O, Boc) 1400, 1163 cm^{-1}; ^1H NMR (400 MHz, $CDCl_3$) δ 3.71 (s, 3H, OMe), 3.63-3.44 (m, 3H, CH), 3.37 -3.32 (m, 1H, CH), 3.07-3.01 (m, 1H, CH), 2.14-2.10 (m, 2H, CH), 1.45 (s, 9H, CMe_3); ^{13}C NMR (100.6 MHz, $CDCl_3$) δ 173.7 (C=O, CO_2Me), 154.4 (C=O, Boc), 79.6 (O*C*Me_3), 52.2 (OMe), 48.2 (CH_2), 45.2 (CH_2), 43.3 (CH), 42.4 (CH_2), 28.6 (C*Me*$_3$); MS (ESI) *m/z* 252 $(M + Na)^+$; HRMS *m/z* calcd for $C_{11}H_{19}NO_4$ $(M + Na)^+$ 252.1206, found 252.1208 (–0.9 ppm error). Spectroscopic data consistent with those reported in the literature.[7]

Lab Book Reference: PJ-06-82.

1,3-Di-*tert*-butyl pyrrolidine-1,3-dicarboxylate 3d

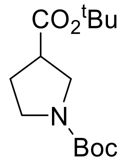

3d

tert-Butyl 2,2,2-trichloroacetimidate (1.43 mL, 8.0 mmol, 2.0 eq.) was added to a stirred solution of 1-[(*tert*-butoxy)carbonyl]pyrrolidine-3-carboxylic acid (861 mg, 4.0 mmol, 1.0 eq.) in a 1:1 mixture of anhydrous Et_2O and anhydrous CH_2Cl_2 (8 mL) at rt under N_2 and the resulting solution was stirred at rt for 4 d. The solids were removed by filtration through Celite and washed with 1:1 Et_2O and CH_2Cl_2 (30 mL) and the filtrate was concentrated under reduced pressure to give the crude product. The residue was partitioned between CH_2Cl_2 (20 mL) and $NaHCO_{3(aq)}$ (20 mL). The two layers were separated and the aqueous layer was extracted with CH_2Cl_2 (3 × 20 mL). The combined organic layers were dried ($MgSO_4$) and concentrated under reduced pressure to give the crude product. Purification by flash column chromatography on silica with 7:3 hexane-EtOAc as eluent gave pyrrolidine **3d** (842 mg, 78%) as a clear oil, R_F (7:3 hexane-EtOAc) 0.39; IR (ATR) 1728 (C=O, ester), 1694 (C=O, Boc), 1401, 1366, 1250, 1150, 1122, 771 cm^{-1}; ^1H NMR (400 MHz, $CDCl_3$) δ 3.62–3.36 (m, 3H, NCH), 3.34–3.27 (m, 1H, NCH), 2.96–2.92 (m, 1H, CH), 2.08–2.05 (m, 2H, CH), 1.45 (s, 9H, CMe_3), 1.44 (s, 9H, CMe_3); ^{13}C NMR (101 MHz, $CDCl_3$) (rotamers) δ 172.5 (C=O, ester), 154.5 (C=O, Boc), 81.1 (*C*Me_3), 79.4 (O*C*Me_3), 48.2

(NCH$_2$), 45.5 (NCH$_2$), 45.2 (NCH$_2$), 44.3 (CH), 43.6 (NCH$_2$), 28.9 (CH$_2$), 28.46 (C*Me*$_3$), 28.5 (CH$_2$), 28.1 (C*Me*$_3$); HRMS (ESI) *m/z* calcd for $C_{14}H_{25}NO_4$ $(M + Na)^+$ 272.1856, found 272.1860 (–1.4 ppm error).

Lab Book Reference: JDF_B_331

1-(*tert*-Butyl) 2-methyl piperidine-1,2-dicarboxylate 3j

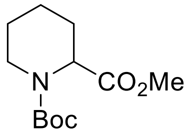

3j

A solution of Boc$_2$O (1.22 g, 5.59 mmol, 2.0 eq.) in CH_2Cl_2 (7.5 mL) was added dropwise to a stirred solution of piperidine-2-methyl ester (400 mg, 2.79 mmol, 1.0 eq.) and Et$_3$N (1.16 mL, 8.37 mmol, 3.0 eq.) in CH_2Cl_2 (7.5 mL) at rt under Ar. The resulting solution was stirred at rt for 16 h. Then, the reaction mixture was evaporated under reduced pressure to give the crude product as a white solid. Purification by flash column chromatography on silica with 90:10 hexane-EtOAc as eluent gave *N*-Boc piperidine-2-methyl ester **3k** (650 mg, 95%) as a colourless oil, R_F (90:10 hexane-EtOAc) 0.11, IR (ATR) 2938, 1742 (C=O, CO$_2$Me), 1693 (C=O, Boc) 1364, 1155 cm^{-1}; ^{1}H NMR (400 MHz, CDCl$_3$) (50:50 mixture of rotamers) δ 4.90-4.79 (m, 0.5H, NCH), 4.71-4.63 (m, 0.5H, NCH), 4.06-3.83 (m, 1H, NCH), 3.69 (s, 3H, OMe), 2.97-2.79 (m, 1H, NCH), 2.18-2.14 (m, 1H, CH), 1.66-1.60 (m, 3H, CH), 1.43-1.40 (m, 10H, CH and CMe$_3$), 1.21-1.10 (m, 1H, CH); ^{13}C NMR (100.6 MHz, CDCl$_3$) (rotamers) δ 172.7 (C=O, CO$_2$Me), 172.5 (C=O, CO$_2$Me), 156.0 (C=O, Boc), 155.6 (C=O, Boc), 80.0 (O*C*Me$_3$), 55.0 (NCH), 53.8 (NCH), 52.1 (OMe), 42.2 (NCH$_2$), 41.2 (NCH$_2$), 28.4 (C*Me$_3$*), 26.8 (CH$_2$), 24.9 (CH$_2$), 24.7 (CH$_2$), 20.9 (CH$_2$); MS (ESI) *m/z* 266 $(M + Na)^+$; HRMS *m/z* calcd for $C_{12}H_{21}NO_4$ $(M + Na)^+$ 266.1363, found 266.1361 (+0.5 ppm error). Spectroscopic data consistent with those reported in the literature.[8]

Lab Book Reference: PJ-06-98.

1-(*tert*-Butyl) 2-methyl pyrrolidine-1,2-dicarboxylate 3k

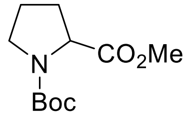

3k

Potassium carbonate (579 mg, 4.2 mmol, 3.0 eq.) was added to a stirred solution of *N*-Boc pyrrolidine-2-carboxylic acid (300 mg, 1.4 mmol, 1.0 eq.) in DMF (6 mL) at rt. The resulting suspension was stirred at rt for 30 min and methyl iodide (0.26 mL, 4.2 mmol, 3.0 eq.) was added. The mixture was then stirred at rt for 18 h. Water (3 mL) was added and the mixture was extracted with EtOAc (4 × 10 mL). The combined organics were washed with brine (4 × 5 mL), dried ($MgSO_4$) and evaporated under reduced pressure to give the crude product as an orange oil. Purification by flash chromatography on silica with 80:20 hexane-EtOAc as eluent gave *N*-Boc pyrrolidine-2-methyl ester **3k** (270 mg, 84%) as a colourless oil, R_F (80:20 hexane-EtOAc) 0.15, IR (ATR) 2975, 1746 (C=O, CO_2Me), 1695 (C=O, Boc) 1391, 1157 cm^{-1}; ^{1}H NMR (400 MHz, $CDCl_3$) 60:40 mixture of rotamers δ 4.32 (dd, J = 8.5, 3.5 Hz, 0.4H, CH), 4.22 (dd, J = 8.5, 4.0 Hz, 0.6H, CH), 3.72 (s, 3H, OMe), 3.59-3.36 (m, 2H, CH), 2.25-2.15 (m, 1H, CH), 1.99-1.90 (m, 2H, CH), 1.88-1.60 (m, 1H, CH), 1.46 (s, 3.6H, CMe_3), 1.41 (s, 5.4H, CMe_3); ^{13}C NMR (100.6 MHz, $CDCl_3$) (rotamers) δ 173.9 (C=O, CO_2Me), 153.9 (C=O, Boc), 80.0 (*C*Me_3), 79.9 (O*C*Me_3), 59.2 (OMe), 58.8 (OMe), 52.2 ($CHCO_2$), 52.1 ($CHCO_2$), 46.6 (CH_2), 46.4 (CH_2), 30.9 (CH_2), 30.0 (CH_2), 28.5 (C*Me*$_3$), 28.4 (C*Me*$_3$), 24.4 (CH_2), 23.8 (CH_2); MS (ESI) *m/z* 252 (M + Na)$^+$; HRMS *m/z* calcd for $C_{11}H_{19}NO_4$ (M + Na)$^+$ 252.1206, found 252.1207 (–0.7 ppm error). Spectroscopic data consistent with those reported in the literature.[9]

Lab Book Reference: PJ-06-79.

1-(*tert*-Butyl) 4-methyl 4-(4-methylphenyl)piperidine-1,4-dicarboxylate 4a

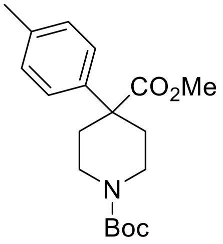

4a

Using general procedure D, *N*-Boc piperidine-4-methyl ester **3a** (150 mg, 0.62 mmol, 1.2 eq.), LiHMDS (1.02 mL of a 1.0 M solution in toluene, 1.02 mmol, 2.0 eq.), [(cinnamyl)$PdCl_2$] (5.2 mg, 0.010 mmol,

0.02 eq.), *t*-$Bu_3P\cdot HBF_4$ (5.8 mg, 0.020 mmol, 0.04 eq.) and 4-bromotoluene (87 mg, 0.51 mmol, 1.0 eq.) in toluene (6 mL) at rt for 16 h gave the crude product as an orange oil. Purification by flash column chromatography on silica with 85:15 hexane-EtOAc as eluent gave *N*-Boc aryl piperidine-4-methyl ester **4a** (120 mg, 70%) as a colourless oil , R_F (80:20 hexane-EtOAc) 0.27, IR (ATR) 2973, 1727 (C=O, CO_2Me), 1690 (C=O, Boc), 1421, 1161 cm^{-1}; ^1H NMR (400 MHz, $CDCl_3$) δ 7.24 (d, J = 8.0 Hz, 2H, Ar), 7.15 (d, J = 8.0 Hz, 2H, Ar), 4.04-3.85 (m, 2H, NCH), 3.65 (s, 3H, OMe), 3.11-2.91 (m, 2H, NCH), 2.50-2.47 (m, 2H, CH), 2.32 (s, 3H, C_6H_4*Me*), 1.93-1.76 (m, 2H, CH), 1.45 (s, 9H, CMe_3); ^{13}C NMR (100.6 MHz, $CDCl_3$) (rotamers) δ 174.9 (C=O, CO_2Me), 155.0 (C=O, Boc), 139.3 (*ipso*-Ar), 137.1 (*ipso*-Ar), 129.5 (Ar), 125.8 (Ar), 79.7 (O*C*Me_3), 52.5 (OMe), 49.2 (C), 42.0 (NCH_2), 41.3 (NCH_2), 34.0 (CH_2), 33.7 (CH_2), 28.5 (C*Me_3*), 21.1 (C_6H_4*Me*); MS (ESI) *m/z* 356 $(M + Na)^+$; HRMS *m/z* calcd for $C_{19}H_{27}NO_4$ $(M + Na)^+$ 356.1832, found 356.1832 (+0.1 ppm error).

Lab Book Reference: PJ-06-89.

1-*tert*-Butyl 3-methyl 3-(4-methylphenyl)piperidine-1,3-dicarboxylate 4b

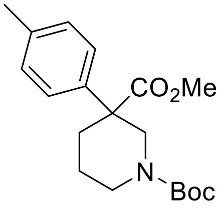

4b

Using general procedure D, *N*-Boc piperidine-3-methyl ester **3b** (191 mg, 0.76 mmol, 1.2 eq.), LiHMDS (1.32 mL of a 1.0 M solution in toluene, 1.32 mmol, 2.0 eq.), [(cinnamyl)$PdCl_2$] (6.8 mg, 0.013 mmol, 0.02 eq.), *t*-$Bu_3P\cdot HBF_4$ (7.5 mg, 0.026 mmol, 0.04 eq.) and 4-bromotoluene (113 mg, 0.66 mmol, 1.0 eq.) in toluene (6 mL) at rt for 16 h gave the crude product as a yellow oil. Purification by flash column chromatography on silica with 95:5-90:10 hexane-EtOAc as eluent gave *N*-Boc aryl piperidine-3-methyl ester **4b** (174 mg, 79%) as a yellow oil, R_F (90:10 hexane-EtOAc) 0.13, IR (ATR) 2950, 1730 (C=O, CO_2Me), 1688 (C=O, Boc), 1424, 1147, 731 cm^{-1}; ^1H NMR (400 MHz, $CDCl_3$) δ 7.30-7.27 (m, 2H, Ar), 7.14 (d, J = 8.0 Hz, 2H, Ar), 4.38 (d, J = 13.5 Hz, 1H, NCH), 3.71-3.68 (m, 1H, NCH), 3.63 (s, 3H, OMe), 3.47 (d, J = 13.5 Hz, 1H, NCH), 3.04 (ddd, J = 13.0, 6.5, 6.5 Hz, 1H, NCH), 2.49-2.43 (m, 1H, CH), 2.32 (s, 3H, C_6H_4*Me*), 1.97-1.92 (m, 1H, CH), 1.69-1.59 (m, 2H, CH), 1.481-1.41 (m, 9H, CMe_3); ^{13}C NMR (100.6 MHz, $CDCl_3$) δ 174.2 (C=O, CO_2Me), 154.7 (C=O, Boc), 137.3 (*ipso*-Ar), 137.2 (*ipso*-Ar), 129.5 (Ar), 126.2 (Ar), 79.7 (O*C*Me_3), 52.3 (OMe), 50.7 (C), 50.1 (NCH_2), 43.2 (NCH_2), 33.1 (CH_2),

28.5 (C*Me₃*), 22.5 (CH_2), 21.1 (C_6H_4*Me*); MS (ESI) *m/z* 356 $(M + Na)^+$; HRMS *m/z* calcd for $C_{19}H_{27}NO_4$ $(M + Na)^+$ 356.1838, found 356.1837 (–0.3 ppm error).
Lab Book Reference: PJ-06-92.

1-*tert*-Butyl 3-methyl 3-(4-methylphenyl)pyrrolidine-1,3-dicarboxylate 4c

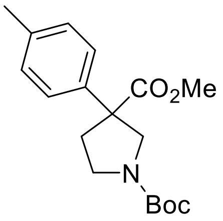

4c

Using general procedure D, *N*-Boc pyrolidine-3-methyl ester **3c** (180 mg, 0.76 mmol, 1.2 eq.), LiHMDS (1.32 mL of a 1.0 M solution in toluene, 1.32 mmol, 2.0 eq.), [(cinnamyl)$PdCl_2$] (6.8 mg, 0.013 mmol, 0.02 eq.), *t*-$Bu_3P\cdot HBF_4$ (7.5 mg, 0.026 mmol, 0.04 eq.) and 4-bromotoluene (113 mg, 0.66 mmol, 1.0 eq.) in toluene (6 mL) at rt for 16 h gave the crude product as an orange oil. Purification by flash column chromatography on silica with 90:10 hexane-EtOAc as eluent gave *N*-Boc aryl pyrolidine-3-methyl ester **4c** (78 mg, 37%) as a white solid, mp 102-104 °C; R_F (80:20 hexane-EtOAc) 0.20, IR (ATR) 2971, 1721 (C=O, CO_2Me), 1686 (C=O, Boc), 1406, 1170, 1151, 1123, 1097 cm^{-1}; ^1H NMR (400 MHz, $CDCl_3$) (50:50 mixture of rotamers) δ 7.20 (d, J = 8.0 Hz, 2H, Ar), 7.14 (d, J = 8.0 Hz, 2H, Ar), 4.36 (d, J = 11.0 Hz, 0.5H, NCH), 4.32 (d, J = 11.0 Hz, 0.5H, NCH), 3.65-3.61 (m, 3.5H, OMe and NCH), 3.53-3.47 (m, 1H, NCH), 3.42-3.31 (m, 1.5H, NCH), 2.90-2.81 (m, 1H, CH), 2.33 (s, 3H, C_6H_4Me), 2.20-2.10 (m, 1H, CH), 1.48 (s, 4.5H, CMe_3), 1.46 (s, 4.5H, CMe_3); ^{13}C NMR (100.6 MHz, $CDCl_3$) (rotamers) δ 174.3 (C=O, CO_2Me), 154.5 (C=O, Boc), 137.5 (*ipso*-Ar), 137.4 (*ipso*-Ar), 136.8 (*ipso*-Ar), 136.7 (*ipso*-Ar), 129.5 (Ar), 126.5 (Ar), 126.4 (Ar), 79.7 (O*C*Me₃), 79.6 (O*C*Me₃), 57.1 (C), 56.2 (NCH_2), 53.9 (OMe), 53.3 (OMe), 52.9 (NCH_2), 44.9 (NCH_2), 44.5 (NCH_2), 34.9 (CH_2), 33.9 (CH_2), 28.6 (C*Me₃*), 21.1 (C_6H_4*Me*); MS (ESI) *m/z* 342 $(M + Na)^+$; HRMS *m/z* calcd for $C_{18}H_{25}NO_4$ $(M + Na)^+$ 342.1676, found 342.1167 (–0.1 ppm error).

Lab Book Reference: PJ-06-85.

1,3-Di-*tert*-butyl 3-(4-methylphenyl)pyrrolidine-1,3-dicarboxylate 4d

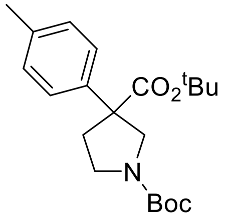

4d

Using general procedure D, *N*-Boc pyrolidine-3-*t*-butyl ester **3d** (205 mg, 0.76 mmol, 1.2 eq.), LiHMDS (1.32 mL of a 1.0 M solution in toluene, 1.32 mmol, 2.0 eq.), [(cinnamyl)PdCl_2] (6.8 mg, 0.013 mmol, 0.02 eq.), *t*-$Bu_3P \cdot HBF_4$ (7.5 mg, 0.026 mmol, 0.04 eq.) and 4-bromotoluene (113 mg, 0.66 mmol, 1.0 eq.) in toluene (6 mL) at rt for 16 h gave the crude product as an orange oil. Purification by flash column chromatography on silica with 90:10 hexane-EtOAc as eluent gave *N*-Boc aryl pyrolidine-3-*t*-butyl ester **4d** (170 mg, 71%) as a white solid, mp 84-86 °C; R_F (70:30 hexane-EtOAc) 0.38, IR (ATR) 2974, 1714 (C=O, CO_2*t*Bu), 1687 (C=O, Boc), 1410, 1147 cm^{-1}; ^1H NMR (400 MHz, $CDCl_3$) (50:50 mixture of rotamers) δ 7.19 (d, J = 8.0 Hz, 2H, Ar), 7.13 (d, J = 8.0 Hz, 2H, Ar), 4.32 (d, J = 11.0 Hz, 1H, NCH), 3.54 (d, J = 11.0 Hz, 0.5H, NCH), 3.52-3.40 (m, 0.5H, NCH), 3.39-3.34 (m, 2H, NCH), 2.82-2.76 (m, 1H, CH), 2.33 (s, 3H, C_6H_4*Me*), 2.14-2.06 (m, 1H, CH), 1.48 (s, 4.5H, C*Me*$_3$), 1.46 (s, 4.5H, CMe_3), 1.34 (s, 4.5H, CMe_3), 1.33 (s, 4.5H, CMe_3); ^{13}C NMR (100.6 MHz, $CDCl_3$) (rotamers) δ 178.2 (C=O, CO_2tBu), 172.8 (C=O, Boc), 154.6 (*ipso*-Ar), 137.1 (*ipso*-Ar), 129.3 (Ar), 126.4 (Ar), 81.5 (O*C*Me_3), 79.6 (O*C*Me_3), 57.9 (C), 57.0 (C), 53.9 (NCH_2), 53.3 (NCH_2), 44.9 (NCH_2), 44.5 (NCH_2), 34.9 (CH_2), 34.0 (CH_2), 28.7 (C*Me*$_3$), 27.9 (C*Me*$_3$), 21.2 (C_6H_4*Me*); MS (ESI) *m/z* 284 $(M + Na)^+$; HRMS *m/z* calcd for $C_{21}H_{31}NO_4$ $(M + Na)^+$ 384.2145, found 384.2138 (+0.9 ppm error).
Lab Book Reference: PJ-07-45.

Methyl 1-(4-methylphenyl)cyclopentane-1-carboxylate 4e

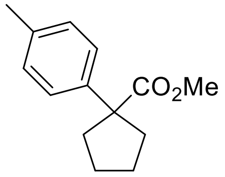

4e

Using general procedure E, *n*-BuLi (1.0 mL of a 2.3 M solution in hexane, 2.3 mmol, 2.0 eq.), diisopropylamine (0.32 mL, 2.3 mmol, 2.0 eq.), methyl cyclopentanecarboxylate (0.2 mL, 1.56 mmol, 1.2 eq.), [(cinnamyl)PdCl_2] (13 mg, 0.026 mmol, 0.02 eq.), *t*-$Bu_3P \cdot HBF_4$ (15 mg, 0.052 mmol, 0.04 eq.)

and 4-bromotoluene (222 mg, 1.30 mmol, 1.0 eq.) in dry toluene (7 mL) at rt for 16 h gave the crude product as an orange oil. Purification by flash column chromatography on silica with 98:2-90:10 hexane-Et_2O as eluent gave aryl cyclopentane ester **4e** (58 mg, 58%) as a white solid, mp 46-48 °C; R_F (90:10 hexane-Et_2O) 0.37; IR (ATR) 2947, 1720 (C=O), 1193, 1157, 742 cm^{-1}; ^1H NMR (400 MHz, $CDCl_3$) δ 7.26 (d, J = 8.0 Hz, 2H, Ar), 7.12 (d, J = 8.0 Hz, 2H, Ar), 3.61 (s, 3H, OMe), 2.66-2.60 (m, 2H, CH_2), 2.33 (s, 3H, C_6H_4*Me*), 1.94-1.87 (m, 2H, CH_2), 1.75-1.69 (m, 4H, CH_2); ^{13}C NMR (100.6 MHz, $CDCl_3$) δ 176.8 (C=O), 140.5 (*ipso*-Ar), 136.4 (*ipso*-Ar), 129.1 (Ar), 126.8 (Ar), 58.8 (OMe), 52.4 (C), 36.3 (CH_2), 23.7 (CH_2), 21.1 (C_6H_4*Me*); MS (ESI) *m/z* 241 $(M + Na)^+$; HRMS *m/z* calcd for $C_{14}H_{18}O_2$ $(M + Na)^+$ 241.1199, found 241.1191 (+0.8 ppm error). Spectroscopic data consistent with those reported in the literature.[10]

Lab Book Reference: PJ-06-44.

Methyl 4-(4-methylphenyl)oxane-4-carboxylate 4f

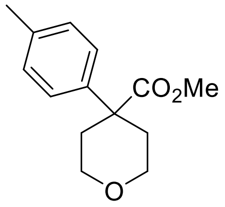

4f

sing general procedure D, methyl oxane-4-carboxylate (0.1 mL, 0.76 mmol, 1.2 eq.), LiHMDS (1.32 mL of a 1.0 M solution in toluene, 1.32 mmol, 2.0 eq.), [(cinnamyl)$PdCl_2$] (6.8 mg, 0.013 mmol, 0.02 eq.), *t*-$Bu_3P\cdot HBF_4$ (7.5 mg, 0.026 mmol, 0.04 eq.) and 4-bromotoluene (113 mg, 0.66 mmol, 1.0 eq.) in toluene (6 mL) at rt for 24 h gave the crude product as a yellow oil. Purification by flash column chromatography on silica with 75:25 hexane-EtOAc as eluent gave aryl THP-4-methyl ester **4f** (75 mg, 48%) as a yellow solid, mp 58-60 °C; R_F (75:25 hexane-EtOAc) 0.31; IR (ATR) 2949, 1719 (C=O), 1445, 1125, 739 cm^{-1}; ^1H NMR (400 MHz, $CDCl_3$) δ 7.26 (d, J = 8.0 Hz, 2H, Ar), 7.16 (d, J = 8.0 Hz, 2H, Ar), 3.92 (ddd, J = 11.5, 4.0, 4.0 Hz, 2H, OCH), 3.66 (s, 3H, OMe), 3.55 (ddd, J = 11.5, 11.0, 2.0 Hz, 2H, OCH), 2.53-2.48 (m, 2H, CH), 2.33 (s, 3H, Ar Me), 1.97 (ddd, J = 13.5, 11.0, 4.0 Hz, 2H, CH); ^{13}C NMR (100.6 MHz, $CDCl_3$) δ 175.1 (C=O), 139.7 (*ipso*-Ar), 137.1 (*ipso*-Ar), 129.5 (Ar), 125.7 (Ar), 65.8 (OCH_2), 52.5 (OMe), 48.5 (C), 34.6 (CH_2), 21.1 (C_6H_4*Me*); MS (ESI) *m/z* 257 $(M + Na)^+$; HRMS *m/z* calcd for $C_{14}H_{18}O_3$ $(M + Na)^+$ 257.1148, found 257.1146 (+1.3 ppm error).

Lab Book Reference: PJ-06-94.

Methyl 1-(4-methoxyphenyl)cyclopentane-1-carboxylate 4l

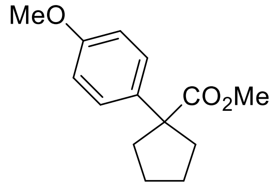

4l

Using general procedure E, *n*-BuLi (0.57 mL of a 2.3 M solution in hexane, 1.32 mmol, 2.0 eq.), diisopropylamine (0.19 mL, 1.32 mmol, 2.0 eq.), methyl cyclopentanecarboxylate (0.1 mL, 0.79 mmol, 1.2 eq.), [(cinnamyl)PdCl_2] (6.8 mg, 0.013 mmol, 0.02 eq.), *t*-Bu_3P·HBF_4 (7.5 mg, 0.026 mmol, 0.04 eq.) and 4-bromoanisole (0.08 mL, 0.66 mmol, 1.0 eq.) in dry toluene (6 mL) at rt for 16 h gave the crude product as a black oil. Purification by flash column chromatography on silica with 98:2 hexane-Et_2O as eluent gave aryl cyclopentane ester **4l** (106 mg, 68%) as a yellow oil, R_F (95:5 hexane-EtOAc) 0.08, IR (ATR) 2950, 1725 (C=O), 1511, 1247, 1181, 1034, 830 cm^{-1}; ^{1}H NMR (400 MHz, $CDCl_3$) δ 7.29 (d, J = 9.0 Hz, 2H, Ar), 6.84 (d, J = 9.0 Hz, 2H, Ar), 3.79 (s, 3H, OMe), 3.60 (s, 3H, OMe), 2.65-2.59 (m, 2H, CH), 1.90-1.84 (m, 2H, CH), 1.73-1.68 (m, 4H, CH); ^{13}C NMR (100.6 MHz, $CDCl_3$) δ 176.8 (C=O), 158.4 (*ipso*-Ar), 135.5 (*ipso*-Ar), 128.0 (Ar), 113.7 (Ar), 58.4 (C), 55.3 (OMe), 52.4 (OMe), 36.4 (CH_2), 23.6 (CH_2); MS (ESI) *m/z* 257 (M + Na)$^+$; HRMS *m/z* calcd for $C_{14}H_{18}O_3$ (M + Na)$^+$ 257.1148 found 257.1146 (+0.8 ppm error).
Lab Book Reference: PJ-06-68.

Methyl 1-(4-(trifluoromethyl)phenyl)cyclopentane-1-carboxylate 4m

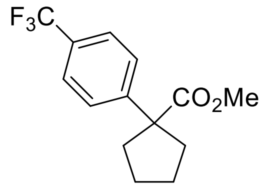

4m

Using general procedure E, *n*-BuLi (0.57 mL of a 2.3 M solution in hexane, 1.32 mmol, 2.6 eq.), diisopropylamine (0.19 mL, 1.32 mmol, 2.6 eq.), methyl cyclopentanecarboxylate (0.1 mL, 0.79 mmol, 1.6 eq.), [(cinnamyl)PdCl_2] (6.8 mg, 0.013 mmol, 0.026 eq.), *t*-Bu_3P·HBF_4 (7.5 mg, 0.026 mmol, 0.052 eq.) and 4-bromobenzotrifluoride (0.07 mL, 0.66 mmol, 1.0 eq.) in dry toluene (6 mL) at rt for 16 h gave the crude product as a black oil. Purification by flash column chromatography on silica with 98:2 hexane-Et_2O as eluent gave aryl cyclopentane ester **4m** (97 mg, 72%) as a white solid, mp 52-54 °C; R_F (90:10

hexane-Et_2O) 0.3, IR (ATR) 2967, 1730 (C=O), 1320, 1158, 1122, 1101, 1067 cm^{-1}; ^{1}H NMR (400 MHz, $CDCl_3$) δ 7.56 (d, J = 8.0 Hz, 2H, Ar), 7.47 (d, J = 8.0 Hz, 2H, Ar), 3.62 (s, 3H, OMe), 2.70-2.64 (m, 2H, CH), 1.95-1.88 (m, 2H, CH), 1.76-1.73 (m, 4H, CH); ^{13}C NMR (100.6 MHz, $CDCl_3$) δ 175.9 (C=O), 147.4 (*ipso*-Ar), 129.2 (q, J = 32.5 Hz, *ipso*-Ar), 127.4 (Ar), 125.5 (q, J = 4.0 Hz, Ar), 122.5 (q, J = 272.0 Hz, CF_3), 59.2 (C), 52.7 (OMe), 36.3 (CH_2), 23.7 (CH_2); MS (ESI) *m/z* 273 $(M + H)^+$; HRMS *m/z* calcd for $C_{14}H_{16}F_3O_2$ $(M + H)^+$ 273.1097 found 273.1098 (–2.2 ppm error).
Lab Book Reference: PJ-06-62.

Methyl 1-(4-fluorophenyl)cyclopentane-1-carboxylate 4n

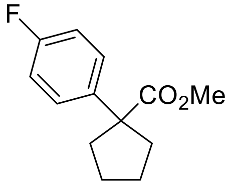

4n

Using general procedure E, *n*-BuLi (0.57 mL of a 2.3 M solution in hexane, 1.32 mmol, 2.0 eq.), diisopropylamine (0.19 mL, 1.32 mmol, 2.0 eq.), methyl cyclopentanecarboxylate (0.1 mL, 0.79 mmol, 1.2 eq.), [(cinnamyl)$PdCl_2$] (6.8 mg, 0.013 mmol, 0.02 eq.), *t*-$Bu_3P\cdot HBF_4$ (7.5 mg, 0.026 mmol, 0.04 eq.) and 4-bromofluorobenzene (0.07 mL, 0.66 mmol, 1.0 eq.) in dry toluene (6 mL) at rt for 16 h gave the crude product as a black oil. Purification by flash column chromatography on silica with 80:20 CH_2Cl_2-hexane as eluent gave aryl cyclopentane ester **4n** (50 mg, 34%) as a colourless oil, R_F (CH_2Cl_2) 0.19, IR (ATR) 2952, 1726 (C=O), 1508, 1228, 1154, 834 cm^{-1}; ^{1}H NMR (400 MHz, $CDCl_3$) δ 7.33 (dd, J = 9.0, 5.5 Hz, 2H, Ar), 7.01-6.96 (dd, J = 9.0, 9.0 Hz, 2H, Ar), 3.61 (s, 3H, OMe), 2.67-2.61 (m, 2H, CH), 1.91-1.83 (m, 2H, CH), 1.76-1.69 (m, 4H, CH); ^{13}C NMR (100.6 MHz, $CDCl_3$) δ 176.4 (C=O), 161.8 (d, J = 245.0 Hz, *ipso*-Ar), 139.2 (d, J = 3.0 Hz, *ipso*-Ar), 128.6 (d, J = 8.0 Hz, Ar), 115.1 (d, J = 21.0 Hz, Ar), 58.6 (C), 52.5 (OMe), 36.4 (CH_2), 23.6 (CH_2); MS (ESI) *m/z* 223 $(M + H)^+$; HRMS *m/z* calcd for $C_{13}H_{15}FO_2$ $(M + H)^+$ 223.1129 found 223.1125 (+1.9 ppm error). Spectroscopic data consistent with those reported in the literature.[10]
Lab Book Reference: PJ-06-67.

Methyl 1-(2-methylphenyl)cyclopentane-1-carboxylate 4o

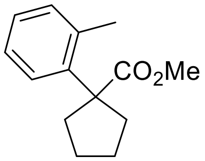

4o

Using general procedure E, *n*-BuLi (0.57 mL of a 2.3 M solution in hexane, 1.32 mmol, 2.0 eq.), diisopropylamine (0.19 mL, 1.32 mmol, 2.0 eq.), methyl cyclopentanecarboxylate (0.1 mL, 0.79 mmol, 1.2 eq.), [(cinnamyl)PdCl_2] (6.8 mg, 0.013 mmol, 0.02 eq.), *t*-$Bu_3P{\cdot}HBF_4$ (7.5 mg, 0.026 mmol, 0.04 eq.) and 2-bromotoluene (0.08 mL, 0.66 mmol, 1.0 eq.) in dry toluene (6 mL) at rt for 16 h gave the crude product as a black oil. Purification by flash column chromatography on silica with 98:2 hexane-Et_2O as eluent gave aryl cyclopentane ester **4o** (52 mg, 36%) as a yellow oil, R_F (90:10 hexane-Et_2O) 0.27, IR (ATR) 2949, 1725 (C=O), 1231, 1152, 741 cm^{-1}; ^{1}H NMR (400 MHz, $CDCl_3$) δ 7.35-7.34 (m, 1H, Ar), 7.19-7.13 (m, 3H, Ar), 3.62 (s, 3H, OMe), 2.56-2.49 (m, 2H, CH), 2.25 (s, 3H, C_6H_4*Me*), 2.08-2.01 (m, 2H, CH), 1.81-1.70 (m, 4H, CH); ^{13}C NMR (100.6 MHz, $CDCl_3$) δ 177.8 (C=O), 142.2 (*ipso*-Ar), 136.7 (*ipso*-Ar), 131.8 (Ar), 126.8 (Ar), 125.9 (Ar), 125.8 (Ar), 58.1 (C), 52.5 (OMe), 36.9 (CH_2), 24.7 (CH_2), 20.9 (C_6H_4*Me*); MS (ESI) *m/z* 241 $(M + Na)^+$; HRMS *m/z* calcd for $C_{14}H_{18}O_2$ $(M + Na)^+$ 241.1199 found 241.1198 (+0.3 ppm error).

Lab Book Reference: PJ-06-63.

Methyl 1-(pyridin-2-yl)cyclopentane-1-carboxylate 4p

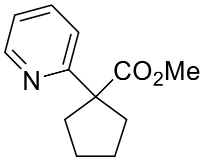

4p

Using general procedure D, methyl cyclopentanecarboxylate (0.2 mL, 1.56 mmol, 1.2 eq.), LiHMDS (2.6 mL of a 1.0 M solution in toluene, 2.6 mmol, 2.0 eq.), [(cinnamyl)PdCl_2] (13 mg, 0.026 mmol, 0.02 eq.), *t*-$Bu_3P{\cdot}HBF_4$ (15 mg, 0.052 mmol, 0.04 eq.) and 2-bromopyridine (0.12 mL, 1.30 mmol, 1.0 eq.) in toluene (7 mL) at rt for 16 h gave the crude product as an orange oil. Purification by flash column chromatography on silica with 90:10-80:20 hexane-Et_2O as eluent gave aryl cyclopentane ester **4p** (193 mg, 72%) as a yellow oil, R_F (80:20 hexane-Et_2O) 0.12, IR (ATR) 2950, 1727 (C=O), 1429, 1153, 750 cm^{-1}; ^{1}H NMR (400 MHz, $CDCl_3$) δ 8.55 (ddd, J = 5.0, 2.0, 1.0 Hz, 1H, 6-py), 7.64 (ddd, J

= 8.0, 8.0, 2.0 Hz, 1H, 4-py), 7.29 (ddd, *J* = 8.0, 1.0, 1.0 Hz, 1H, 3-py), 7.14 (ddd, *J* = 8.0, 5.0, 1.0 Hz, 1H, 5-py), 3.66 (s, 3H, OMe), 2.56-2.50 (m, 2H, CH), 2.22-2.17 (m, 2H, CH), 1.77-1.71 (m, 4H, CH); ^{13}C NMR (100.6 MHz, $CDCl_3$) δ 176.3 (C=O), 162.6 (*ipso*-Ar), 149.0 (Ar), 136.5 (Ar), 121.7 (Ar), 121.1 (Ar), 61.7 (C), 52.4 (OMe), 36.2 (CH_2), 24.6 (CH_2); MS (ESI) *m/z* 206 $(M + H)^+$; HRMS *m/z* calcd for $C_{12}H_{15}NO$ $(M + Na)^+$ 206.1170, found 206.0964 (+3.7 ppm error).
Lab Book Reference: PJ-06-56.

Using general procedure D, cyclopentanecarboxylate (1.18 mL, 9.36 mmol, 1.2 eq), LiHMDS (15.6 mL of a 1.0 M solution in toluene, 15.6 mmol, 2.0 eq), [(cinnamyl)$PdCl_2$] (80 mg, 0.156 mmol, 0.02 eq), *t*-$Bu_3P·HBF_4$ (90 mg, 0.312 mmol, 0.04 eq) and 2-bromopyridine (0.74 mL, 7.8 mmol, 1.0 eq) in toluene (36 mL) at rt for 16 h gave the crude product as an orange oil. Purification by flash column chromatography on silica with 90:10-80:20 hexane-Et_2O as eluent gave aryl cyclopentane ester **4p** (1.13 g, 71%) as a yellow oil.
Lab Book - PJ-07-15.

1-(*tert*-Butyl) 3-methyl 3-(4-fluorophenyl)piperidine-1,3-dicarboxylate 4q

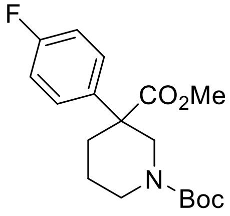

4q

Using general procedure D, *N*-Boc piperidine-3-methyl ester **3b** (192 mg, 0.76 mmol, 1.2 eq.), LiHMDS (1.32 mL of a 1.0 M solution in toluene, 1.32 mmol, 2.0 eq.), [(cinnamyl)$PdCl_2$] (6.8 mg, 0.013 mmol, 0.02 eq.), *t*-$Bu_3P·HBF_4$ (7.5 mg, 0.026 mmol, 0.04 eq.) and 4-fluorobromobenzene (0.07 mL, 0.66 mmol, 1.0 eq.) in toluene (6 mL) at rt for 16 h gave the crude product as a yellow oil. Purification by flash column chromatography on silica with 90:10-80:20 hexane-EtOAc as eluent gave *N*-Boc aryl piperidine-3-methyl ester **4q** (132 mg, 59%) as an orange oil, R_F (70:30 hexane-EtOAc) 0.24; IR (ATR) 2951, 1731 (C=O, CO_2Me), 1688 (C=O, Boc), 1511, 1423, 1147 cm^{-1}; ^{1}H NMR (400 MHz, $CDCl_3$) δ 7.40-7.37 (s, 2H, Ar), 7.02 (dd, *J* = 9.0, 9.0 Hz, 2H, Ar), 4.30-4.27 (m, 1H, NCH), 3.62 (s, 3H, OMe), 3.67-3.54 (m, 2H, NCH), 3.30-2.94 (s, 1H, NCH), 2.45-2.39 (m, 1H, CH), 1.97 (ddd, *J* = 13.5, 7.0, 7.0 Hz, 1H, CH), 1.67-1.60 (m, 2H, CH), 1.47-1.37 (m, 9H, CMe_3); ^{13}C NMR (100.6 MHz, $CDCl_3$) δ 174.0 (C=O, CO_2Me), 162.3 (d, *J* = 246.5 Hz, *ipso*-Ar), 154.7 (C=O, Boc), 135.8 (d, *J* = 3.5 Hz, *ipso*-Ar), 128.3 (d, *J*

= 8.0 Hz, Ar), 115.6 (d, J = 21.0 Hz, Ar), 79.9 (O*C*Me$_3$), 52.4 (OMe), 50.6 (C), 49.8 (NCH$_2$), 43.2 (NCH$_2$), 33.1 (CH$_2$), 28.5 (C*Me$_3$*), 22.3 (CH$_2$); MS (ESI) *m/z* 360 (M + Na)$^+$; HRMS *m/z* calcd for $C_{18}H_{24}NO_4$ (M + Na)$^+$ 360.1582, found 360.1589 (–2.5 ppm error).
Lab Book Reference: PJ-07-11.

Methyl 4-(4-methoxyphenyl)oxane-4-carboxylate 4r

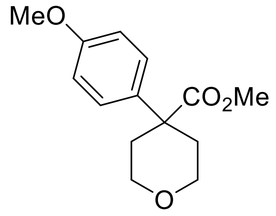

4r

A solution of methyl oxane-4-carboxylate (0.1 mL, 0.76 mmol, 1.2 eq.), in toluene (1 mL) was added dropwise to a stirred solution of LiHMDS (1.32 mL of a 1.0 M solution in toluene, 1.32 mmol, 2.0 eq.) in 95:5 toluene-THF (2.1 mL) at rt under Ar. The resulting solution was vigorously stirred at rt for 40 min. In a separate reaction flask, [(cinnamyl)PdCl$_2$] (6.8 mg, 0.013 mmol, 0.02 eq.), *t*-Bu$_3$P·HBF$_4$ (7.5 mg, 0.026 mmol, 0.04 eq.) and 4-bromoanisole (0.083 mL, 0.66 mmol, 1.0 eq.) were added, followed by dry toluene (5 mL). The reaction flask was then evacuated under reduced pressure and back-filled with Ar three times. The LiHMDS/ester solution was then added dropwise to the reaction flask and the resulting solution was stirred and heated at 50 °C for 16 h. The solution was poured into 1 M $AcOH_{(aq)}$ (10 mL) and the aqueous layer was extracted with EtOAc (3 × 10 mL). The combined organics were washed with brine (20 mL) and sat $NaHCO_{3(aq)}$ (20 mL), dried ($MgSO_4$) and evaporated under reduced pressure to give the crude product as an orange oil. Purification by flash column chromatography on silica with 70:30 hexane-EtOAc as eluent gave aryl THP-4-methyl ester **4r** (104 mg, 63%) as a yellow solid, mp 56-58 °C; R_F (70:30 hexane-EtOAc) 0.25; IR (ATR) 2928, 1720 (C=O), 1512, 1100, 1027, 827 cm^{-1}; ^{1}H NMR (400 MHz, CDCl$_3$) δ 7.29 (d, J = 9.0 Hz, 2H, Ar), 6.87 (d, J = 9.0 Hz, 2H, Ar), 3.92 (ddd, J = 12.0, 4.0, 4.0 Hz, 2H, OCH), 3.79 (s, 3H, OMe), 3.66 (s, 3H, OMe), 3.53 (ddd, J = 12.0, 12.0, 2.0 Hz, 2H, OCH), 2.53-2.48 (m, 2H, CH), 1.94 (ddd, J = 13.0, 12.0, 2.0 Hz, 2H, CH); ^{13}C NMR (100.6 MHz, CDCl$_3$) δ 175.1 (C=O), 158.7 (*ipso*-Ar), 134.6 (*ipso*-Ar), 126.9 (Ar), 114.1 (Ar), 65.7 (OCH$_2$), 55.3 (OMe), 52.4 (OMe), 34.6 (CH$_2$); MS (ESI) *m/z* 273 (M + Na)$^+$; HRMS *m/z* calcd for $C_{14}H_{18}O_4$ (M + Na)$^+$ 273.1097, found 273.1094 (+3.5 ppm error).
Lab Book Reference: PJ-07-26.

Methyl 4-(pyridin-2-yl)oxane-4-carboxylate 4s

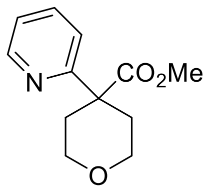

4s

A solution of methyl oxane-4-carboxylate (0.1 mL, 0.76 mmol, 1.2 eq.), in toluene (1 mL) was added dropwise to a stirred solution of LiHMDS (1.32 mL of a 1.0 M solution in toluene, 1.32 mmol, 2.0 eq.) in 95:5 toluene-THF (2.1 mL) at rt under Ar. The resulting solution was vigorously stirred at rt for 40 min. Io a separate reaction flask, [(cinnamyl)$PdCl_2$] (6.8 mg, 0.013 mmol, 0.02 eq.), *t*-$Bu_3P\cdot HBF_4$ (7.5 mg, 0.026 mmol, 0.04 eq.) and 2-bromopyridine (0.063 mL, 0.66 mmol, 1.0 eq.) were added, followed by dry toluene (5 mL). The reaction flask was then evacuated under reduced pressure and back-filled with Ar three times. The LiHMDS/ester solution was then added dropwise to the reaction flask and the resulting solution was stirred and heated at 50 °C for 16 h. The solution was poured into 1 M $AcOH_{(aq)}$ (10 mL) and the aqueous layer was extracted with EtOAc (3 × 10 mL). The combined organics were washed with brine (20 mL) and sat $NaHCO_{3(aq)}$ (20 mL), dried ($MgSO_4$) and evaporated under reduced pressure to give the crude product as an orange oil. Purification by flash column chromatography on silica with 70:30 hexane-EtOAc as eluent gave aryl THP-4-methyl ester **4s** (77 mg, 53%) as a yellow oil, R_F (80:20 hexane-EtOAc) 0.07; IR (ATR) 2954, 1726 (C=O), 1430, 1127, 1104 cm^{-1}; ^{1}H NMR (400 MHz, $CDCl_3$) δ 8.59-8.57 (m, 1H, 6-py), 7.67 (ddd, J = 8.0, 8.0, 2.0 Hz, 1H, 4-py), 7.34-7.32 (m, 1H, 3-py), 7.18 (ddd, J = 8.0, 5.0, 1.0 Hz, 1H, 5-py), 3.87 (ddd, J = 12.0, 4.0, 4.0 Hz, 2H, OCH), 3.70 (s, 3H, OMe), 3.62 (ddd, J = 12.0, 10.0, 2.5 Hz, 2H, OCH), 2.47 (ddd, J = 14.0, 4.0, 2.5 Hz, 2H, CH), 2.16 (ddd, J = 14.0, 10.0, 4.0 Hz, 2H, CH); ^{13}C NMR (100.6 MHz, $CDCl_3$) δ 174.5 (C=O), 161.4 (*ipso*-Ar), 149.4 (Ar), 137.0 (Ar), 122.3 (Ar), 120.7 (Ar), 65.6 (OCH_2), 52.6 (OMe), 33.8 (CH_2); MS (ESI) *m/z* 244 (M + Na)$^+$; HRMS *m/z* calcd for $C_{12}H_{15}NO_3$ (M + Na)$^+$ 244.0944, found 244.0940 (+0.2 ppm error).

Lab Book Reference: PJ-07-30.

Methyl 2-[(4-fluorophenyl)methyl]oxolane-2-carboxylate 5a

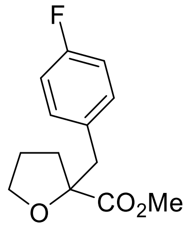

5a

Using general procedure F, LiHMDS (1.08 mL of a 1.0 M solution in toluene, 1.08 mmol, 1.4 eq.), methyl oxolane-2-carboxylate (0.09 mL, 0.77 mmol, 1.0 eq.) and 4-fluorobenzyl bromide (0.13 mL, 1.08 mmol, 1.4 eq.) in THF (5 mL) gave the crude product as a colourless oil. Purification by flash column chromatography on silica with 90:10 hexane-EtOAc as eluent gave aryl THF-2-methyl ester **5a** (89 mg, 49%) as a colourless oil, R_F (90:10 hexane-EtOAc) 0.14; IR (ATR) 2952, 1729 (C=O), 1508, 1218, 1098, 837 cm^{-1}; ^{1}H NMR (400 MHz, $CDCl_3$) δ 7.21-7.18 (m, 2H, Ar), 6.96-6.92 (m, 2H, Ar), 3.95-3.86 (m, 2H, OCH), 3.66 (s, 3H, OMe), 3.17 (d, J = 14.0 Hz, 1H, CHAr), 2.93 (d, J = 14.0 Hz, 1H, CHAr), 2.29-2.23 (m, 1H, CH), 1.93-1.79 (m, 2H, CH), 1.71-1.63 (m, 1H, CH); ^{13}C NMR (100.6 MHz, $CDCl_3$) δ 175.2 (C=O), 162.0 (d, J = 244.5 Hz, *ipso*-Ar), 132.2 (d, J = 3.5 Hz, *ipso*-Ar), 131.8 (d, J = 8.0 Hz, Ar), 115.0 (d, J = 21.0 Hz, Ar), 86.8 (C), 69.4 (OCH_2), 52.3 (OMe), 42.6 (CH_2Ar), 34.9 (CH_2), 25.4 (CH_2); MS (ESI) *m/z* 261 (M + Na)$^+$; HRMS *m/z* calcd for $C_{13}H_{15}FO_3$ (M + Na)$^+$ 261.0897, found 261.0892 (+2.3 ppm error).

Lab Book Reference: PJ-07-22.

Methyl 2-[(4-fluorophenyl)methyl]oxane-2-carboxylate 5b

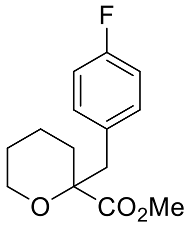

5b

Using general procedure F, LiHMDS (1.08 mL of a 1.0 M solution in toluene, 1.08 mmol, 1.4 eq.), methyl oxane-2-carboxylate (0.1 mL, 0.77 mmol, 1.0 eq.) and 4-fluorobenzyl bromide (0.13 mL, 1.08 mmol, 1.4 eq.) in THF (5 mL) gave the crude product as a colourless oil. Purification by flash column chromatography on silica with 90:10 hexane-EtOAc as eluent gave aryl THP-2-methyl ester **5b** (151 mg, 77%) as a colourless oil, R_F (90:10 hexane-EtOAc) 0.19; IR (ATR) 2947, 1729 (C=O), 1508, 1218, 1073, 1047 cm^{-1}; ^{1}H NMR (400 MHz, $CDCl_3$) δ 7.14-7.10 (m, 2H, Ar), 6.96-6.92 (m, 2H, Ar), 3.92-3.87 (m,

1H, OCH), 3.67-3.60 (m, 4H, OMe and OCH), 2.91 (s, 2H, CHAr), 2.19-2.15 (m, 1H, CH), 1.74-1.70 (m, 1H, CH), 1.58-1.36 (m, 4H, CH); ^{13}C NMR (100.6 MHz, $CDCl_3$) δ 173.9 (C=O), 162.0 (d, J = 244.5 Hz, *ipso*-Ar), 131.8 (d, J = 8.0 Hz, Ar), 131.6 (d, J = 3.5 Hz, *ipso*-Ar), 114.9 (d, J = 21.0 Hz, Ar), 81.0 (C), 65.2 (OCH_2), 51.9 (OMe), 45.9 (CH_2Ar), 32.5 (CH_2), 25.2 (CH_2), 20.8 (CH_2); MS (ESI) *m/z* 275 (M + Na)$^+$; HRMS *m/z* calcd for $C_{14}H_{17}FO_3$ (M + Na)$^+$ 275.1054, found 275.1051 (+1.2 ppm error).
Lab Book Reference: PJ-07-35.

Methyl 4-[(2-bromophenyl)methyl]oxane-4-carboxylate 5c

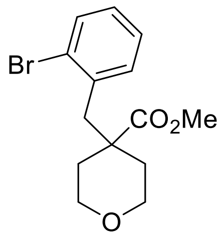

5c

Using general procedure F, LiHMDS (2.1 mL of a 1.0 M solution in toluene, 2.1 mmol, 1.4 eq.), methyl oxane-4-carboxylate (0.2 mL, 1.5 mmol, 1.0 eq.) in THF (10 mL) and 2-bromobenzyl bromide (525 mg, 2.1 mmol, 1.4 eq.) in THF (5 mL) gave the crude product as a colourless oil. Purification by flash column chromatography on silica with 19:1 CH_2Cl_2-acetone as eluent gave aryl THP-4-methyl ester **5c** (368 mg, 76%) as a white solid, mp 44-46 °C; R_F (19:1 CH_2Cl_2-acetone) 0.25; IR (ATR) 2956, 1713 (C=O), 1438, 1210, 1134, 1104, 774 cm^{-1}; ^{1}H NMR (400 MHz, $CDCl_3$) δ 7.54-7.52 (m, 1H, Ar), 7.25-7.18 (m, 1H, Ar), 7.09-7.04 (m, 2H, Ar), 3.87-3.83 (m, 2H, OCH), 3.69 (s, 3H, OMe), 3.35 (ddd, J = 12.0, 12.0, 2.0 Hz, 2H, OCH), 3.06 (s, 2H, CH_2Ar), 2.11-2.06 (m, 2H, CH), 1.72 (ddd, J = 13.5, 12.0, 4.5 Hz, 2H, CH); ^{13}C NMR (100.6 MHz, $CDCl_3$) δ 175.5 (C=O), 136.2 (*ipso*-Ar), 133.2 (Ar), 131.9 (Ar), 128.6 (Ar), 127.1 (Ar), 125.9 (*ipso*-Ar), 65.7 (OCH_2), 52.1 (OMe), 47.3 (C), 45.4 (CH_2Ar), 34.2 (CH_2); MS (ESI) *m/z* 335 (M + Na)$^+$; HRMS *m/z* calcd for $C_{14}H_{17}{}^{79}BrO_3$ (M + Na)$^+$ 335.0253, found 335.0249 (–1.7 ppm error).
Lab Book Reference: PJ-08-12.

1-*tert*-Butyl 2-methyl 2-[(4-fluorophenyl)methyl]pyrrolidine-1,2-dicarboxylate 5d

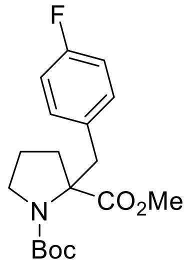

5d

Using general procedure F, LiHMDS (0.42 mL of a 1.0 M solution in toluene, 0.42 mmol, 1.4 eq.), *N*-Boc pyrrolidine-2-methyl ester **3k** (69 mg, 0.3 mmol, 1.0 eq.) and 4-fluorobenzyl bromide (0.052 mL, 0.42 mmol, 1.4 eq.) in THF (5 mL) gave the crude product as a colourless oil. Purification by flash column chromatography on silica with 90:10 hexane-EtOAc as eluent gave *N*-Boc aryl pyrrolidine-2-methyl ester **5d** (89 mg, 86%) as a colourless oil, R_F (90:10 hexane-EtOAc) 0.14; IR (ATR) 2976, 1739 (C=O, CO_2Me), 1692 (C=O, Boc), 1509, 1386, 1158 cm^{-1}; ^1H NMR (400 MHz, $CDCl_3$) (60:40 mixture of rotamers) δ 7.12-7.07 (m, 2H, Ar), 6.99-6.94 (m, 2H, Ar), 3.75 (s, 3H, OMe), 3.74-3.68 (m, 0.4H, CHAr), 3.54-3.49 (m, 1.2H, CHAr and NCH), 3.40 (ddd, J = 10.5, 7.0, 7.0 Hz, 0.4H, NCH), 3.08-2.96 (m, 1.6H, CHAr and NCH), 2.89 (ddd, J = 10.5, 7.5, 5.5 Hz, 0.4H, NCH), 2.07-1.99 (m, 2H, CH), 1.65-1.58 (m, 1H, CH), 1.50 (s, 3.6H, CMe_3), 1.48 (s, 5.4H, CMe_3), 1.04-0.99 (m, 0.4H, CH), 0.98-0.89 (m, 0.6H, CH); ^{13}C NMR (100.6 MHz, $CDCl_3$) (rotamers) δ 175.3 (C=O, CO_2Me), 175.2 (C=O, CO_2Me), 163.2 (d, J = 245.0 Hz, *ipso*-Ar), 160.9 (d, J = 244.5 Hz *ipso*-Ar), 154.3 (C=O, Boc), 153.7 (C=O, Boc), 133.1 (d, J = 3.0 Hz, *ipso*-Ar), 132.7 (d, J = 3.0 Hz, *ipso*-Ar), 132.3 (d, J = 7.5 Hz, Ar), 132.2 (d, J = 7.5 Hz, Ar), 115.3 (d, J = 21.0 Hz, Ar), 115.0 (d, J = 21.0 Hz, Ar), 80.6 (O*C*Me_3), 79.9 (O*C*Me_3), 68.4 (C), 68.1 (C), 52.5 (OMe), 52.4 (OMe), 48.4 (NCH_2), 48.4 (NCH_2), 39.1 (CH_2Ar), 37.8 (CH_2Ar), 36.7 (CH_2), 35.5 (CH_2), 28.6 (CMe_3), 28.5 (CMe_3), 22.9 (CH_2), 22.4 (CH_2); MS (ESI) *m/z* 360 $(M + Na)^+$; HRMS *m/z* calcd for $C_{18}H_{24}FNO_4$ $(M + Na)^+$ 360.1582, found 360.1574 (+1.9 ppm error).

Lab Book Reference: PJ-07-29.

1-*tert*-Butyl 3-methyl 3-[(4-fluorophenyl)methyl]pyrrolidine-1,3-dicarboxylate 5e

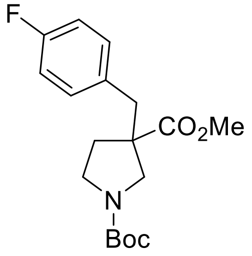

5e

Using general procedure F, LiHMDS (0.61 mL of a 1.0 M solution in toluene, 0.61 mmol, 1.4 eq.), *N*-Boc pyrrolidine-3-methyl ester **3c** (100 mg, 0.43 mmol, 1.0 eq.) and 4-fluorobenzyl bromide (0.08 mL, 0.61 mmol, 1.4 eq.) in THF (5 mL) gave the crude product as a colourless oil. Purification by flash column chromatography on silica with 90:10 hexane-EtOAc as eluent gave *N*-Boc aryl pyrrolidine-3-methyl ester **5e** (97 mg, 67%) as a colourless oil, R_F (90:10 hexane-EtOAc) 0.16; IR (ATR) 2976, 1732 (C=O, CO_2Me), 1691 (C=O, Boc), 1509, 1398, 1168 cm^{-1}; ^{1}H NMR (400 MHz, $CDCl_3$) (50:50 mixture of rotamers) δ 7.05-6.98 (m, 2H, Ar), 6.97-6.92 (m, 2H, Ar), 3.80-3.77 (m, 0.5H, NCH), 3.67-3.65 (m, 3.5H, OMe and NCH), 3.48-3.44 (m, 0.5H. NCH), 3.37-3.30 (m, 2.5H, NCH), 3.01-2.90 (m, 2H, CH_2Ar), 2.31-2.24 (m, 1H, CH), 1.91-1.84 (m, 1H, CH), 1.46-1.44 (m, 9H, CMe_3); ^{13}C NMR (100.6 MHz, $CDCl_3$) (rotamers) δ 174.7 (C=O, CO_2Me), 174.6 (C=O, CO_2Me), 162.1 (d, J = 245.5 Hz, *ipso*-Ar), 154.5 (C=O, Boc), 154.4 (C=O, Boc), 132.8 (*ipso*-Ar), 131.0 (d, J = 8.0 Hz, Ar), 115.4 (d, J = 21.0 Hz, Ar), 79.7 (O*C*Me_3), 79.6 (O*C*Me_3), 54.7 (C), 54.0 (C), 53.1 (NCH_2), 52.2 (OMe), 44.9 (NCH_2), 44.3 (NCH_2), 41.2 (CH_2Ar), 41.0 (CH_2Ar), 33.7 (CH_2), 32.8 (CH_2), 28.6 (C*Me*$_3$); MS (ESI) *m/z* 360 (M + Na)$^+$; HRMS *m/z* calcd for $C_{18}H_{24}FNO_4$ (M + Na)$^+$ 360.1582, found 360.1577 (+1.5 ppm error).
Lab Book Reference: PJ-07-71.

1-*tert*-Butyl 2-methyl 2-[(2-fluorophenyl)methyl]pyrrolidine-1,2-dicarboxylate 5f

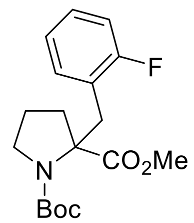

5f

Using general procedure F, LiHMDS (0.61 mL of a 1.0 M solution in toluene, 0.61 mmol, 1.4 eq.), *N*-Boc pyrrolidine-2-methyl ester **3k** (100 mg, 0.43 mmol, 1.0 eq.) and 2-fluorobenzyl bromide (0.07 mL, 0.61 mmol, 1.4 eq.) in THF (5 mL) gave the crude product as a colourless oil. Purification by flash

column chromatography on silica with 90:10 hexane-EtOAc as eluent gave *N*-Boc aryl pyrrolidine-2-methyl ester **5f** (130 mg, 90%) as a colourless oil, R_F (90:10 hexane-EtOAc) 0.11; IR (ATR) 2976, 1740 (C=O, CO_2Me), 1693 (C=O, Boc), 1385, 1165, 1103, 757 cm^{-1}; ^1H NMR (400 MHz, $CDCl_3$) (60:40 mixture of rotamers δ 7.23-7.19 (m, 1H, Ar), 7.14-7.08 (m, 1H, Ar), 7.07-7.00 (m, 2H, Ar), 3.76 (s, 3H, OMe), 3.58 (d, J = 13.5 Hz, 0.4H, CHAr), 3.50-3.35 (m, 2.6H, CHAr and NCH), 3.06-3.04 (m, 0.6H, NCH), 3.04-3.01 (m, 0.4H, NCH), 2.12-2.04 (m, 2H, CH), 1.65-1.60 (m, 1H, CH), 1.51-1.49 (m, 9H, CMe_3), 1.02-0.88 (m, 1H, CH); ^{13}C NMR (100.6 MHz, $CDCl_3$) (rotamers) δ 175.2 (C=O, CO_2Me), 175.1 (C=O, CO_2Me), 161.9 (d, J = 245.0 Hz, *ipso*-Ar), 154.3 (C=O, Boc), 153.7 (C=O, Boc), 133.2 (d, J = 4.5 Hz, Ar), 132.9 (d, J = 4.5 Hz, Ar), 128.6 (d, J = 8.0 Hz, Ar), 128.4 (d, J = 8.0 Hz, Ar), 124.2 (*ipso*-Ar), 124.2 (*ipso*-Ar), 124.0 (d, J = 4.0 Hz, Ar), 123.9 (d, J = 4.0 Hz, Ar), 115.4 (d, J = 22.5 Hz, Ar), 115.1 (d, J = 22.5 Hz, Ar), 80.5 (O*C*Me3), 79.8 (O*C*Me3), 68.7 (C), 68.2 (C), 52.5 (OMe), 52.4 (OMe), 48.3 (NCH_2), 48.3 (NCH_2), 36.8 (CH_2), 35.6 (CH_2), 32.6 (CH_2Ar), 31.4 (CH_2Ar), 28.6 (C*Me3*), 28.5 (C*Me3*), 23.2 (CH_2), 22.6 (CH_2); MS (ESI) *m/z* 360 $(M + Na)^+$; HRMS *m/z* calcd for $C_{18}H_{24}FNO_4$ $(M + Na)^+$ 360.1582, found 360.1576 (+1.7 ppm error).

Lab Book Reference: PJ-07-64.

1-*tert*-Butyl 2-methyl 2-[(2-bromophenyl)methyl]pyrrolidine-1,2-dicarboxylate 5g

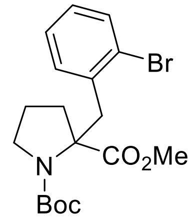

5g

Using general procedure F, LiHMDS (3.19 mL of a 1.0 M solution in toluene, 3.19 mmol, 1.4 eq.), *N*-Boc pyrrolidine-2-methyl ester **3k** (500 mg, 2.28 mmol, 1.0 eq.) and 2-bromobenzyl bromide (796 mL, 3.19 mmol, 1.4 eq.) in THF (5 mL) gave the crude product as a colourless oil. Purification by flash column chromatography on silica with 90:10 hexane-EtOAc as eluent gave *N*-Boc aryl pyrrolidine-2-methyl ester **5g** (813 mg, 89%) as a colourless oil, R_F (90:10 hexane-EtOAc) 0.19; IR (ATR) 2974, 1739 (C=O, CO_2Me), 1692 (C=O, Boc), 1384, 1163, 1024, 759 cm^{-1}; ^1H NMR (400 MHz, $CDCl_3$) (60:40 mixture of rotamers) δ 7.56-7.52 (m, 1H, Ar), 7.22-7.20 (m, 1H, Ar), 7.16-7.13 (m, 1H, Ar), 7.11-7.07 (m, 1H, Ar), 3.76-3.71 (m, 3H, OMe), 3.69-3.65 (m, 1.4H, CHAr and NCH), 3.54-3.47 (m, 1.6H, CHAr

and NCH), 3.06-3.05 (m, 0.6H, NCH), 3.04-3.03 (m, 0.4H, NCH), 2.23-2.17 (m, 1H, CH), 2.06-2.01 (m, 1H, CH), 1.68-1.63 (m, 1H, CH), 1.61-1.50 (m, 3.6H, CMe_3), 1.50-1.47 (m, 5.4H, CMe_3), 1.01-0.96 (m, 1H, CH); ^{13}C NMR (100.6 MHz, $CDCl_3$) (rotamers) δ 175.0 (C=O, CO_2Me), 174.9 (C=O, CO_2Me), 154.4 (C=O, Boc), 153.7 (C=O, Boc), 137.5 (*ipso*-Ar), 137.1 (*ipso*-Ar), 133.1 (Ar), 132.9 (Ar), 132.9 (Ar), 132.6 (Ar), 128.5 (Ar), 128.3 (Ar), 127.6 (Ar), 127.3 (Ar), 126.4 (*ipso*-Ar), 80.6 (O*C*Me_3), 79.9 (O*C*Me_3), 69.2 (C), 68.9 (C), 52.5 (OMe), 52.5 (OMe), 48.3 (NCH_2), 48.2 (NCH_2), 38.2 (CH_2Ar), 37.1 (CH_2Ar), 36.4 (CH_2), 35.3 (CH_2), 28.6 (C*Me₃*), 28.5 (C*Me₃*), 23.2 (CH_2), 22.6 (CH_2); MS (ESI) *m/z* 420 $(M + Na)^+$; HRMS *m/z* calcd for $C_{18}H_{24}{}^{79}BrNO_4$ $(M + Na)^+$ 420.0781, found 420.0774 (+0.6 ppm error).

Lab Book Reference: PJ-08-09.

1-*tert*-Butyl 2-methyl 2-[(3-fluorophenyl)methyl]pyrrolidine-1,2-dicarboxylate 5h

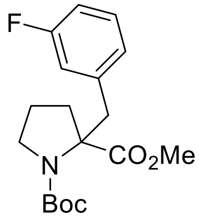

5h

Using general procedure F, LiHMDS (0.61 mL of a 1.0 M solution in toluene, 0.61 mmol, 1.4 eq.), *N*-Boc pyrrolidine-2-methyl ester **3k** (100 mg, 0.43 mmol, 1.0 eq.) and 3-fluorobenzyl bromide (0.075 mL, 0.61 mmol, 1.4 eq.) in THF (5 mL) gave the crude product as a colourless oil. Purification by flash column chromatography on silica with 90:10 hexane-EtOAc as eluent gave *N*-Boc aryl pyrrolidine-2-methyl ester **5h** (133 mg, 92%) as a colourless oil, R_F (90:10 hexane-EtOAc) 0.13; IR (ATR) 2976, 1739 (C=O, CO_2Me), 1691 (C=O, Boc), 1386, 1247, 1163 cm^{-1}; ^{1}H NMR (400 MHz, $CDCl_3$) (60:40 mixture of rotamers) δ 7.25-7.20 (m, 1H, Ar), 6.97-6.91 (m, 2H, Ar), 6.88-6.85 (m, 1H, Ar), 3.79-3.75 (m, 0.4H, CHAr), 3.74 (s, 3H, OMe), 3.55 (d, J = 14.0 Hz, 0.6H, CHAr), 3.52-3.45 (m, 0.6H, NCH), 3.40 (ddd, J = 10.5, 7.0, 7.0 Hz, 0.4H, NCH), 3.08-3.01 (m, 1.6H, CHAr and NCH), 2.91 (ddd, J = 10.5, 7.5, 6.0 Hz, 0.4H, NCH), 2.09-2.01 (m, 2H, CH), 1.66-1.58 (m, 1H, CH), 1.51 (s, 3.6H, CMe_3), 1.49 (s, 5.4H, CMe_3), 1.06-0.87 (m, 1H, CH); ^{13}C NMR (100.6 MHz, $CDCl_3$) (rotamers) δ 175.2 (C=O, CO_2Me), 175.1 (C=O, CO_2Me), 162.8 (d, J = 245.5 Hz, *ipso*-Ar), 154.4 (C=O, Boc), 153.6 (C=O, Boc), 140.0 (d, J = 7.5 Hz, *ipso*-Ar), 139.6 (d, J = 7.5 Hz, *ipso*-Ar), 129.8 (d, J = 8.0 Hz, Ar), 129.4 (d, J = 8.0 Hz, Ar), 126.6 (d, J

= 3.0 Hz, Ar), 126.5 (d, J = 3.0 Hz, Ar), 117.8 (d, J = 2.5 Hz, Ar), 117.6 (d, J = 2.5 Hz, Ar), 113.8 (d, J = 21.0 Hz, Ar), 113.5 (d, J = 21.0 Hz, Ar), 80.7 (O*C*Me3), 80.0 (O*C*Me3), 68.3 (C), 68.0 (C), 52.5 (OMe), 52.4 (OMe), 48.4 (NCH_2), 39.8 (CH_2Ar), 38.5 (CH_2Ar), 36.8 (CH_2), 35.5 (CH_2), 28.6 (C*Me3*), 28.5 (C*Me3*), 22.9 (CH_2), 22.4 (CH_2); MS (ESI) *m/z* 360 (M + Na)$^+$; HRMS *m/z* calcd for $C_{18}H_{24}FNO_4$ (M + Na)$^+$ 360.1582, found 360.1579 (+0.1 ppm error).

Lab Book Reference: PJ-07-68.

1-*tert*-Butyl 2-methyl 2-[(3-methoxyphenyl)methyl]pyrrolidine-1,2-dicarboxylate 5i

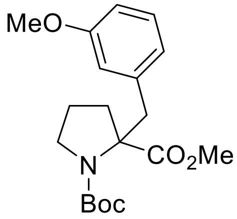

5i

Using general procedure F, LiHMDS (0.61 mL of a 1.0 M solution in toluene, 0.61 mmol, 1.4 eq.), *N*-Boc pyrrolidine-2-methyl ester **3k** (100 mg, 0.43 mmol, 1.0 eq.) and 3-methoxybenzyl bromide (0.085 mL, 0.61 mmol, 1.4 eq.) in THF (5 mL) gave the crude product as a colourless oil. Purification by flash column chromatography on silica with 90:10 hexane-EtOAc as eluent gave *N*-Boc aryl pyrrolidine-2-methyl ester **5i** (103 mg, 68%) as a colourless oil, R_F (90:10 hexane-EtOAc) 0.12; IR (ATR) 2974, 1739 (C=O, CO_2Me), 1692 (C=O, Boc), 1388, 1249, 1166, 1118 cm^{-1}; ^{1}H NMR (400 MHz, $CDCl_3$) (60:40 mixture of rotamers) δ 7.20-7.15 (m, 1H, Ar), 6.80-6.67 (m, 3H, Ar), 3.78 (s, 1.2H, OMe), 3.77 (s, 1.8H, OMe), 3.75 (s, 3H, OMe), 3.74-3.71 (m, 0.4H, CHAr), 3.56 (d, J = 14.0 Hz, 0.6H, CHAr), 3.49 (ddd, J = 10.5, 7.5, 7.5 Hz, 0.6H, NCH), 3.41 (ddd, J = 10.5, 7.5, 7.5 Hz, 0.4H, NCH), 3.04-3.02 (m, 1.6H, CHAr and NCH), 2.99-2.97 (m, 0.4H, NCH), 2.11-2.01 (m, 2H, CH), 1.63-1.56 (m, 1H, CH), 1.50 (s, 3.6H, CMe_3), 1.49 (s, 5.4H, CMe_3), 0.99-0.94 (m, 1H, CH); ^{13}C NMR (100.6 MHz, $CDCl_3$) (rotamers) δ 175.4 (C=O, CO_2Me), 175.3 (C=O, CO_2Me), 159.5 (*ipso*- Ar), 159.4 (*ipso*-Ar), 154.2 (C=O, Boc), 153.6 (C=O, Boc), 139.0 (*ipso*-Ar), 138.6 (*ipso*-Ar), 129.3 (Ar), 129.0 (Ar), 123.5 (Ar), 123.2 (Ar), 116.4 (Ar), 116.1 (Ar), 112.5 (Ar), 112.2 (Ar), 80.4 (O*C*Me3), 79.7 (O*C*Me3), 68.5 (C), 68.1 (C), 55.3 (OMe), 55.2 (OMe), 52.5 (OMe), 52.4 (OMe), 48.4 (NCH_2), 48.3 (NCH_2), 39.9 (CH_2Ar), 38.6 (CH_2Ar), 36.8 (CH_2), 35.5 (CH_2), 28.6 (C*Me3*), 28.4 (C*Me3*), 23.0 (CH_2), 22.4 (CH_2); MS (ESI) *m/z* 372 (M + Na)$^+$; HRMS *m/z* calcd for $C_{19}H_{27}NO_5$ (M + Na)$^+$ 372.1781, found 372.1780 (+0.5 ppm error).

Lab Book Reference: PJ-07-59.

1-*tert*-Butyl 2-methyl 2-[(4-methoxyphenyl)methyl]pyrrolidine-1,2-dicarboxylate 5j

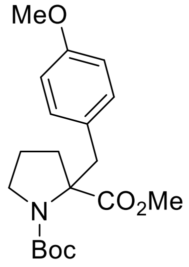

5j

Using general procedure F, LiHMDS (0.61 mL of a 1.0 M solution in toluene, 0.61 mmol, 1.4 eq.), *N*-Boc pyrrolidine-2-methyl ester **3k** (100 mg, 0.43 mmol, 1.0 eq.) and 4-methoxybenzyl bromide (123 mg, 0.61 mmol, 1.4 eq.) in THF (5 mL) gave the crude product as a colourless oil. Purification by flash column chromatography on silica with 90:10 hexane-EtOAc as eluent gave *N*-Boc aryl pyrrolidine-2-methyl ester **5j** (137 mg, 91%) as a colourless oil, R_F (90:10 hexane-EtOAc) 0.09; IR (ATR) 2973, 1738 (C=O, CO_2Me), 1691 (C=O, Boc) 1512, 1387, 1246, 1166 cm^{-1}; ^1H NMR (400 MHz, MeOD-d_4) (55:45 mixture of rotamers) δ 7.07-7.03 (m, 2H, Ar), 6.83-6.80 (m, 2H, Ar), 3.79 (s, 1.35H, OMe), 3.78 (s, 1.65H, OMe), 3.74 (s, 1.65H, OMe), 3.71 (s, 1.35H, OMe), 3.58 (d, J = 14.0 Hz, 0.45H, CHAr), 3.46 (d, J = 14.0 Hz, 0.55H, CHAr), 3.42-3.36 (m, 1H, NCH), 2.97 (d, J = 14.0 Hz, 0.55H, CHAr), 2.96 (d, J = 14.0 Hz, 0.45H, CHAr), 2.88 (ddd, J = 10.0, 7.5, 5.0, Hz, 1H, NCH), 2.09-1.95 (m, 2H, CH), 1.69-1.53 (m, 1H, CH), 1.50 (s, 4.05H, CMe_3), 1.48 (s, 4.95H, CMe_3), 1.04-0.89 (m, 1H, CH); ^{13}C NMR (100.6 MHz, $CDCl_3$) (rotamers) δ 175.5 (C=O, CO_2Me), 175.4 (C=O, CO_2Me), 158.6 (*ipso*-Ar), 158.4 (*ipso*-Ar), 154.3 (C=O, Boc), 153.7 (C=O, Boc), 131.8 (Ar), 131.7 (Ar), 129.4 (*ipso*-Ar), 128.9 (*ipso*-Ar), 113.8 (Ar), 113.6 (Ar), 80.4 (O*C*Me_3), 79.7 (O*C*Me_3), 68.5 (C), 68.2 (C), 55.3 (OMe), 52.4 (OMe), 52.3 (OMe), 48.4 (NCH_2), 48.3 (NCH_2), 38.9 (CH_2Ar), 37.7 (CH_2Ar), 36.7 (CH_2), 35.5 (CH_2), 28.6 (C*Me_3*), 28.5 (C*Me_3*), 23.0 (CH_2), 22.4 (CH_2); MS (ESI) *m/z* 372 $(M + Na)^+$; HRMS *m/z* calcd for $C_{19}H_{27}NO_5$ $(M + Na)^+$ 372.1781, found 372.1775 (+1.5 ppm error).

Lab Book Reference: PJ-08-15.

1-*tert*-Butyl 2-methyl 2-[(pyridin-2-yl)methyl]pyrrolidine-1,2-dicarboxylate 5k

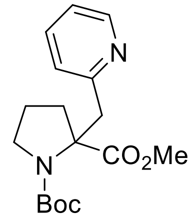

5k

LiHMDS (0.61 mL of a 1.0 M solution in toluene, 0.61 mmol, 1.4 eq.) was added dropwise to a stirred solution of *N*-Boc pyrrolidine-2-methyl ester **3k** (100 mg, 0.43 mmol, 1.0 eq.) in THF (4 mL) at –78 °C under Ar. The resulting solution was stirred at –78 °C for 1.5 h. In a separate flask sat $NaHCO_{3(aq)}$ (5 mL) was added to 2(bromomethyl)pyridine·HBr (153 mg, 0.61 mmol) and the solution was extracted with CH_2Cl_2 (3 × 5 mL). The combined organics were dried (Na_2CO_3) and evaporated under reduced pressure to give a red oil. The oil was taken up into THF (2 mL) and added dropwise to the enolate reaction mixture at –78 °C under Ar. The resulting solution was stirred at –78 °C and slowly warmed up to rt over 16 h. The solution was poured into sat $NH_4Cl_{(aq)}$ (10 mL) and the two layers were separated. The aqueous layer was extracted with EtOAc (3 × 10 mL) and the combined organics were dried ($MgSO_4$) and evaporated under reduced pressure to give the crude product as an orange oil. Purification by flash column chromatography on silica with 60:40 hexane-EtOAc as eluent gave *N*-Boc aryl pyrrolidine-2-methyl ester **5k** (45 mg, 33%) as a colourless oil, R_F (90:10 hexane-EtOAc) 0.11; IR (ATR) 2974, 1739 (C=O, CO_2Me), 1691 (C=O, Boc), 1387, 1162, 1131 cm^{-1}; ^{1}H NMR (400 MHz, $CDCl_3$) (60:40 mixture of rotamers) δ 8.54-8.51 (m, 1H, 6-py), 7.61-7.56 (m, 1H, 4-py), 7.17-7.4 (m, 1H, 3-py), 7.12-7.08 (m, 1H, 5-py), 3.83 (d, J = 13.5 Hz, 0.4H, CHAr), 3.75 (s, 3H, OMe), 3.64 (d, J = 13.5 Hz, 0.6H, CHAr), 3.48 (ddd, J = 10.5, 7.0, 7.0 Hz, 0.6H, NCH), 3.35 (ddd, J = 10.5, 7.0, 7.0 Hz, 0.4H, NCH), 3.32 (d, J = 13.5 Hz, 0.6H, CHAr), 3.30 (d, J = 13.5 Hz, 0.4H, CHAr), 2.88 (ddd, J = 10.5, 7.5, 6.0 Hz, 0.6H, NCH), 2.83 (ddd, J = 10.5, 7.0, 7.0 Hz, 0.4H, NCH), 2.60-2.54 (m, 1H, CH), 2.10-2.05 (m, 1H, CH), 1.69-1.62 (m, 1H, CH), 1.51 (s, 5.6H, CMe_3), 1.50 (s, 3.4H, CMe_3), 0.94 (ddddd, J = 13.5, 7.0, 7.0, 7.0, 7.0 Hz, 0.4H, CH), 0.92-0.84 (m, 0.6H, CH); ^{13}C NMR (100.6 MHz, $CDCl_3$) (rotamers) δ 175.1 (C=O, CO_2Me), 158.0 (*ipso*-Ar), 153.7 (C=O, Boc), 149.1 (Ar), 148.8 (Ar), 136.3 (Ar), 136.1 (Ar), 125.6 (Ar), 125.3 (Ar), 121.8 (Ar), 121.6 (Ar), 80.6 (O*C*Me$_3$), 79.7 (O*C*Me$_3$), 68.4 (C), 68.0 (C), 52.5 (OMe), 52.4 (OMe), 48.4 (NCH_2), 48.3 (NCH_2), 42.2 (CH_2Ar), 41.1 (CH_2Ar), 36.4 (CH_2), 35.4 (CH_2), 28.6 (C*Me$_3$*), 22.9 (CH_2), 22.4 (CH_2); MS (ESI) *m/z* 321 $(M + H)^+$; HRMS *m/z* calcd for $C_{17}H_{25}N_2O_4$ $(M + H)^+$ 321.1809, found 321.1807 (+0.7 ppm error).

Lab Book Reference: PJ-07-48.

Methyl (3*R,4*R**)-4-(2-fluorophenyl)pyrrolidine-3-carboxylate hydrochloride 8a•HCl**

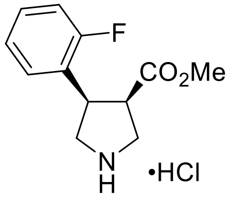

8a•HCl

Using general procedure G, *N*-Boc pyrrolidine **2i** (131 mg, 0.405 mmol, 1.0 eq.) in Et_2O (5 mL) and HCl (2.03 mL of a 2 M solution in Et_2O, 4.05 mmol, 10.0 eq.) gave pyrrolidine **8a**•HCl (105 mg, quant.) as a white solid, mp 81-83 °C; IR (ATR) 1723 (C=O), 1626, 1433 cm^{-1}; ^1H NMR (400 MHz, MeOD-*d*4) δ 7.40-7.34 (m, 1H, Ar), 7.25-7.12 (m, 3H, Ar), 4.21-4.12 (m, 1H, C*H*Ar), 3.91-3.66 (m, 5H, NCH, C*H*CO_2Me), 3.30 (s, 3H, OMe); ^{13}C NMR (100.6 MHz, MeOD-*d*4) δ 173.2 (C=O) 161.7 (d, *J* = 246.0 Hz, CF), 129.3 (d, *J* = 8.5 Hz, Ar), 127.0 (d, *J* = 3.5 Hz, Ar), 125.7 (d, *J* = 3.0 Hz, Ar), 123.4 (d, *J* = 14.5 Hz, *ipso*-Ar), 116.5 (d, *J* = 22.0 Hz, Ar), 48.4 (OMe), 47.9 (NCH_2), 43.3 (NCH_2), 40.0 (C*H*Ar), 36.8 (C*H*CO_2Me); MS (ESI) *m/z* 224 [M^+, 100]; HRMS (ESI) *m/z* calcd for $C_{12}H_{15}FNO_2$ M^+ 224.1081, found 224.1081 (+0.3 ppm error).

Lab Book Reference: TD 5/38

Methyl (3*R,4*R**)-4-(pyrimidin-5-yl)piperidine-3-carboxylate hydrochloride 8b•HCl**

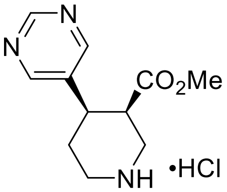

8b•HCl

Using general procedure G, *N*-Boc piperidine **2j** (122 mg, 0.38 mmol, 1.0 eq.) in Et_2O (5 mL) and HCl (1.90 mL of a 2 M solution in Et_2O, 3.80 mmol, 10.0 eq.) gave pyrrolidine **8b**•HCl (90 mg, 92%) as a yellow oil, IR (ATR) 2951, 1729 (C=O), 1494, 1219 cm^{-1}; ^1H NMR (400 MHz, MeOD-*d*4) δ 9.42 (br s, 1H, Ar), 9.18 (br s, 2H, Ar), 3.86-3.70 (m, 1H, C*H*Ar), 3.68-3.63 (m, 2H, NCH), 3.60 (s, 3H, OMe), 3.55-3.48 (m, 3H, NCH, C*H*CO_2Me), 2.52-2.49 (m, 1H, CH), 2.26-2.16 (m, 1H, CH); ^{13}C NMR (100.6 MHz, MeOD-*d*4) δ 172.5 (C=O), 157.8 (Ar), 153.7 (Ar), 121.8 (*ipso*-Ar), 53.0 (OMe), 46.1 (NCH_2),

44.9 (NCH_2), 43.4 (C*H*Ar), 37.5 (C*H*CO_2Me), 23.2 (NCH_2*C*H_2); MS (ESI) *m/z* 222 [M^+, 100]; HRMS (ESI) *m/z* calcd for $C_{11}H_{16}N_3O_2$ M^+ 222.1237, found 222.1236 (+0.6 ppm error).

Lab Book Reference: TD 5/39

Methyl 4-(4-methylphenyl)piperidine-4-carboxylate hydrochloride 8c·HCl

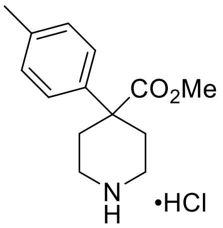

8c•HCl

Using general procedure G, *N*-Boc piperidine **4a** (111 mg, 0.3 mmol, 1.0 eq.) and HCl (1 mL of a 4 M solution in dioxane) gave piperidine **8c**•HCl (80 mg, 99%) as an orange solid, mp 230-232 °C; IR (ATR) 2934, 2730, 1725 (C=O), 1144, 495 cm^{-1}; ^{1}H NMR (400 MHz, $CDCl_3$) δ 9.66 (br s, 1H, NH), 9.52 (br s, 1H, NCH), 7.21-7.14 (m, 4H, Ar), 3.68 (s, 3H, OMe), 3.45-3.40 (m, 2H, NCH), 3.08-3.04 (m, 2H, NCH), 2.70-2.66 (m, 2H, CH), 2.40-2.32 (m, 2H, CH), 2.31 (s, 3H, Me); ^{13}C NMR (100.6 MHz, $CDCl_3$) δ 173.7 (C=O, CO_2Me), 137.9 (*ipso*-Ar), 137.0 (*ipso*-Ar), 129.9 (Ar), 125.5 (Ar), 53.0 (OMe), 47.9 (C), 41.9 (NCH_2), 30.5 (CH_2), 21.1 (C_6H_4*Me*); MS (ESI) *m/z* 234 $(M)^+$; HRMS *m/z* calcd for $C_{14}H_{20}NO_2$ M^+ 234.1489, found 234.1493 (–1.6 ppm error).
Lab Book Reference: PJ-07-98.

Methyl 3-(4-methylphenyl)piperidine-3-carboxylate hydrochloride 8d·HCl

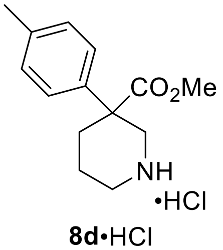

8d•HCl

Using general procedure G, *N*-Boc piperidine **4b** (60 mg, 0.18 mmol, 1.0 eq.) and HCl (1 mL of a 4 M solution in dioxane) gave piperidine **8d**•HCl (47 mg, 97%) as an orange oil, IR (ATR) 2951, 1728 (C=O, CO_2Me), 1449, 1239, 1151, 728 cm^{-1}; ^{1}H NMR (400 MHz, $CDCl_3$) δ 10.52 (s, 1H, NH), 8.65 (s, 1H, NH), 7.17 (s, 4H, Ar), 4.18 (d, J = 13.0 Hz, 1H, NCH), 3.82 (s, 3H, OMe), 3.63-3.60 (m, 1H, NCH), 3.03-3.00 (m, 1H, NCH), 2.97-2.89 (m, 1H, NCH), 2.76-2.73 (m, 1H, CH), 2.32 (s, 3H, C_6H_4*Me*), 1.99-

1.85 (m, 3H, CH); ^{13}C NMR (100.6 MHz, $CDCl_3$) δ 172.9 (C=O), 138.4 (*ipso*-Ar), 135.7 (*ipso*-Ar), 130.0 (Ar), 125.3 (Ar), 53.7 (OMe), 49.8 (NCH_2), 48.7 (C), 43.7 (NCH_2), 30.8 (CH_2), 21.1 (C_6H_4*Me*), 20.4 (CH_2); MS (ESI) *m/z* 234 $(M)^+$; HRMS *m/z* calcd for $C_{14}H_{21}NO_2$ M^+ 234.1489, found 234.1490 (+0.4 ppm error).

Lab Book Reference: PJ-08-89.

Methyl 3-(4-fluorophenyl)piperidine-3-carboxylate hydrochloride 8e·HCl

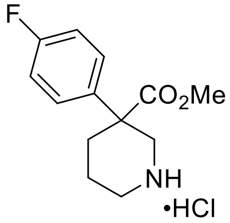

8e•HCl

Using general procedure G, *N*-Boc piperidine **4q** (100 mg, 0.3 mmol, 1.0 eq.) and HCl (1 mL of a 2 M solution in Et_2O) for 40 h gave piperidine **8e**•HCl (82 mg, 100%) as a brown solid, mp 122-124 °C; IR (ATR) 2955, 1728 (C=O), 1513, 1145, 831 cm^{-1}; ^{1}H NMR (400 MHz, $CDCl_3$) δ 10.40-10.38 (m, 1H, NH), 8.75-8.75 (m, 1H, NH), 7.29 (dd, *J* = 9.0, 5.0 Hz, 2H, Ar), 7.05 (dd, *J* = 9.0, 9.0 Hz, 2H, Ar), 4.18 (d, *J* = 13.0 Hz, 1H, NCH), 3.82 (s, 3H, OMe), 3.63-3.59 (m, 1H, NCH), 3.05-2.99 (m, 1H, NCH), 2.99-2.89 (m, 1H, NCH), 2.75-2.71 (m, 1H, CH), 2.01-1.86 (m, 3H, CH); ^{13}C NMR (100.6 MHz, $CDCl_3$) δ 172.6 (C=O), 162.5 (d, *J* = 248.5 Hz, *ipso*-Ar), 134.4 (d, *J* = 3.5 Hz, *ipso*-Ar), 127.4 (d, *J* = 8.5 Hz, Ar), 116.2 (d, *J* = 21.5 Hz, Ar), 53.8 (OMe), 49.7 (NCH_2), 48.5 (C), 43.6 (NCH_2), 30.8 (CH_2), 20.3 (CH_2); MS (ESI) *m/z* 238 $(M)^+$; HRMS *m/z* calcd for $C_{13}H_{17}FNO_2$ M^+ 238.1238, found 238.1237 (–0.3 ppm error).

Lab Book Reference: PJ-07-75.

Methyl 3-(4-methylphenyl)pyrrolidine-3-carboxylate hydrochloride 8f·HCl

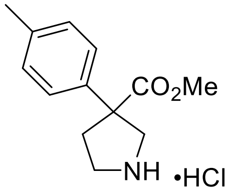

8f•HCl

Using general procedure G, *N*-Boc pyrrolidine **4c** (72 mg, 0.23 mmol, 1.0 eq.) and HCl (1 mL of a 4 M solution in dioxane) gave piperidine **8f**•HCl (58 mg, 99%) as a cream solid, mp 50-52 °C; IR (ATR) 2918, 1728 (C=O), 1212, 729 cm^{-1}; ^1H NMR (400 MHz, $CDCl_3$) δ 10.14-10.14 (m, 1H, NH), 9.95-9.95 (m, 1H, NH), 7.18-7.13 (m, 4H, Ar), 4.40-4.35 (m, 1H, NCH), 3.71 (s, 3H, OMe), 3.58-3.50 (m, 2H, NCH), 3.41-3.39 (m, 1H, NCH), 3.04-2.99 (m, 1H, CH), 2.34-2.29 (m, 1H, CH), 2.29 (s, 3H, C_6H_4*Me*); ^{13}C NMR (100.6 MHz, $CDCl_3$) δ 172.7 (C=O), 138.3 (*ipso*-Ar), 134.7 (*ipso*-Ar), 129.8 (Ar), 126.4 (Ar), 57.5 (C), 53.7 (OMe), 51.8 (NCH_2), 44.3 (NCH_2), 34.2 (CH_2), 21.1 (C_6H_4*Me*); MS (ESI) *m/z* 220 $(M)^+$; HRMS *m/z* calcd for $C_{13}H_{18}NO_2$ M^+ 220.1332, found 220.1333 (–0.2 ppm error).
Lab Book Reference: PJ-07-92.

Methyl 3-[(4-fluorophenyl)methyl]pyrrolidine-3-carboxylate hydrochloride 8g·HCl

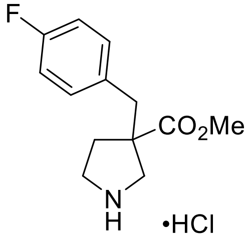

8g•HCl

Using general procedure G, *N*-Boc pyrrolidine **5e** (200 mg, 0.62 mmol, 1.0 eq.) and HCl (2 mL of a 4 M solution in dioxane) gave pyrrolidine **8g**•HCl (157 mg, 93%) as a white foam, IR (ATR) 2952, 2739, 1730 (C=O), 1604, 1509, 1448, 1218, 1160, 1102, 842, 761 cm^{-1}; ^1H NMR (400 MHz, MeOD-*d*4) δ 7.21–7.18 (m, 2H, Ar), 7.06–7.02 (m, 2H, Ar), 3.76 (s, 3H, OMe), 3.69 (d, J = 12.0 Hz, 1H, CH_2), 3.52–3.45 (m, 1H, CH_2), 3.32–3.24 (m, 2H, CH_2), 3.21 (d, J = 14.0 Hz, 1H, CH_2), 3.08 (d, J = 14.0 Hz, 1H, CH_2), 2.50–2.44 (m, 1H, CH_2), 2.20–2.18 (m, 1H, CH_2); ^{13}C NMR (101 MHz, MeOD-*d*4) δ 174.7 (C=O), 163.6 (d, J = 245.0 Hz, *ipso*-Ar), 133.7 (d, J = 3.0 Hz, *ipso*-Ar), 132.3 (d, J = 8.0 Hz, Ar), 116.4 (d, J = 21 Hz, Ar), 56.3 (*C*CO_2Me), 53.3 (OMe), 52.4 (CH_2), 45.7 (CH_2), 41.5 (CH_2), 34.7 (CH_2); HRMS (ESI) *m/z* calcd for $C_{13}H_{17}FNO_2$ M^+ 238.1238, found 238.1239 (–0.6 ppm error).

Lab Book Reference: JDF_B_397_8

Methyl 2-[(2-fluorophenyl)methyl]pyrrolidine-2-carboxylate hydrochloride 8h·HCl

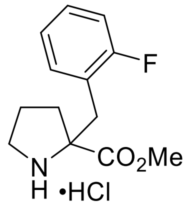

8h•HCl

Using general procedure G, *N*-Boc pyrrolidine **5f** (109 mg, 0.32 mmol, 1.0 eq.) and HCl (1 mL of a 4 M solution in dioxane) gave pyrrolidine **8h**•HCl (82 mg, 94%) as a white solid, mp 265 °C (decomp.); IR (ATR) 2954, 2660, 2484, 1744 (C=O), 1617, 1494, 1454, 1281, 1231, 1181, 1109, 762 cm^{-1}; ^{1}H NMR (400 MHz, MeOD-*d*4) δ 7.39–7.30 (m, 2H, Ar), 7.19–7.11 (m, 2H, Ar), 3.76 (s, 3H, OMe), 3.54–3.48 (m, 2H, CH_2Ar + NCH_2), 3.36–3.28 (m, 2H, CH_2Ar + NCH_2), 2.61–2.56 (m, 1H, CH_2), 2.27–2.17 (m, 2H, CH_2), 2.00–1.95 (m, 1H, CH_2); ^{13}C NMR (101 MHz, MeOD-*d*4) δ 170.9 (C=O), 162.6 (d, J = 244.0 Hz, *ipso*-Ar), 133.2 (d, J = 4.0 Hz, Ar), 131.6 (d, J = 8.5 Hz, Ar), 126.0 (d, J = 3.5 Hz, Ar), 122.1 (d, J = 16.0 Hz, *ipso*-Ar), 116.7 (d, J = 22.5 Hz, Ar), 74.9 (NC), 54.2 (OMe), 46.4 (CH_2), 35.2 (CH_2), 35.1 (CH_2), 22.6 (CH_2); HRMS (ESI) *m/z* calcd for $C_{13}H_{17}FNO_2$ M^+ 238.1238, found 238.1239 (–0.6 ppm error).

Lab Book Reference: JDF_B_397_12

Methyl 2-[(3-fluorophenyl)methyl]pyrrolidine-2-carboxylate hydrochloride 8i·HCl

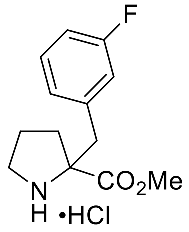

8i•HCl

Using general procedure G, *N*-Boc pyrrolidine **5h** (125 mg, 0.37 mmol, 1.0 eq.) and HCl (1 mL of a 4 M solution in dioxane) gave pyrrolidine **8i**•HCl (98 mg, 97%) as a white solid, mp 162–163 °C; IR (ATR) 2955, 2663, 1742 (C=O), 1615, 1588, 1449, 1252, 1223, 1146, 696, 521 cm^{-1}; ^{1}H NMR (400 MHz, MeOD-*d*4) δ 7.40–7.34 (m, 1H, Ar), 7.08–7.01 (m, 3H, Ar), 3.63 (s, 3H, OMe), 3.56–3.47 (m, 2H, CH_2Ar

+ NCH_2), 3.38–3.31 (m, 1H, NCH_2), 3.32 (d, *J* = 14.5 Hz, 1H, C*H*$_2$Ar), 2.61–2.56 (m, 1H, CH_2), 2.24–2.16 (m, 2H, CH_2), 2.07–1.93 (m, 1H, CH_2); ^{13}C NMR (101 MHz, MeOD-*d*4) *δ* 171.2 (C=O), 164.4 (d, *J* = 245.5 Hz, *ipso*-Ar), 137.8 (d, *J* = 7.5 Hz, *ipso*-Ar), 132.0 (d, *J* = 8.5 Hz, Ar), 126.5 (d, *J* = 3.0 Hz, Ar), 117.4 (d, *J* = 22.0 Hz, Ar), 116.0 (d, *J* = 21.0 Hz, Ar), 75.3 (NC), 54.2 (OMe), 46.6 (CH_2), 41.1 (CH_2), 36.0 (CH_2), 22.9 (CH_2); HRMS (ESI) *m/z* calcd for $C_{13}H_{17}FNO_2$ M^+ 238.1238, found 238.1236 (+0.7 ppm error).

Lab Book Reference: JDF_B_397_11

Methyl 2-[(3-methoxyphenyl)methyl]pyrrolidine-2-carboxylate hydrochloride 8j·HCl

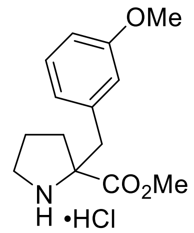

8j•HCl

Using general procedure G, *N*-Boc pyrrolidine **5i** (97 mg, 0.28 mmol, 1.0 eq.) and HCl (1 mL of a 4 M solution in dioxane) gave pyrrolidine **8j**•HCl (73 mg, 91%) as a white solid, mp 159–160 °C; IR (ATR) 2953, 2668, 1743 (C=O), 1602, 1454, 1438, 1288, 1264, 1227, 1039 cm^{-1}; ^{1}H NMR (400 MHz, MeOD-*d*4) *δ* 7.28 (dd, *J* = 8.0, 8.0 Hz, 1H, Ar), 6.89 (dd, *J* = 8.0, 2.5 Hz, 1H, Ar), 6.85–6.83 (m, 1H, Ar), 6.80 (d, *J* = 8.0 Hz, 1H, Ar), 3.84 (s, 3H, OMe), 3.80 (s, 3H, OMe), 3.54–3.47 (m, 2H, C*H*$_2$Ar + NCH_2), 3.39–3.30 (m, 1H, NCH_2), 3.22 (d, *J* = 14.5 Hz, 1H, C*H*$_2$Ar), 2.64–2.59 (m, 1H, CH_2), 2.27–2.17 (m, 2H, CH_2), 2.01–1.92 (m, 1H, CH_2); ^{13}C NMR (101 MHz, MeOD-*d*4) *δ* 171.3 (C=O), 161.6 (*ipso*-Ar), 136.5 (*ipso*-Ar), 131.3 (Ar), 122.4 (Ar), 116.1 (Ar), 114.6 (Ar), 75.5 (NCH), 55.7 (CH_3), 54.1 (OMe), 46.6 (NCH_2), 41.7 (*C*H$_2$Ar), 36.1 (CH_2), 22.9 (CH_2); HRMS (ESI) *m/z* calcd for $C_{14}H_{20}NO_3$ M^+ 250.1438, found 250.1434 (+1.4 ppm error).

Lab Book Reference: JDF_B_396

Methyl 2-[(4-methoxyphenyl)methyl]pyrrolidine-2-carboxylate hydrochloride 8k·HCl

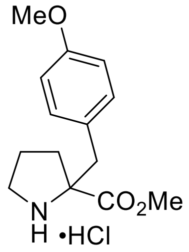

8k•HCl

Using general procedure G, *N*-Boc pyrrolidine **5j** (50 mg, 0.14 mmol, 1.0 eq.) and HCl (1 mL of a 4 M solution in dioxane) gave pyrrolidine **8k**•HCl (39 mg, 99%) as a white solid, mp 172-174 °C; IR (ATR) 2884, 2704, 1755 (C=O), 1513, 1255, 1202, 1031 cm^{-1}; ^{1}H NMR (400 MHz, $CDCl_3$) δ 11.55 (s, 1H, NH), 8.24 (s, 1H, NH), 7.32-7.27 (m, 2H, Ar), 6.84-6.79 (m, 2H, Ar), 3.80-3.68 (m, 8H, OMe, NCH and CHAr), 3.47-3.34 (m, 2H, NCH and CHAr), 2.57-2.46 (m, 1H, CH), 2.35-2.15 (m, 2H, CH), 1.95-1.81 (m, 1H, CH); ^{13}C NMR (100.6 MHz, $CDCl_3$) δ 170.5 (C=O), 159.3 (*ipso*-Ar), 130.8 (Ar), 125.8 (*ipso*-Ar), 114.4 (Ar), 74.7 (C), 55.3 (OMe), 53.7 (OMe), 45.5 (NCH_2), 39.5 (CH_2Ar), 34.1 (CH_2), 22.3 (CH_2); MS (ESI) *m/z* 250 (M)$^+$; HRMS *m/z* calcd for $C_{14}H_{20}NO_3$ M$^+$ 250.1438, found 250.1436 (+0.7 ppm error). Lab Book Reference: PJ-08-15.

Methyl 2-[(pyridin-2-yl)methyl]pyrrolidine-2-carboxylate dihydrochloride 8l·HCl

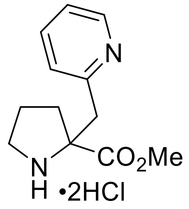

8l•2HCl

Using general procedure G, *N*-Boc pyrrolidine **5k** (45 mg, 0.14 mmol, 1.0 eq.) and HCl (1 mL of a 4 M solution in dioxane) gave pyrrolidine **8l**•HCl (41 mg, 99%) as a white solid, mp 88–90 °C; IR (ATR) 2667, 1743 (C=O), 1619, 1440, 1229, 768, 628, 598 cm^{-1}; ^{1}H NMR (400 MHz, MeOD-*d*4) δ 8.82 (br d, J = 5.5 Hz, 1H, Ar), 8.49 (br t, J = 8.0 Hz, 1H, Ar), 7.98 (br d, J = 8.0 Hz, 1H, Ar), 7.95–7.92 (m, 1H, Ar), 3.95 (d, J = 15.5 Hz, 1H, CH_2Ar), 3.90 (d, J = 15.5 Hz, 1H, CH_2Ar), 3.83 (s, 3H, OMe), 3.66–3.50 (m, 2H, NCH_2), 2.55–2.51 (m, 1H, CH_2), 2.44–2.37 (m, 1H, CH_2), 2.30–2.27 (m, 1H, CH_2), 2.11–2.06 (m, 1H, CH_2); ^{13}C NMR (101 MHz, MeOD-*d*4) δ 170.6 (C=O), 151.8 (*ipso*-Ar), 146.4 (Ar), 145.0 (Ar),

128.9 (Ar), 127.1 (Ar), 72.6 (NCH), 54.9 (OMe), 47.1 (CH_2), 39.0 (CH_2), 36.1 (CH_2), 23.4 (CH_2); HRMS (ESI) *m/z* calcd for $C_{12}H_{16}N_2O_2$ M^+ 221.1285, found 221.1283 (+0.8 ppm error).

Lab Book Reference: JDF_B_395

Methyl (3*R,4*R**)-1-acetyl-4-phenylpyrrolidine-3-carboxylate 8m**

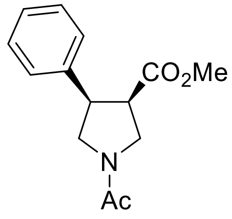

8m

TFA (2.0 mL, 26.1 mmol, 32.0 eq.) was added dropwise to a stirred solution of pyrrolidine **2h** (253 mg, 0.83 mmol, 1.0 eq.) in CH_2Cl_2 (10 mL) at rt under Ar. The resulting solution was stirred at rt for 16 h. Then, the solvent was evaporated under reduced pressure to give the crude amine•TFA salt. Et_3N (1.16 mL, 8.29 mmol, 10.0 eq.) was then added dropwise to a stirred solution of the crude amine•TFA salt in CH_2Cl_2 (10 mL) at rt under Ar. The resulting solution was stirred at rt for 10 min. Then, AcCl (0.18 mL, 2.49 mmol, 3.0 eq.) was added dropwise and the resulting solution was stirred at rt for 16 h. Then, the solvent was evaporated under reduced pressure to give the crude product. Purification by flash column chromatography on silica with EtOAc and then 80:20 EtOAc-MeOH as eluent gave pyrrolidine **8m** (101 mg, 49%) as a colourless oil, R_F (EtOAc) 0.10; IR (ATR) 1731 (C=O, CO_2Me), 1639 (C=O, NC(O)Me), 1420, 1209 cm^{-1}; ^{1}H NMR (400 MHz, $CDCl_3$) (50:50 mixture of rotamers) δ 7.33-7.24 (m, 3H, Ph), 7.17-7.12 (m, 2H, Ph), 3.99-3.86 (m, 3H, NCH or C*H*Ph), 3.80-3.71 (m, 2H, NCH or C*H*Ph), 3.50 (ddd, J = 7.5, 7.5, 7.5 Hz, 0.5 H, C*H*CO_2Me), 3.44-3.39 (m, 3.5H, C*H*CO_2Me, CO_2Me), 2.14 (s, 1.5H, NC(O)Me), 2.13 (s, 1.5H, NC(O)Me); ^{13}C NMR (100.6 MHz, $CDCl_3$) (rotamers) 171.9 (C=O, NC(O)Me), 171.2 (C=O, NC(O)Me), 169.5 (C=O, CO_2Me), 169.4 (C=O, CO_2Me), 138.2 (*ipso*-Ph), 137.6 (*ipso*-Ph), 128.8 (Ph), 128.7 (Ph), 127.8 (Ph), 127.6 (Ph), 127.5 (Ph), 127.39 (Ph), 51.8 (OMe), 51.7 (OMe), 51.4 (NCH_2), 50.3 (NCH_2), 49.2 (*C*HCO_2Me), 48.0 (NCH_2), 47.6 (*C*HCO_2Me), 47.4 (NCH_2), 46.8 (*C*HPh), 45.0 (*C*HPh), 22.6 (NC(O)*Me*), 22.50 (NC(O)*Me*); MS (ESI) *m/z* 248 [$(M + H)^+$, 14], 270 [$(M + Na)^+$, 100]; HRMS (ESI) *m/z* calcd for $C_{14}H_{17}NO_3$ $(M + Na)^+$ 270.1101, found 270.1103 (–1.1 ppm error).

Lab Book Reference: TD 3/72

Methyl 1-acetyl-3-(4-methylphenyl)pyrrolidine-3-carboxylate 8n

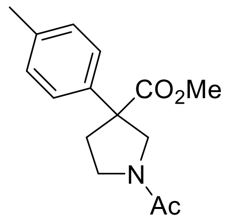

8n

Et_3N (0.32 mL, 2.3 mmol, 10.0 eq.) was added dropwise to a stirred solution of aryl pyrrolidine-3-methyl ester **8f**·HCl (59 mg, 0.23 mmol, 1.0 eq.) in CH_2Cl_2 (5 mL) at rt under Ar. The resulting solution was stirred at rt for 30 min. Then, AcCl (0.02 mL, 0.34 mmol, 1.5 eq.) was added dropwise and the solution was stirred at rt for 16 h. The solution was poured into water (10 mL) and the two layers were separated. The aqueous layer was extracted with CH_2Cl_2 (3 × 10 mL) and the combined organics were dried ($MgSO_4$) and evaporated under reduced pressure to give the crude product as an orange oil. Purification by flash column chromatography on silica with 70:29:1-50:49:1 hexane-EtOAc-$NH_4OH_{(aq)}$ as eluent gave *N*-acyl aryl pyrrolidine-3-methyl ester **8n** (60 mg, 99%) as a yellow solid, mp 64-66 °C, R_F (70:29:1 hexane-EtOAc- $NH_4OH_{(aq)}$) 0.07; IR (ATR) 2957, 1723 (C=O, CO_2Me), 1634 (C=O, MeCO), 1416, 1193, 1115 cm^{-1}; ^1H NMR (400 MHz, $CDCl_3$) (50:50 mixture of rotamers) δ 7.22-7.14 (m, 4H, Ar), 4.51 (d, J = 11.5 Hz, 0.5H, NCH), 4.48 (d, J = 11.5 Hz, 0.5H, NCH), 3.70 (d, J = 11.5 Hz, 0.5H, NCH), 3.65 (s, 1.5H, OMe), 3.68-3.62 (m, 0.5H, NCH), 3.62 (s, 1.5H, OMe), 3.58-3.52 (m, 1.5H, OMe), 3.43 (ddd, J = 12.5, 10.0, 7.0 Hz, 0.5H, NCH), 3.01-2.91 (m, 0.5H, CH), 2.91-2.88 (m, 0.5H, CH), 2.34 (s, 1.5H, C_6H_4*Me*), 2.33 (s, 1.5H, C_6H_4*Me*), 2.27- 2.12 (m, 1H, CH), 2.12 (s, 1.5H, MeCO), 2.04 (s, 1.5H, MeCO); ^{13}C NMR (100.6 MHz, $CDCl_3$) (rotamers) δ 174.1 (C=O, CO_2Me), 173.8 (C=O, CO_2Me), 169.5 (C=O, *C*OMe), 169.4 (C=O, *C*OMe), 137.9 (*ipso*-Ar), 137.7 (*ipso*-Ar), 136.4 (*ipso*-Ar), 136.0 (*ipso*-Ar), 129.7 (Ar), 129.6 (Ar), 126.5 (Ar), 126.3 (Ar), 57.5 (C), 55.7 (NCH_2), 55.3 (OMe), 53.2 (NCH_2), 53.0 (OMe), 46.1 (NCH_2), 44.4 (NCH_2), 35.1 (CH_2), 33.2 (CH_2), 22.6 (CO*Me*), 22.2 (CO*Me*), 21.2 (C_6H_4*Me*); MS (ESI) *m/z* 284 $(M + Na)^+$; HRMS *m/z* calcd for $C_{15}H_{19}NO_3$ $(M + Na)^+$ 284.1257, found 284.1253 (+1.8 ppm error).

Lab Book Reference: PJ-07-95.

1-[(3*R,4*R**)-3-(hydroxymethyl)-4-phenylpyrrolidin-1-yl]ethan-1-one 8o**

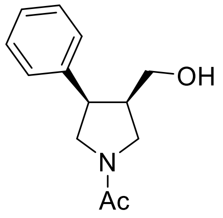

8o

$LiBH_4$ (1.21 mL of a 4 M solution in THF, 4.85 mmol, 4.0 eq.) was added dropwise to a stirred solution of ester **8m** (300 mg, 1.21 mmol, 1.0 eq.) in THF (5 mL) at 0 °C under Ar. The solution was allowed to warm to rt and then stirred at rt for 16 h. Then, H_2O (0.3 mL) and 2 M $NaOH_{(aq)}$ (0.6 mL) were added sequentially and the mixture was evaporated under reduced pressure. The residue was dissolved in EtOAc (10 mL) and then washed with H_2O (2 × 10 mL), dried (Na_2SO_4) and evaporated under reduced pressure to give the crude product. Purification by flash column chromatography on silica with 50:50 hexane-EtOAc as eluent gave alcohol **8o** (97 mg, 37%) as an orange oil, R_F (50:50 hexane-EtOAc) 0.16; IR (ATR) 3371 (OH), 2936, 1621 (C=O), 1456, 1423 cm^{-1}; ^{1}H NMR (400 MHz, $CDCl_3$) (50:50 mixture of rotamers) δ 7.35-7.27 (m, 3H, Ph), 7.25-7.14 (m, 2H, Ph), 3.90-3.85 (m, 1H, NCH), 3.81-3.63 (m, 2H, NCH), 3.59-3.49 (m, 1.5H, C*H*Ph, NCH), 3.45-3.34 (m, 1H, NCH, C*H*HO), 3.33-3.28 (m, 1H, HOC*H*), 3.26-3.23 (m, 0.5H, HOC*H*), 2.77 (ddddd, *J* = 7.0, 7.0, 7.0, 7.0, 7.0 Hz, 0.5H, C*H*CH_2OH), 2.70 (ddddd, *J* = 7.0, 7.0, 7.0, 7.0, 7.0 Hz, 0.5H, C*H*CH_2OH), 2.13 (s, 1.5 H, NC(O)Me), 2.12 (s, 1.5 H, NC(O)Me); ^{13}C NMR (100.6 MHz, $CDCl_3$) (rotamers) δ 169.8 (C=O), 169.7 (C=O), 139.1 (*ipso*-Ph), 139.0 (*ipso*-Ph), 129.0 (Ph), 128.9 (Ph), 127.8 (Ph), 127.7 (Ph), 127.3 (Ph), 127.2 (Ph), 61.7 ($HOCH_2$), 61.5 ($HOCH_2$), 52.9 (NCH_2), 50.3 (NCH_2), 49.3 (NCH_2), 47.5 (NCH_2), 45.8 (*C*HCH_2OH), 45.5 (*C*HCH_2OH), 44.2 (*C*HPh), 44.0 (*C*HPh), 22.6 (NC(O)*Me*); MS (ESI) *m/z* 220 [(M + H)$^+$, 15], 242 [(M + Na)$^+$, 100], 258 [(M + K)$^+$, 9]; HRMS (ESI) *m/z* calcd for $C_{13}H_{17}NO_2$ (M + Na)$^+$ 242.1151, found 242.1152 (–0.2 ppm error).

Lab Book Reference: TD 4/40

Methyl 1-acetyl-3-(4-methylphenyl)piperidine-3-carboxylate S10

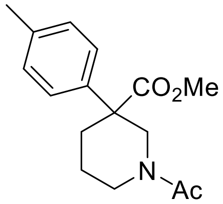

S10

Et_3N (0.4 mL, 2.89 mmol, 10.0 eq.) was added dropwise to a stirred solution of aryl piperidine-3-methyl ester **8d**·HCl (78 mg, 0.28 mmol, 1.0 eq.) in CH_2Cl_2 (5 mL) at rt under Ar. The resulting solution was stirred at rt for 30 min. Then, AcCl (0.03 mL, 0.43 mmol, 1.5 eq.) was added dropwise and the solution was stirred at rt for 16 h. The solution was poured into water (10 mL) and the two layers were separated. The aqueous layer was extracted with CH_2Cl_2 (3 × 10 mL) and the combined organics were dried ($MgSO_4$) and evaporated under reduced pressure to give the crude product as an orange oil. Purification by flash column chromatography on silica with 50:50-70:30 EtOAc-hexane as eluent gave *N*-acyl aryl piperidine-3-methyl ester **S10** (68 mg, 88%) as a yellow oil, R_F (70:30 EtOAc-hexane) 0.1; IR (ATR) 2949, 1726 (C=O, CO_2Me), 1631 (C=O, MeCO), 1430, 1271, 1233, 1142 cm^{-1}; ^1H NMR (400 MHz, $CDCl_3$) (70:30 mixture of rotamers) δ 7.32-7.30 (m, 0.6H, Ar), 7.25-7.23 (m, 1.4H, Ar), 7.18-7.13 (m, 2H, Ar), 4.64-4.59 (m, 0.7H, NCH), 4.59-4.51 (m, 1H, NCH), 3.71 (d, J = 13.5 Hz, 0.3H, NCH), 3.67 (s, 2.1H, OMe), 3.61 (s, 0.9H, OMe), 3.53-3.50 (m, 0.3H, NCH), 3.23 (ddd, J = 13.0, 8.5, 4.0 Hz, 0.3H, NCH), 3.09 (d, J = 13.5 Hz, 0.7H, NCH), 2.72-2.68 (m, 0.7H, CH), 2.54 (ddd, J = 13.0, 13.0, 3.0 Hz, 0.7H, NCH), 2.33 (s, 2.1H, C_6H_4*Me*), 2.31 (s, 0.9H, C_6H_4*Me*), 2.27 (s, 2.1H, MeCO), 2.16-2.06 (m, 0.3H, CH), 2.07 (s, MeCO), 1.90-1.76 (m, 1.4H, CH), 1.72-1.65 (m, 0.6H, CH), 1.57-1.46 (m, 1H, CH); ^{13}C NMR (100.6 MHz, $CDCl_3$) (rotamers) δ 174.2 (C=O, CO_2Me), 173.8 (C=O, CO_2Me), 170.1 (C=O, Me*C*O), 169.4 (C=O, Me*C*O), 137.8 (*ipso*-Ar), 137.2 (*ipso*-Ar), 136.8 (*ipso*-Ar), 129.7 (Ar), 129.5 (Ar), 126.3 (Ar), 125.6 (Ar), 54.0 (NCH_2), 52.5 (OMe), 52.4 (OMe), 51.1 (C), 49.9 (C), 47.4 (NCH_2), 46.6 (NCH_2), 41.4 (NCH_2), 33.1 (CH_2), 32.6 (CH_2), 23.1 (CH_2), 22.8 (CH_2), 22.0 (*Me*CO), 21.5 (*Me*CO), 21.1 (C_6H_4*Me*); MS (ESI) *m/z* 298 $(M + Na)^+$; HRMS *m/z* calcd for $C_{16}H_{21}NO_3$ $(M + Na)^+$ 298.1414, found 298.1408 (+1.6 ppm error).

Lab Book Reference: PJ-07-97.

1-Acetyl-3-(4-methylphenyl)piperidine-3-carboxylic acid 8p

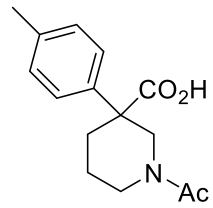

8p

KOH (139 mg, 2.47 mmol, 10.0 eq.) was added to a stirred solution of *N*-acyl aryl piperidine-3-methyl ester **S10** (68 mg, 0.24 mmol, 1.0 eq.) in EtOH (10 mL) at rt. The resulting mixture was stirred and heated at 100 °C for 16 h. The reaction mixture was allowed to cool to rt and evaporated under reduced pressure to give the crude product as an orange solid. The solid was taken up into water (10 mL) and washed with CH_2Cl_2 (3 × 10 mL). The aqueous layer was acidified with 1 M $HCl_{(aq)}$ (1 mL) and extracted with CH_2Cl_2 (3 × 10 mL). The combined organics were washed with brine (20 mL), dried (Na_2SO_4) and evaporated under reduced pressure to give *N*-acyl aryl piperidine-3-carboxylic acid **8p** (63 mg, 100%) as a white solid, mp 58-60 °C; IR (ATR) 2933, 2863, 1714 (C=O, CO_2H), 1591 (C=O, Me*C*O), 726 cm^{-1}; ^{1}H NMR (400 MHz, $CDCl_3$) (70:30 mixture of rotamers) δ 7.33 (d, *J* = 8.0 Hz, 0.6H, Ar), 7.30 (d, *J* = 8.0 Hz, 1.4H, Ar), 7.16 (d, *J* = 8.0 Hz, 1.4H, Ar), 7.13 (d, *J* = 8.0 Hz, 0.6H, Ar), 4.72 (d, *J* = 13.5 Hz, 0.3H, NCH), 4.66-4.62 (m, 0.7H, NCH), 4.54 (dd, *J* = 13.5, 2.0 Hz, 0.7H, NCH), 3.58 (ddd, *J* = 13.0, 4.5, 4.5 Hz, 0.3H, NCH), 3.45 (d, *J* = 13.5 Hz, 0.3H, NCH), 3.17 (ddd, *J* = 13.0, 9.0, 4.0 Hz,0.3H, NCH), 3.07 (d, *J* = 13.5 Hz, 0.7H, NCH), 2.75-2.70 (m, 0.7H, CH), 2.56-2.49 (m, 1.0H, NCH and CH), 2.33 (s, 2.1H, C_6H_4*Me*), 2.31 (s, 0.9H, C_6H_4*Me*), 2.27 (s, 2.1H, MeCO), 2.08 (s, 0.9H, MeCO), 1.99 (ddd, *J* = 13.5, 10.0, 4.5 Hz, 0.3H, CH), 1.86-1.76 (m, 1.7H, CH), 1.71-1.63 (m, 1H, CH); ^{13}C NMR (100.6 MHz, $CDCl_3$) (rotamers) δ 176.9 (C=O, CO_2H), 176.6 (C=O, CO_2H), 171.3 (C=O, Me*C*O), 170.3 (C=O, Me*C*O), 137.8 (*ipso*-Ar), 137.4 (*ipso*-Ar), 137.1 (*ipso*-Ar), 136.6 (*ipso*-Ar), 129.7 (Ar), 129.6 (Ar), 126.3 (Ar), 125.8 (Ar), 53.8 (NCH_2), 50.8 (C), 47.8 (NCH_2), 46.7 (NCH_2), 41.7 (NCH_2), 32.7 (CH_2), 23.0 (CH_2), 21.8 (*Me*CO), 21.4 (*Me*CO), 21.1 (C_6H_4*Me*); MS (ESI) *m/z* 284 $(M + Na)^+$; HRMS *m/z* calcd for $C_{15}H_{19}NO_3$ $(M + Na)^+$ 284.1257, found 284.1255 (+1.2 ppm error).

Lab Book Reference: PJ-07-100.

(1*R,2*S**)-2-(Thiophen-3-yl)cyclopentane-1-carboxamide 8q**

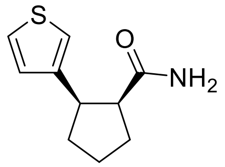

8q

Ammonia (0.04 mL of a 35% solution in H_2O, 0.765 mmol, 3.0 eq.) was added to a stirred solution of acid **7c** (50 mg, 0.255 mmol, 1.0 eq.), T3P® (0.243 mL of a 50% solution in EtOAc, 0.382 mmol, 1.5 eq.) and *i*Pr_2NEt (0.133 mL, 0.765 mmol, 3.0 eq.) in CH_2Cl_2 (3 mL) at rt under Ar. The resulting solution was stirred at rt for 18 h. Then, 2 M $HCl_{(aq)}$ (5 mL) was added and the mixture was extracted with CH_2Cl_2 (2 × 5 mL). The combined organic extracts were washed with $NaHCO_{3(aq)}$ (2 × 10 mL), dried (Na_2SO_4) and evaporated under reduced pressure to give amide **8q** (24 mg, 48%) as a white solid, mp 107-110 °C; IR (ATR) 3322 (NH), 3189 (NH), 2952, 1653 (C=O) cm^{-1}; ^{1}H NMR (400 MHz, $CDCl_3$) δ 7.23 (dd, J = 5.0, 3.0 Hz, 1H, Ar), 7.04-7.00 (m, 1H, Ar), 6.98 (dd, J = 5.0, 1.5 Hz, 1H, Ar), 5.17 (br s, 1H, NH), 4.97 (br s, 1H, NH), 3.42 (ddd, J = 8.0, 8.0, 8.0 Hz, 1H, C*H*Ar), 2.88 (ddd, J = 8.0, 8.0, 5.5 Hz, 1H, C*H*$CONH_2$), 2.17-1.89 (m, 4H, CH), 1.75-1.67 (m, 2H, CH); ^{13}C NMR (100.6 MHz, $CDCl_3$) δ 176.8 (C=O), 142.5 (*ipso*-Ar), 127.9 (Ar), 125.4 (Ar), 121.0 (Ar). 50.5 (*C*HCONH*2*), 45.0 (*C*HAr), 31.7 (CH_2), 28.3 (CH_2), 24.1 (CH_2); MS (ESI) *m/z* 196 [(M + H)$^+$, 5], 218 [(M + Na)$^+$, 100]; HRMS (ESI) *m/z* calcd for $C_{10}H_{13}ONS$ (M + Na)$^+$ 218.0610, found 218.0608 (+0.8 ppm error).

Lab Book Reference: TD 4/100

(3*R,4*R**)-4-(3-Methoxyphenyl)oxane-3-carboxamide 8r**

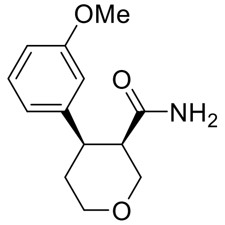

8r

Ammonia (0.06 mL of a 35% solution in H_2O, 1.27 mmol, 3.0 eq.) was added to a stirred solution of acid **7k** (100 mg, 0.42 mmol, 1.0 eq.), T3P® (0.404 mL of a 50% solution in EtOAc, 0.635 mmol, 1.5 eq.) and *i*Pr_2NEt (0.221 mL, 1.27 mmol, 3.0 eq.) in CH_2Cl_2 (5 mL) at rt under Ar. The resulting solution was stirred at rt for 18 h. Then, 2 M $HCl_{(aq)}$ (5 mL) was added and the mixture was extracted with CH_2Cl_2 (2

× 5 mL). The combined organic extracts were washed with $NaHCO_{3(aq)}$ (2 × 10 mL), dried (Na_2SO_4) and evaporated under reduced pressure to give amide **8r** (40 mg, 41%) as a white solid, mp 71-73 °C; IR (ATR) 2980 (NH), 1736 (C=O), 1180 cm^{-1}; ^1H NMR (400 MHz, $CDCl_3$) δ 7.25-7.22 (m, 1H, Ar), 6.85-6.83 (m, 1H, Ar), 6.78-6.75 (m, 2H, Ar), 6.56 (br s, 1H, NH), 5.66 (br s, 1H, NH), 4.25 (br d, J = 12.0 Hz, 1H, OCH), 4.21 (br dd, J = 12.0, 4.5 Hz, 1H, OCH), 3.79 (s, 3H, OMe), 3.68 (dd, J = 12.0, 3.0 Hz, OCH), 3.58 (ddd, J = 12.0, 12.0, 2.5 Hz, 1H, OCH), 3.14-3.07 (m, 1H, C*H*Ar), 2.66 (br s, 1H, C*H*CONH$_2$), 2.42 (dddd, 1H, J = 13.0, 12.0, 12.0, 4.5 Hz, 1H, OCH$_2$C*H*), 1.76-1.72 (m, 1H, OCH$_2$C*H*); ^{13}C NMR (100.6 MHz, $CDCl_3$) δ 174.4 (C=O), 159.7 (*ipso*-Ar), 143.6 (*ipso*-Ar), 129.6 (Ar), 119.7 (Ar), 113.6 (Ar), 111.9 (Ar), 69.5 (O*C*H$_2$CH), 68.6 (O*C*H$_2$CH$_2$), 55.2 (OMe), 48.3 (*C*HCONH$_2$), 42.6 (*C*HAr), 27.1 (OCH$_2$*C*H$_2$); MS (ESI) *m/z* 236 [(M + H)$^+$, 25], 258 [(M + Na)$^+$, 100]; HRMS (ESI) *m/z* calcd for $C_{13}H_{17}O_3N$ (M + Na)$^+$ 258.1101, found 258.1096 (+1.9 ppm error).

Lab Book Reference: TD 5/28

[(1*R,2*S**)-2-(Pyridin-3-yl)cyclopentyl]methanol 8s**

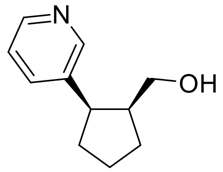

8s

Using general procedure H, $LiAlH_4$ (37 mg, 0.97 mmol, 2.0 eq.) in THF (2 mL), ester **2a** (100 mg, 0.487 mmol, 1.0 eq.) in THF (3 mL) gave alcohol **8s** (72 mg, 83%) as a colourless oil, IR (ATR) 3269 (OH), 2950, 2871, 1425 cm^{-1}; ^1H NMR (400 MHz, $CDCl_3$) δ 8.44 (br s, 1H, Ar), 8.38 (br s, 1H, Ar), 7.50 (d, J = 8.0 Hz, 1H, Ar), 7.22-7.19 (m, 1H, Ar), 3.28 (ddd, J = 7.5, 7.5, 7.5 Hz, 1H, C*H*Ar), 3.22 (dd, J = 10.5, 7.5 Hz, 1H, HOC*H*), 3.17 (dd, J = 10.5, 7.5 Hz, 1H, HOC*H*), 2.39 (ddddd, J = 7.5, 7.5, 7.5, 7.5, 7.5 Hz, 1H, C*H*CH$_2$OH), 2.16 (br s, 1H, OH), 2.11-2.04 (m, 1H, CH), 1.98-1.81 (m, 3H, CH), 1.78-1.72 (m, 1H, CH), 1.61-1.51 (m, 1H, CH); ^{13}C NMR (100.6 MHz, $CDCl_3$) δ 149.9 (Ar), 147.3 (Ar), 138.4 (*ipso*-Ar), 135.9 (Ar), 123.3 (Ar), 63.6 (HOCH$_2$), 46.4 (*C*HCH$_2$OH), 44.8 (*C*HAr), 31.0 (CH$_2$), 28.3 (CH$_2$), 24.0 (CH$_2$); MS (ESI) *m/z* 178 [(M + H)$^+$, 100]; HRMS (ESI) *m/z* calcd for $C_{11}H_{15}NO$ (M + H)$^+$ 178.1226, found 178.1226 (+0.4 ppm error).

Lab Book Reference: TD 5/9

[(1*R,2*S**)-2-(1-methyl-1H-pyrazol-4-yl)cyclopentyl]methanol 8t**

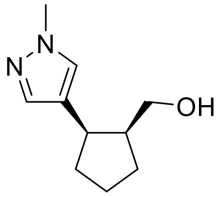

8t

Using general procedure H, $LiAlH_4$ (219 mg, 5.76 mmol, 2.0 eq.) in THF (5 mL), ester **2b** (600 mg, 2.88 mmol, 1.0 eq.) in THF (10 mL) gave alcohol **8t** (503 mg, 98%) as a colourless oil, IR (ATR) 3342 (br, OH), 2946, 2871, 1401 cm^{-1}; ^{1}H NMR (400 MHz, $CDCl_3$) δ 7.31 (s, 1H, Ar), 7.15 (s, 1H, Ar), 3.85 (s, 3H, NMe), 3.40-3.31 (m, 2H, HOC*H*), 3.20 (ddd, *J* = 7.0, 7.0, 7.0 Hz, C*H*Ar), 2.26 (ddddd, *J* = 7.0, 7.0, 7.0, 7.0, 7.0 Hz, 1H, C*H*CH_2OH), 2.04-1.95 (m, 1H, CH), 1.87-1.78 (m, 2H, CH), 1.75-1.64 (m, 2H, CH), 1.46-1.37 (m, 1H, CH), 1.32 (br s, 1H, OH); ^{13}C NMR (100.6 MHz, $CDCl_3$) δ 138.8 (Ar), 128.5 (Ar), 122.5 (*ipso*-Ar), 64.3 ($HOCH_2$), 46.0 (*C*HCH_2OH), 39.0 (NMe), 37.2 (*C*HAr), 32.6 (*C*H_2CH), 27.8 (*C*H_2CH), 23.6 (CH_2*C*H_2CH_2); MS (ESI) *m/z* 181 [$(M + H)^+$, 100], 203 [$(M + Na)^+$, 18]; HRMS (ESI) *m/z* calcd for $C_{10}H_{16}N_2O$ $(M + H)^+$ 181.1335, found 181.134 (+0.7 ppm error).

Lab Book Reference: TD 4/61

[(3*R,4*S**)-4-(2-Fluorophenyl)oxolan-3-yl]methanol 8u**

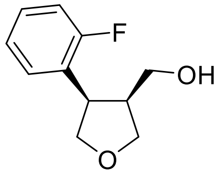

8u

Using general procedure H, $LiAlH_4$ (295 mg, 7.76 mmol, 2.0 eq.) in THF (10 mL), and a 60:40 mixture of ester **2g** and 2-fluoroboronic acid (1.42 g (i.e. 870 mg (3.88 mmol, 1.0 eq.) of ester **2g**) in THF (10 mL) gave alcohol **8u** (598 mg, 79%) as a colourless oil, R_F (50:50 hexane-EtOAc) 0.20; IR (ATR) 3393 (br, OH), 1491, 759 cm^{-1}; ^{1}H NMR (400 MHz, $CDCl_3$) δ 7.35-7.31 (m, 1H, Ar), 7.25-7.20 (m, 1H, Ar), 7.14-7.11 (m, 1H, Ar), 7.07-7.02 (m, 1H, Ar), 4.17-4.09 (m, 3H, OCH), 3.87-3.82 (m, 1H, C*H*Ar), 3.78 (dd, *J* = 8.5, 7.5 Hz, 1H, OCH), 3.41-3.36 (m, 1H, HOC*H*), 3.31-3.24 (m, 1H, HOC*H*), 2.87 (ddddd, *J* = 7.5, 7.5, 7.5, 7.5, 7.5 Hz, 1H, C*H*CH_2OH), 1.43 (br s, 1H, OH); ^{13}C NMR (100.6 MHz, $CDCl_3$) δ 160.9 (d, *J* = 244.0 Hz, CF), 129.0 (d, *J* = 4.0 Hz, Ar), 128.5, (d, *J* = 8.0 Hz, Ar), 126.6 (d, *J* = 14.5 Hz, *ipso*-Ar), 124.7 (d, *J* = 3.5 Hz, Ar), 115.3 (d, *J* = 23.0 Hz, Ar), 73.0 (OCH_2), 70.4 (OCH_2), 62.1 ($HOCH_2$),

45.6 (*C*HCH$_2$OH), 38.8 (*C*HAr); MS (ESI) *m/z* 219 [(M + Na)$^+$, 100]; HRMS (ESI) *m/z* calcd for $C_{11}H_{13}O_2F$ (M + Na)$^+$ 219.0792, found 219.0790 (+0.8 ppm error).

Lab Book Reference: TD 5/32

[1-Methyl-3-(4-methylphenyl)pyrrolidin-3-yl]methanol 8v

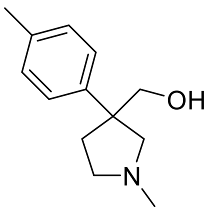

8v

Using general procedure H, $LiAlH_4$ (63 mg, 1.66 mmol, 5 eq.) in THF (5 mL) in THF (5 mL), ester **4c** (120 mg, 0.33 mmol, 1.0 eq.) in THF (5 mL) gave the crude product as a green oil. Purification by flash column chromatography on silica with 90:9:1 CH_2Cl_2-MeOH-$NH_4OH_{(aq)}$ as eluent gave *N*-methyl aryl pyrrolidine alcohol **8v** (20 mg, 31%) as a colourless oil, R_F (90:9:1 CH_2Cl_2-MeOH-$NH_4OH_{(aq)}$) 0.17, IR (ATR) 3347 (OH), 2921, 1515, 1449, 1049, 818 cm^{-1}; ^{1}H NMR (400 MHz, $CDCl_3$) δ 7.13 (d, J = 8.0 Hz, 2H, Ar), 7.07 (d, J = 8.0 Hz, 2H, Ar), 3.69 (d, J = 10.0 Hz, 1H, OCH), 3.51 (dd, J = 10.0, 2.0 Hz, 1H, OCH), 3.42 (d, J = 9.0 Hz, 1H, NCH), 3.27-3.20 (m, 1H, NCH), 2.64 (dd, J = 9.0, 2.0 Hz, 1H, NCH), 2.50-2.42 (m, 1H, CH), 2.40 (s, 3H, NMe), 2.32 (s, 3H, C_6H_4*Me*), 2.27-2.24 (m, 2H, CH and NCH); ^{13}C NMR (100.6 MHz, $CDCl_3$) δ 141.9 (*ipso*-Ar), 136.2 (*ipso*-Ar), 129.2 (Ar), 126.6 (Ar), 75.3 (OCH_2), 66.0 (NCH_2), 56.0 (NCH_2), 51.2 (C), 41.9 (NMe), 34.3 (CH_2), 21.0 (C_6H_4*Me*); MS (ESI) *m/z* 206 (M + H)$^+$; HRMS *m/z* calcd for $C_{13}H_{19}NO$ (M + H)$^+$ 206.1539, found 206.1537 (+1.0 ppm error).
Lab Book Reference: PJ-07-90.

[1-(Pyridin-2-yl)cyclopentyl]methanol 8w

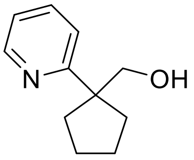

8w

Using general procedure H, $LiAlH_4$ (37 mg, 0.98 mmol, 2.0 eq.) in THF (2.5 mL), ester **4p** (100 mg, 0.49 mmol, 1.0 eq.) in THF (2.5 mL) gave alcohol **8w** (85 mg, 98%) as an orange oil, IR (ATR) 3350 (OH), 2949, 1590, 1471, 1048, 748 cm^{-1}; ^{1}H NMR (400 MHz, $CDCl_3$) δ 8.46-8.45 (m, 1H, 6-py), 7.67-7.63 (m, 1H, 4-py), 7.31-7.29 (m, 1H, 3-py), 7.14-7.11 (m, 5-py), 3.73 (s, 2H, C*H₂*OH), 1.99-1.94 (m, 2H, CH), 1.90-1.82 (m, 2H, CH), 1.80-1.76 (m, 4H, CH); ^{13}C NMR (100.6 MHz, $CDCl_3$) δ 169.0 (*ipso*-Ar), 147.8 (Ar), 137.0 (Ar), 121.2 (Ar), 121.0 (Ar), 69.2 (C), 53.6 (OCH_2), 36.5 (CH_2), 25.5 (CH_2); MS (ESI) *m/z* 178 $(M + H)^+$; HRMS *m/z* calcd for $C_{11}H_{15}NO$ $(M + H)^+$ 178.1226, found 178.1226 (+0.3 ppm error).

Lab Book Reference: PJ-07-23.

[4-(Pyridin-2-yl)oxan-4-yl]methanol 8x

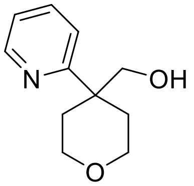

8x

Using general procedure H, $LiAlH_4$ (147 mg, 3.89 mmol, 2.0 eq.) in THF (8 mL), ester **4s** (430 mg, 1.95 mmol, 1.0 eq.) in THF (7 mL) gave the crude product as an orange oil. Purification by flash column chromatography on silica with EtOAc as eluent gave aryl THP alcohol **8x** (312 mg, 82%) as a colourless oil, R_F (EtOAc) 0.17; IR (ATR) 3378 (OH), 2952, 1432, 1043, 561 cm^{-1}; ^{1}H NMR (400 MHz, $CDCl_3$) δ 8.56-8.54 (m, 1H, 6-py), 7.74-7.70 (m, 1H, 4-py), 7.36-7.34 (m, 1H, 3-py), 7.25-7.18 (m, 1H, 5-py), 4.04 (br s, 1H, OH), 3.83-3.78 (m, 4H, OCH), 3.68-3.62 (m, 2H, OCH), 2.21-2.14 (m, 2H, CH), 1.91-1.85 (m, 2H, CH); ^{13}C NMR (100.6 MHz, $CDCl_3$) δ 165.5 (*ipso*-Ar), 148.8 (Ar), 137.2 (Ar), 121.8 (Ar), 120.9 (Ar), 68.5 (OCH_2), 64.3 (OCH_2), 42.6 (C), 32.5 (CH_2); MS (ESI) *m/z* 194 $(M + H)^+$; HRMS *m/z* calcd for $C_{11}H_{15}NO_2$ $(M + H)^+$ 194.1176, found 194.1176 (+0.2 ppm error).

Lab Book Reference: PJ-07-82.

3-[(1*R,2*S**)-2-(Fluoromethyl)cyclopentyl]pyridine 8y**

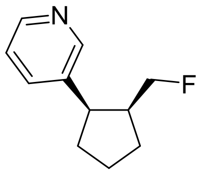

8y

PyFluor (68 mg, 0.31 mmol, 1.1 eq.) was added portionwise to a stirred solution of alcohol **8s** (68 mg, 0.282 mmol, 1.0 eq.) and DBU (0.115 mL, 0.564 mmol, 2.0 eq.) in toluene (1 mL) at rt under Ar. The resulting solution was stirred and heated at 50 °C for 40 h. The reaction mixture was allowed to cool to rt and evaporated under reduced pressure to give the crude product. Purification by flash column chromatography on silica with 50:50 hexane-EtOAc as eluent gave cyclopentane **8y** (18 mg, 36%) as a colourless oil, R_F (70:30 EtOAc-hexane) 0.46; IR (ATR) 2956, 2873, 1424 cm^{-1}; ^{1}H NMR (400 MHz, $CDCl_3$) δ 8.47-8.45 (m, 2H, Ar, Ar), 7.55-7.52 (m, 1H, Ar), 7.22 (dd, J = 8.0, 5.0 Hz, 1H, Ar), 4.06 (ddd, J = 47.5, 9.5, 6.0 Hz, 1H, CHF), 4.00 (ddd, J = 47.5, 9.5, 6.0 Hz, 1H, CHF), 3.30 (ddd, J = 8.0, 8.0, 8.0 Hz, 1H, C*H*Ar), 2.59-2.51 (m, 1H, C*H*CH_2F), 2.10-2.01 (m, 1H, CH), 2.00-1.91 (m, 2H, CH), 1.82-1.74 (m, 2H, CH), 1.67-1.61 (m, 1H, CH); ^{13}C NMR (100.6 MHz, $CDCl_3$) δ 150.1 (Ar), 147.8 (Ar), 137.4 (*ipso*-Ar), 135.8 (Ar), 123.3 (Ar), 84.7 (d, J = 167.5 Hz, CH_2F), 44.8 (d, J = 4.0 Hz, *C*HAr), 44.2 (d, J = 18.0 Hz, *C*HCH_2F), 31.0 (CH_2), 27.5 (d, J = 5.5 Hz, CH_2), 24.1 (CH_2); MS (ESI) *m/z* 180 [(M + H)$^+$, 100]; HRMS (ESI) *m/z* calcd for $C_{11}H_{14}NF$ (M + H)$^+$ 180.1183, found 180.1183 (+0.3 ppm error).

Lab Book Reference: TD 5/12

4-[(1*R,2*S**)-2-(Fluoromethyl)cyclopentyl]-1-methyl-1H-pyrazole 8z**

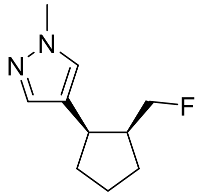

8z

PyFluor (249 mg, 1.54 mmol, 1.1 eq.) was added portionwise to a stirred solution of alcohol **8t** (253 mg, 1.40 mmol, 1.0 eq.) and DBU (0.418 mL, 2.80 mmol, 2.0 eq.) in toluene (2 mL) at rt under Ar. The resulting solution was stirred and heated at 70 °C for 64 h. The reaction mixture was allowed to cool to rt and evaporated under reduced pressure to give the crude product. Purification by flash column

chromatography on silica with 50:50 hexane-EtOAc as eluent gave cyclopentane **8z** (57 mg, 22%) as a colourless oil, R_F (50:50 hexane-EtOAc) 0.30; IR (ATR) 2953, 1444, 1399, 986 cm^{-1}; ^{1}H NMR (400 MHz, $CDCl_3$) δ 7.26 (s, 1H, Ar), 7.11 (s, 1H, Ar), 4.13 (ddd, J = 47.5, 9.0, 6.5 Hz, 1H, CHF), 4.09 (ddd, J = 47.5, 9.0, 7.0 Hz, 1H, CHF), 3.85 (s, 3H, NMe), 3.18 (ddd, J = 7.0, 7.0, 7.0 Hz, 1H, C*H*Ar), 2.41-2.32 (m, 1H, C*H*CH_2F), 2.02-1.97 (m, 1H, CH), 1.87-1.67 (m, 4H, CH), 1.51-1.46 (m, 1H, CH); ^{13}C NMR (100.6 MHz, $CDCl_3$) δ 138.7 (Ar), 128.6 (Ar), 121.7 (*ipso*-Ar), 85.3 (d, J = 165.5 Hz, CH_2F), 43.8 (d, J = 18.0 Hz, *C*HCH_2F), 38.9 (NMe), 37.1 (d, J = 4.5 Hz, *C*HAr), 32.6 (CH_2), 26.7 (d, J = 4.5 Hz, CH_2), 23.4 (CH_2); MS (ESI) *m/z* 183 [(M + H)$^+$, 100], 205 [(M + Na)$^+$, 7]; HRMS (ESI) *m/z* calcd for $C_{10}H_{15}N_2F$ (M + H)$^+$ 183.1292, found 183.1291 (+0.8 ppm error).

Lab Book Reference: TD 4/87

2-[1-(Fluoromethyl)cyclopentyl]pyridine 8aa

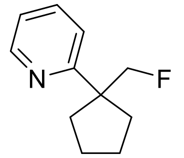

8aa

DBU (0.08 mL, 0.56 mmol, 2.0 eq.) was added dropwise to a stirred solution of aryl cyclopentane alcohol **8w** (50 mg, 0.28 mmol, 1.0 eq.) and PyFluor (50 mg, 0.31 mmol, 1.1 eq.) in toluene (5 mL) at rt under Ar. The resulting solution was stirred and heated at 80 °C for 48 h. The reaction mixture was allowed to cool to rt and evaporated under reduced pressure to give the crude product as a yellow solid. Purification by flash column chromatography on silica with 60:40 hexane-EtOAc as eluent gave aryl cyclopentane fluoride **8aa** (35 mg, 70%) as a yellow oil, R_F (60:40 hexane-EtOAc) 0.57; IR (ATR) 2954, 1588, 1470, 1431, 991, 747 cm^{-1}; ^{1}H NMR (400 MHz, $CDCl_3$) δ 8.59 (ddd, J = 5.0, 2.0, 1.0 Hz, 1H, 6-py), 7.62 (ddd, J = 8.0, 8.0, 2.0 Hz, 1H, 4-py), 7.35-7.33 (m, 1H, 3-py), 7.12 (ddd, J = 8.0, 5.0, 1.0 Hz, 1H, 5-py), 4.51 (d, J = 48.0 Hz, 2H, FCH_2), 2.18-2.10 (m, 2H, CH), 2.00-1.80 (m, 2H, CH), 1.78-1.71 (m, 4H, CH); ^{13}C NMR (100.6 MHz, $CDCl_3$) δ 165.0 (d, J = 2.0 Hz, *ipso*-Ar), 149.1 (Ar), 136.2 (Ar), 121.7 (Ar), 121.4. (Ar), 88.7 (d, J = 175.0 Hz, FCH_2), 54.4 (d, J = 16.5 Hz, C), 34.1 (d, J = 4.5 Hz, CH_2), 25.0 (CH_2); MS (ESI) *m/z* 179 (M + H)$^+$; HRMS *m/z* calcd for $C_{11}H_{14}FN$ (M + H)$^+$ 180.1183, found 180.1180 (+2.0 ppm error).

Lab Book Reference: PJ-07-34.

2-[4-(Fluoromethyl)oxan-4-yl]pyridine 8ab

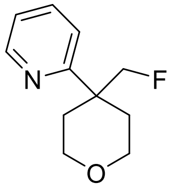

8ab

DBU (0.25 mL, 1.03 mmol, 2.0 eq.) was added dropwise to a stirred solution of aryl THP alcohol **8x** (100 mg, 0.52 mmol, 1.0 eq.) and PyFluor (92 mg, 0.57 mmol, 1.1 eq.) at rt under Ar. The resulting solution was stirred and heated at 80 °C for 48 h. The reaction mixture was allowed to cool to rt and evaporated under reduced pressure to give the crude product as an orange solid. Purification by flash column chromatography on silica with 60:40 hexane-EtOAc as eluent gave aryl THP fluoride **8ab** (86 mg, 85%) as a colourless oil, R_F (60:40 hexane-EtOAc) 0.35; IR (ATR) 2956, 1588, 1469, 1235, 1106, 729 cm^{-1}; ^1H NMR (400 MHz, $CDCl_3$) δ 8.64 (ddd, J = 5.0, 2.0, 1.0 Hz, 1H, 6-py), 7.69 (ddd, J = 7.5, 7.5, 2.0 Hz, 1H, 4-py), 7.37-7.35 (m, 1H, 3-py), 7.18 (ddd, J = 7.5, 5.0, 1.0 Hz, 1H, 5-py), 4.45 (d, J = 47.5 Hz, 2H, FCH_2), 3.84 (ddd, J = 12.0, 4.0, 4.0 Hz, 2H, OCH), 3.50 (ddd, J = 12.0, 10.0, 2.5 Hz, 2H, OCH), 2.39-2.33 (m, 2H, CH), 1.96 (ddd, J = 14.0, 10.0, 4.0 Hz, 2H, CH); ^{13}C NMR (100.6 MHz, $CDCl_3$) δ 161.2 (*ipso*-Ar), 149.6 (Ar), 136.4 (Ar), 122.4 (Ar), 121.8 (Ar), 90.3 (d, J = 176.5 Hz, FCH_2), 64.4 (OCH_2), 43.8 (d, J = 17.5 Hz, C), 31.2 (d, J = 5.0 Hz, CH_2); MS (ESI) *m/z* 196 $(M + H)^+$; HRMS *m/z* calcd for $C_{11}H_{14}FNO$ $(M + H)^+$ 196.1132, found 196.1127 (+3.1 ppm error).
Lab Book Reference: PJ-07-84.

[(3*R,4*R**)-4-(2-Fluorophenyl)oxolan-3-yl]methyl methanesulfonate S11**

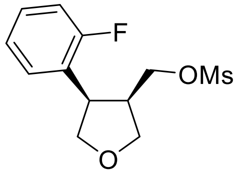

S11

Methanesulfonyl chloride (0.265 mL, 3.43 mmol, 1.2 eq.) was added dropwise to a stirred solution of alcohol **8u** (560 mg, 2.85 mmol, 1.0 eq.) and Et_3N (0.796 mL, 5.71 mmol, 2.0 eq.) in CH_2Cl_2 (10 mL) at rt. The resulting solution was stirred at rt for 16 h. Then, H_2O (10 mL) was added and the aqueous layer was extracted with CH_2Cl_2 (2 × 10 mL). The combined organic extracts were dried (Na_2SO_4) and evaporated under reduced pressure to give mesylate **S11** (492 mg, 63%) as a yellow oil, R_F (50:50 hexane-EtOAc) 0.30; IR (ATR) 1491, 1355 (S=O), 1174 (S=O) cm^{-1}; ^1H NMR (400 MHz, $CDCl_3$) δ 7.33-7.29

(m, 1H, Ar), 7.29-7.24 (m, 1H, Ar), 7.15 (dddd, *J* = 7.5, 7.5, 1.0, 1.0 Hz, 1H, Ar), 7.09-7.04 (m, 1H, Ar), 4.19-4.11 (m, 3H, OCH), 3.96 (dd, *J* = 10.0, 6.0 Hz, 1H, OCH), 3.91-3.88 (m, 1H, C*H*Ar), 3.87-3.78 (m, 2H, OCH), 3.09 (ddddd, *J* = 7.5, 7.5, 7.5, 7.5, 7.5 Hz, C*H*CH_2OS), 2.83 (s, 3H, SO_2Me); ^{13}C NMR (100.6 MHz, $CDCl_3$) δ 160.8 (d, *J* = 245.0 Hz, CF), 129.1-129.0 (m, Ar), 125.5 (d, *J* = 14.5 Hz, *ipso*-Ar), 124.9 (d, *J* = 3.5 Hz, Ar), 115.6 (d, *J* = 22.5 Hz, Ar), 72.9 (OCH_2), 70.2 (OCH_2), 68.7 (OCH_2), 42.3 (*C*HCH_2OS), 38.8 (*C*HAr), 37.2 (SO_2Me); MS (ESI) *m/z* 275 [(M + H)$^+$, 5], 297 [(M + Na)$^+$, 100], 313 [(M + K)$^+$, 6]; HRMS (ESI) *m/z* calcd for $C_{12}H_{15}O_4SF$ (M + Na)$^+$ 297.0567, found 297.0565 (+0.8 ppm error).

Lab Book Reference: TD 5/33

(3*R,4*S**)-3-(Azidomethyl)-4-(2-fluorophenyl)oxolane S12**

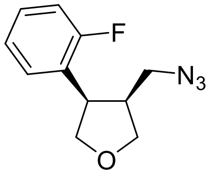

S12

Sodium azide (350 mg, 5.38 mmol, 3.0 eq.) was added portionwise to a stirred solution of mesylate **S11** (492 mg, 1.79 mmol, 1.0 eq.) in DMF (5 mL) at rt under Ar. The resulting mixture was stirred at rt for 10 min and then stirred and heated at 120 °C for 16 h. The solution was allowed to cool to rt. Then, H_2O (25 mL) and Et_2O (25 mL) were added and the two layers were separated. The aqueous layer was extracted with Et_2O (2 × 25 mL) and the combined organic extracts were washed with brine (25 mL), dried ($MgSO_4$) and evaporated under reduced pressure to give azide **S12** (336 mg, 89%) as a colourless oil, R_F (50:50 hexane-EtOAc) 0.47; IR (ATR) 2097 (N_3), 1490, 758 cm^{-1}; ^{1}H NMR (400 MHz, $CDCl_3$) δ 7.31-7.22 (m, 2H, Ar), 7.15 (ddd, *J* = 7.5, 7.5, 1.5 Hz, 1H, Ar), 7.09-7.04 (m, 1H, Ar), 4.18-4.10 (m, 3H, OCH), 3.87-3.82 (m, 1H, C*H*Ar), 3.75 (dd, *J* = 9.0, 6.5 Hz, 1H, OCH), 3.17 (br dd, *J* = 9.0, 6.5 Hz, 1H, CHN_3), 2.91-2.84 (m, 2H, CHN_3, C*H*CH_2N_3); ^{13}C NMR (100.6 MHz, $CDCl_3$) δ 160.8 (d, *J* = 245.0 Hz, CF), 128.9 (d, *J* = 4.0 Hz, Ar), 124.7 (d, *J* = 8.0 Hz, Ar), 125.9 (d, *J* = 14.5 Hz, *ipso*-Ar), 124.7 (d, *J* = 3.5 Hz, Ar), 115.4 (d, *J* = 22.5 Hz, Ar), 72.7 (OCH_2), 71.0 (OCH_2), 51.3 (CH_2N_3), 42.7 (*C*HCH_2N_3), 39.3 (d, *J* = 3.0 Hz, *C*HAr); MS (ESI) *m/z* 244 [(M + Na)$^+$, 100]; HRMS (ESI) *m/z* calcd for $C_{11}H_{12}ON_3F$ (M + Na)$^+$ 244.0857, found 244.0856 (+0.2 ppm error).

Lab Book Reference: TD 5/34

1-[(3*R,4*S**)-4-(2-Fluorophenyl)oxolan-3-yl]methanamine 8ac**

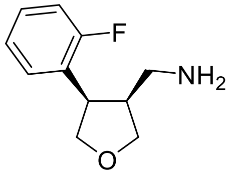

8ac

Triphenylphosphine (832 mg, 3.17 mmol, 2.0 eq.) was added portionwise to a stirred solution of azide **S12** (335 mg, 1.59 mmol, 1.0 eq.) in 5:1 THF-H_2O (10 mL) at rt under Ar. The solution was stirred and heated at 65 °C for 16 h. Then, the mixture was allowed to cool to rt and the solvent was evaporated under reduced pressure to give the crude product. Purification by flash column chromatography on silica with 50:50 hexane-EtOAc and then 80:20 EtOAc-MeOH as eluent gave amine **8ac** (270 mg, 87%) as a yellow oil, R_F (90:10 EtOAc-MeOH) 0.17; IR (ATR) 2937 (NH), 2868 (NH), 1490, 759 cm^{-1}; ^1H NMR (400 MHz, $CDCl_3$) δ 7.32 (ddd, J = 7.5, 7.5, 2.0 Hz, 1H, Ar), 7.23 (dddd, J = 8.0, 7.5, 5.5, 2.0 Hz, 1H, Ar), 7.13 (ddd, J = 7.5, 7.5, 1.5 Hz, 1H, Ar), 7.04 (ddd, J = 10.5, 8.0, 1.5 Hz, 1H, Ar), 4.18-4.08 (m, 3H, OCH), 3.85-3.81 (m, 1H, C*H*Ar), 3.68 (dd, J = 8.5, 8.5 Hz, 1H, OCH), 2.72 (ddddd, J = 8.0, 8.0, 8.0, 8.0, 8.0 Hz, 1H, C*H*CH_2NH_2), 2.49 (dd, J = 12.5, 8.0 Hz, 1H, C*H*NH_2), 2.35 (dd, J = 12.5, 8.0 Hz, 1H, C*H*NH_2), 2.12 (br s, 2H, NH_2); ^{13}C NMR (100.6 MHz, $CDCl_3$) δ 160.8 (d, J = 244 Hz, CF) , 129.3 (d, J = 4.0 Hz, Ar), 128.4 (d, J = 8.5 Hz, Ar), 127.0 (d, J = 14.5 Hz, *ipso*-Ar), 124.7 (d, J = 3.5 Hz, Ar), 115.2 (d, J = 23.0 Hz, Ar), 73.4 (OCH_2), 70.9 (OCH_2), 46.7 (*C*HCH_2NH_2), 41.1 (CH_2NH_2), 39.1 (d, J = 3.0 Hz, *C*HAr); MS (ESI) *m/z* 196 [(M + H)$^+$, 100], 218 [(M + Na)$^+$, 5]; HRMS (ESI) *m/z* calcd for $C_{11}H_{14}ONF$ (M + H)$^+$ 196.1132, found 196.1128 (+2.0 ppm error).

Lab Book Reference: TD 5/37

[1-(Pyridin-2-yl)cyclopentyl]methyl methanesulfonate S13

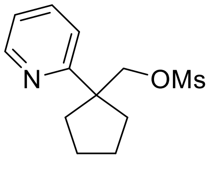

S13

MsCl (0.026 mL, 0.34 mmol, 1.2 eq.) was added dropwise to a stirred solution of aryl cyclopentane alcohol **8w** (50 mg, 0.28 mmol, 1.0 eq.) and Et_3N (0.08 mL, 0.56 mmol, 2.0 eq.) in CH_2Cl_2 (5 mL) at rt under Ar. The resulting solution was stirred at rt for 16 h. The reaction mixture was evaporated under reduced pressure to give the crude product as a yellow oil. Purification by flash column chromatography

on silica with 70:30 hexane-EtOAc as eluent gave aryl cyclopentane mesylate **S13** (66 mg, 91%) as a colourless oil, R_F (70:30 hexane-EtOAc) 0.09; IR (ATR) 2960, 1350, 1171, 941, 748 cm^{-1}; ^{1}H NMR (400 MHz, $CDCl_3$) δ 8.56 (ddd, J = 5.0, 2.0, 1.0 Hz, 1H, 6-py), 7.63 (ddd, J = 7.5, 7.5, 2.0 Hz, 1H, 4-py), 7.34-7.32 (m, 1H, 3-py), 7.13 (ddd, J = 7.5, 5.0, 1.0 Hz, 1H, 5-py), 4.43 (s, 2H, CH_2O), 2.71 (s, 3H, SO_2Me), 2.15-2.10 (m, 2H, CH), 2.00-1.94 (m, 2H, CH), 1.79-1.71 (m, 4H, CH); ^{13}C NMR (100.6 MHz, $CDCl_3$) δ 164.1 (*ipso*-Ar), 149.1 (Ar), 136.5 (Ar), 121.7 (Ar), 121.5 (Ar), 76.1 (CH_2O), 53.5 (C), 36.8 (Me), 34.8 (CH_2), 24.6 (CH_2); MS (ESI) *m/z* 256 (M + H)$^+$; HRMS *m/z* calcd for $C_{12}H_{17}NO_3S$ (M + H)$^+$ 256.1002, found 256.0998 (+1.7 ppm error).
Lab Book Reference: PJ-07-28.

2-[1-(Azidomethyl)cyclopentyl]pyridine S14

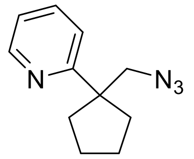

S14

A solution of aryl cyclopentane mesylate **S13** (100 mg, 0.39 mmol, 1.0 eq.) in DMF (1 mL) was added dropwise to a stirred solution of NaN_3 (76 mg, 1.18 mmol, 3.0 eq.) in DMF (1 mL) at rt under Ar. The resulting mixture was stirred and heated at 60 °C for 24 h and then at 120 °C for 24 h. The reaction mixture was allowed to cool to rt and poured into water (10 mL) and Et_2O (10 mL). The two layers were separated, and the aqueous layer was extracted with Et_2O (3 × 5 mL). The combined organics were dried ($MgSO_4$) and evaporated under reduced pressure to give a colourless oil. Purification by flash column chromatography on silica with 90:10 hexane-EtOAc as eluent gave aryl cyclopentane azide **S14** (68 mg, 86%) as a colourless oil, R_F (70:30 hexane-EtOAc) 0.42; IR (ATR) 2956, 2093 (N_3), 1587, 1469, 747 cm^{-1}; ^{1}H NMR (400 MHz, $CDCl_3$) δ 8.59-8.58 (m, 1H, 6-py), 7.64 (ddd, J = 8.0, 8.0, 2.0 Hz, 1H, 4-py), 7.33-7.31 (m, 1H, 3-py), 7.13 (ddd, J = 8.0, 5.0, 1.0 Hz, 1H, 5-py), 3.60 (s, 2H, N_3CH), 2.16-2.10 (m, 2H, CH), 1.95-1.89 (m, 2H, CH), 1.78-1.69 (m, 4H, CH); ^{13}C NMR (100.6 MHz, $CDCl_3$) δ 165.2 (*ipso*-Ar), 149.2 (Ar), 136.3 (Ar), 121.5 (Ar), 121.3 (Ar), 60.4 (N_3CH_2), 54.7 (C), 35.6 (CH_2), 24.5 (CH_2); MS (ESI) *m/z* 203 (M + H)$^+$; HRMS *m/z* calcd for $C_{11}H_{14}N_4$ (M + H)$^+$ 203.1291, found 203.1292 (–2.1 ppm error).
Lab Book Reference: PJ-07-74.

1-[1-(Pyridin-2-yl)cyclopentyl]methanamine 8ad

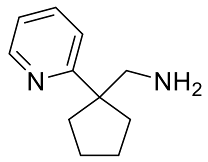

8ad

PPh_3 (166 mg, 0.63 mmol, 2.0 eq.) was added to a stirred solution of aryl cyclopentane azide **S14** (64 mg, 0.32 mmol, 1.0 eq.) in 5:1 THF-water (6 mL) at rt. The resulting mixture was stirred and heated at 65 °C for 16 h. The reaction mixture was allowed to cool to rt and evaporated under reduced pressure to give the crude product as a colourless oil. Purification by flash column chromatography on silica with 85:14:1 CH_2Cl_2-MeOH-$NH_4OH_{(aq)}$ as eluent gave aryl cyclopentane amine **8ad** (53 mg, 95%) as a green oil, R_F (85:14:1 CH_2Cl_2-MeOH-$NH_4OH_{(aq)}$) 0.43; IR (ATR) 3066 (NH), 1474, 1430, 740, 690, 489 cm^{-1}; ^1H NMR (400 MHz, $CDCl_3$) δ 8.54 (m, 1H, 6-py), 7.65-7.61 (m, 1H, 4-py), 7.30-7.28 (m, 1H, 3-py), 7.13-7.10 (m, 1H, 5-py), 2.98 (s, 2H, NCH_2), 2.66 (br s, 2H, NH_2) 2.16-2.03 (m, 2H, CH), 1.93-1.87 (m, 2H, CH), 1.79-1.70 (m, 4H, CH); ^{13}C NMR (100.6 MHz, $CDCl_3$) δ 166.8 (*ipso*-Ar), 148.7 (Ar), 136.6 (Ar), 121.4 (Ar), 121.2 (Ar), 54.5 (NCH_2), 51.0 (C), 35.9 (CH_2), 24.6 (CH_2); MS (ESI) *m/z* 177 (M + H)$^+$; HRMS *m/z* calcd for $C_{11}H_{16}N_2$ (M + H)$^+$ 177.1386, found 177.1386 (–0.4 ppm error).
Lab Book Reference: PJ-07-81.

[4-(Pyridin-2-yl)oxan-4-yl]methyl methanesulfonate S15

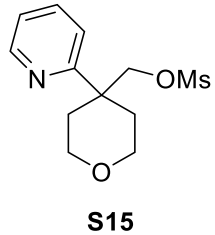

S15

MsCl (0.07 mL, 0.93 mmol, 1.2 eq.) was added dropwise to a stirred solution of aryl THP alcohol **8x** (150 mg, 0.78 mmol, 1.0 eq.) and Et_3N (0.11 mL, 1.55 mmol, 2.0 eq.) in CH_2Cl_2 (5 mL) at rt under Ar. The resulting solution was stirred at rt for 16 h. The reaction mixture was evaporated under reduced pressure to give the crude product as an orange oil. Purification by flash column chromatography on silica with 80:20 EtOAc-heaxane as eluent gave aryl THP mesylate **S15** (143 mg, 67%) as a colourless oil, R_F (80:20 EtOAc-heaxane) 0.26; IR (ATR) 2958, 1350, 1172, 952, 729 cm^{-1}; ^1H NMR (400 MHz, $CDCl_3$) δ 8.62 (ddd, J = 5.0, 2.0, 1.0 Hz, 1H, 6-py), 7.72-7.68 (m, 1H, 4-py), 7.35 (ddd, J = 8.0, 1.0, 1.0 Hz, 1H, 3-py), 7.19 (ddd, J = 7.5, 5.0, 1.0 Hz, 1H, 5-py), 4.34 (s, 2H, OCH_2), 3.83 (ddd, J = 12.0, 4.5,

4.5 Hz, 2H, OCH), 3.50 (ddd, J = 12.0, 9.5, 3.0 Hz, 2H, OCH), 2.67 (s, 3H, Me), 2.36 (dddd, J = 13.5, 4.5, 3.0 Hz, 2H, CH), 1.95 (ddd, J = 13.5, 9.5, 4.5 Hz, 2H, CH); ^{13}C NMR (100.6 MHz, $CDCl_3$) δ 160.8 (*ipso*-Ar), 149.7 (Ar), 136.6 (Ar), 122.4 (Ar), 122.1 (Ar), 76.8 ($MsOCH_2$), 64.1 (OCH_2), 42.8 (C), 36.8 (Me), 31.9 (CH_2); MS (ESI) *m/z* 272 $(M + H)^+$; HRMS *m/z* calcd for $C_{12}H_{17}NO_4S$ $(M + H)^+$ 272.0951, found 272.0944 (+2.1 ppm error).
Lab Book Reference: PJ-07-85.

2-[4-(Azidomethyl)oxan-4-yl]pyridine S16

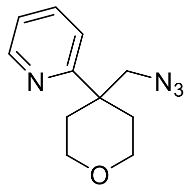

S16

A solution of aryl THP mesylate **S15** (120 mg, 0.44 mmol, 1.0 eq.) in DMF (2 mL) was added dropwise to a stirred solution of NaN_3 (86 mg, 1.33 mmol, 3.0 eq.) in DMF (1 mL) at rt under Ar. The resulting mixture was stirred and heated at 120 °C for 24 h. The reaction mixture was allowed to cool to rt and poured into water (10 mL) and Et_2O (10 mL). The two layers were separated, and the aqueous layer was extracted with Et_2O (3 × 5 mL). The combined organics were dried ($MgSO_4$) and evaporated under reduced pressure to give the crude product as a colourless oil. Purification by flash column chromatography on silica with 90:10 hexane-EtOAc as eluent gave aryl THP azide **S16** (80 mg, 84%) as a yellow oil, R_F (50:50 hexane-EtOAc) 0.32; IR (ATR) 2954, 2095 (N_3), 1587, 1105, 748 cm^{-1}; ^{1}H NMR (400 MHz, $CDCl_3$) δ 8.64 (ddd, J = 5.0, 2.0, 1.0 Hz, 1H, 6-py), 7.70 (ddd, J = 7.5, 7.5, 2.0 Hz, 1H, 4-py), 7.34 (ddd, J = 7.5, 1.0, 1.0 Hz, 1H, 3-py), 7.19 (ddd, J = 7.5, 5.0, 1.0 Hz, 1H, 5-py), 3.82 (ddd, J = 12.0, 4.5, 4.5 Hz, 2H, OCH), 3.52 (s, 2H, N_3CH_2), 3.48 (ddd, J = 12.0, 10.0, 2.5 Hz, 2H, OCH), 2.40-2.34 (m, 2H, CH), 1.89 (ddd, J = 14.0, 10.0, 4.5 Hz, 2H, CH); ^{13}C NMR (100.6 MHz, $CDCl_3$) δ 161.9 (*ipso*-Ar), 149.7 (Ar), 136.6 (Ar), 122.1 (Ar), 122.0 (Ar), 64.4 (OCH_2), 62.0 (N_3CH_2), 43.7 (C), 33.1 (CH_2); MS (ESI) *m/z* 219 $(M + H)^+$; HRMS *m/z* calcd for $C_{11}H_{14}N_4O$ $(M + H)^+$ 219.1240, found 219.1239 (+0.8 ppm error).

Lab Book Reference: PJ-07-88.

1-[4-(pyridin-2-yl)oxan-4-yl]methanamine 8ae

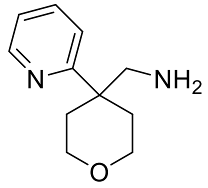

8ae

PPh_3 (194 mg, 0.74 mmol, 2.0 eq.) was added to a stirred solution of aryl THP azide **S16** (80 mg, 0.37 mmol, 1.0 eq.) in 5:1 THF-water (6 mL) at rt. The resulting mixture was stirred and heated at 65 °C for 16 h. The reaction mixture was allowed to cool to rt and evaporated under reduced pressure to give the crude product as a colourless oil. Purification by flash column chromatography on silica with 90:9:1 CH_2Cl_2-MeOH-$NH_4OH_{(aq)}$ as eluent gave aryl THP amine **8ae** (66 mg, 92%) as a yellow oil, R_F (90:9:1 CH_2Cl_2-MeOH-$NH_4OH_{(aq)}$) 0.18, IR (ATR) 3368 (NH), 2926, 2852, 1587, 1105, 749 cm^{-1}; ^{1}H NMR (400 MHz, $CDCl_3$) δ 8.62-8.61 (m, 1H, 6-py), 7.70-7.66 (m, 1H, 4-py), 7.31-7.26 (m, 1H, 3-py), 7.17-7.13 (m, 1H, 5-py), 3.84-3.79 (m, 2H, OCH), 3.49-3.43 (m, 2H, OCH), 2.88 (s, 2H, NCH_2), 2.37-2.32 (m, 2H, CH), 1.85-1.77 (m, 2H, CH); ^{13}C NMR (100.6 MHz, $CDCl_3$) δ 163.5 (*ipso*-Ar), 149.5 (Ar), 136.5 (Ar), 121.8 (Ar), 121.4 (Ar), 64.7 (OCH_2), 53.6 (NCH_2), 44.1 (C), 33.4 (CH_2); MS (ESI) *m/z* 193 (M + H)$^+$; HRMS *m/z* calcd for $C_{11}H_{16}N_2O$ (M + H)$^+$ 193.1335, found 193.1333 (+1.6 ppm error). Spectroscopic data consistent with those reported in the literature.[11]

Lab Book Reference: PJ-07-94.

***tert*-Butyl (3*R**,4*R**)-3-(2-fluorophenyl)-4-(hydroxymethyl)pyrrolidine-1-carboxylate S17**

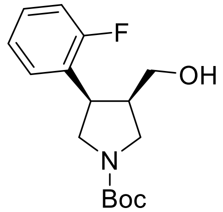

S17

Using general procedure H, $LiAlH_4$ (55 mg, 1.44 mmol, 2.0 eq.) in THF (5 mL), ester **2i** (233 mg, 0.72 mmol, 1.0 eq.) in THF (5 mL) gave alcohol **S17** (166 mg, 56%) as a yellow oil, IR (ATR) 3280 (OH), 1696 (C=O), 871 cm^{-1}; ^{1}H NMR (400 MHz, $CDCl_3$) δ 7.27-7.21 (m, 1H, Ar), 7.16-7.10 (m, 2H, Ar), 7.07-7.03 (m, 1H, Ar), 3.86-3.60 (m, 4H, NCH), 3.42-3.37 (m, 1H, HOC*H*), 3.34-3.27 (m, 2H, C*H*Ar,

HOC*H*), 2.80-2.75 (m, 1H, C*H*CH_2OH), 1.48 (s, 9H, CMe_3); ^{13}C NMR (100.6 MHz, $CDCl_3$) (rotamers) δ 161.0 (d, *J* = 236 Hz, CF), 156.2 (C=O, Boc), 129.8 (d, *J* = 8.5 Hz, Ar), 128.6 (d, *J* = 8.5 Hz, Ar), 128.4 (d, *J* = 4.5 Hz, Ar), 127.6 (d, *J* = 5.0 Hz *ipso*-Ar), 126.4 (d, *J* = 5.0 Hz, Ar), 124.8 (d, *J* = 4.1 Hz, Ar), 124.7 (d, *J* = 3.9 Hz, Ar), 115.4 (d, *J* = 25.4 Hz, Ar), 79.8 (*C*Me_3), 62.0 ($HOCH_2$), 50.0 (NCH_2), 48.0 (NCH_2), 47.6 (NCH_2), 44.8 (*C*HCH_2OH), 44.1 (*C*HCH_2OH), 37.1 (*C*HAr), 37.0 (*C*HAr), 28.7 (C*Me*$_3$); MS (ESI) *m/z* 295 [(M + H), 100]; HRMS (ESI) *m/z* calcd for $C_{16}H_{22}FNO_3$ $(M + H)^+$ 295.1584, found 295.1580 (–1.0 ppm error).

Lab Book Reference: TD 5/30

[(3*R,4*R**)-4-(2-fluorophenyl)pyrrolidin-3-yl]methanol hydrochloride 8af•HCl**

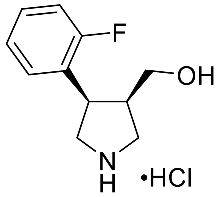

8af•HCl

Using general procedure G, *N*-Boc pyrrolidine **S17** (136 mg, 0.46 mmol, 1.0 eq.) in Et_2O (50 mL) and HCl (2.30 mL of a 2 M solution in Et_2O, 4.60 mmol, 10.0 eq.) gave pyrrolidine **8af**•HCl (106 mg, 97%) as a white solid, mp 53-55 °C; IR (ATR) 3359 (OH or NH), 2925, 1493, 757 cm^{-1}; ^{1}H NMR (400 MHz, MeOD-*d*4) δ 7.42-7.33 (m, 2H, Ar), 7.18 (dd, *J* = 7.5, 7.5 Hz, 1H, Ar), 7.13 (dd, *J* = 10.5, 8.5 Hz, 1H, Ar), 4.00-3.95 (m, 1H, NCH), 3.78 (dd, *J* = 11.0, 11.0 Hz, 1H, NCH), 3.68-3.54 (m, 3H, C*H*Ar, NCH), 3.37 (dd, *J* = 10.5, 4.0 Hz, 1H, HOC*H*), 3.21 (dd, *J* = 10.5, 5.5 Hz, 1H, HOC*H*), 2.80 (br s, 1H, C*H*CH_2OH); ^{13}C NMR (100.6 MHz, MeOD-*d*4) δ 162.4 (d, *J* = 244.5 Hz, CF), 130.6 (d, *J* = 8.5 Hz, Ar), 130.0 (d, *J* = 4.0 Hz, Ar), 125.6 (d, *J* = 3.5 Hz, Ar), 124.3 (d, *J* = 14.5 Hz, *ipso*-Ar), 116.4 (d, *J* = 22.5 Hz, Ar), 61.4 ($HOCH_2$), 49.6 (NCH_2), 49.2 (NCH_2), 43.4 (C*H*CH_2OH), 40.4 (d, *J* = 2.0 Hz, *C*HAr); MS (ESI) *m/z* 196 [M$^+$, 100]; HRMS (ESI) *m/z* calcd for $C_{11}H_{15}FNO$ M$^+$ 196.1132, found 196.1131 (+0.8 ppm error).

Lab Book Reference: TD 5/35

***tert*-Butyl 3-(hydroxymethyl)-3-(4-fluorophenyl)piperidine-1-carboxylate S18**

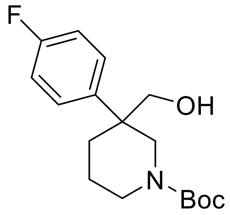

S18

Using general procedure H, $LiAlH_4$ (77 mg, 2.03 mmol, 2.0 eq.) in THF (8 mL), ester **4q** ((300 mg, 0.89 mmol, 1.0 eq.) in THF (7 mL) gave *N*-Boc aryl piperidine alcohol **S18** (268 mg, 98%) as a white solid, mp 98-100 °C; R_F (70:30 hexane-EtOAc) 0.12; IR (ATR) 3517 (OH), 2859, 1666 (C=O, Boc), 1431, 1156, 1060, 834 cm^{-1}; ^{1}H NMR (400 MHz, MeOD-d_4) (50:50 mixture of rotamers) δ 7.46 (dd, J = 9.0, 5.0 Hz, 2H, Ar), 70.4 (m, 2H, Ar), 4.30 (d, J = 13.5 Hz, 0.5H, NCH), 4.16 (d, J = 13.5 Hz, 0.5H, NCH), 3.72-3.65 (m, 1H, NCH), 3.52-3.50 (m, 2H, OCH_2), 3.32-3.30 (m, 1H, NCH), 3.27-3.25 (m, 0.5H, NCH), 3.23-3.15 (m, 0.5H, NCH), 2.16-2.07 (m, 1H, CH), 1.85 (m, 1H, CH), 1.57 (m, 1H, CH), 1.47-1.29 (m, 10H, C*Me*$_3$ and CH); ^{13}C NMR (100.6 MHz, MeOD-d_4) (rotamers) δ 162.8 (d, J = 243.5 Hz, *ipso*-Ar), 156.5 (C=O , Boc), 139.9 (*ipso*-Ar), 130.4 (Ar), 115.7 (d, J = 21.0 Hz, Ar), 81.3 (O*C*Me$_3$), 81.1 (O*C*Me$_3$), 70.5 (OCH_2), 70.1 (OCH_2), 51.0 (NCH_2), 48.4 (NCH_2), 45.8 (NCH_2), 45.1 (C), 44.8 (NCH_2), 32.3 (CH_2), 31.9 (CH_2), 28.7 (C*Me*$_3$), 22.3 (CH_2); MS (ESI) *m/z* 332 (M + Na)$^+$; HRMS *m/z* calcd for $C_{17}H_{24}FNO_3$ (M + Na)$^+$ 332.1632, found 332.1622 (+4.5 ppm error).

Lab Book Reference: PJ-07-78.

[3-(4-Fluorophenyl)piperidin-3-yl]methanol hydrochloride 8ag

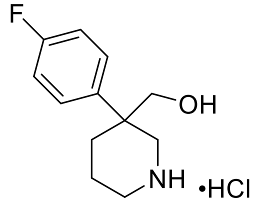

8ag•HCl

Using general procedure G, *N*-Boc pyrrolidine **S17** (90 mg, 0.29 mmol, 1.0 eq.) and HCl (1 mL of a 4 M solution in dioxane) gave pyrrolidine **8ag**•HCl (70 mg, 99%) as a white solid, mp 100-102 °C; IR (ATR) 3346 (OH), 2950, 1513, 1234, 832, 727 cm^{-1}; ^{1}H NMR (400 MHz, $CDCl_3$) δ 9.83-8.83 (m, 1H,

NH), 7.35-7.31 (m, 2H, Ar), 7.05 (dd, J = 8.5, 8.5 Hz, 2H, Ar), 4.19 (d, J = 12.0 Hz, 1H, OCH), 4.03 (d, J = 13.0 Hz, 1H, NCH), 3.72 (d, J = 12.0 Hz, 1H, OCH), 3.53-3.49 (m, 1H, NCH), 3.03 (d, J = 13.0 Hz, 1H, NCH), 2.92-2.86 (m, 1H, NCH), 2.25-2.11 (m, 2H, CH), 1.93-1.87 (m, 2H, CH); ^{13}C NMR (100.6 MHz, $CDCl_3$) δ 161.9 (d, J = 247.0 Hz, *ipso*-Ar), 138.6 (d, J = 3.0 Hz, *ipso*-Ar), 127.5 (d, J = 8.0 Hz, Ar), 115.8 (d, J = 21.0 Hz, Ar), 66.5 (OCH_2), 48.5 (NCH_2), 43.7 (NCH_2), 40.4 (C), 30.8 (CH_2), 19.4 (CH_2); MS (ESI) *m/z* 210 $(M)^+$; HRMS *m/z* calcd for $C_{12}H_{18}FNO$ $(M)^+$ 210.1289, found 210.1287 (+0.7 ppm error).

Lab Book Reference: PJ-07-87.

tert-Butyl 3-(4-fluorophenyl)-3-{[(pyridine-2-sulfonyl)oxy]methyl}piperidine-1-carboxylate S19

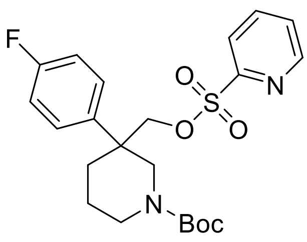

S19

DBU (0.12 mL, 0.80 mmol, 2.0 eq.) was added dropwise to a stirred solution of *N*-Boc aryl piperidine alcohol **S18** (124 mg, 0.40 mmol, 1.0 eq.) and PyFluor (71 mg, 0.44 mmol, 1.1 eq.) at rt under Ar. The resulting solution was stirred and heated at 80 °C for 48 h. The reaction mixture was allowed to cool to rt and evaporated under reduced pressure to give the crude product as an orange oil. Purification by flash column chromatography on silica with 99:1 CH_2Cl_2-acetone as eluent gave *N*-Boc aryl piperidine sulfonate **S19** (137 mg, 76%) as a colourless oil, R_F (99:1 CH_2Cl_2-acetone) 0.17; IR (ATR) 2976, 1638 (C=O), 1427, 1364, 1154, 830, 593 cm^{-1}; ^{1}H NMR (400 MHz, $CDCl_3$) δ 8.63 (ddd, J = 5.0, 1.0, 1.0 Hz, 1H, 6-py), 7.88-7.81 (m, 1H, 4-py and 3-py), 7.51 (ddd, J = 7.0, 5.0, 1.0 Hz, 1H, 5-py), 7.28-7.24 (m, 2H, Ar), 6.90 (dd, J = 8.5, 8.5 Hz, 2H, Ar), 4.34 (s, 2H, OCH_2), 3.98 (m, 1H, NCH), 3.53-3.49 (m, 2H, NCH), 3.24-3.18 (m, 1H, NCH), 2.05-1.96 (m, 2H, CH), 1.72-1.54 (m, 1H, CH), 1.46-1.24 (m, 10H, CMe_3 and CH); ^{13}C NMR (100.6 MHz, $CDCl_3$) (rotamers) δ 161.7 (d, J = 246.0 Hz, *ipso*-Ar), 154.9 (C=O or *ipso*-Ar), 154.5 (C=O or *ipso*-Ar), 150.2 (Ar), 138.2 (Ar), 136.0 (d, J = 3.5 Hz, Ar), 128.7 (d, J = 8.0 Hz, Ar), 127.6 (Ar), 123.1 (Ar), 115.3 (d, J = 21.0 Hz, Ar), 80.1 ($OCMe_3$), 77.4 (OCH_2), 49.4 (NCH_2), 48.4 (NCH_2), 44.5 (NCH_2), 43.5 (NCH_2), 42.2 (C), 31.0 (CH_2), 28.5 (CMe_3), 21.0 (CH_2); MS

(ESI) *m/z* 473 $(M + Na)^+$; HRMS *m/z* calcd for $C_{22}H_{27}FN_2O_5S$ $(M + Na)^+$ 473.1517, found 473.1512 (+1.7 ppm error).

Lab Book Reference: PJ-07-80.

***tert*-Butyl 3-(azidomethyl)-3-(4-fluorophenyl)piperidine-1-carboxylate S20**

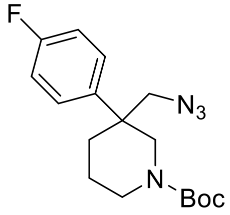

S20

A solution of *N*-Boc aryl piperidine sulfonate **S19** (137 mg, 0.30 mmol, 1.0 eq.) in DMF (3 mL) was added dropwise to a stirred solution of NaN_3 (59 mg, 0.91 mmol, 3.0 eq.) in DMF (1 mL) at rt under Ar. The resulting mixture was stirred and heated at 120 °C for 24 h. The reaction mixture was allowed to cool to rt and poured into water (10 mL) and Et_2O (10 mL). The two layers were separated, and the aqueous layer was extracted with Et_2O (3 × 5 mL). The combined organics were dried ($MgSO_4$) and evaporated under reduced pressure to give a yellow oil. Purification by flash column chromatography on silica with 95:5-80:20 hexane-EtOAc as eluent gave *N*-Boc aryl piperidine azide **S20** (66 mg, 65%) as a colourless oil, R_F (95:5 hexane-EtOAc) 0.06; IR (ATR) 2935, 2098 (N_3), 1683 (C=O), 1425, 1153, 832 cm^{-1}; ^{1}H NMR (400 MHz, $CDCl_3$) δ 7.38-7.34 (m, 2H, Ar), 7.04 (dd, J = 9.0, 9.0 Hz, 2H, Ar), 3.90 (d, J = 13.5 Hz, 1H, NCH), 3.50-3.47 (m, 2H, NCH), 3.44-3.40 (m, 2H, N_3CH), 3.36-3.32 (m, 1H, NCH), 2.02-1.99 (m, 1H, CH), 1.92-1.89 (m, 1H, CH), 1.68-1.57 (m, 2H, CH), 1.57-1.44 (m, 9H, CMe_3); ^{13}C NMR (100.6 MHz, $CDCl_3$) (rotamers) δ 161.8 (d, J = 246.0 Hz, *ipso*-Ar), 154.7 (C=O or *ipso*-Ar) 154.6 (C=O or *ipso*-Ar), 137.7 (d, J = 2.5 Hz, *ipso*-Ar), 128.5 (d, J = 8.0 Hz, Ar), 115.5 (d, J = 21.0 Hz, Ar), 80.1 (O*C*Me_3), 59.8 (N_3CH_2), 50.4 (NCH_2), 49.5 (NCH_2), 44.6 (NCH_2), 43.6 (NCH_2), 42.6 (C), 32.6 (CH_2), 32.3 (CH_2), 28.5 (C*Me*$_3$), 21.4 (CH_2); MS (ESI) *m/z* 357 $(M + Na)^+$; HRMS *m/z* calcd for $C_{17}H_{23}FN_4O_2$ $(M + Na)^+$ 357.1697, found 357.1695 (+0.0 ppm error).

Lab Book Reference: PJ-07-91

***tert*-Butyl 3-(aminomethyl)-3-(4-fluorophenyl)piperidine-1-carboxylate S21**

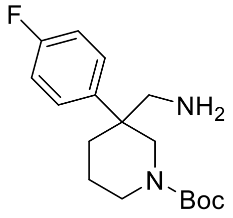

S21

PPh_3 (103 mg, 0.40 mmol, 2.0 eq.) was added to a stirred solution of *N*-Boc aryl piperidine azide **S20** (66 mg, 0.20 mmol, 1.0 eq.) in 5:1 THF-water (6 mL) at rt. The resulting mixture was stirred and heated at 65 °C for 16 h. The reaction mixture was allowed to cool to rt and evaporated under reduced pressure to give the crude product as a colourless oil. Purification by flash column chromatography on silica with 95:5 CH_2Cl_2-MeOH as eluent gave *N*-Boc aryl piperidine amine **S21** (49 mg, 79%) as a white solid, mp 82-84 °C; R_F (95:5 CH_2Cl_2-MeOH) 0.05; IR (ATR) 3360 (NH), 2979, 1676 (C=O), 1431, 1120, 836 cm^{-1}; ^{1}H NMR (400 MHz, $CDCl_3$) δ 7.30 (dd, J = 9.0, 5.5 Hz, 2H, Ar), 7.03 (dd, J = 9.0, 9.0 Hz, 2H, Ar), 3.83-3.73 (m, 1H, NCH), 3.64-3.50 (m, 1H, NCH), 3.46-3.32 (m, 2H, NCH), 2.84-2.72 (m, 2H, NCH), 1.96-1.90 (m, 1H, CH), 1.85-1.79 (m, 1H, CH), 1.61 (m, 1H, CH), 1.48-1.45 (m, 10H, CMe_3 and CH); ^{13}C NMR (100.6 MHz, $CDCl_3$) (rotamers) δ 161.4 (d, J = 245.5 Hz, *ipso*-Ar), 155.2 (C=O, Boc), 154.6 (C=O, Boc), 139.1 (*ipso*-Ar), 138.9 (*ipso*-Ar), 128.5 (d, J = 6.0 Hz, Ar), 115.4 (d, J = 21.0 Hz, Ar), 79.9 (O*C*Me_3), 50.6 (NCH_2), 50.2 (NCH_2), 49.1 (NCH_2), 44.8 (NCH_2), 43.9 (NCH_2), 43.4 (C), 33.6 (CH_2), 33.3 (CH_2), 28.6 (C*Me_3*), 21.7 (CH_2), 21.5 (CH_2); MS (ESI) *m/z* 309 (M + H)$^+$; HRMS *m/z* calcd for $C_{17}H_{25}FN_2O_2$ (M + H)$^+$ 309.1973, found 309.1974 (–0.6 ppm error).
Lab Book Reference: PJ-08-02

1-[3-(4-fluorophenyl)piperidin-3-yl]methanamine dihydrochloride 8ah·2HCl

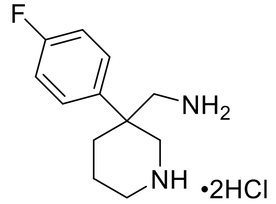

8ah•2HCl

Using general procedure G, *N*-Boc pyrrolidine **S21** (49 mg, 0.15 mmol, 1.0 eq.) and HCl (1 mL of a 4 M solution in dioxane) gave pyrrolidine **8ah**•HCl (36 mg, 99%) as a white solid, mp 242-244 °C; IR (ATR) 3391 (NH), 2827, 1607, 1513, 1227, 1173, 842 cm^{-1}; ^{1}H NMR (400 MHz, MeOD-*d_4*) δ 7.62 (dd, J = 9.0, 5.0 Hz, 2H, Ar), 7.31 (dd, J = 9.0, 9.0 Hz, 2H, Ar), 3.92 (d, J = 13.5 Hz, 1H, NCH), 3.51 (d, J = 13.5 Hz,

1H, NCH), 3.31 (s, 2H, NCH), 3.20-3.19 (m, 2H, NCH), 2.60-2.56 (m, 1H, CH), 2.09-1.89 (m, 2H, CH), 1.88-1.86 (m, 1H, CH); ^{13}C NMR (100.6 MHz, d$_4$-MeOH) δ 164.1 (d, J = 247.0 Hz, *ipso*-Ar), 133.5 (d, J = 3.0 Hz, *ipso*-Ar), 130.5 (d, J = 8.5 Hz, Ar), 117.9 (d, J = 21.5 Hz, Ar), 50.3 (NCH_2), 49.6 (NCH_2) 45.3 (NCH_2), 40.8 (C), 29.8 (CH_2), 19.7 (CH_2); MS (ESI) *m/z* 209 (M)$^+$; HRMS *m/z* calcd for $C_{12}H_{18}FN_2$ (M)$^+$ 209.1449, found 209.1449 (+2.9 ppm error).

Lab Book Reference: PJ-08-11.

4-(4-Methylphenyl)oxane-4-carboxylic acid 8ai

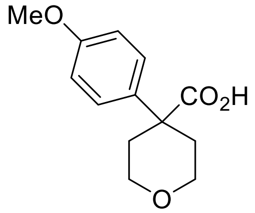

8ai

KOH (444 mg, 7.92 mmol, 10.0 eq.) was added to a stirred solution of aryl THP-4-methyl ester **4r** (198 mg, 0.79 mmol, 1.0 eq.) in EtOH (10 mL) at rt. The resulting mixture was stirred and heated at 100 °C for 16 h. The reaction mixture was allowed to cool to rt and evaporated under reduced pressure to give the crude product as an orange solid. The solid was taken up into water (20 mL) and washed with CH_2Cl_2 (3 × 10 mL). The aqueous layer was acidified with 1M $HCl_{(aq)}$ (1 mL) and extracted with CH_2Cl_2 (3 × 10 mL). The combined organics were washed with brine (20 mL), dried (Na_2SO_4) and evaporated under reduced pressure to give aryl THP-4-carboxylic acid **8ai** (164 mg, 88%) as a cream solid, mp 160-162 °C; IR (ATR) 2972, 1673 (C=O), 1511, 1257, 1239, 1027 cm^{-1}; ^{1}H NMR (400 MHz, $CDCl_3$) δ 7.33 (d, J = 9.0 Hz, 2H, Ar), 6.89 (d, J = 9.0 Hz, 2H, Ar), 3.91 (ddd, J = 12.0, 4.0, 4.0 Hz, 2H, OCH), 3.80 (s, 3H, OMe), 3.64-3.58 (m, 2H, OCH), 2.52-2.47 (m, 2H, CH), 1.96 (ddd, J = 14.0, 11.0, 4.0 Hz, 2H, CH); ^{13}C NMR (100.6 MHz, $CDCl_3$) δ 180.2 (C=O), 159.0 (*ipso*-Ar), 133.8 (*ipso*-Ar), 127.2 (Ar), 114.2 (Ar), 65.6 (OCH_2), 55.4 (OMe), 47.6 (C), 34.2 (CH_2); MS (ESI) *m/z* 259 (M + Na)$^+$; HRMS *m/z* calcd for $C_{13}H_{16}O_4$ (M + Na)$^+$ 259.0941, found 259.0929 (+2.9 ppm error).

Lab Book Reference: PJ-07-83.

1-[(*tert*-Butoxy)carbonyl]-3-[(4-fluorophenyl)methyl]pyrrolidine-3-carboxylic acid S22

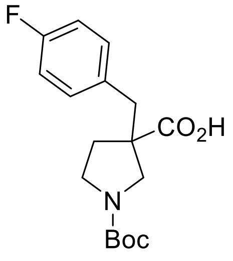

S22

KOH (83 mg, 1.5 mmol, 10.0 eq.) was added to a stirred solution of *N*-Boc aryl pyrrolidine-3-methyl ester **5e** (50 mg, 0.15 mmol, 1.0 eq.) in EtOH (10 mL) at rt. The resulting mixture was stirred and heated at 100 °C for 16 h. The reaction mixture was allowed to cool to rt and evaporated under reduced pressure to give the crude product as an orange solid. The solid was taken up into water (10 mL) and washed with CH_2Cl_2 (3 × 50 mL). The aqueous layer was then acidified with 1 M $HCl_{(aq)}$ (1 mL) and extracted with CH_2Cl_2 (3 × 10 mL). The combined organics were washed with brine (10 mL), dried (Na_2SO_4) and evaporated under reduced pressure to give *N*-Boc aryl pyrrolidine-3-carboxylic acid **S22** (48 mg, 100%) as a white solid, mp 122-124 °C; IR (ATR) 2915, 1717 (C=O, CO_2H), 1632 (C=O, Boc), 1433, 1141, 893 cm^{-1}; ^1H NMR (400 MHz, $CDCl_3$) (50:50 mixture of rotamers) δ 7.13-7.09 (m, 2H, Ar), 6.99-6.95 (m, 2H, Ar), 3.82 (d, J = 11.5 Hz, 0.5H, CHAr), 3.71 (d, J = 11.5 Hz, 0.5H, CHAr), 3.49-3.31 (m, 3H, NCH and CHAr), 3.05-2.94 (m, 2H, CH), 2.35-2.26 (m, 1H, CH), 1.93-1.86 (m, 1H, CH), 1.46 (s, 9H, C*Me*$_3$); ^{13}C NMR (100.6 MHz, $CDCl_3$) (rotamers) δ 181.9 (C=O, CO_2H), 179.3 (C=O, CO_2H), 162.1 (d, J = 246.0 Hz, *ipso*-Ar), 154.7 (C=O, Boc), 132.5 (*ipso*-Ar), 131.2 (d, J = 8.0 Hz, Ar), 115.5 (d, J = 21.0 Hz, Ar), 80.1 (O*C*Me$_3$), 79.9 (O*C*Me$_3$), 54.5 (C), 53.8 (C), 53.0 (CH_2Ar), 44.9 (NCH_2), 44.4 (NCH_2), 40.9 (CH_2), 40.6 (CH_2), 33.5 (CH_2), 32.9 (CH_2), 28.6 (C*Me*$_3$); MS (ESI) *m/z* 346 $(M + Na)^+$; HRMS *m/z* calcd for $C_{17}H_{22}FNO_4$ $(M + Na)^+$ 346.1425, found 346.1427 (–1.4 ppm error).

Lab Book Reference: PJ-08-07.

3-[(4-Fluorophenyl)methyl]pyrrolidine-3-carboxylic acid hydrochloride 8aj·HCl

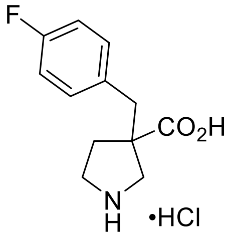

8aj•HCl

Using general procedure G, *N*-Boc pyrrolidine **S22** (45 mg, 0.14 mmol, 1.0 eq.) and HCl (1 mL of a 4 M solution in dioxane) gave pyrrolidine **8aj**•HCl (36 mg, 99%) as a white solid, mp 148–150 °C; IR (ATR) 2924, 1722 (C=O), 1604, 1510, 1394, 1221, 1161, 840, 763 cm^{-1}; ^{1}H NMR (400 MHz, MeOD-d_4) δ 7.28–7.24 (m, 2H, Ar), 7.07–7.01 (m, 2H, Ar), 3.66 (d, J = 14.0 Hz, 1H, CH_2Ar), 3.52–3.43 (m, 1H, CH_2), 3.32–3.20 (m, 3H, CH_2), 3.04 (d, J = 14.0 Hz, 1H, CH_2Ar), 2.50–2.44 (m, 1H, CH_2), 2.16–2.08 (m, 1H, CH_2); ^{13}C NMR (101 MHz, MeOD-d_4) δ 176.1 (C=O), 163.5 (d, J = 244.0 Hz, *ipso*-Ar), 133.9 (*ipso*-Ar), 132.4 (d, J = 8.0 Hz, Ar), 116.3 (d, J = 21.5 Hz, Ar), 56.0 (*C*CO_2H), 52.4 (CH_2), 45.8 (CH_2), 41.3 (CH_2), 35.1 (CH_2); HRMS (ESI) *m/z* calcd for $C_{12}H_{15}FNO_2$ M$^+$ 224.1081, found 224.1080 (+0.5 ppm error).

Lab Book Reference: JDF_B_397_10

***tert*-Butyl 3-{[(*tert*-butoxy)carbonyl]amino}-3-[(4-fluorophenyl)methyl]pyrrolidine-1-carboxylate S23**

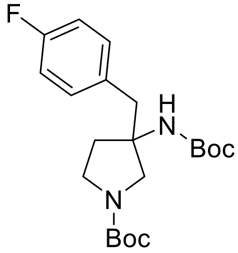

S23

Et_3N (0.06 mL, 0.44 mmol, 2.0 eq.) was added dropwise to a stirred solution of *N*-Boc pyrrolidine-3-carboxylic acid **S22** (72 mg, 0.22 mmol, 1.0 eq.), DPPA (0.06 mL, 0.27 mmol, 1.2 eq.) and 3Å MS (50 mg) in *t*-BuOH (10 mL) at rt under Ar. The resulting solution was stirred and heated at 40 °C for 1 h and then at 100 °C for 16 h. The reaction mixture was allowed to cool to rt and the solids were removed by filtration through Celite and washed with MeOH (10 mL). The filtrate was evaporated under redued pressure to give the crude product as a yellow oil. Purification by flash column chromatography on silica with 95:5-80:20 hexane-EtOAc as eluent gave *N*-Boc aryl pyrrolidine amine **S23** (44 mg, 51%) as a

colourless oil, R_F (80:20 hexane-EtOAc) 0.25; IR (ATR) 2977, 1627 (C=O), 1509, 1404, 1216, 1156 cm^{-1}; ^{1}H NMR (400 MHz, $CDCl_3$) (50:50 mixture of rotamers) δ 7.10-7.05 (m, 2H, Ar), 7.00-6.93 (m, 2H, Ar), 3.50-3.33 (m, 5H, NCH and CHAr), 2.99-2.93 (m, 1H, CHAr), 2.35 (m, 0.5H, CH), 1.98 (m, 0.5H, CH), 1.86 (ddd, J = 12.5, 8.5, 8.5 Hz, 1H, CH), 1.50-1.41 (m, 18H, CMe_3); ^{13}C NMR (100.6 MHz, $CDCl_3$) (rotamers) δ 161.9 (d, J = 244.5 Hz, *ipso*-Ar), 154.7 (C=O, Boc), 133.2 (*ipso*-Ar), 131.4 (d, J = 7.0 Hz, Ar), 115.2 (d, J = 21.0 Hz, Ar), 79.8 (O*C*Me_3), 79.7 (O*C*Me_3), 62.0 (C), 61.6 (C), 56.1 (NCH_2), 44.4 (NCH_2), 43.7 (NCH_2), 39.6 (CH_2Ar), 39.2 (CH_2Ar), 35.4 (CH_2), 34.9 (CH_2), 29.8 (C*Me_3*), 28.6 (C*Me_3*), 28.5 (C*Me_3*); MS (ESI) *m/z* 417 (M + Na)$^+$; HRMS *m/z* calcd for $C_{21}H_{31}FN_2O_4$ (M + Na)$^+$ 417.2160, found 417.2160 (+0.0 ppm error).
Lab Book Reference: PJ-07-70.

3-[(4-Fluorophenyl)methyl]pyrrolidin-3-amine dihydrochloride 8ak·HCl

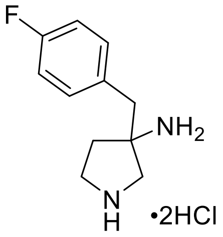

8ak•2HCl

Using general procedure G, *N*-Boc pyrrolidine **S23** (46 mg, 0.12 mmol, 1.0 eq.) and HCl (1 mL of a 4 M solution in dioxane) gave pyrrolidine **8ak**•HCl (32 mg, 99%) as a white solid, mp 68-70 °C; IR (ATR) 2923, 2852, 2484, 2220, 2072, 1608, 1513, 1227, 1118, 971, 825, 499 cm^{-1}; ^{1}H NMR (400 MHz, MeOD-*d*4) δ 7.45–7.41 (m, 2H, Ar), 7.19–7.15 (m, 2H, Ar), 3.72–3.65 (m, 2H, CH_2), 3.59 (d, J = 13.5 Hz, 1H, CH_2), 3.52 (ddd, J = 12.0, 8.0, 6.5 Hz, 1H, CH_2), 3.27 (d, J = 14.5 Hz, 1H, CH_2), 3.22 (d, J = 14.5 Hz, 1H, CH_2), 2.54–2.47 (m, 1H, CH_2), 2.36–2.29 (m, 1H, CH_2); ^{13}C NMR (101 MHz, MeOD-*d*4) δ 164.2 (d, J = 246.0 Hz, *ipso*-Ar), 133.6 (d, J = 8.5 Hz, Ar), 130.4 (d, J = 3.5 Hz, *ipso*-Ar), 117.2 (d, J = 22.0 Hz, Ar), 63.4 (CNH_2), 53.0 (CH_2), 45.7 (CH_2), 41.5 (CH_2), 34.9 (CH_2); HRMS (ESI) *m/z* calcd for $C_{11}H_{16}FN_2$ M$^+$ 195.1292, found 195.1289 (+1.7 ppm error).

Lab Book Reference: +_B_397_13

Benzyl *N*-[(3*R,4*S**)-4-(3-methoxyphenyl)oxan-3-yl]carbamate S24**

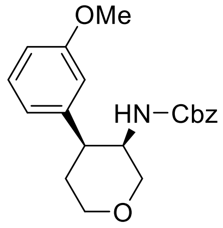

S24

Diphenylphosphorylazide (0.161 mL, 0.745 mmol, 1.1 eq.) was added dropwise to a stirred solution of acid **7k** (160 mg, 0.677 mol, 1 eq.) and Et_3N (0.189 mL, 1.35 mmol, 2 eq.) in benzyl alcohol (2 mL) at rt under Ar. The resulting solution was stirred at rt for 1 h and then stirred and heated at 85 °C for 16 h. The resulting solution was allowed to cool to rt and the solids were removed by filtration through Celite. The filtrate was evaporated under reduced pressure to give the crude product. Purification by flash column chromatography with 50:50 hexane-EtOAc as eluent gave Cbz-protected amine **S24** (144 mg, 62%) as a colourless oil, R_F 0.33 (50:50 hexane-EtOAc); IR (ATR) 2954 (NH), 1709 (C=O), 1234, 696 cm^{-1}; ^{1}H NMR (400 MHz, $CDCl_3$) (80:20 mixture of rotamers) δ 7.35-7.19 (m, 5.6H, Ar), 6.97 (br s, 0.4H, Ar), 6.84-6.75 (m, 3H, Ar), 5.28 (br d, J = 9.0 Hz, 1H, NH), 4.94 (d, J = 12.5 Hz, 0.8H, OC*H*Ph), 4.90 (d, J = 12.5 Hz, 0.8H, OC*H*Ph), 4.85 (d, J = 12.5 Hz, 0.2H, OC*H*Ph), 4.42 (d, J = 12.5 Hz, 0.2H, OC*H*Ph), 4.13-4.03 (m, 1.8H, OCH_2CH$_2$, C*H*NH), 4.00 (d, J = 11.5 Hz, 0.8H, OCH_2CH), 3.97-3.79 (m, 0.4H, OCH_2CH$_2$, C*H*NH), 3.77 (s, 2.4H, OMe), 3.74 (s, 0.6H, OMe), 3.66 (br d, J = 11.5 Hz, 1H, OCH_2CH), 3.54 (br dd, J = 12.5, 12.5 Hz, 1H, C*H*NH), 3.10-3.00 (m, 1H, C*H*Ar), 2.12-2.10 (m, 0.2H, CH), 2.05 (dddd, J = 12.5, 12.5, 12.5, 4.5 Hz, 0.8H, CH), 1.76-1.67 (m, 0.8H, CH), 1.63-1.57 (m, 0.2H, CH); ^{13}C NMR (100.6 MHz, $CDCl_3$) δ 159.7 (*ipso*-Ar), 155.9 (C=O), 142.9 (*ipso*-Ar), 136.7 (*ipso*- Ar), 129.5 (Ar), 128.6 (Ar), 128.1 (Ar), 128.0 (Ar), 119.9 (Ar), 113.3 (Ar), 112.1 (Ar), 72.4 (O*C*H$_2$CH), 68.3 (O*C*H$_2$CH$_2$), 66.6 (O*C*H$_2$Ph), 55.3 (OMe), 50.8 (CHNH), 43.3 (*C*HAr), 26.1 (CH$_2$); MS (ESI) *m/z* 342 [(M + H)$^+$, 10], 364 [(M + Na)$^+$, 100]; HRMS (ESI) *m/z* calcd for $C_{20}H_{23}O_4N$ (M + Na)$^+$ 364.1519, found 364.1516 (+1.0 ppm error).

Lab Book Reference: TD 5/29

(3*R,4*S**)-4-(3-Methoxyphenyl)oxan-3-amine 8al**

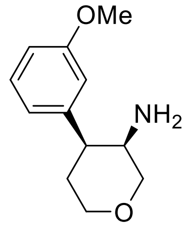

8al

Using general procedure C, Cbz-protected amine **S24** (144 mg, 0.42 mmol, 1.0 eq.) and 10% $Pd(OH)_2/C$ (14 mg, 0.01 mmol, 0.02 eq.) in MeOH (10 mL) for 16 h gave amine **8al** as a yellow oil (89 mg, quant.), IR (ATR) 3362 (NH_2), 3290 (NH_2), 1600, 1583, 725 cm^{-1}; ^{1}H NMR (400 MHz, $CDCl_3$) δ 7.27-7.23 (m, 1H, Ar), 6.82-6.76 (m, 3H, Ar), 4.17-4.04 (m, 4H, OCH, NH_2), 3.79 (s, 3H, OMe), 3.62 (br d, J = 12.0 Hz, 1H, OCH_2CH), 3.47-3.41 (m, 1H, OCH), 3.19 (br s, 1H, C$H$$NH_2$), 3.06-3.01 (m, 1H, C$H$Ar), 2.42 (dddd, J = 13.0, 13.0, 13.0, 4.5 Hz, 1H, OCH_2CH), 1.58 (br d, J = 13.0 Hz, 1H, OCH_2CH_2); ^{13}C NMR (100.6 MHz, $CDCl_3$) δ 160.1 (*ipso*-Ar), 142.7 (*ipso*-Ar) , 130.0 (Ar), 119.9 (Ar), 113.5 (Ar), 112.5 (Ar), 71.5 (OCH$_2$CH), 68.3 (OCH$_2$CH$_2$), 55.4 (OMe), 51.7 ($CHNH_2$), 43.6 (CHAr), 24.3 ($OCH_2$$CH_2$); MS (ESI) m/z 208 [$(M + H)^+$, 100], 230 [$(M + Na)^+$, 6], ; HRMS (ESI) m/z calcd for $C_{12}H_{17}NO_2$ $(M + H)^+$ 208.1332, found 208.1329 (+1.2 ppm error).

Lab Book Reference: TD 5/36

1-[(*tert*-Butoxy)carbonyl]-3-(4-fluorophenyl)piperidine-3-carboxylic acid S25

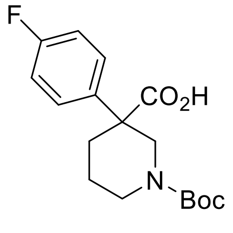

S25

KOH (476 mg, 8.47 mmol, 10.0 eq.) was added to a stirred solution of *N*-Boc aryl piperidine-3-methyl ester **4q** (250 mg, 0.74 mmol, 1.0 eq.) in EtOH (10 mL) at rt. The resulting mixture was stirred and heated at 100 °C for 16 h. The reaction mixture was allowed to cool to rt and evaporated under reduced pressure to give the crude product as an orange solid. The solid was taken up into water (20 mL) and washed with CH_2Cl_2 (3 × 10 mL). The aqueous layer was acidified with 1 M $HCl_{(aq)}$ (1 mL) and

extracted with CH_2Cl_2 (3 × 10 mL). The combined organics were washed with brine (20 mL), dried (Na_2SO_4) and evaporated under reduced pressure to give *N*-Boc aryl piperidine-3-carboxylic acid **S25** (226 mg, 95%) as an orange solid, mp 178-180 °C; IR (ATR) 2978, 1717 (C=O, CO_2H), 1663 (C=O, Boc), 1436, 1149, 646 cm^{-1}; ^1H NMR (400 MHz, $CDCl_3$) δ 7.43-7.40 (m, 2H, Ar), 7.03 (dd, *J* = 9.0, 9.0 Hz, 2H, Ar), 4.27 (m, 1H, NCH), 3.64-3.48 (m, 2H, NCH), 3.13 (m, 1H, NCH), 2.44-2.38 (m, 1H, CH), 2.04-2.00 (m, 1H, CH), 1.99-1.97 (m, 2H, CH), 1.44 (s, 9H, CMe_3); ^{13}C NMR (100.6 MHz, $CDCl_3$) δ 179.0 (C=O, CO_2H), 162.2 (d, *J* = 247.0 Hz, *ipso*-Ar), 154.8 (C=O, Boc), 135.1 (d, *J* = 3.5 Hz, *ipso*-Ar), 128.4 (d, *J* = 8.0 Hz, Ar), 115.6 (d, *J* = 21.0 Hz, Ar), 80.2 (O*C*Me$_3$), 50.5 (NCH_2), 49.6 (C), 43.1 (NCH_2), 32.6 (CH_2), 28.5 (C*Me$_3$*), 22.2 (CH_2); MS (ESI) *m/z* 346 $(M + Na)^+$; HRMS *m/z* calcd for $C_{17}H_{22}FNO_4$ $(M + Na)^+$ 346.1425, found 346.1458 (–4.8 ppm error).

Lab Book Reference: PJ-07-76.

tert-Butyl 3-carbamoyl-3-(4-fluorophenyl)piperidine-1-carboxylate S26

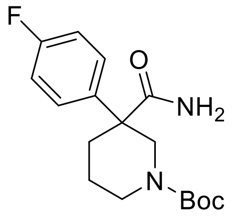

S26

T3P® (0.31 mL of a 50% wt. solution in EtOAc, 1.07 mmol, 1.7 eq.) was added dropwise to a stirred solution of *N*-Boc aryl piperidine-3-carboxylic acid **S25** (200 mg, 0.62 mmol, 1.0 eq.), 35% $NH_{3(aq)}$ (0.031 mL, 0.78 mmol, 1.2 eq.) and DIPEA (0.37 mL, 2.1 mmol, 3.4 eq.) in CH_2Cl_2 (5 mL) at rt under Ar. The resulting solution was stirred at rt for 16 h. The reaction mixture was poured into water (10 mL) and acidified using 1 M $HCl_{(aq)}$ (5 mL) was added. The two layers were separated, and the aqueous layer was extracted with EtOAc (3 × 10 mL). The combined organics were washed with 2 M $NaOH_{(aq)}$ (10 mL) and brine (10 mL), dried ($MgSO_4$) and evaporated under reduced pressure to give the crude product as an orange oil. Purification by flash column chromatography on silica with 99:1 CH_2Cl_2-MeOH as eluent gave *N*-Boc aryl piperidine-3-amide **S26** (76 mg, 38%) as an orange solid, mp 150-152 °C; R_F (99:1 CH_2Cl_2-MeOH) 0.23, IR (ATR) 2951, 1666 (C=O), 1626 (C=O), 1434, 1156, 830 cm^{-1}; ^1H NMR (400 MHz, $CDCl_3$) δ 7.44-7.40 (m, 2H, Ar), 7.03 (dd, *J* = 9.0, 9.0 Hz, 3H, Ar and NH), 5.36-5.19 (m, 1H, NH), 4.63-4.44 (m, 1H, NCH), 4.05-3.78 (m, 1H, NCH), 3.16-3.12 (m, 1H,

NCH), 3.03-2.86 (m, 1H, NCH), 2.85-2.74 (m, 1H, CH), 1.87-1.69 (m, 1H, CH), 1.64-1.59 (m, 2H, CH), 1.46 (s, 9H, CMe$_3$); ^{13}C NMR (100.6 MHz, CDCl$_3$) δ 175.9 (C=O, CONH$_2$), 162.1 (d, J = 247.0 Hz, *ipso*-Ar), 155.5 (C=O, Boc), 137.2 (*ipso*-Ar), 127.9 (Ar), 115.7 (d, J = 21.0 Hz, Ar), 80.8 (O*C*Me$_3$), 50.4 (C), 49.9 (NCH$_2$), 44.6 (NCH$_2$), 33.6 (CH$_2$), 28.5 (C*Me$_3$*), 23.2 (CH$_2$); MS (ESI) *m/z* 345 (M + Na)$^+$; HRMS *m/z* calcd for $C_{17}H_{23}FN_2O_3$ (M + Na)$^+$ 345.1585, found 345.1581 (+0.6 ppm error).

Lab Book Reference: PJ-07-79

3-(4-Fluorophenyl)piperidine-3-carboxamide hydrochloride 8am·HCl

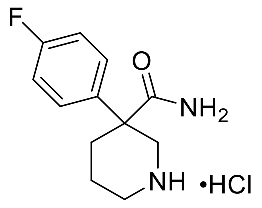

8am•HCl

Using general procedure G, *N*-Boc pyrrolidine **S26** (76 mg, 0.24 mmol, 1.0 eq.) and HCl (1 mL of a 4 M solution in dioxane) gave pyrrolidine **8am**•HCl (63 mg, 99%) as a cream solid, mp 230-231 °C; IR (ATR) 2954, 1650 (C=O, CONH$_2$), 1510, 1229, 1165, 814 cm^{-1}; ^{1}H NMR (400 MHz, MeOH-*d$_4$*) δ 7.47-7.43 (m, 2H, Ar), 7.15 (dd, J = 9.0, 9.0 Hz, 2H, Ar), 3.89 (dd, J = 12.5, 2.5 Hz, 1H, NCH), 3.37-3.30 (m, 1H, NCH), 2.98 (ddd, J = 12.5, 12.5, 3.5 Hz, 1H, NCH), 2.80 (d, J = 12.5 Hz, 1H, NCH), 2.57-2.53 (m, 1H, CH), 2.32 (ddd, J = 13.5, 13.5, 3.5 Hz, 1H, CH), 2.07 (ddddd, J = 15.0, 3.5, 3.5, 3.5, 3.5 Hz, 1H, CH), 1.92-1.83 (m, 1H, CH); ^{13}C NMR (100.6 MHz, MeOD-*d$_4$*) δ 178.1 (C=O, CONH$_2$), 163.9 (d, J = 246.5 Hz, *ipso*-Ar), 136.9 (d, J = 3.0 Hz, *ipso*-Ar), 129.4 (d, J = 8.0 Hz, Ar), 116.9 (d, J = 22.0 Hz, Ar), 51.9 (NCH$_2$), 48.4 (C), 44.5 (NCH$_2$), 30.6 (CH$_2$), 21.0 (CH$_2$); MS (ESI) *m/z* 223 (M)$^+$; HRMS *m/z* calcd for $C_{12}H_{16}FN_2O$ (M)$^+$ 223.1241, found 223.1240 (+1.3 ppm error).

Lab Book Reference: PJ-08-01.

1-[(*tert*-Butoxy)carbonyl]-2-[(4-fluorophenyl)methyl]pyrrolidine-2-carboxylic acid S27

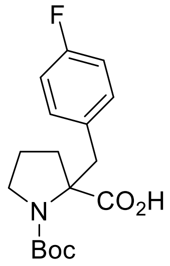

S27

KOH (90 mg, 1.6 mmol, 10.0 eq.) was added to a stirred solution of *N*-Boc aryl pyrrolidine-2-methyl ester **5d** (55 mg, 0.16 mmol, 1.0 eq.) in EtOH (10 mL) at rt. The resulting mixture was stirred and heated at 100 °C for 16 h. The reaction mixture was allowed to cool to rt and evaporated under reduced pressure to give the crude product as an orange solid. The solid was taken up into water (10 mL) and washed with CH_2Cl_2 (3 × 50 mL). The aqueous layer was then acidified with 1 M $HCl_{(aq)}$ (1 mL) and extracted with CH_2Cl_2 (3 × 10 mL). The combined organics were washed with brine (10 mL), dried (Na_2SO_4) and evaporated under reduced pressure to give *N*-Boc aryl pyrrolidine-2-carboxylic acid **S27** (50 mg, 98%) as a white solid, mp 178-180 °C; IR (ATR) 2984, 1739 (C=O, CO_2H), 1636 (C=O, Boc), 1419, 1219, 1148, 847, 770 cm^{-1}; ^1H NMR (400 MHz, MeOH-d_4) (60:40 mixture of rotamers) δ 7.18-7.14 (m, 2H, Ar), 7.04-6.98 (m, 2H, Ar), 3.68 (d, J = 14.0 Hz, 0.4H, CHAr), 3.52 (d, J = 14.0 Hz, 0.6H, CHAr), 3.46-3.39 (m, 1H, NCH), 3.04-3.00 (m, 1H, CHAr), 2.94-2.88 (m, 1H, NCH), 2.16-2.07 (m, 2H, CH), 1.67-1.61 (m, 1H, CH), 1.51 (s, 3.4H, C*Me*$_3$), 1.50 (s, 5.6H, C*Me*$_3$), 1.03-0.92 (m, 1H, CH); ^{13}C NMR (100.6 MHz, MeOD-d_4) (rotamers) δ 177.9 (C=O, CO_2H), 177.8 (C=O, CO_2H), 164.6 (d, J = 244.0 Hz, *ipso*-Ar), 162.1 (d, J = 244.0 Hz, *ipso*-Ar) 155.9 (C=O, Boc), 155.8 (C=O, Boc), 134.5 (d, J = 3.5 Hz, *ipso*-Ar), 134.4 (d, J = 3.5 Hz, *ipso*-Ar), 133.4 (d, J = 8.0 Hz, Ar), 133.3 (d, J = 8.0 Hz, Ar), 115.9 (d, J = 21.5 Hz, Ar), 115.7 (d, J = 21.5 Hz, Ar), 82.2 (O*C*Me$_3$), 81.1 (O*C*Me$_3$), 69.5 (C), 69.5 (C), 49.7 (NCH_2), 49.6 (NCH_2), 39.8 (CH_2Ar), 38.5 (CH_2Ar), 37.8 (CH_2), 36.7 (CH_2), 28.9 (C*Me*$_3$), 28.7 (C*Me*$_3$), 23.7 (CH_2), 23.2 (CH_2); MS (ESI) *m/z* 346 $(M + Na)^+$; HRMS *m/z* calcd for $C_{17}H_{22}FNO_4$ $(M + Na)^+$ 346.1425, found 346.1420 (+1.5 ppm error).

Lab Book Reference: PJ-07-44.

***tert*-Butyl 2-carbamoyl-2-[(4-fluorophenyl)methyl]pyrrolidine-1-carboxylate S28**

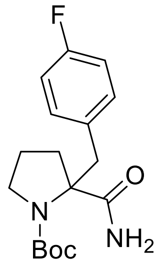

S28

T3P® (0.07 mL of a 50% wt solution in EtOAc, 0.23 mmol, 1.5 eq.) was added dropwise to a stirred solution of *N*-Boc aryl pyrrolidine-2-carboxylic acid **S27** (50 mg, 0.15 mmol, 1.0 eq.), DIPEA (0.08 mL, 0.46 mmol, 3.0 eq.) and 35% $NH_{3(aq)}$ (0.018 mL, 0.47 mmol, 3.0 eq.) in CH_2Cl_3 (3 mL) at rt under Ar. The resulting solution was stirred at rt for 16 h. Then, water (10 mL) was added and the layers were separated. The aqueous layer was extracted with CH_2Cl_2 (3 × 5 mL) and the combined organics were washed with 2 M $NaOH_{(aq)}$ (10 mL) and brine (10 mL), dried (Na_2SO_4) and evaporated under redued pressure to give the crude product as a colourless oil. Purification by flash column chromatography on silica with EtOAc as eluent gave *N*-Boc aryl pyrrolidine amide **S28** (21 mg, 45%) as a colourless oil, R_F (EtOAc) 0.36; IR (ATR) 3381 (NH), 2982, 1658 (C=O), 1509, 1395, 1159 cm^{-1}; ^{1}H NMR (400 MHz, $CDCl_3$) (60:40 mixture of rotamers) δ 7.46 (br s, 0.6H, NH), 7.14-7.07 (m, 2H, Ar), 7.00-6.93 (m, 2H, Ar), 5.92 (br s, 0.4H, NH). 5.61 (br s, 1H, NH), 3.71 (d, *J* = 14.0 Hz, 0.6H, CHAr), 3.55 (d, *J* = 14.0 Hz, 0.4H, CHAr), 3.48-3.43 (m, 0.4H, NCH), 3.40 (ddd, *J* = 11.0, 8.0, 3.0 Hz, 0.6H, NCH), 3.16 (d, *J* = 14.0 Hz, 0.4H, CHAr), 3.10 (d, *J* = 14.0 Hz, 0.6H, CHAr), 3.00-2.89 (m, 1H, NCH), 2.44 (ddd, *J* = 12.5, 6.5, 3.5 Hz, 0.6H, CH), 2.16-2.13 (m, 0.6H, CH), 1.81 (ddd, *J* = 13.0, 11.0, 7.0 Hz, 0.6H, CH), 1.61-1.56 (m, 1.2H, CH), 1.54 (s, 3.6H, C*Me*$_3$), 1.53 (s, 5.4H, C*Me*$_3$), 1.38-1.33 (m, 0.6H, CH), 1.04-0.94 (m, 0.4H, CH); ^{13}C NMR (100.6 MHz, $CDCl_3$) (rotamers) δ 177.8 (C=O, CONH), 177.0 (C=O, CONH), 161.9 (d, *J* = 245.0 Hz, *ipso*-Ar), 155.5 (C=O, Boc), 153.7 (C=O, Boc), 133.0 (d, *J* = 3.0 Hz, *ipso*-Ar), 132.4 (d, *J* = 3.0 Hz, *ipso*-Ar), 132.1 (d, *J* = 8.0 Hz, Ar), 131.9 (d, *J* = 8.0 Hz, Ar), 115.5 (d, *J* = 21.0 Hz, Ar), 115.1 (d, *J* = 21.0 Hz, Ar), 81.5 (O*C*Me$_3$), 80.7 (O*C*Me$_3$), 70.8 (C), 69.2 (C), 49.6 (NCH_2), 48.5 (NCH_2), 38.0 (CH_2Ar), 37.9 (CH_2Ar), 37.5 (CH_2), 34.3 (CH_2), 28.6 (C*Me*$_3$), 22.2 (C*Me*$_3$), 22.0 (CH_2); MS (ESI) *m/z* 345 (M + Na)$^+$; HRMS *m/z* calcd for $C_{17}H_{23}FN_2O_3$ (M + Na)$^+$ 345.1585, found 345.1581 (+1.0 ppm error).

Lab Book Reference: PJ-07-61.

2-[(4-Fluorophenyl)methyl]pyrrolidine-2-carboxamide hydrochloride 8an·HCl

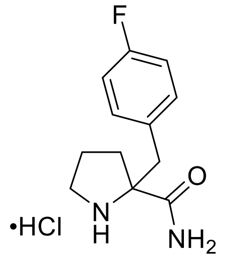

8an•HCl

Using general procedure G, *N*-Boc pyrrolidine **S28** (19 mg, 0.06 mmol, 1.0 eq.) and HCl (1 mL of a 4 M solution in dioxane) gave pyrrolidine **8an**•HCl (13 mg, 85%) as a white solid, mp 242–243 °C; IR (ATR) 2732, 2425, 1677 (C=O), 1605, 1511, 1459, 1418, 1224, 1162, 842 cm^{-1}; ^1H NMR (400 MHz, MeOD-*d*4) δ 7.38–7.34 (m, 2H, Ar), 7.10–7.06 (m, 2H, Ar), 3.54 (d, J = 15.0 Hz, 1H, CH_2Ar), 3.46–3.39 (m, 1H, NCH_2), 3.35–3.29 (m, 1H, NCH_2), 3.25 (d, J = 15.0 Hz, 1H, CH_2Ar), 2.59–2.53 (m, 1H, CH_2), 2.23–2.15 (m, 2H, CH_2), 2.00–1.93 (m, 1H, CH_2); ^{13}C NMR (101 MHz, MeOD-*d*4) δ 172.7 (C=O), 163.9 (d, J = 245.0 Hz, *ipso*-Ar), 132.5 (d, J = 8.0 Hz, Ar), 131.2 (*ipso*-Ar), 116.6 (d, J = 22.0 Hz, Ar), 75.6 (NC), 46.5 (CH_2), 40.8 (CH_2), 36.7 (CH_2), 23.3 (CH_2); HRMS (ESI) *m/z* calcd for $C_{12}H_{16}FN_2O$ M^+ 223.1241, found 223.1240 (+0.6 ppm error).

Lab Book Reference: JDF_B_397_9

2',4'-Dihydro-1'H-spiro[oxane-4,3'-quinolin]-2'-one 8ao

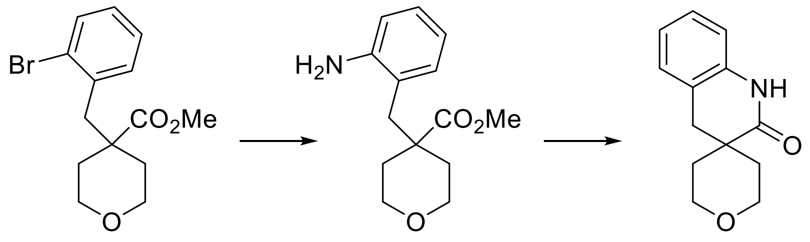

5c **S29** **8ao**

$Pd(OAc)_2$ (3.5 mg, 0.016 mmol, 0.05 eq.) was added to a stirred solution of aryl THP-4-methyl ester **5c** (100 mg, 0.31 mmol, 1.0 eq.), benzophenone imine (0.08 mL, 0.46 mmol, 1.5 eq.), Cs_2CO_3 (304 mg, 0.93 mmol, 3.0 eq.) and Xantphos (18 mg, 0.031 mmol) in dioxane (6 mL) at rt. The reaction flask was evacuated under reduced pressure and back-filled with Ar three times. The resulting mixture was stirred and heated at 95 °C for 16 h. The reaction mixture was allowed to cool to rt and 1 M $HCl_{(aq)}$ (3 mL) was added. The resulting solution was stirred at rt for 10 min. 2 M $NaOH_{(aq)}$ (1 mL) was then added and the two the layers were separated. The aqueous layer was extracted with CH_2Cl_2 (3 × 10 mL) and the

combined organics were dried (Na_2CO_3) and evaporated under reduced pressure to give the crude product as a black oil. Purification by flash column chromatography on silica with 80:20-60:40 hexane-EtOAc as eluent gave a 65:35 mixture (by ^{1}H NMR spectroscopy) of spirocycle **8ao** and aniline **S29** (66 mg) as a white solid. The solid was taken up into MeOH (5 mL) and the resulting solution was stirred and heated at 50 °C for 16 h. The reaction mixture was allowed to cool to rt and evaporated under reduced pressure to afford spirocycle THP **8ao** (61 mg, 91%) as a white solid, mp 188-190 °C; R_F (80:20 hexane-EtOAc) 0.04; IR (ATR) 3058 (NH), 2984, 1662 (C=O), 1490, 1239, 747, 486 cm^{-1}; ^{1}H NMR (400 MHz, $CDCl_3$) δ 8.44 (s, 1H, NH), 7.21-7.16 (m, 2H, Ar), 7.01-6.98 (m, 1H, Ar), 6.79-6.77 (m, 1H, Ar), 3.90 (ddd, J = 11.5, 7.5, 3.5 Hz, 2H, OCH), 3.74 (ddd, J = 11.5, 7.0, 4.0 Hz, 2H, OCH), 2.92 (s, 2H, CH_2Ar), 2.01 (ddd, J = 14.0, 7.0, 4.0 Hz, 2H, CH), 1.47 (ddd, J = 14.0, 7.5, 3.5 Hz, 1H); ^{13}C NMR (100.6 MHz, $CDCl_3$) δ 175.4 (C=O), 136.6 (*ipso*-Ar), 128.7 (Ar), 127.8 (Ar), 123.3 (Ar), 122.3 (*ipso*-Ar), 114.8 (Ar), 64.0 (OCH_2), 37.8 (C), 37.4 (CH_2Ar), 32.2 (CH_2); MS (ESI) *m/z* 240 (M + Na)$^+$; HRMS *m/z* calcd for $C_{13}H_{15}NO_2$ (M + Na)$^+$ 240.0995, found 240.0996 (–1.6 ppm error).
Lab Book Reference: PJ-08-14.

1-*tert*-Butyl 2-methyl 2-[(2-aminophenyl)methyl]pyrrolidine-1,2-dicarboxylate S30

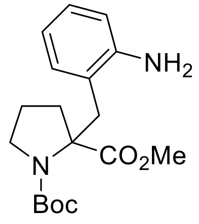

S30

$Pd(OAc)_2$ (2.8 mg, 0.013 mmol, 0.05 eq.) was added to a stirred solution of *N*-Boc aryl pyrrolidine-3-methyl ester **5g** (100 mg, 0.25 mmol, 1.0 eq.), benzophenone imine (0.063 mL, 0.38 mmol, 1.5 eq.), Cs_2CO_3 (246 mg, 0.76 mmol, 3.0 eq.) and Xantphos (14.5 mg, 0.025 mmol) in dioxane (6 mL) at rt. The reaction flask was evacuated under reduced pressure and back-filled with Ar three times. The resulting mixture was stirred and heated at 95 °C for 16 h. The reaction mixture was allowed to cool to rt and 1 M $HCl_{(aq)}$ (3 mL) was added. The resulting solution was stirred at rt for 10 min. 2 M $NaOH_{(aq)}$ (1 mL) was then added and the two layers were separated. The aqueous layer was extracted with CH_2Cl_2 (3 × 10 mL) and the combined organics were dried (Na_2CO_3) and evaporated under reduced pressure to give the crude product as a black oil. Purification by flash column chromatography on silica with 80:20 hexane-EtOAc as eluent gave aniline *N*-Boc pyrrolidine-2-methyl ester **S30** (73 mg, 87%) as a yellow solid, mp 80-82 °C; R_F (80:20 hexane-EtOAc) 0.11; IR (ATR) 3488 (NH), 3371 (NH). 2976, 1741 (C=O, CO_2Me), 1692

(C=O, Boc), 1395, 1250, 1121, 748 cm^{-1}; ^{1}H NMR (400 MHz, $CDCl_3$) (60:40 mixture of rotamers) δ 7.04-7.01 (m, 1H, Ar), 6.99-6.94 (m, 1H, Ar), 6.71-6.61 (m, 2H, Ar), 3.75 (s, 1.8H, OMe), 3.74 (s, 1.2H, OMe), 3.63 (d, J = 14.5 Hz, 0.4H, CHAr), 3.51 (ddd, J = 10.5, 7.5, 7.5 Hz, 0.6H, NCH), 3.42-3.36 (m, 1H, CHAr and NCH), 3.20 (ddd, J = 10.5, 7.5, 4.5 Hz, 0.6H, NCH), 3.12-3.04 (m, 1.4H, CHAr and NCH), 2.24-2.18 (m, 1H, CH), 2.07-2.00 (m, 1H, CH), 1.71-1.64 (m, 1H, CH), 1.47 (s, 3.6H, C*Me*3), 1.43 (s, 5.4H, C*Me*3), 1.26-1.19 (m, 1H, CH); ^{13}C NMR (100.6 MHz, $CDCl_3$) (rotamers) δ 175.7 (C=O, CO_2Me), 175.5 (C=O, CO_2Me), 155.3 (C=O, Boc), 153.7 (C=O, Boc), 146.5 (*ipso*-Ar), 145.9 (*ipso*-Ar), 132.8 (Ar), 132.7 (Ar), 128.0 (Ar), 127.8 (Ar), 121.5 (*ipso*-Ar), 121.1 (*ipso*-Ar), 118.7 (Ar), 118.0 (Ar), 116.1 (Ar), 115.8 (Ar), 80.7 (O*C*Me3), 80.0 (O*C*Me3), 69.2 (C), 68.9 (C), 52.5 (OMe), 52.4 (OMe), 48.4 (NCH_2), 37.1 (CH_2), 35.8 (CH_2), 35.3 (CH_2Ar), 34.5 (CH_2Ar), 28.5 (C*Me*3), 28.4 (C*Me*3), 23.3 (CH_2), 22.7 (CH_2); MS (ESI) *m/z* 335 $(M + H)^+$; HRMS *m/z* calcd for $C_{18}H_{26}N_2O_4$ $(M + H)^+$ 335.1965, found 335.1973 (–2.1 ppm error).
Lab Book Reference: PJ-08-10.

2',4'-Dihydro-1'H-spiro[pyrrolidine-2,3'-quinolin]-2'-one 8ap

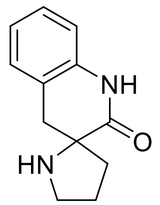

8ap

p-TsOH (36 mg, 0.21 mmol, 1.0 eq.) was added to a stirred solution of aniline *N*-Boc pyrrolidine-2-methyl ester **S30** (70 mg, 0.21 mmol, 1.0 eq.) in EtOH (5 mL) at rt. The resulting solution was stirred and heated at 80 °C for 16 h. The reaction mixture was allowed to cool to rt and evaporated under reduced pressure to give an orange oil. Sat $NaHCO_{3(aq)}$ (10 mL) was added and the mixture was extracted with EtOAc (3 × 10 mL). The combined organics were dried (Na_2SO_4) and evaporated under reduced pressure to give the crude product as an orange oil. Purification by flash column chromatography on silica with 95:5 CH_2Cl_2-MeOH as eluent gave 5,6-spirocycle **8ap** (31 mg, 74%) as a cream soild, mp 116-118 °C, R_F (95:5 CH_2Cl_2-MeOH) 0.11; IR (ATR) 3057 (NH), 2927, 1668 (C=O), 1492, 1369, 750 cm^{-1}; ^{1}H NMR (400 MHz, $CDCl_3$) δ 9.04 (br s, 1H, NH) 7.19-7.13 (m, 2H, Ar), 6.98 (ddd, J = 7.5, 7.5, 1.0 Hz, 1H, Ar), 6.83 (dd, J = 7.5, 1.0 Hz, 1H, Ar), 3.28-3.22 (m, 1H, NCH), 3.10-3.00 (m, 2H, NCH and CHAr), 2.86 (d, J = 15.5 Hz, 1H, CHAr), 1.89-1.73 (m, 4H, CH). ^{13}C NMR (100.6 MHz, $CDCl_3$) δ 175.8 (C=O), 136.8 (*ipso*-Ar), 128.7 (Ar), 127.7 (Ar), 123.8 (*ipso*-Ar), 123.2 (Ar), 115.3 (Ar), 63.9 (C), 47.7 (NCH_2), 39.8

(CH_2Ar), 35.5 (CH_2), 26.2 (CH_2); MS (ESI) *m/z* 203 $(M + H)^+$; HRMS *m/z* calcd for $C_{12}H_{14}N_2O$ (M + H)$^+$ 203.1179, found 203.1179 (–0.4 ppm error).

Lab Book Reference: PJ-08-13.

2. Library Analysis

2.1. General

Shape analysis

3-Dimensional structures were generated Pipeline Pilot 16.5.0.143, 2016, Accelrys Software Inc.

Prior to conformer generation a wash step was performed, which involved stripping salts and ionising the molecule at pH 7.4. SMILES strings were converted to their canonical representation and the original stereochemistry at each chiral centre was recorded. Any stereocentre created during the ionisation would have undefined stereochemistry. A SMILES files was written that contained all possible stereoisomers of the molecule. Conformers were generated using Catalyst with the BEST conformational analysis method and relative stereochemistry. Catalyst was run directly on the server and not through the built-in Conformation Generator component. The maximum relative energy threshold was left at the default 20 kcal mol^{-1} and a maximum of 255 conformers were generated for each compound. The aim of this was to give the best possible coverage of conformational space. The resulting conformations from Catalyst were read and only those where the stereochemistry matched the original molecule or its enantiomer were kept. These were then all standardised to the original stereochemistry by mirroring the coordinates of the enantiomers. Duplicate conformations were filtered with a RMSD threshold of 0.1. Each conformation was minimised using 200 steps of Conjugate Gradient minimisation with an RMS gradient tolerance of 0.1. This was performed using the CHARMm forcefield with Momany-Rone partial charge estimation and a Generalised Born implicit solvent model. After minimisation, duplicates were filtered again with a RMSD threshold of 0.1.

Generated conformations were used to generate the three Principal Moments of Inertia (I1, I2 and I3) which were then normalised by dividing the two lower values by the largest (I1/I3 and I2/I3) using Pipeline Pilot built-in components.

Principal moments of inertia (PMI) about the principal axes of a molecule were calculated according to the following rules:

1. The moments of inertia are computed for a series of straight lines through the centre of mass.

2. Distances are established along each line proportional to the reciprocal of the square root of I on either side of the centre of mass. The locus of these distances forms an ellipsoidal surface. The principal moments are associated with the principal axes of the ellipsoid.

Cumulative PMI analysis was performed in the following way. The ΣNPR (ΣNPR = NPR1 + NPR2) was calculated for each conformer and then the mean ΣNPR for each fragment was obtained. This value was used as a measure of the three-dimentionality of each fragment. The cumulative percentage of fragments within a defined distance from the rod-disc axis (ΣNPR) was calculated and plotted.

Molecular properties

Molecular Weight (MW), heavy atom count (HAC), lipophilicity (SlogP), number of hydrogen bond donors (HBD), number of hydrogen bond acceptors (HBA), rotatable bond count (RBC), fraction of sp^3 carbons (Fsp^3) and topological polar surface area (TPSA) were calculated using RDKit v3.4 in KNIME v3.5.2. Prior to calculation, salts were stripped and canonical SMILES were generated. ClogP values were caluculated using Daylight/BioByte ClogP v4.3.

Solubility

Fragment solubility was assessed in a 20 mM sodium phosphate buffer (pH 7.48) (1 mM concentration of fragment). Fragment solubility was determined by ^1H NMR spectroscopy with the aid of a reference compound, 4,4-dimethyl-4-silapentane-1-sulfonic acid (DSS), of known concentration. Integrals of the fragment were compared with those of DSS and the fragment concentration was calculated using the following formula: $C_x = I_x/I_{cal} \times N_{cal}/N_x \times C_{cal}$ where I, N, and C are the integral area, number of nuclei, and concentration of the fragment (x) and the calibrant (cal), respectively.

Stability

Fragment stability in solution was assessed in both a 20 mM sodium phosphate buffer (pH 7.48) (1 mM concentration of fragment) at 1 h and 24 h and in DMSO (2 mM concentration of fragment) at 24 h and 6 weeks using ^1H NMR spectroscopy.

2.2 Virtual Library Enumeration

A virtual library of 504 compounds was enumerated as shown in Figure S1. 336 fragments were derived from 12 heterocyclic cores, four aryl rings, and seven R groups. 168 fragments were derived from six heterocyclic cores, four aryl rings, and seven R groups.

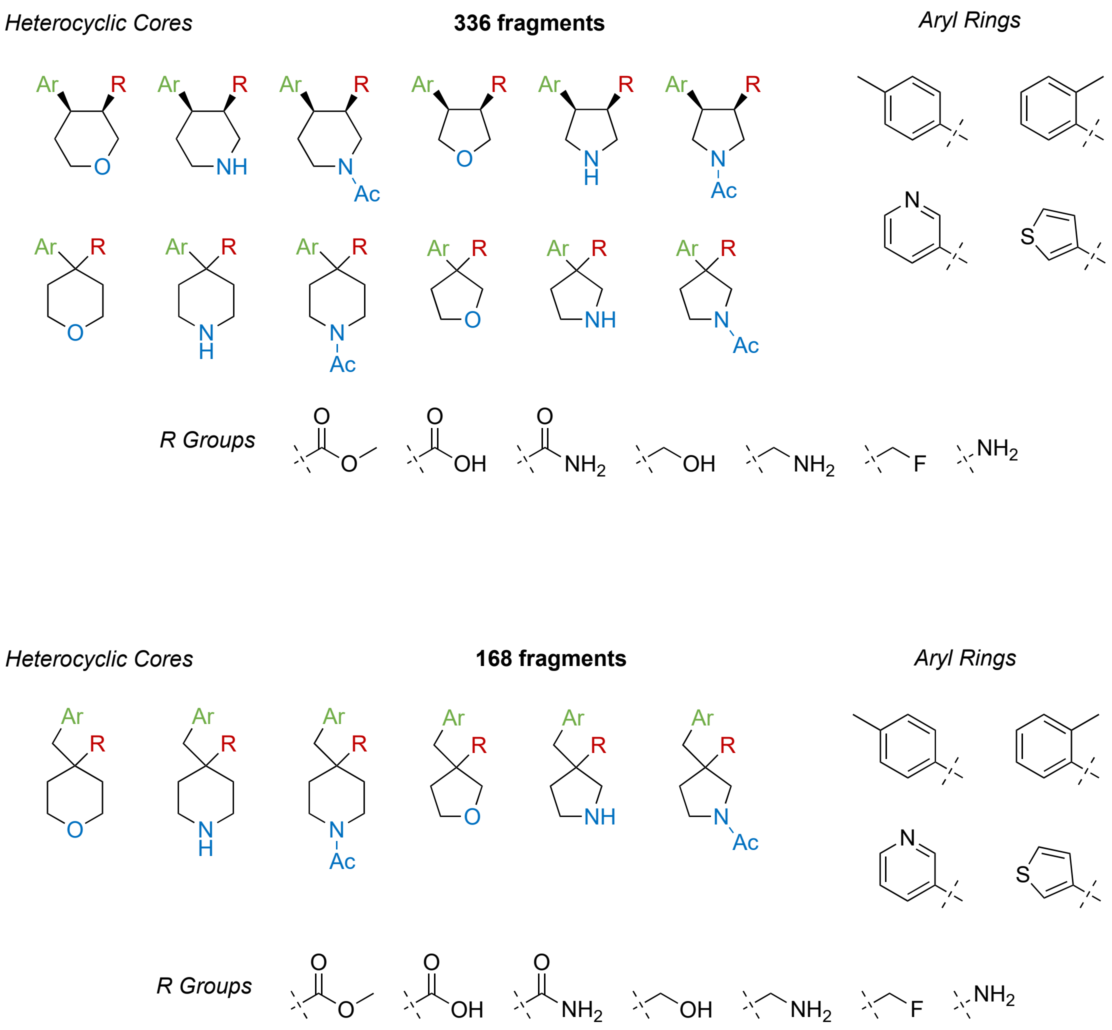

Figure S1

2.3 Shape Analysis of Virtual Library of *trans*-Isomers of 2

Figure S2 shows a PMI plot of the *trans*-isomers of potential fragments derived from **2**. These compounds are significantly less 3-D than the *cis*-isomers **2** (see Figure 1C)

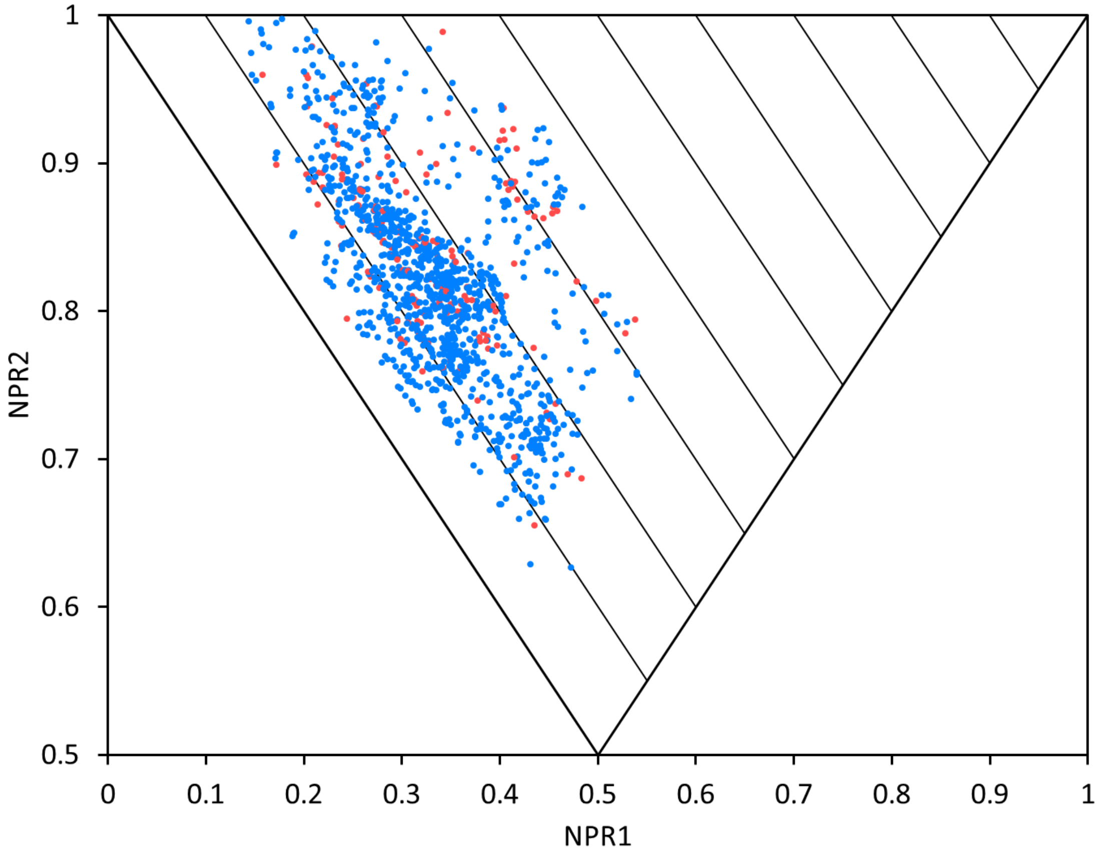

Figure S2. PMI analysis of potential fragments *trans*-**2.** Red dots indicate ground state conformers and blue dots higher energy conformers

2.4 Commercial Fragment Library Details

The following commercially available fragment libraries were analysed:

Maybridge RO3 Diversity Fragment Library: Core Set (1000 compounds, https://www.maybridge.com/portal/alias__Rainbow/lang__en/tabID__230/DesktopDefault.aspx; last accessed 18/10/2017)

Chembridge Fragment Library (9067 compounds, https://www.chembridge.com/screening_libraries/fragment_library/; last accessed 23/04/2018)

Enamine RO3 Fragment Library (69583 compounds, https://enamine.net/index.php?option=com_content&task=view&id=208; last accessed 19/03/2018)

Life Chemicals 3D Fragment Library (1377 compounds, https://lifechemicals.com/screening-libraries/fragment-libraries; last accessed 21/8/2018)

ChemDiv 3D FL Fragments Library (4460 compounds, http://www.chemdiv.com/3d-fl-fragments-library/; last accessed 10/01/2019)

Enamine 3D Shape Diverse Fragment Library (1200 compounds, https://enamine.net/index.php?option=com_content&view=article&id=640&Itemid=716; last accessed 10/01/2019)

2.5 Structures and Smiles Files of the 58 3-D Fragments

The structures of the 58 3-D fragments are shown in Figure S3 and the Smiles files are shown in Table S1.

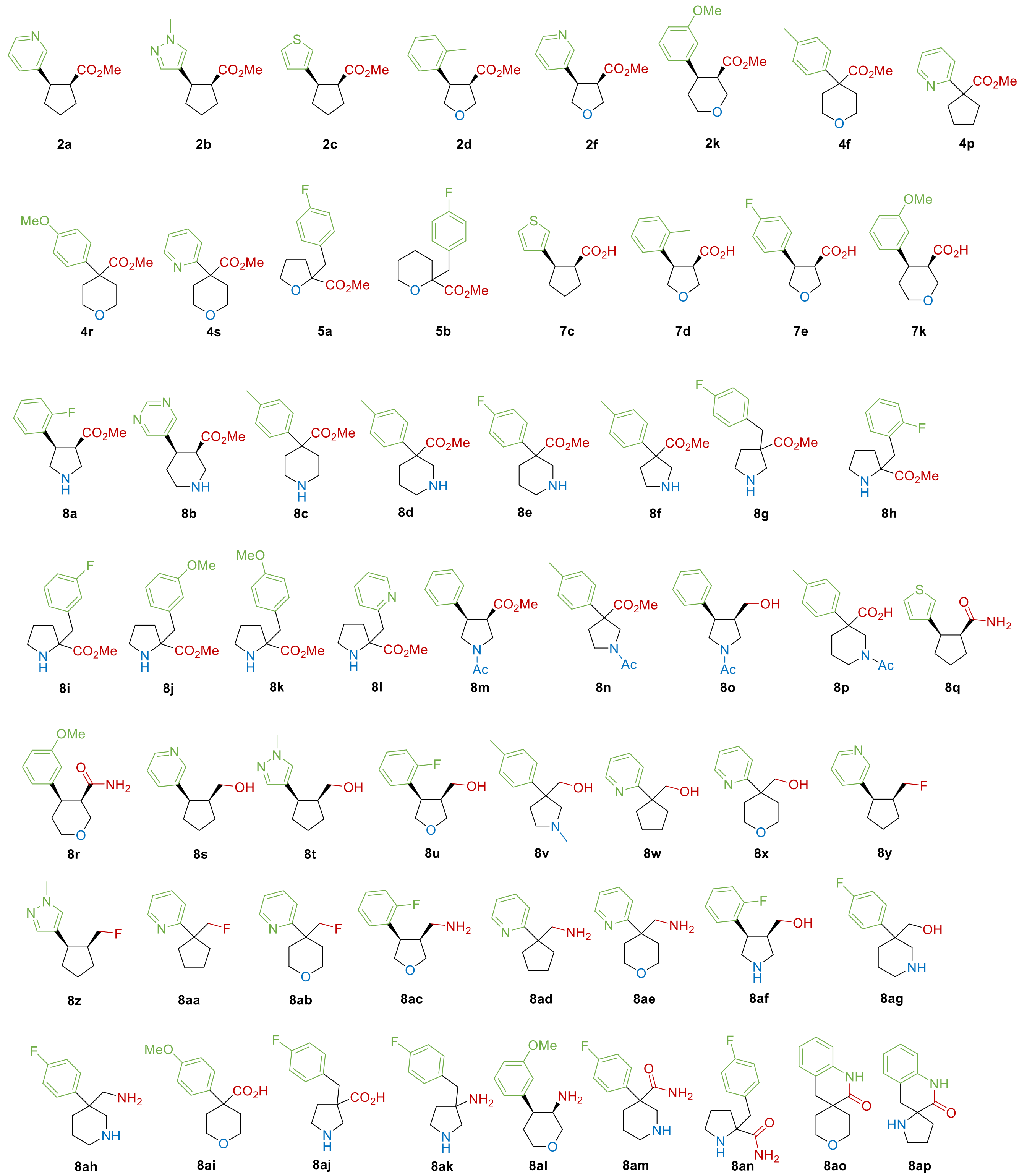

Figure S3.

Table S1: Smiles files for the 58 3-D fragments.

**2a**	O=C([C@@H]1[C@H](C2=CN=CC=C2)CCC1)OC
**2b**	CN(N=C1)C=C1[C@H]2[C@@H](C(OC)=O)CCC2
**2c**	O=C([C@@H]1[C@H](C2=CSC=C2)CCC1)OC
**2d**	CC(C=CC=C1)=C1[C@H]2[C@@H](C(OC)=O)COC2
**2f**	O=C([C@@H]1[C@H](C2=CN=CC=C2)COC1)OC
**2k**	COC1=CC([C@H]2[C@@H](C(OC)=O)COCC2)=CC=C1
**4f**	O=C(OC)C1(C2=CC=C(C)C=C2)CCOCC1
**4p**	O=C(OC)C1(C2=NC=CC=C2)CCCC1
**4r**	O=C(OC)C1(C2=CC=C(OC)C=C2)CCOCC1
**4s**	O=C(OC)C1(C2=CC=CC=N2)CCOCC1
**5a**	FC(C=C1)=CC=C1CC2(C(OC)=O)OCCC2
**5b**	FC(C=C1)=CC=C1CC2(CCCCO2)C(OC)=O
**7c**	O=C([C@@H]1[C@H](C2=CSC=C2)CCC1)O
**7d**	CC(C=CC=C1)=C1[C@H]2[C@@H](C(O)=O)COC2
**7e**	FC(C=C1)=CC=C1[C@H]2[C@@H](C(O)=O)COC2
**7k**	COC1=CC([C@H]2[C@@H](C(O)=O)COCC2)=CC=C1
**8a**	FC1=CC=CC=C1[C@H]2[C@@H](C(OC)=O)CNC2
**8b**	O=C([C@H]1CNCC[C@H]1C2=CN=CN=C2)OC
**8c**	CC(C=C1)=CC=C1C2(C(OC)=O)CCNCC2
**8d**	CC(C=C1)=CC=C1C2(C(OC)=O)CNCCC2
**8e**	O=C(OC)C1(C2=CC=C(F)C=C2)CNCCC1
**8f**	CC(C=C1)=CC=C1C2(C(OC)=O)CNCC2
**8g**	O=C(OC)C1(CC2=CC=C(F)C=C2)CNCC1
**8h**	O=C(OC)C1(CC2=CC=CC=C2F)NCCC1
**8i**	O=C(OC)C1(CC2=CC=CC(F)=C2)NCCC1
**8j**	O=C(OC)C1(CC2=CC=CC(OC)=C2)NCCC1
**8k**	O=C(OC)C1(CC2=CC=C(OC)C=C2)NCCC1
**8l**	O=C(OC)C1(CC2=CC=CC=N2)NCCC1
**8m**	O=C(N1C[C@H](C(OC)=O)[C@H](C2=CC=CC=C2)C1)C
**8n**	CC(C=C1)=CC=C1C2(C(OC)=O)CN(C(C)=O)CC2
**8o**	OC[C@@H]1[C@H](C2=CC=CC=C2)CN(C(C)=O)C1
**8p**	OC(C1(C2=CC=C(C)C=C2)CN(C(C)=O)CCC1)=O
**8q**	O=C([C@@H]1[C@H](C2=CSC=C2)CCC1)N
**8r**	COC1=CC([C@H]2[C@@H](C(N)=O)COCC2)=CC=C1
**8s**	c1cc(cnc1)[C@@H]2CCC[C@@H]2CO
**8t**	OC[C@@H]1[C@H](C2=CN(C)N=C2)CCC1
**8u**	OC[C@@H]1[C@H](C2=CC=CC=C2F)COC1
**8v**	OCC1(C2=CC=C(C)C=C2)CN(C)CC1
**8w**	OCC1(C2=NC=CC=C2)CCCC1

**8x**	OCC1(C2=CC=CC=N2)CCOCC1
**8y**	FC[C@@H]1[C@H](C2=CN=CC=C2)CCC1
**8z**	FC[C@@H]1[C@H](C2=CN(C)N=C2)CCC1
**8aa**	FCC1(C2=NC=CC=C2)CCCC1
**8ab**	FCC1(C2=CC=CC=N2)CCOCC1
**8ac**	NC[C@@H]1[C@H](C2=CC=CC=C2F)COC1
**8ad**	NCC1(C2=NC=CC=C2)CCCC1
**8ae**	NCC1(C2=CC=CC=N2)CCOCC1
**8af**	OC[C@@H]1[C@H](C2=CC=CC=C2F)CNC1
**8ag**	OCC1(C2=CC=C(F)C=C2)CNCCC1
**8ah**	FC(C=C1)=CC=C1C2(CN)CNCCC2
**8ai**	O=C(O)C1(C2=CC=C(OC)C=C2)CCOCC1
**8aj**	OC(C1(CC2=CC=C(F)C=C2)CNCC1)=O
**8ak**	NC1(CC2=CC=C(F)C=C2)CNCC1
**8al**	N[C@H]1COCC[C@H]1C2=CC=CC(OC)=C2
**8am**	O=C(N)C1(C2=CC=C(F)C=C2)CNCCC1
**8an**	FC(C=C1)=CC=C1CC2(C(N)=O)NCCC2
**8ao**	O=C(NC(C=CC=C1)=C1C2)C32CCOCC3
**8ap**	O=C1NC2=C(C=CC=C2)CC31NCCC3

2.6 Yields for the 42 3-D Fragments Synthesised from Building Blocks (Scheme 4)

The number of steps and yields for each of the 42 3-D fragments whose synthesis starting from building blocks **2**, **4**, **5** and **7** is shown in Scheme 4 are reported in Figure S4.

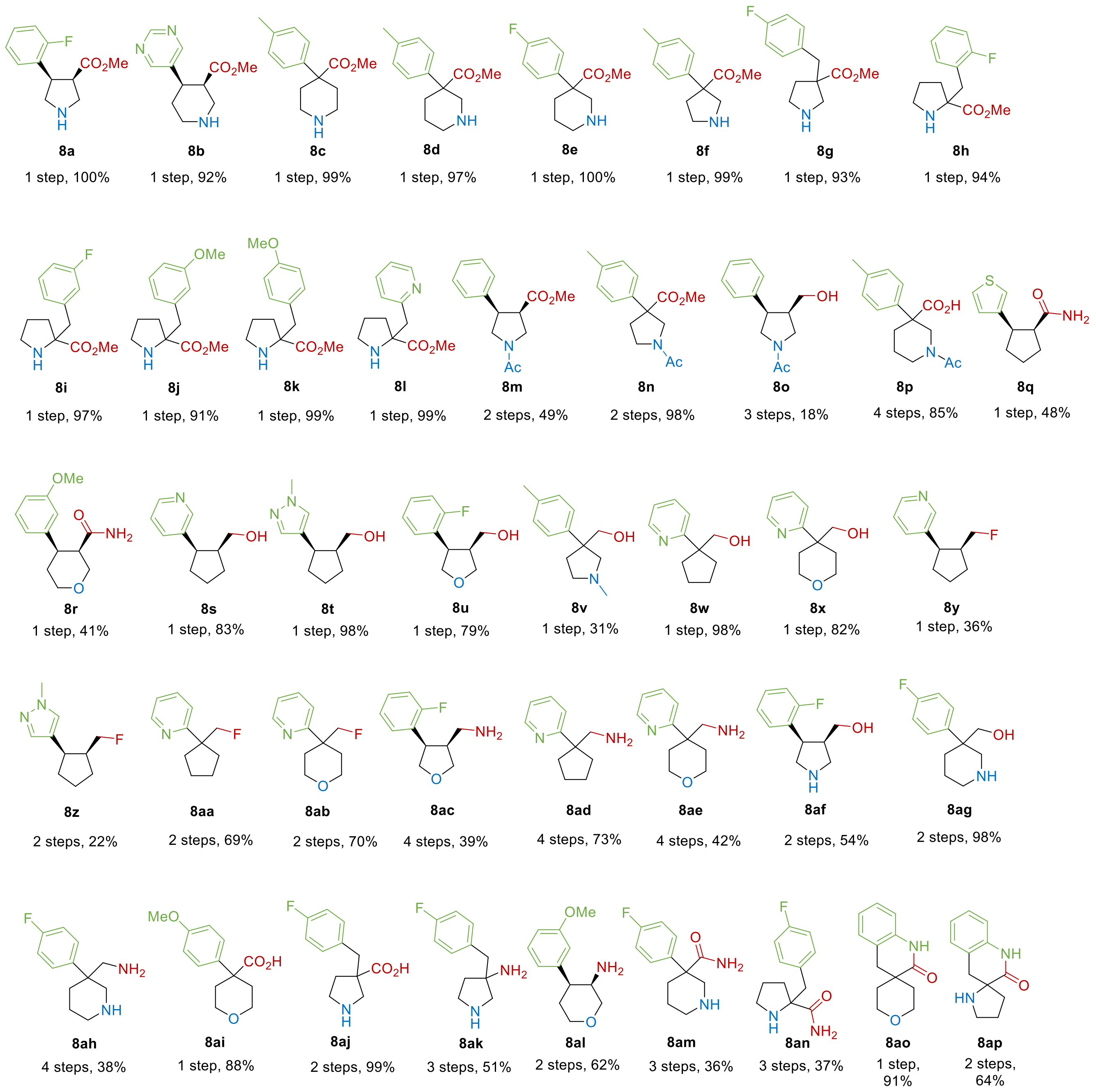

Figure S4

3. MGATV Methods

3.1 Protein production, Crystallization and Complex Generation

MGATV protein was expressed using an insect cell/baculovirus system, and purified as previously described.[12] Concentrated MGATV protein stocks (10 mg.mL^{-1}), in 20 mM HEPES (pH 7.4), 200 mM NaCl, 1 mM DTT buffer were used for crystallization. MGATV crystals were grown using the sitting drop vapour-diffusion method in Swissci 48-well “Maxi” plates, using 0.1 M HEPES pH 8.0, 0.3 M Li_2SO_4, 30 % (w/v) PEG 3350, 10 % (v/v) ethylene glycol, and typical protein:well solution ratios of 500:500 nL.

Fragment complexes were generated by applying concentrated stock solutions of fragment to drops containing unliganded MGATV crystals, to produce a final concentration of ~25 mM fragment. Crystals were typically soaked for ~3 h, before harvesting and flash cooling in liquid N_2 for data collection.

3.2 Data Collection and Processing

Diffraction data were collected at the Diamond Light Source (Harwell, UK). Diffraction images were autoprocessed using the xia2 3dii pipeline8, and solved by directly refining against the structure of unliganded MGATV (PDB accession 6YJQ). The solved **8p** complex structure was improved by iterative rounds of manual model building and refinement using COOT11 and REFMAC, respectively. Ligand co-ordinates were built using jLigand13. Refinement statistics for the complex are presented in Table S2.

Structures of SARS-CoV-2 Mac1 in complex with **7c** (5S3T) and **7k** (5S3X) were solved as part of a previous study,[13] using the PanDDA analysis method for detection of weaker binding ligands.[14]

All structure figures were prepared using Pymol.

Table S2: Refinement statistics for the MGATV **8p** complex. Values in parentheses are for highest-resolution shell.

	MGATV-**8p** (PDB 8CE3)
**Data collection**	
Space group	P1
a, *b*, *c* (Å)	46.86, 68.54, 91.31
α, β, γ (°)	107.3, 92.16, 107.05
Resolution (Å)	60.47–1.89 (1.92–1.89)
R_{meas}	0.047 (1.297)
$I / \sigma I$	13.6 (1.0)
Completeness (%)	97.3 (96.5)
Redundancy	3.5 (3.4)
$CC_{1/2}$	1.0 (0.6)
**Refinement**	
Unique reflections	80017
R_{work} / R_{free}	0.19/0.23
No. atoms	
Protein	8033
Ligand/ion	112
Water	349
B-factors	
Protein	57.4
Ligand/ion	83.5
Water	54.0
R.m.s. deviations	
Bond lengths (Å)	0.007
Bond angles (°)	1.41

3.3 Thermal Shift Assays

Assays were carried out in 96-well PCR plates (Agilent 401334) or 8-well PCR strips. Samples contained SYPRO-orange dye (2X), MGATV (final concentration of 4 µM), and compounds from the 3D fragment libraries at 8 mM or 4 mM or the equivalent quantity of DMSO. Samples were made up to 25 µL with buffer (50 mM MES, pH 6.5, 25 mM NaCl, 1 mM DTT). 5 mM UDP-GlcNAc donor substrate (Promega) was used as a control. The assay was run using an Agilent Stratagene Mx3005P rtPCR machine heated from 25 °C to 95 °C in increments of +1 °C every 30 seconds over 71 cycles. Curves were fitted using an online tool developed by P. Bond (Bond P. JTSA 2019. Available from: paulsbond.co.uk/jtsa)

Table S3: Change in melting temperature (ΔT_m) of MGATV with fragments **7d** and **8p** at 4 mM and 8 mM fragment concentrations.

Fragment	ΔT_m (°C)	
	4 mM	8 mM
**7d**	1.03 ± 0.003	1.10 ± 0.206
**8p**	0.66 ± 0.004	1.06 ± 0.156

3.4 UDP-Glo Enzyme Inhibition Kinetics

All enzyme reactions were carried out in white F96 microwell plates (Nunc), using the UDP-Glo glycosyltransferase assay kit (Promega), following the manufacturer's instructions. For inhibition kinetics, reaction mixtures containing 200 μM acceptor substrate M592 (Dextra), 5.0 nM MGATV and 0−8 mM Fragment **8p** were prepared in 25 μL final volumes of assay buffer (50 mM MES pH 6.25, 25 mM NaCl, 0.1 mg.mL^{-1} w/v BSA, 0.1% v/v Triton X-100). Reaction volumes were mixed by orbital shaking of the microplate for 1 min, before incubation at ambient temperature for 30 min. To visualize the reaction, 25 μL UDP detection reagent was added to each well, volumes mixed by orbital shaking for 1 minute, then microplate incubated for another hour at ambient temperature. Developed UDP-Glo reactions were measured using a Polarstar plate reader (BMG Labtech). Datapoints were calibrated to a standard curve of 0–25 μM UDP (Sigma) produced by serial dilution in assay buffer. The IC_{50} value (n = 1) was determined by fitting a constrained four-parameter logistic curve using nonlinear regression in GraphPad Prism version 8.1.0 (Figure S5)

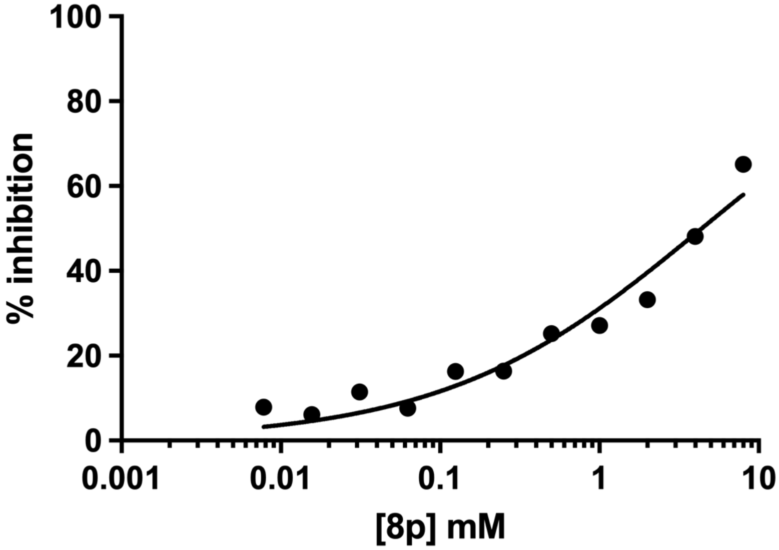

Figure S5: Enzyme kinetics plot for determination of IC_{50} of **8p**.

3.5 STD NMR Studies

NMR experiments were carried out on a Bruker Advance Neo 700 MHz NMR spectrometer equipped with a triple resonance probe and z axis gradients. Data were processed using Topspin 3.2 software. STD NMR[15] experiments were collected at 288K; the samples contained 10 µM MGATV, 500 µM fragment **8p**, 100 µM sodium trimethylsilylpropanesulfonate (DSS) in phosphate buffer (50 mM Sodium Phosphate, pH 6.25, 25 mM NaCl) containing 5% D_2O. The experiments were recorded using the standard STD NMR pulse sequence with a 15 ms 6 kHz spin-lock pulse to reduce the background protein resonances and excitation sculpting to suppress the water resonance. Saturation of the protein NMR signals of the enzyme were performed using a train of 70 selective 69 Hz Gaussian pulses with duration of 50 ms, adding up to a total saturation time of 3.5 s. The on-resonance frequency was set to 0.5 ppm and off resonance irradiation was applied at 50 ppm. STD NMR spectra were acquired with a total of 480 transients in addition to 16 scans to allow the sample to come to equilibrium. Spectra were performed with a sweep width of 16 ppm and 16k data points corresponding to an acquisition time of 0.7 s and with an additional relaxation delay of 0.1 s giving a total repetition time of 4.3 s for the experiment.

We confirmed the validity of **8p** as an MGATV ligand by STD NMR studies (Figure S6). STD NMR showed clear peaks at *δ* 7.35–7.22 (Ar), 2.31 and 2.30 (s, Ar*Me*), 2.20 and 2.03 (s, *Me*CO) ppm in the STD spectrum of **8p** with MGATV (note that the methyl singlets are split 70:30 due to amide rotamers). This series of peaks correspond to the aryl ring and two methyl moieties of the fragment and are clearly indicative of ligand/enzyme interaction. The highly coupled and broadened signals corresponding to the piperidine ring are not visible in the STD spectrum.

The weaker singlet of the split methyl singlet peaks in the reference spectrum is enhanced in the STD spectrum to a greater extent than the stronger peak (Figure S6C). This shifts the ratio from 70:30 to close to 50:50, perhaps suggesting an altered rotamer preference in the MGATV-bound form.

The STD-NMR signal, thermal shift data, and partial response IC_{50} curve demonstrate consistent behavior of fragment **8p** across three assays. This is an excellent example of the utility of orthogonal assays in validating weak fragments hits.

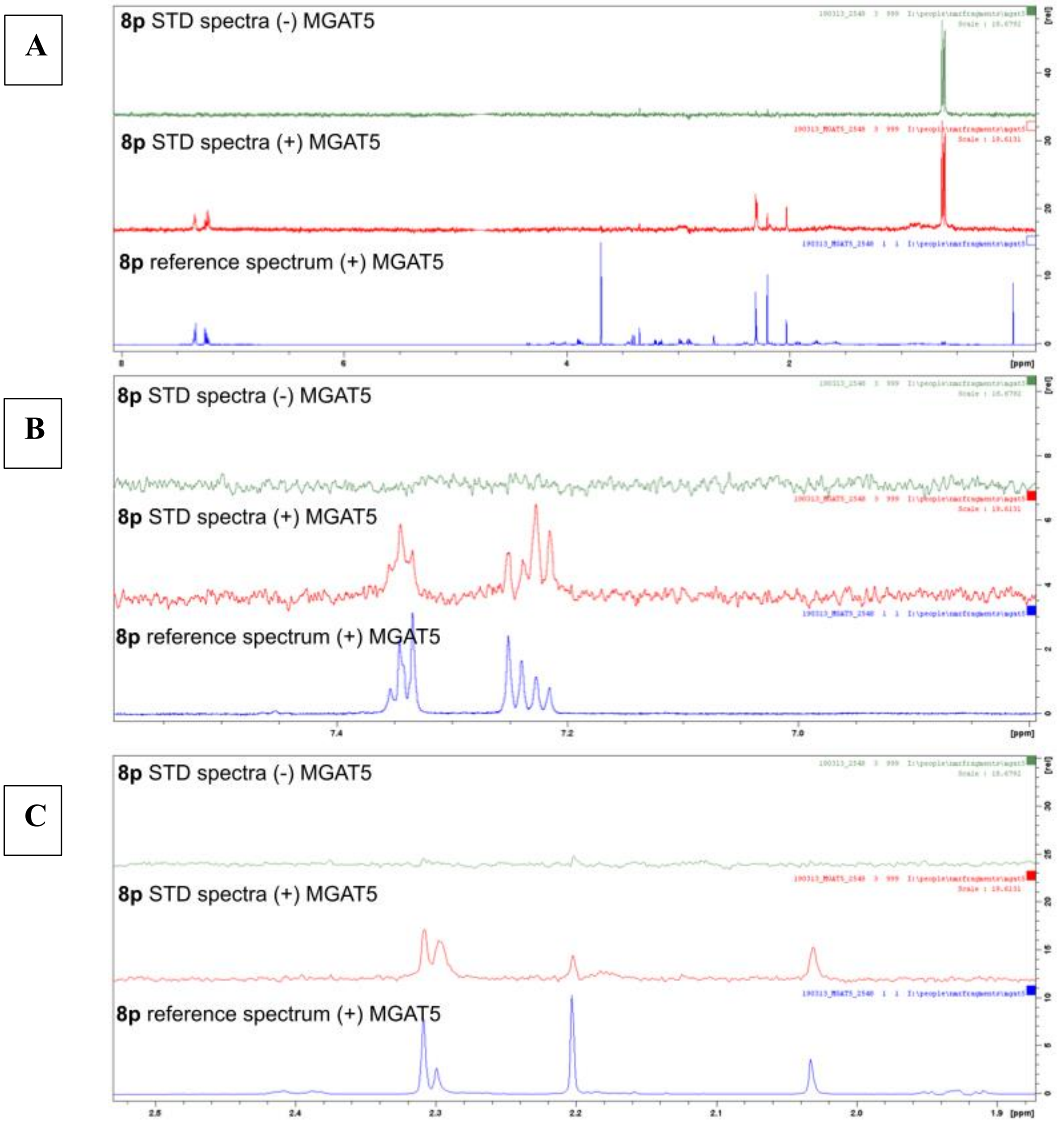

Figure S6. **A.** NMR spectrum of fragment **8p** showing the ^{1}H reference spectra (blue), and STD NMR spectrum in the presence (red), and absence (green) of MGATV. **B.** Detail of the aromatic region coloured as in **A**. **C.** Detail of the aliphatic region coloured as in **A**.

4. ^{1}H and ^{13}C NMR Spectra

400 MHz ^{1}H NMR spectrum; 100.6 MHz ^{13}C NMR spectrum; $CDCl_3$ of **1a**

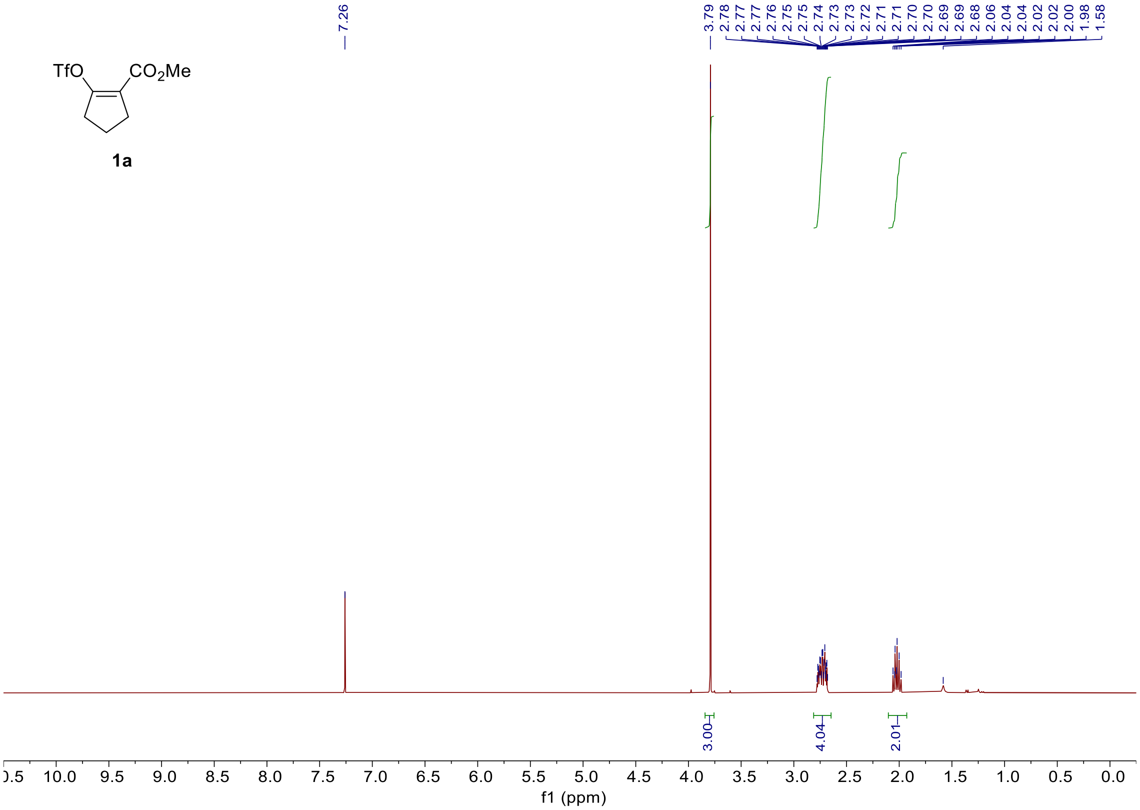

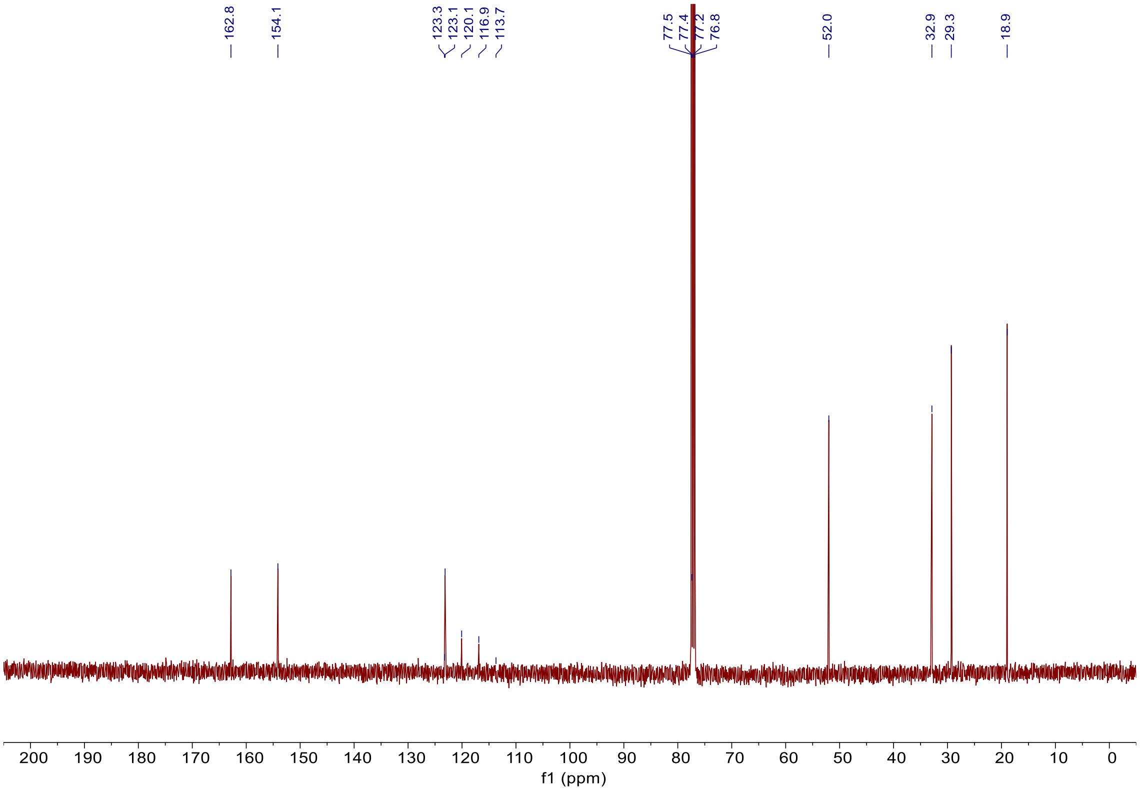

400 MHz ^{1}H NMR spectrum; 100.6 MHz ^{13}C NMR spectrum; $CDCl_3$ of **S1**

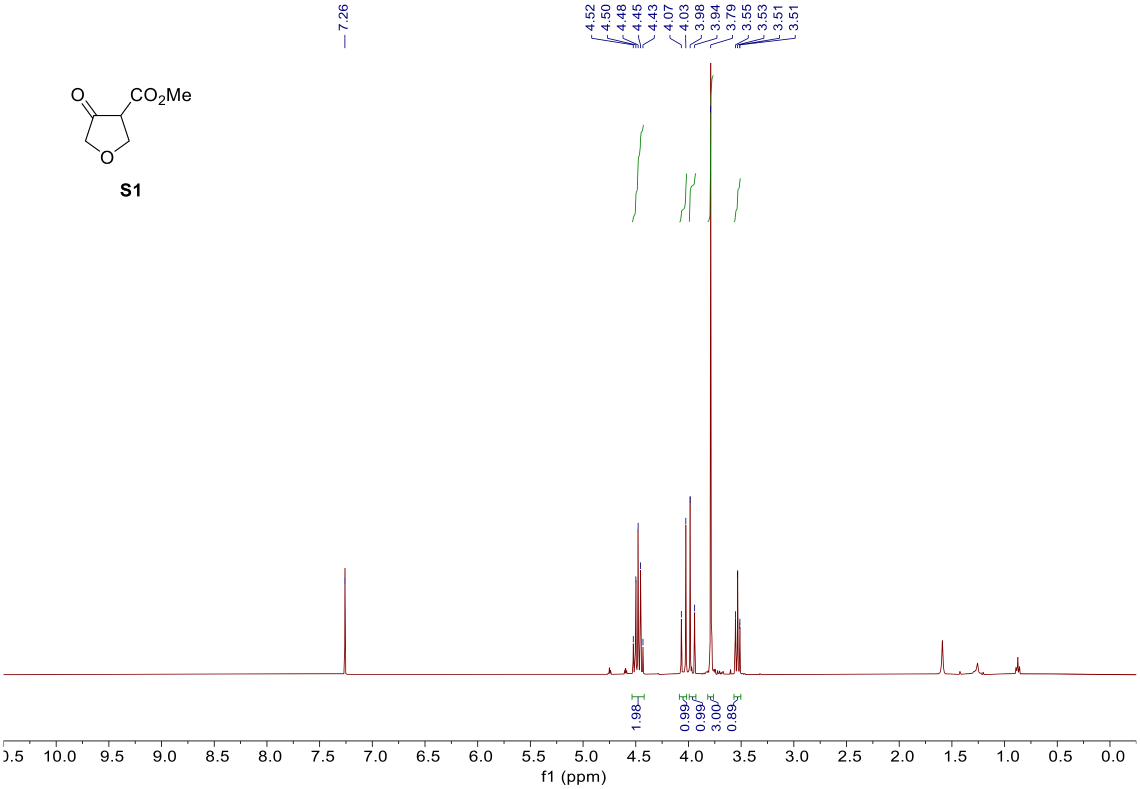

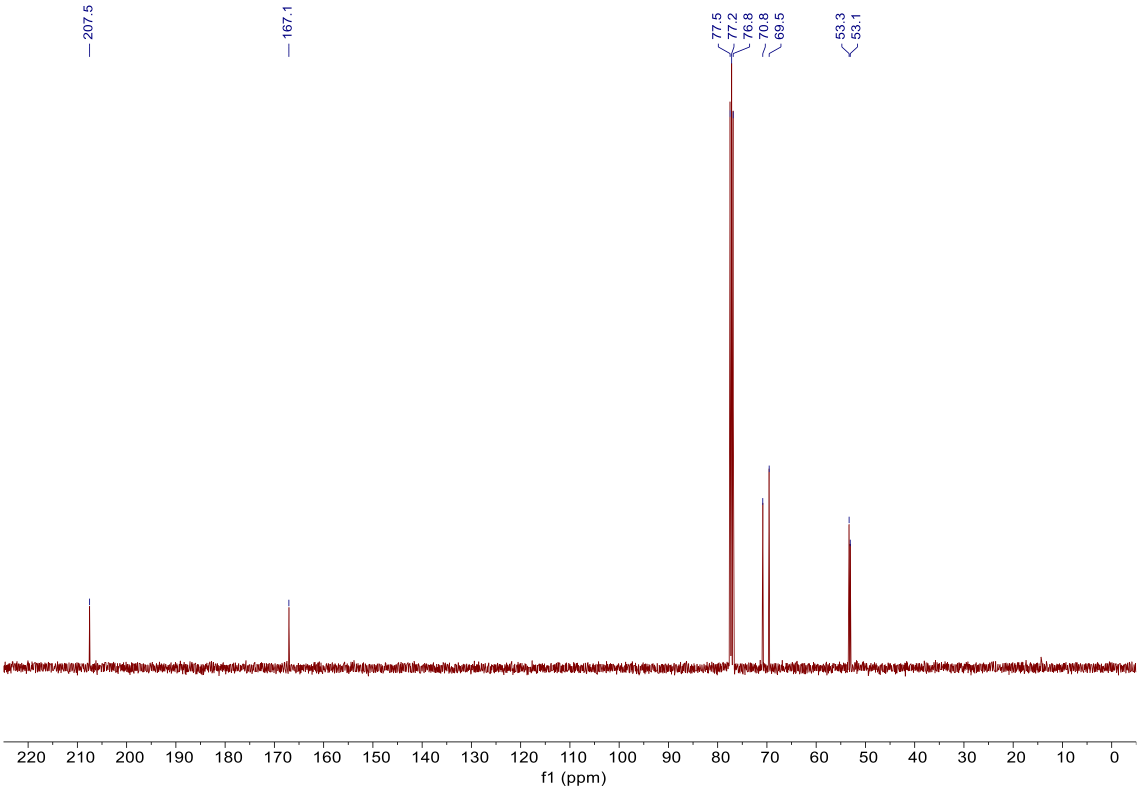

400 MHz ^1H NMR spectrum; 100.6 MHz ^{13}C NMR spectrum; $CDCl_3$ of **S2**

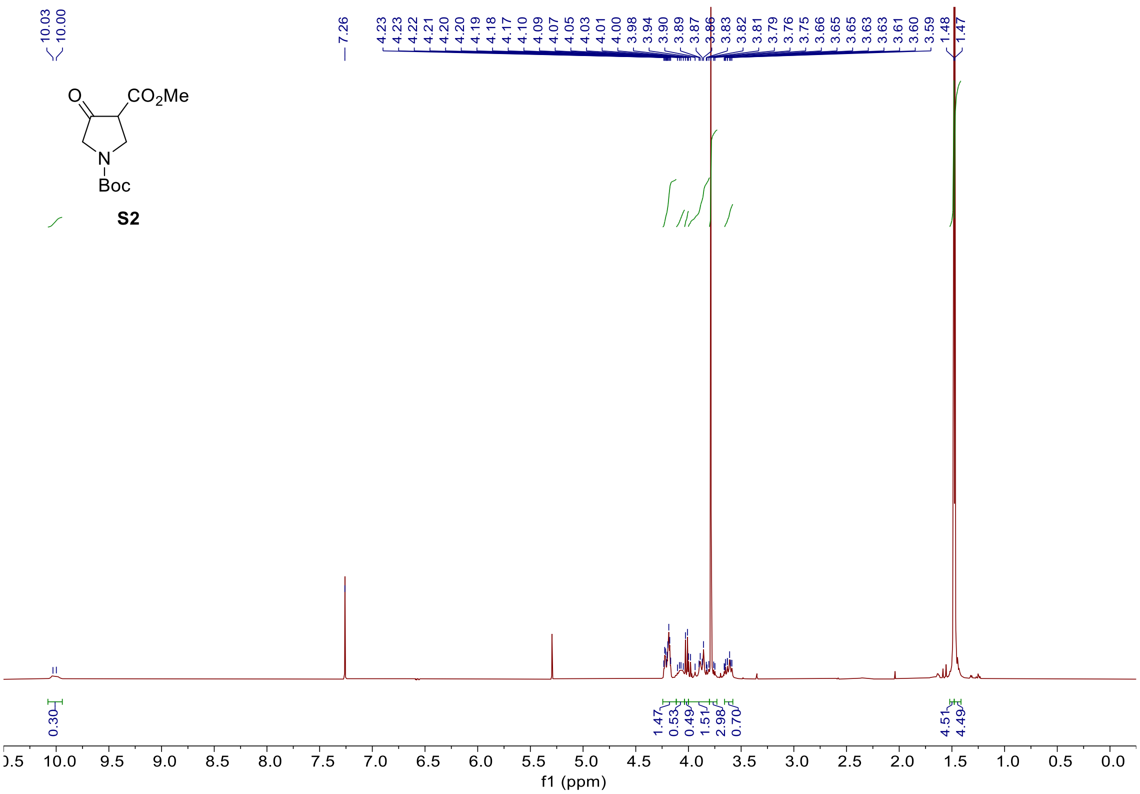

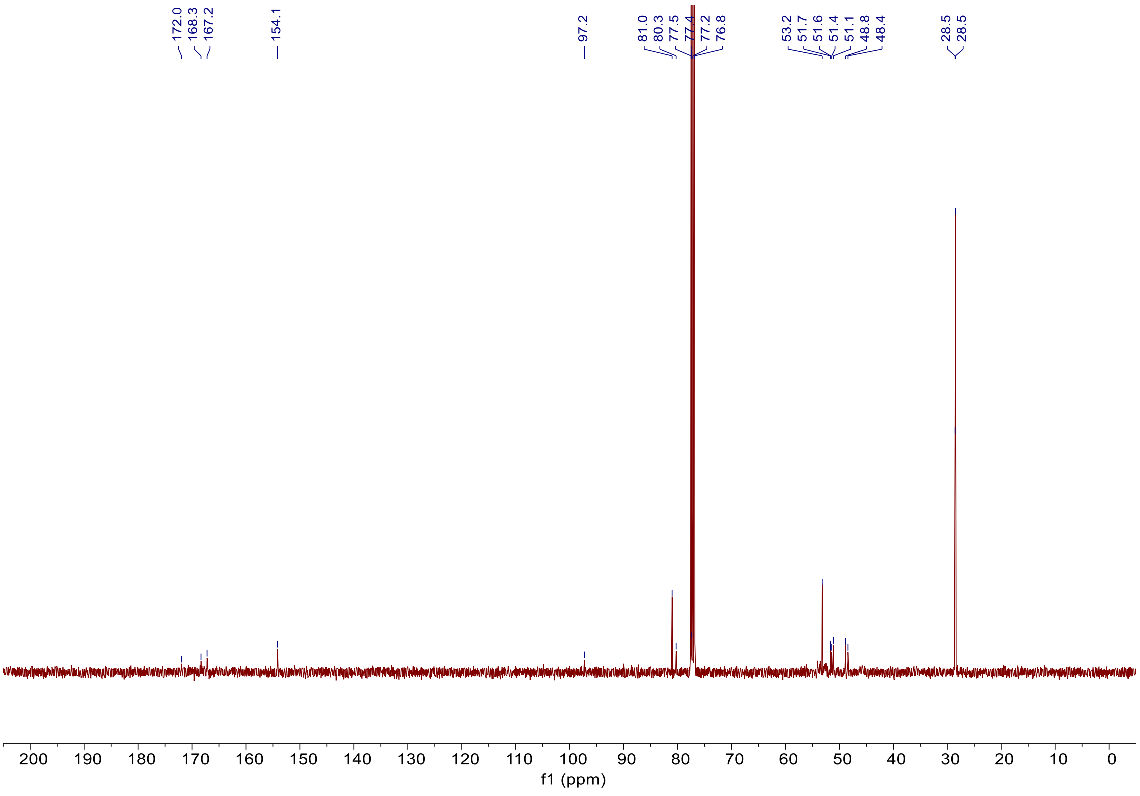

400 MHz ^1H NMR spectrum; 100.6 MHz ^{13}C NMR spectrum; $CDCl_3$ of **1b**

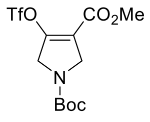

1b

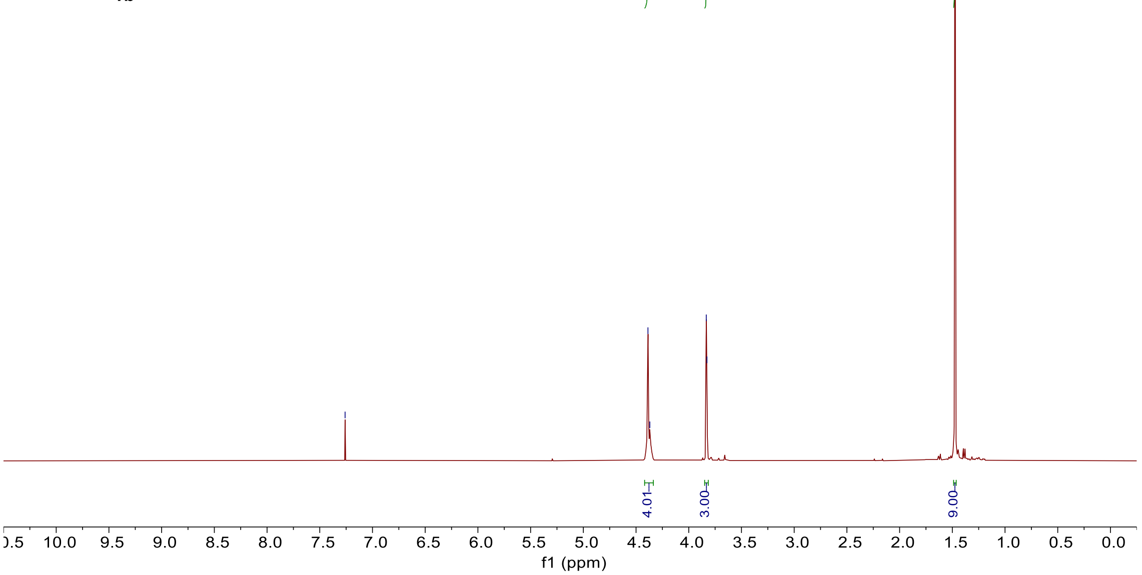

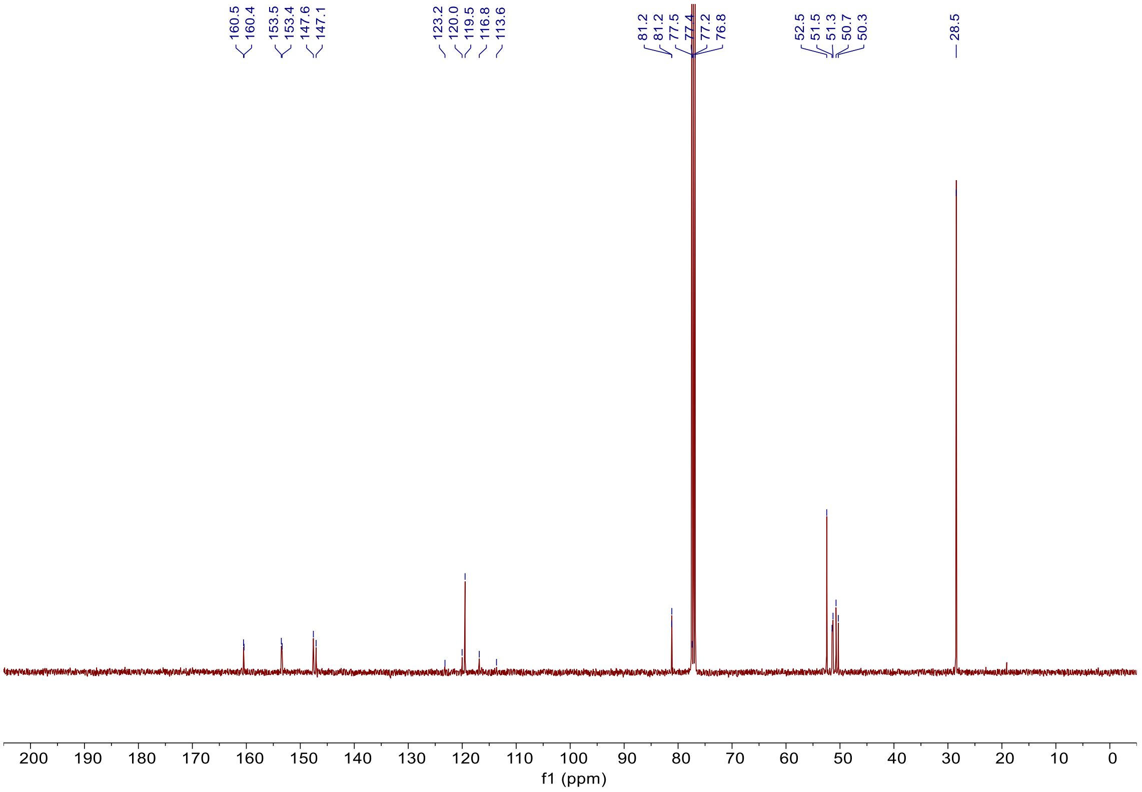

400 MHz ^1H NMR spectrum; 100.6 MHz ^{13}C NMR spectrum; $CDCl_3$ of **S3**

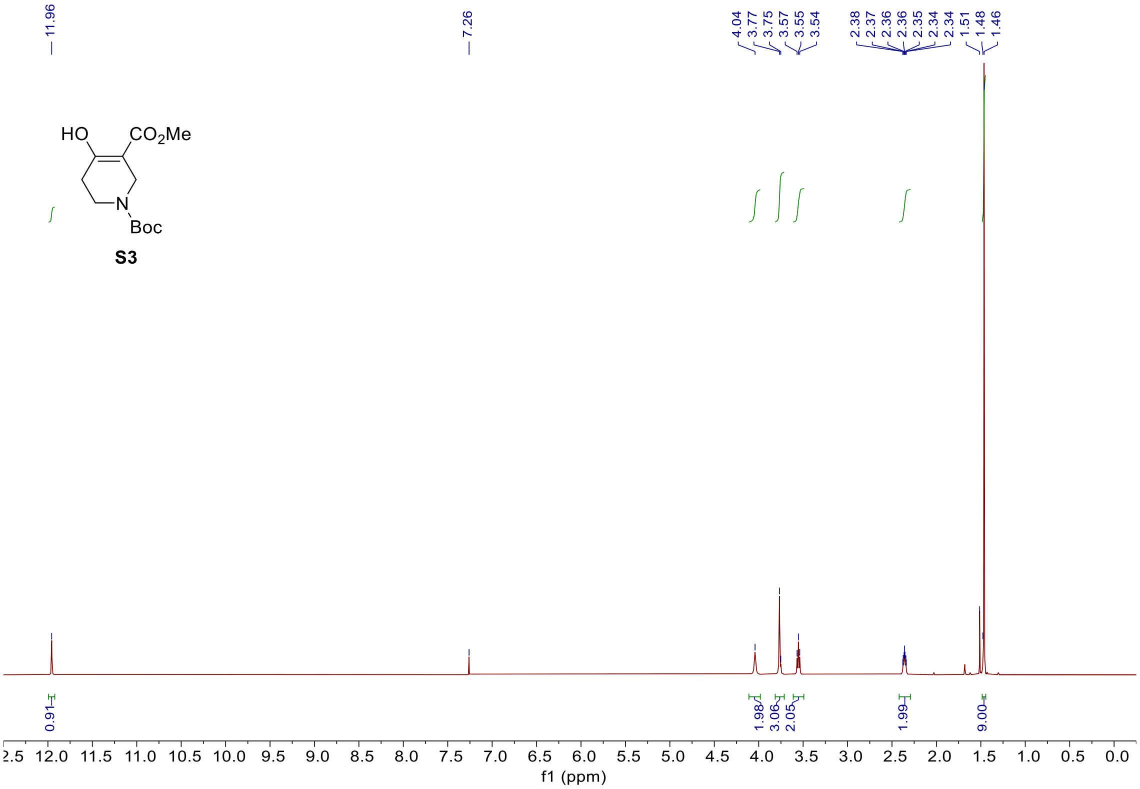

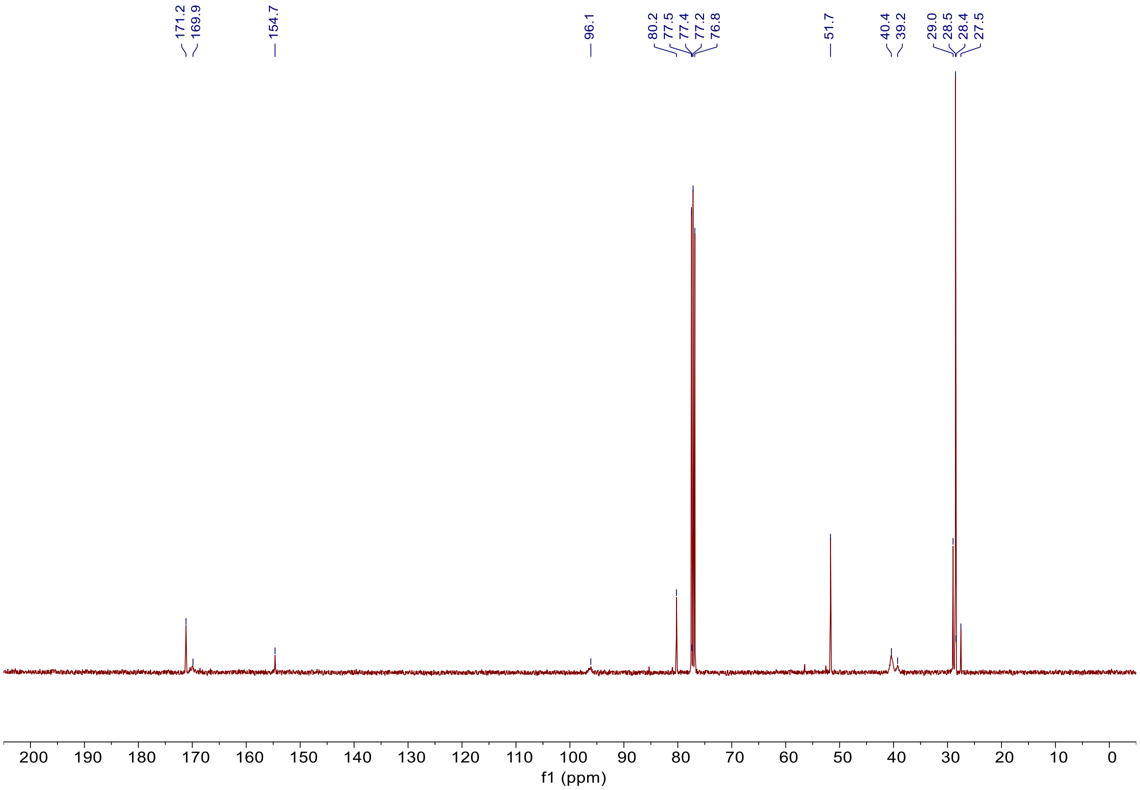

400 MHz ^{1}H NMR spectrum; 100.6 MHz ^{13}C NMR spectrum; $CDCl_3$ of **1c**

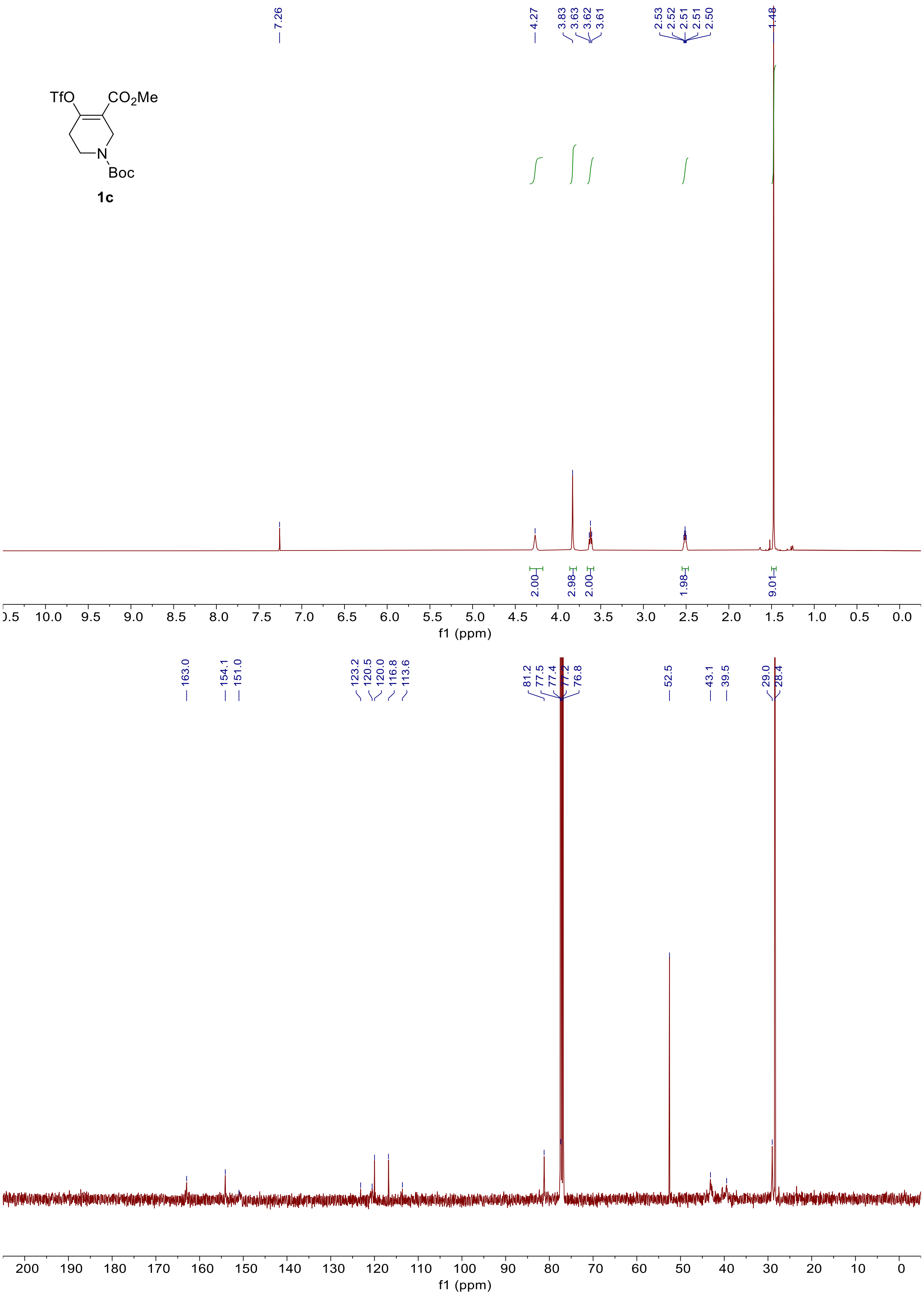

400 MHz ^{1}H NMR spectrum; 100.6 MHz ^{13}C NMR spectrum; $CDCl_3$ of **S4**

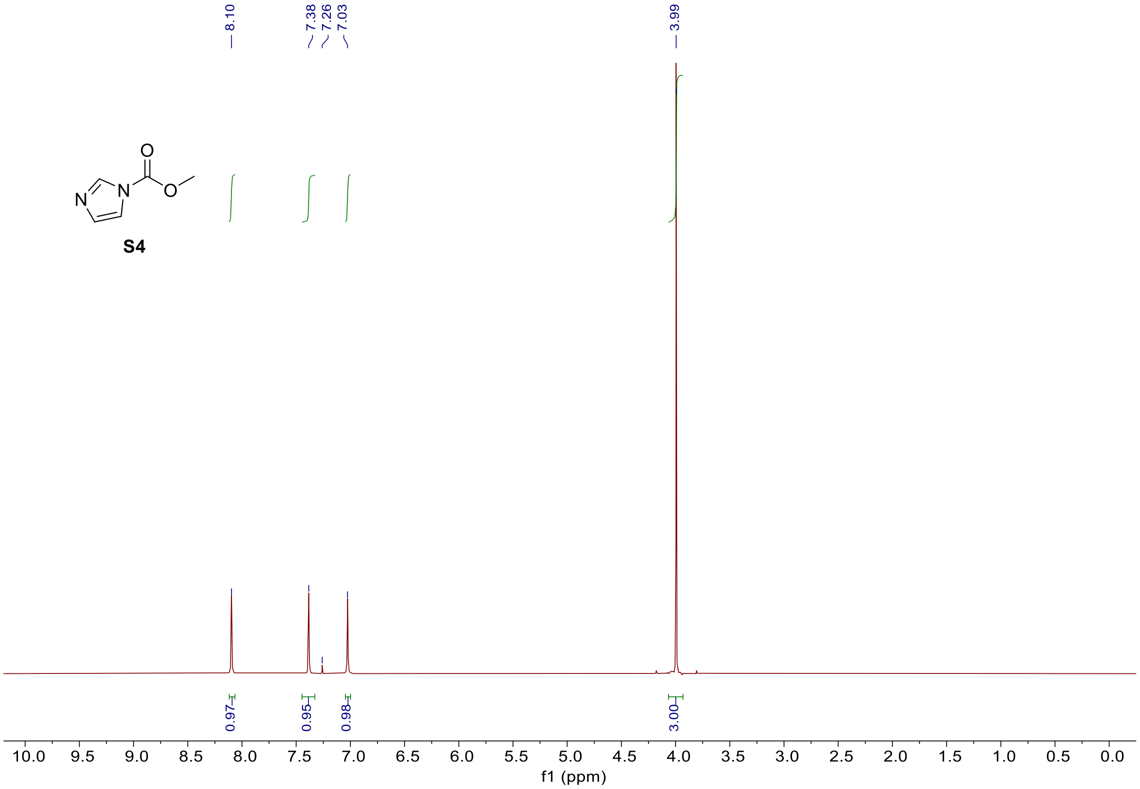

S4

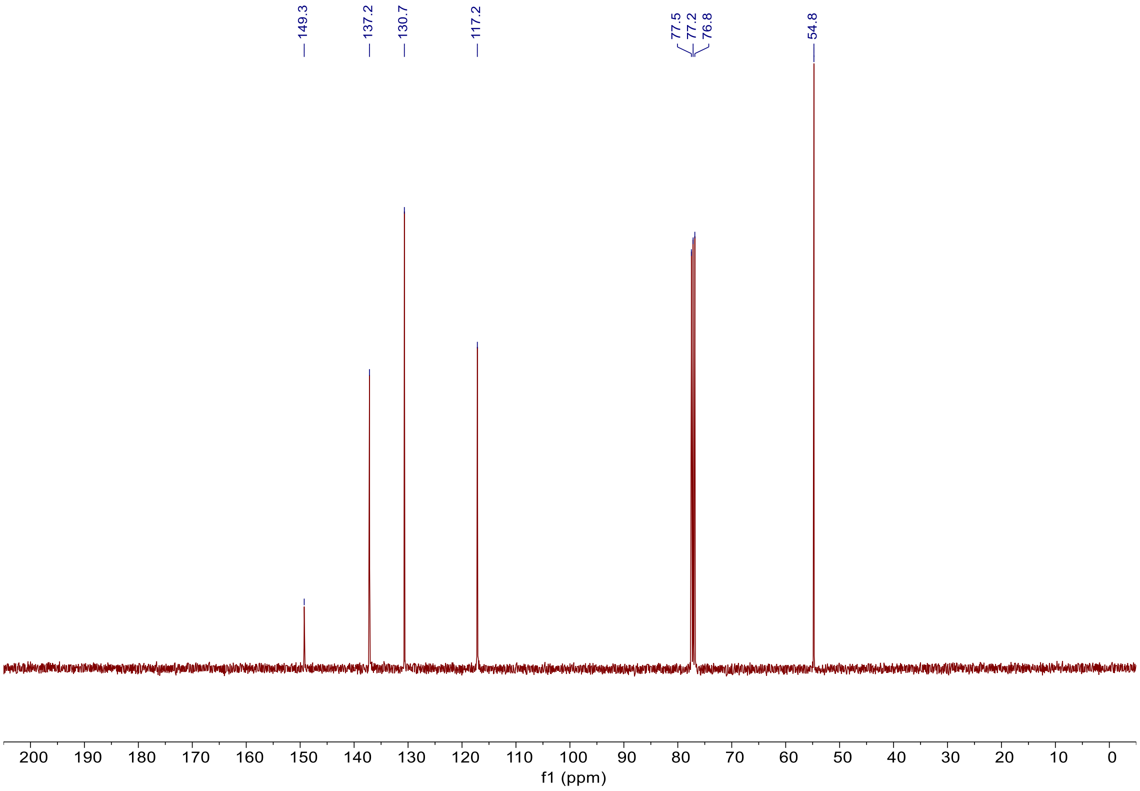

400 MHz ^{1}H NMR spectrum; 100.6 MHz ^{13}C NMR spectrum; $CDCl_3$ of **S5**

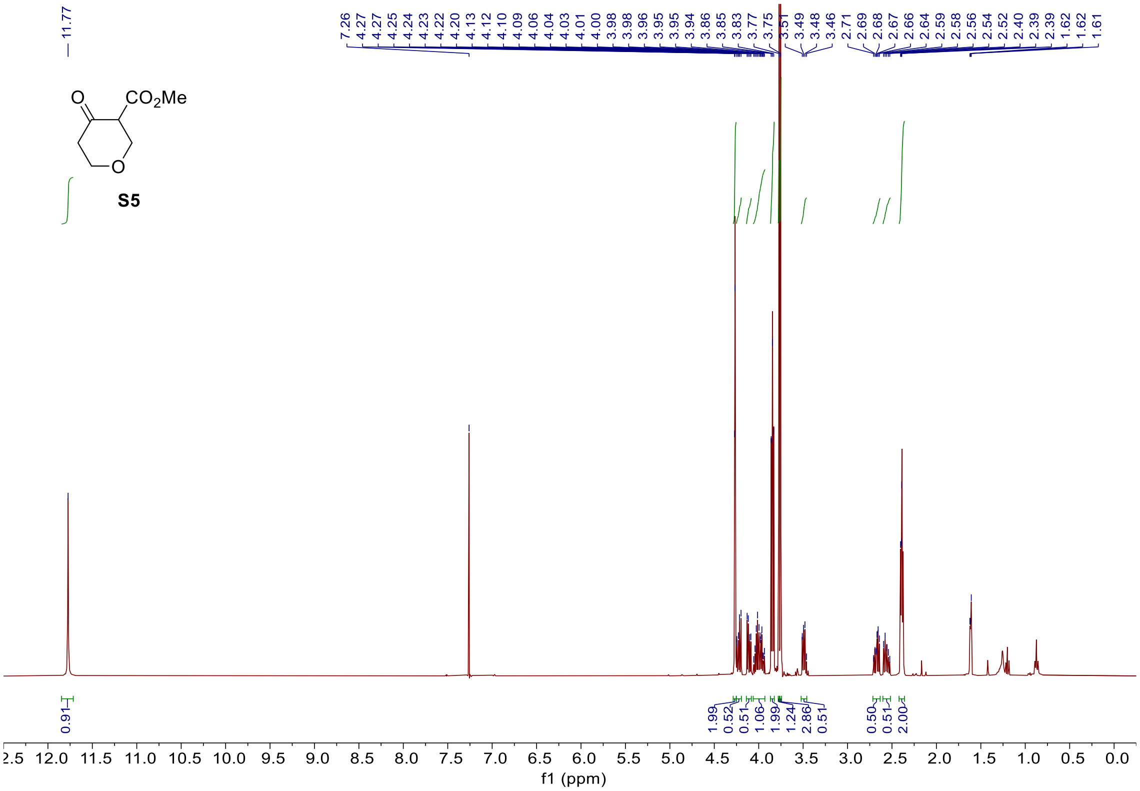

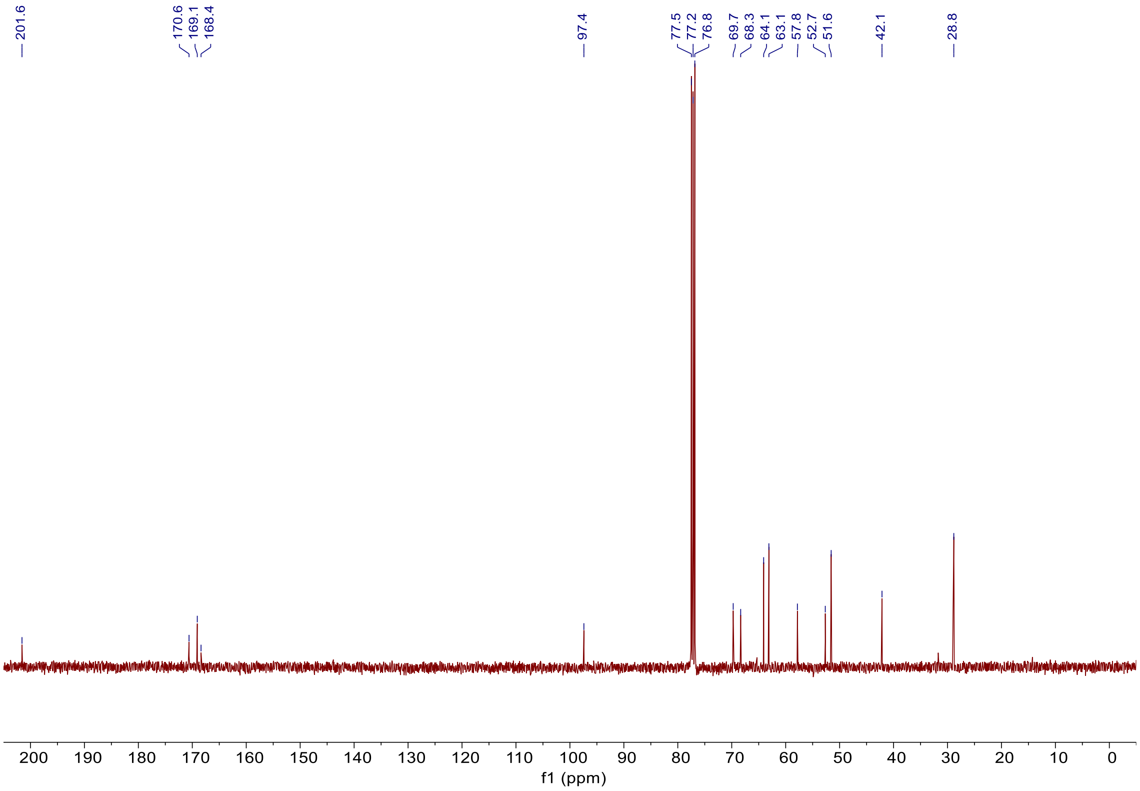

400 MHz ^{1}H NMR spectrum; 100.6 MHz ^{13}C NMR spectrum; $CDCl_3$ of **6a**

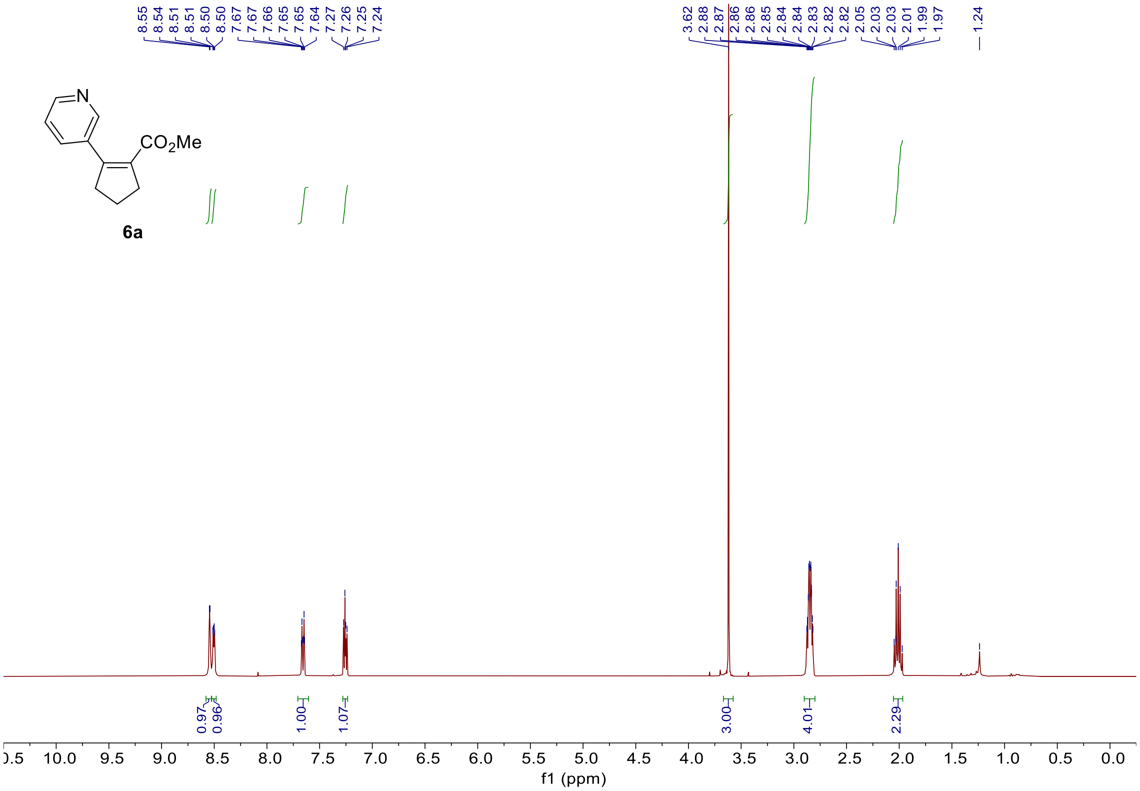

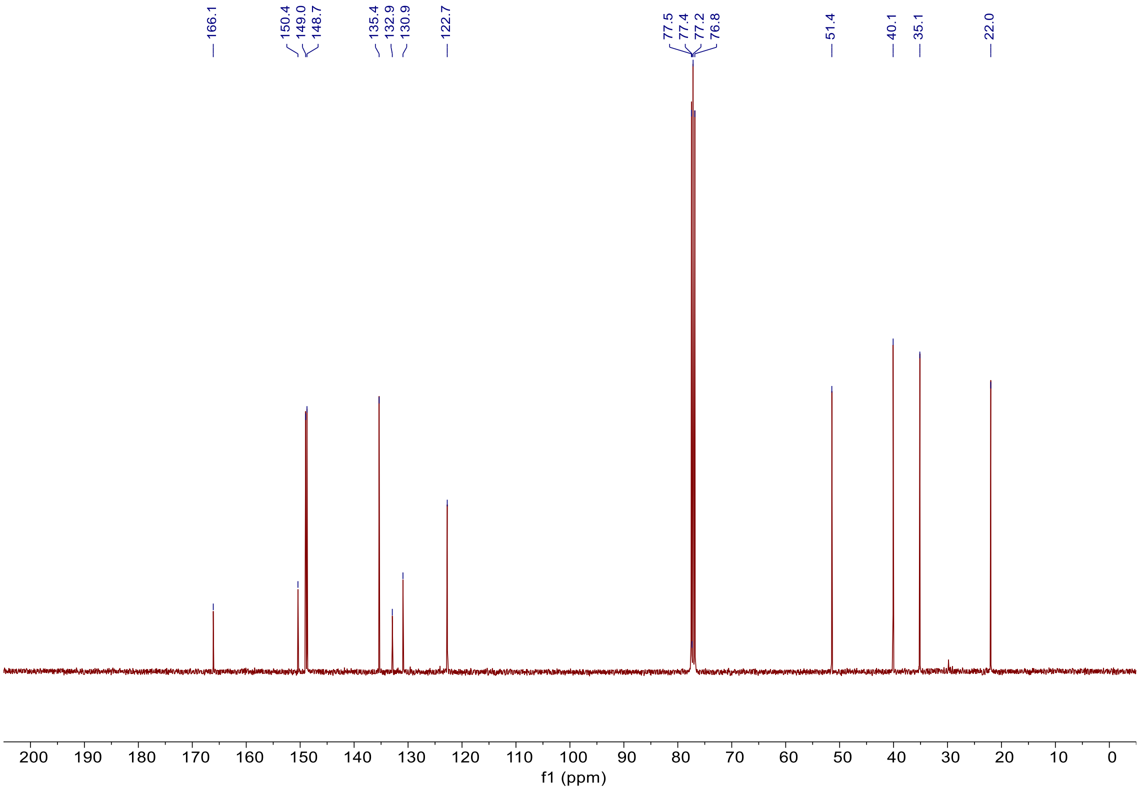

400 MHz ^{1}H NMR spectrum; 100.6 MHz ^{13}C NMR spectrum; $CDCl_3$ of **6b**

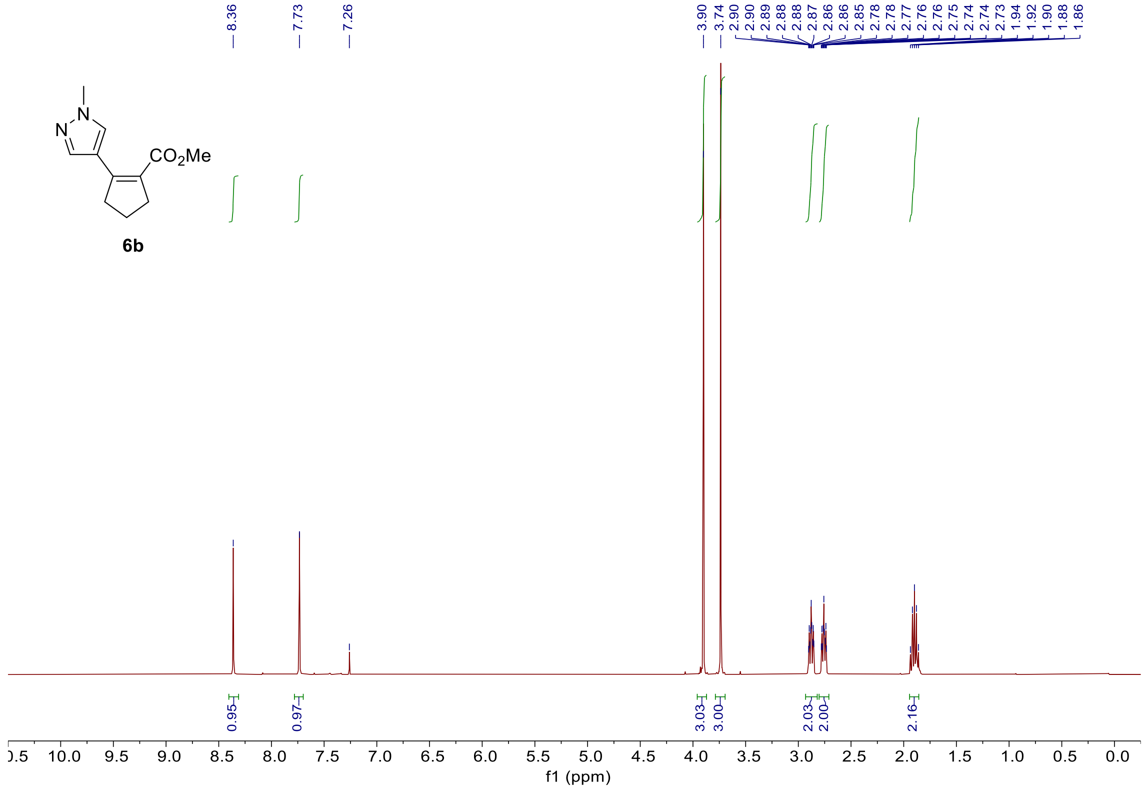

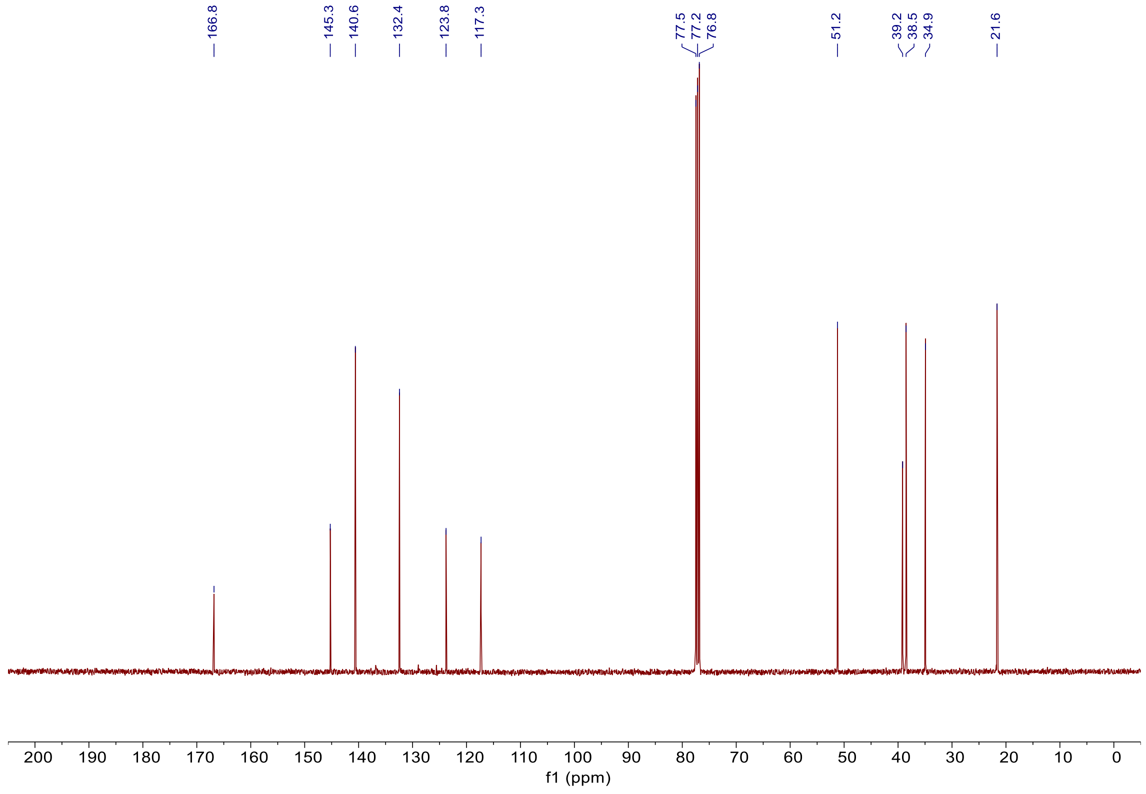

400 MHz ^{1}H NMR spectrum; 100.6 MHz ^{13}C NMR spectrum; $CDCl_3$ of **6c**

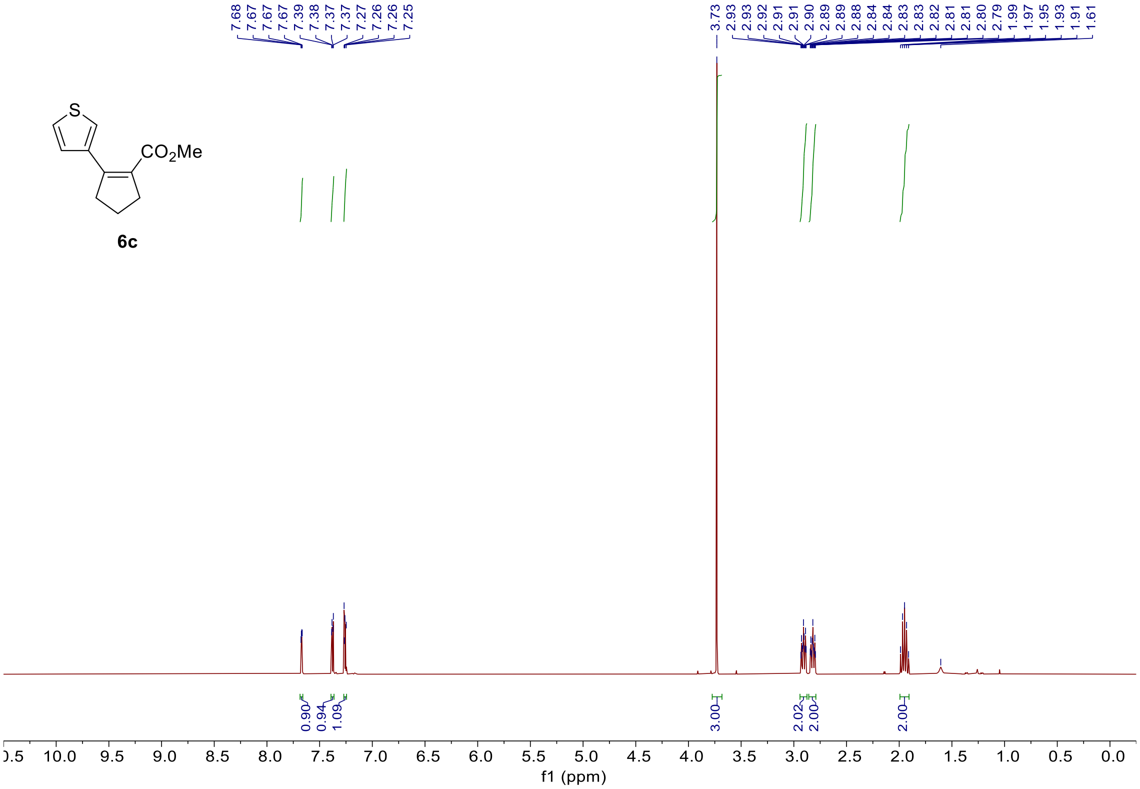

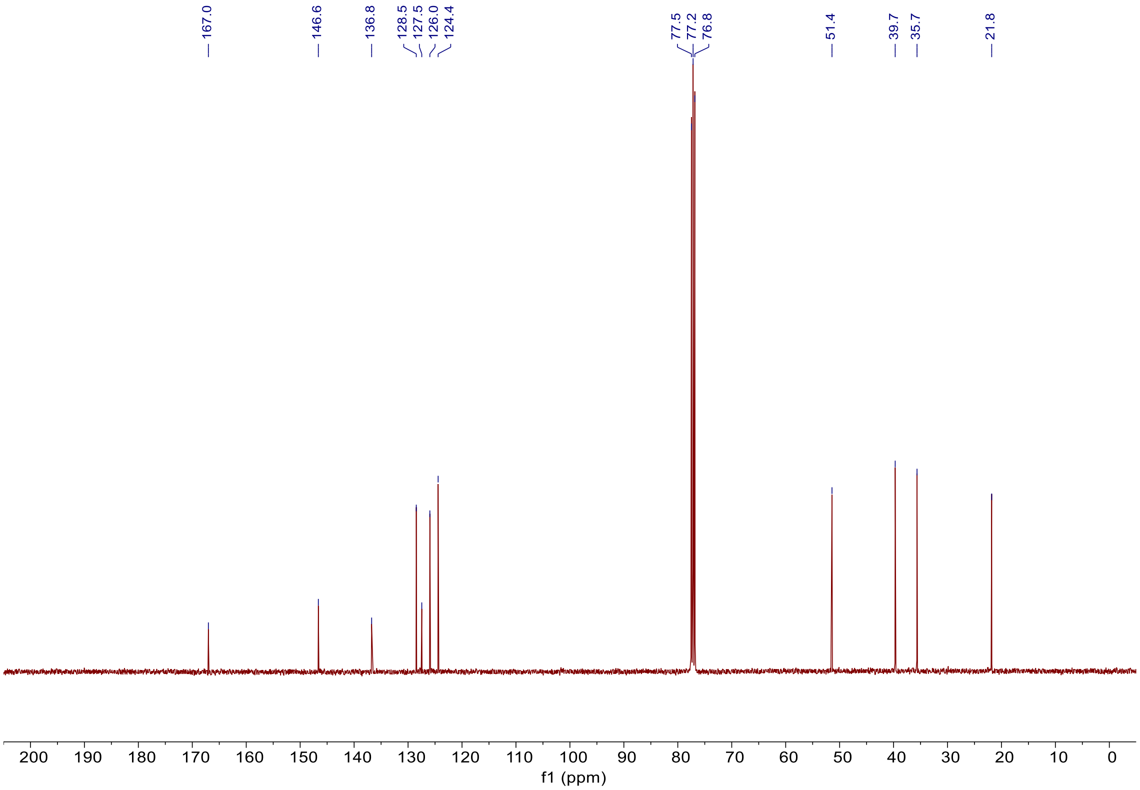

400 MHz ^{1}H NMR spectrum; 100.6 MHz ^{13}C NMR spectrum; $CDCl_3$ of **6d**

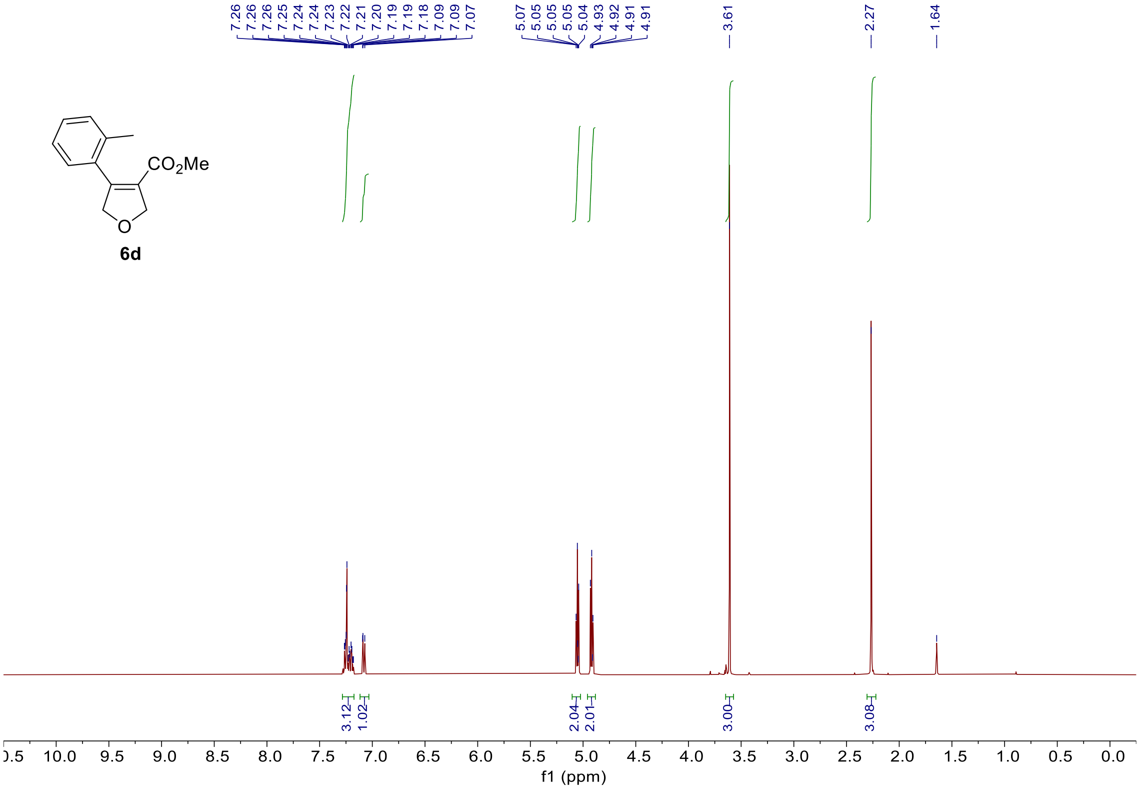

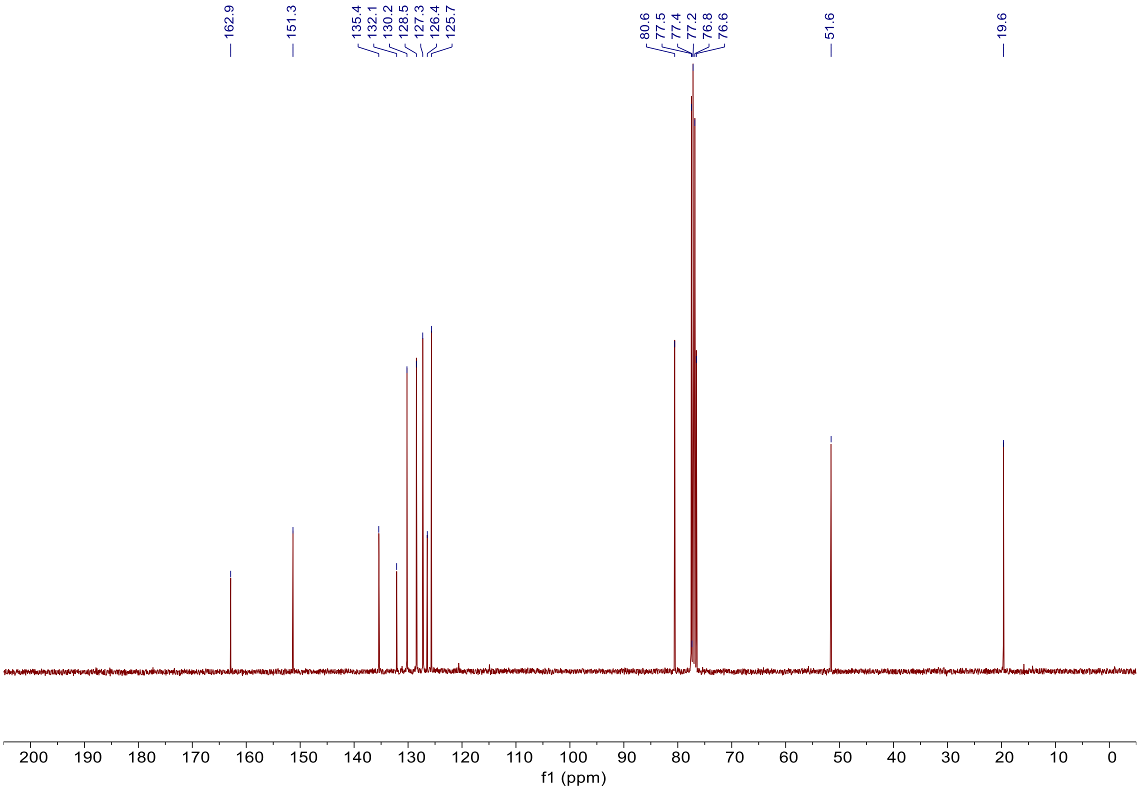

400 MHz ^{1}H NMR spectrum; 100.6 MHz ^{13}C NMR spectrum; $CDCl_3$ of **6e**

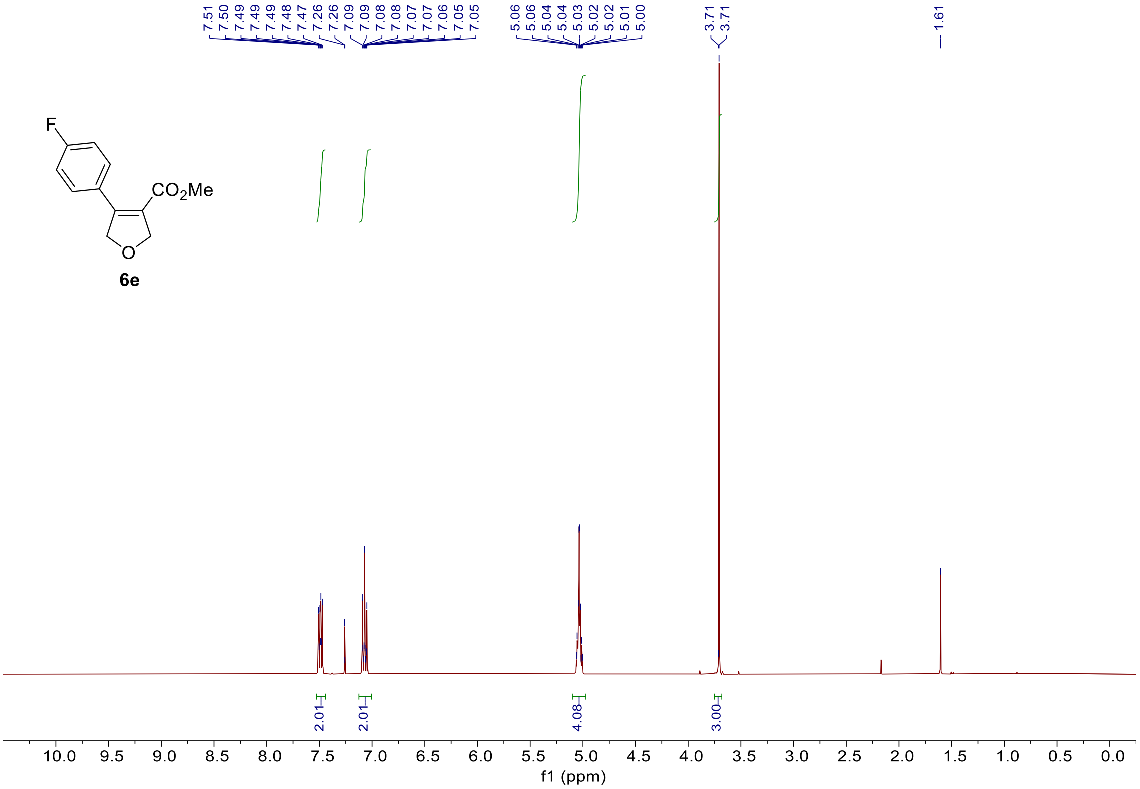

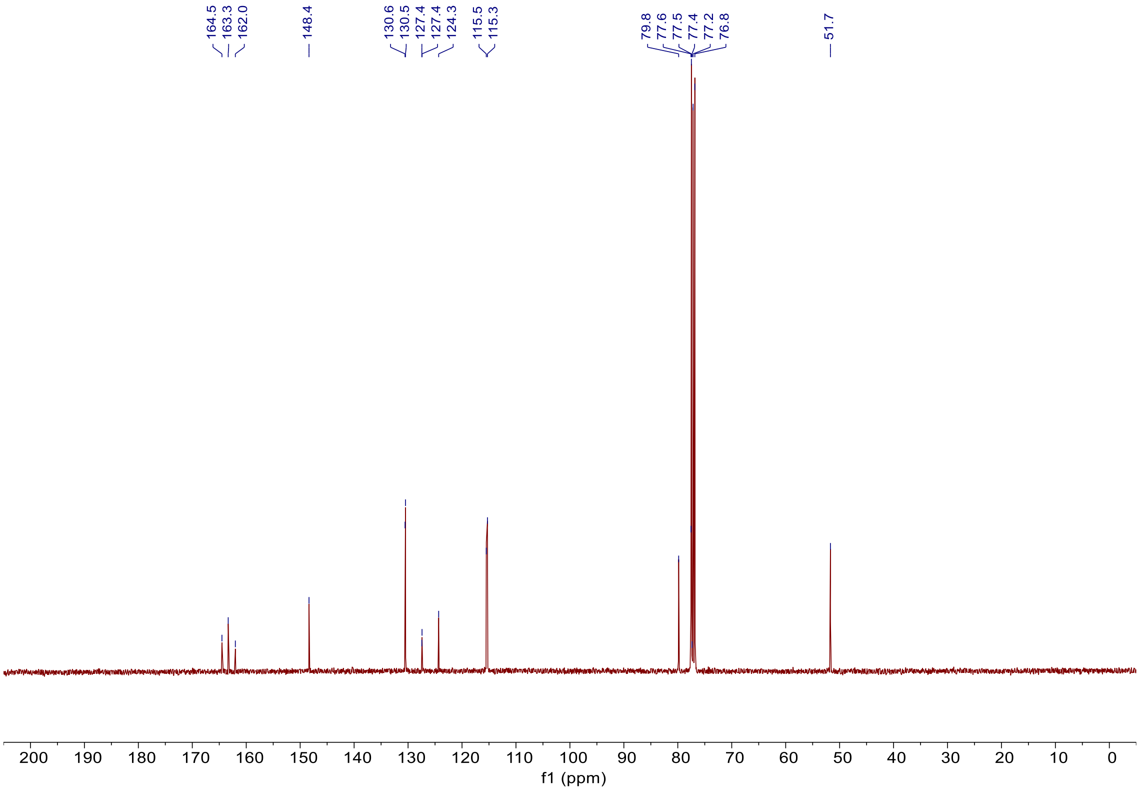

400 MHz ^{1}H NMR spectrum; 100.6 MHz ^{13}C NMR spectrum; $CDCl_3$ of **6f**

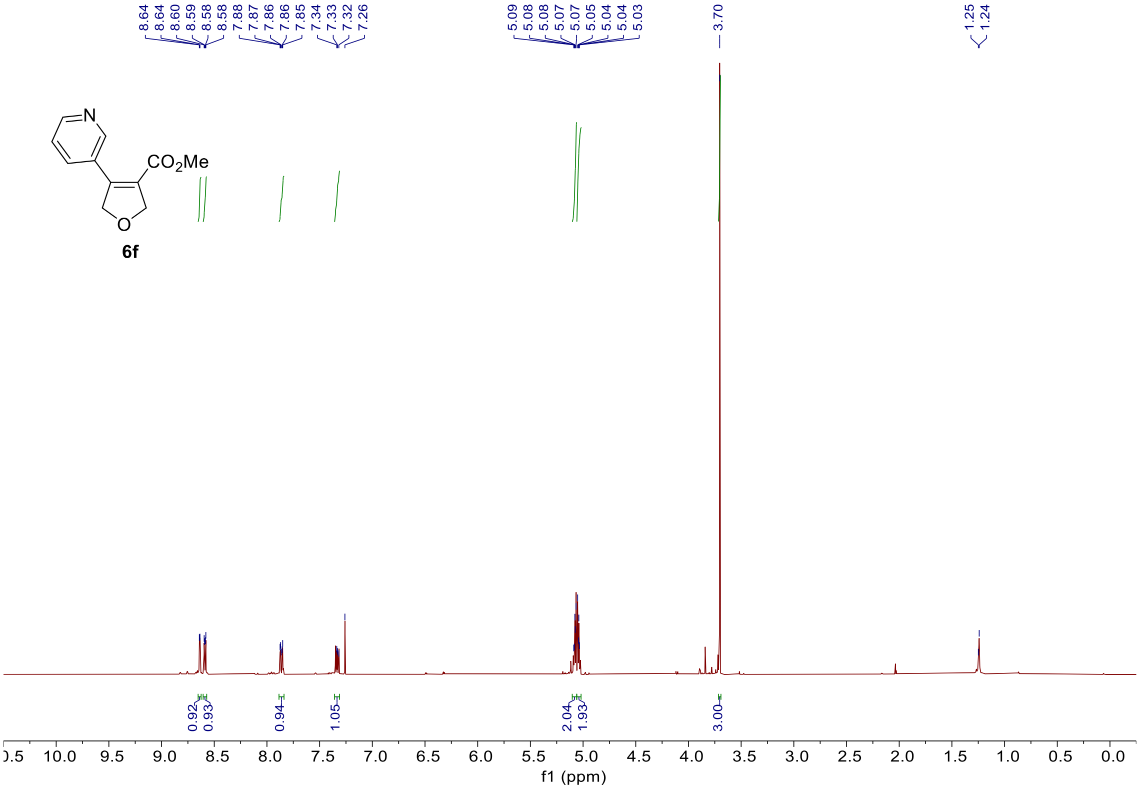

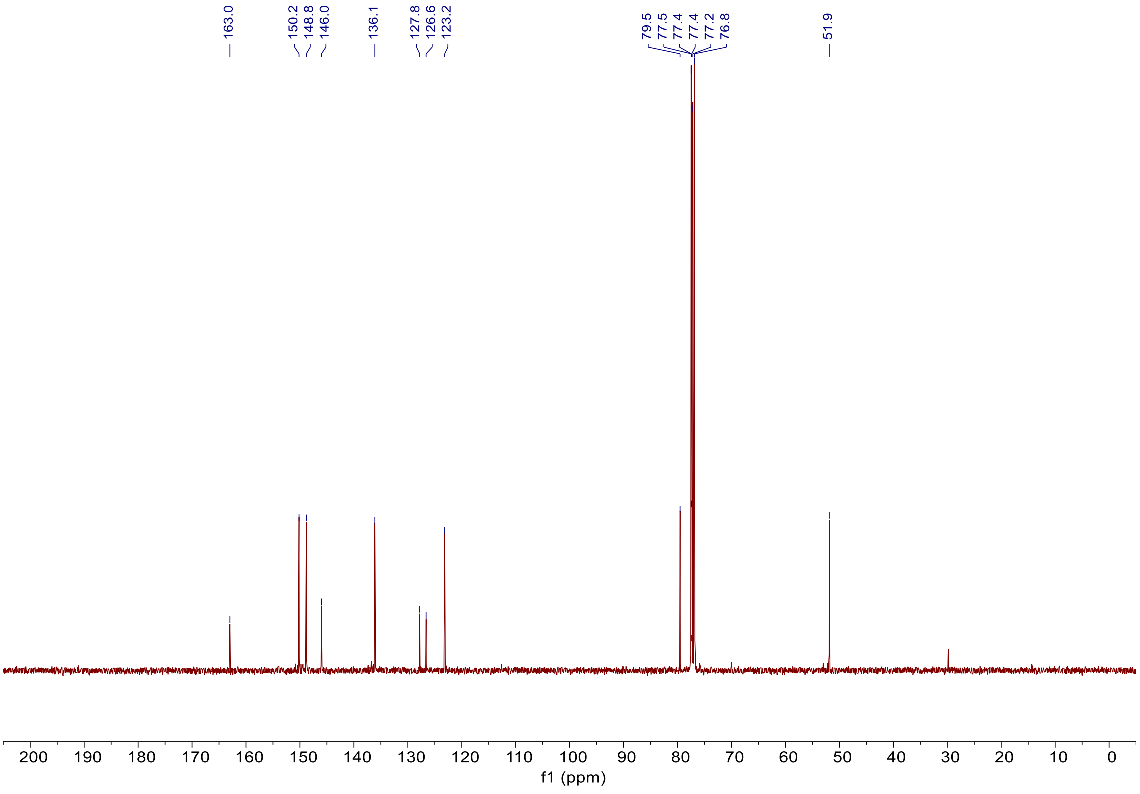

400 MHz ^1H NMR spectrum; $CDCl_3$ of **6g**

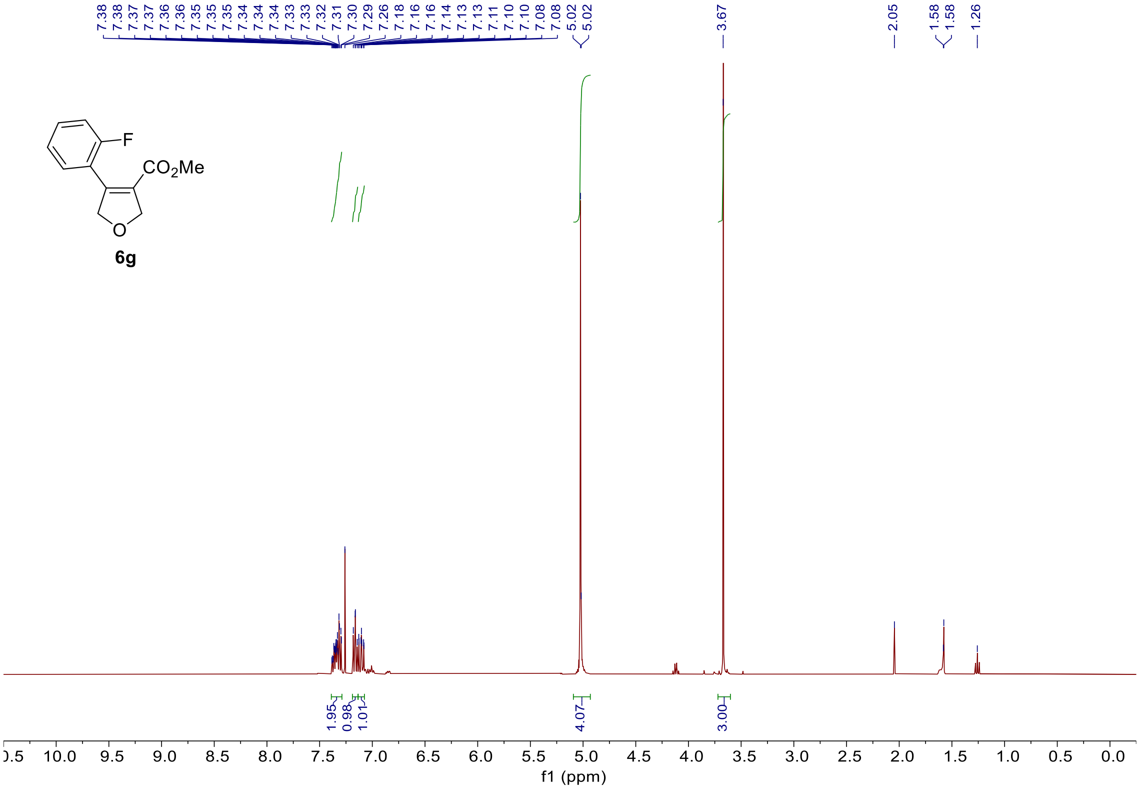

400 MHz ^{1}H NMR spectrum; 100.6 MHz ^{13}C NMR spectrum; $CDCl_3$ of **6h**

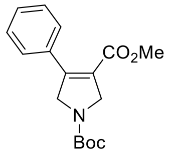

6h

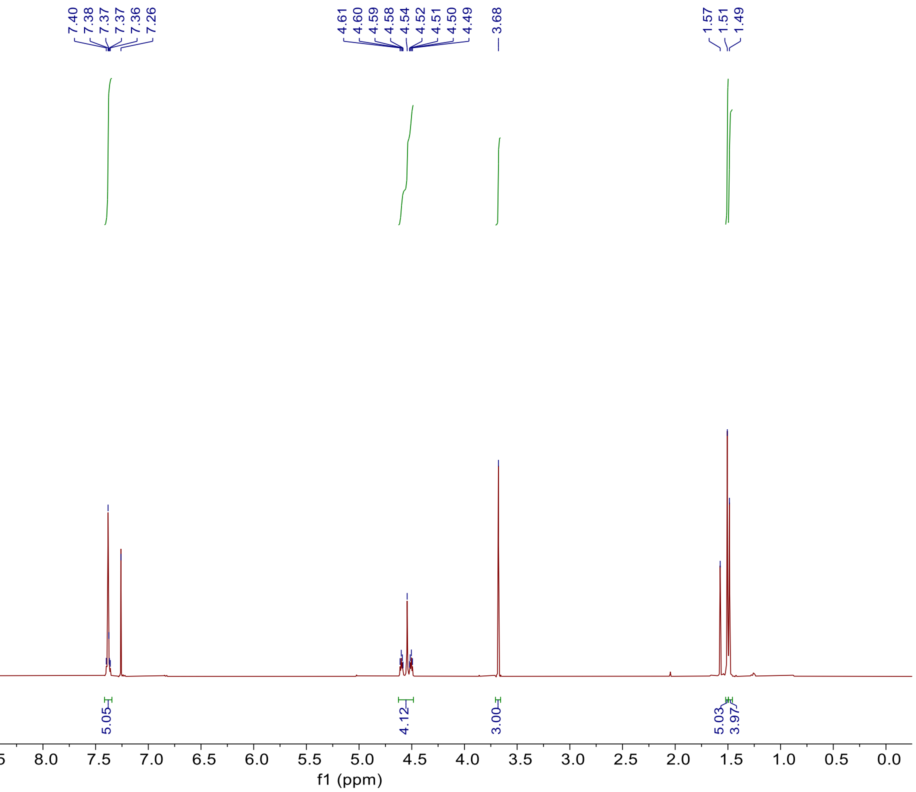

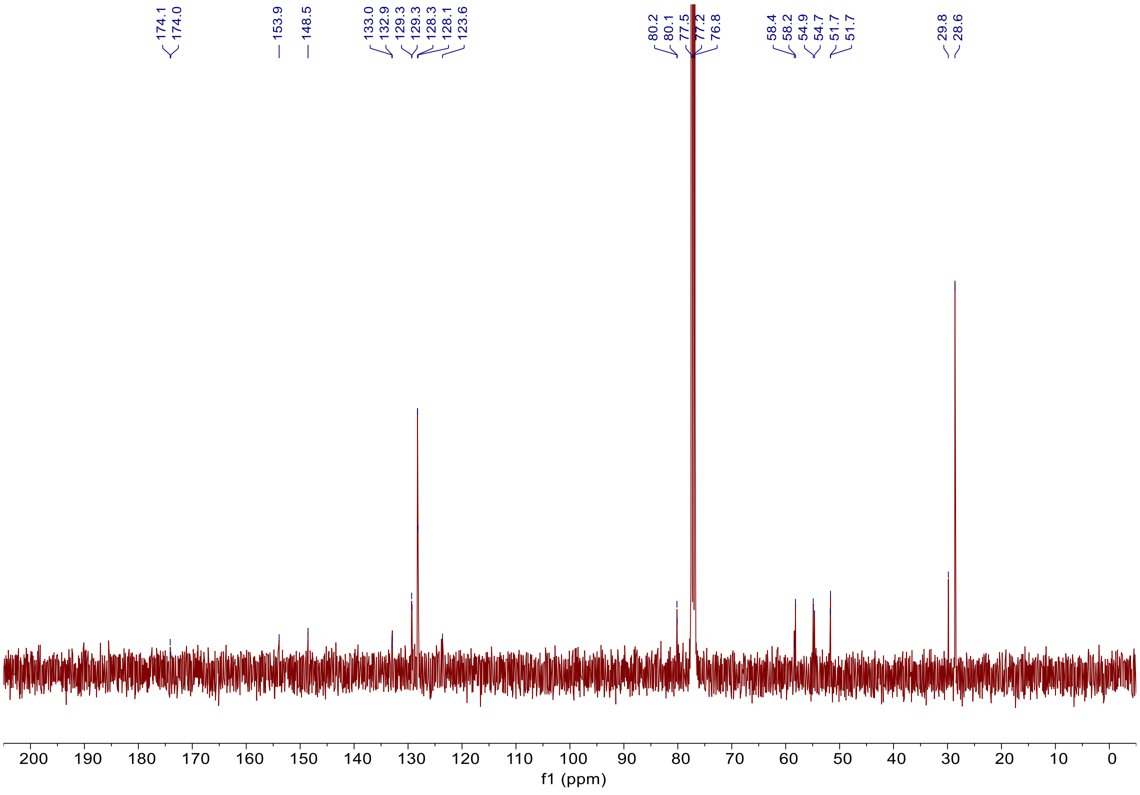

400 MHz ^{1}H NMR spectrum; 100.6 MHz ^{13}C NMR spectrum; $CDCl_3$ of **6i**

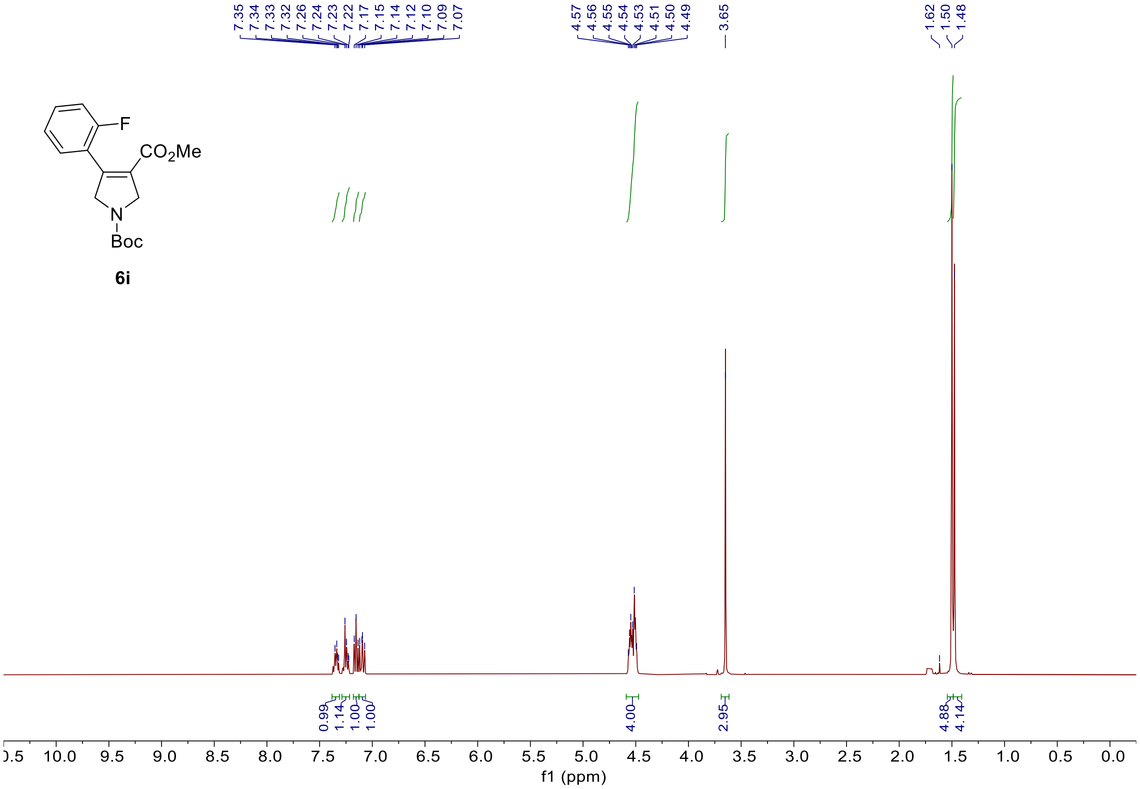

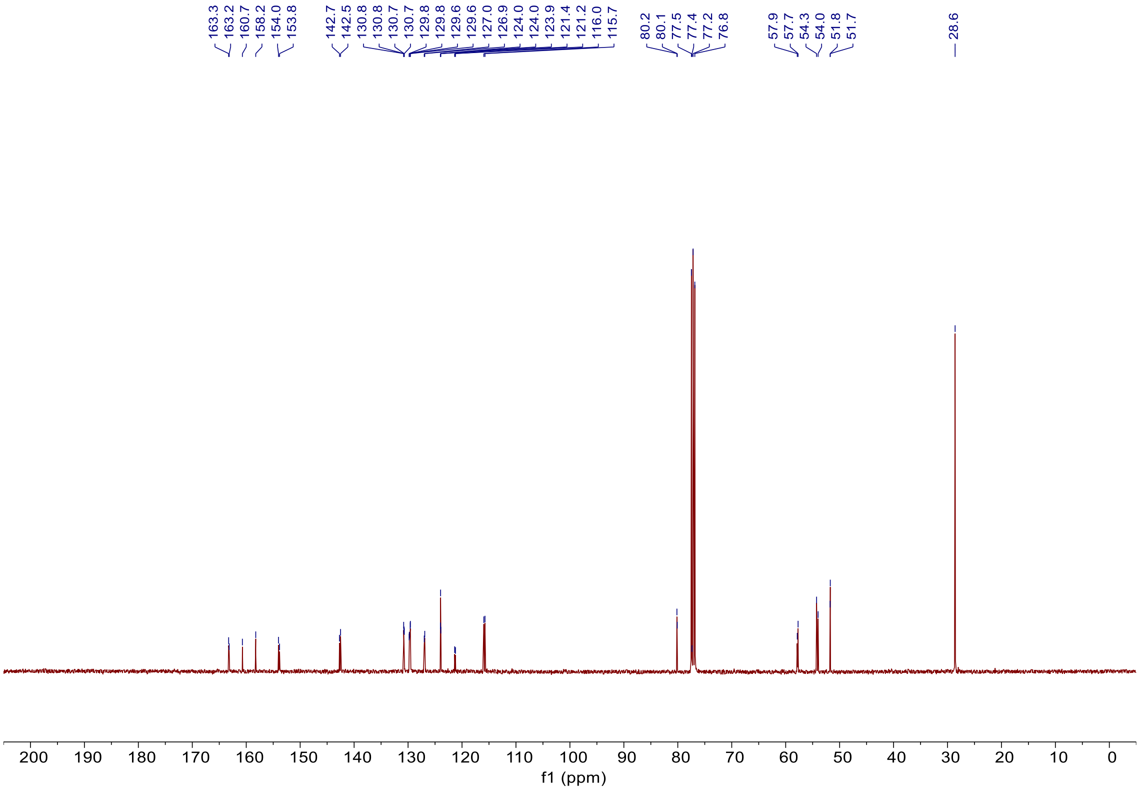

400 MHz ^{1}H NMR spectrum; 100.6 MHz ^{13}C NMR spectrum; $CDCl_3$ of **6j**

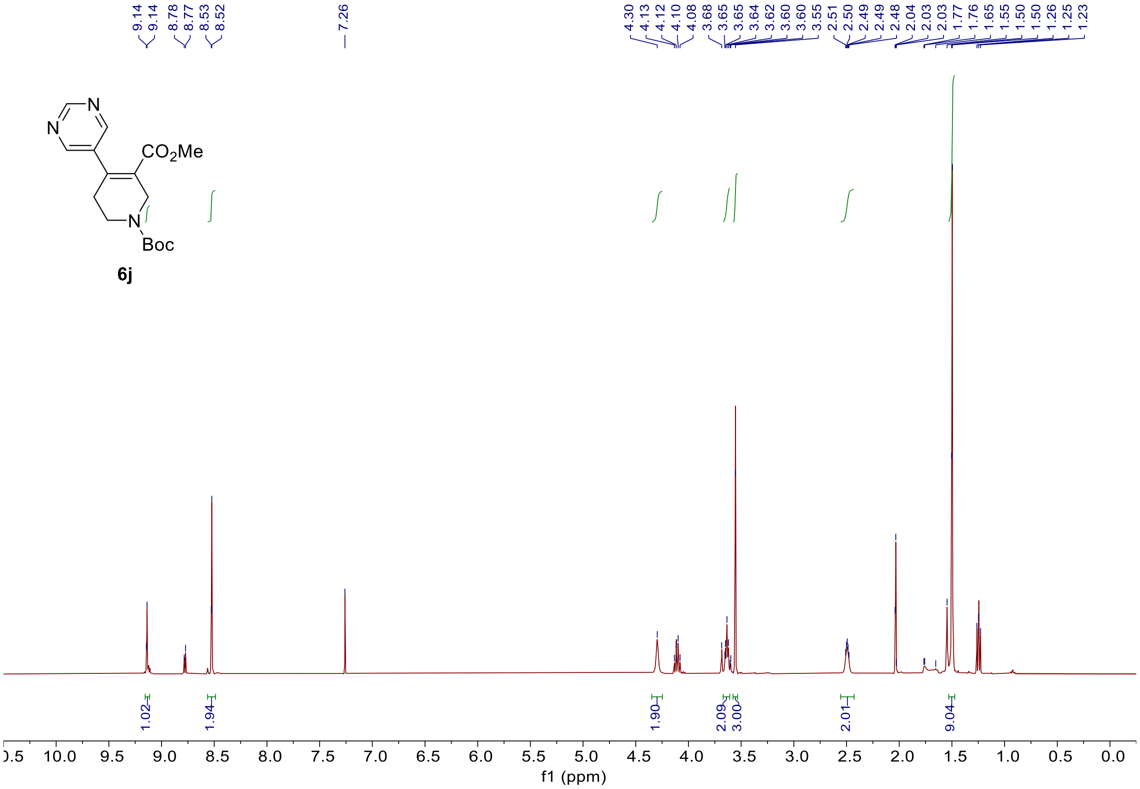

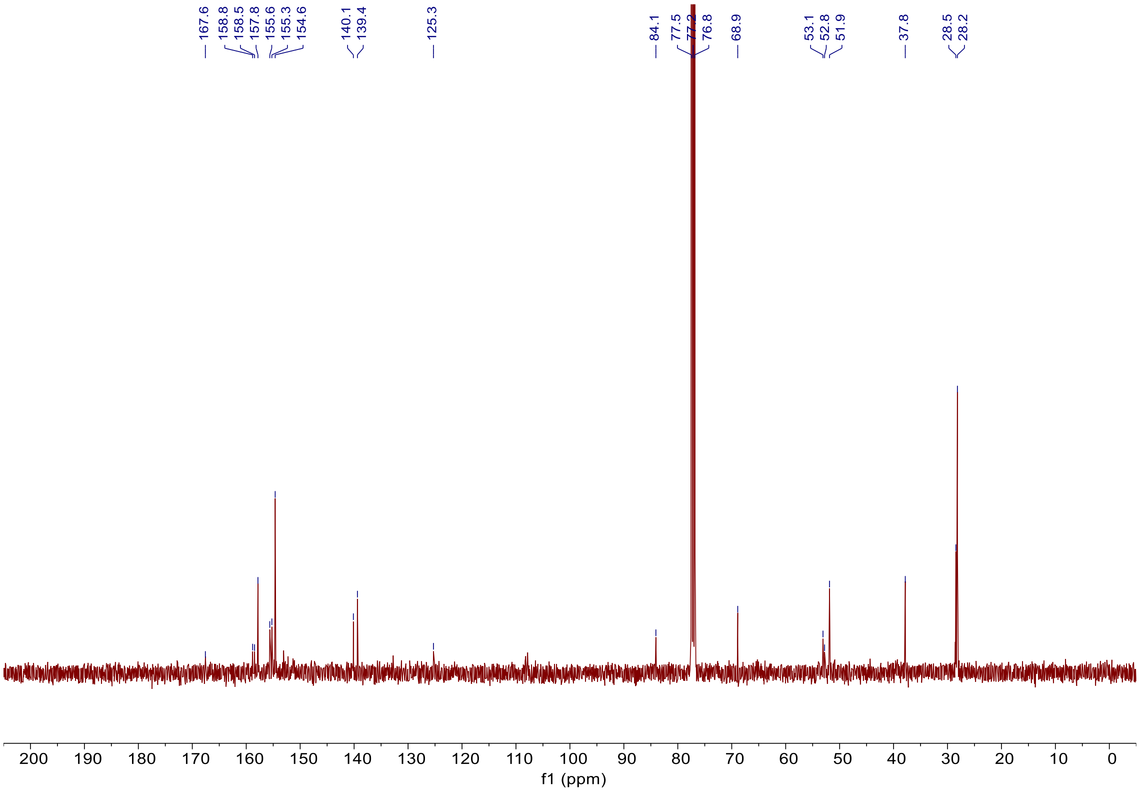

400 MHz ^{1}H NMR spectrum; 100.6 MHz ^{13}C NMR spectrum; $CDCl_3$ of **6k**

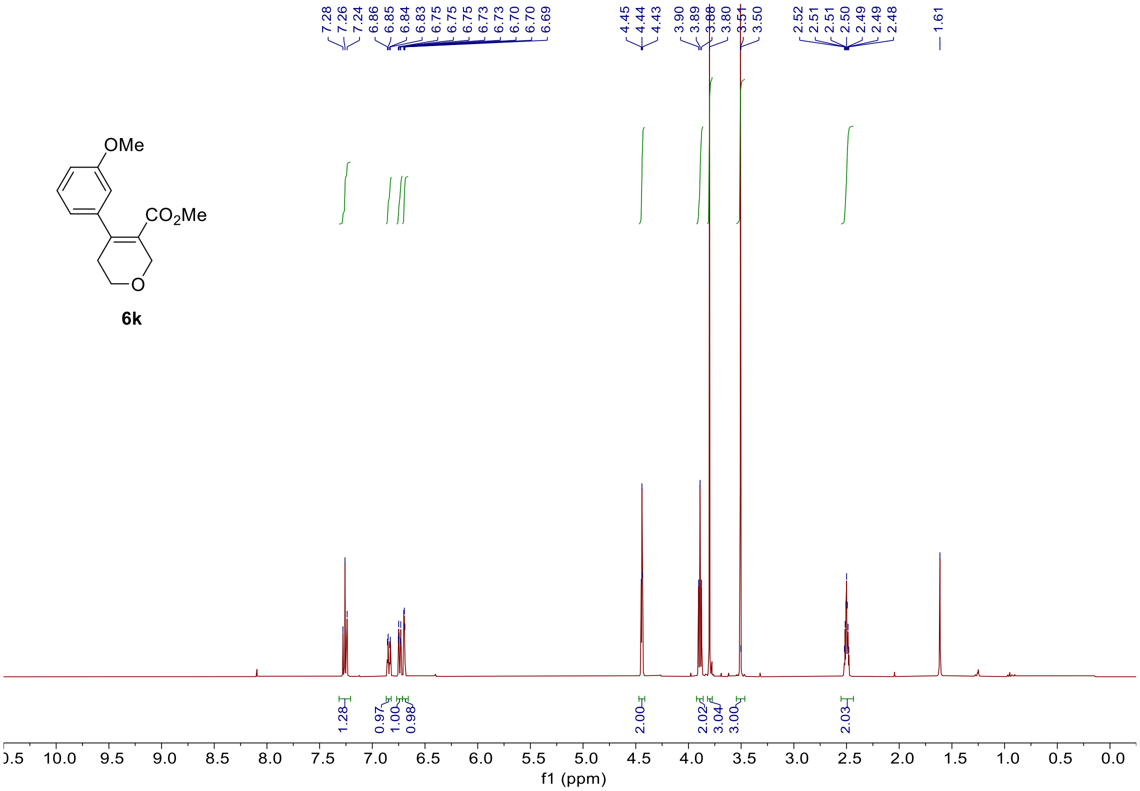

6k

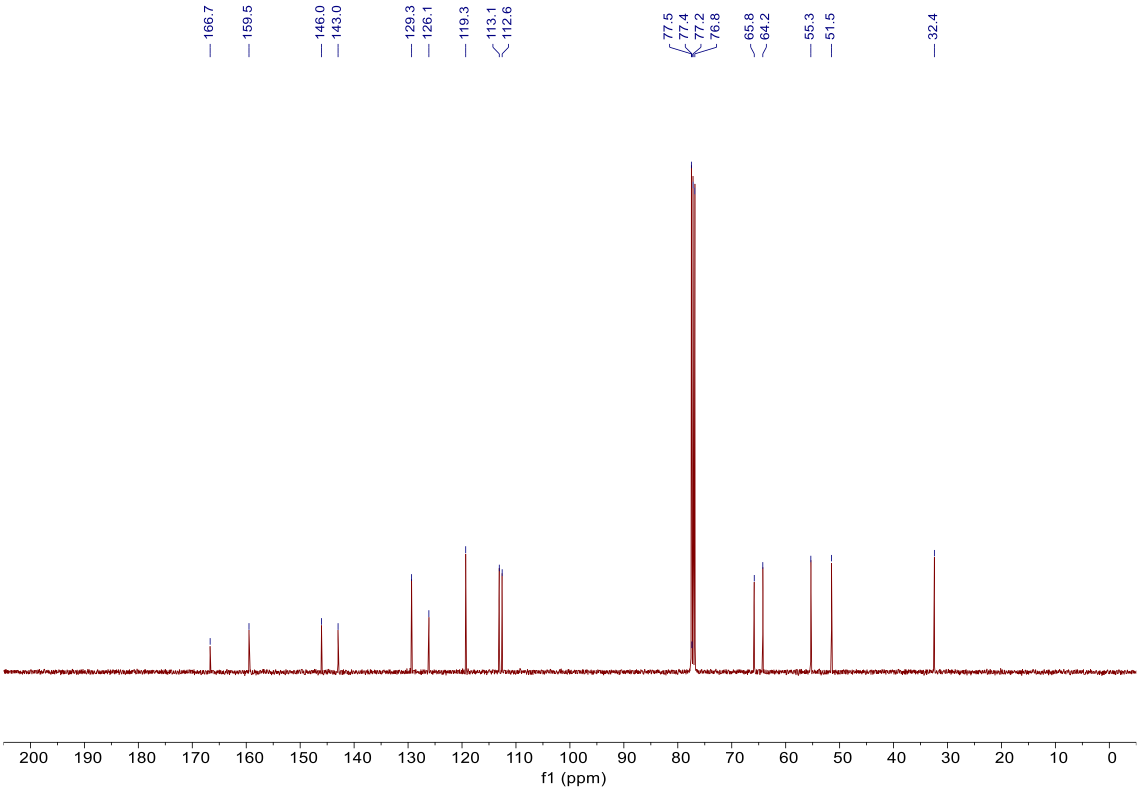

400 MHz ^{1}H NMR spectrum; 100.6 MHz ^{13}C NMR spectrum; $CDCl_3$ of **2a**

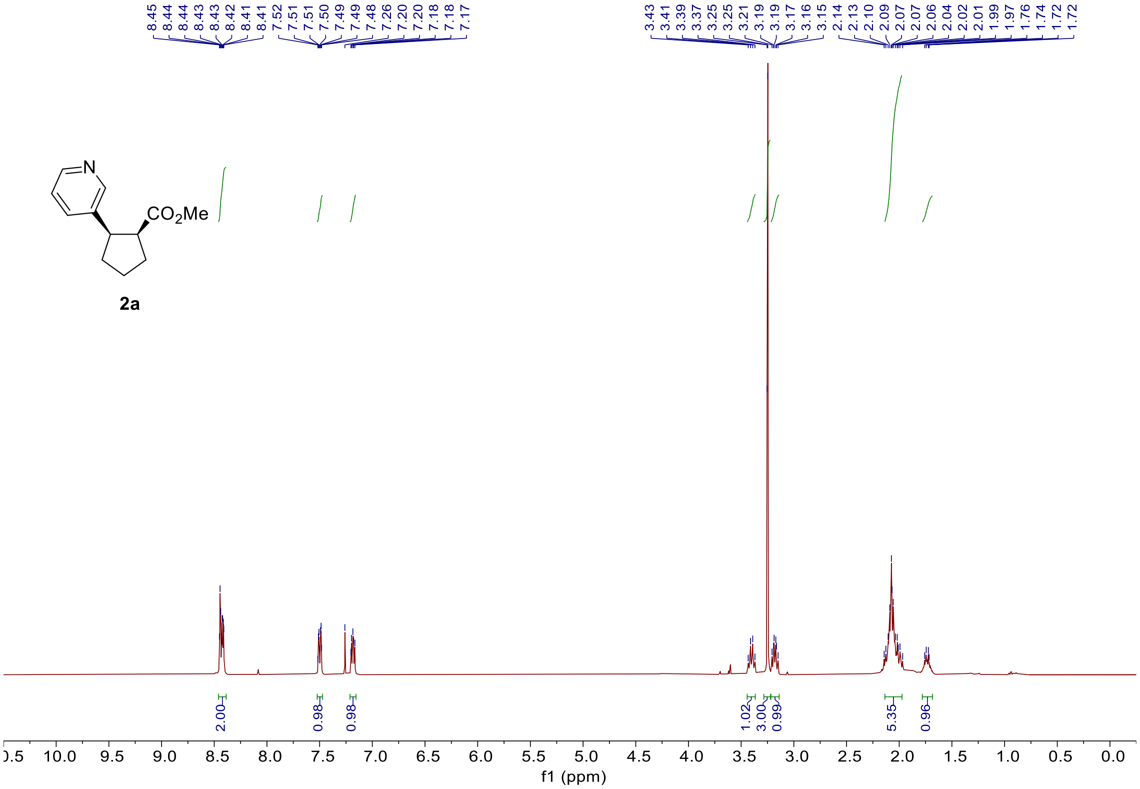

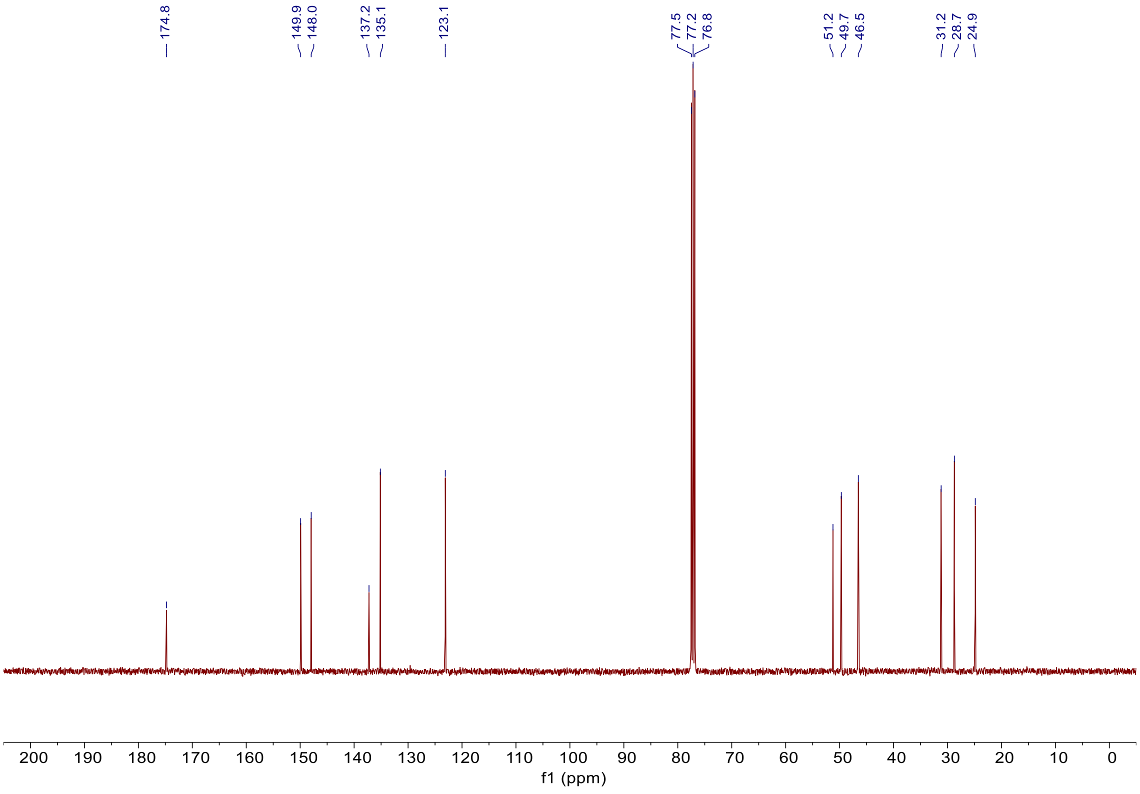

400 MHz ^{1}H NMR spectrum; 100.6 MHz ^{13}C NMR spectrum; $CDCl_3$ of **2b**

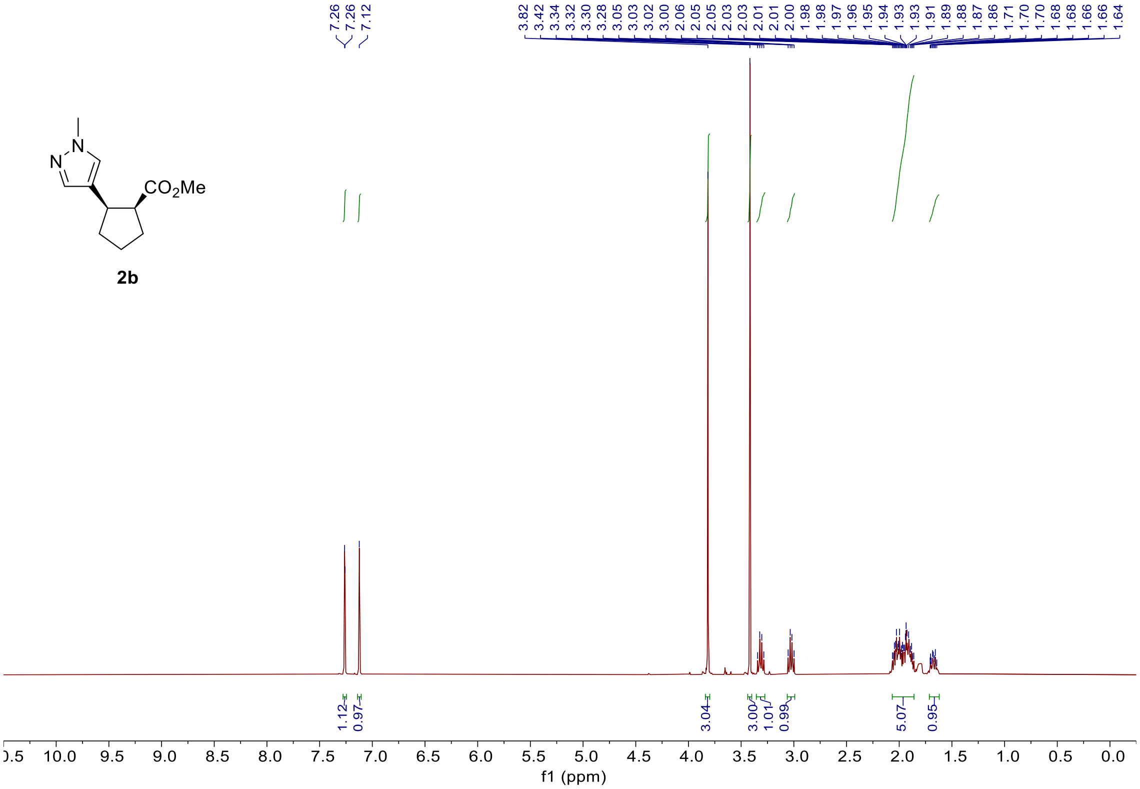

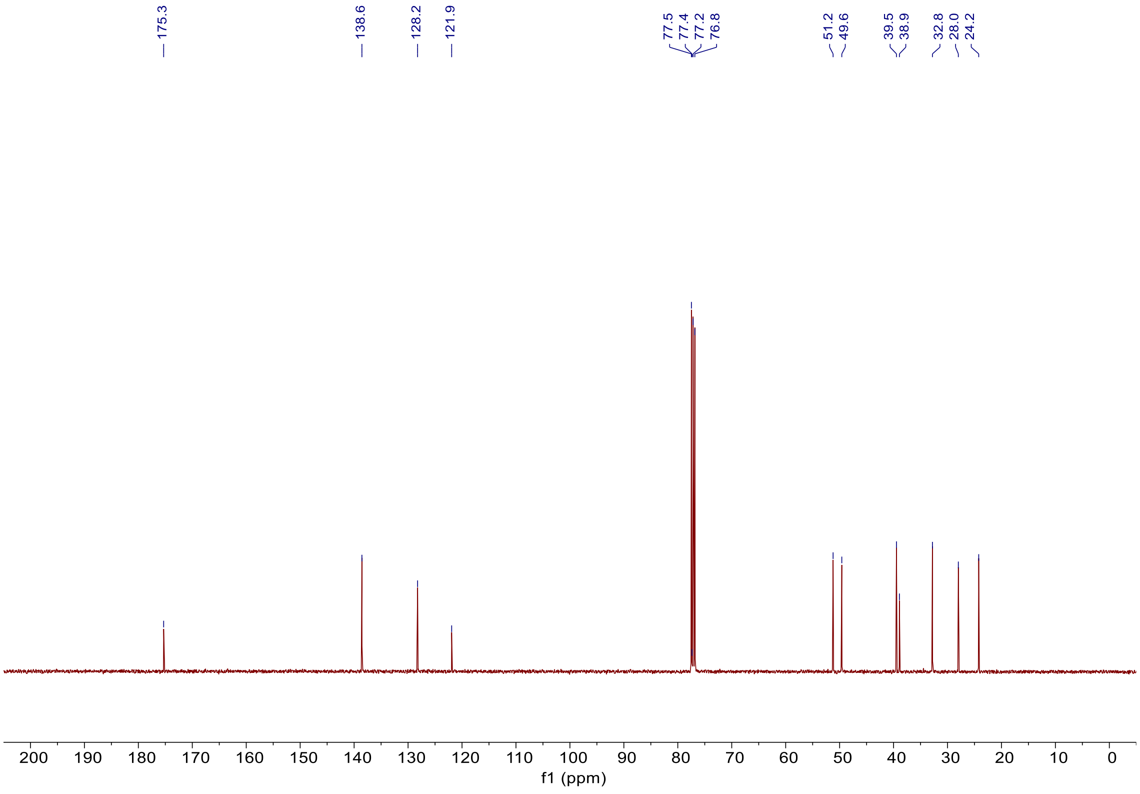

400 MHz ^{1}H NMR spectrum; 100.6 MHz ^{13}C NMR spectrum; $CDCl_3$ of **2c**

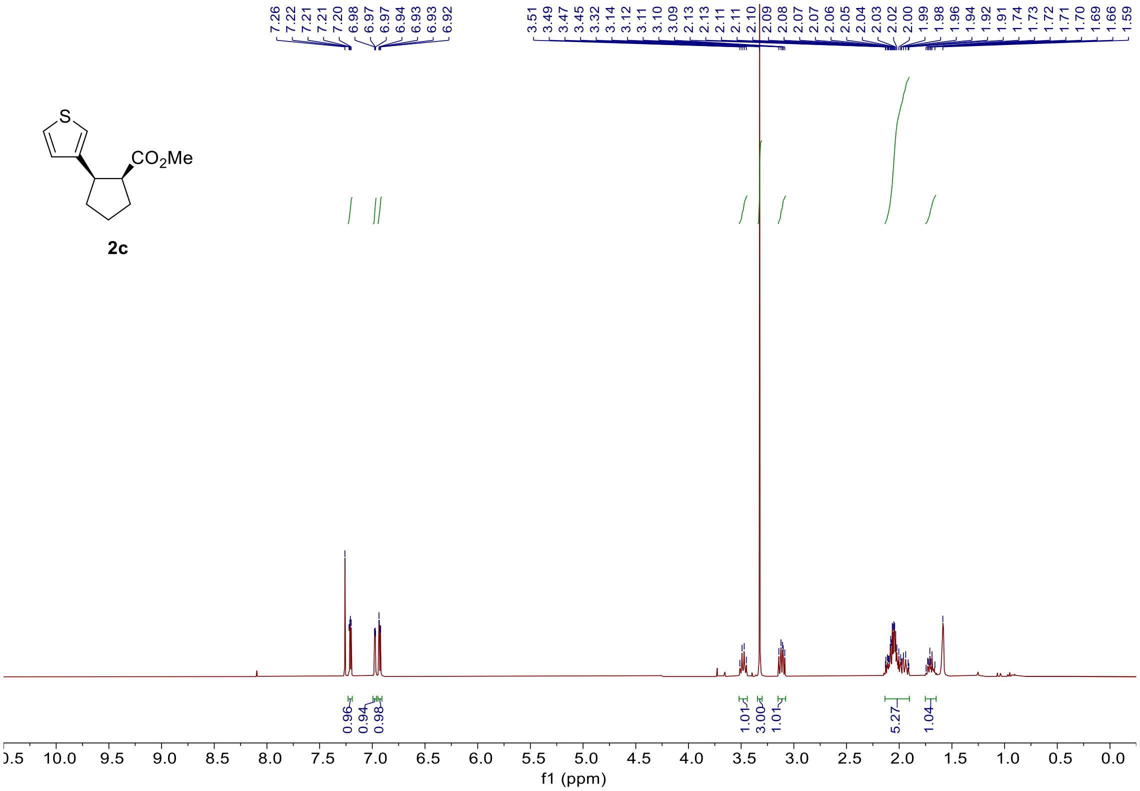

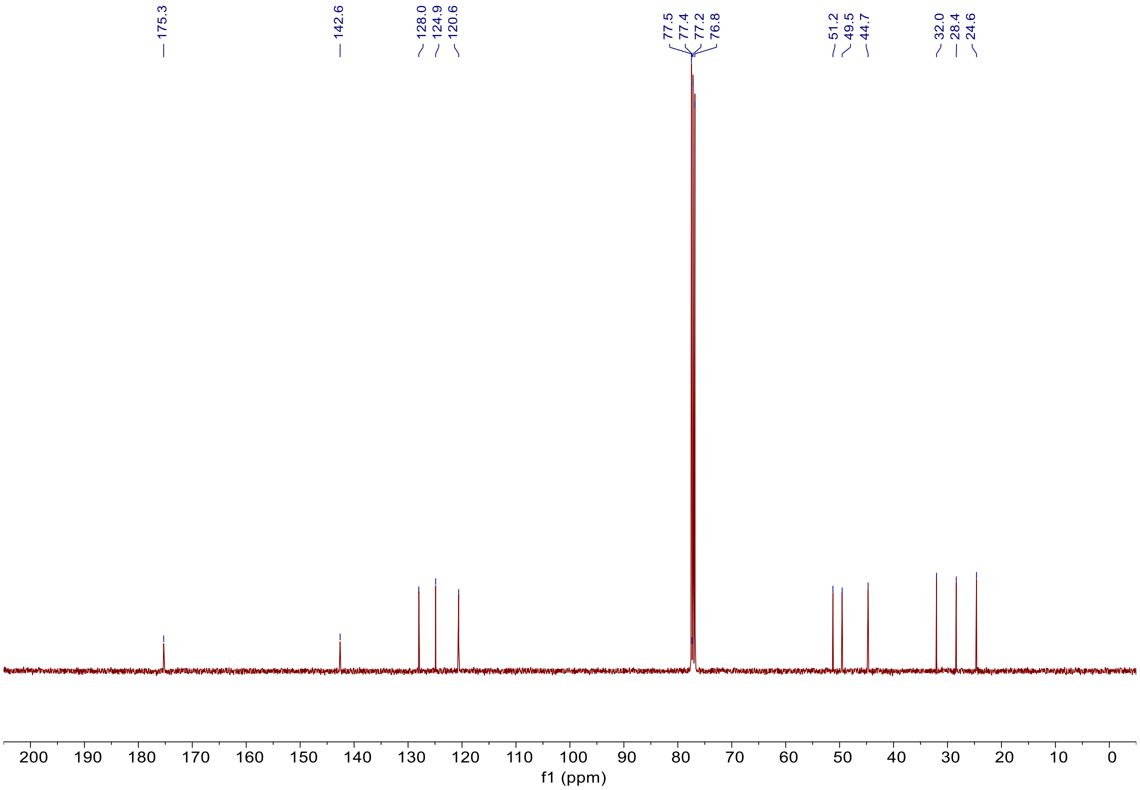

400 MHz ^{1}H NMR spectrum; 100.6 MHz ^{13}C NMR spectrum; $CDCl_3$ of **2d**

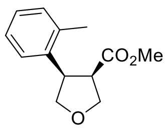

2d

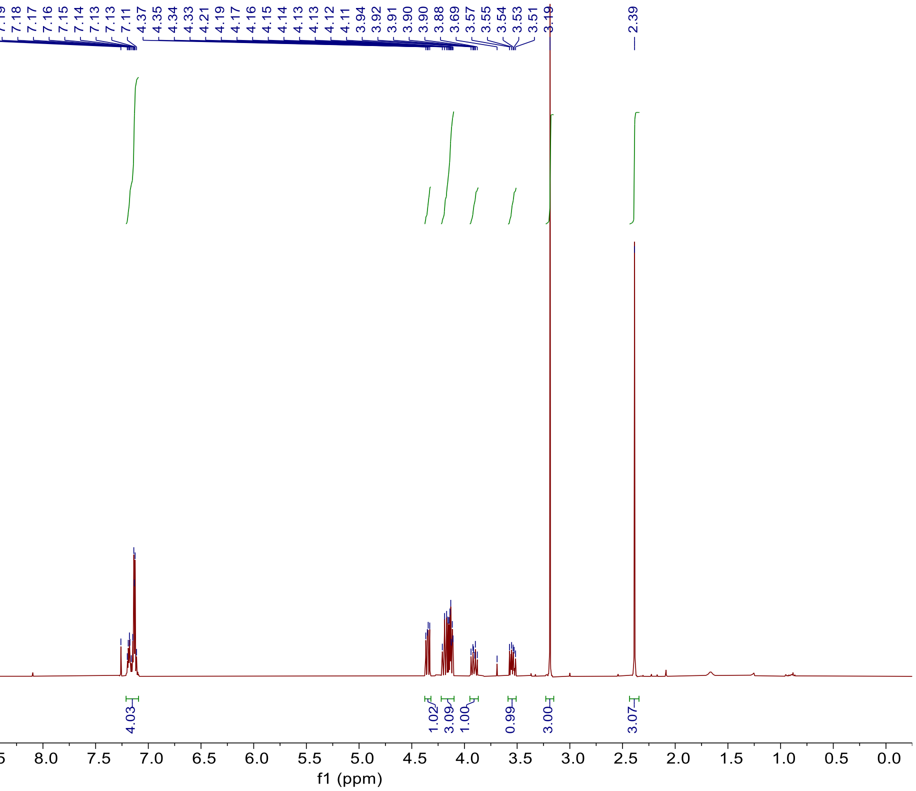

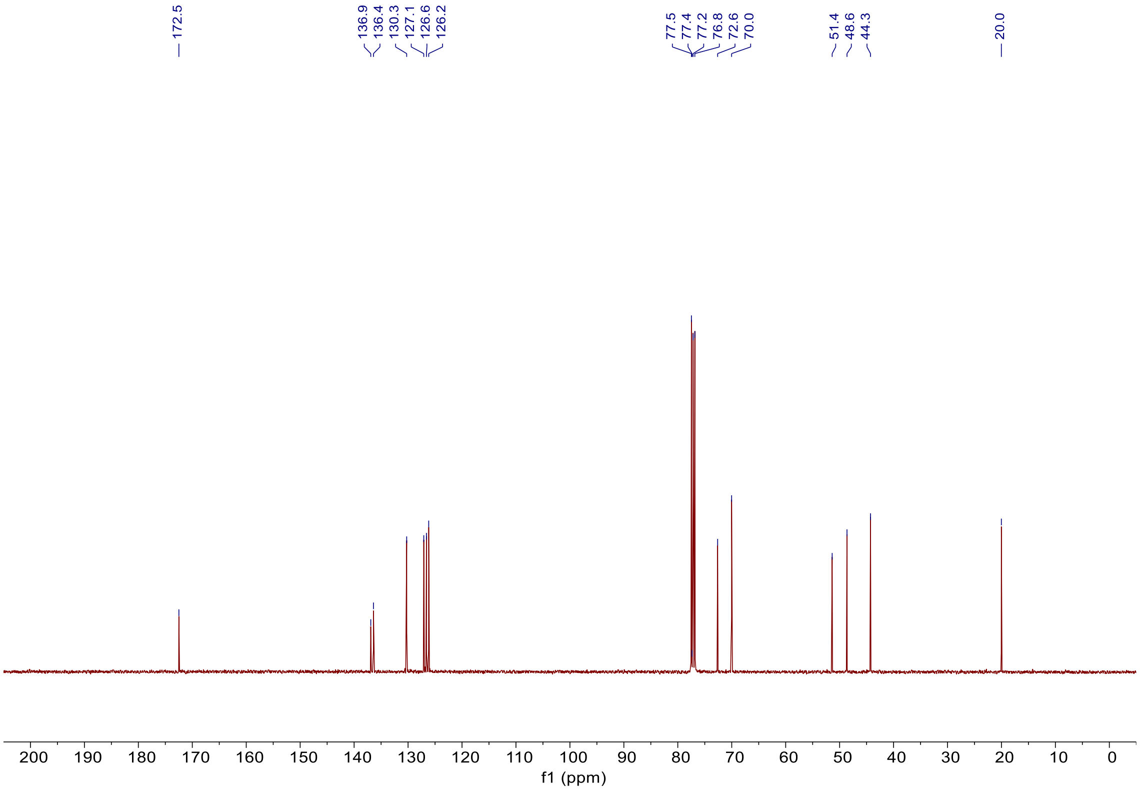

400 MHz ^{1}H NMR spectrum; 100.6 MHz ^{13}C NMR spectrum; $CDCl_3$ of **2f**

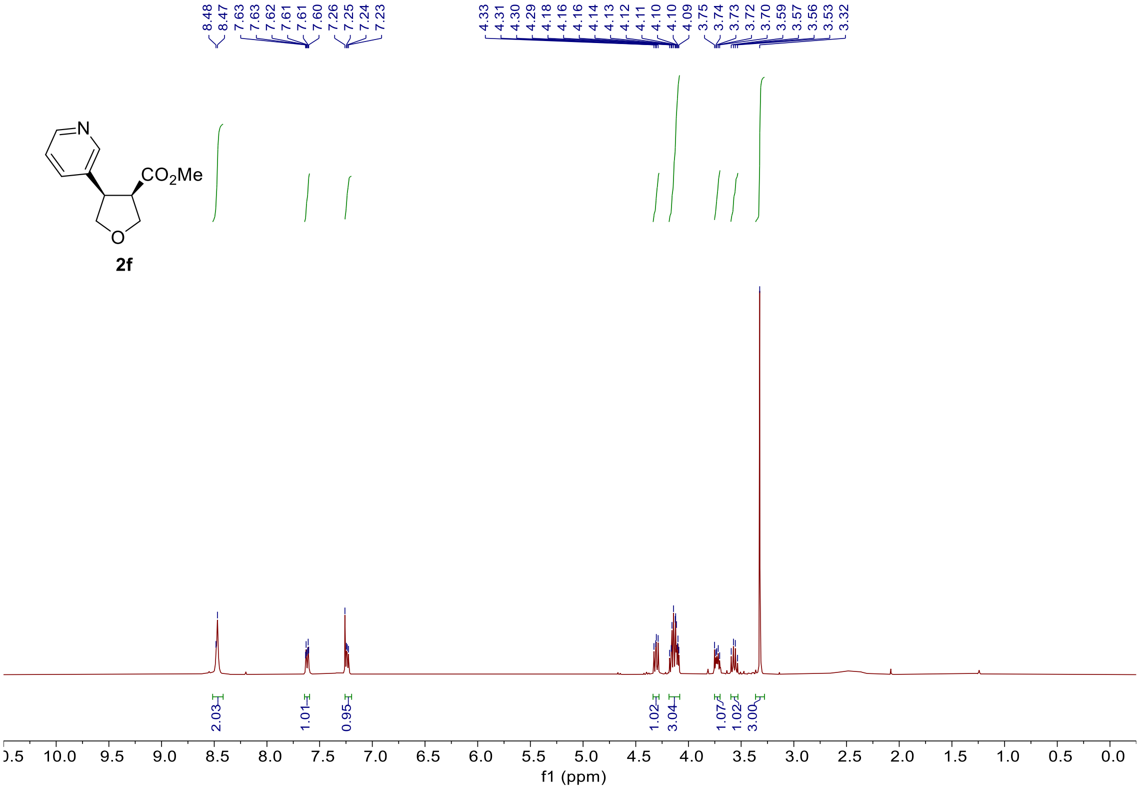

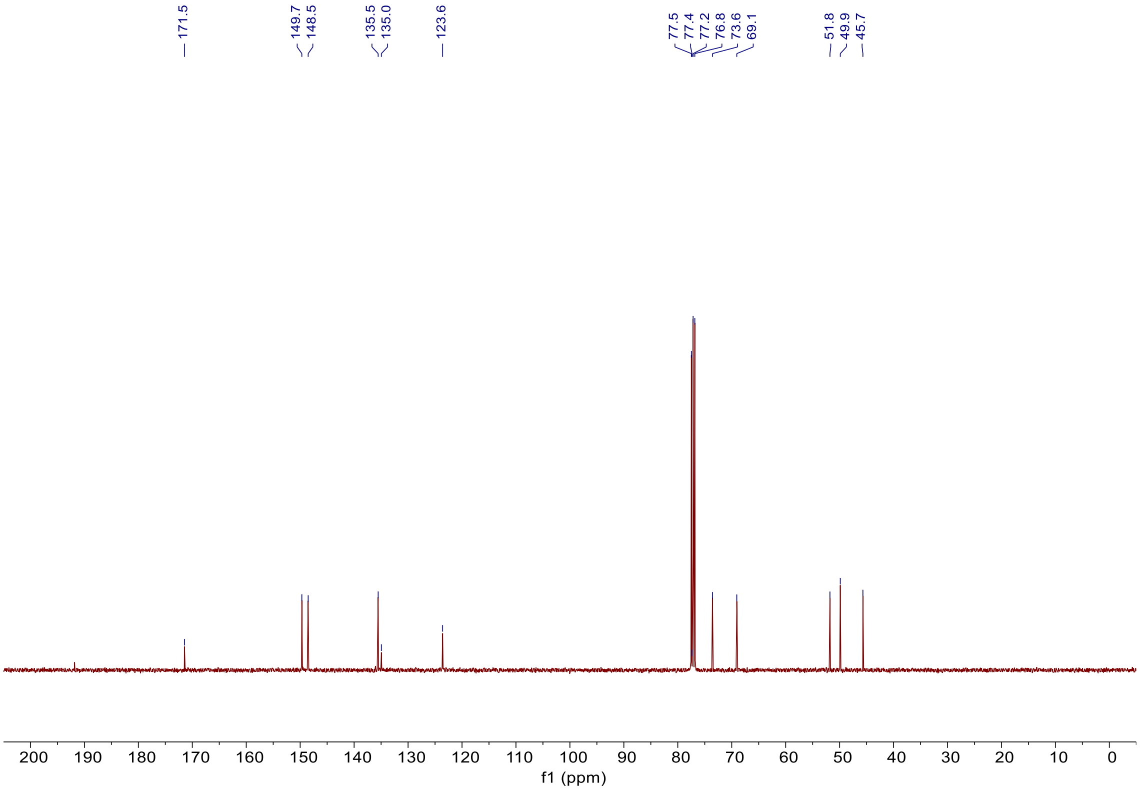

400 MHz ^{1}H NMR spectrum; $CDCl_3$ of 60:40 **2g** and 2-fluorophenylboronic acid

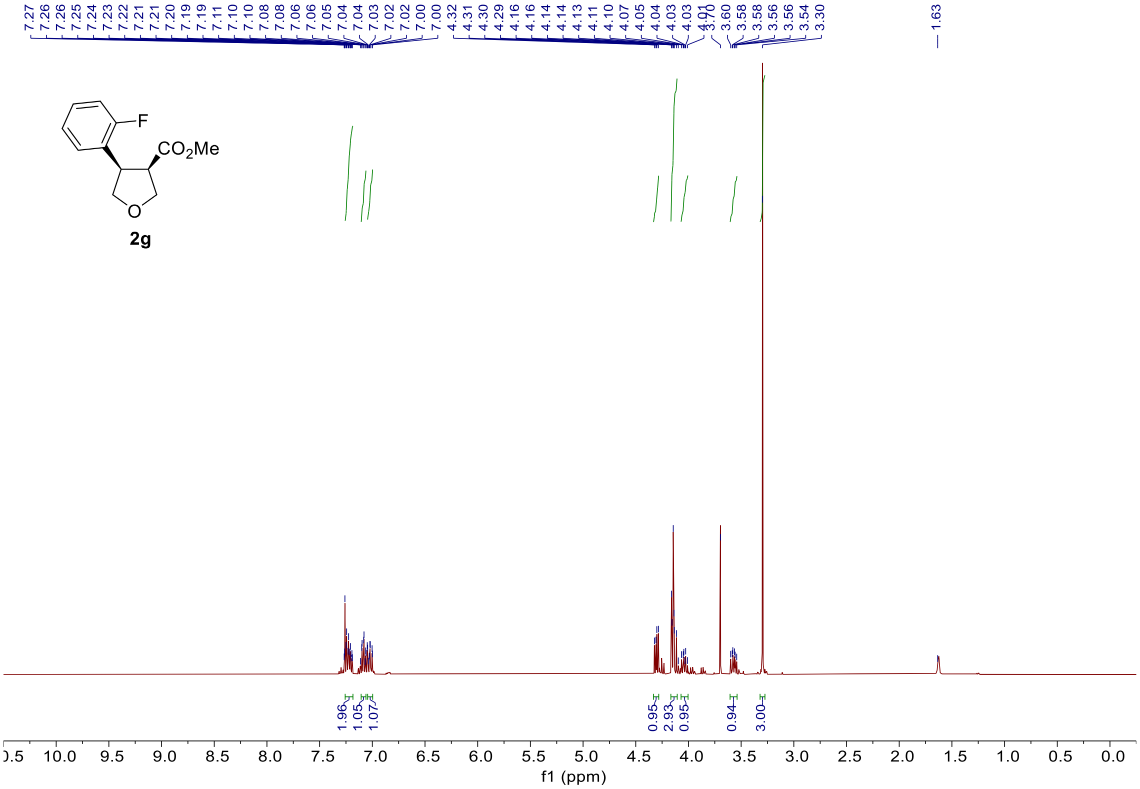

400 MHz ^{1}H NMR spectrum; 100.6 MHz ^{13}C NMR spectrum; $CDCl_3$ of **2h**

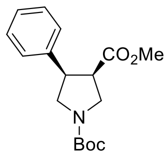

2h

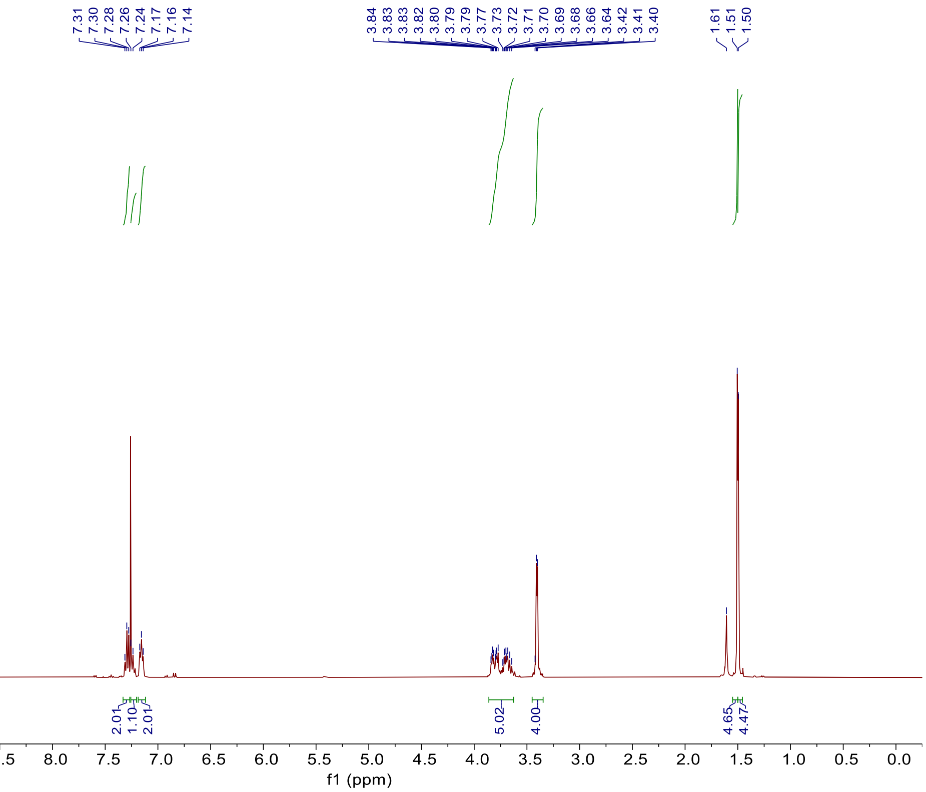

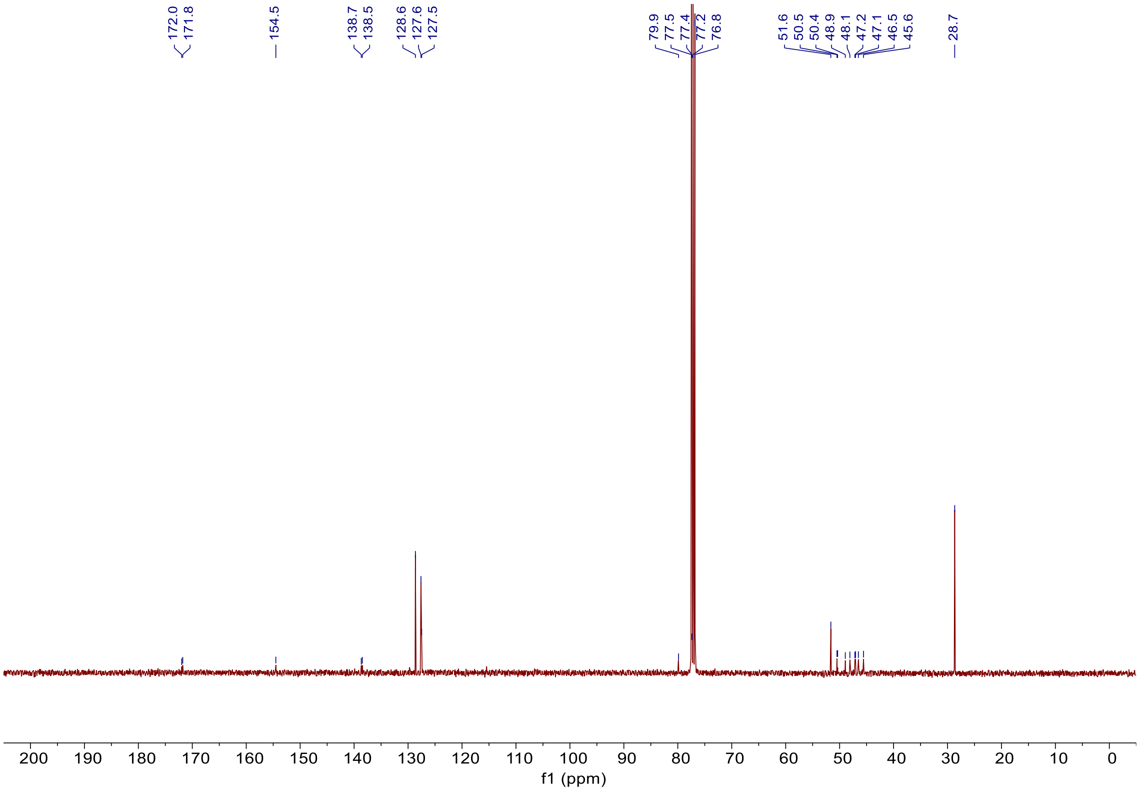

400 MHz ^{1}H NMR spectrum; 100.6 MHz ^{13}C NMR spectrum; $CDCl_3$ of **2i**

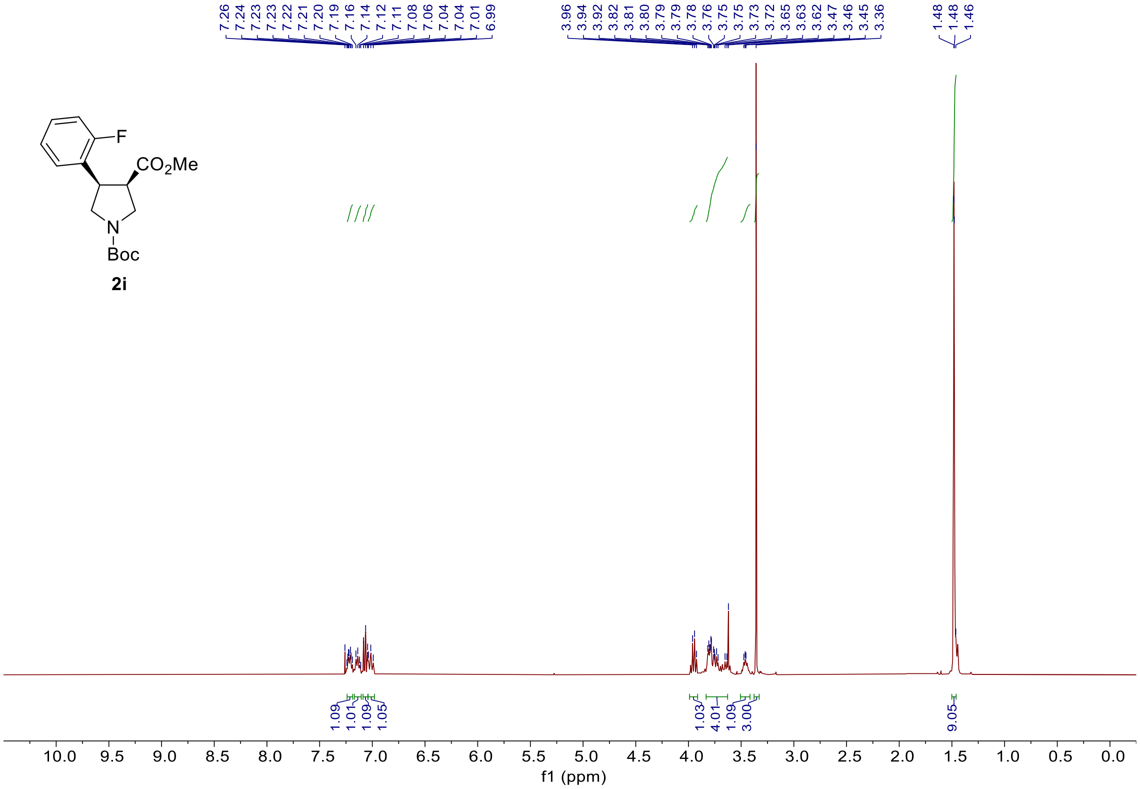

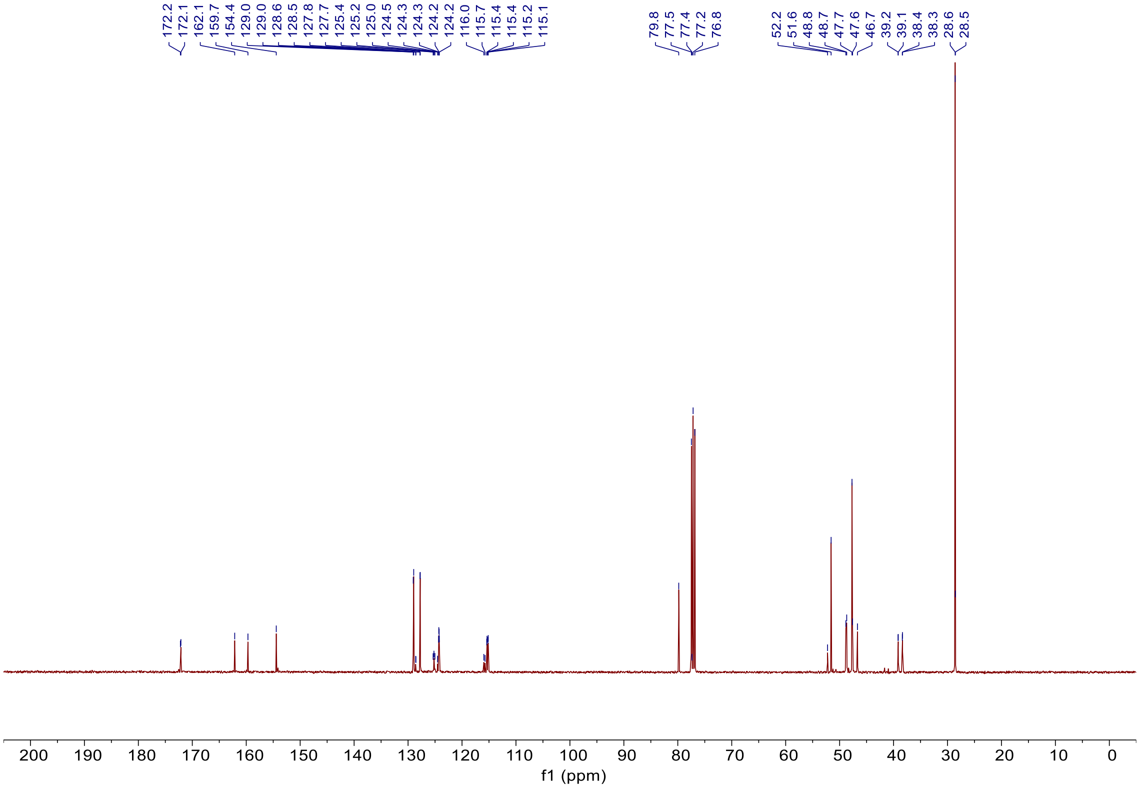

400 MHz ^{1}H NMR spectrum; 100.6 MHz ^{13}C NMR spectrum; $CDCl_3$ of **2j**

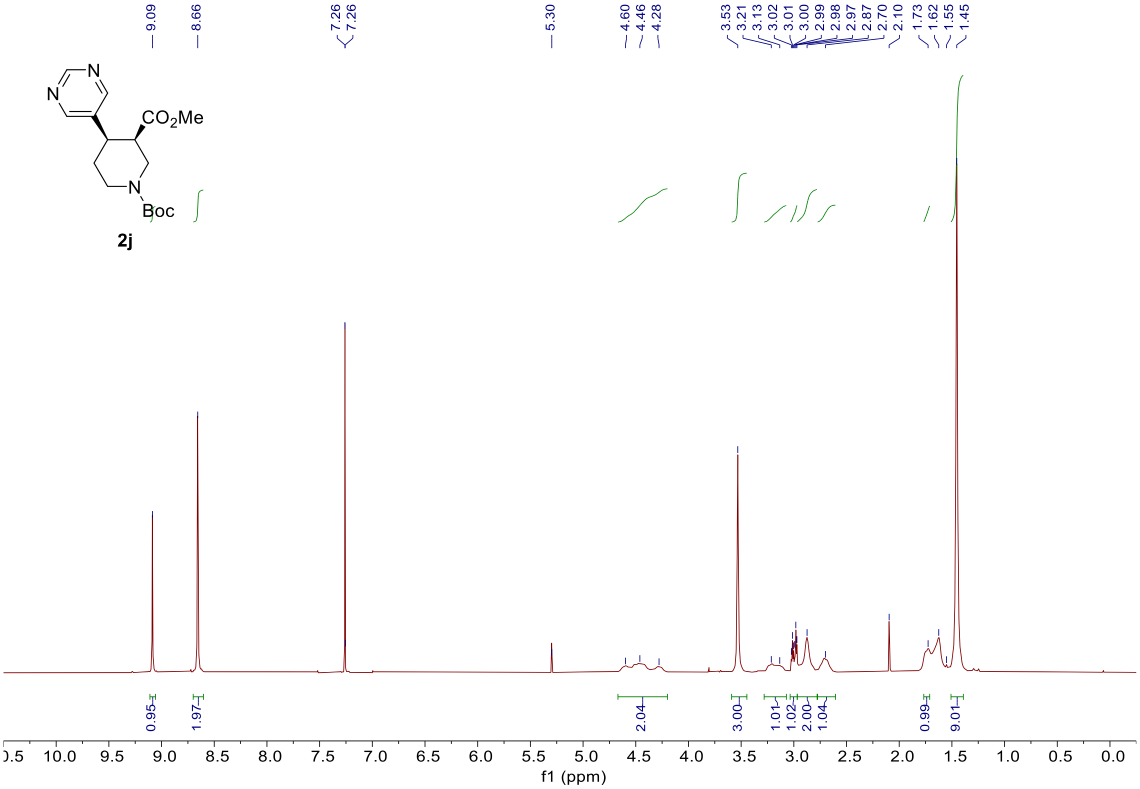

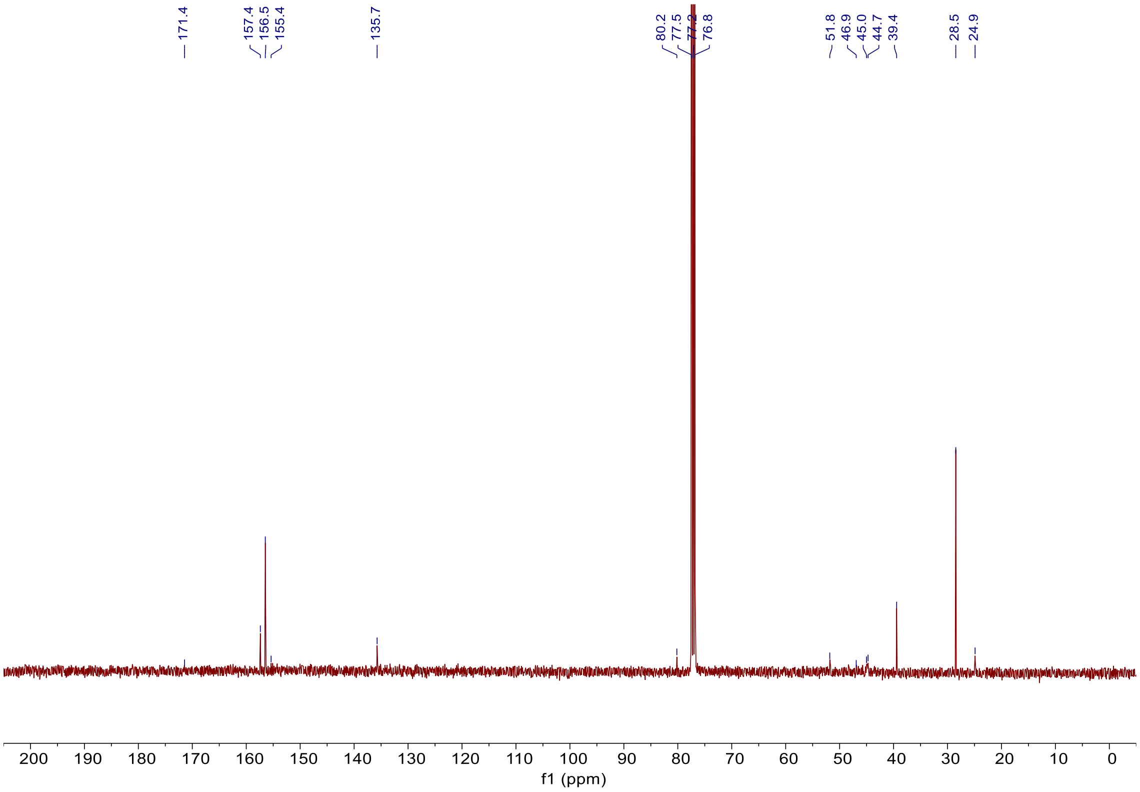

400 MHz ^{1}H NMR spectrum; 100.6 MHz ^{13}C NMR spectrum; $CDCl_3$ of **2k**

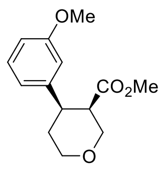

2k

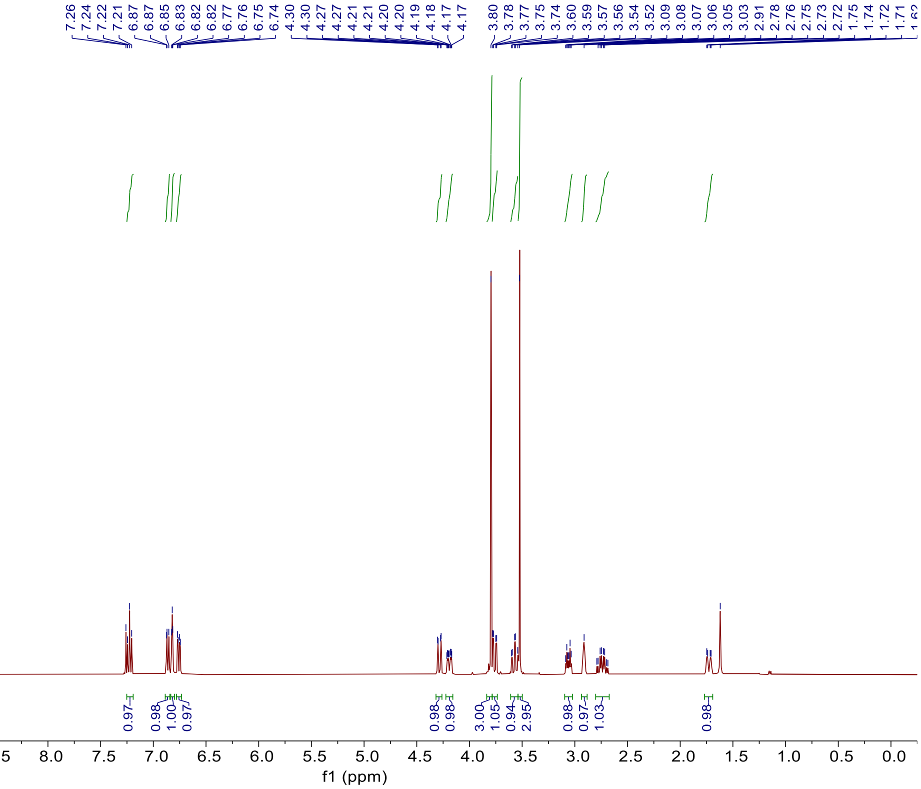

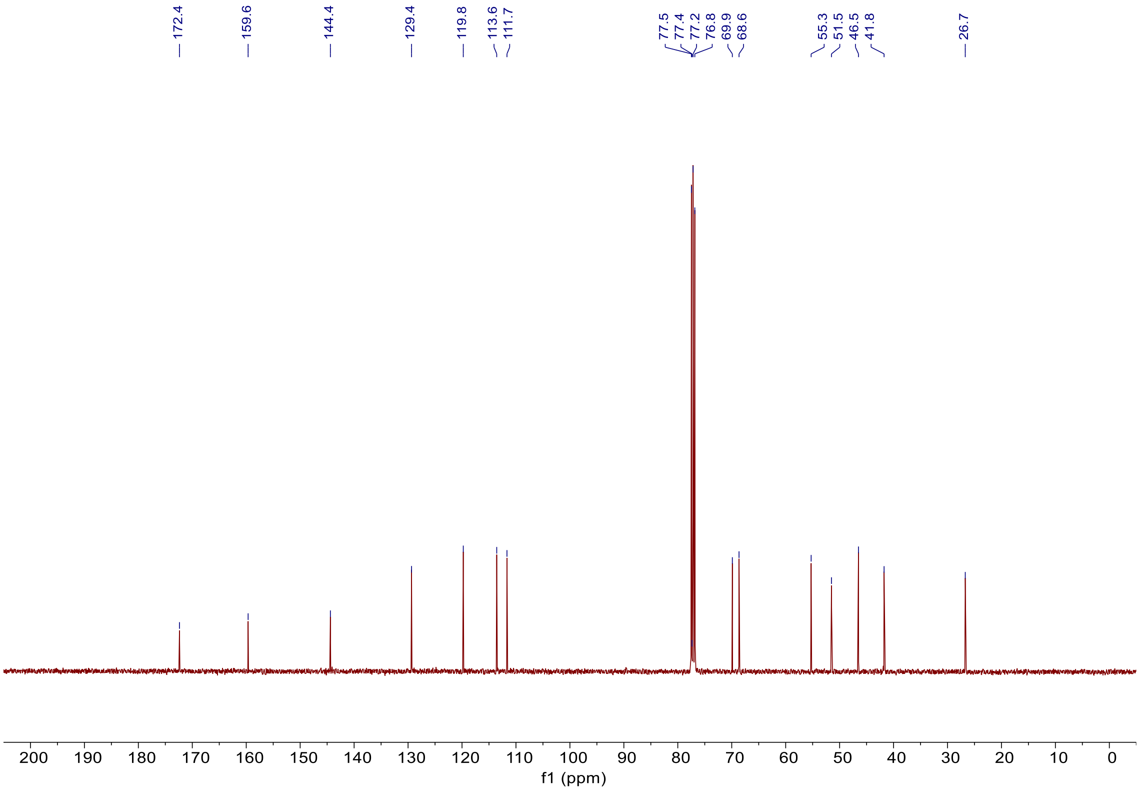

400 MHz ^{1}H NMR spectrum; 100.6 MHz ^{13}C NMR spectrum; $CDCl_3$ of **S6**

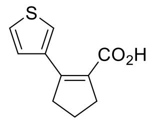

S6

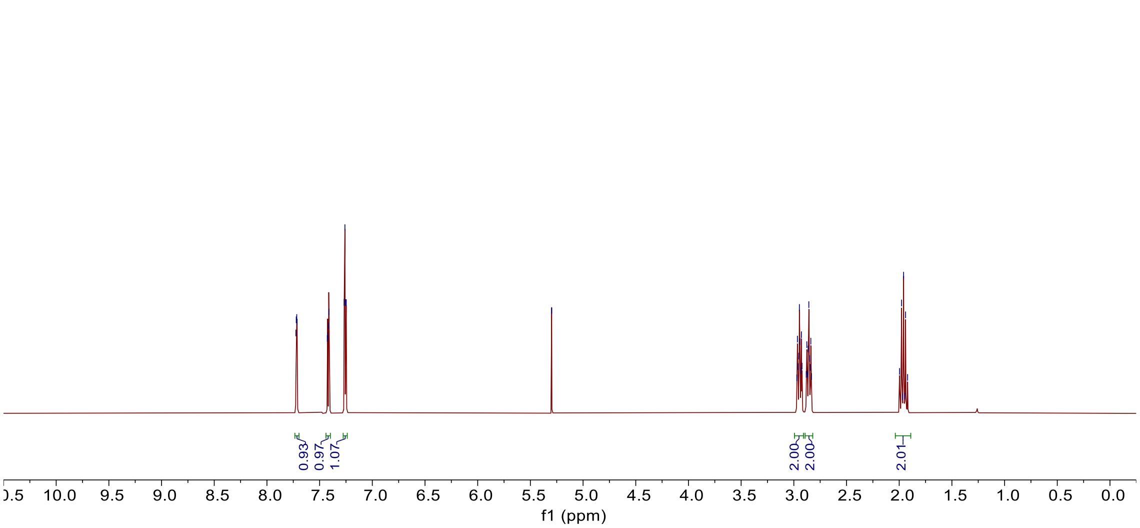

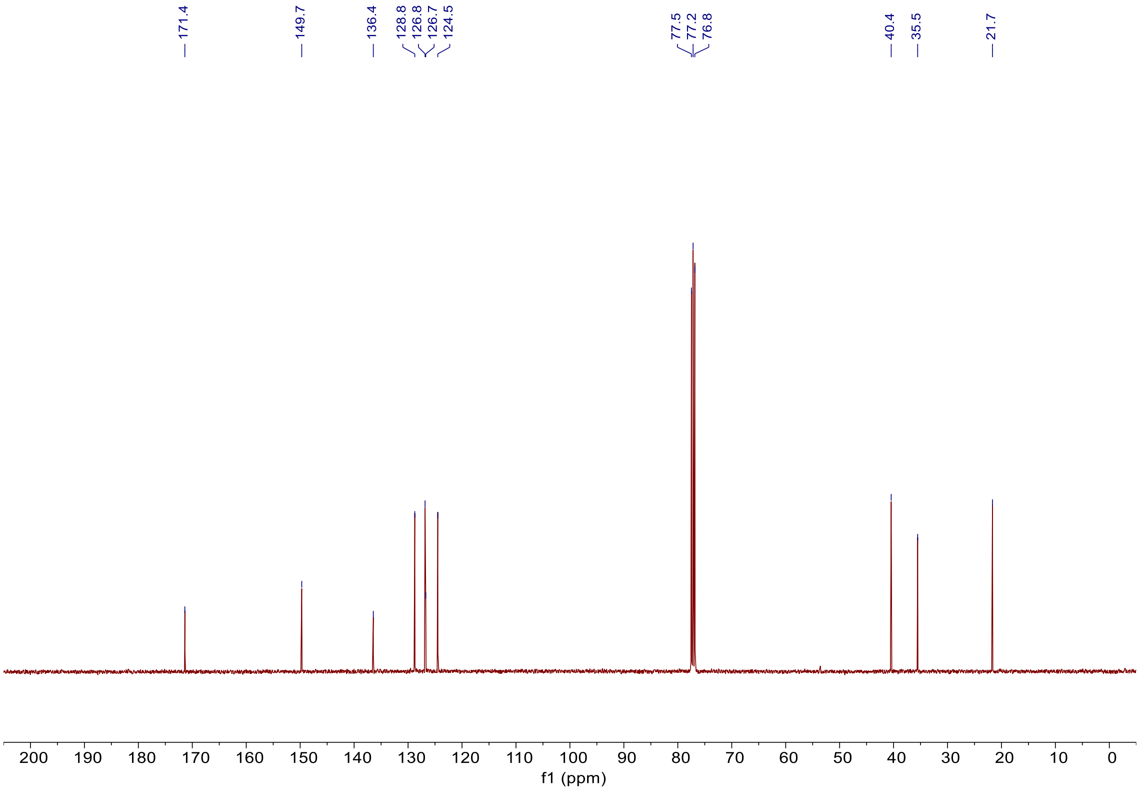

400 MHz ^{1}H NMR spectrum; 100.6 MHz ^{13}C NMR spectrum; $CDCl_3$ of **7c**

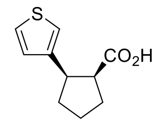

7c

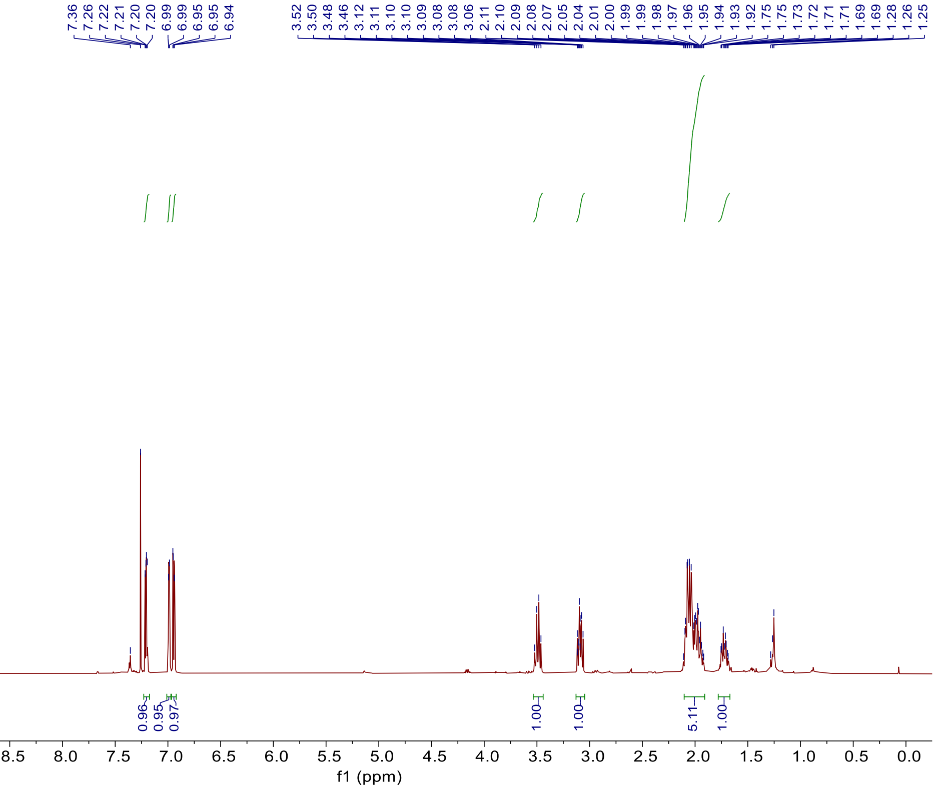

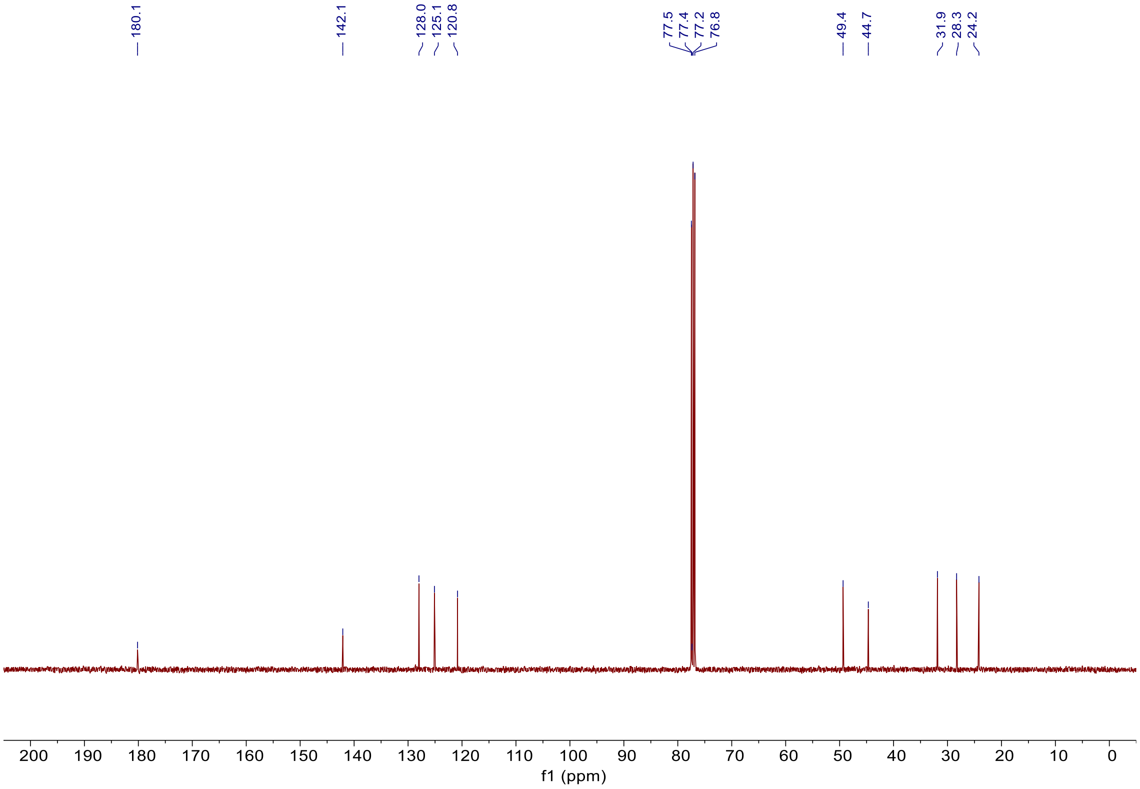

400 MHz ^{1}H NMR spectrum; 100.6 MHz ^{13}C NMR spectrum; $CDCl_3$ of **S7**

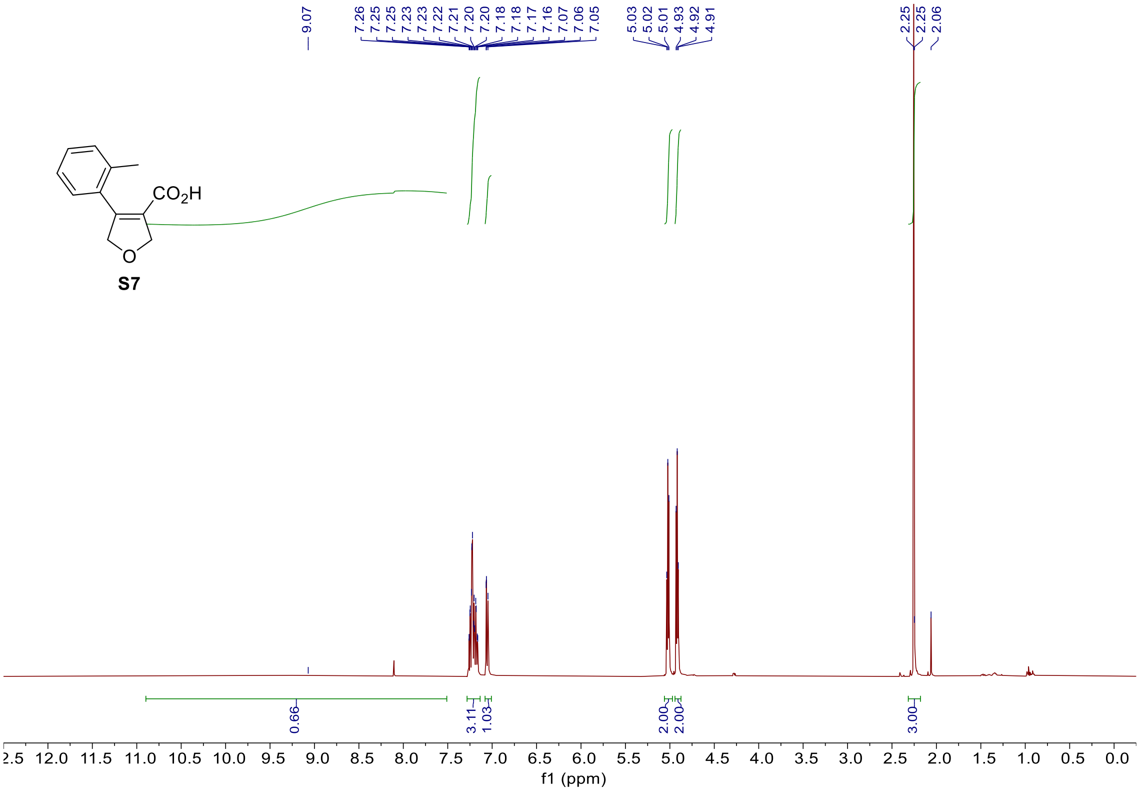

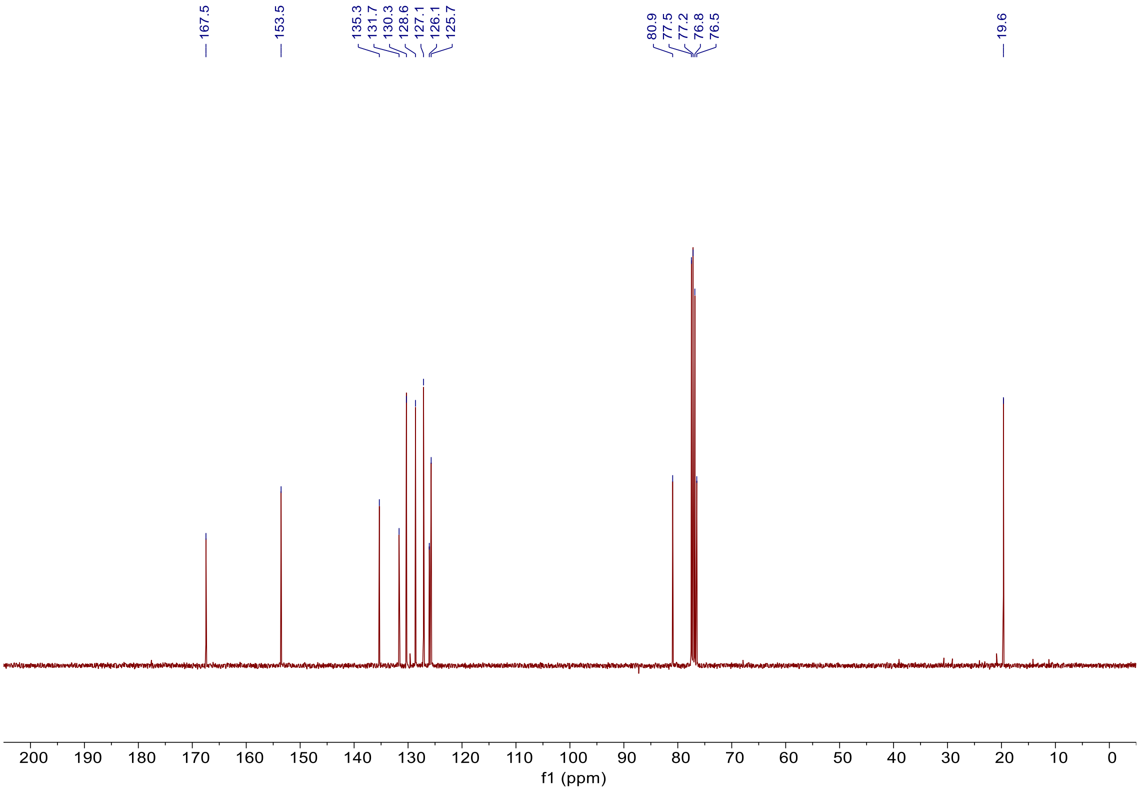

400 MHz ^{1}H NMR spectrum; 100.6 MHz ^{13}C NMR spectrum; $CDCl_3$ of **7d**

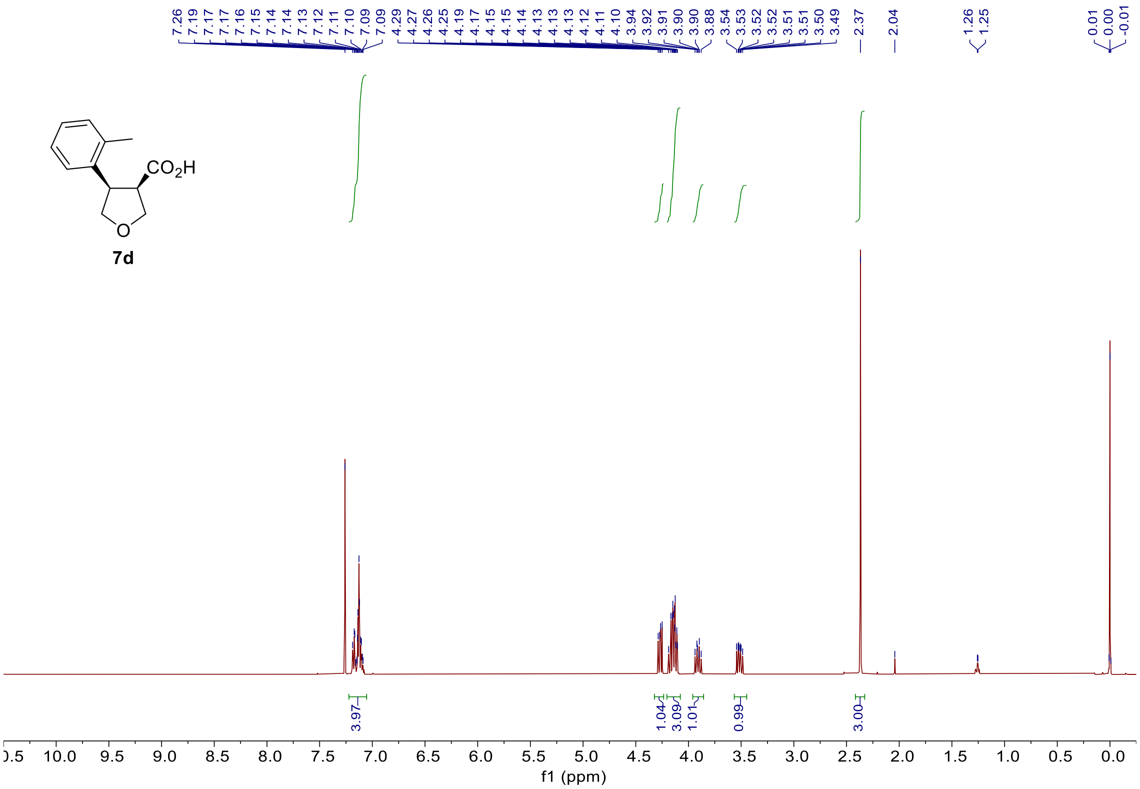

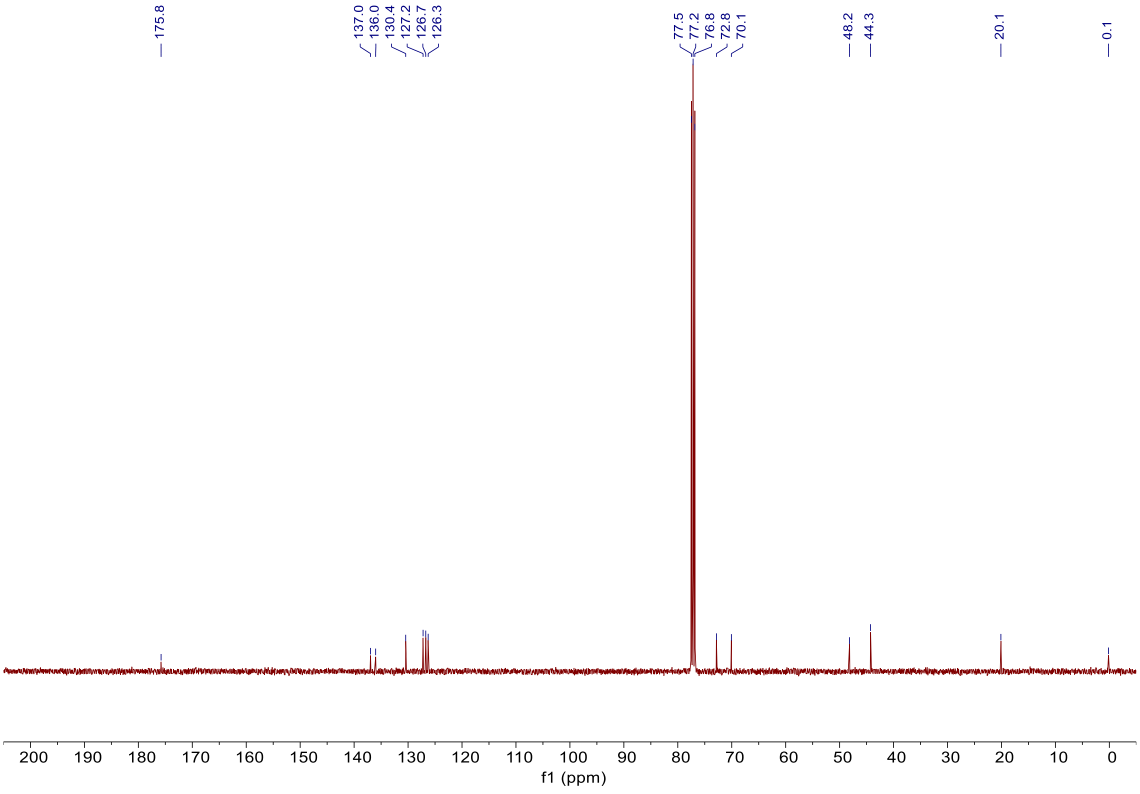

400 MHz ¹H NMR spectrum; 100.6 MHz ¹³C NMR spectrum; $CDCl_3$ of **S8**

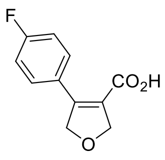

S8

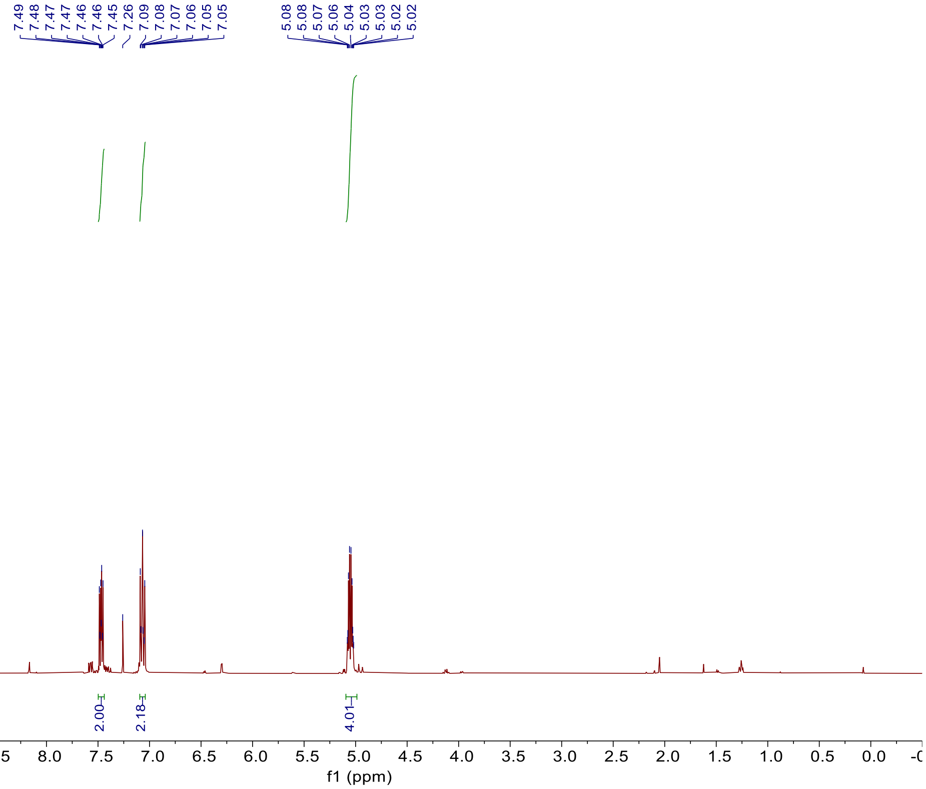

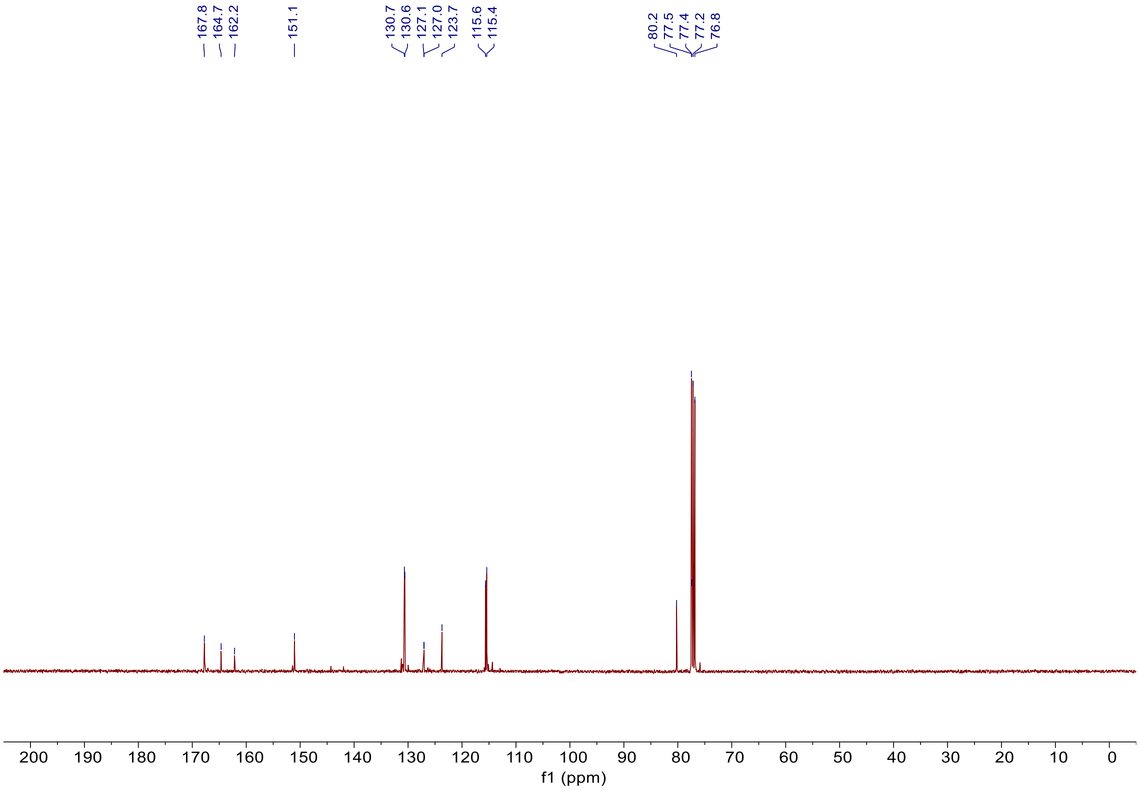

400 MHz ^{1}H NMR spectrum; 100.6 MHz ^{13}C NMR spectrum; $CDCl_3$ of **7e**

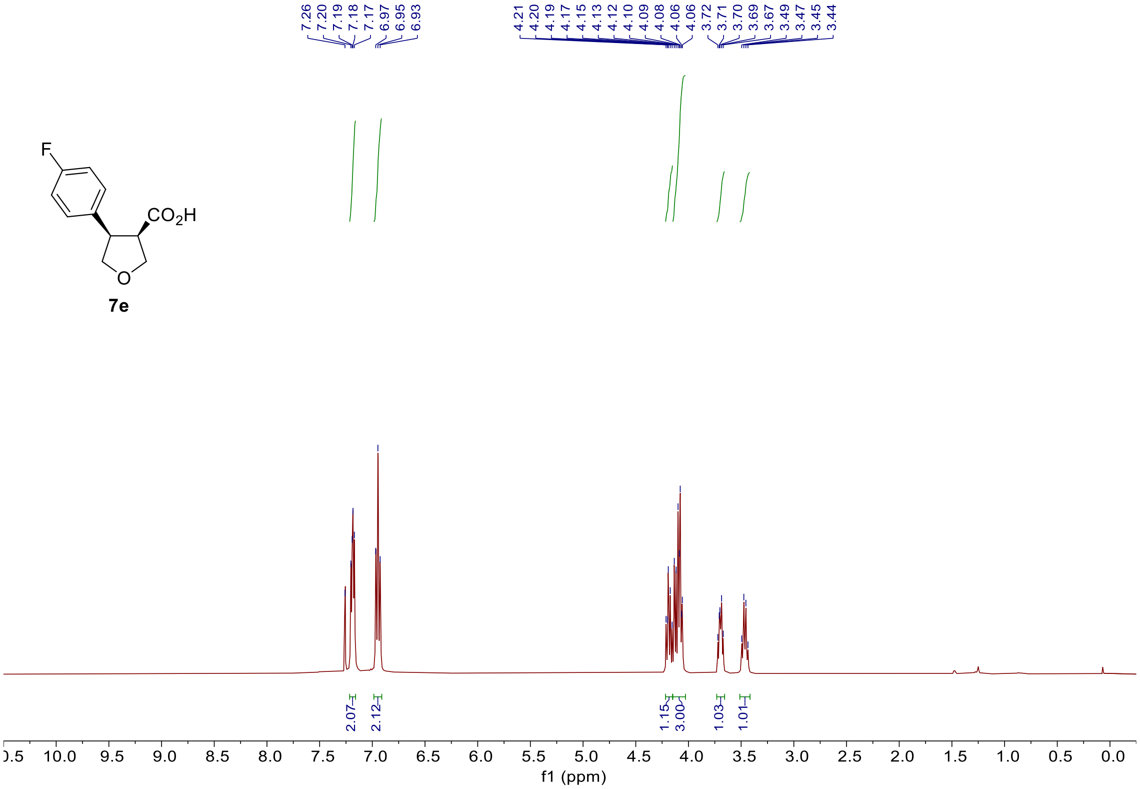

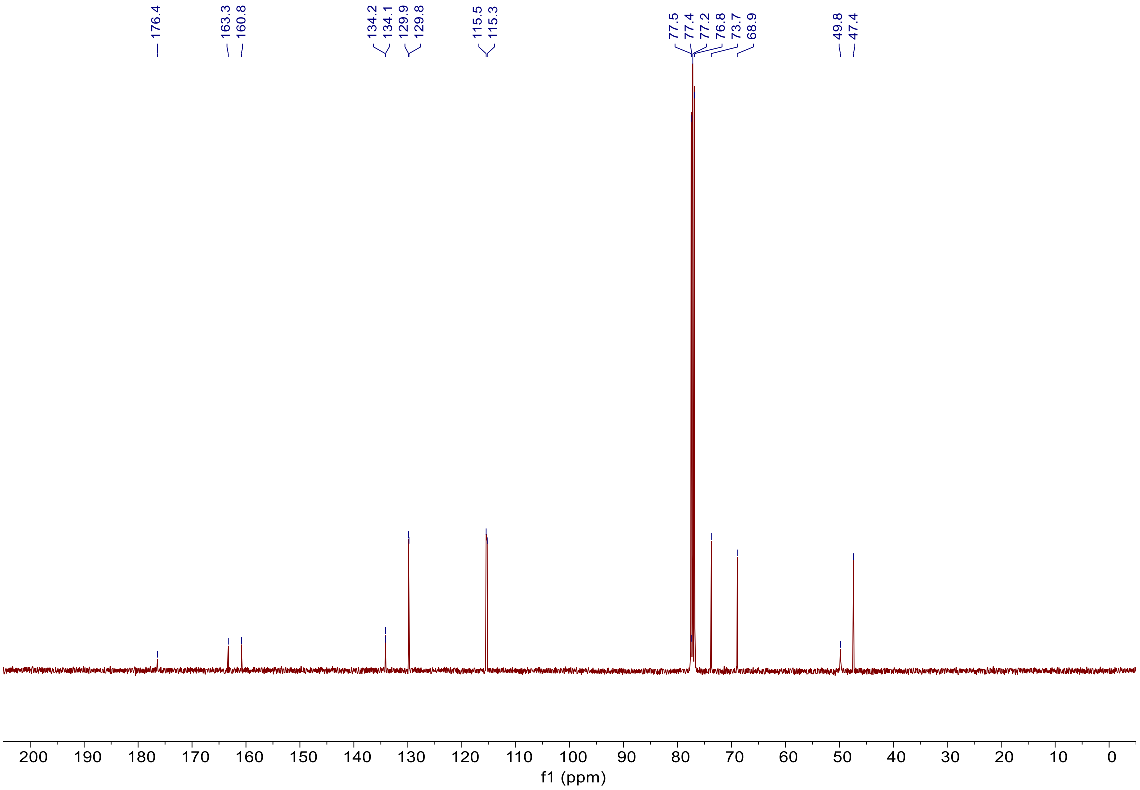

400 MHz ^{1}H NMR spectrum; 100.6 MHz ^{13}C NMR spectrum; $CDCl_3$ of **S9**

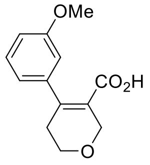

S9

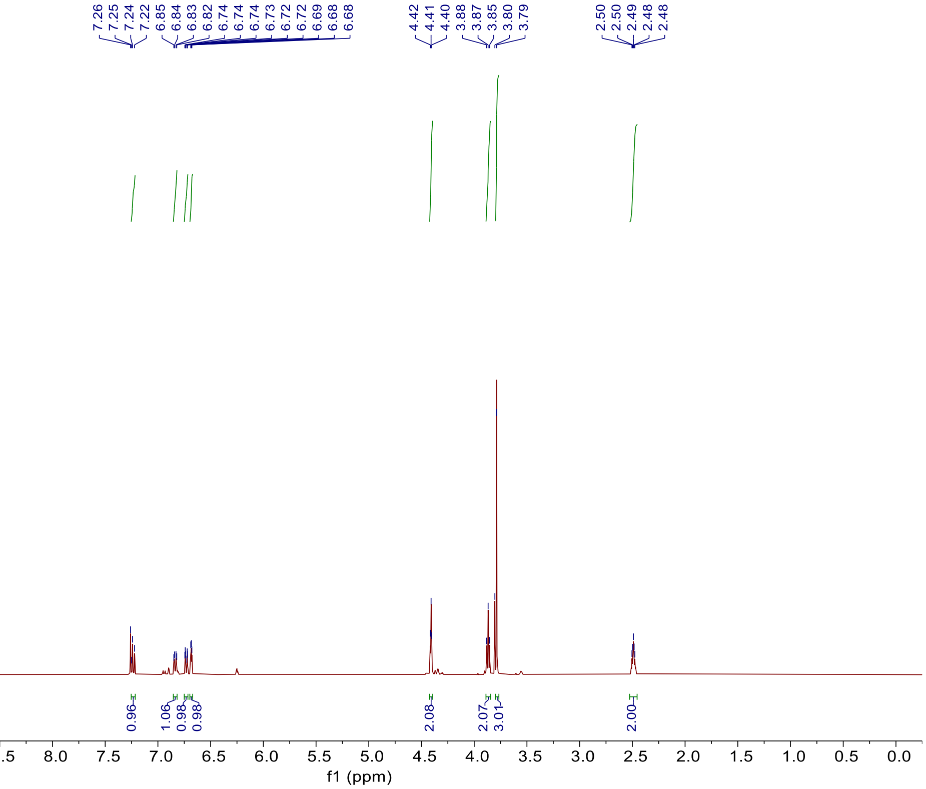

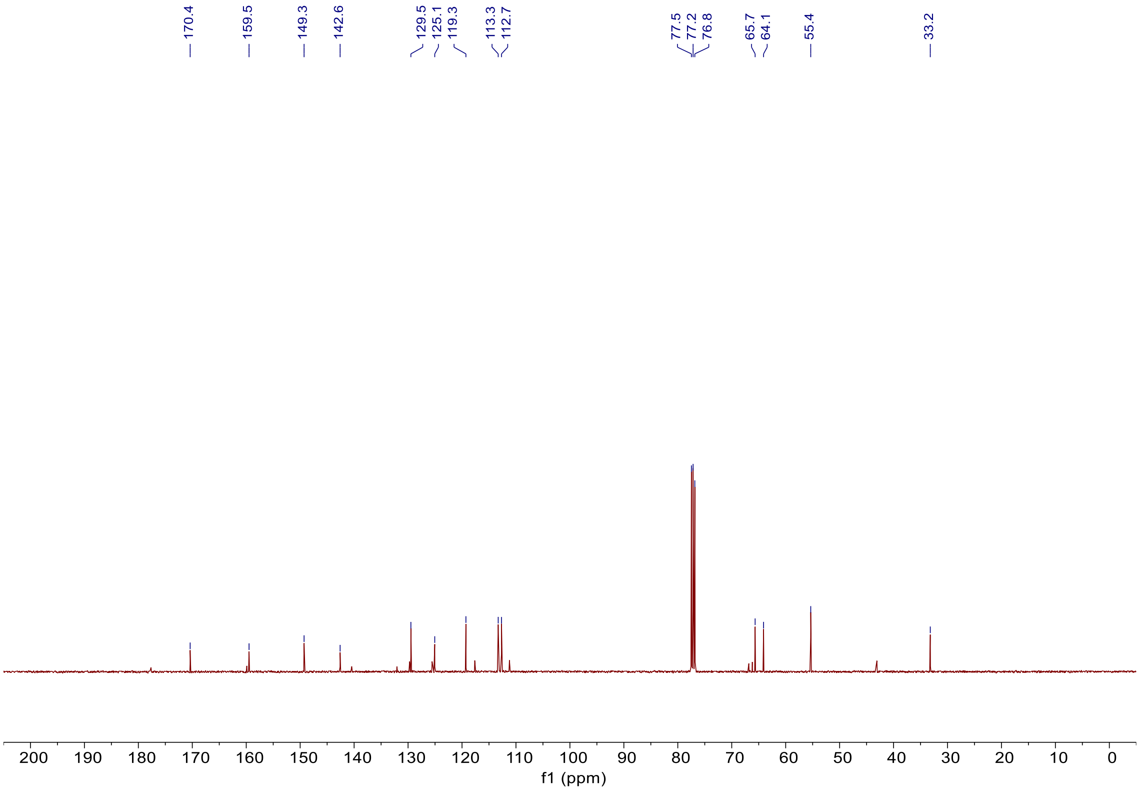

400 MHz ^{1}H NMR spectrum; 100.6 MHz ^{13}C NMR spectrum; $CDCl_3$ of **7k**

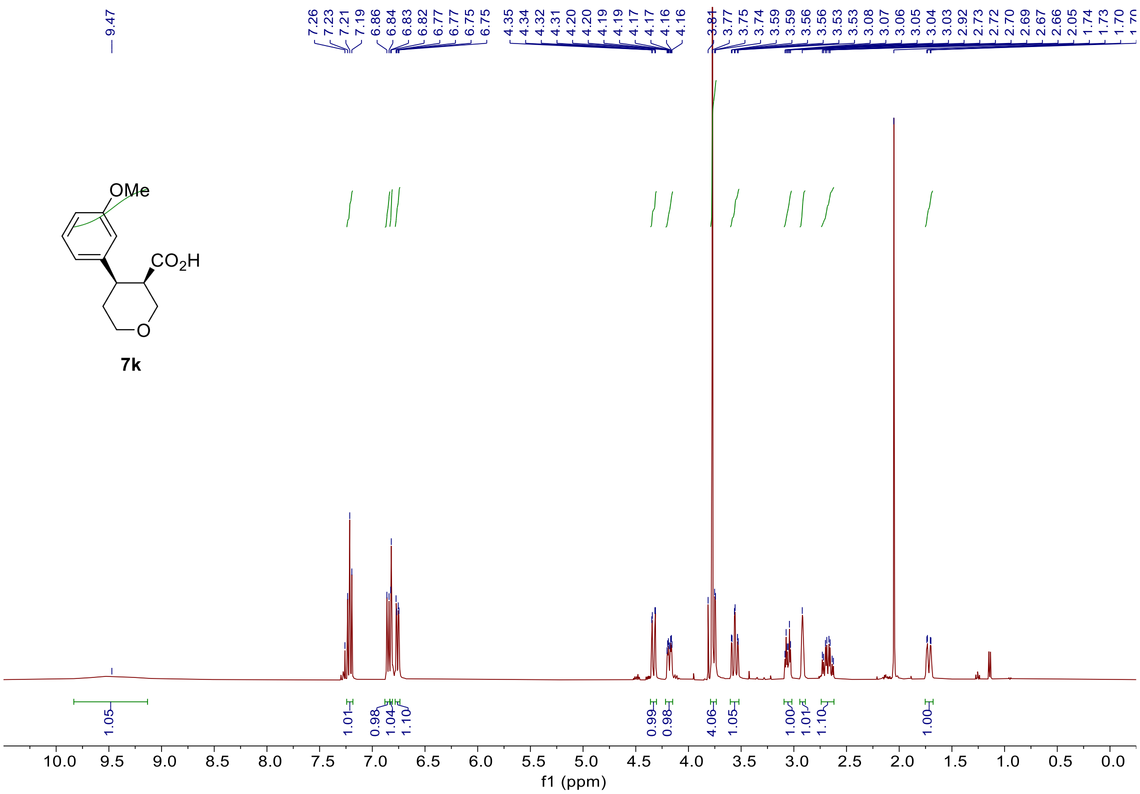

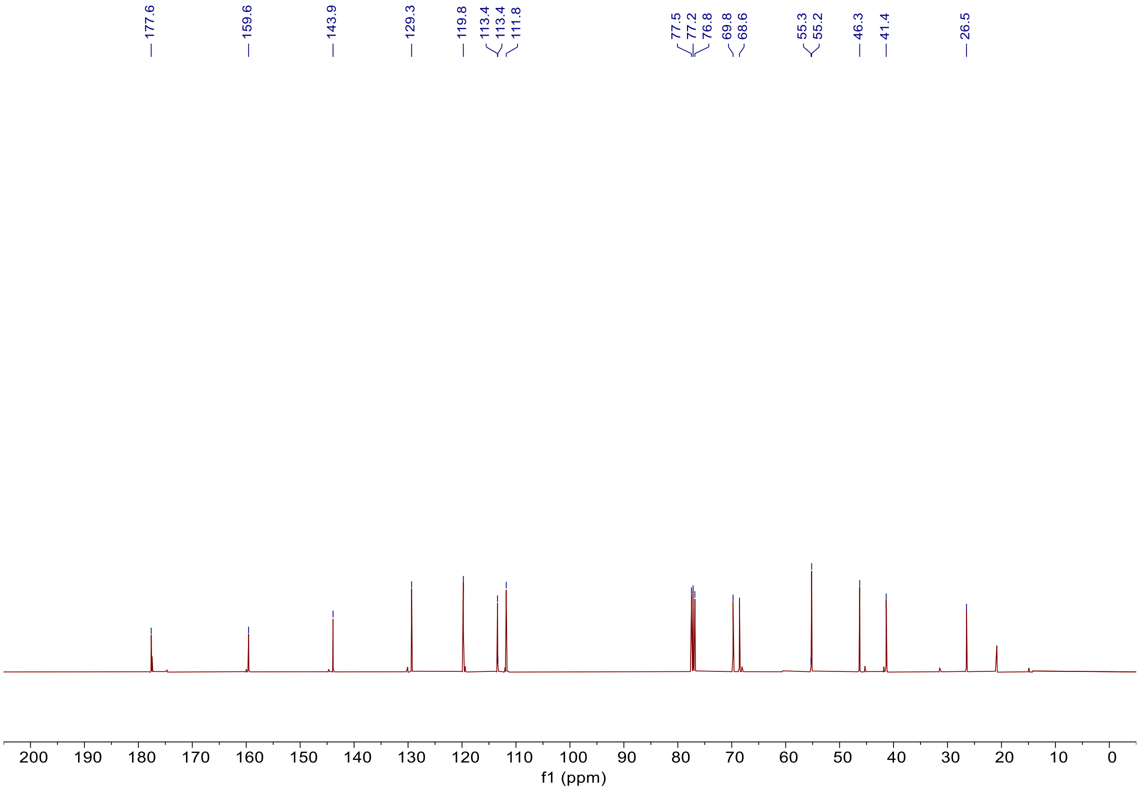

400 MHz ^{1}H NMR spectrum; 100.6 MHz ^{13}C NMR spectrum; $CDCl_3$ of **3a**

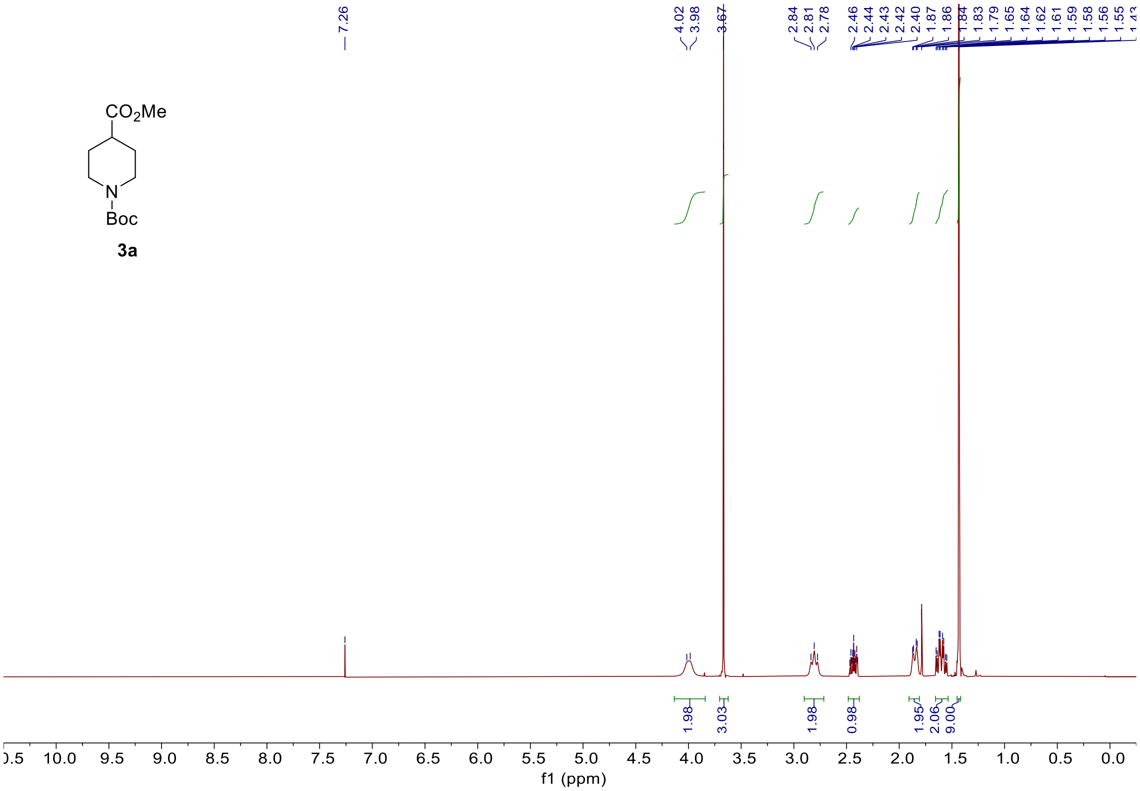

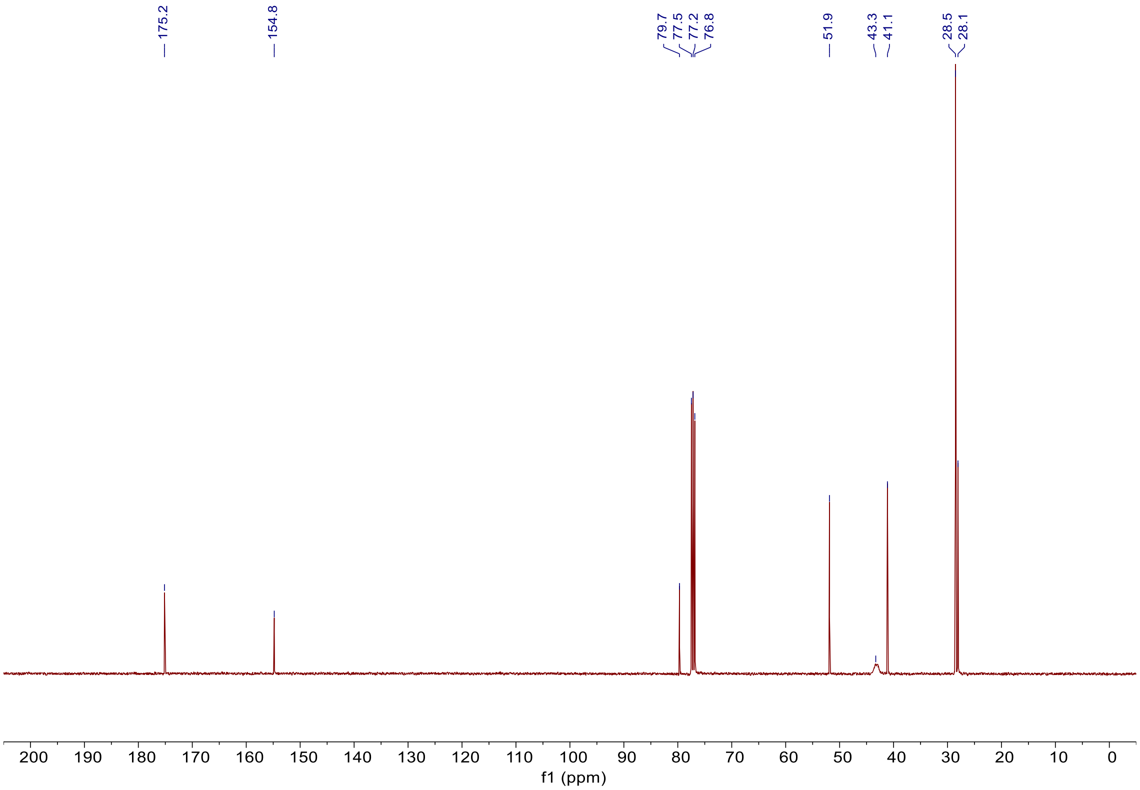

400 MHz ^{1}H NMR spectrum; 100.6 MHz ^{13}C NMR spectrum; $CDCl_3$ of **3b**

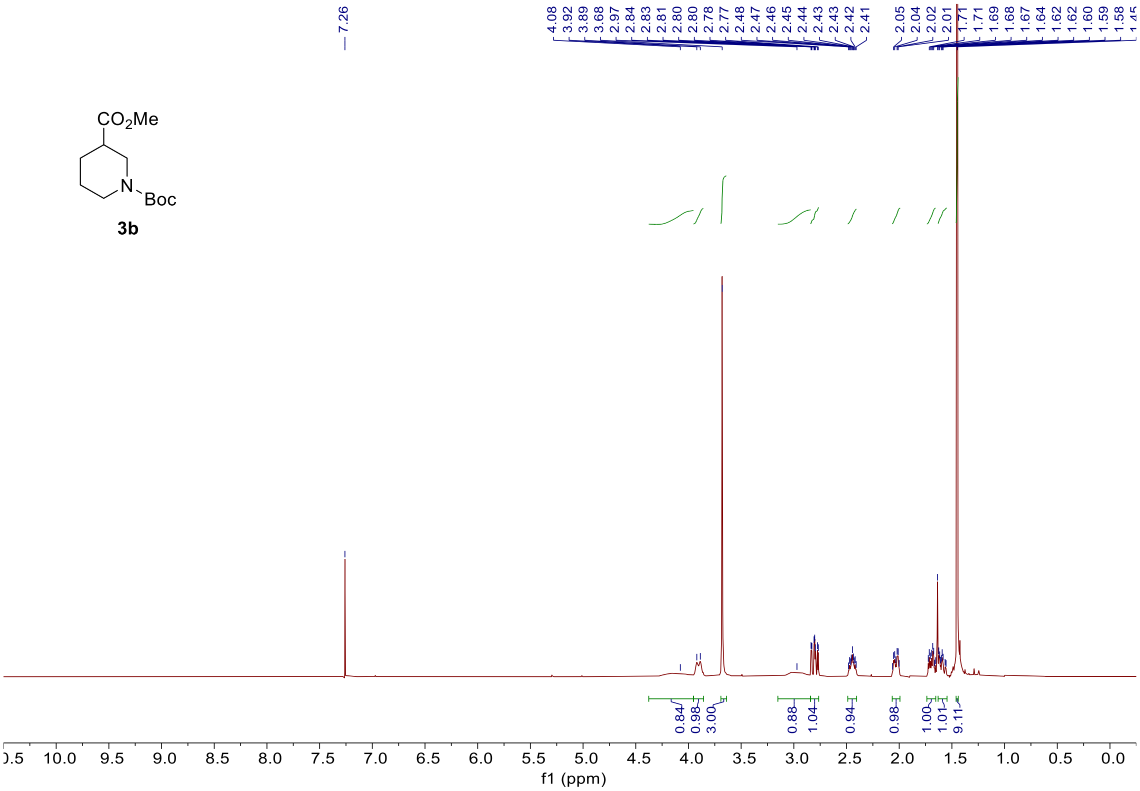

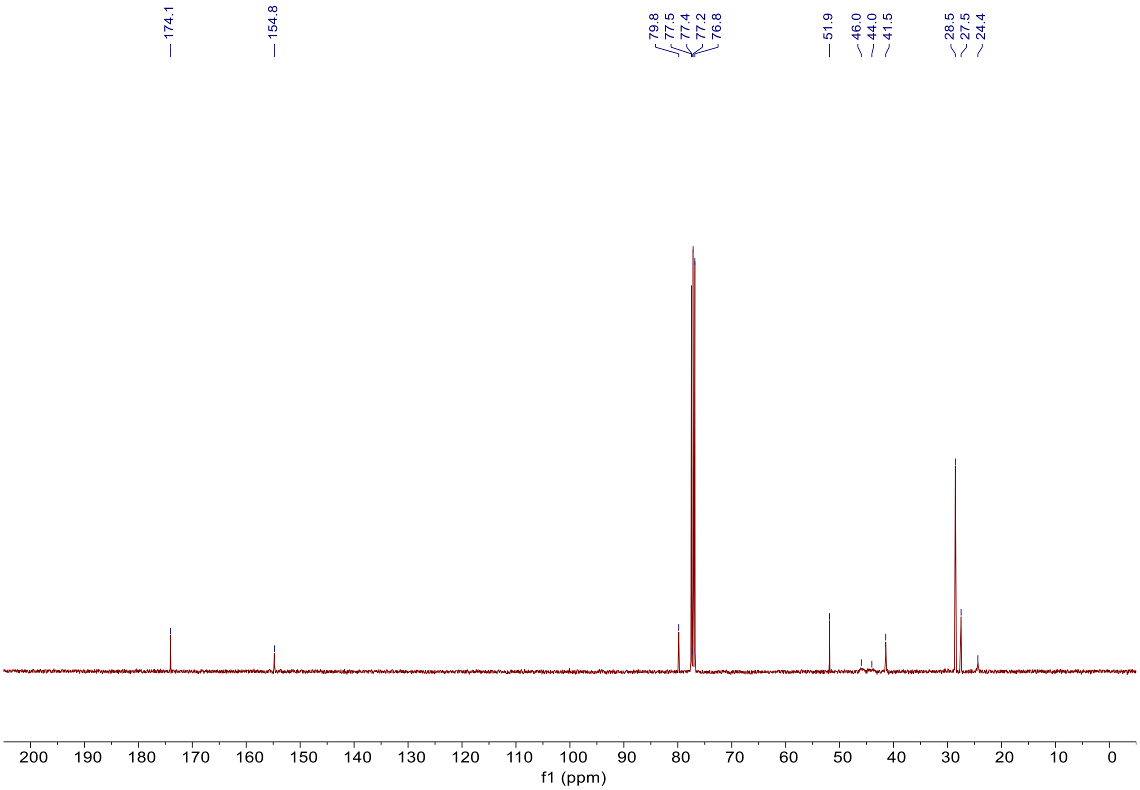

400 MHz ^{1}H NMR spectrum; 100.6 MHz ^{13}C NMR spectrum; $CDCl_3$ of **3c**

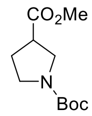

3c

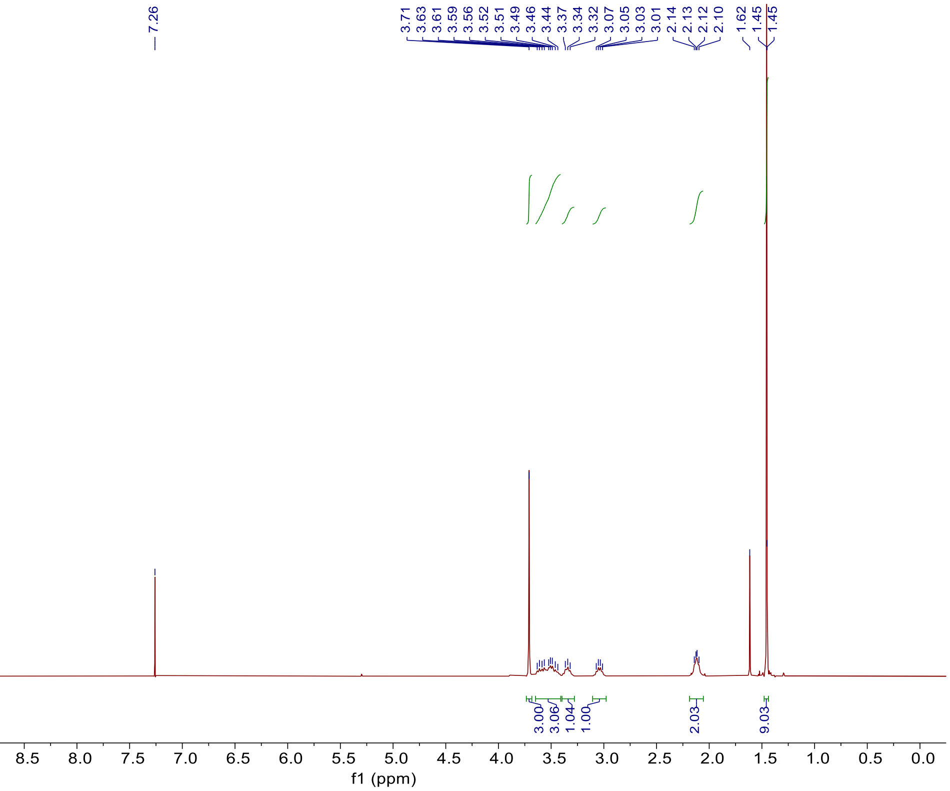

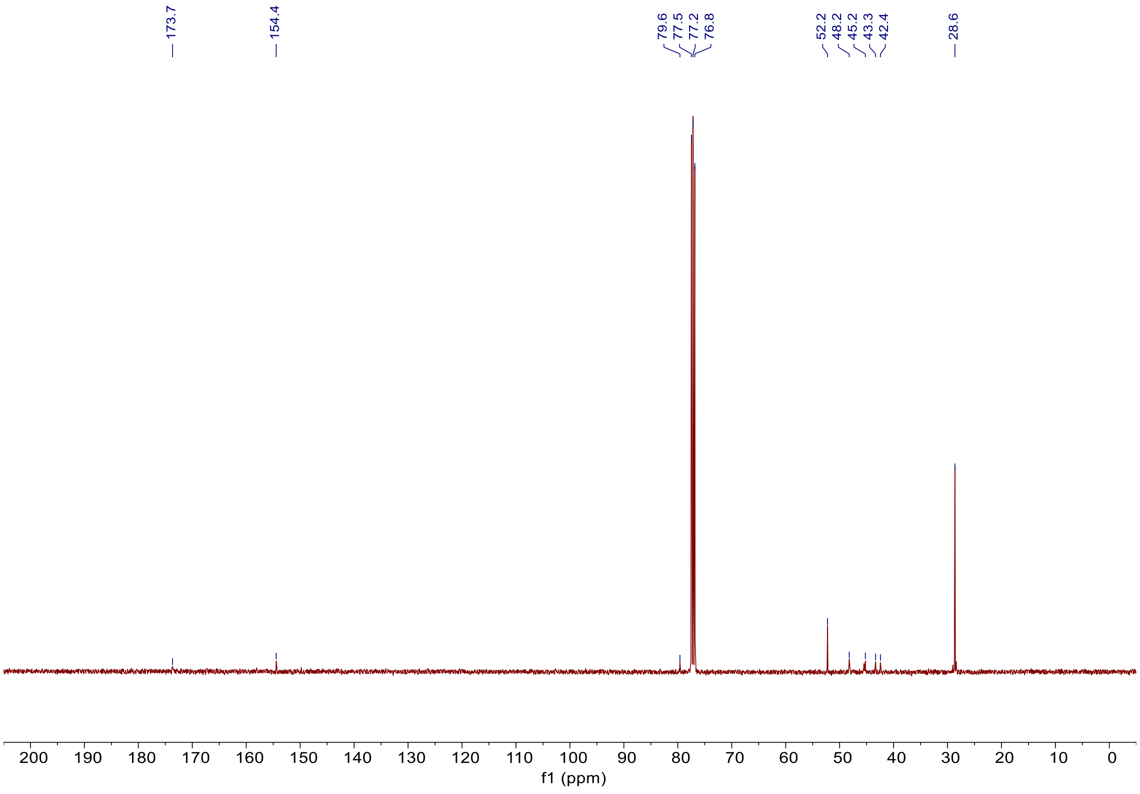

400 MHz ^{1}H NMR spectrum; 100.6 MHz ^{13}C NMR spectrum; $CDCl_3$ of **3d**

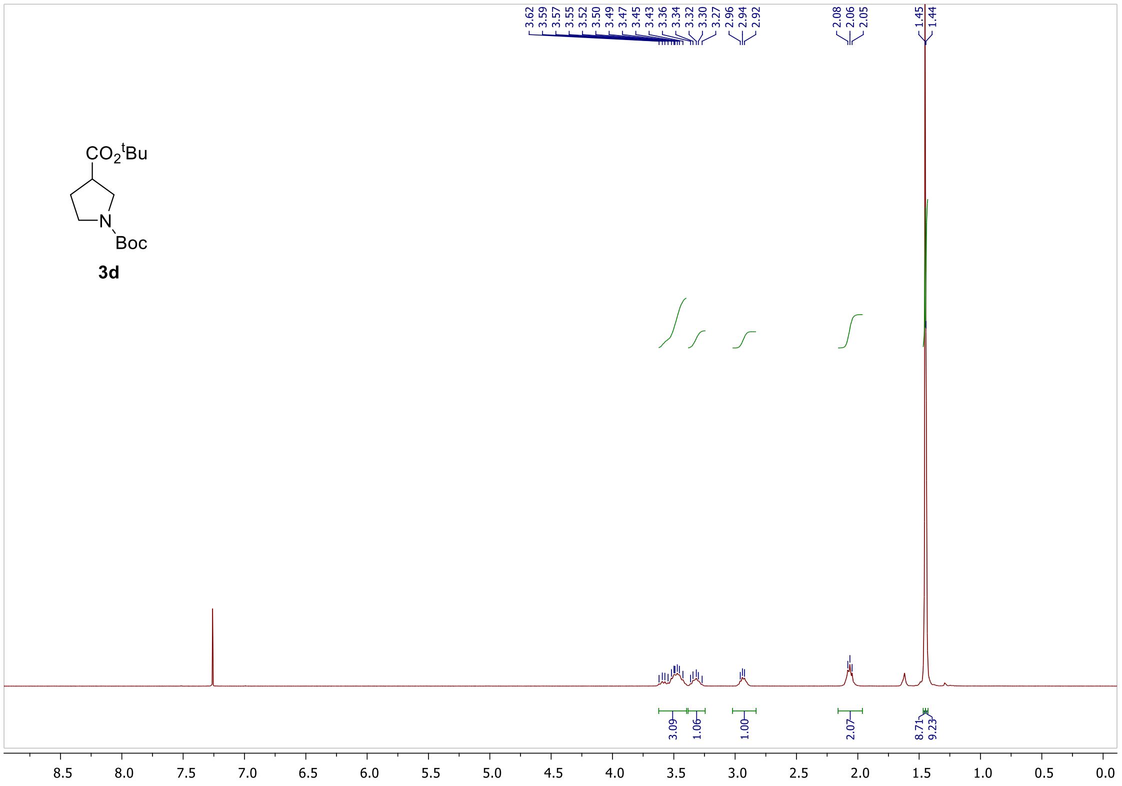

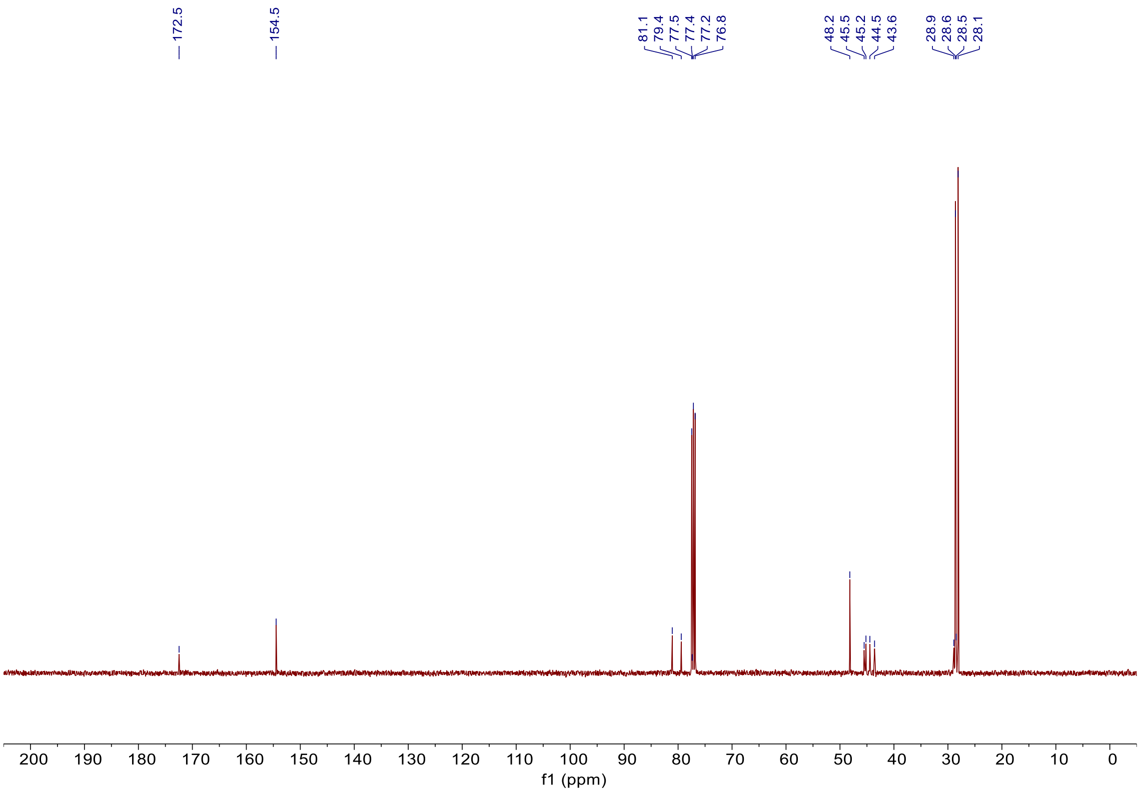

400 MHz ^{1}H NMR spectrum; 100.6 MHz ^{13}C NMR spectrum; $CDCl_3$ of **3j**

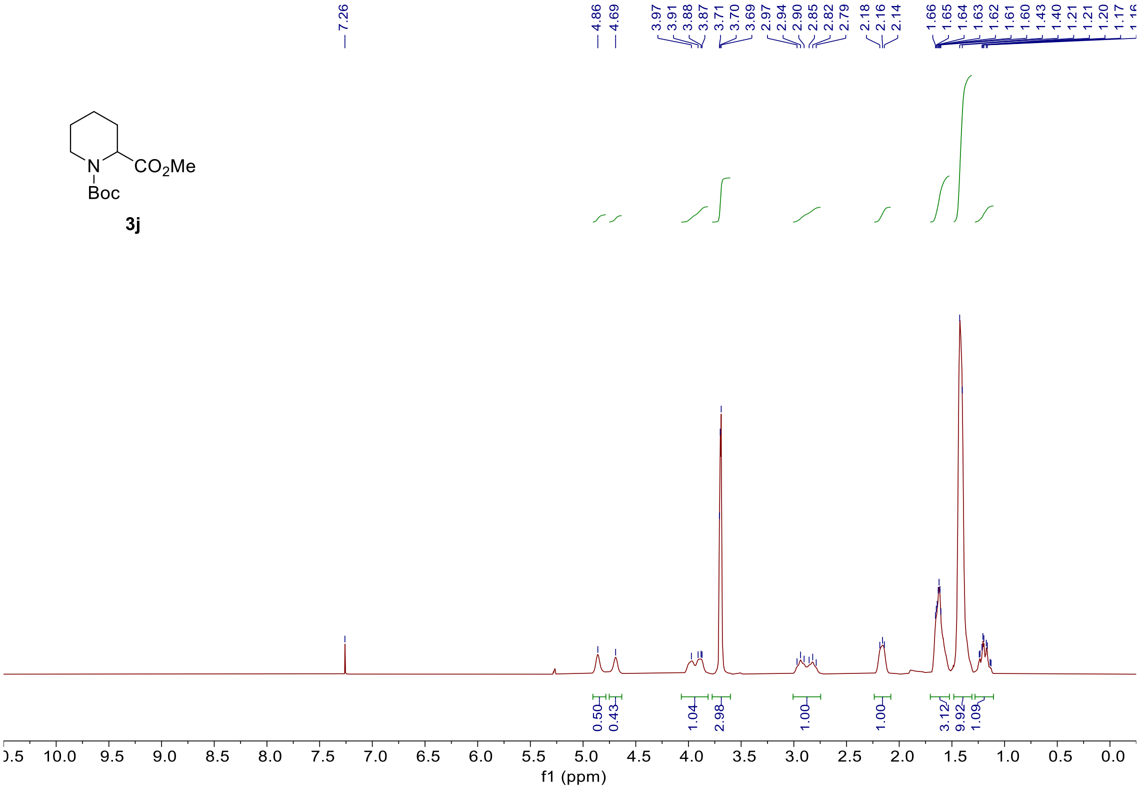

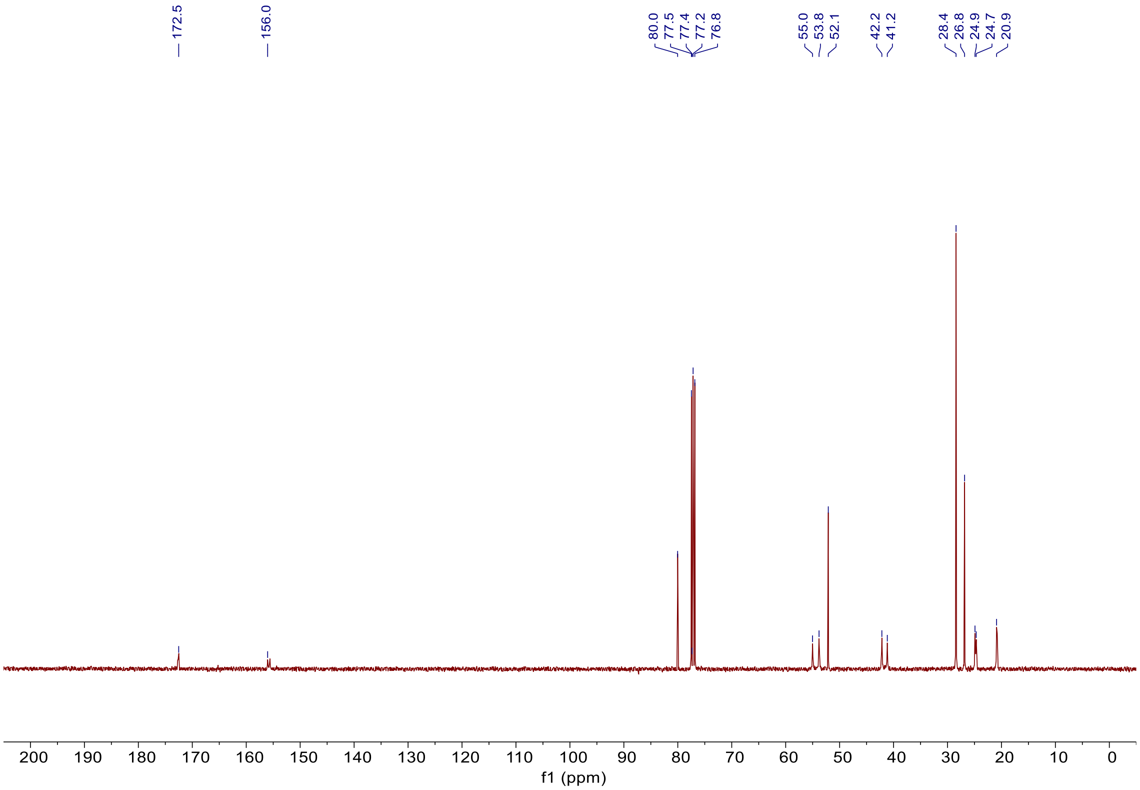

400 MHz ^{1}H NMR spectrum; 100.6 MHz ^{13}C NMR spectrum; $CDCl_3$ of **3k**

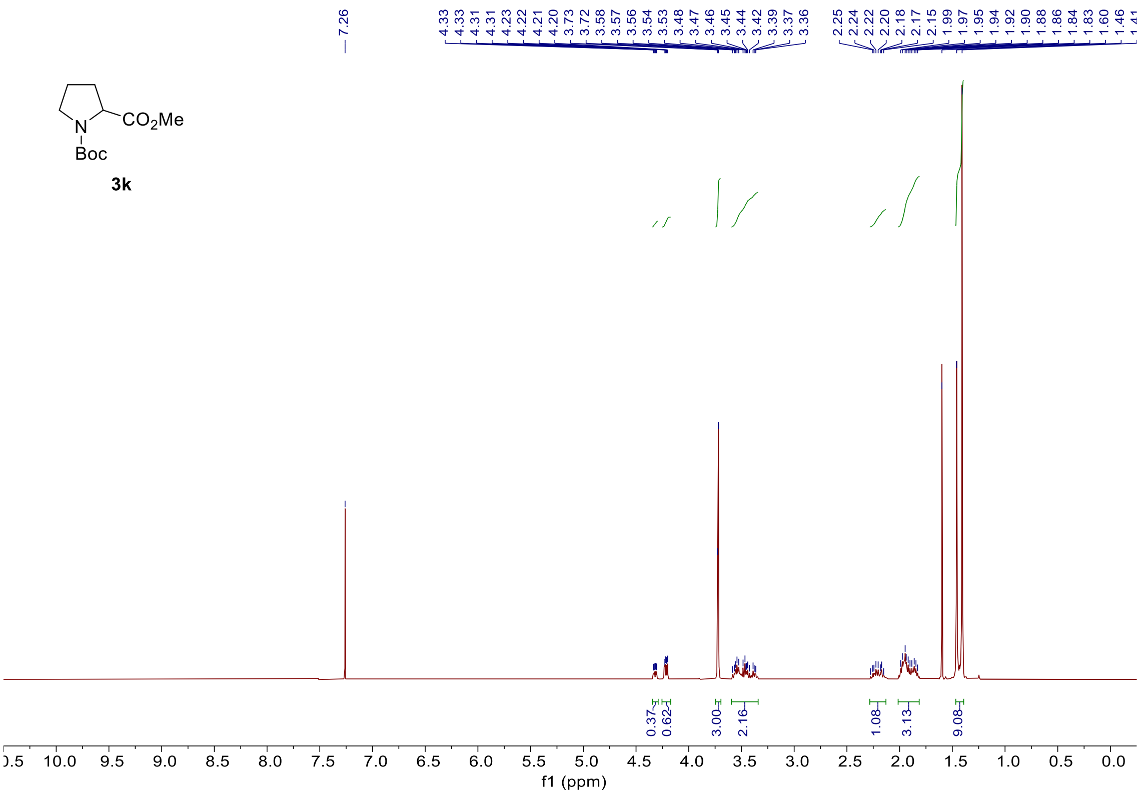

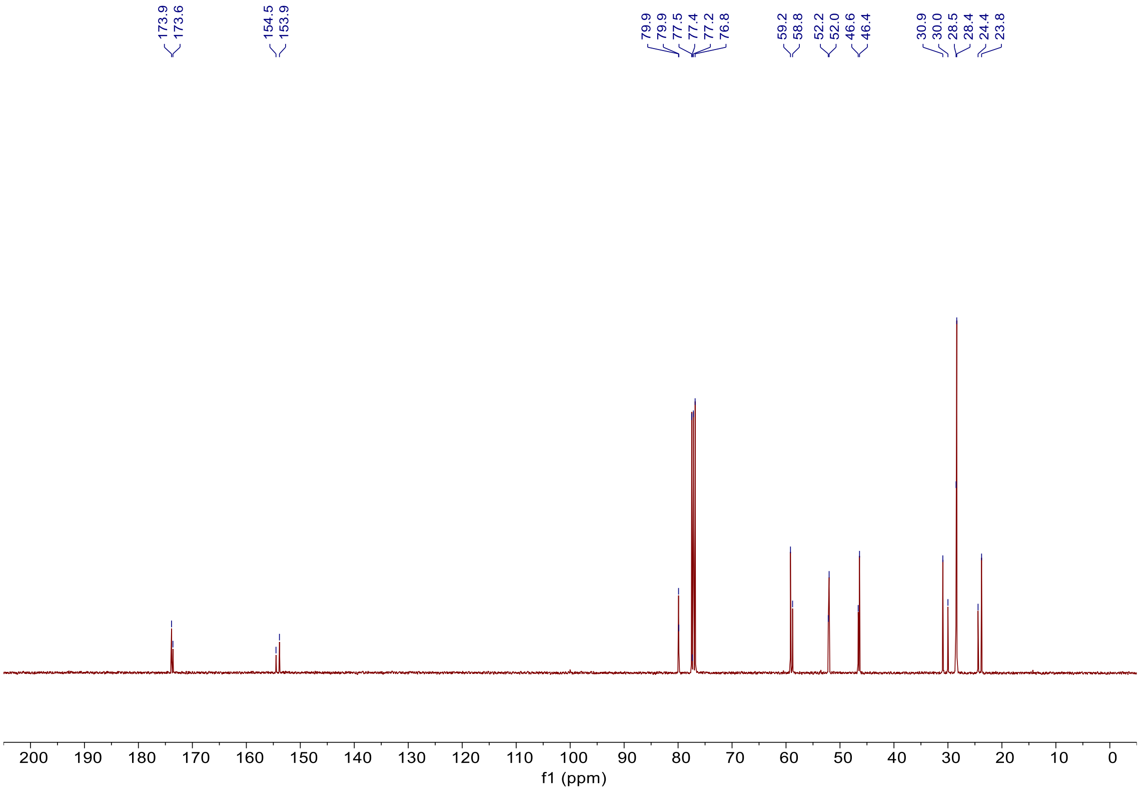

400 MHz ^{1}H NMR spectrum; 100.6 MHz ^{13}C NMR spectrum; $CDCl_3$ of **4a**

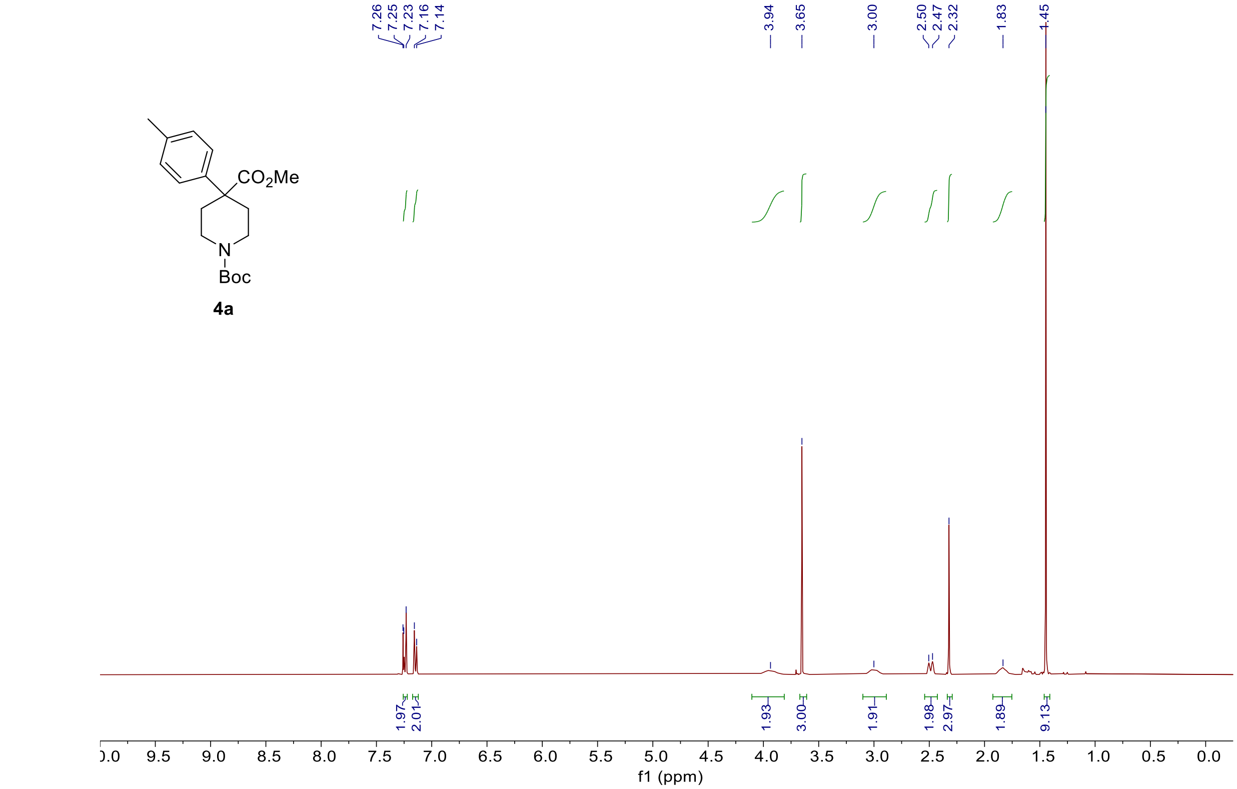

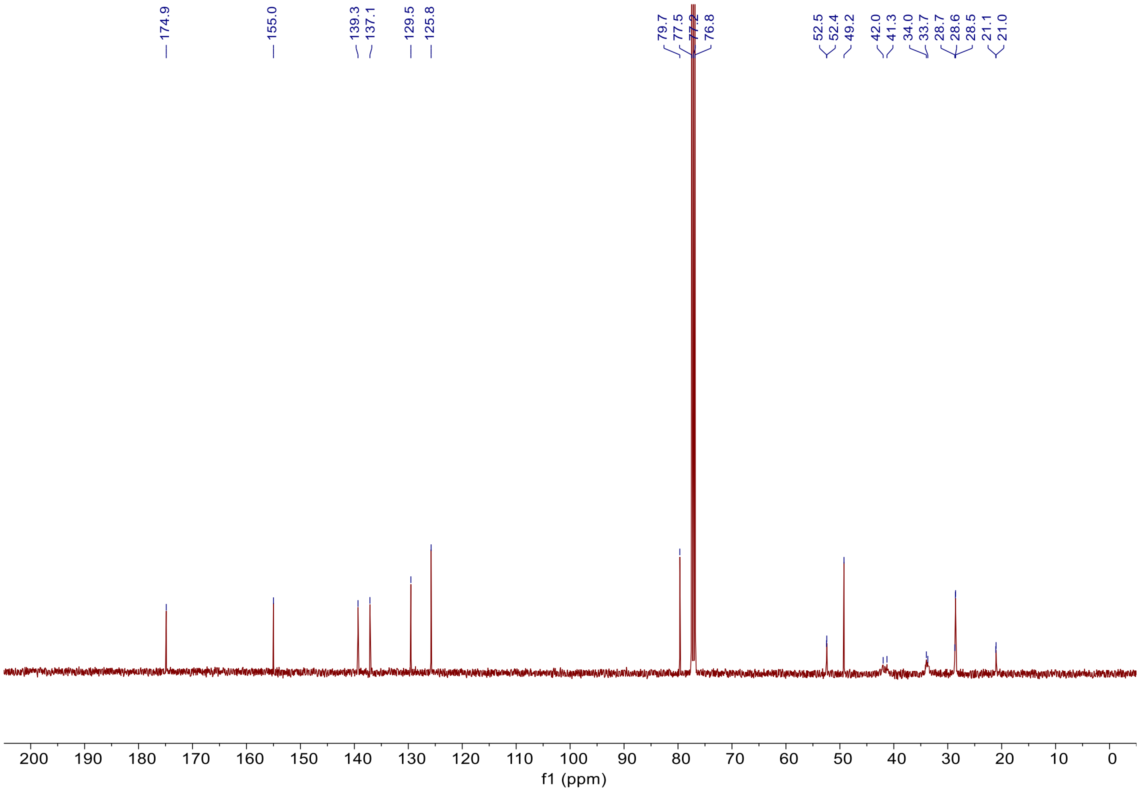

400 MHz ^{1}H NMR spectrum; 100.6 MHz ^{13}C NMR spectrum; $CDCl_3$ of **4b**

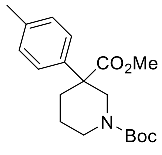

4b

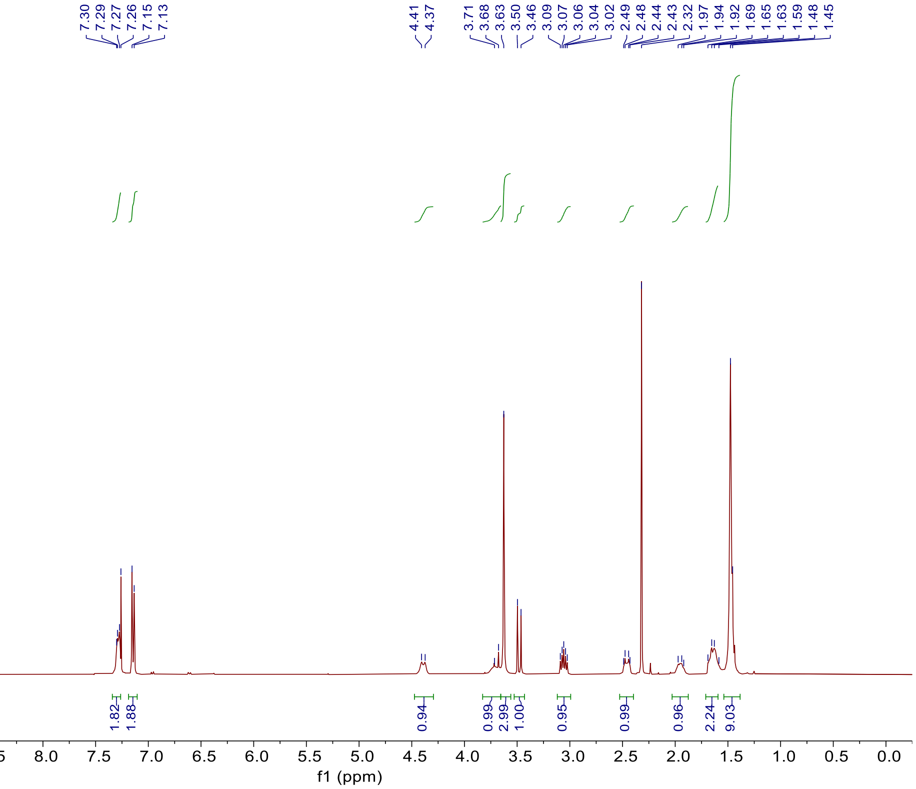

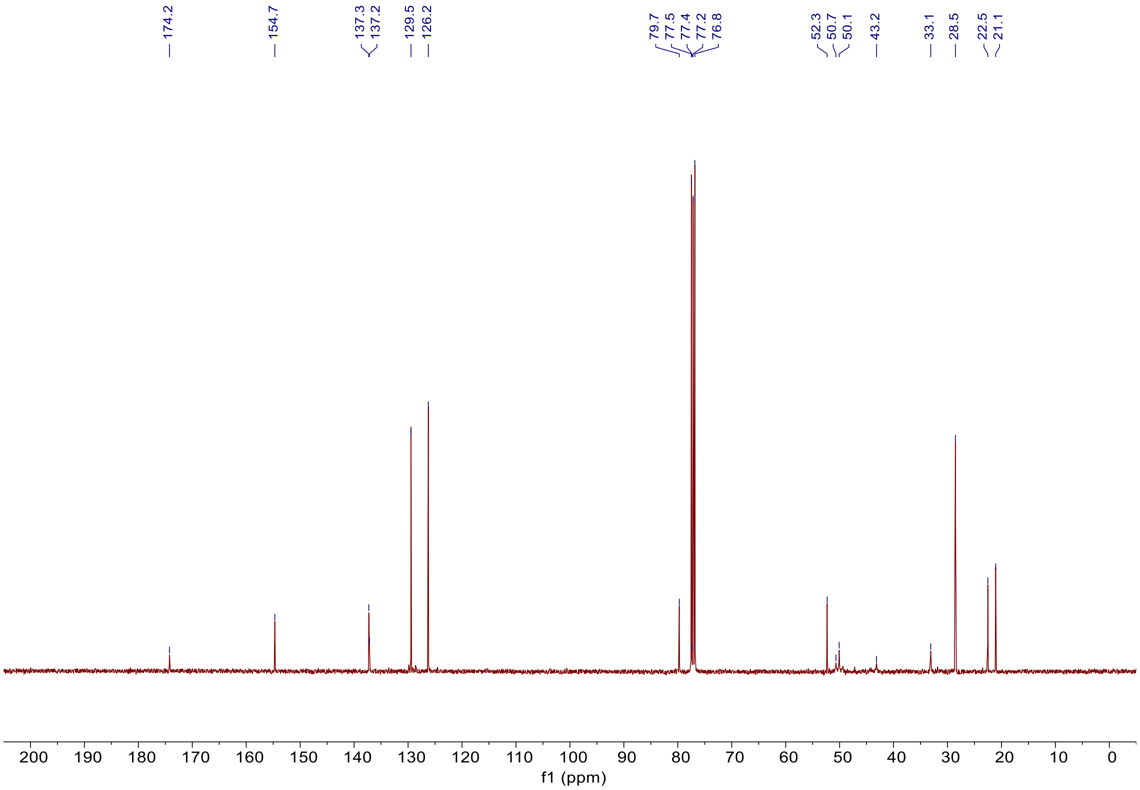

400 MHz ^{1}H NMR spectrum; 100.6 MHz ^{13}C NMR spectrum; $CDCl_3$ of **4c**

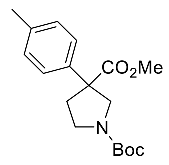

4c

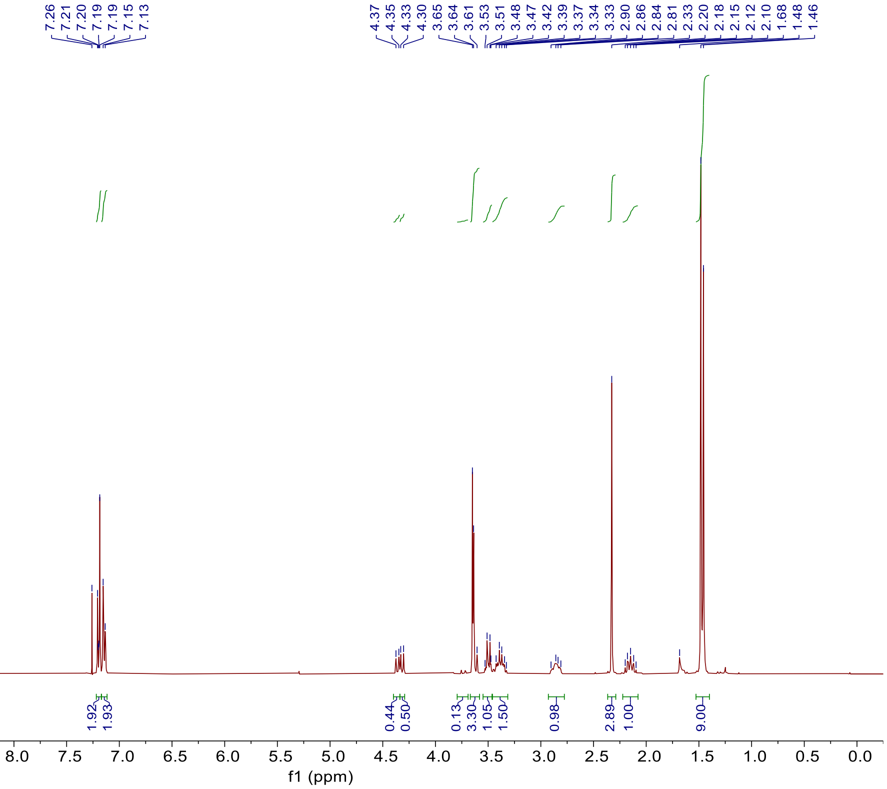

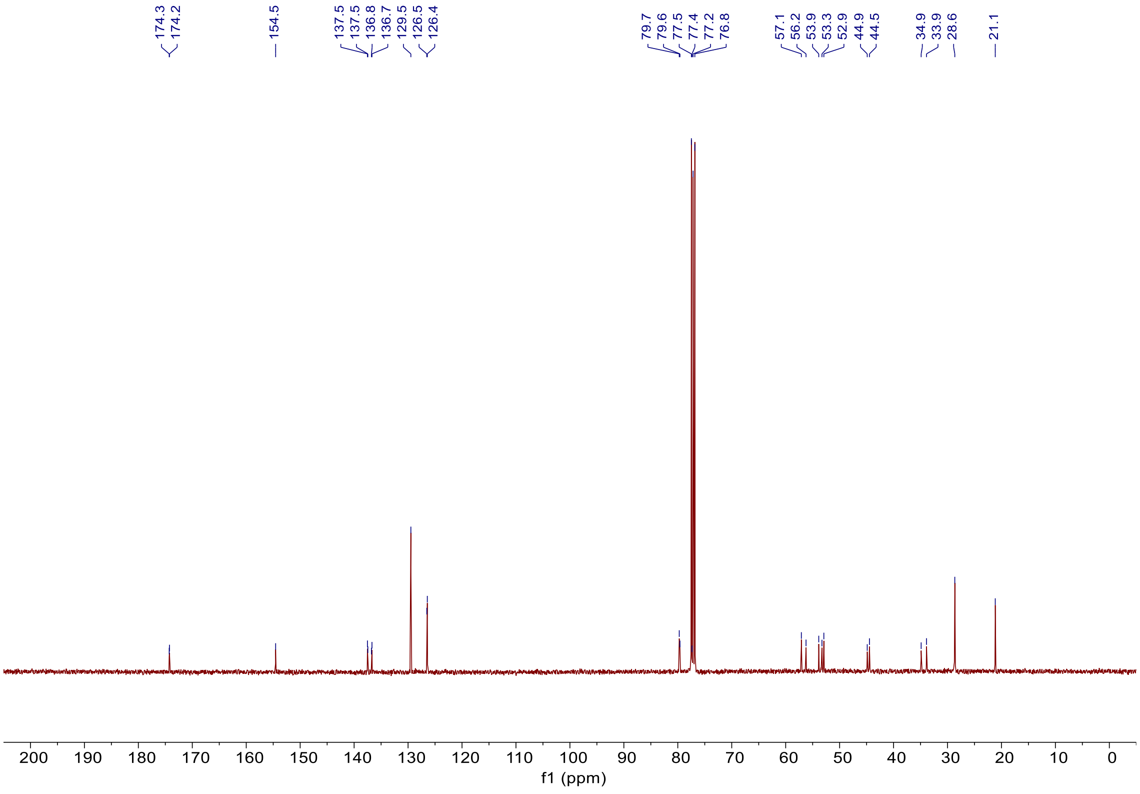

400 MHz ^{1}H NMR spectrum; 100.6 MHz ^{13}C NMR spectrum; $CDCl_3$ of **4d**

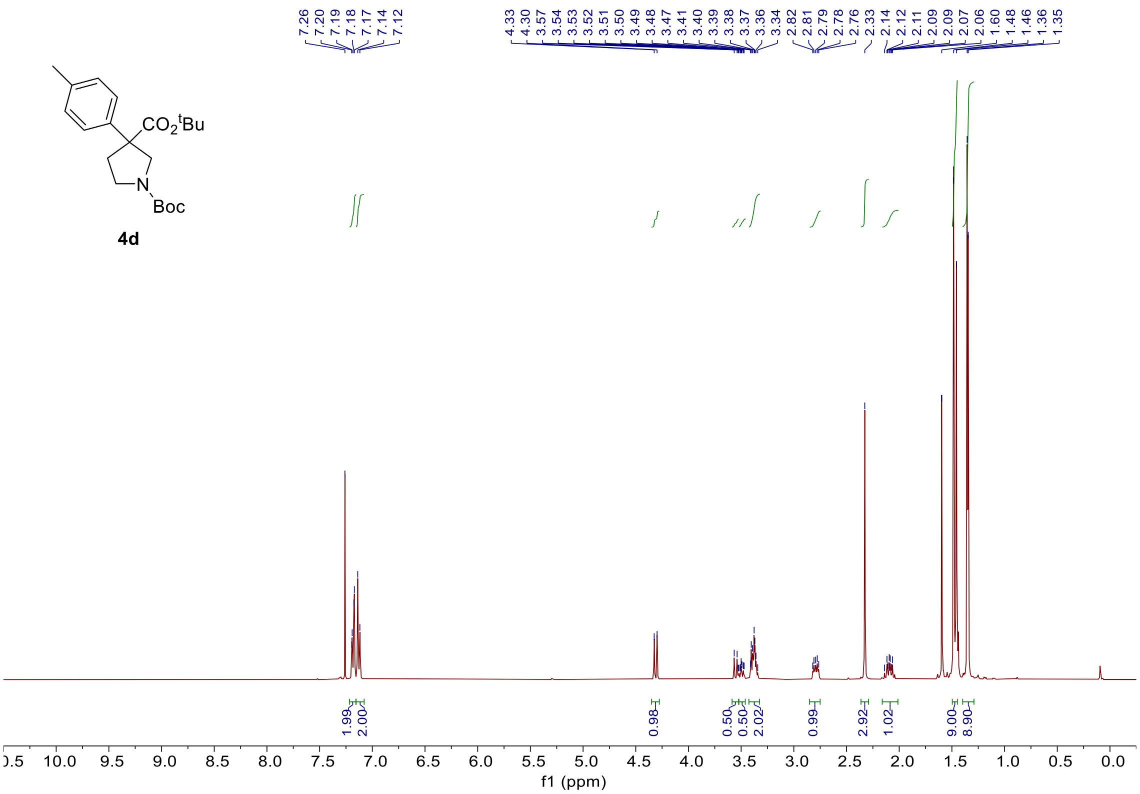

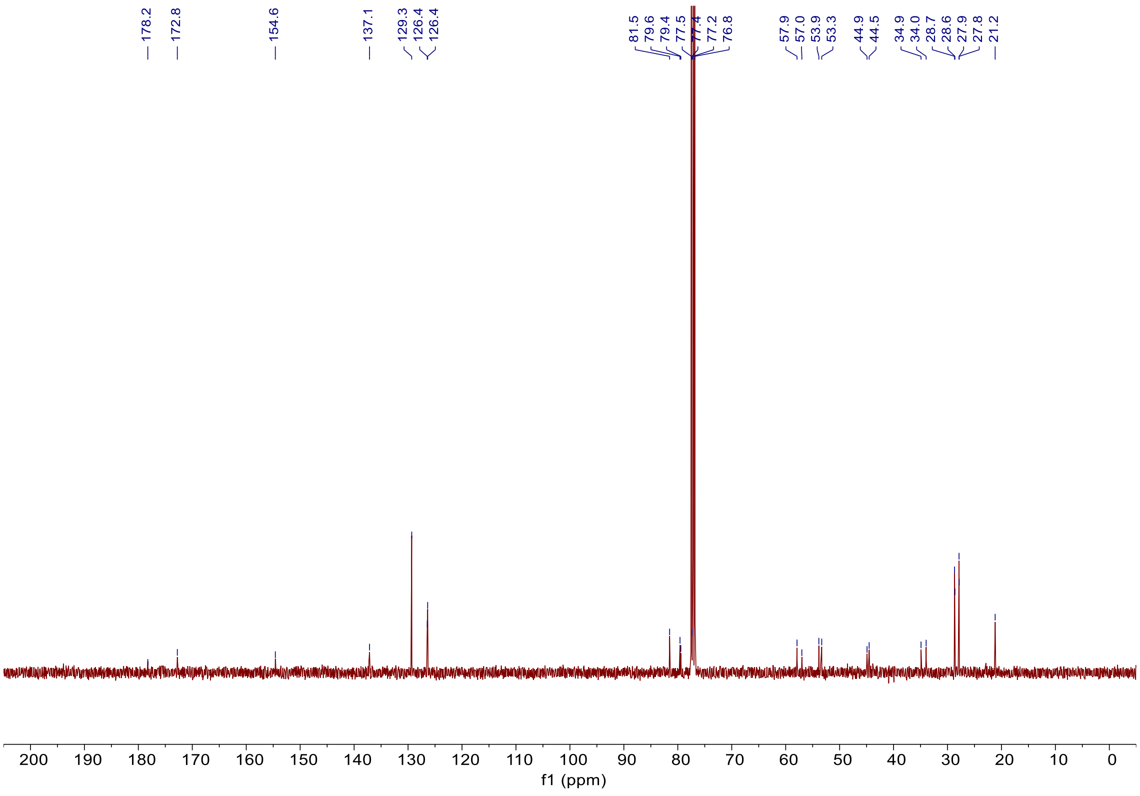

400 MHz ^{1}H NMR spectrum; 100.6 MHz ^{13}C NMR spectrum; $CDCl_3$ of **4e**

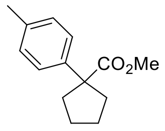

4e

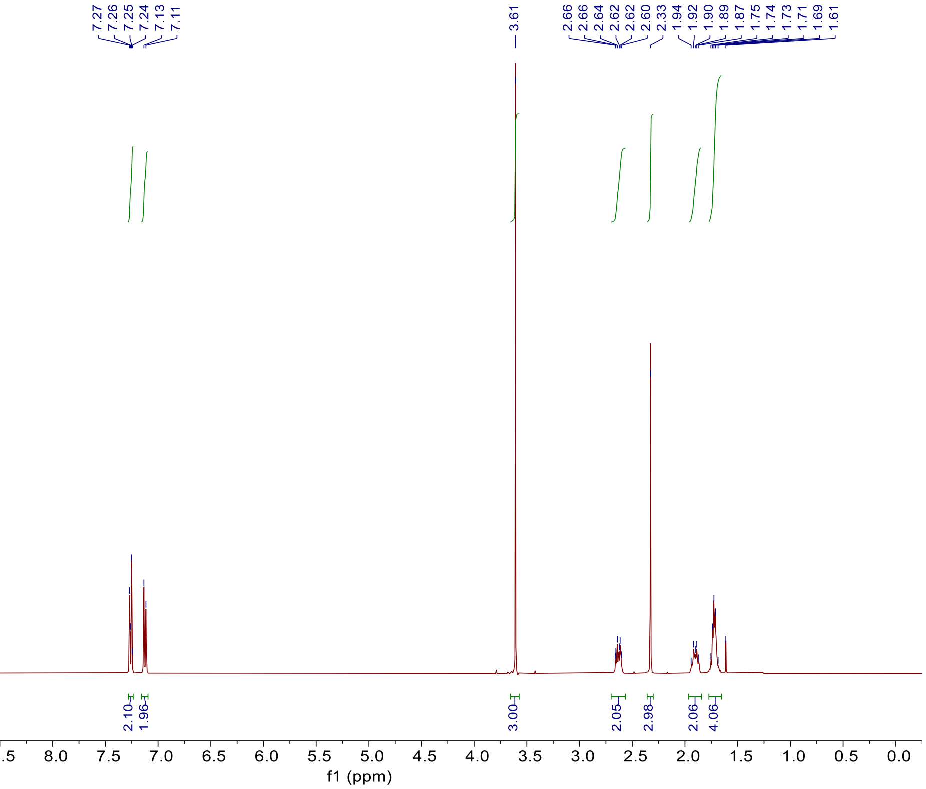

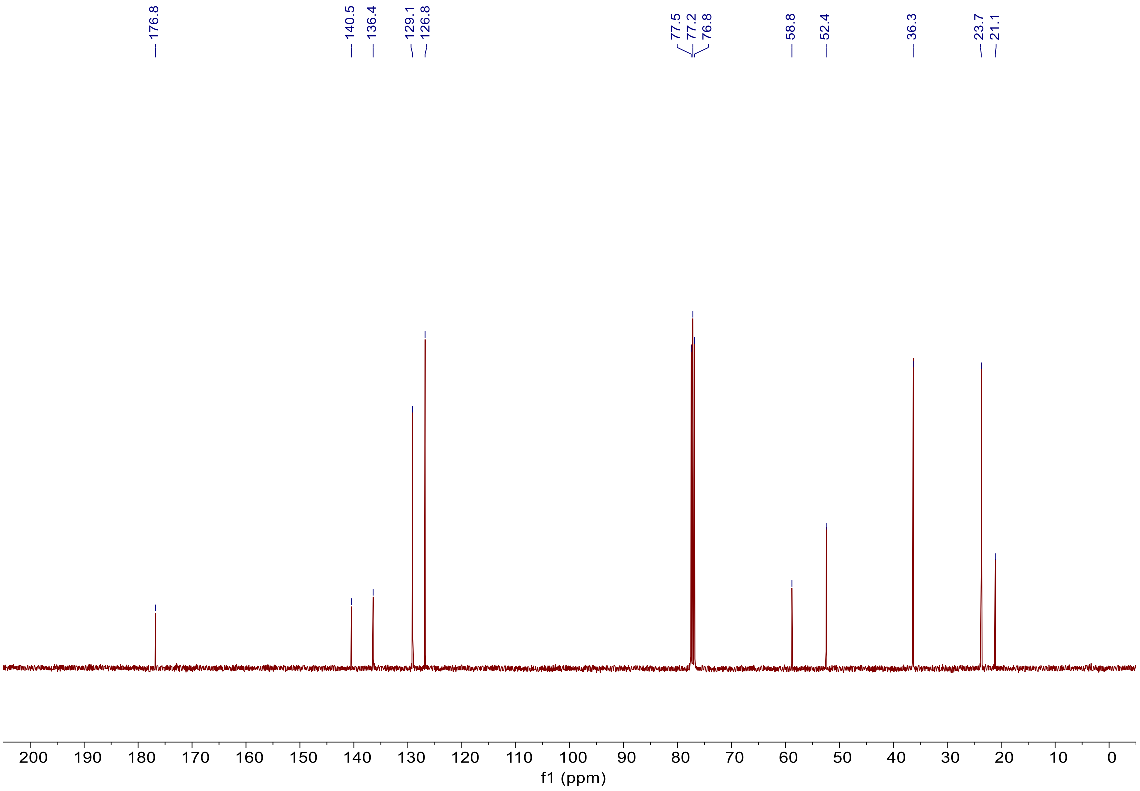

400 MHz ^{1}H NMR spectrum; 100.6 MHz ^{13}C NMR spectrum; $CDCl_3$ of **4f**

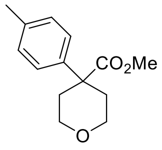

4f

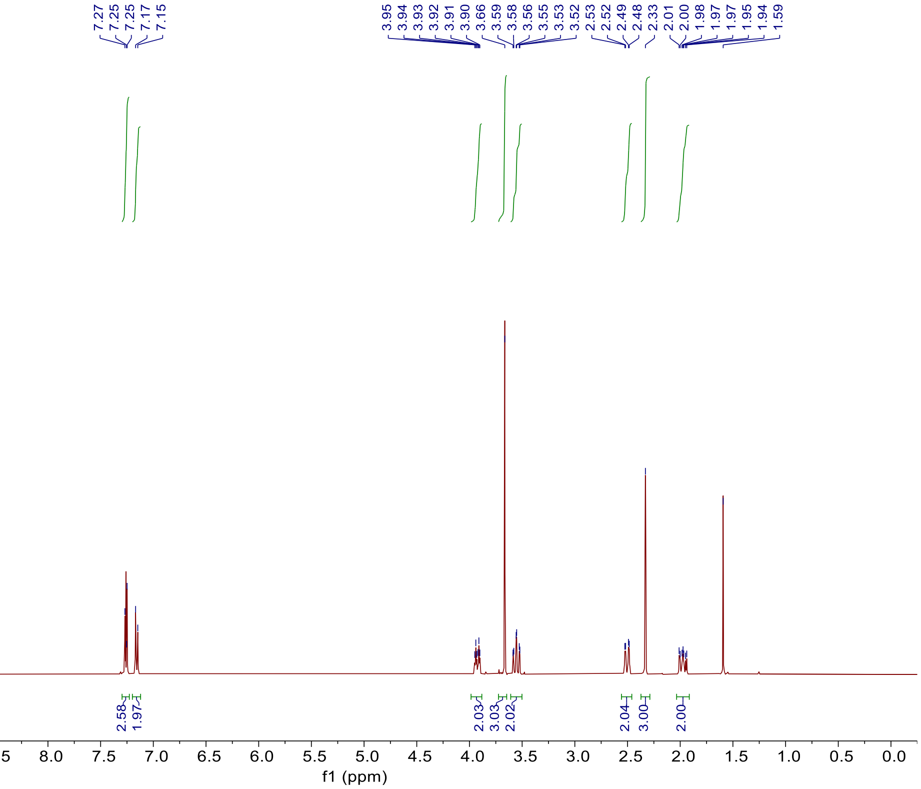

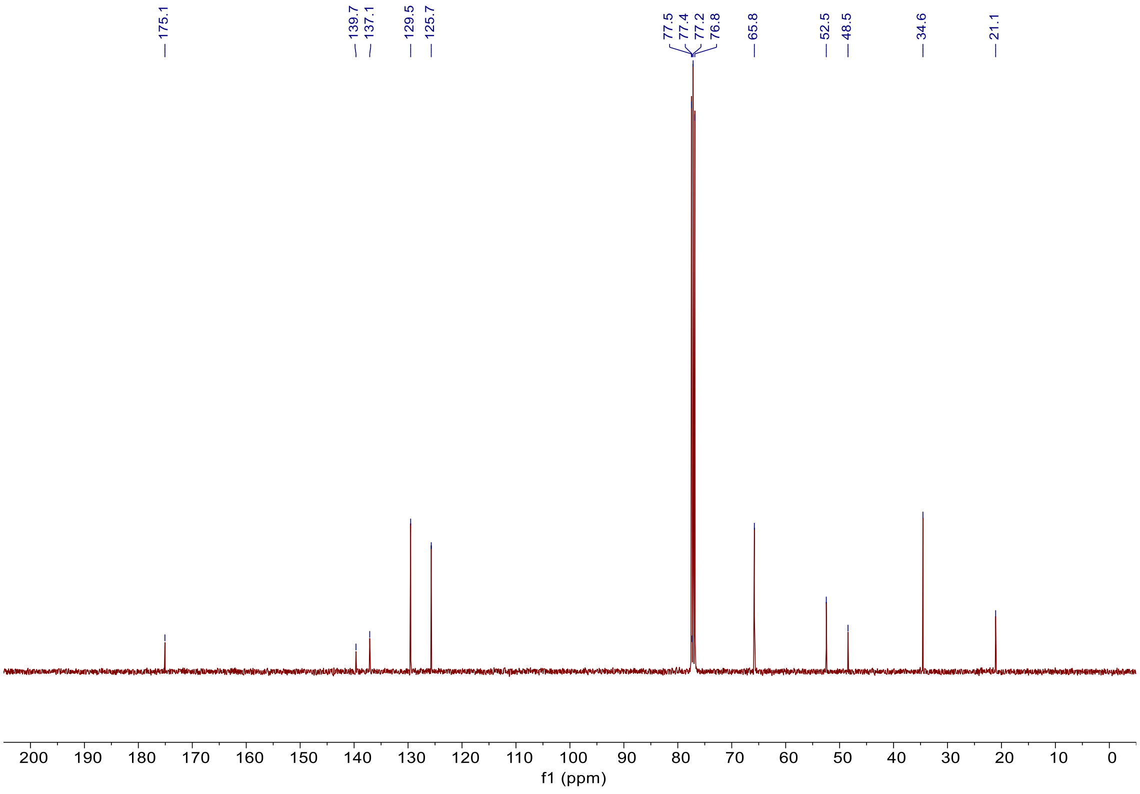

400 MHz ^{1}H NMR spectrum; 100.6 MHz ^{13}C NMR spectrum; $CDCl_3$ of **4l**

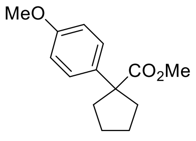

4l

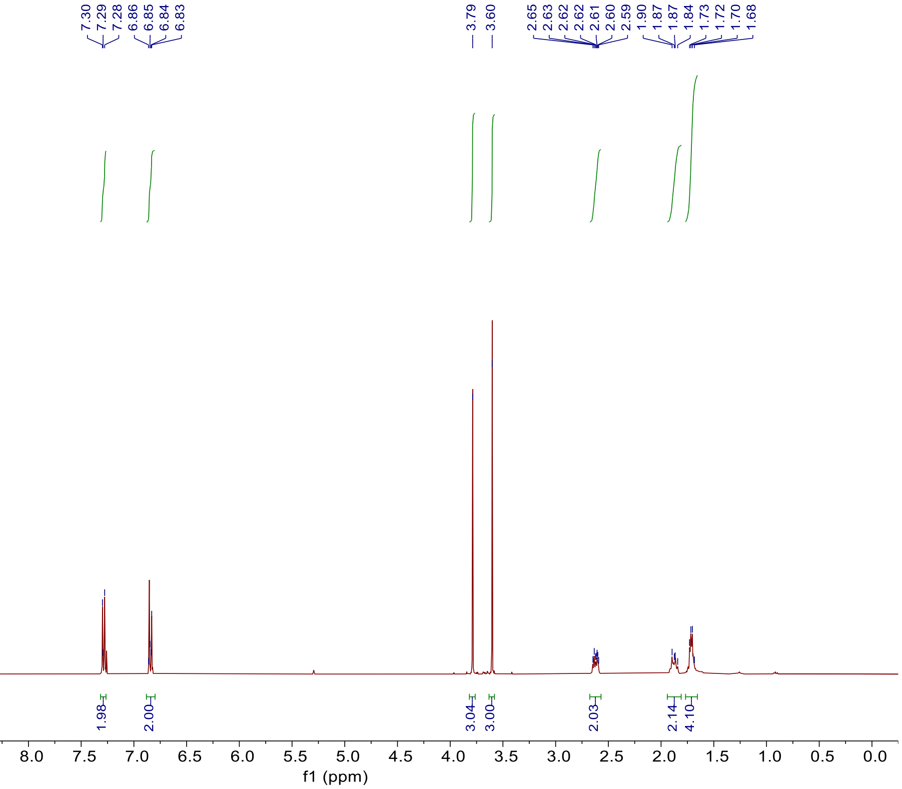

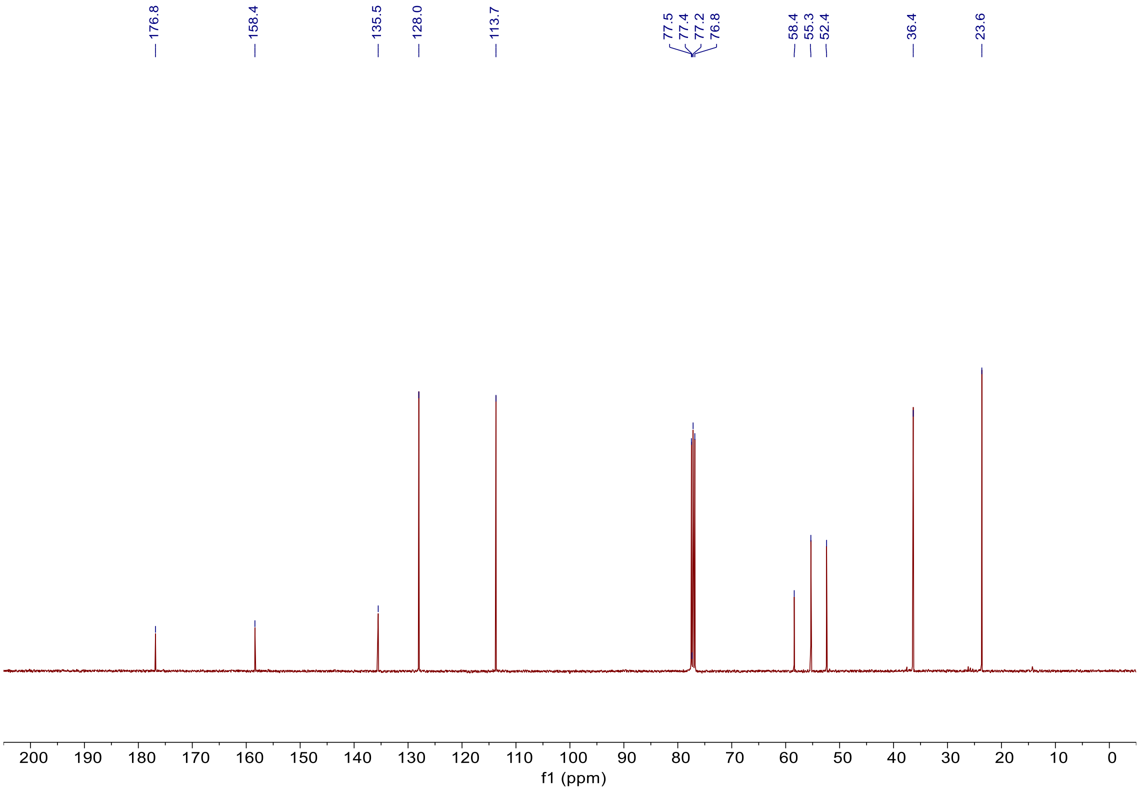

400 MHz ^{1}H NMR spectrum; 100.6 MHz ^{13}C NMR spectrum; $CDCl_3$ of **4m**

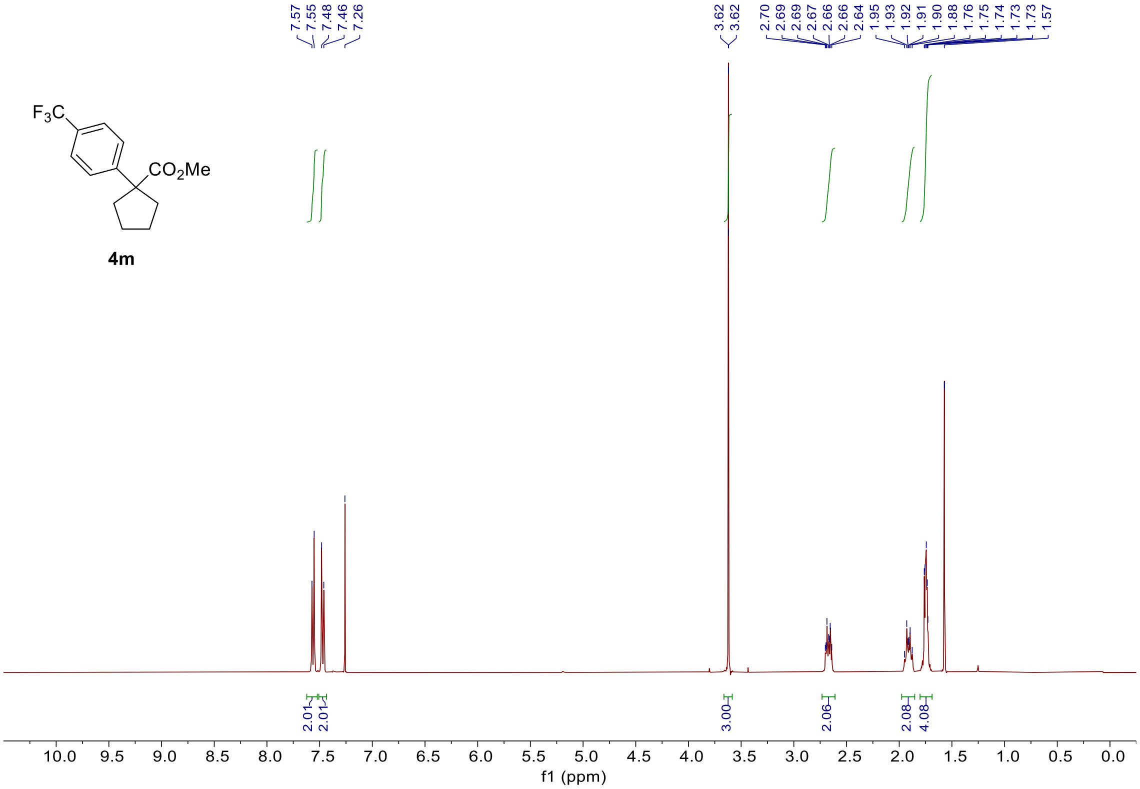

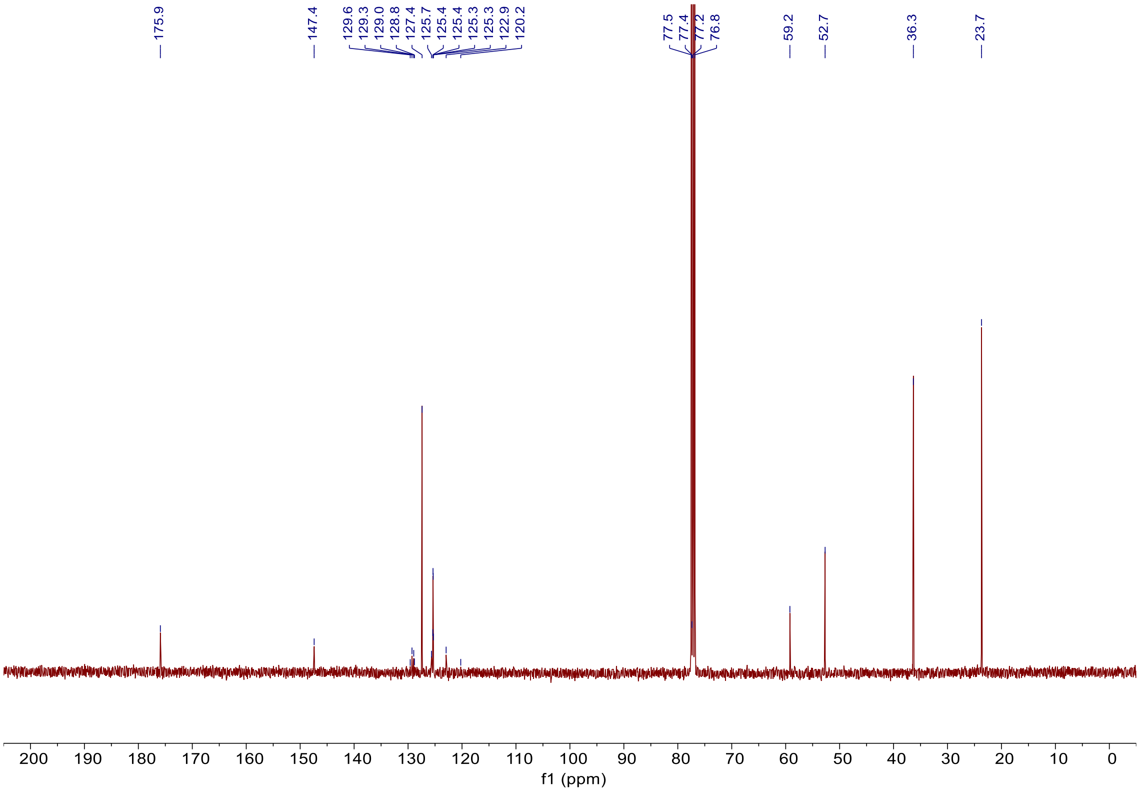

400 MHz ^1H NMR spectrum; 100.6 MHz ^{13}C NMR spectrum; $CDCl_3$ of **4n**

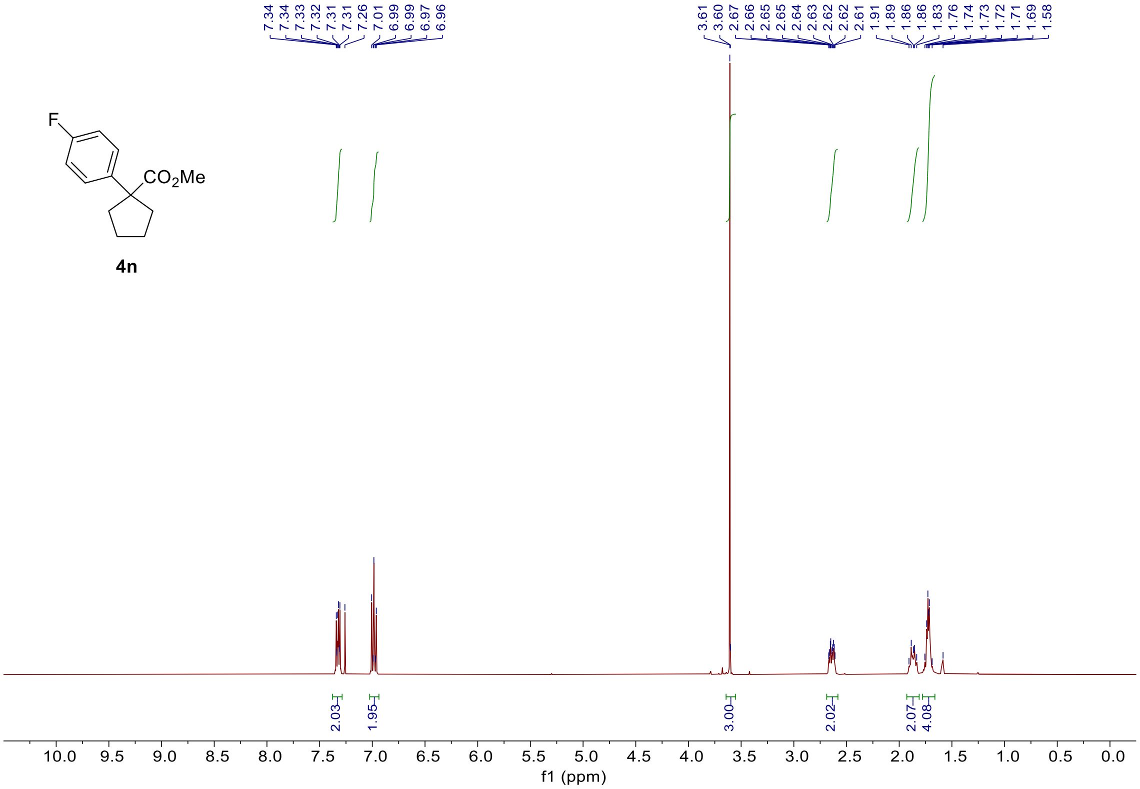

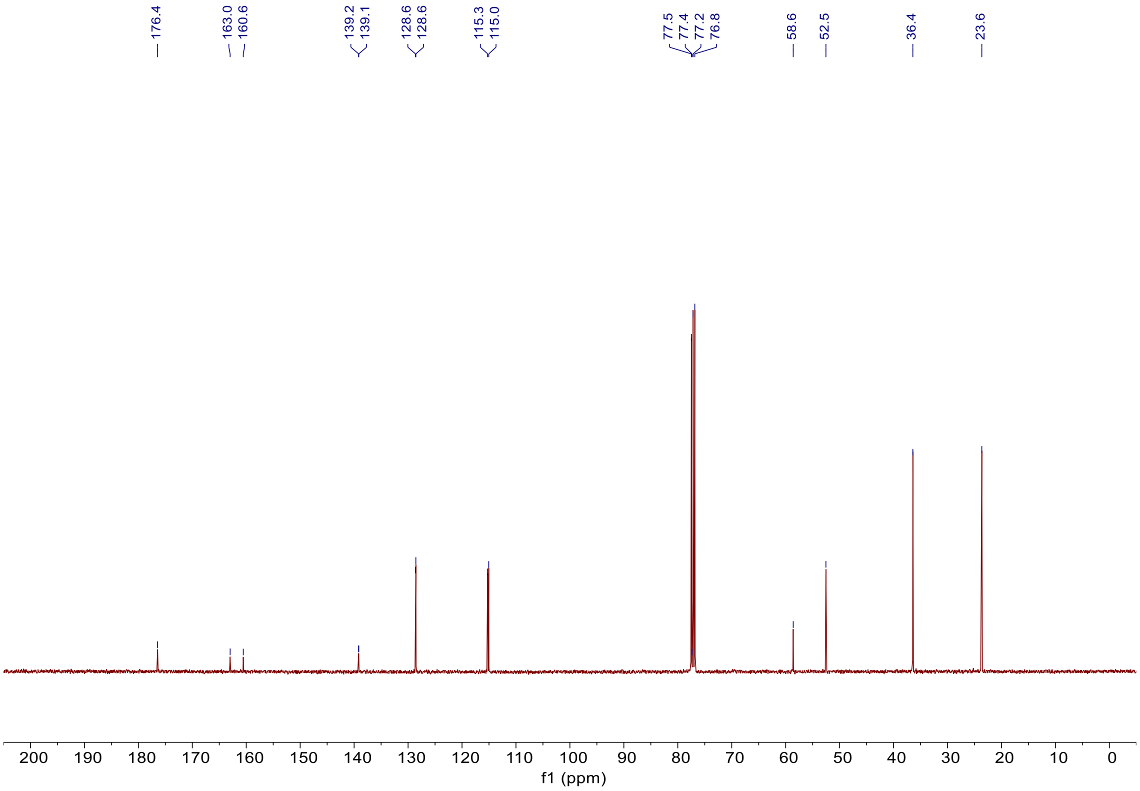

400 MHz ^{1}H NMR spectrum; 100.6 MHz ^{13}C NMR spectrum; $CDCl_3$ of **4o**

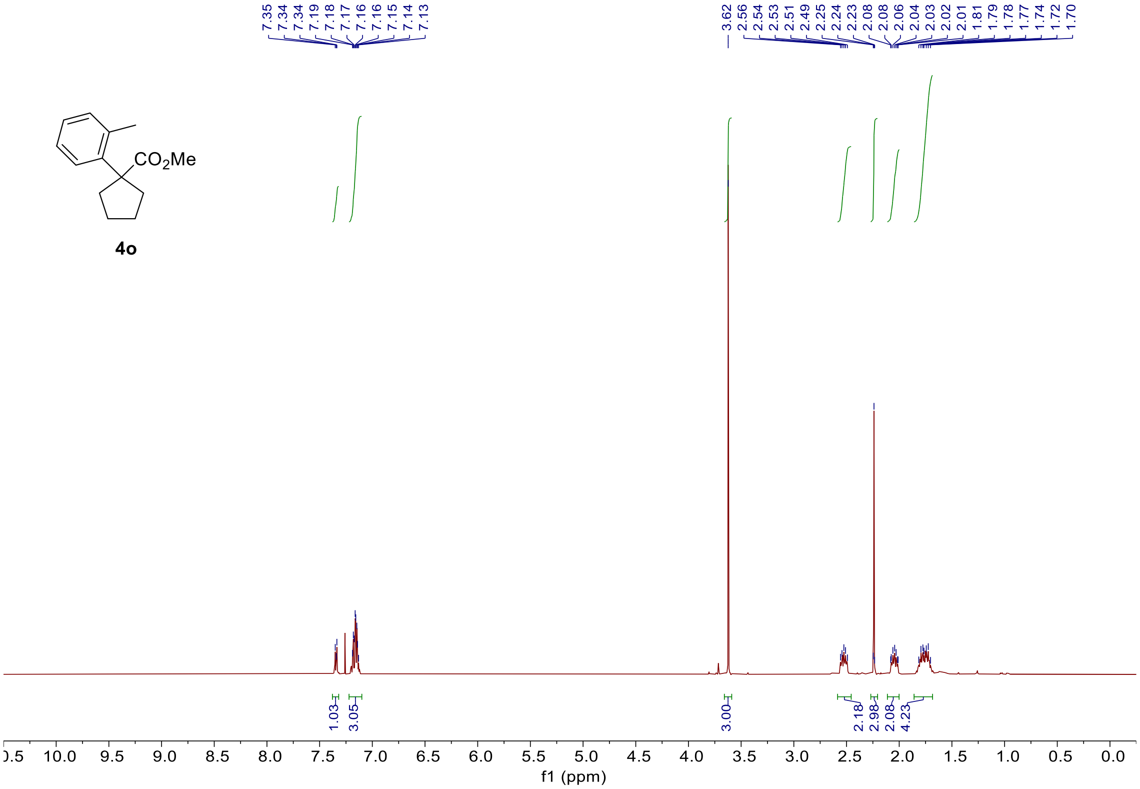

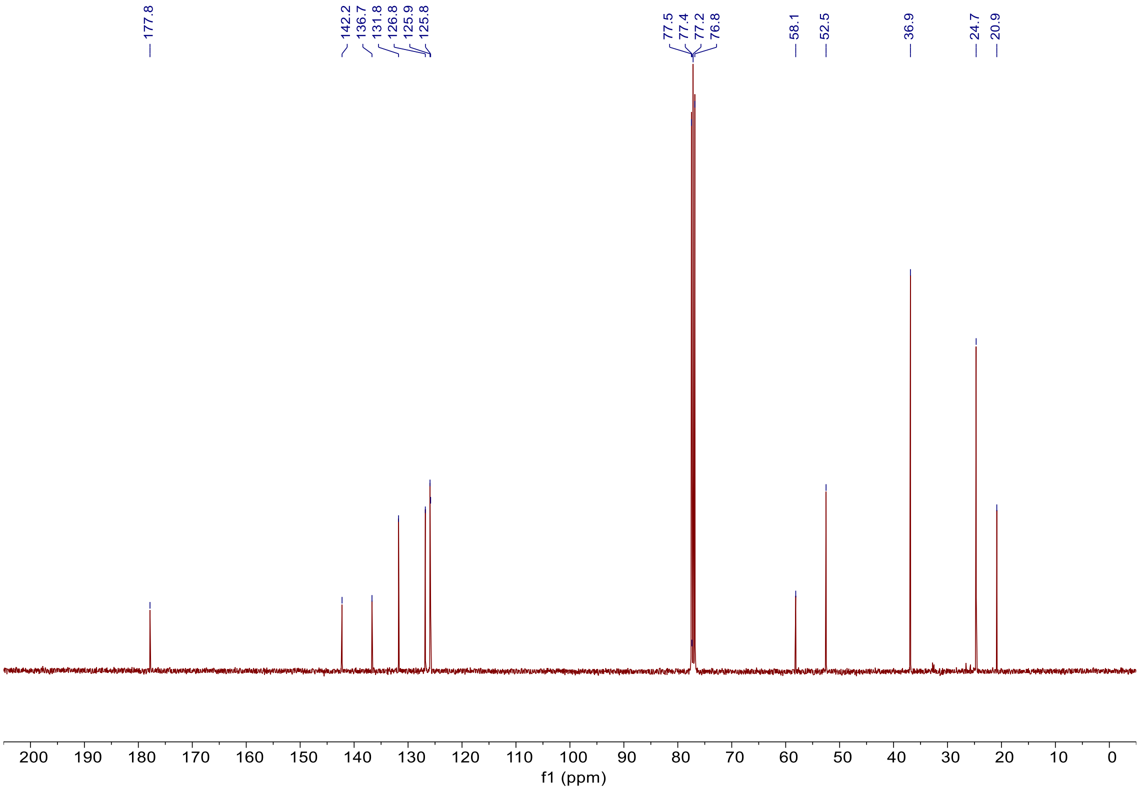

400 MHz ^{1}H NMR spectrum; 100.6 MHz ^{13}C NMR spectrum; $CDCl_3$ of **4p**

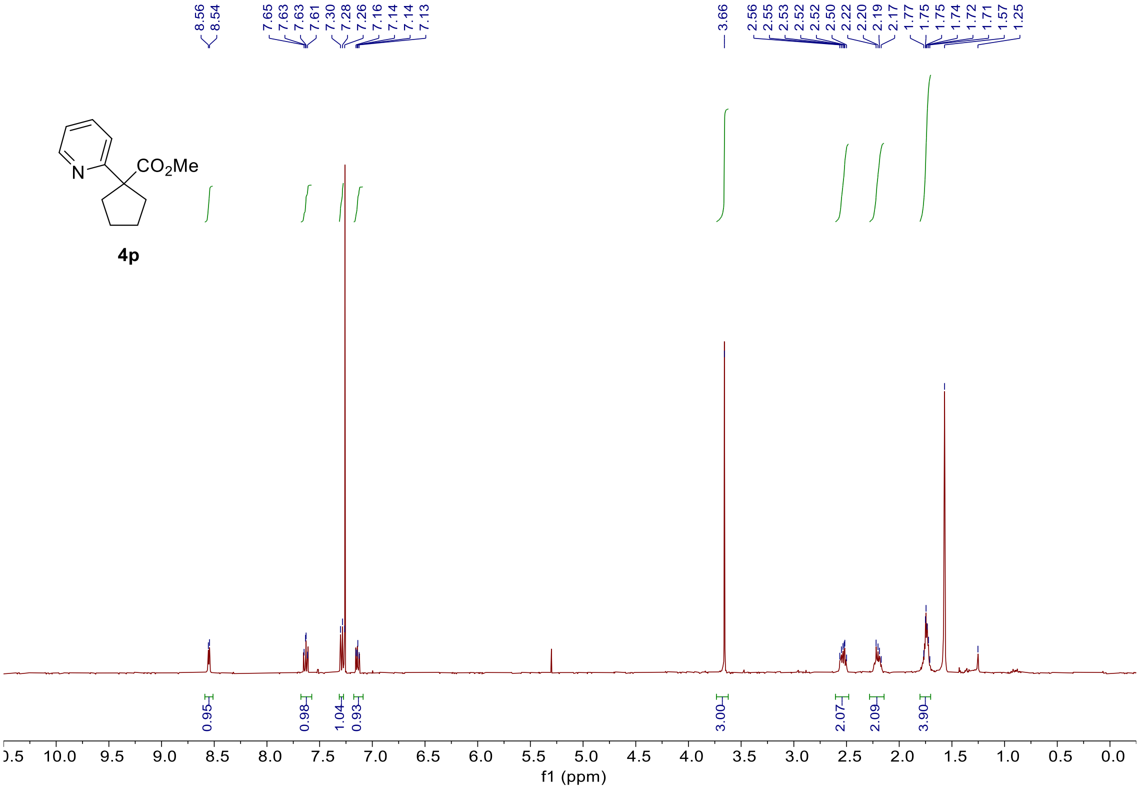

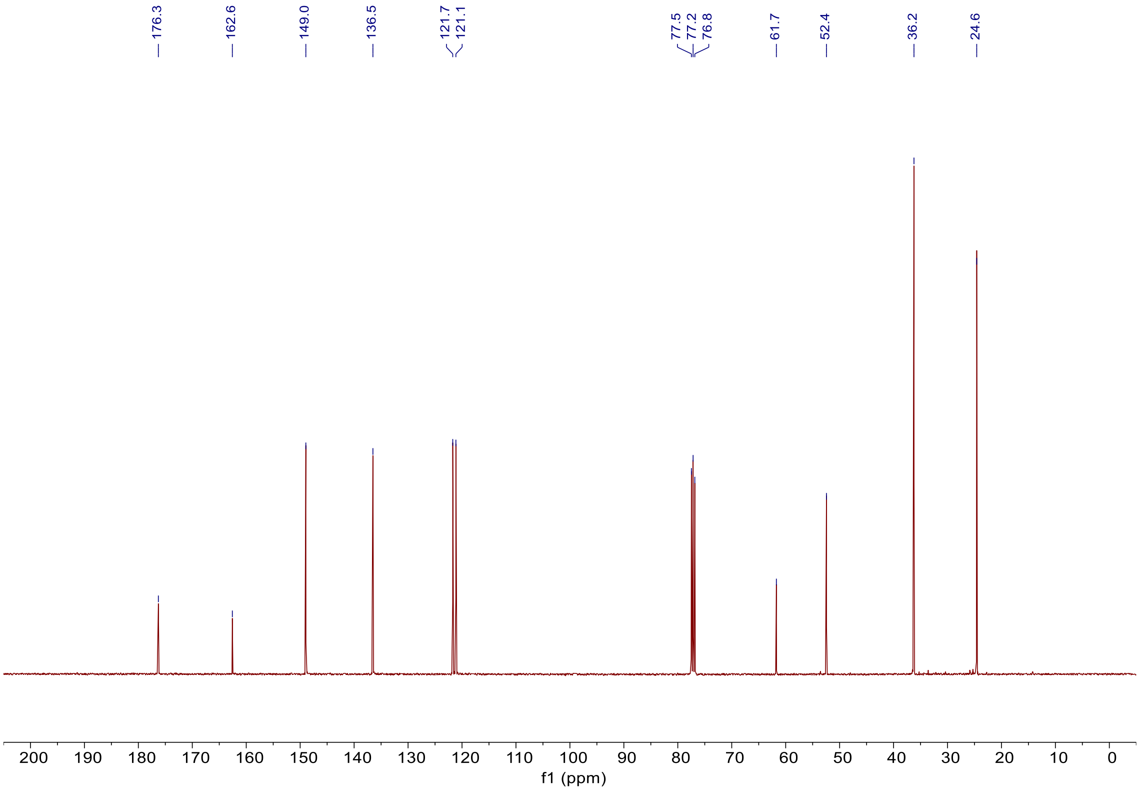

400 MHz ^{1}H NMR spectrum; 100.6 MHz ^{13}C NMR spectrum; $CDCl_3$ of **4q**

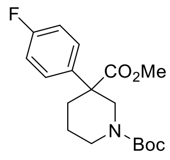

4q

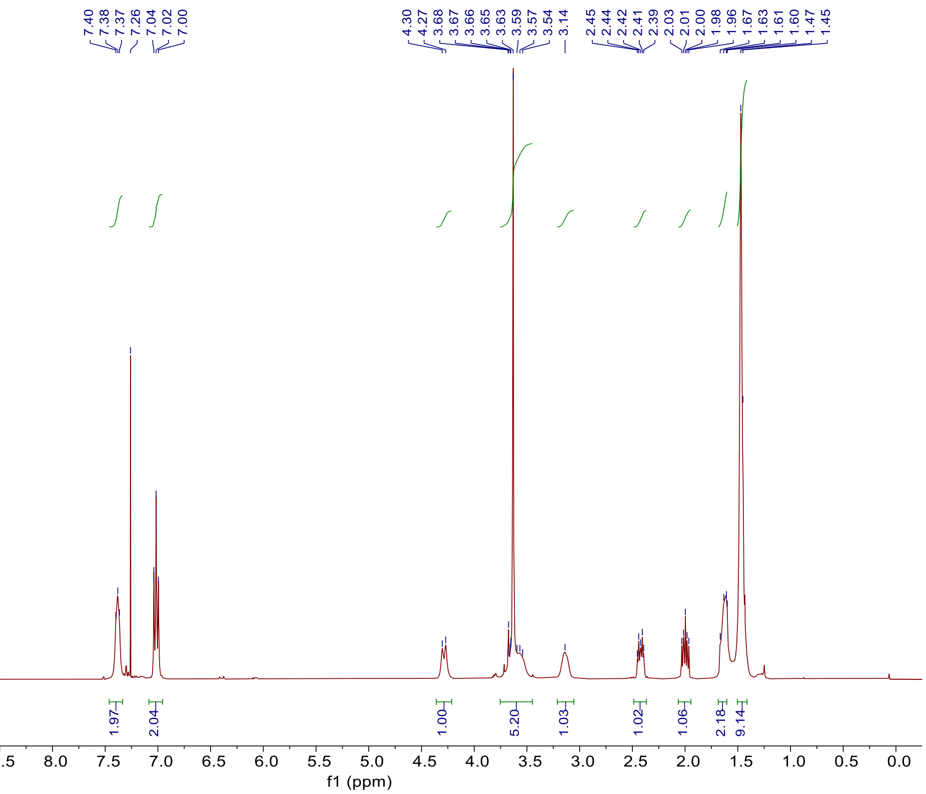

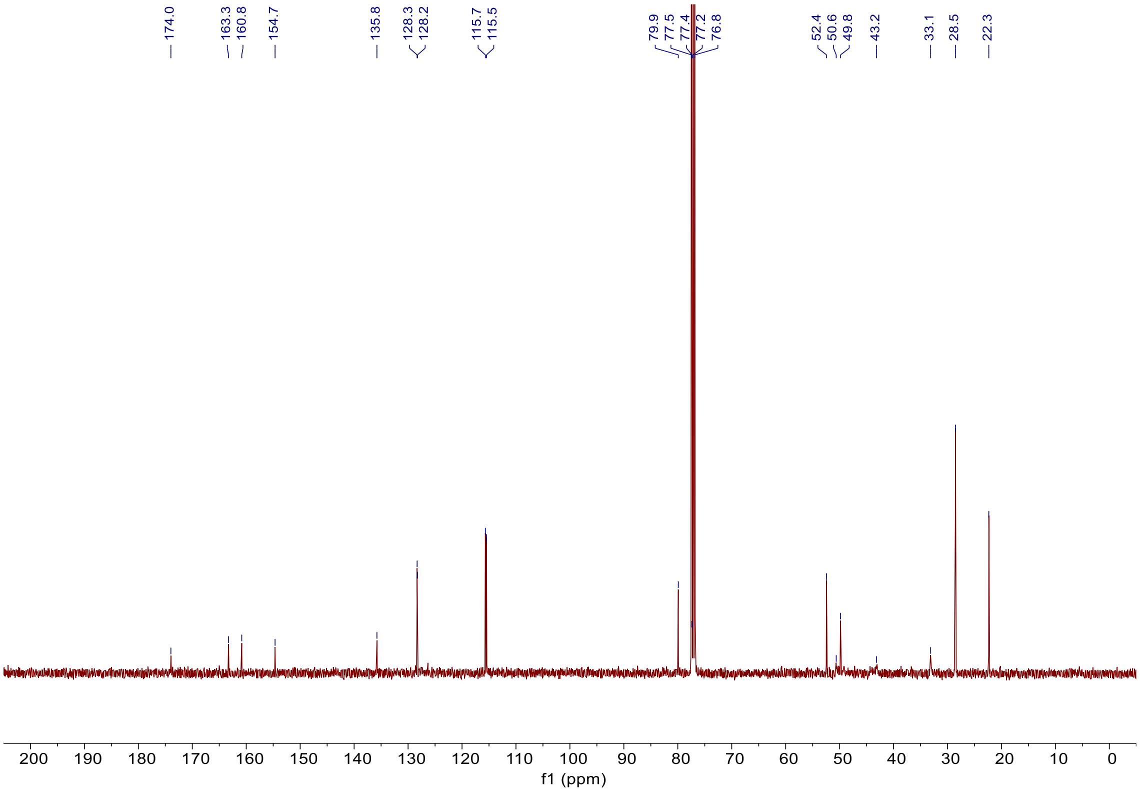

400 MHz ^{1}H NMR spectrum; 100.6 MHz ^{13}C NMR spectrum; $CDCl_3$ of **4r**

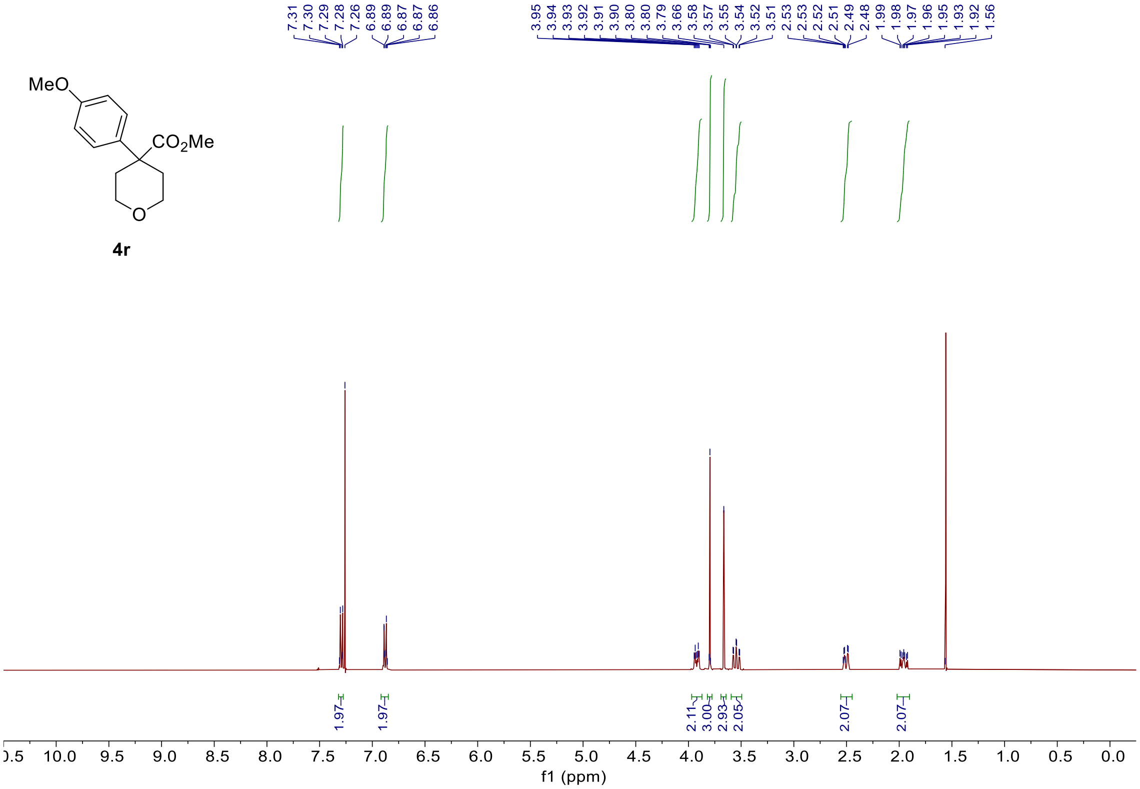

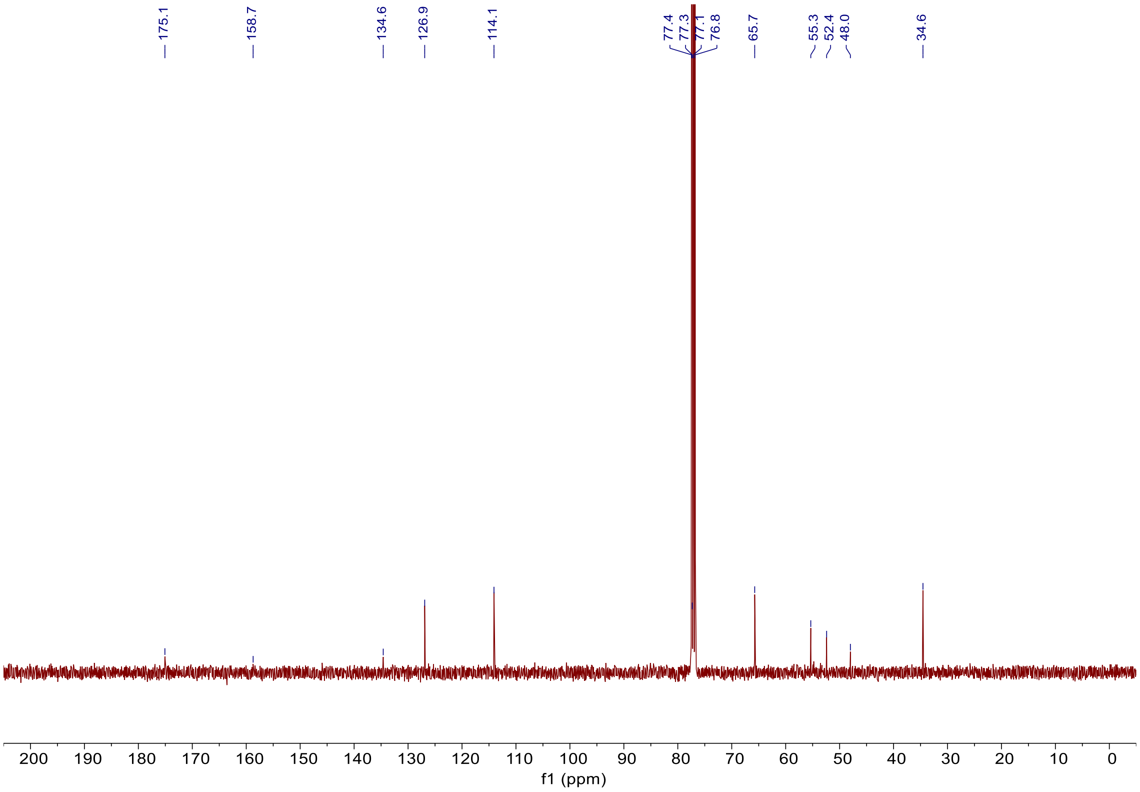

400 MHz ^{1}H NMR spectrum; 100.6 MHz ^{13}C NMR spectrum; $CDCl_3$ of **4s**

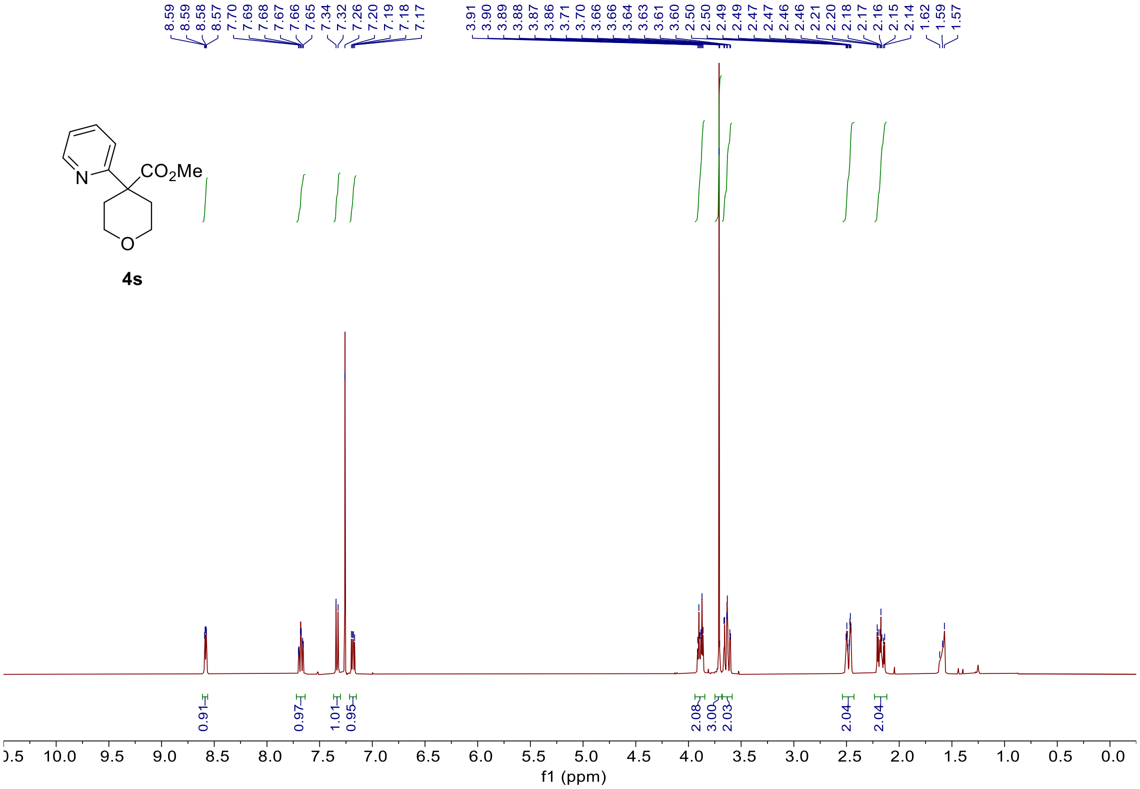

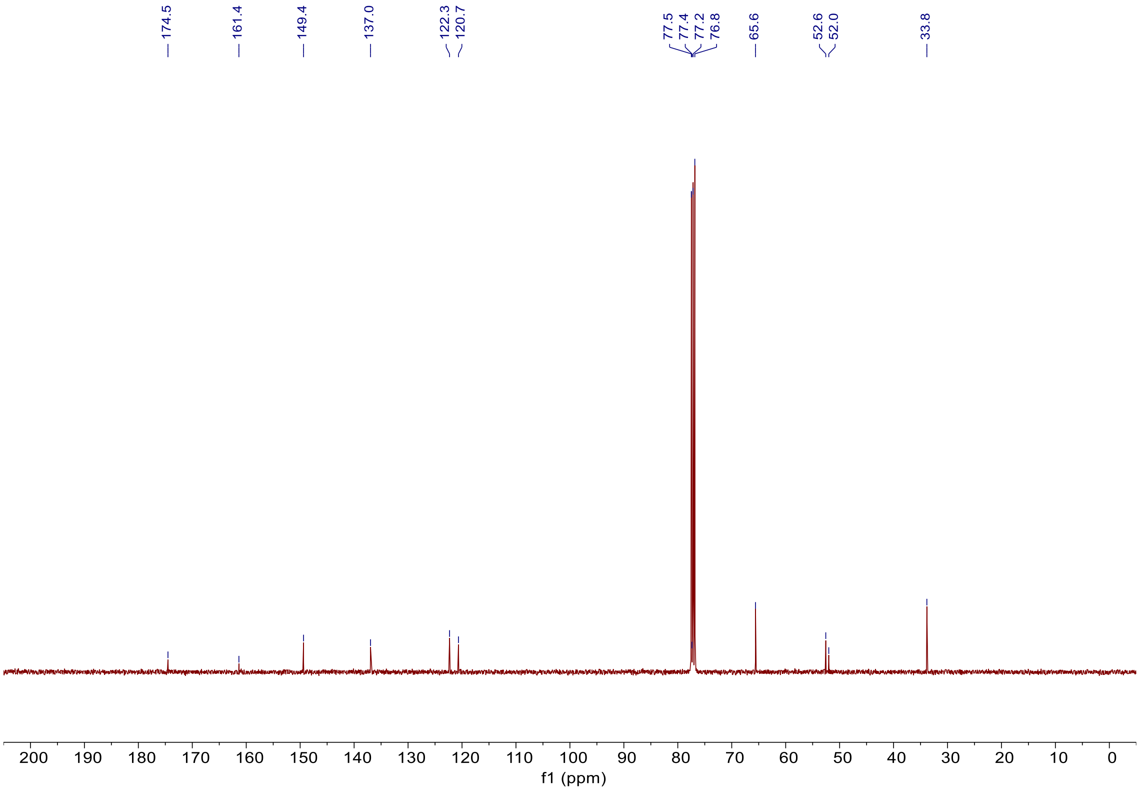

400 MHz ^{1}H NMR spectrum; 100.6 MHz ^{13}C NMR spectrum; $CDCl_3$ of **5a**

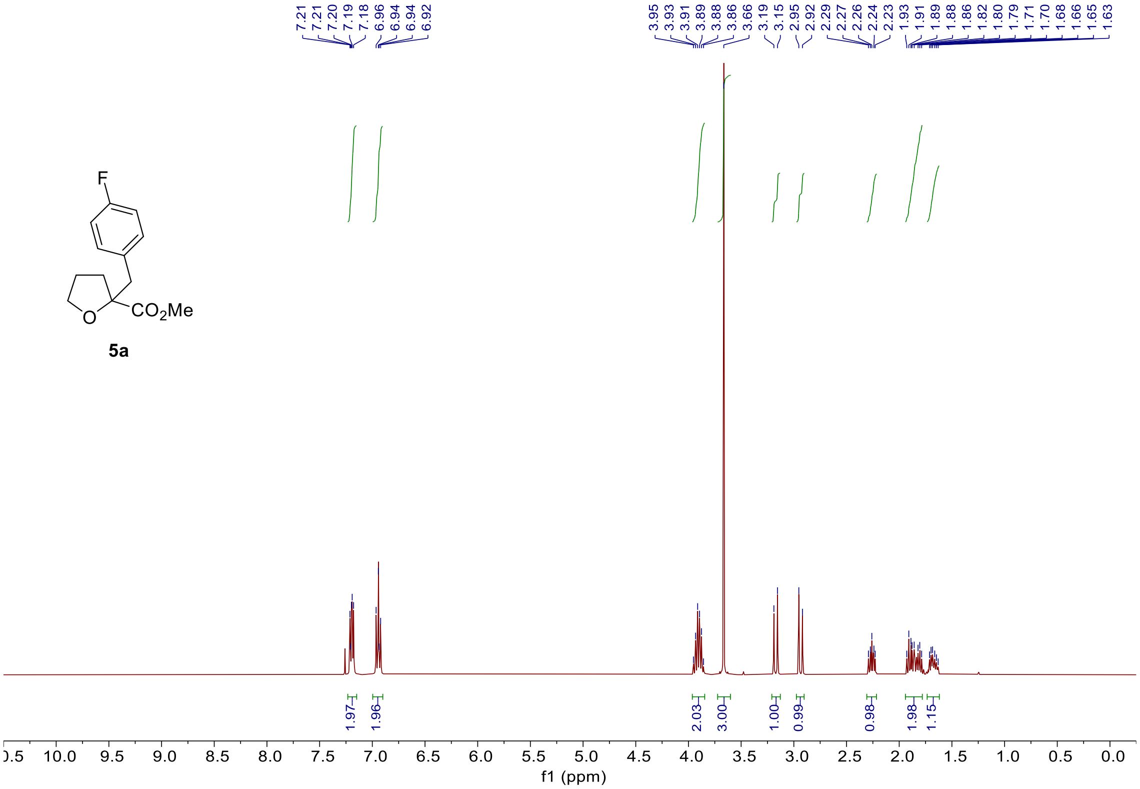

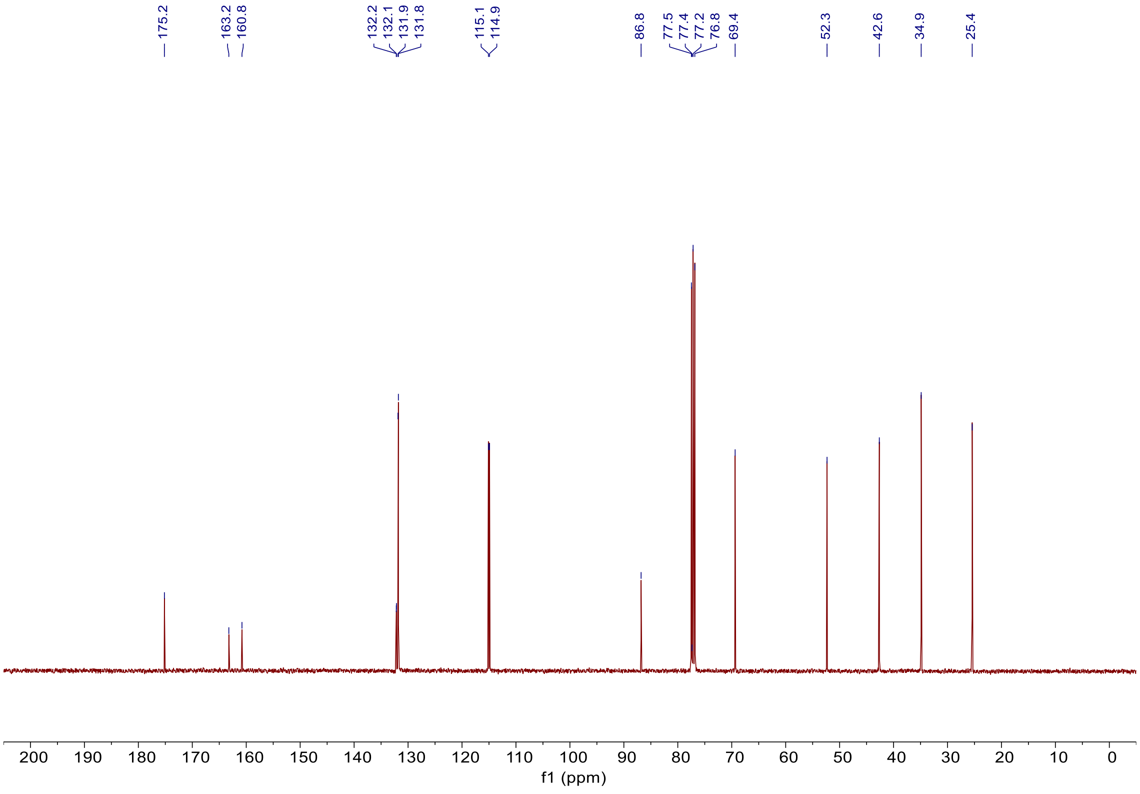

400 MHz ^{1}H NMR spectrum; 100.6 MHz ^{13}C NMR spectrum; $CDCl_3$ of **5b**

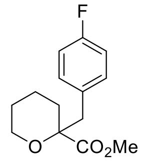

5b

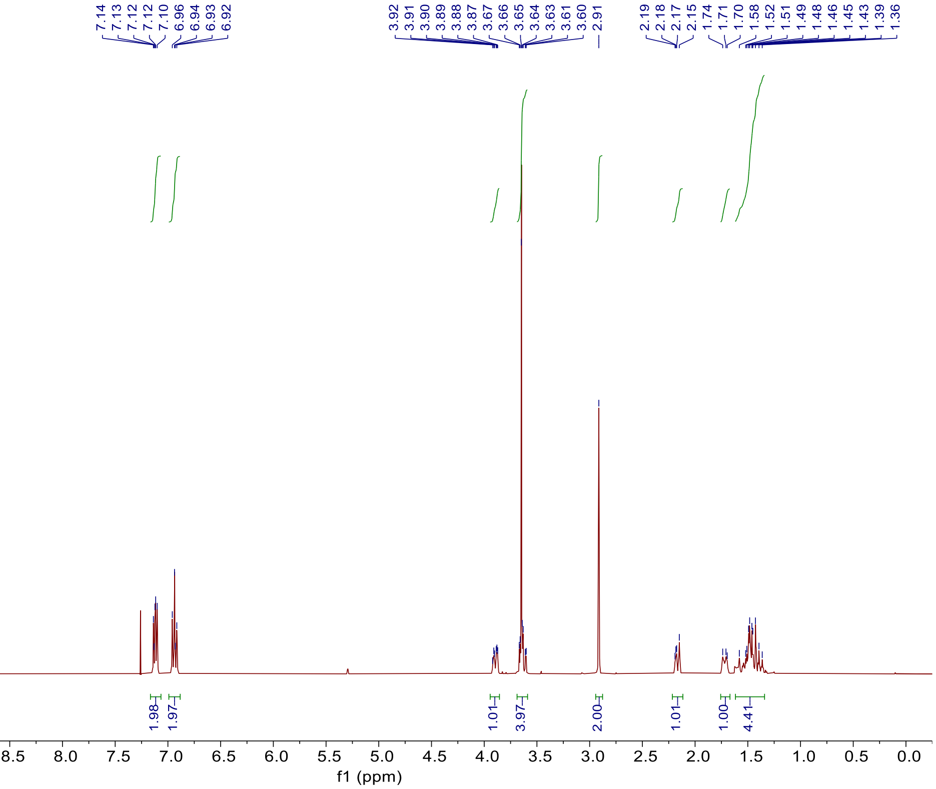

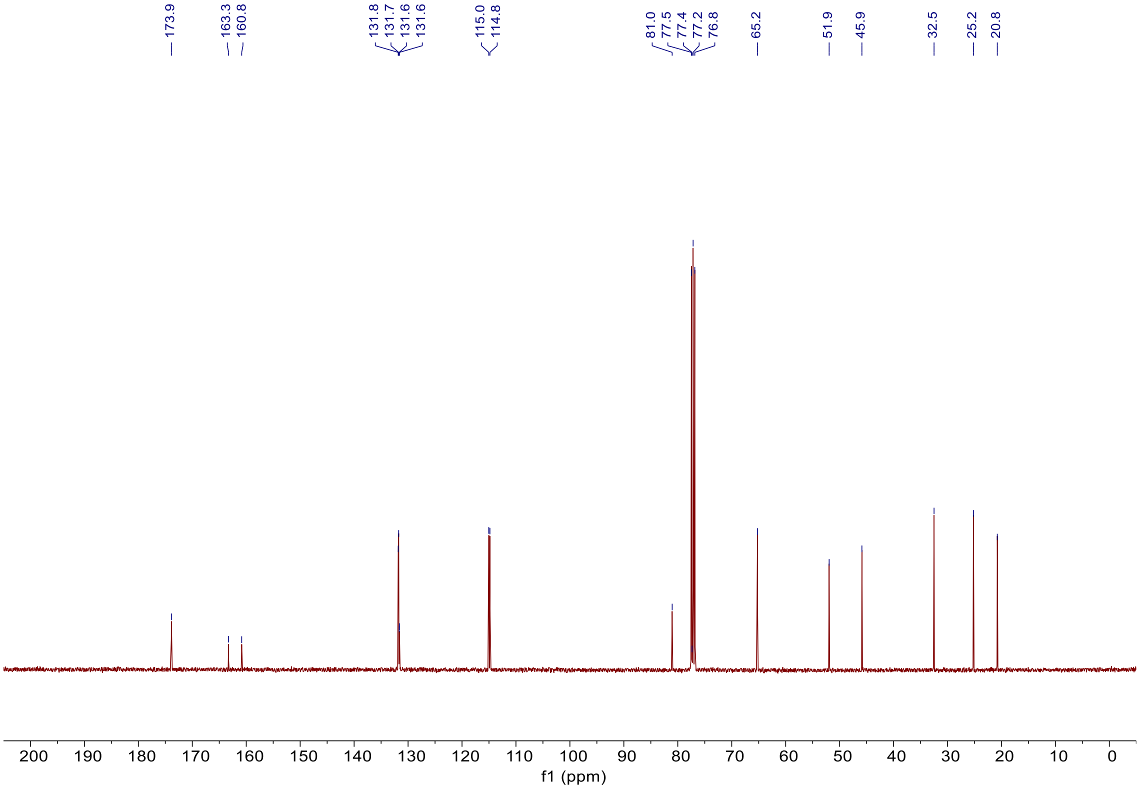

400 MHz ^{1}H NMR spectrum; 100.6 MHz ^{13}C NMR spectrum; $CDCl_3$ of **5c**

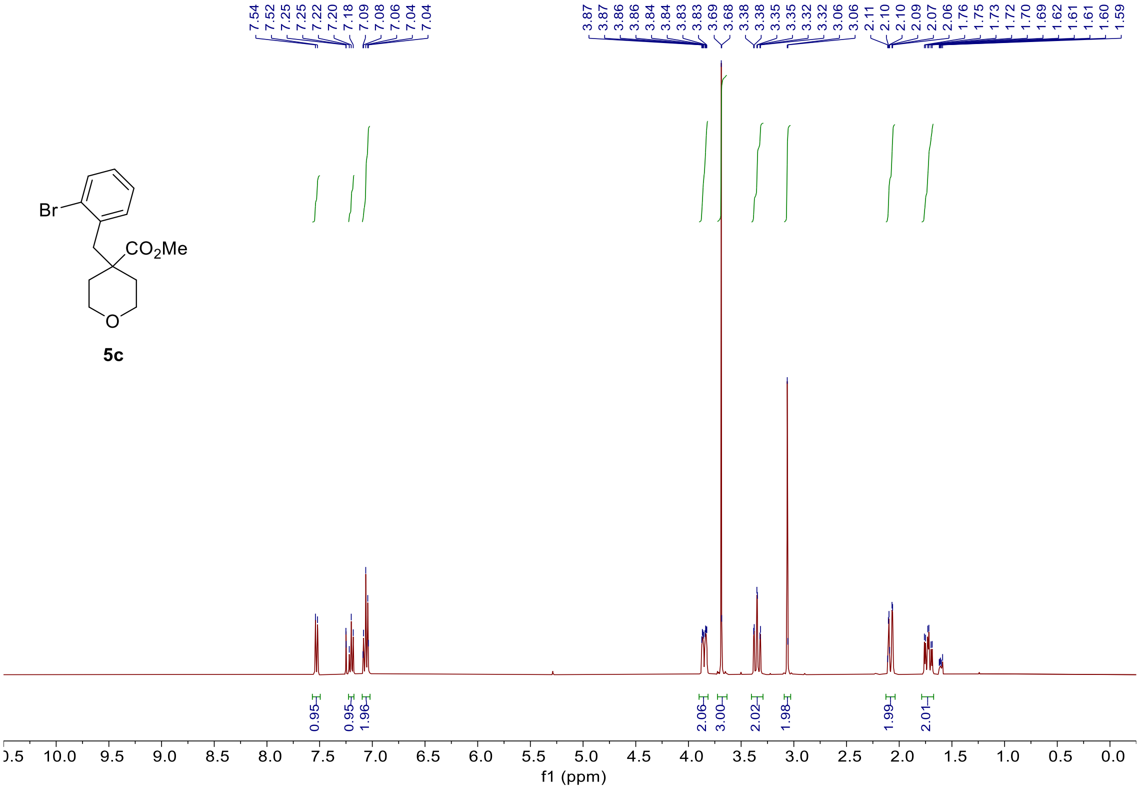

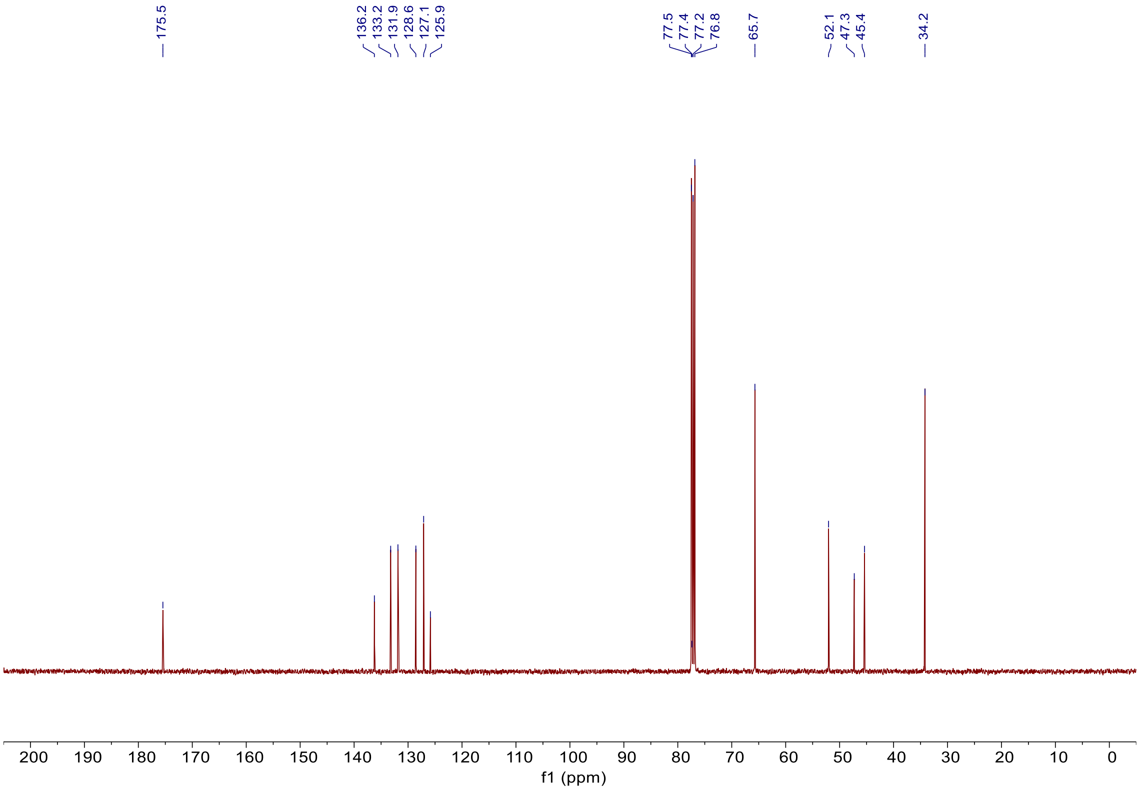

400 MHz ^{1}H NMR spectrum; 100.6 MHz ^{13}C NMR spectrum; $CDCl_3$ of **5d**

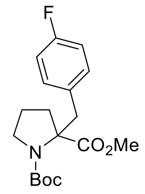

5d

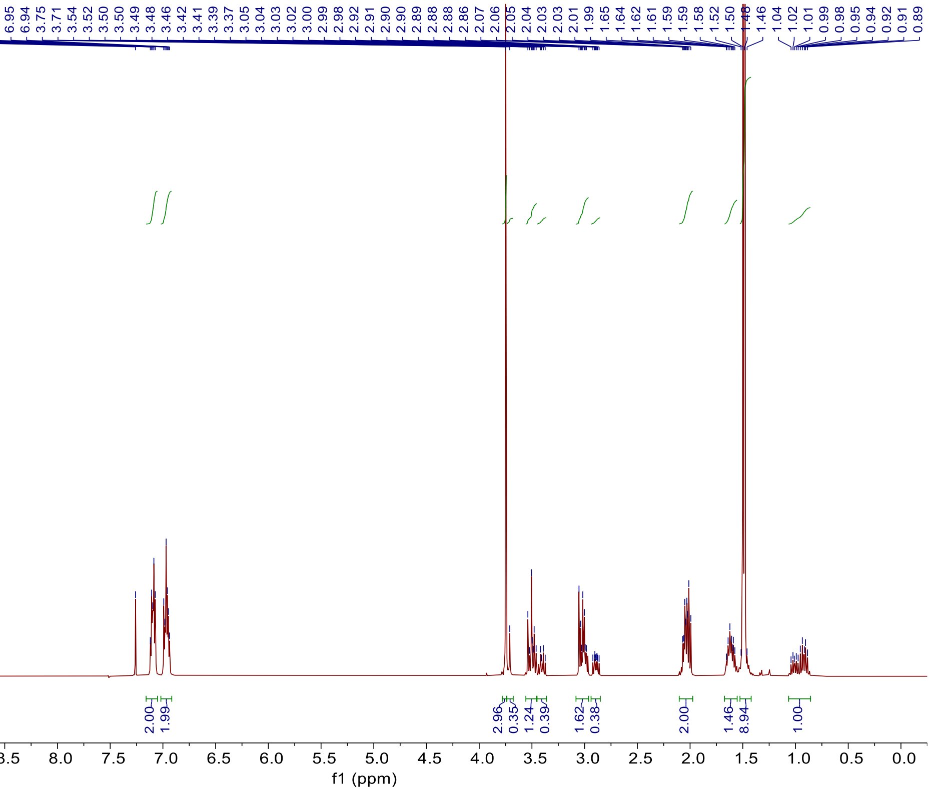

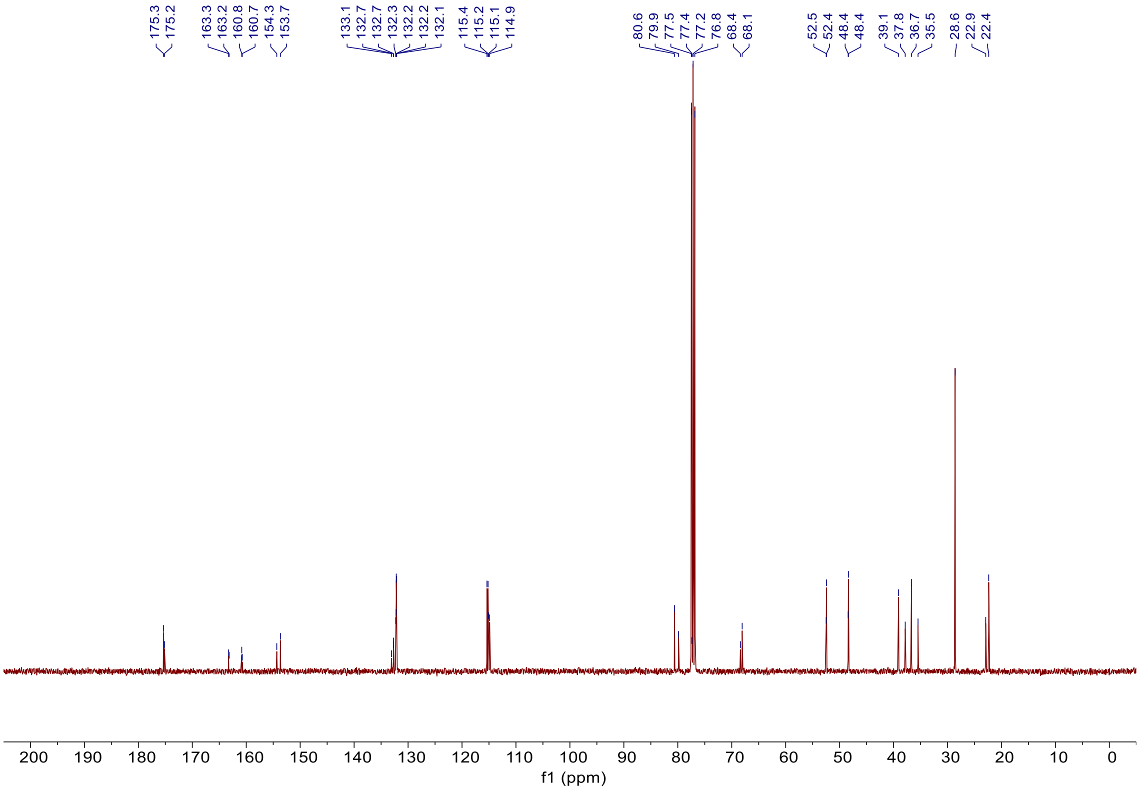

400 MHz ^{1}H NMR spectrum; 100.6 MHz ^{13}C NMR spectrum; $CDCl_3$ of **5e**

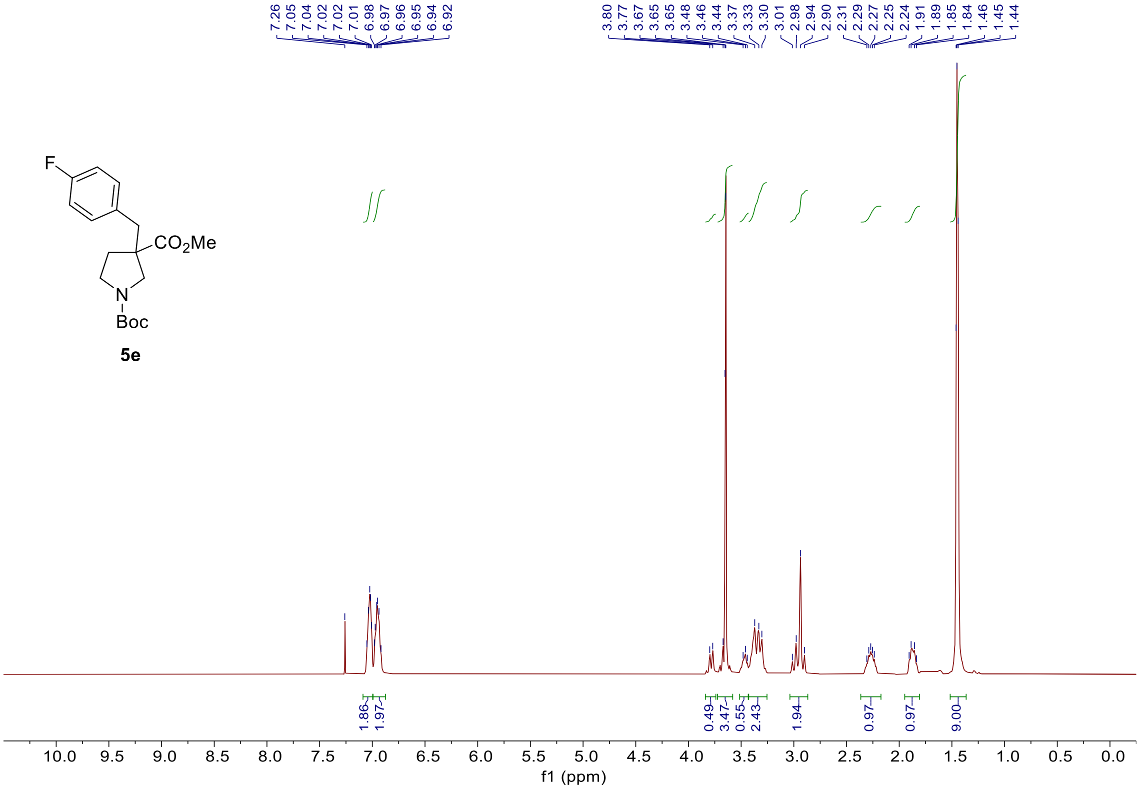

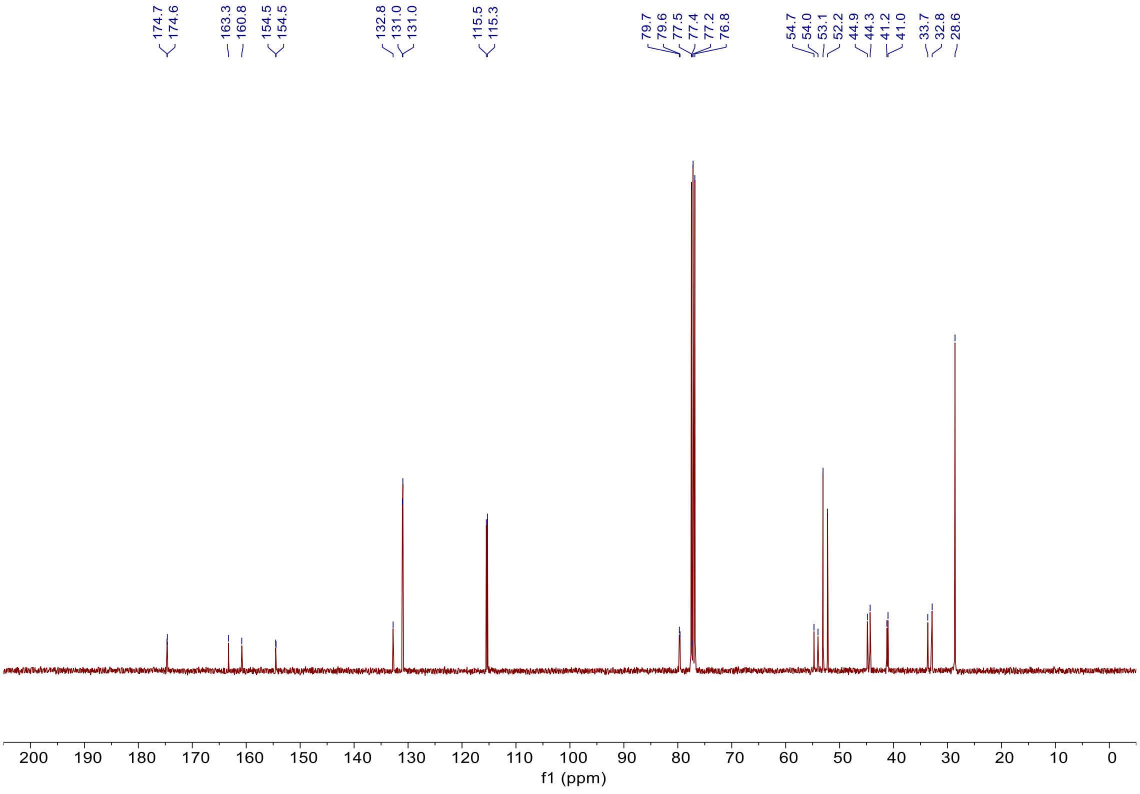

400 MHz ^{1}H NMR spectrum; 100.6 MHz ^{13}C NMR spectrum; $CDCl_3$ of **5f**

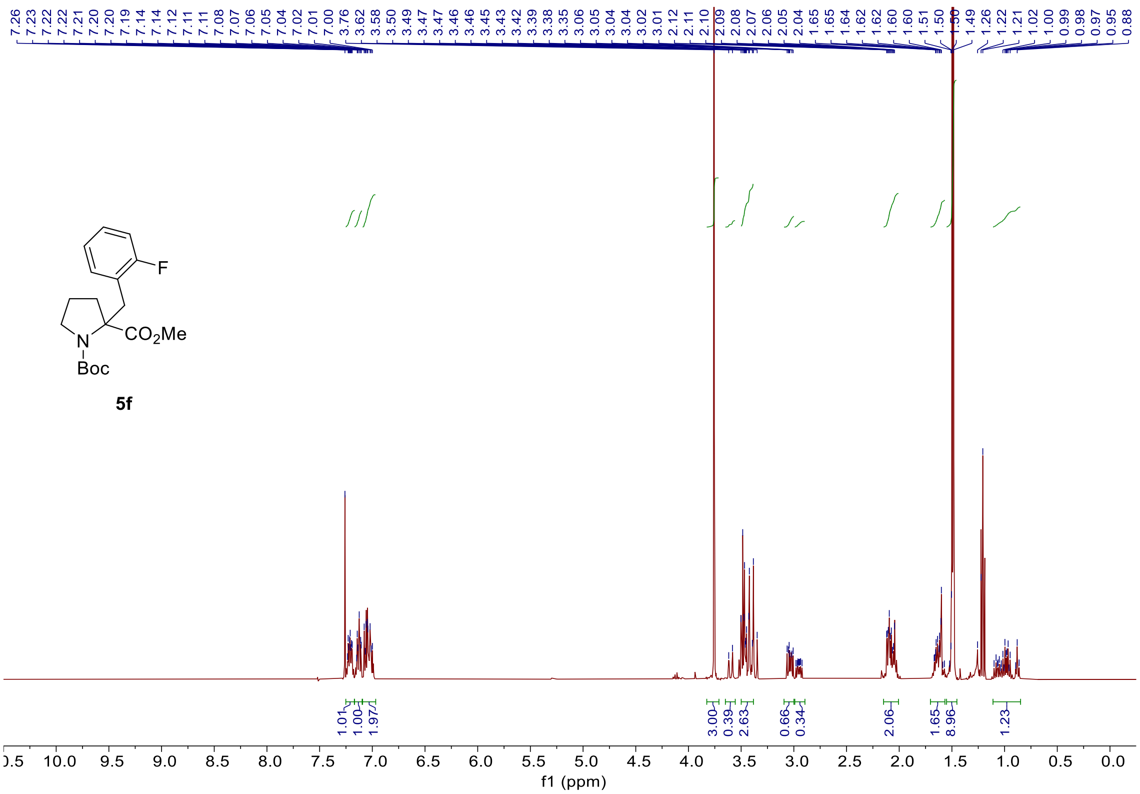

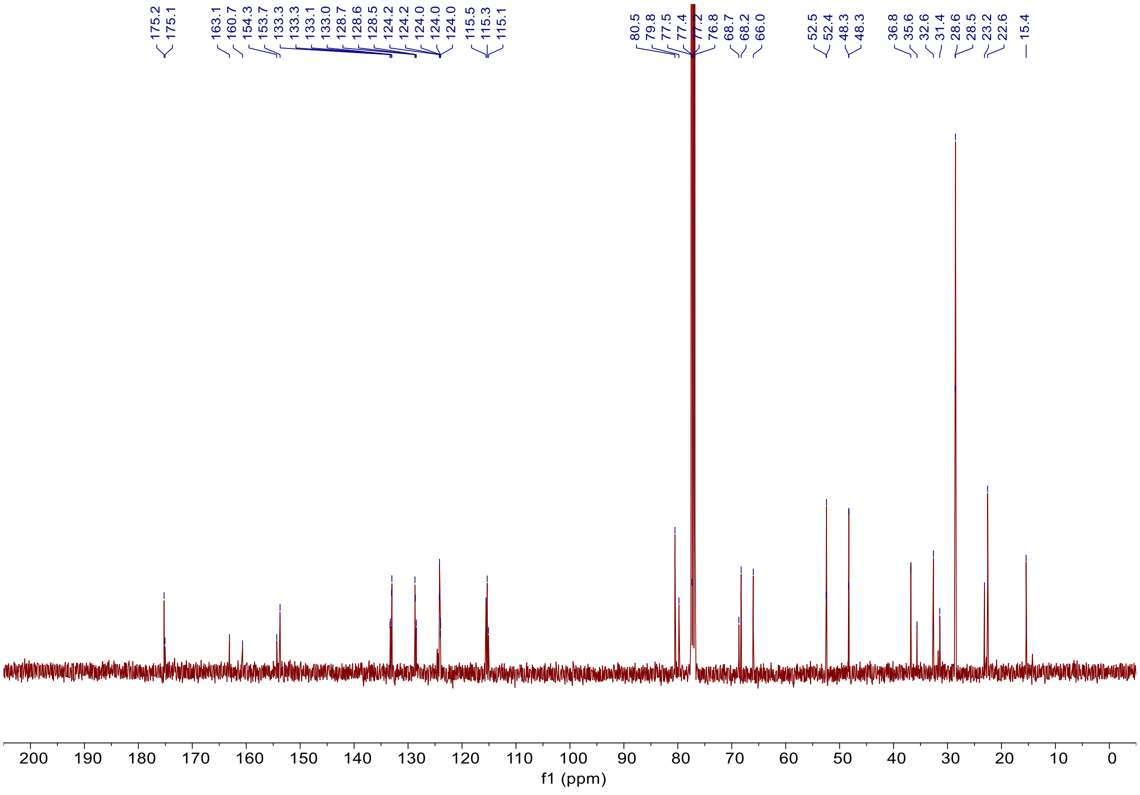

400 MHz ^{1}H NMR spectrum; 100.6 MHz ^{13}C NMR spectrum; $CDCl_3$ of **5g**

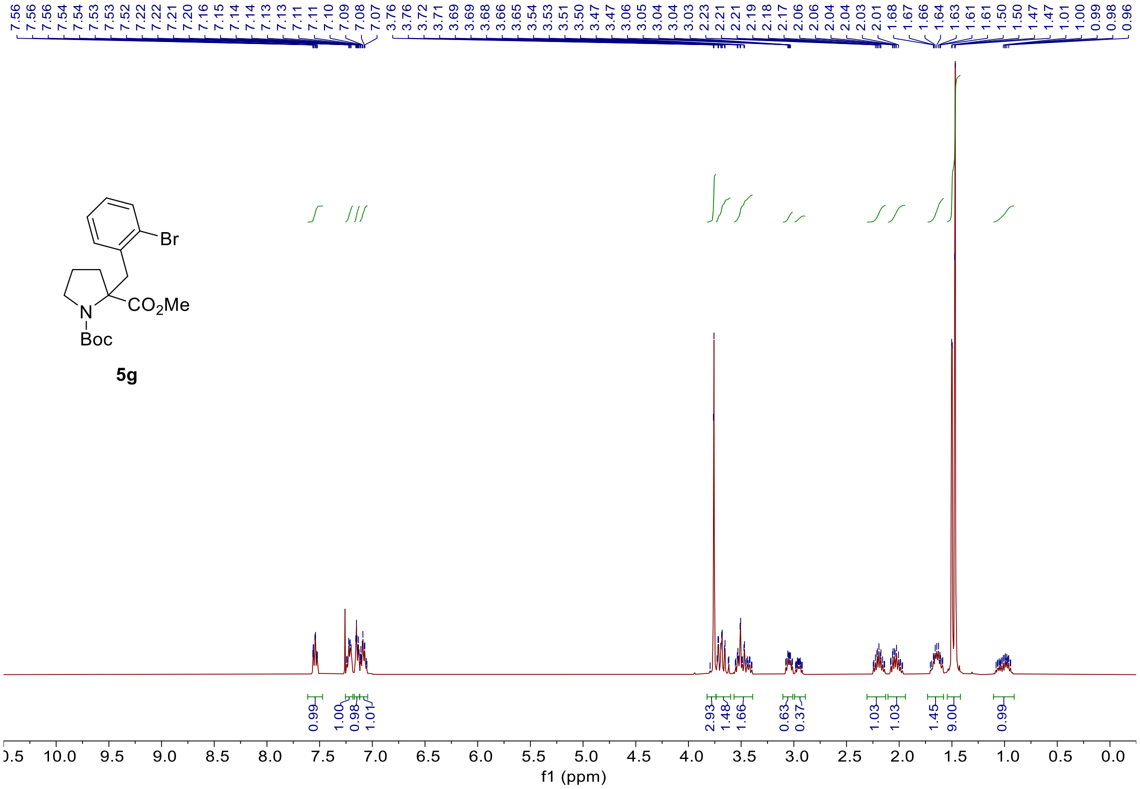

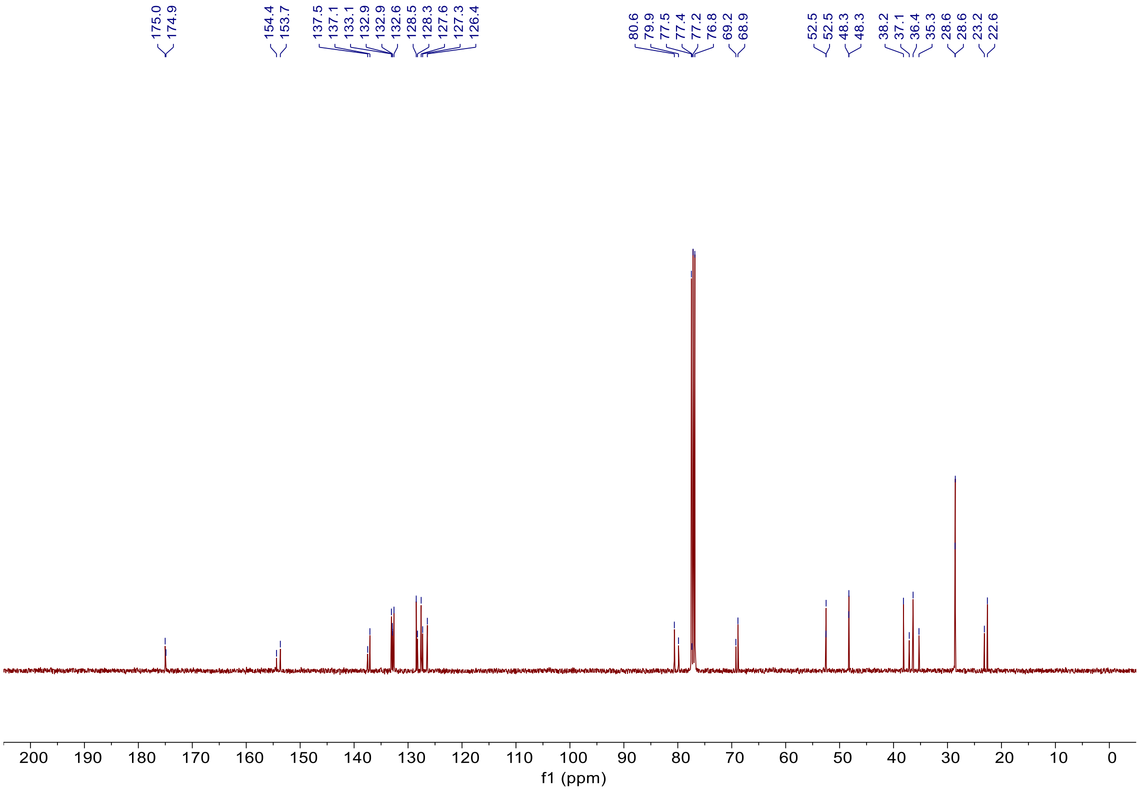

400 MHz ^{1}H NMR spectrum; 100.6 MHz ^{13}C NMR spectrum; $CDCl_3$ of **5h**

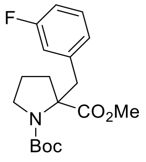

5h

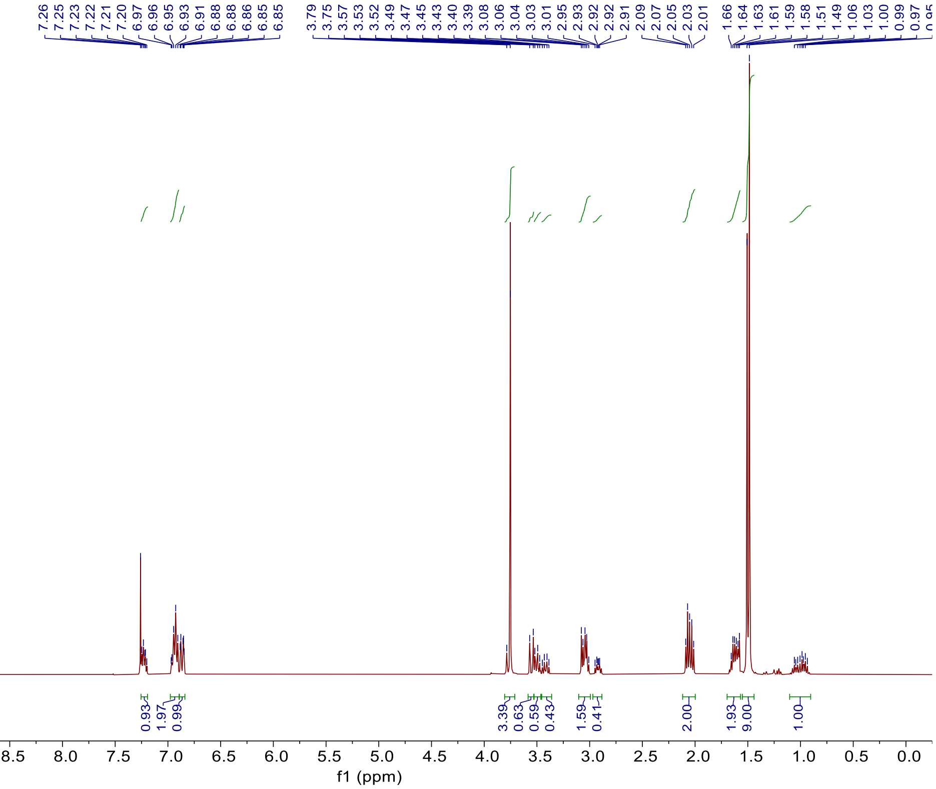

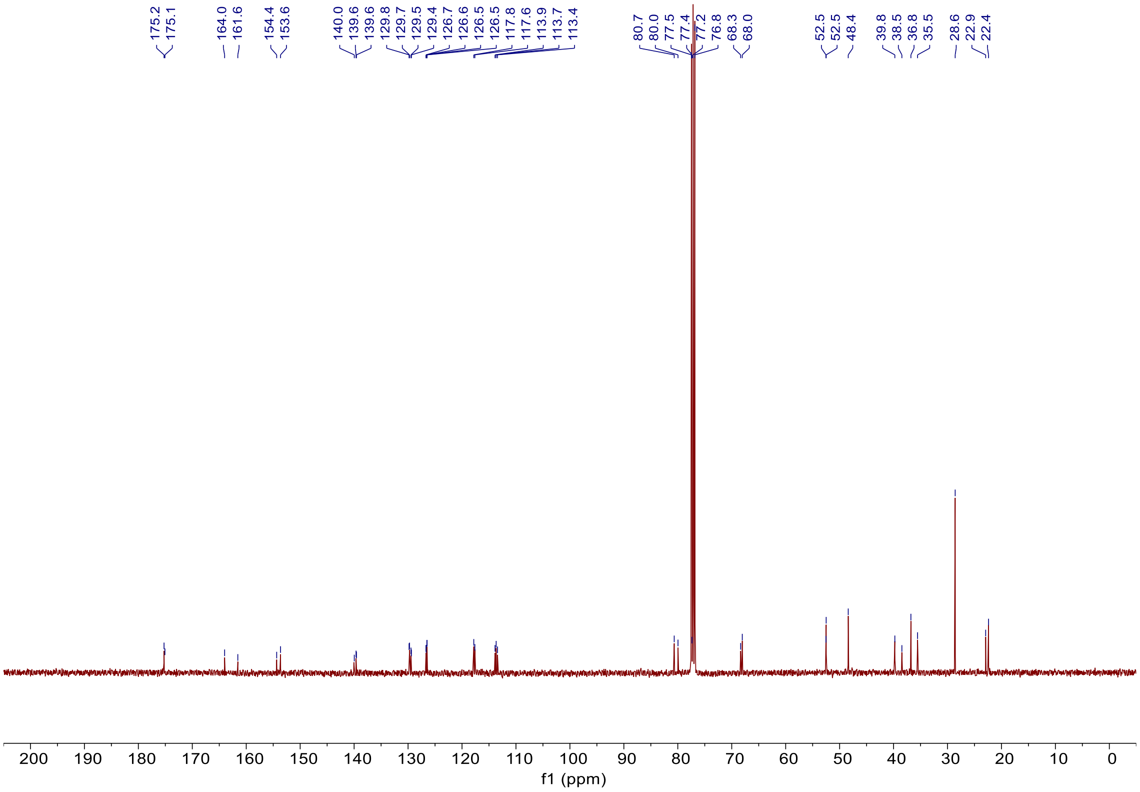

400 MHz ^{1}H NMR spectrum; 100.6 MHz ^{13}C NMR spectrum; $CDCl_3$ of **5i**

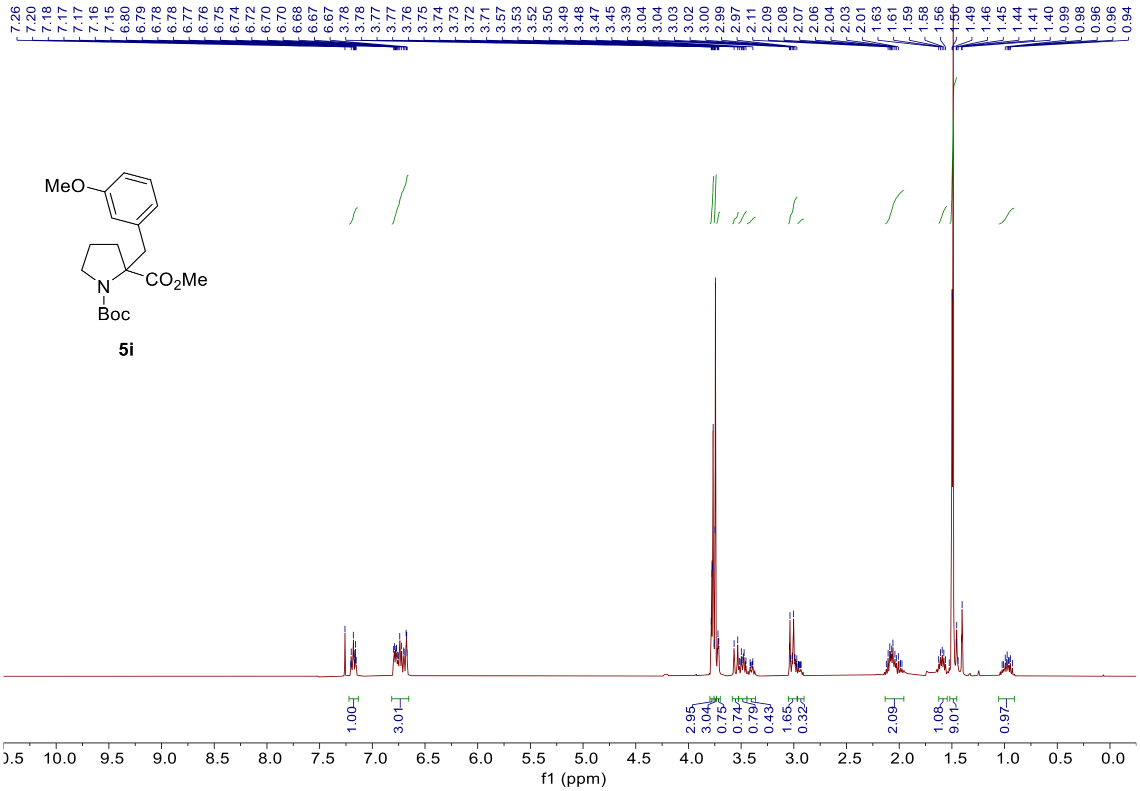

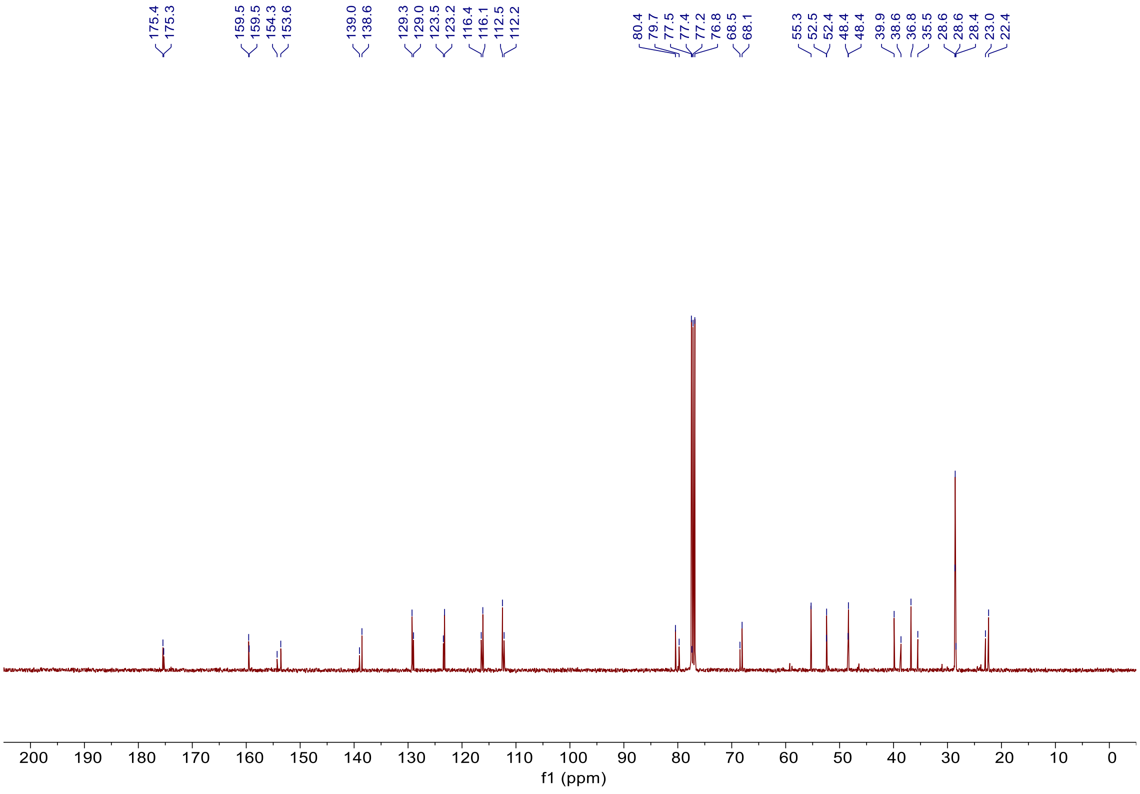

400 MHz ^{1}H NMR spectrum; 100.6 MHz ^{13}C NMR spectrum; $CDCl_3$of **5j**

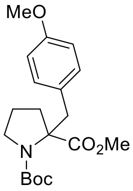

5j

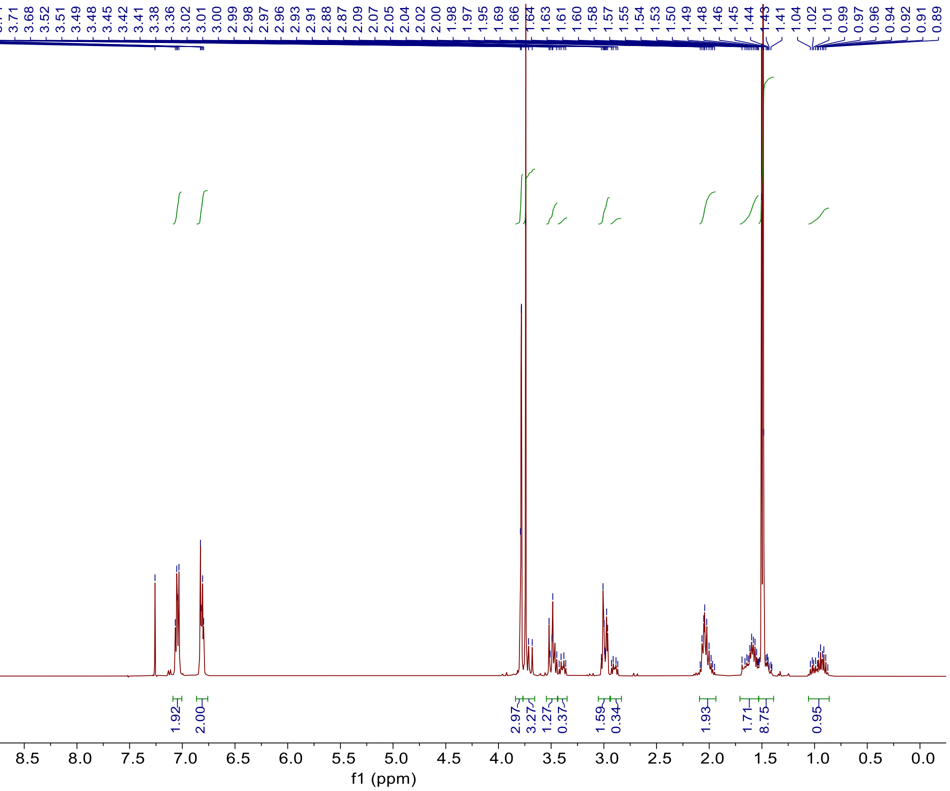

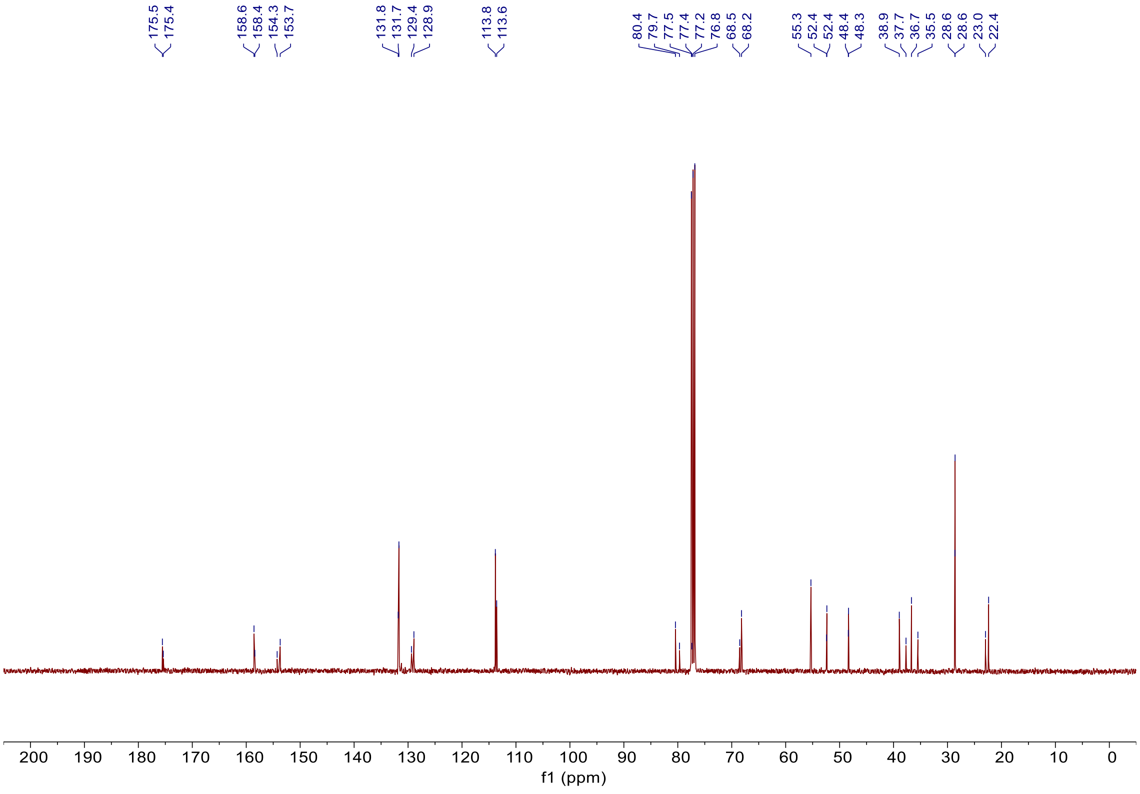

400 MHz ^1H NMR spectrum; 100.6 MHz ^{13}C NMR spectrum; $CDCl_3$ of **5k**

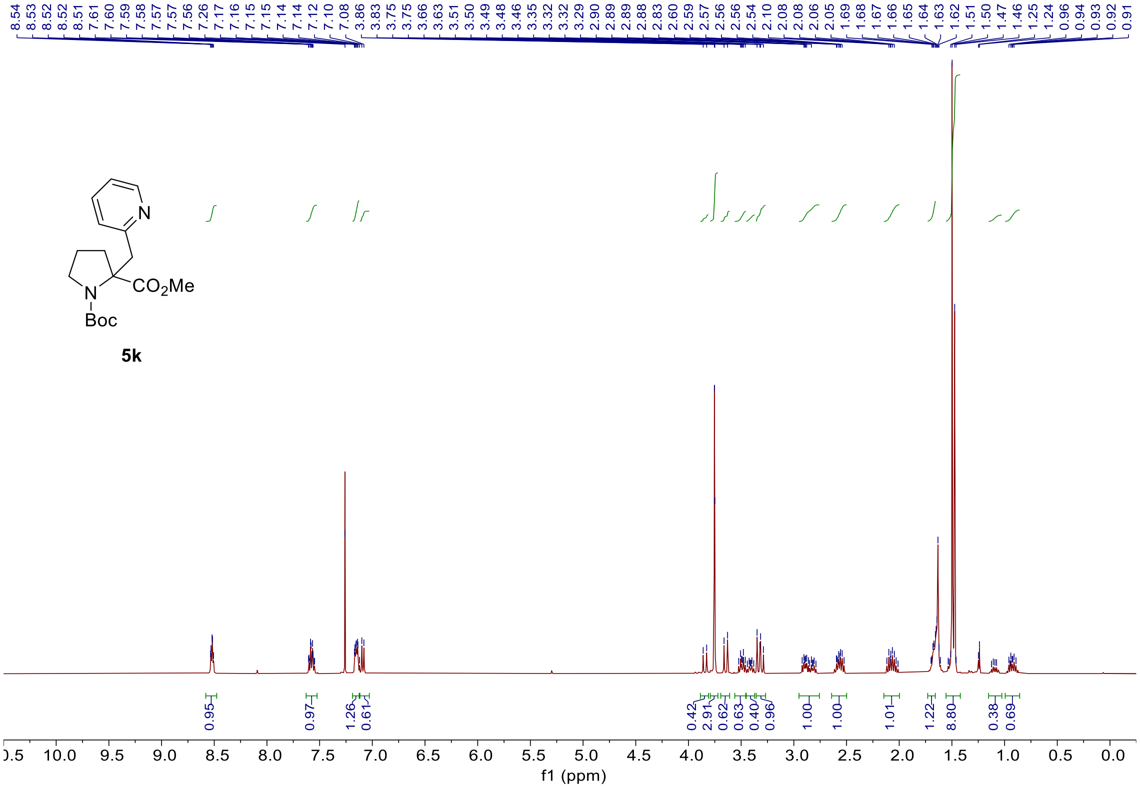

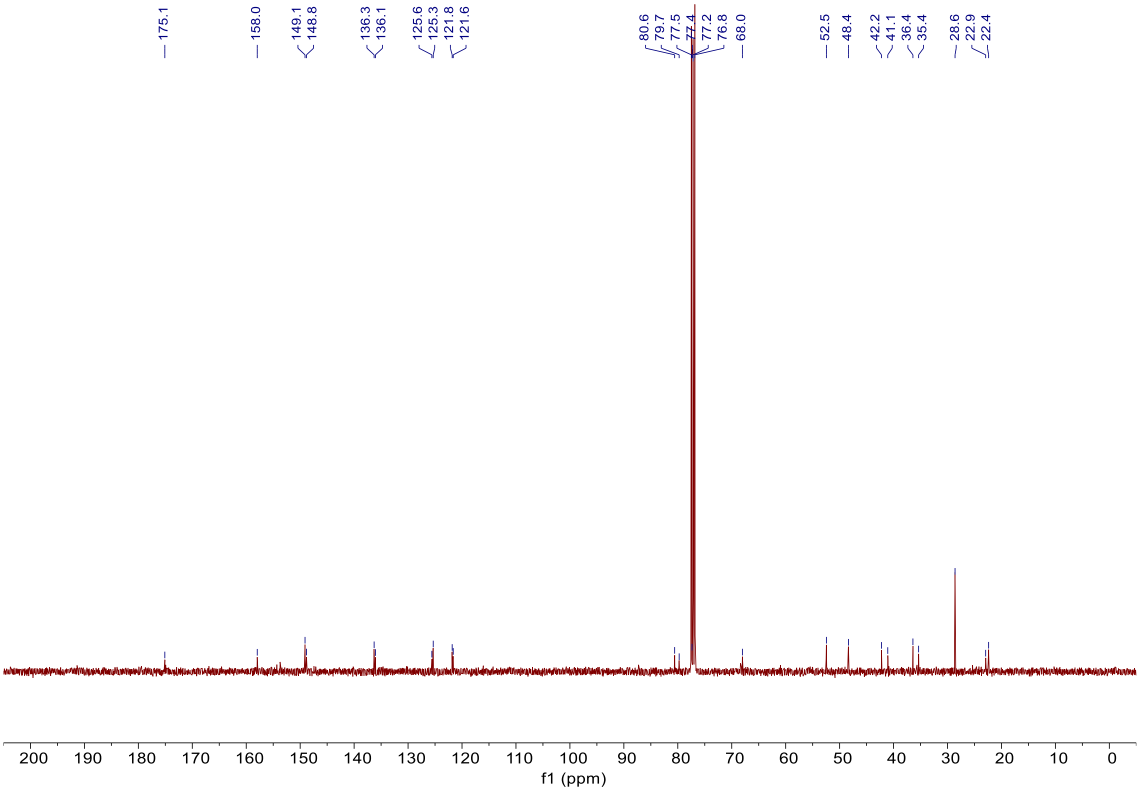

400 MHz ^{1}H NMR spectrum; 100.6 MHz ^{13}C NMR spectrum; MeOD-d_4 of **8a**•HCl

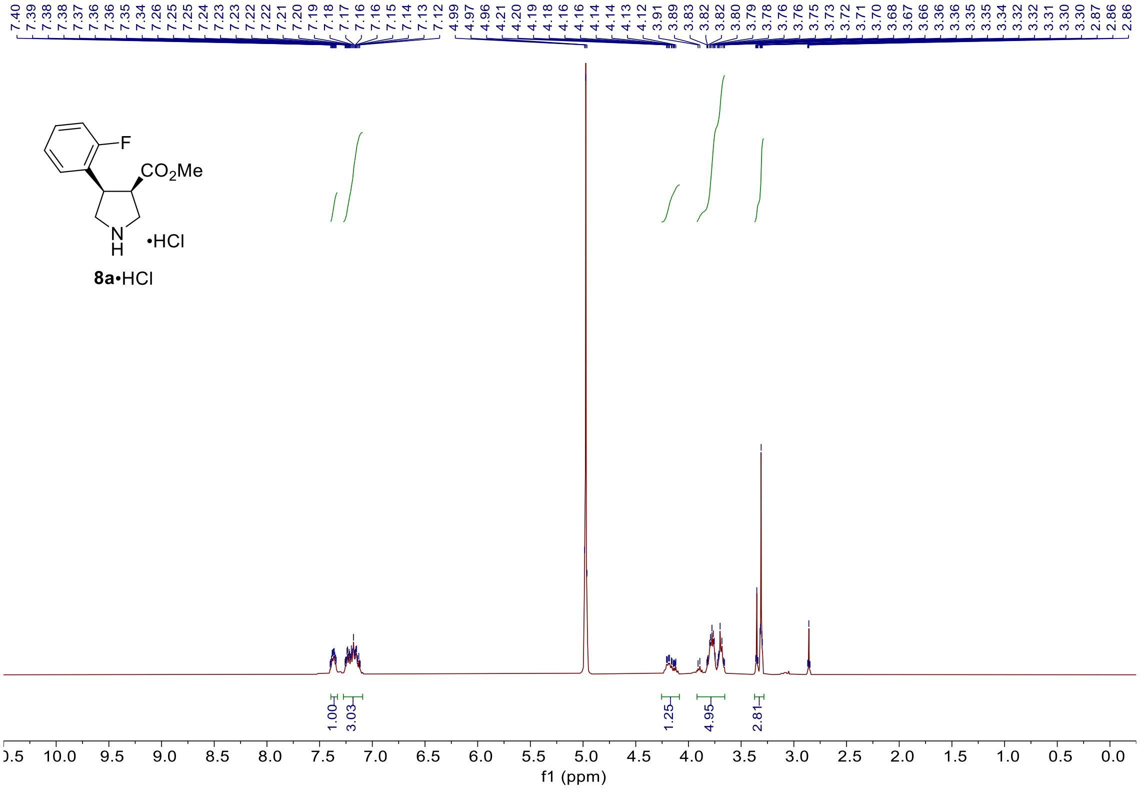

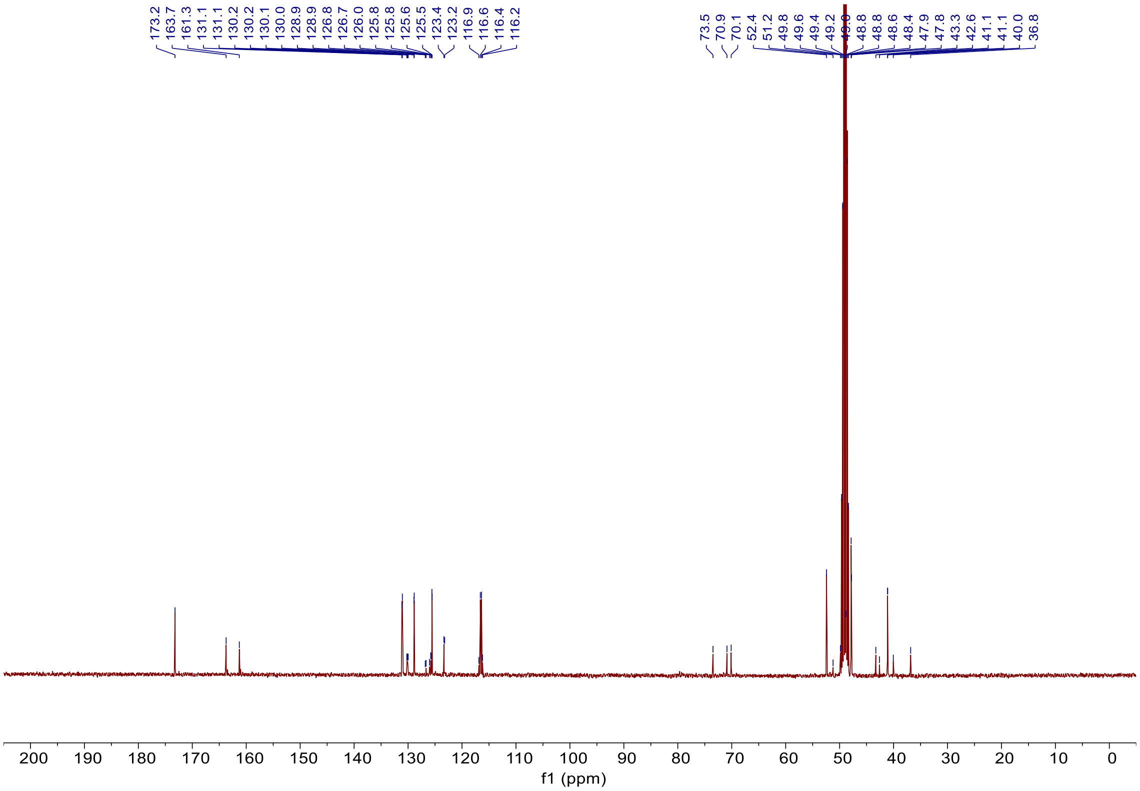

400 MHz ^{1}H NMR spectrum; 100.6 MHz ^{13}C NMR spectrum; MeOD-d_4 of **8b**•HCl

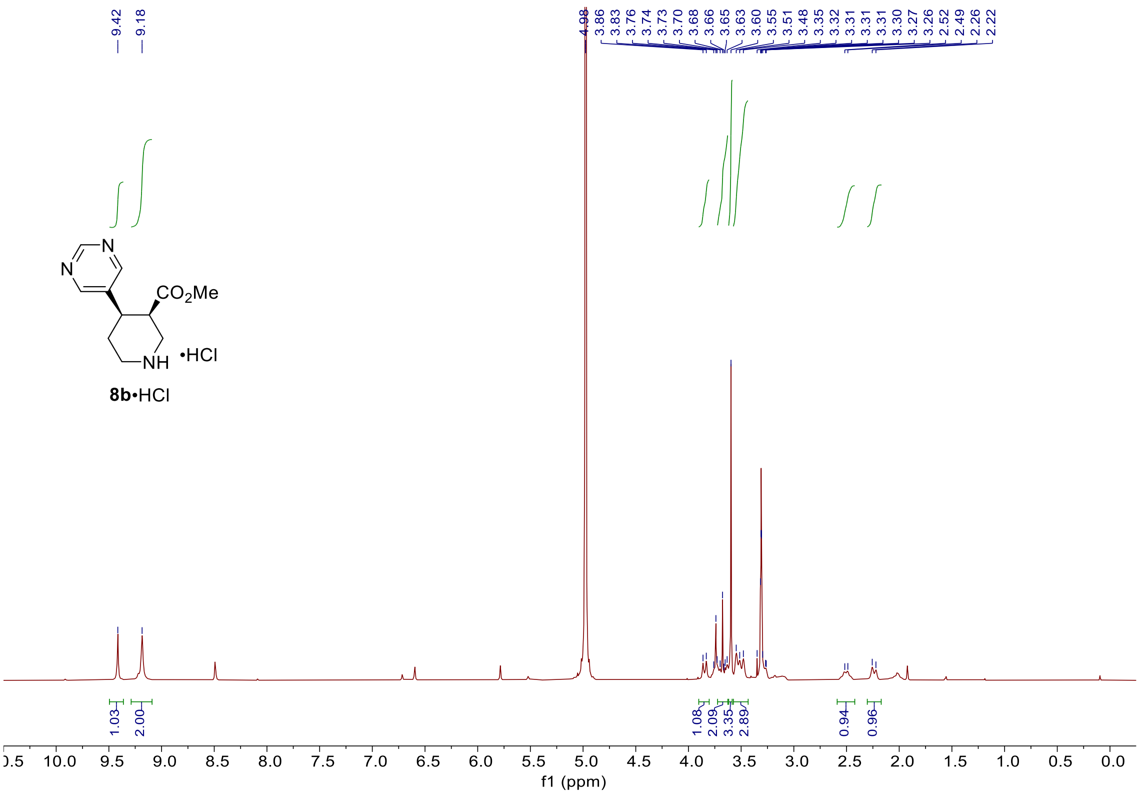

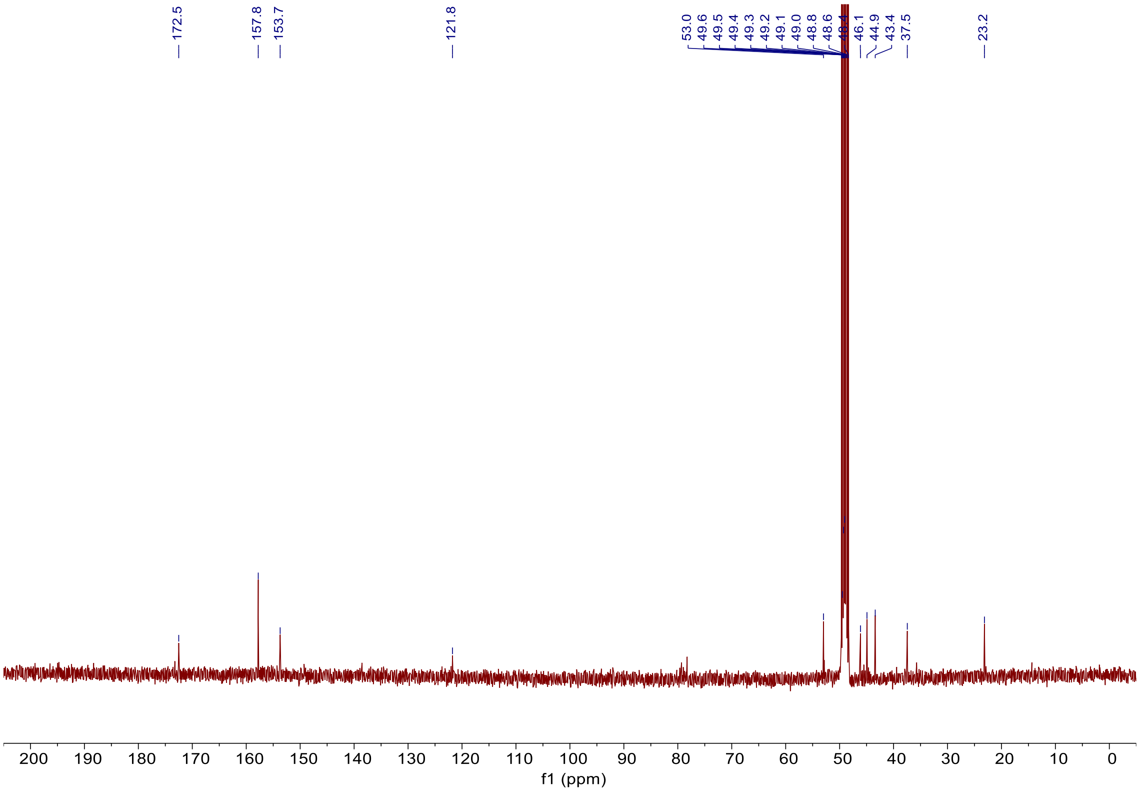

400 MHz ^{1}H NMR spectrum; 100.6 MHz ^{13}C NMR spectrum; $CDCl_3$ of **8c**•HCl

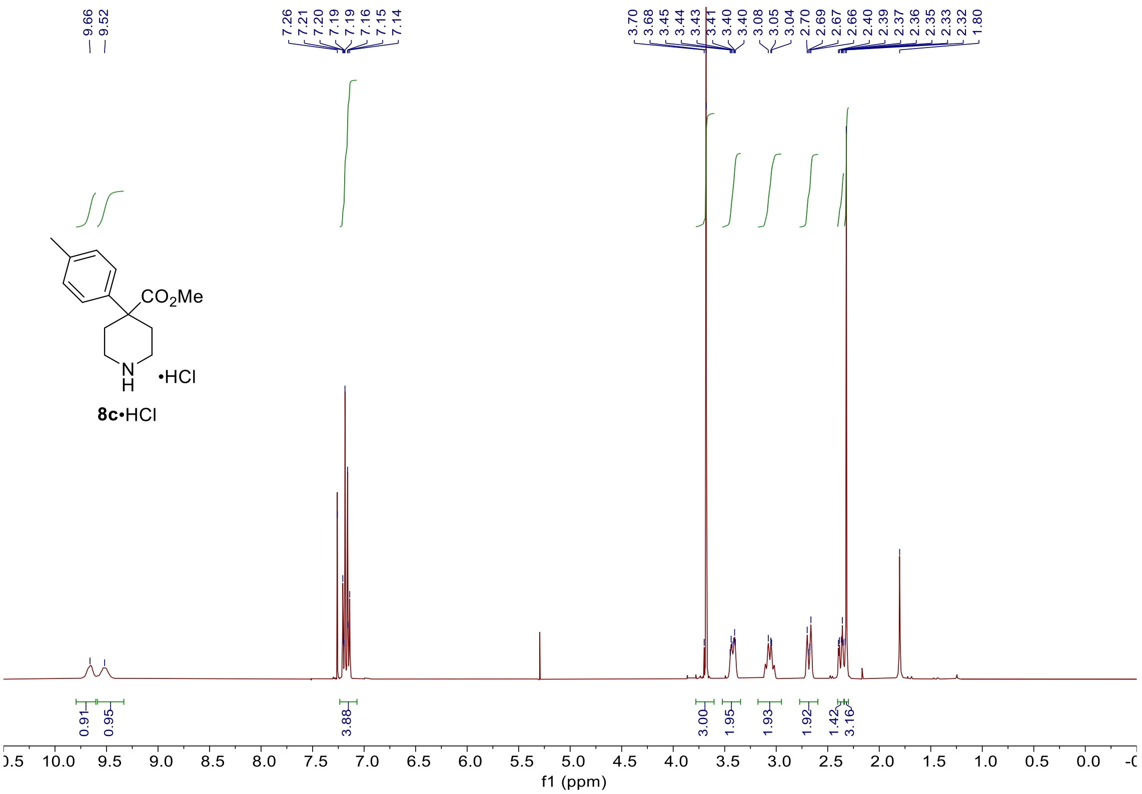

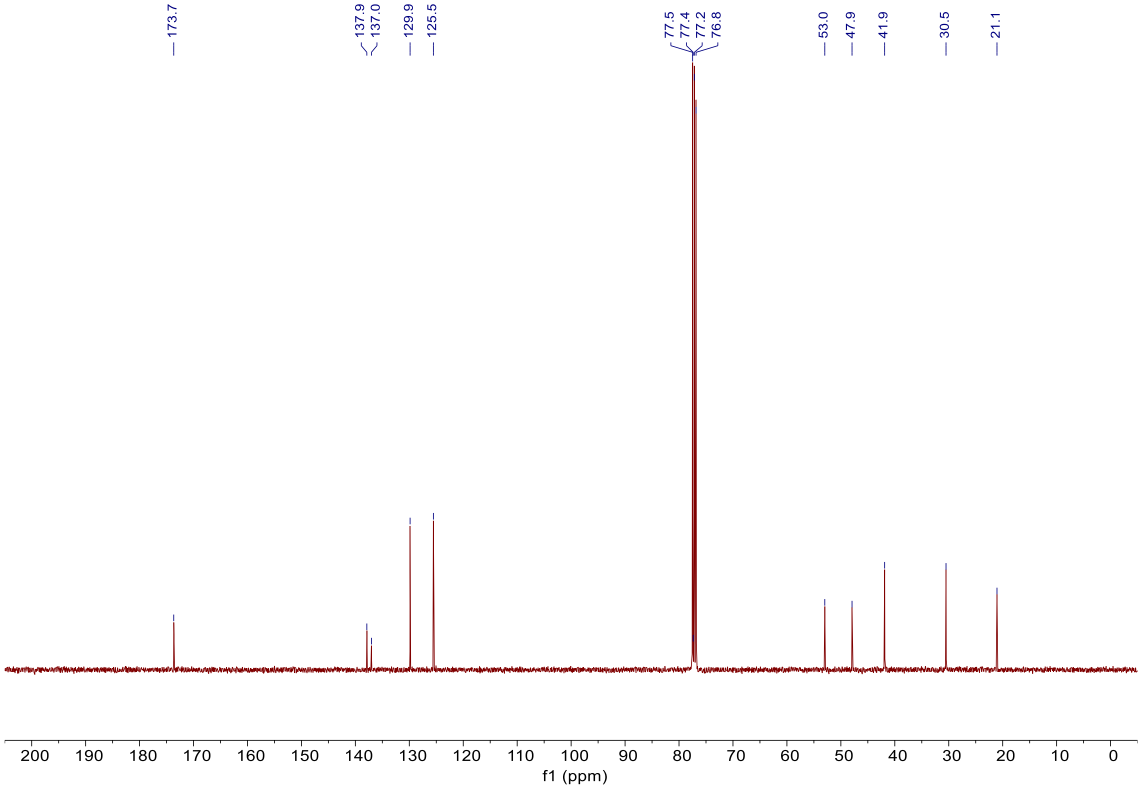

400 MHz ^{1}H NMR spectrum; 100.6 MHz ^{13}C NMR spectrum; $CDCl_3$ of **8d**•HCl

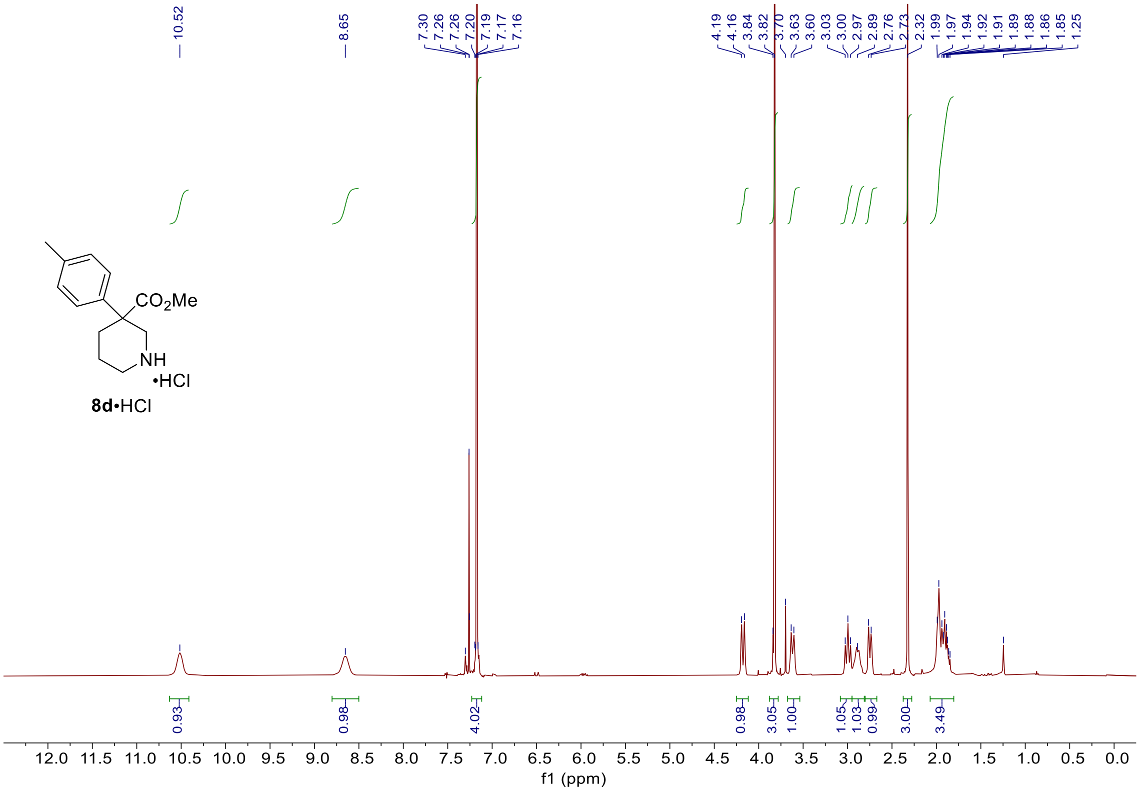

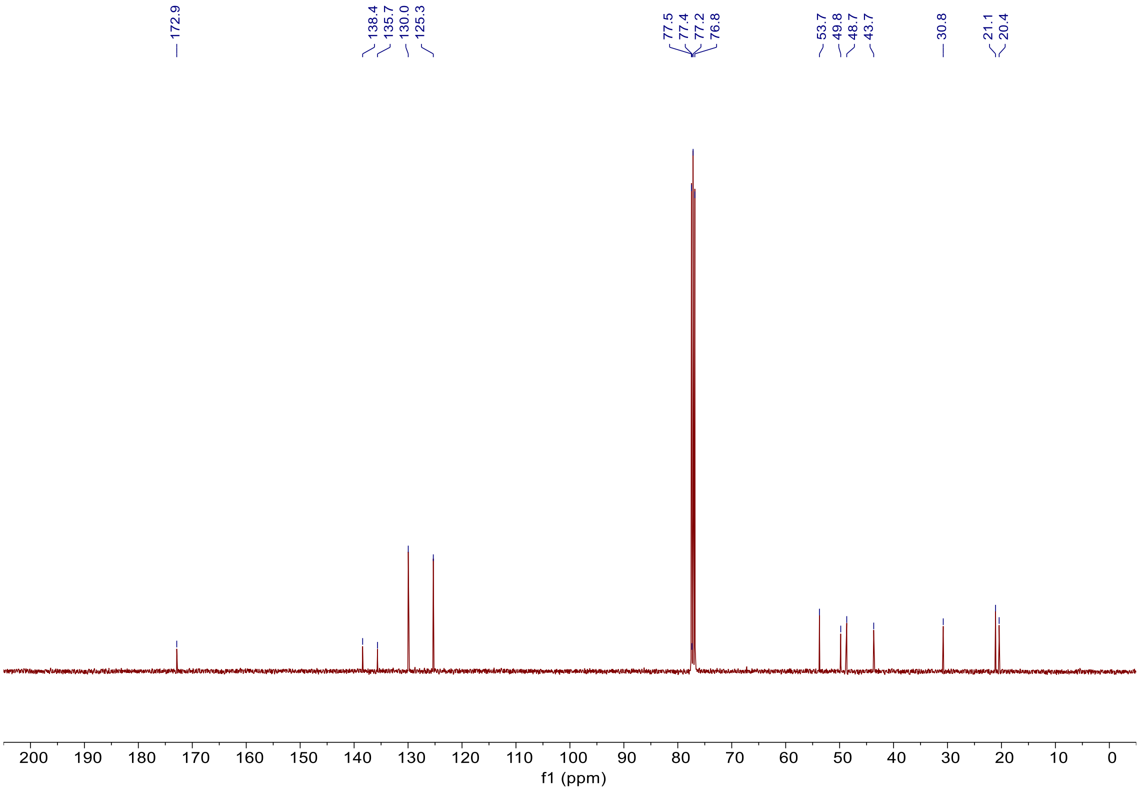

400 MHz ^{1}H NMR spectrum; 100.6 MHz ^{13}C NMR spectrum; $CDCl_3$ of **8e**•HCl

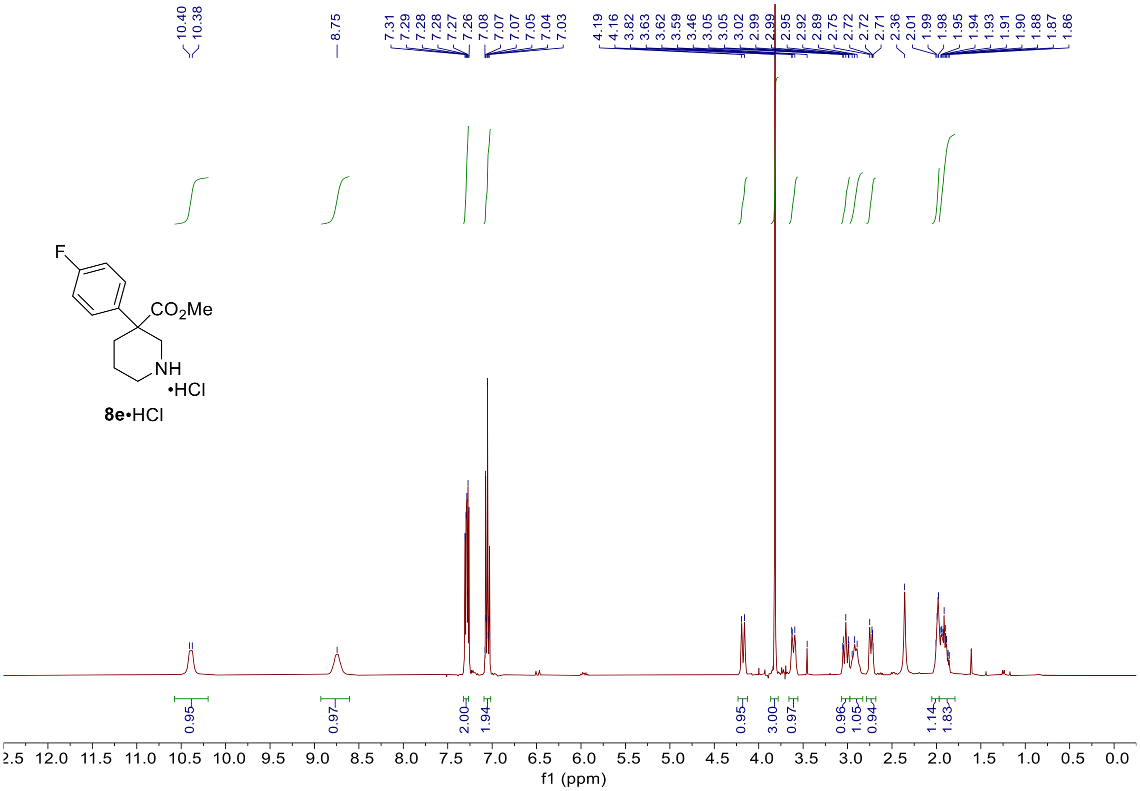

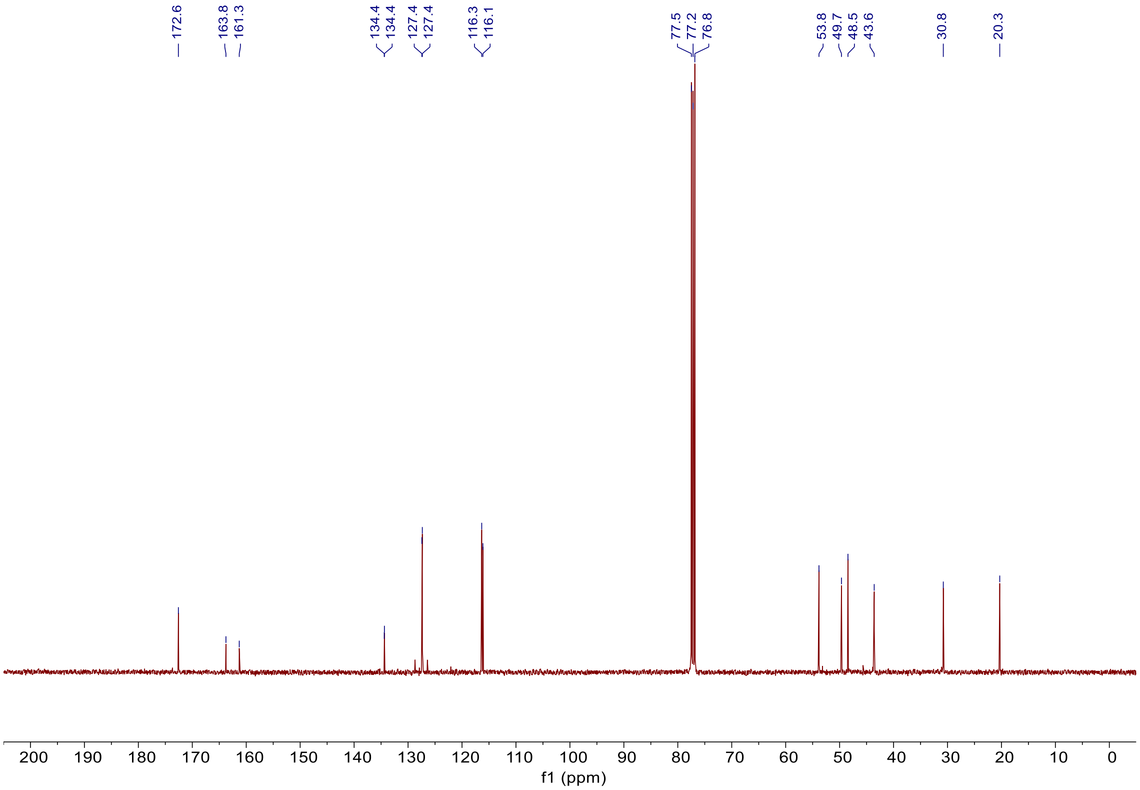

400 MHz ^{1}H NMR spectrum; 100.6 MHz ^{13}C NMR spectrum; $CDCl_3$ of **8f**•HCl

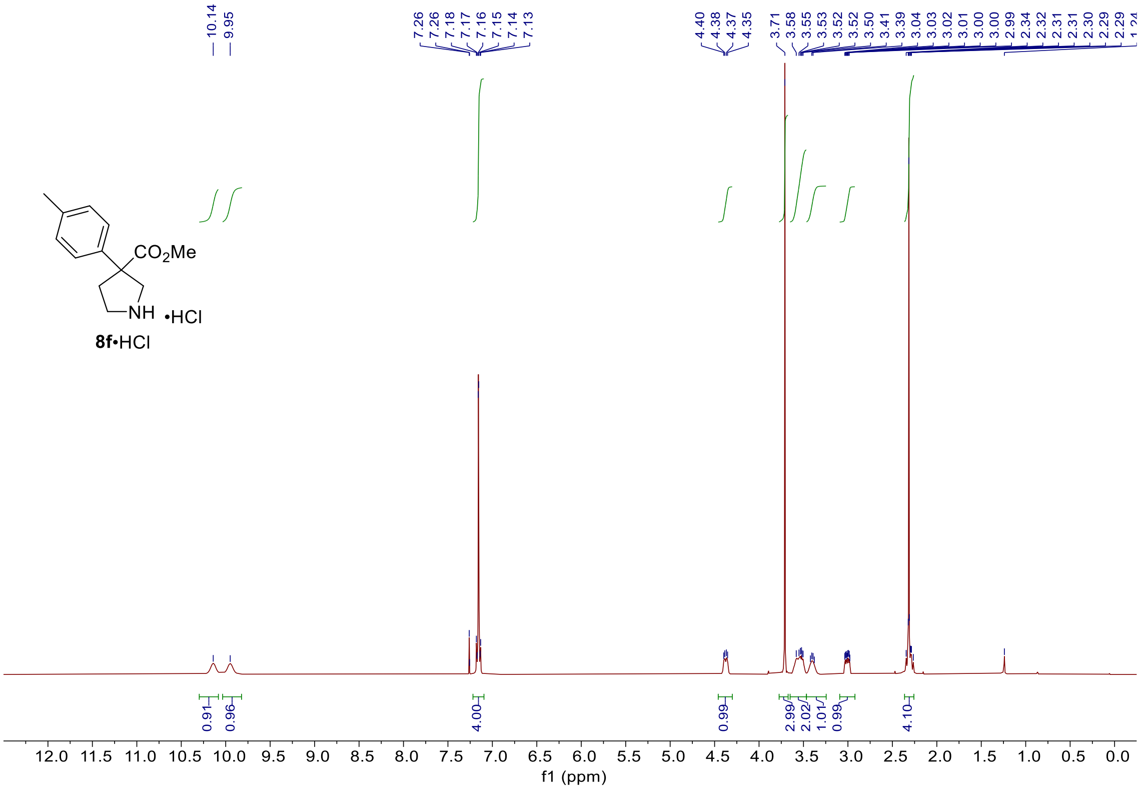

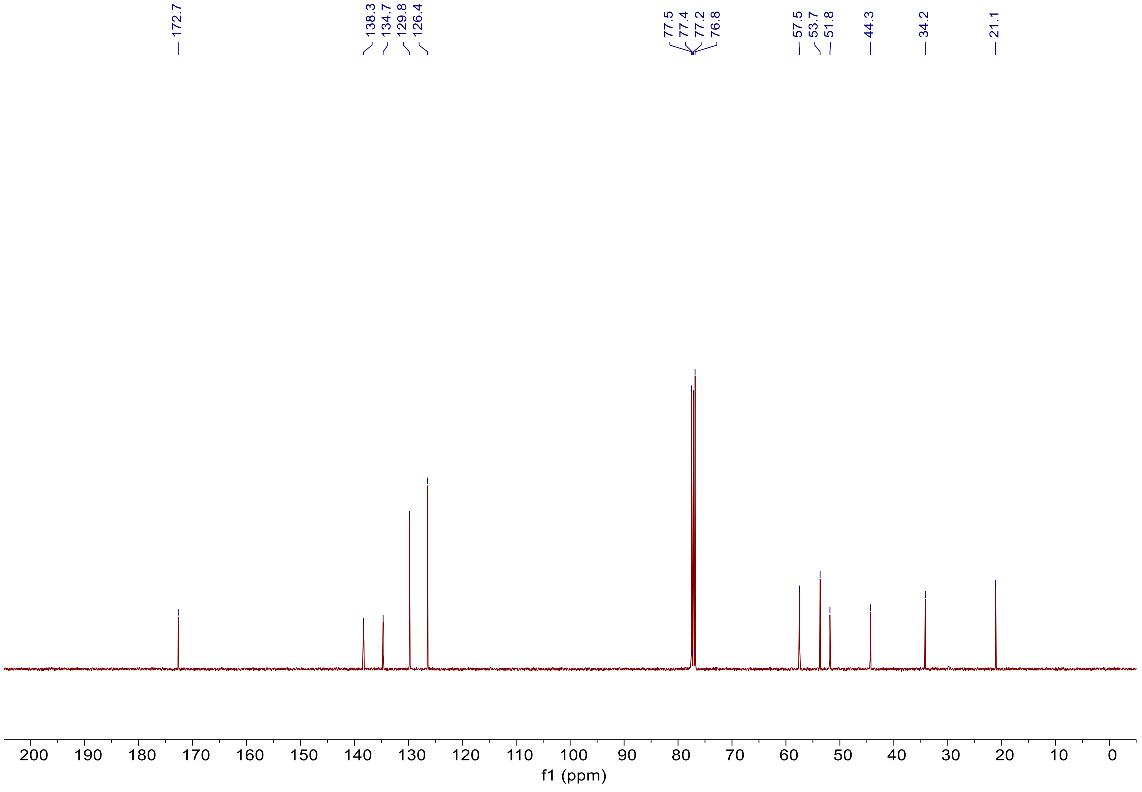

400 MHz ^{1}H NMR spectrum; 100.6 MHz ^{13}C NMR spectrum; MeOD-d_4 of **8g**•HCl

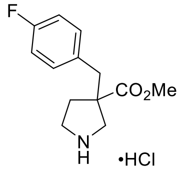

8g•HCl

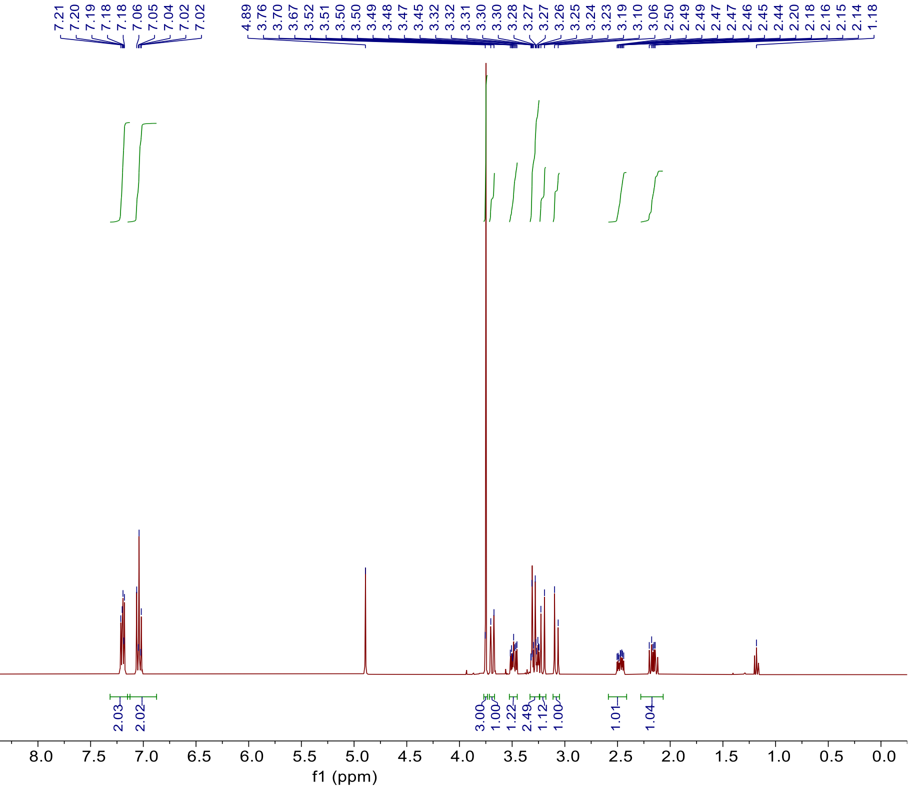

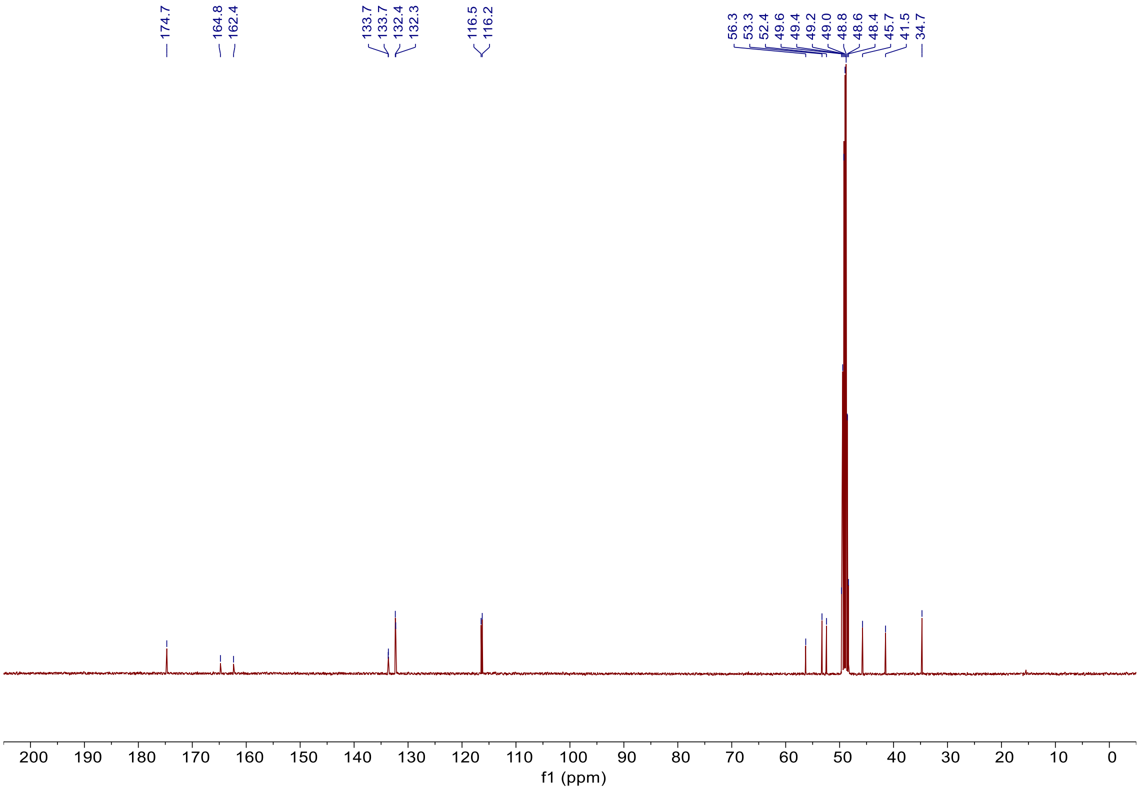

400 MHz ^{1}H NMR spectrum; 100.6 MHz ^{13}C NMR spectrum; MeOD-d_4 of **8h**•HCl

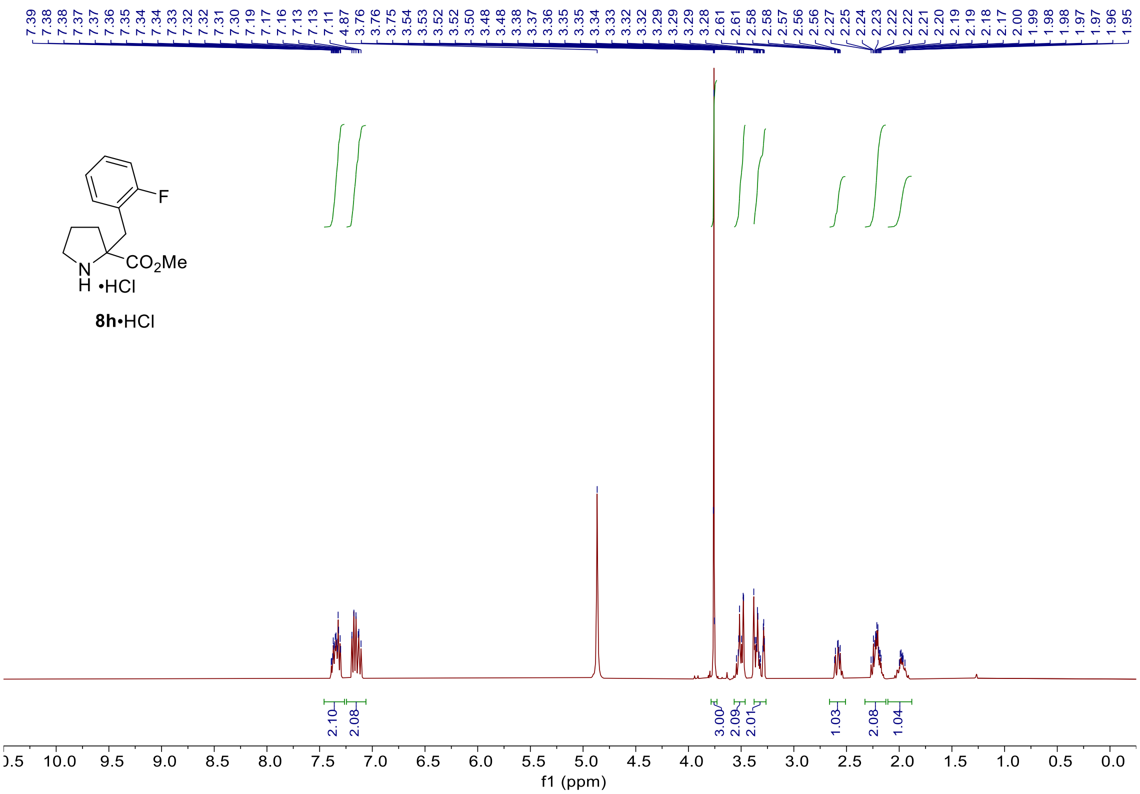

8h•HCl

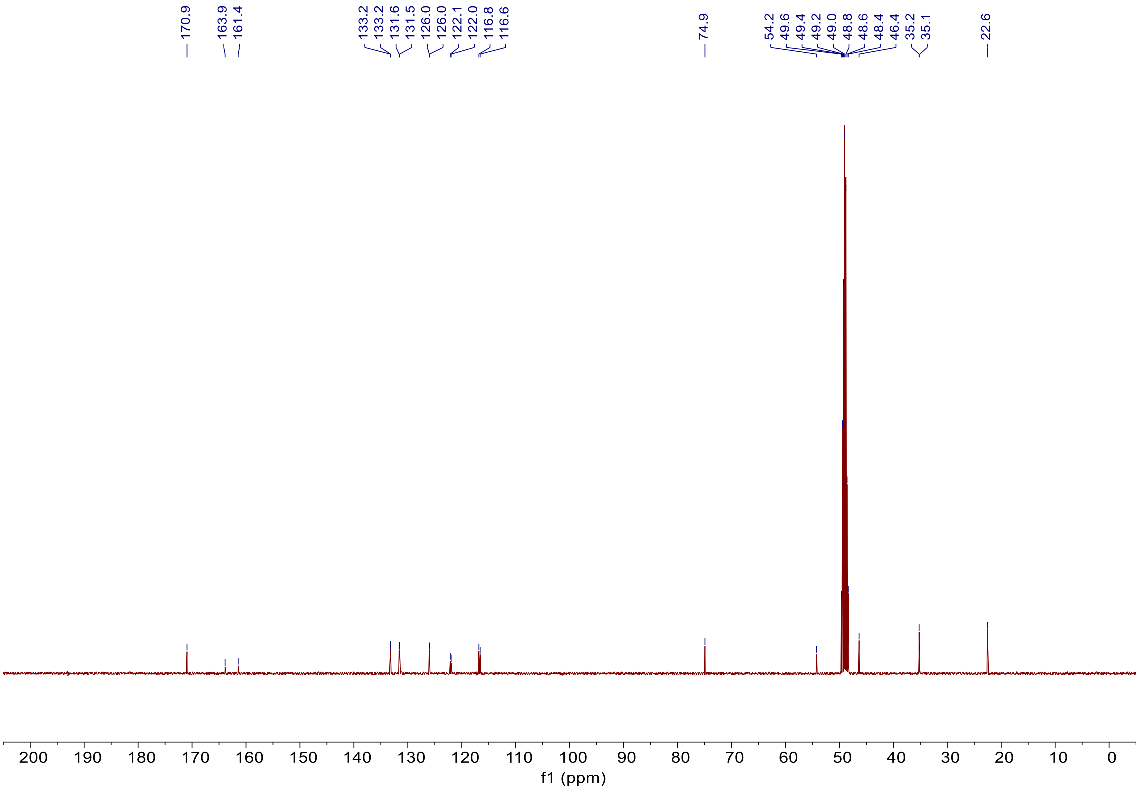

400 MHz ^{1}H NMR spectrum; 100.6 MHz ^{13}Ci NMR spectrum; MeOD-d_4 of **8i**•HCl

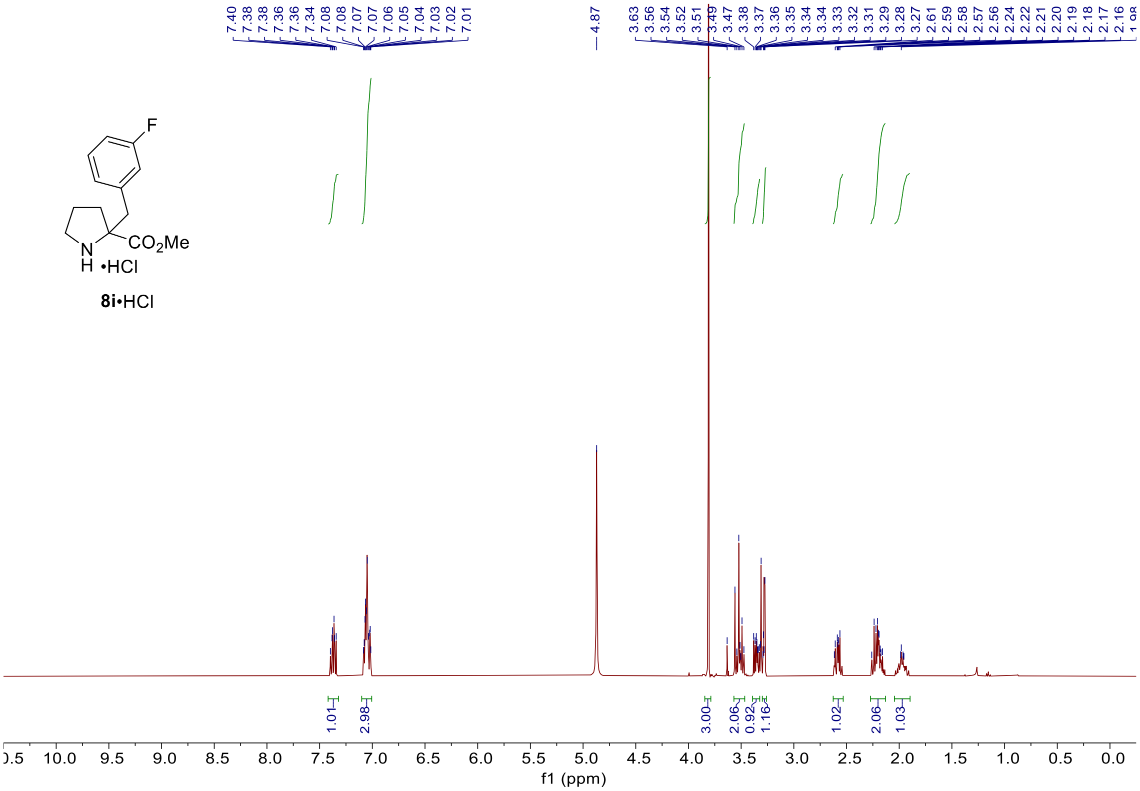

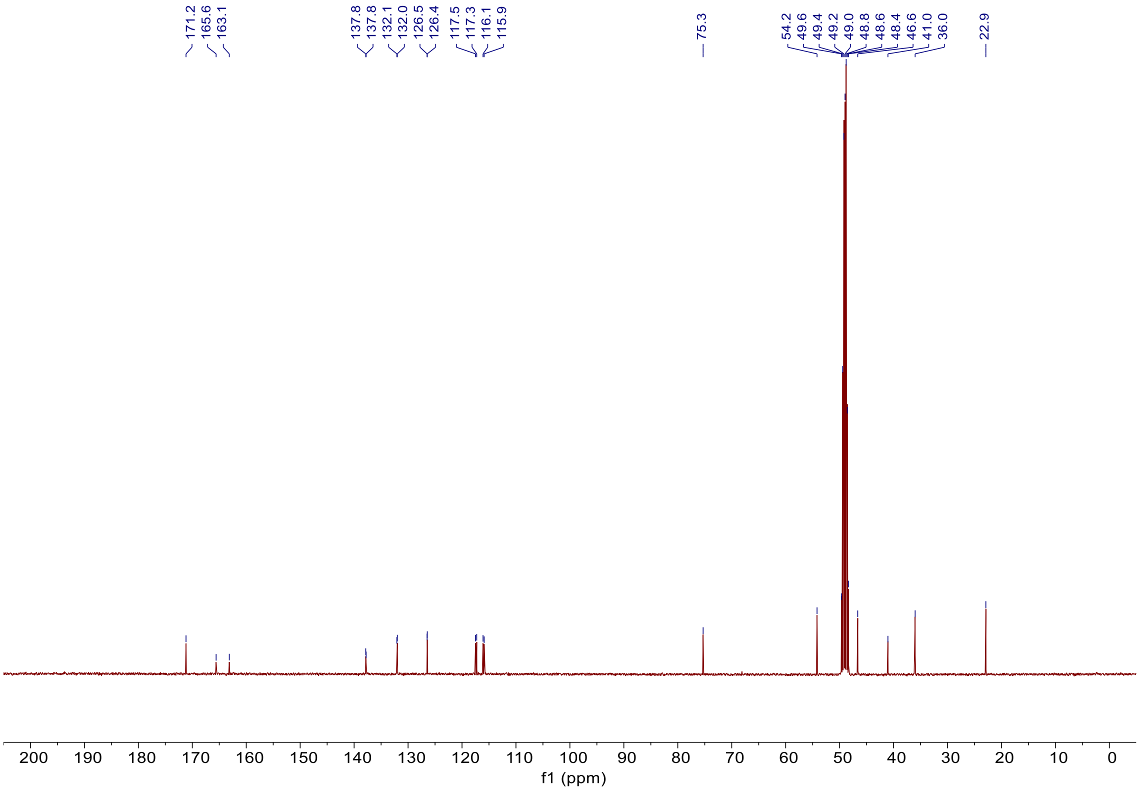

400 MHz ^{1}H NMR spectrum; 100.6 MHz ^{13}C NMR spectrum; MeOD-d_4 of **8j**•HCl

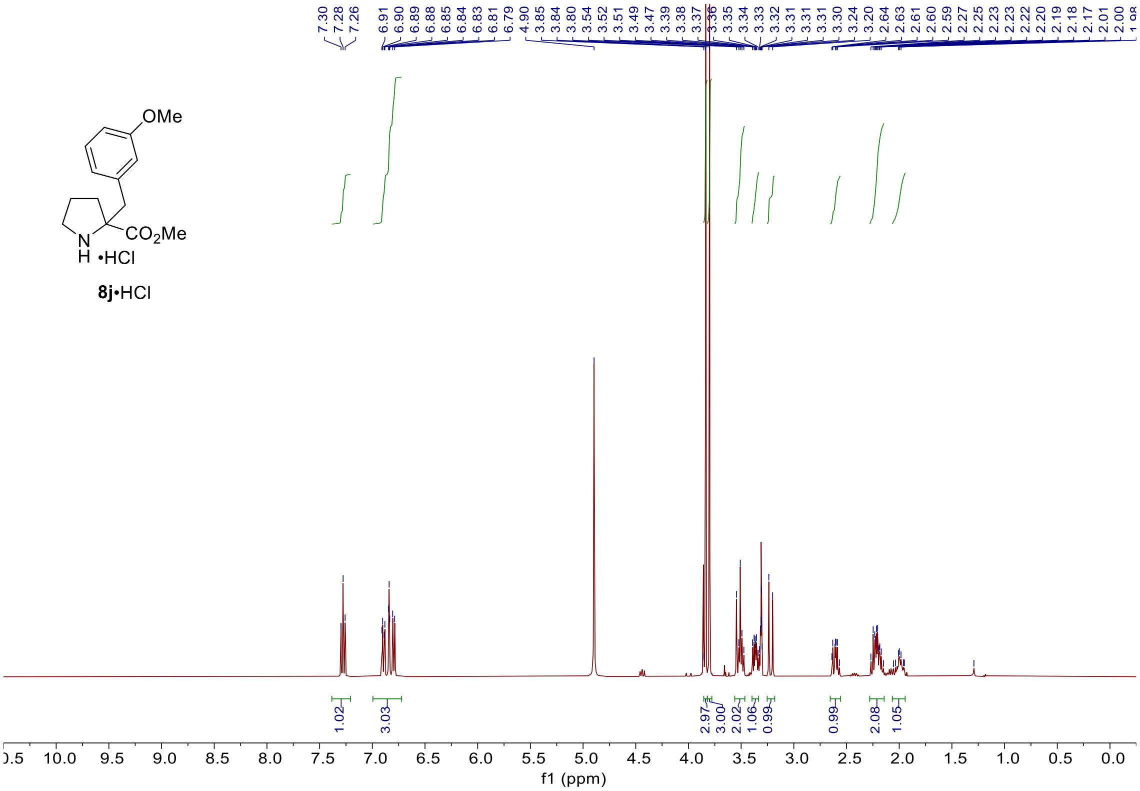

8j•HCl

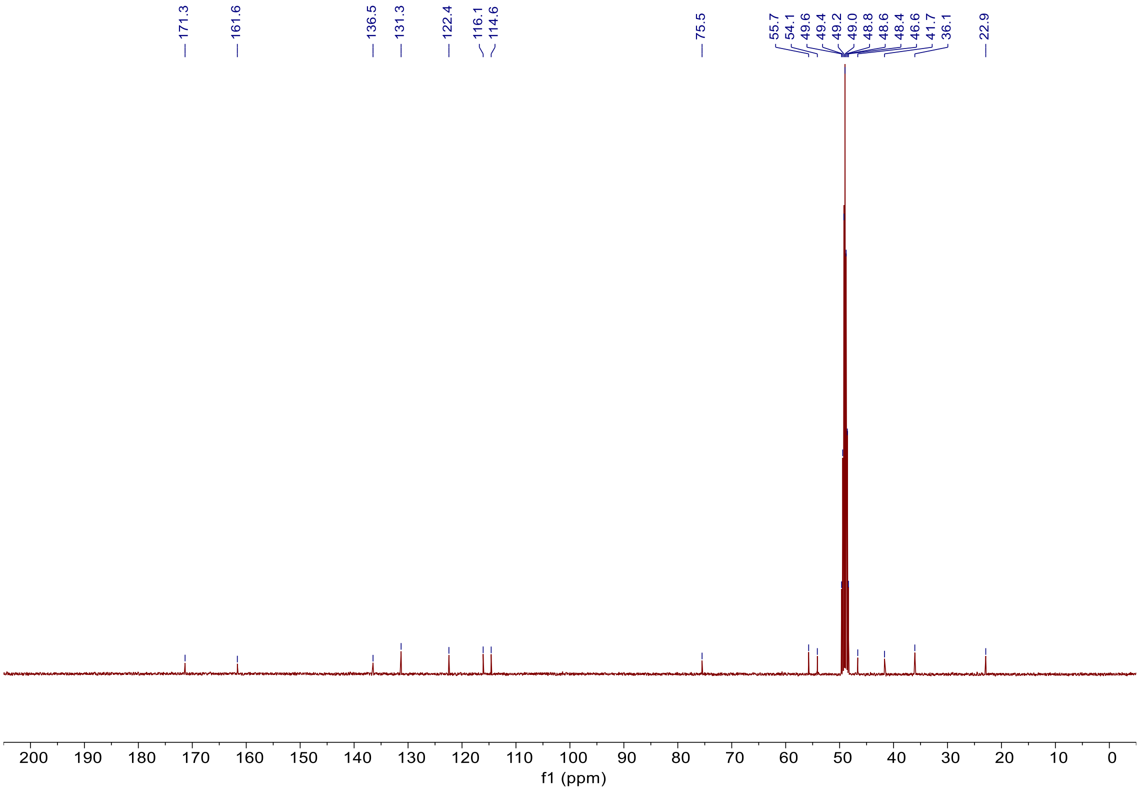

400 MHz ^{1}H NMR spectrum; 100.6 MHz ^{13}C NMR spectrum; $CDCl_3$ of **8k**•HCl

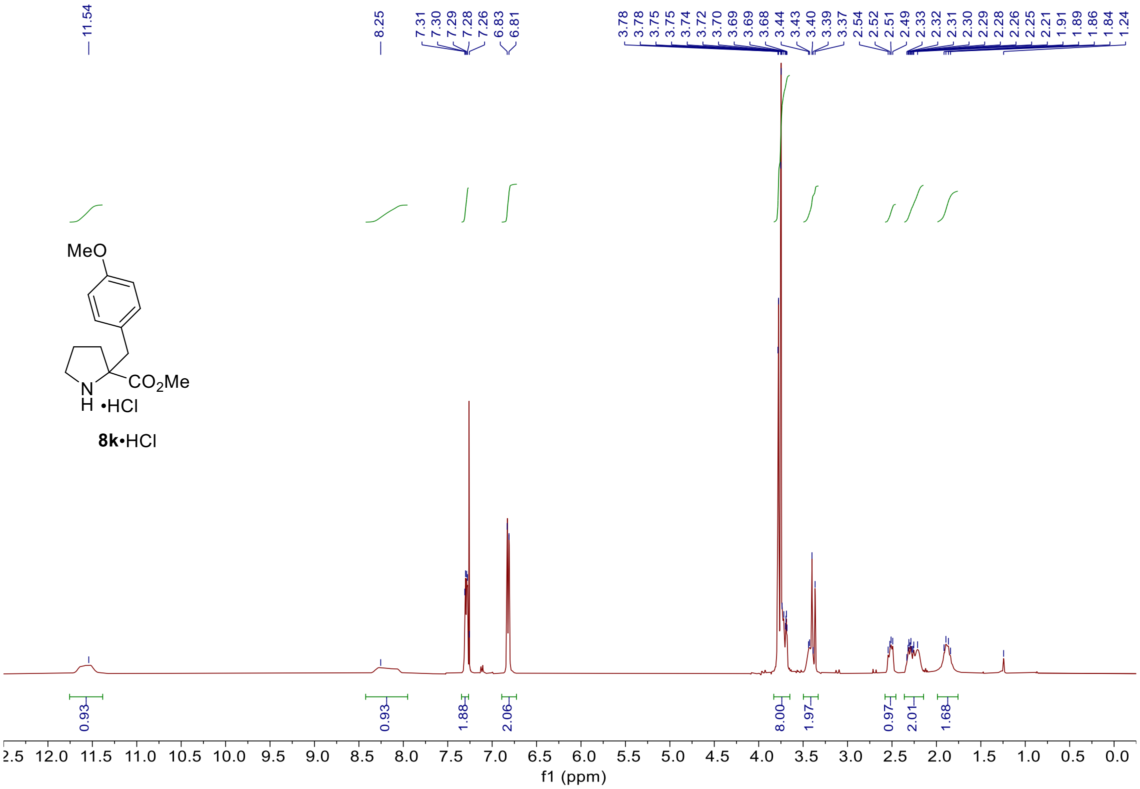

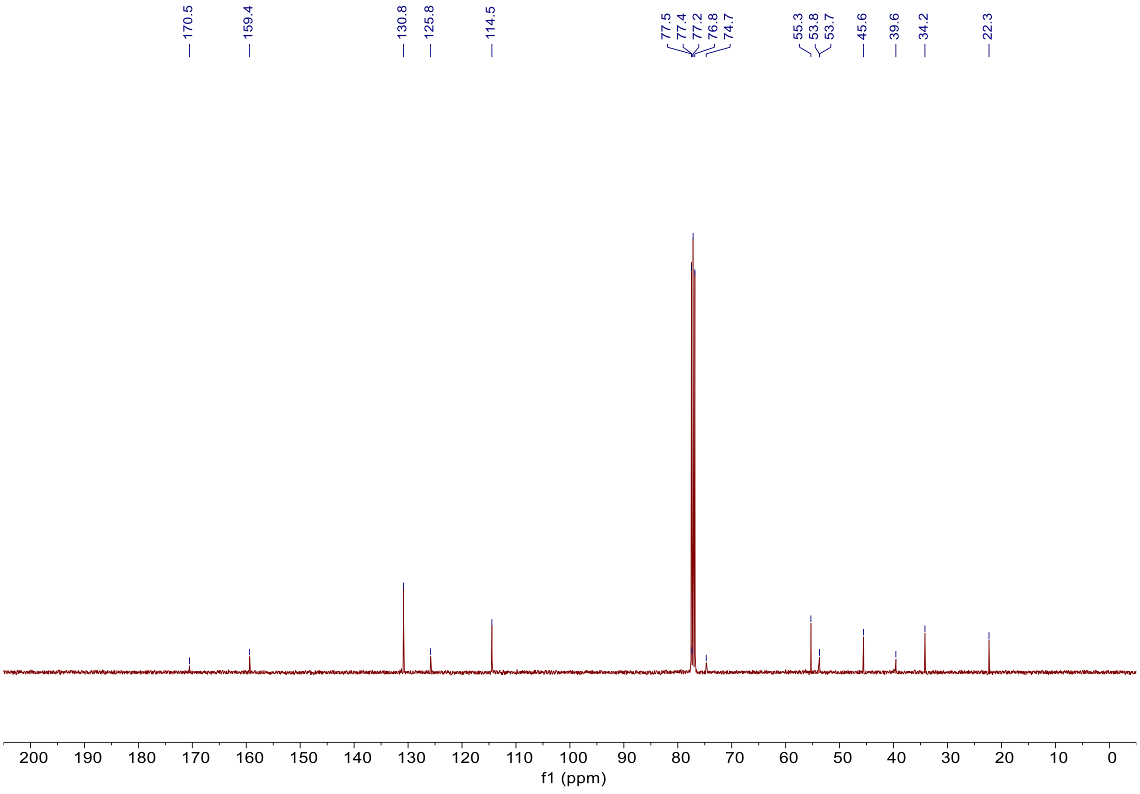

400 MHz ^{1}H NMR spectrum; 100.6 MHz ^{13}C NMR spectrum; MeOD-d_4 of **8l**•2HCl

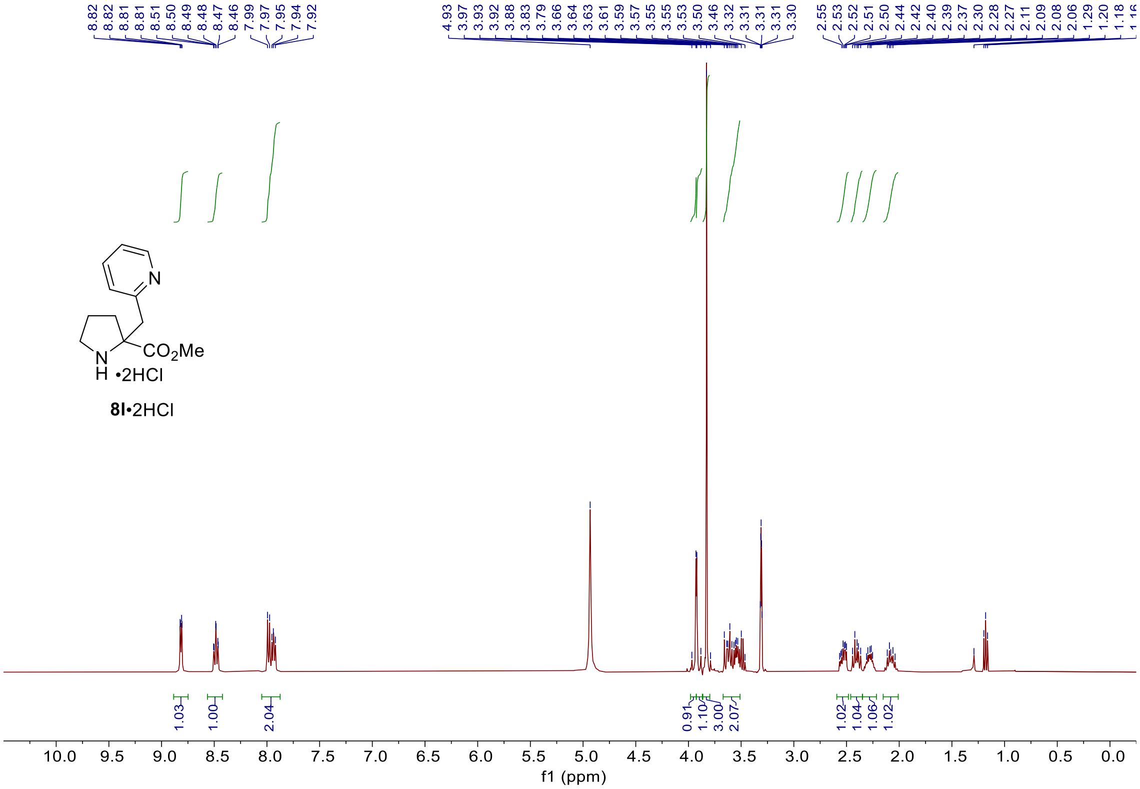

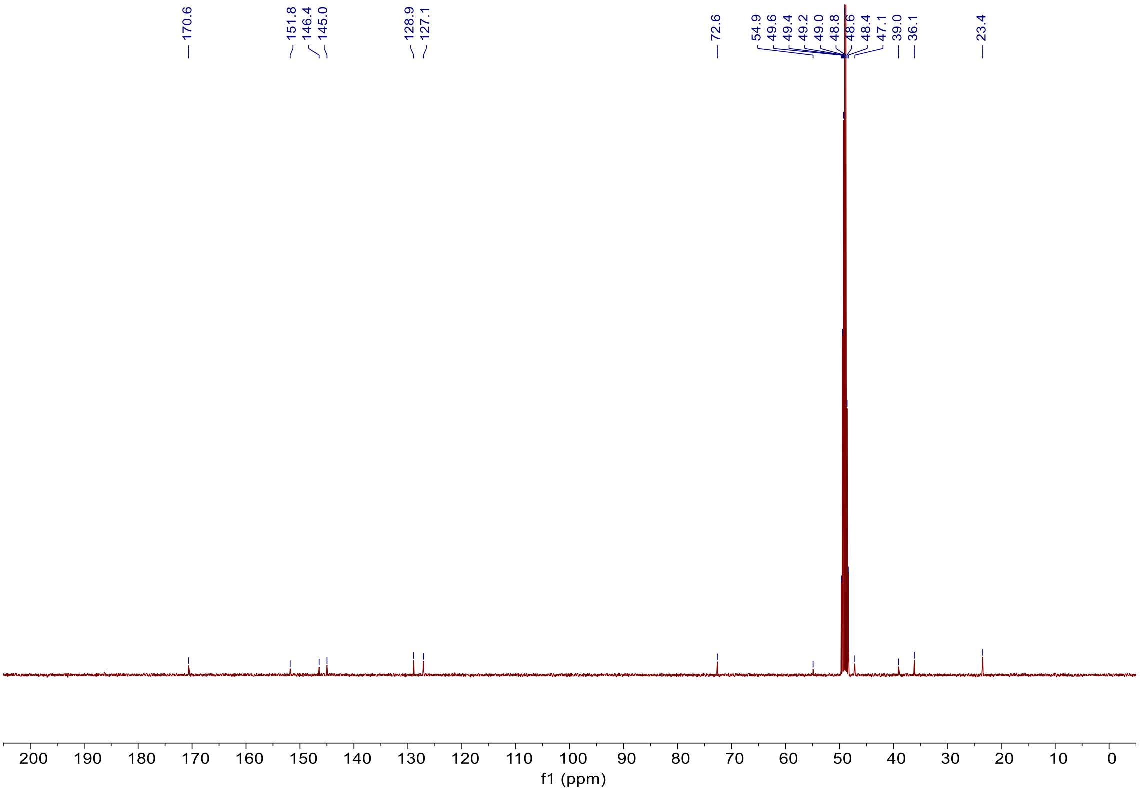

400 MHz ^{1}H NMR spectrum; 100.6 MHz ^{13}C NMR spectrum; $CDCl_3$ of **8m**

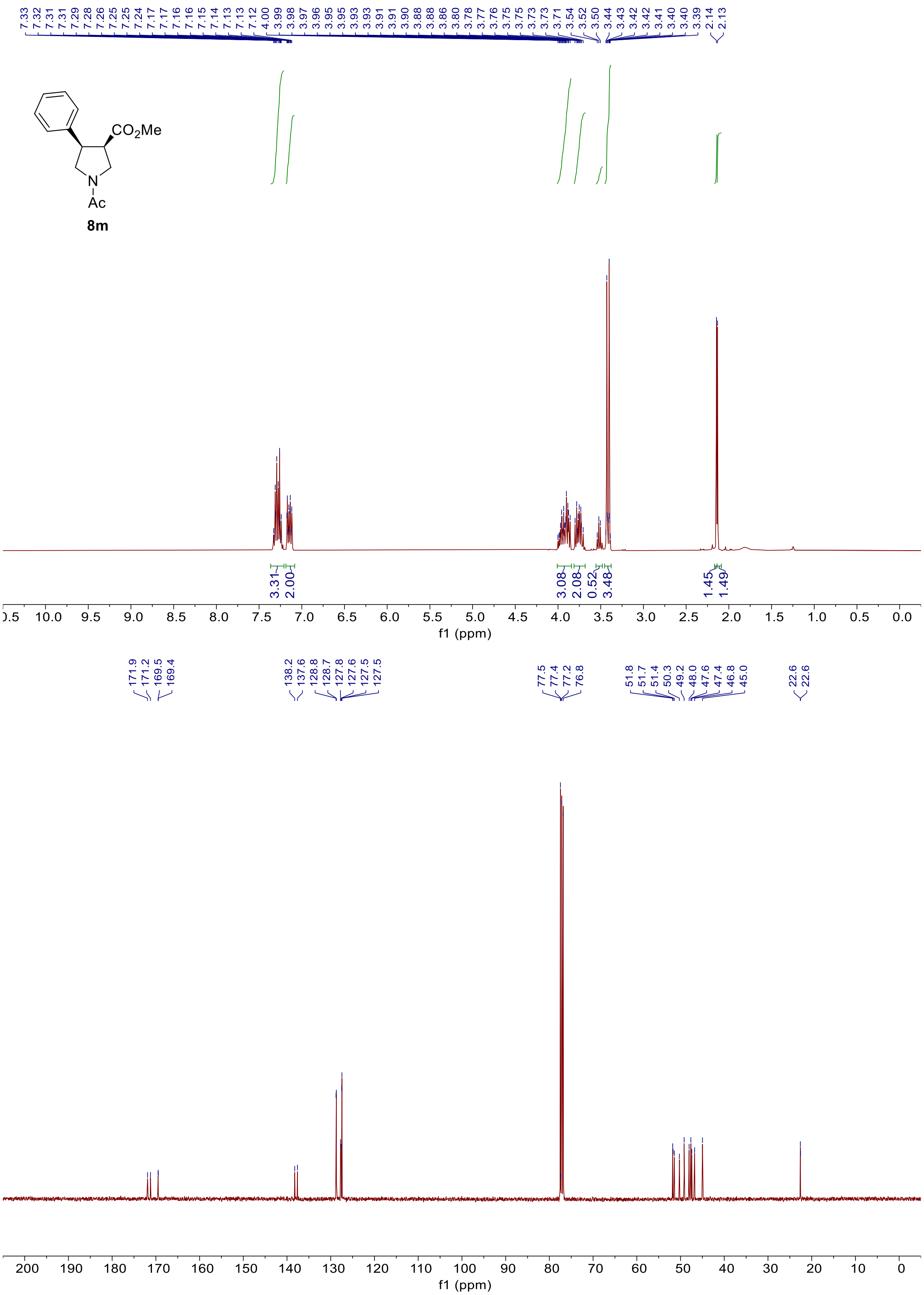

400 MHz ^{1}H NMR spectrum; 100.6 MHz ^{13}C NMR spectrum; $CDCl_3$ of **8n**

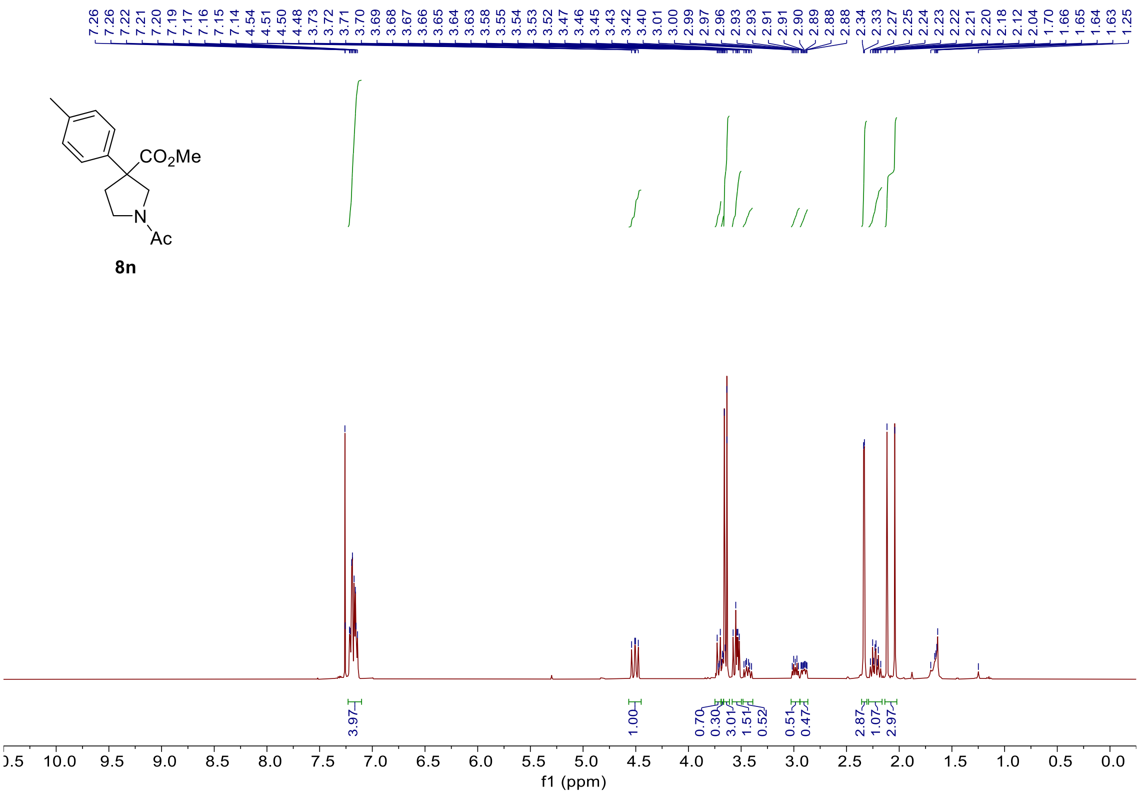

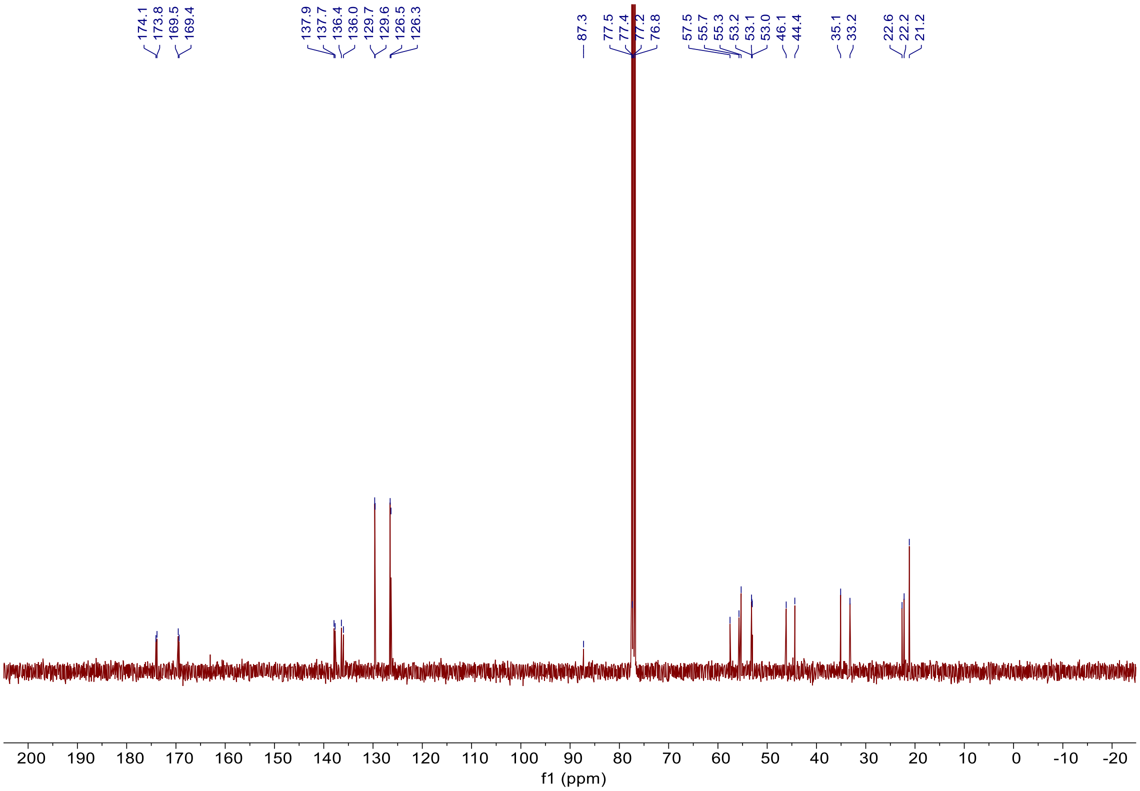

400 MHz ^{1}H NMR spectrum; 100.6 MHz ^{13}C NMR spectrum; $CDCl_3$ of **8o**

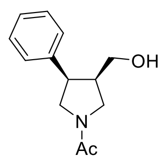

8o

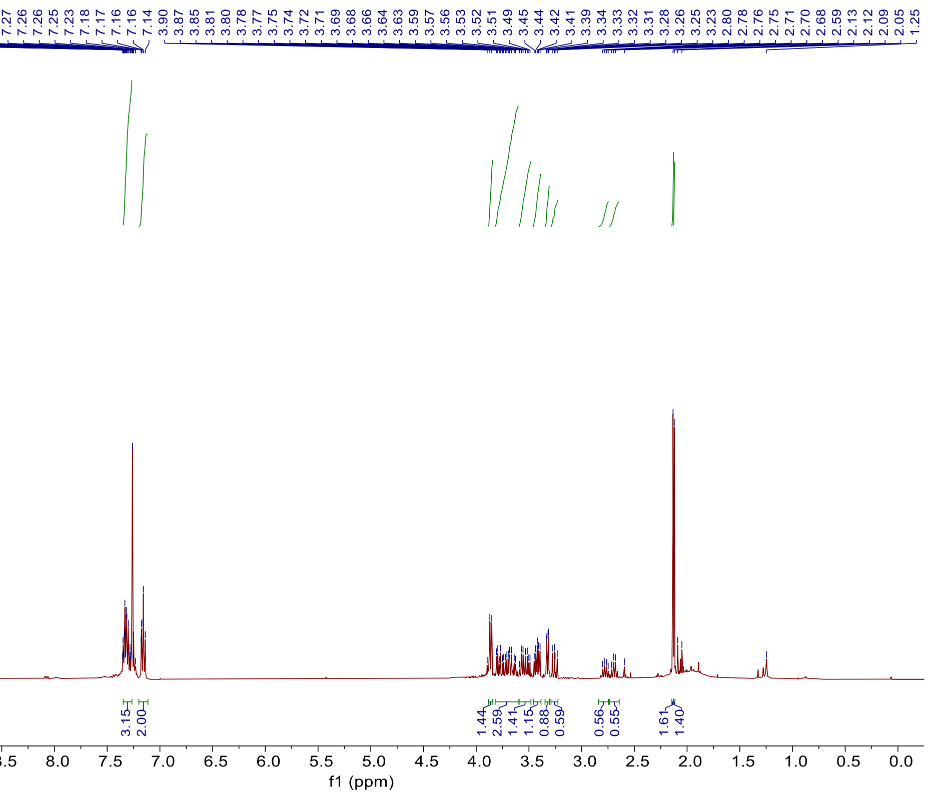

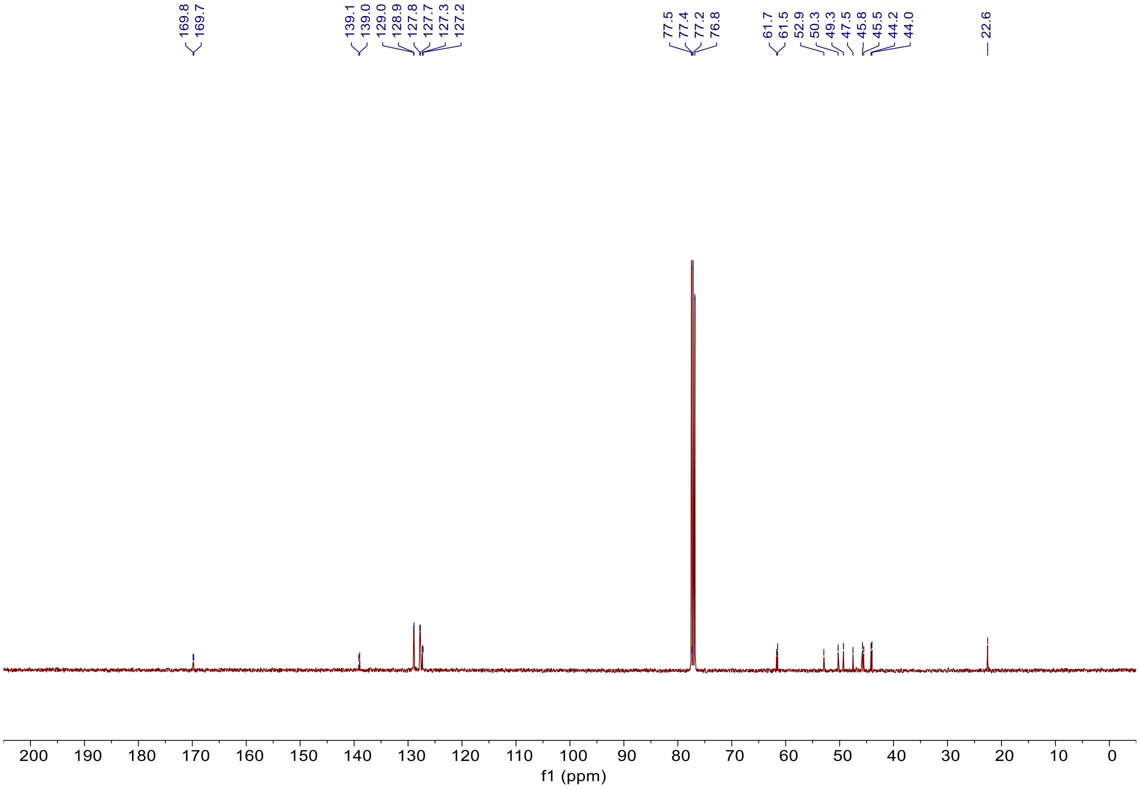

400 MHz ^{1}H NMR spectrum; 100.6 MHz ^{13}C NMR spectrum; $CDCl_3$ of **S10**

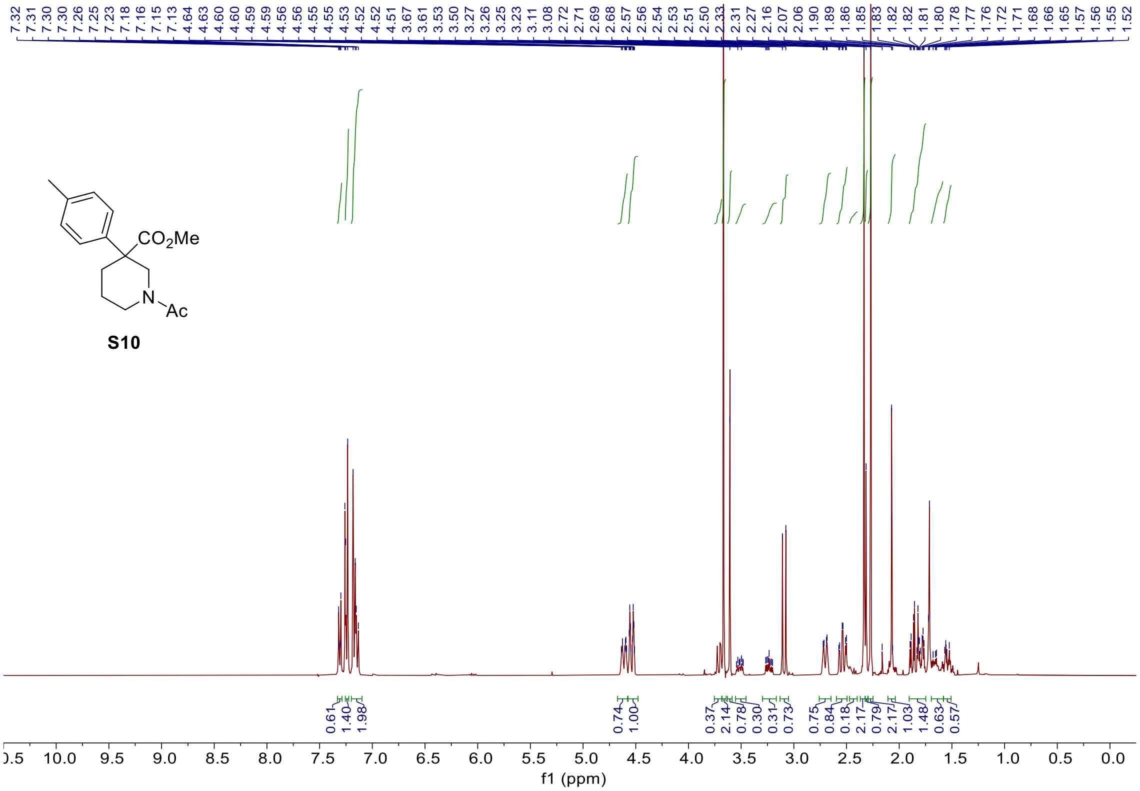

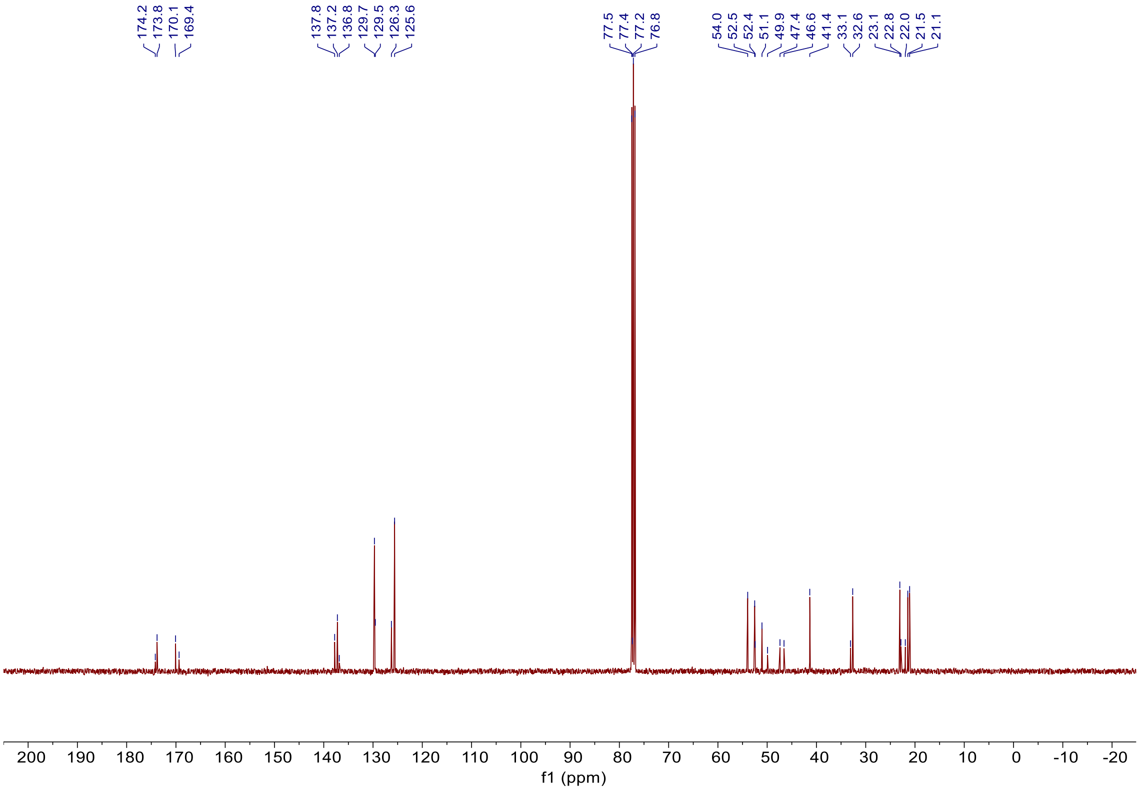

400 MHz ^{1}H NMR spectrum; 100.6 MHz ^{13}C NMR spectrum; $CDCl_3$ of **8p**

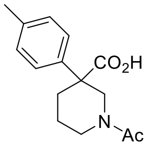

8p

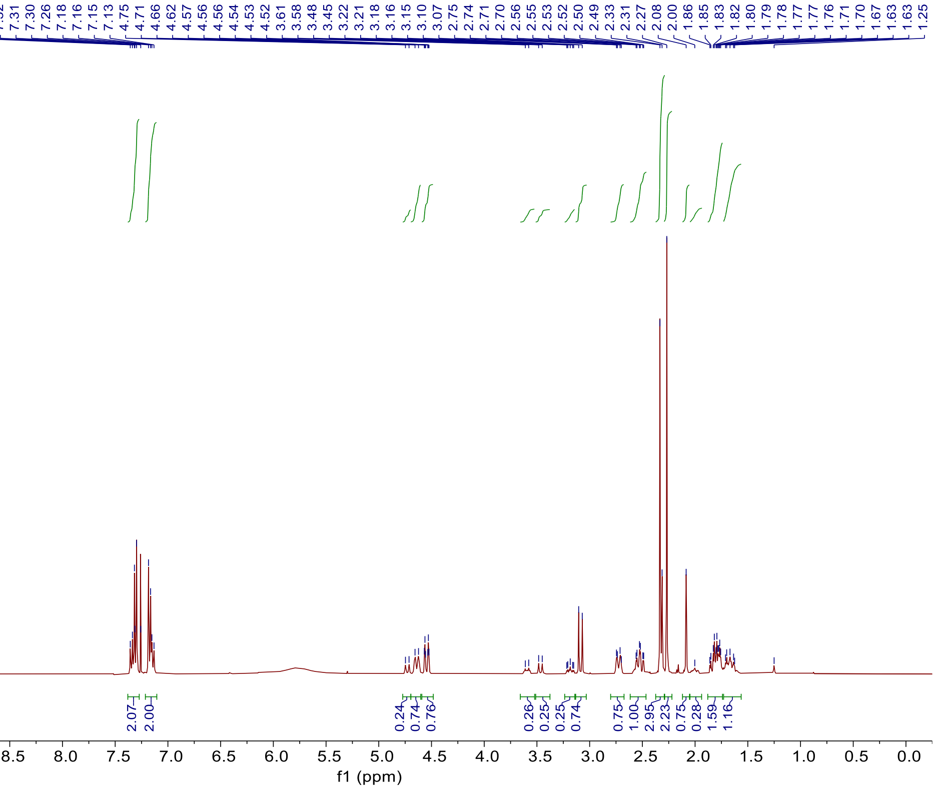

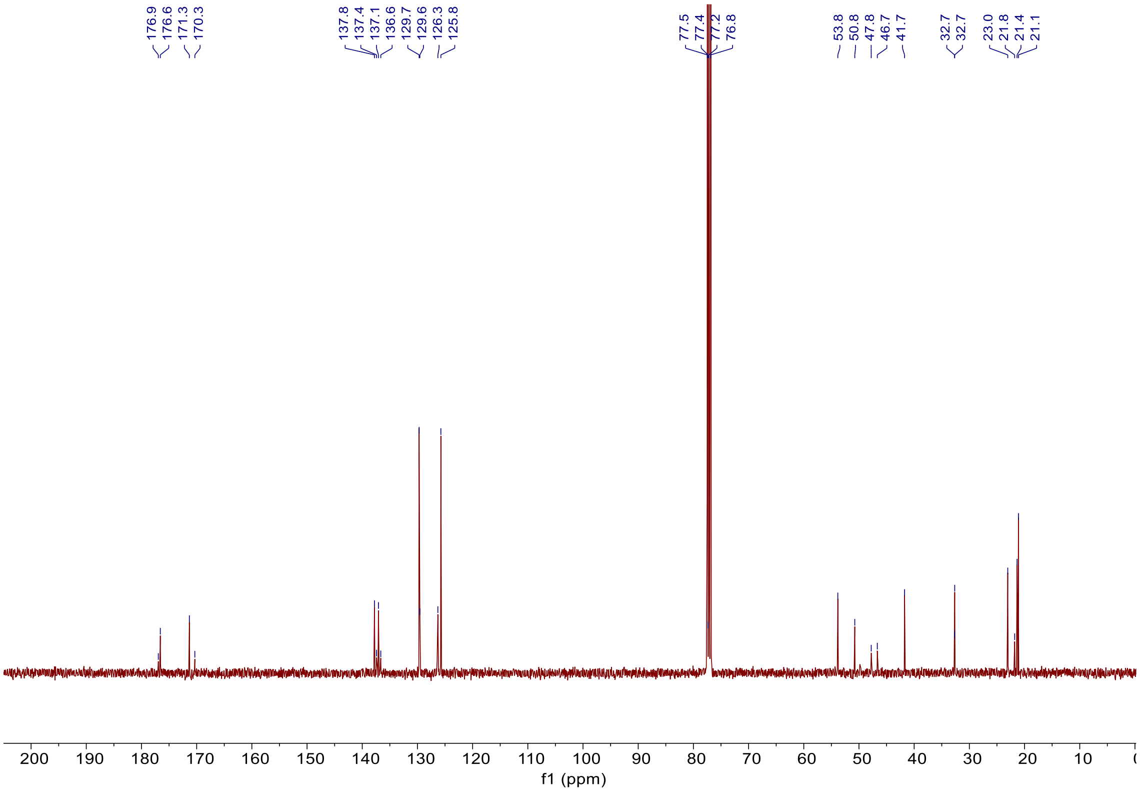

400 MHz ^{1}H NMR spectrum; 100.6 MHz ^{13}C NMR spectrum; $CDCl_3$ of **8q**

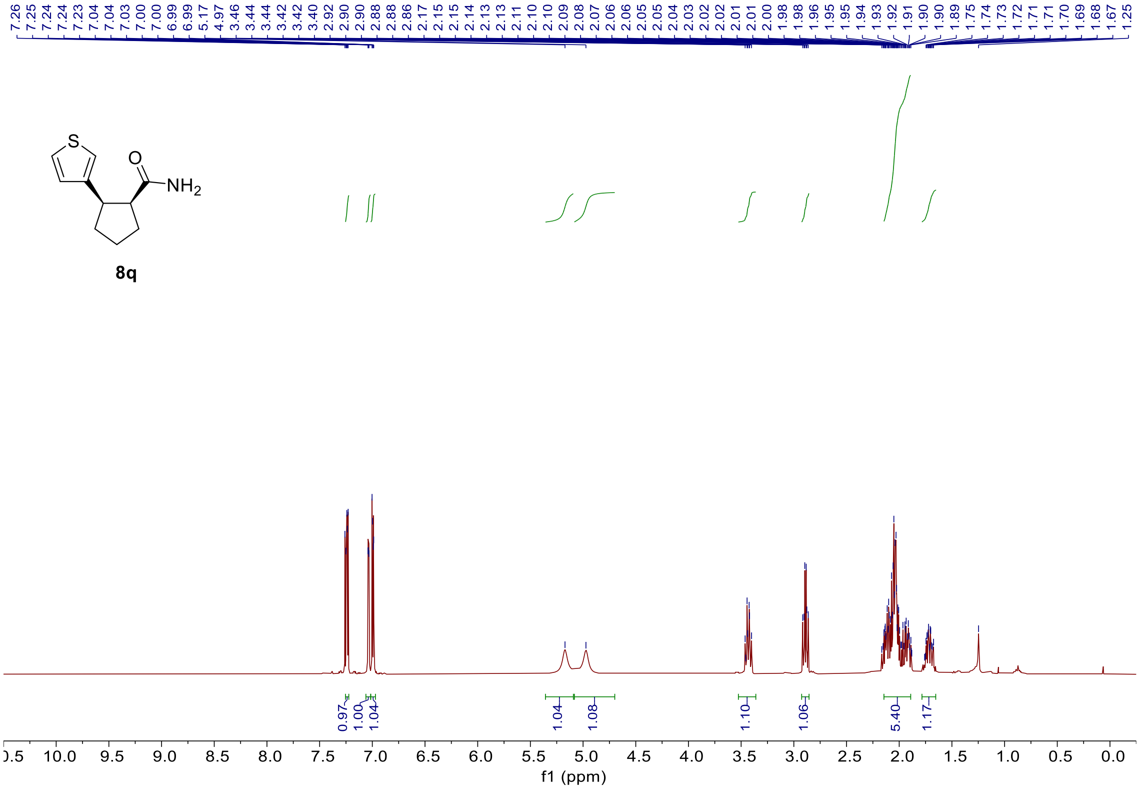

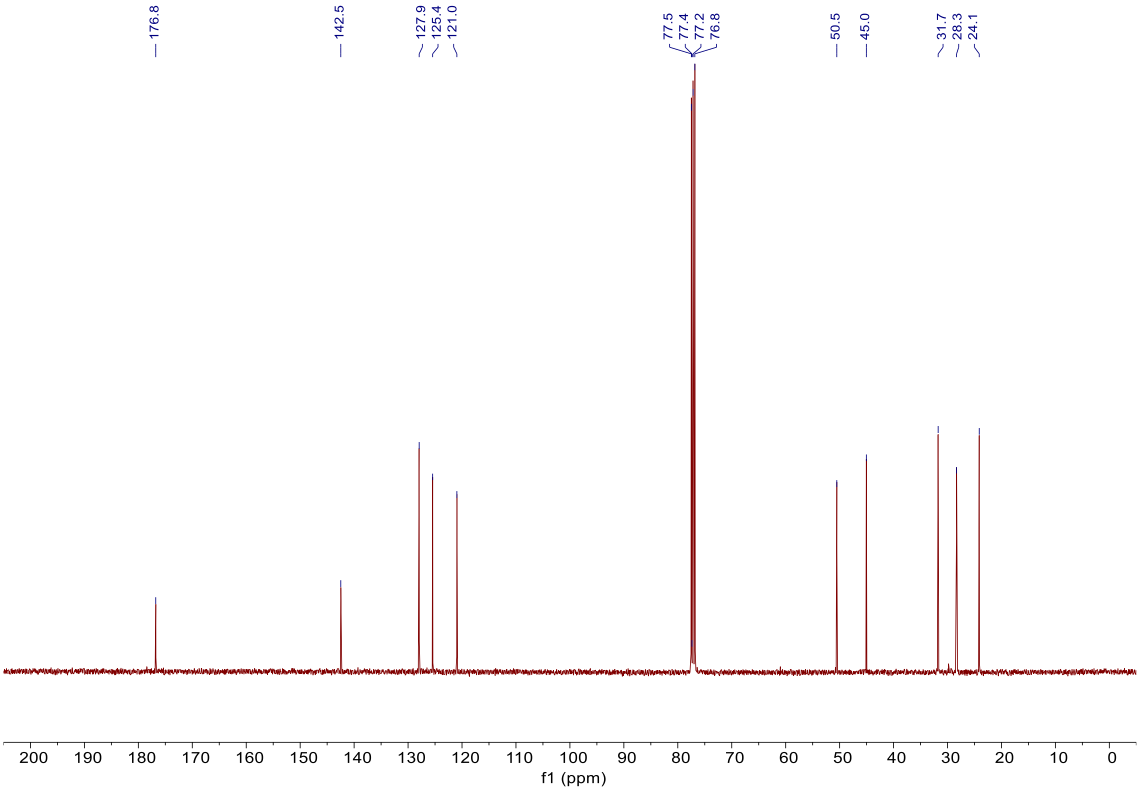

400 MHz ^{1}H NMR spectrum; 100.6 MHz ^{13}C NMR spectrum; $CDCl_3$ of **8r**

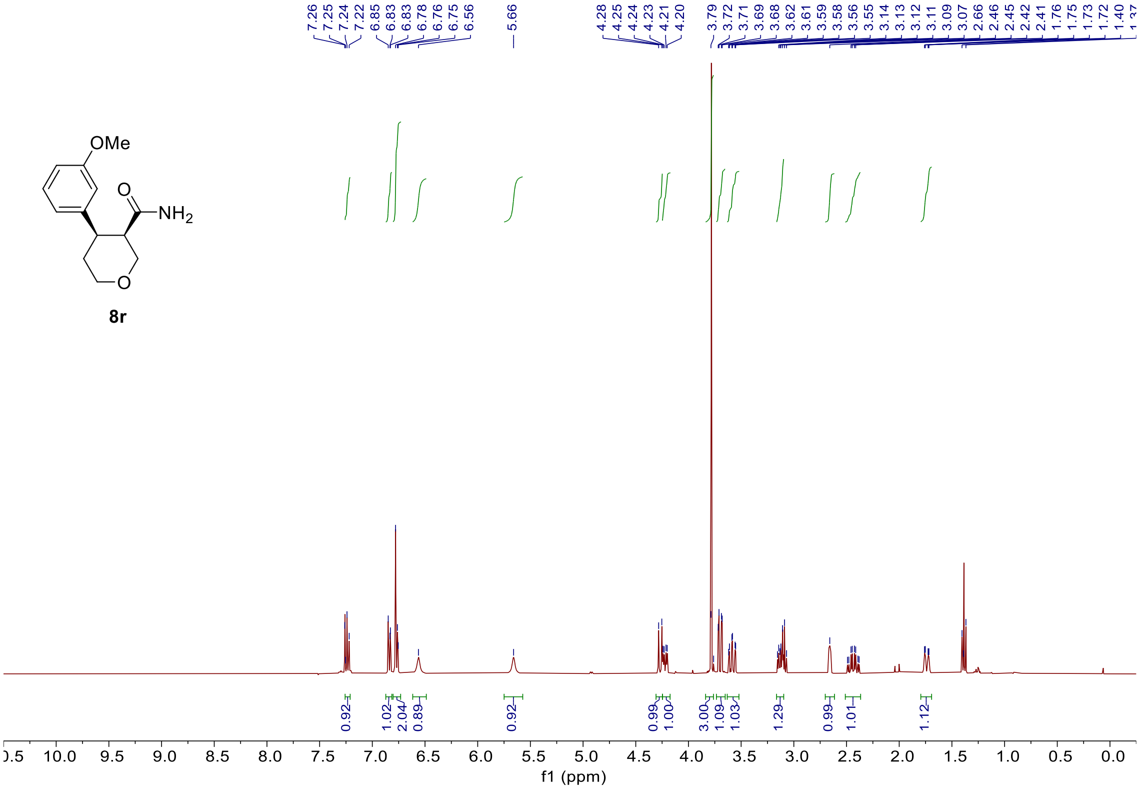

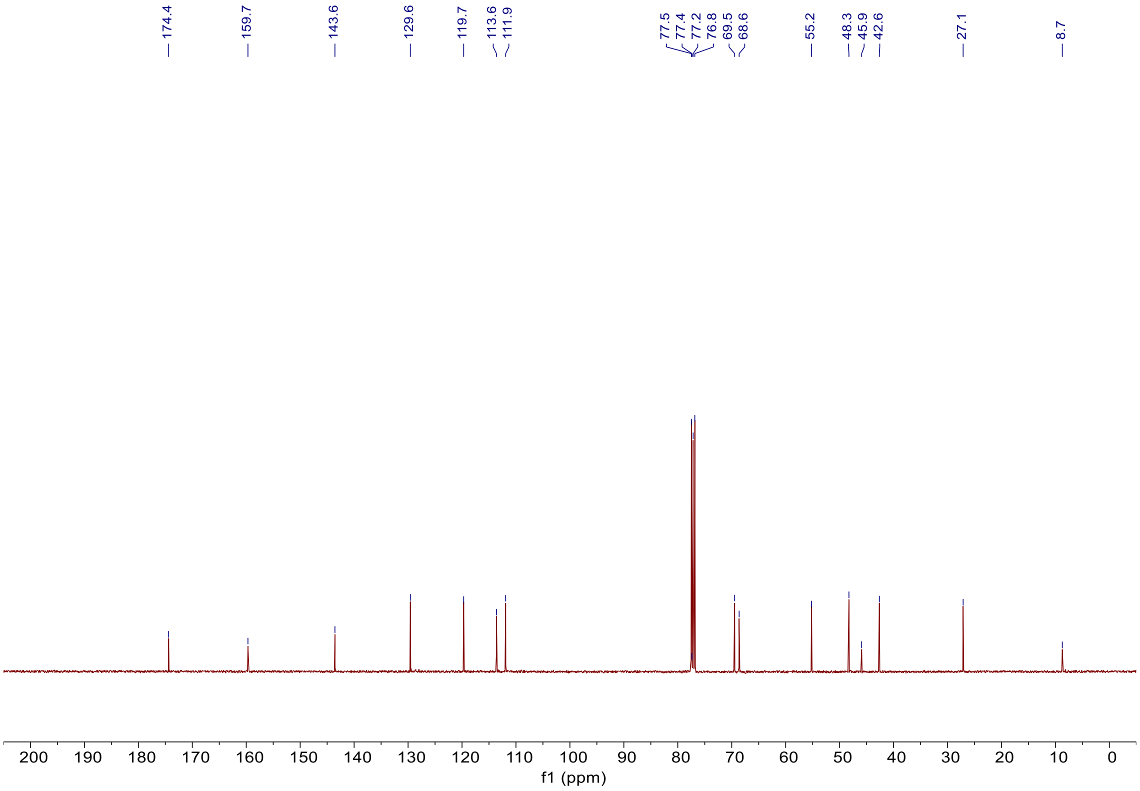

400 MHz ^{1}H NMR spectrum; 100.6 MHz ^{13}C NMR spectrum; $CDCl_3$ of **8s**

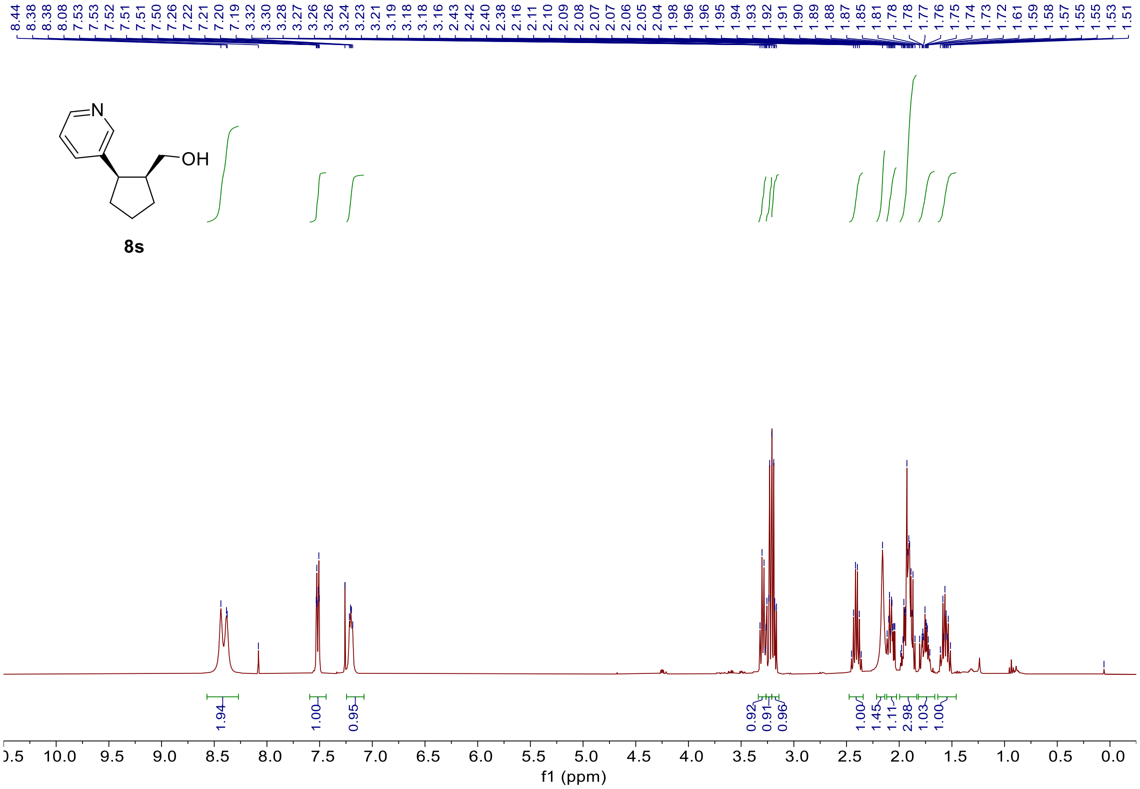

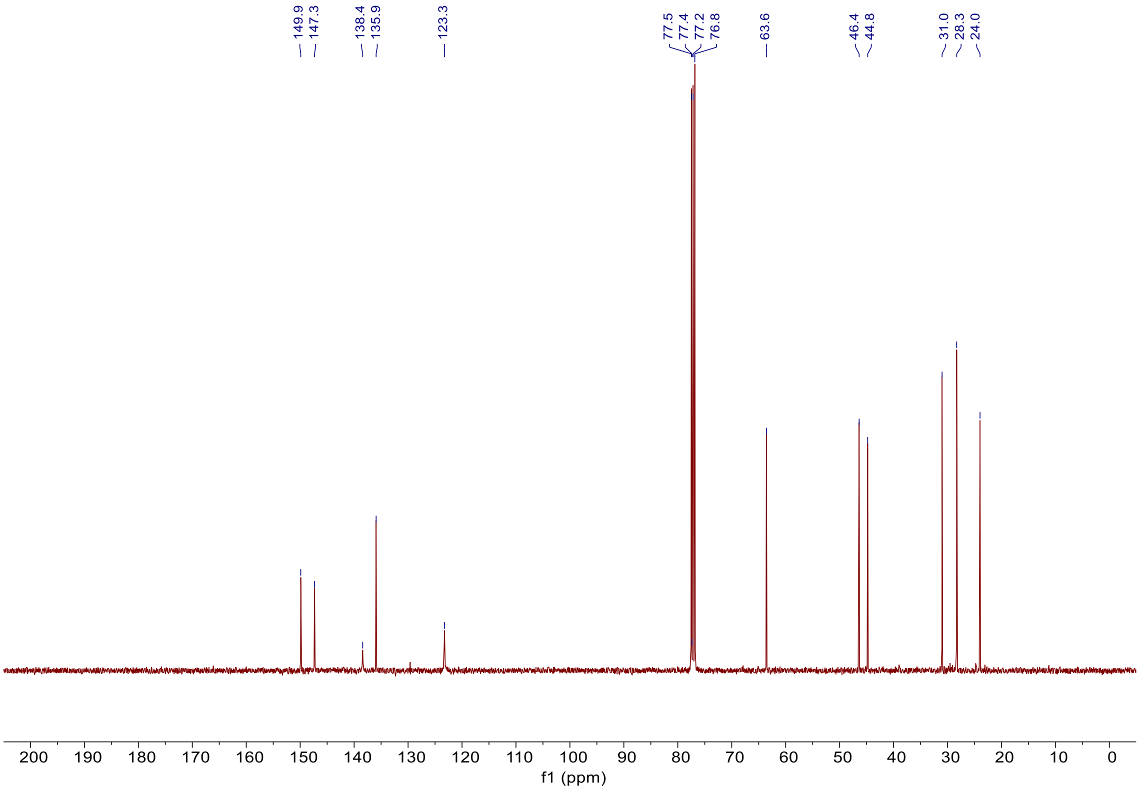

400 MHz ^{1}H NMR spectrum; 100.6 MHz ^{13}C NMR spectrum; $CDCl_3$ of **8t**

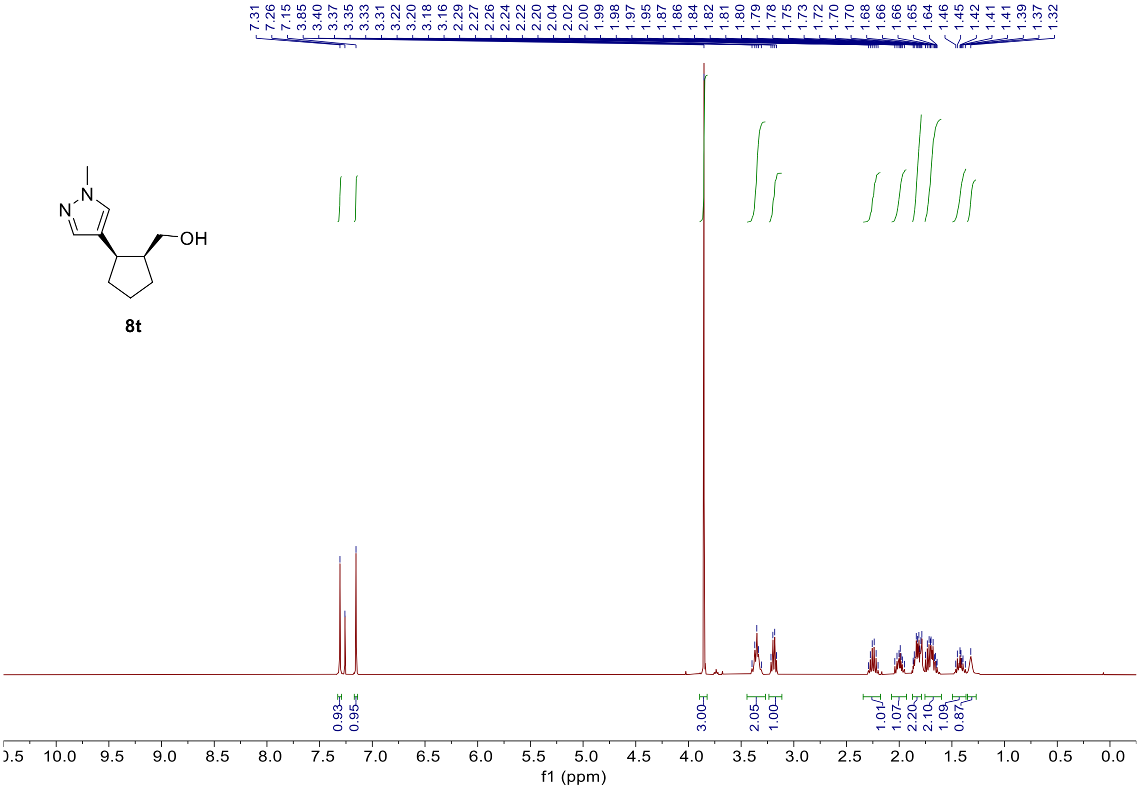

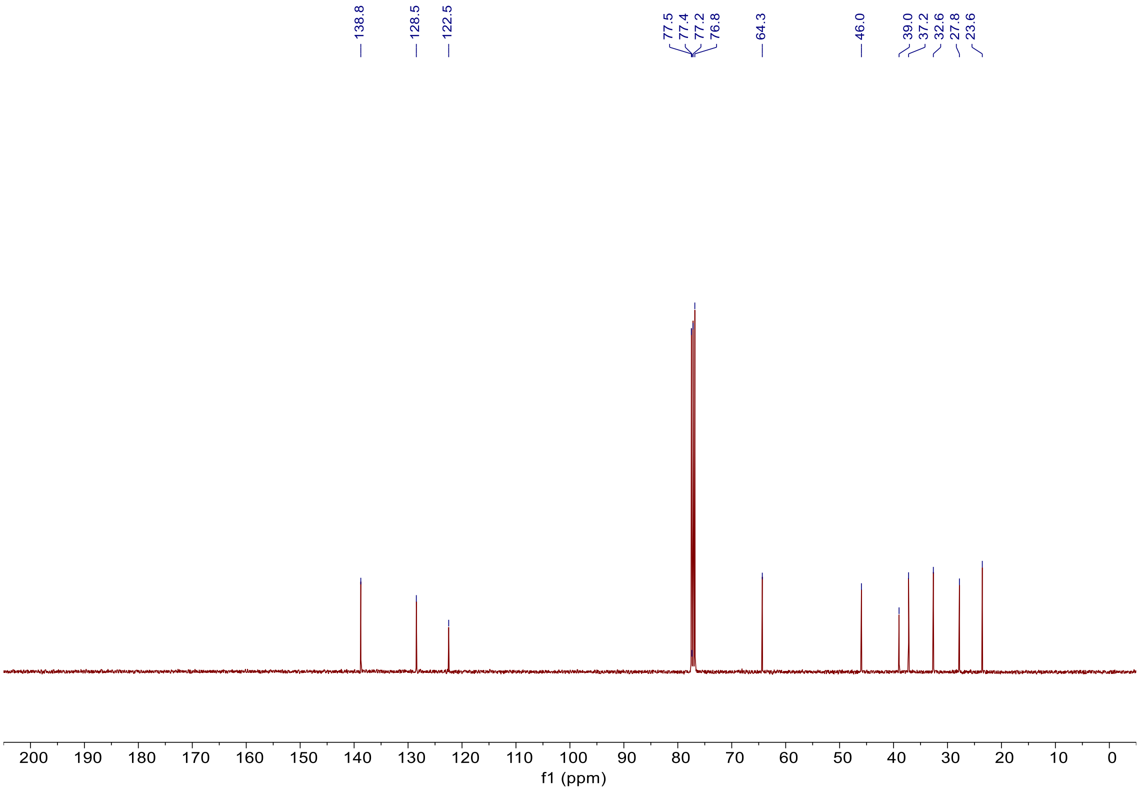

400 MHz ^{1}H NMR spectrum; 100.6 MHz ^{13}C NMR spectrum; $CDCl_3$ of **8u**

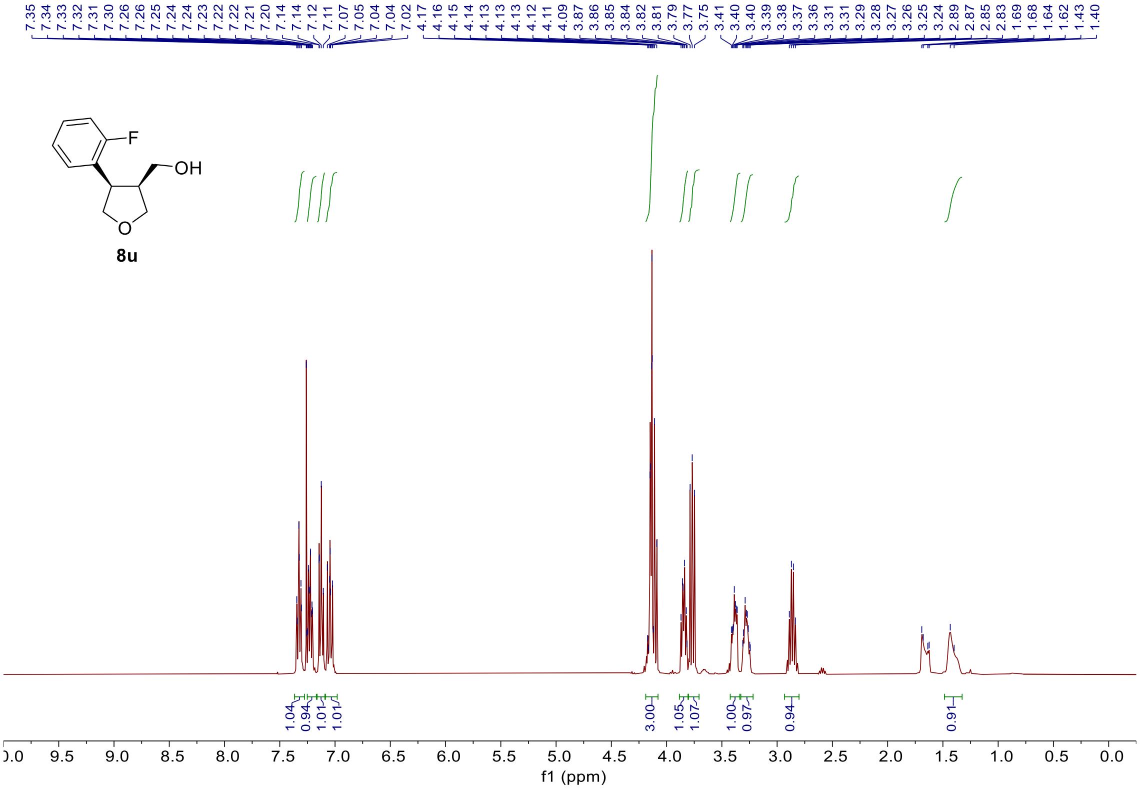

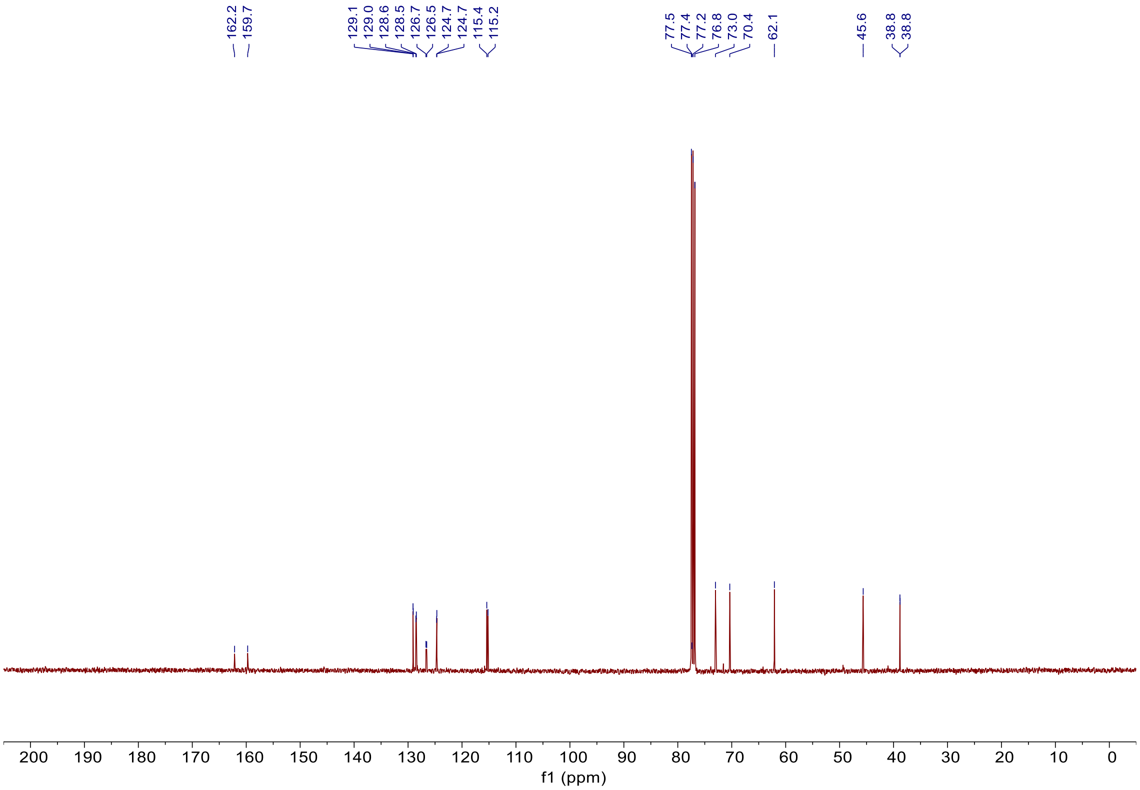

400 MHz ^{1}H NMR spectrum; 100.6 MHz ^{13}C NMR spectrum; $CDCl_3$ of **8v**

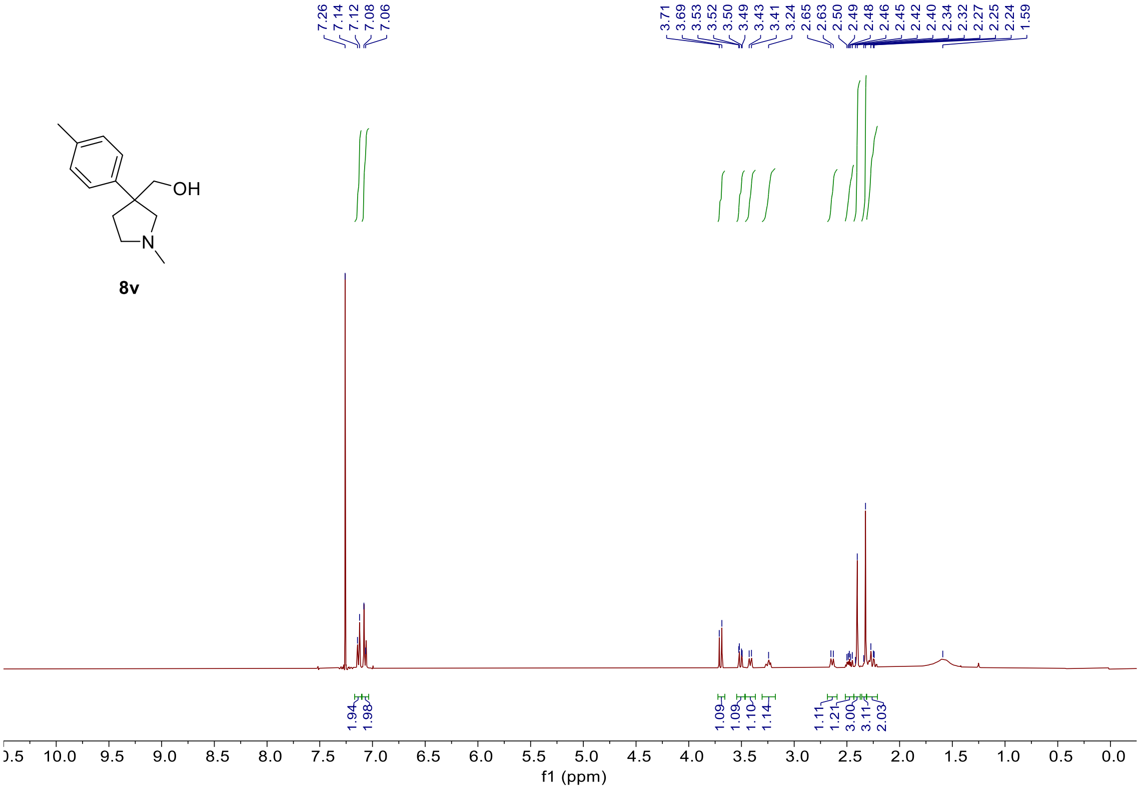

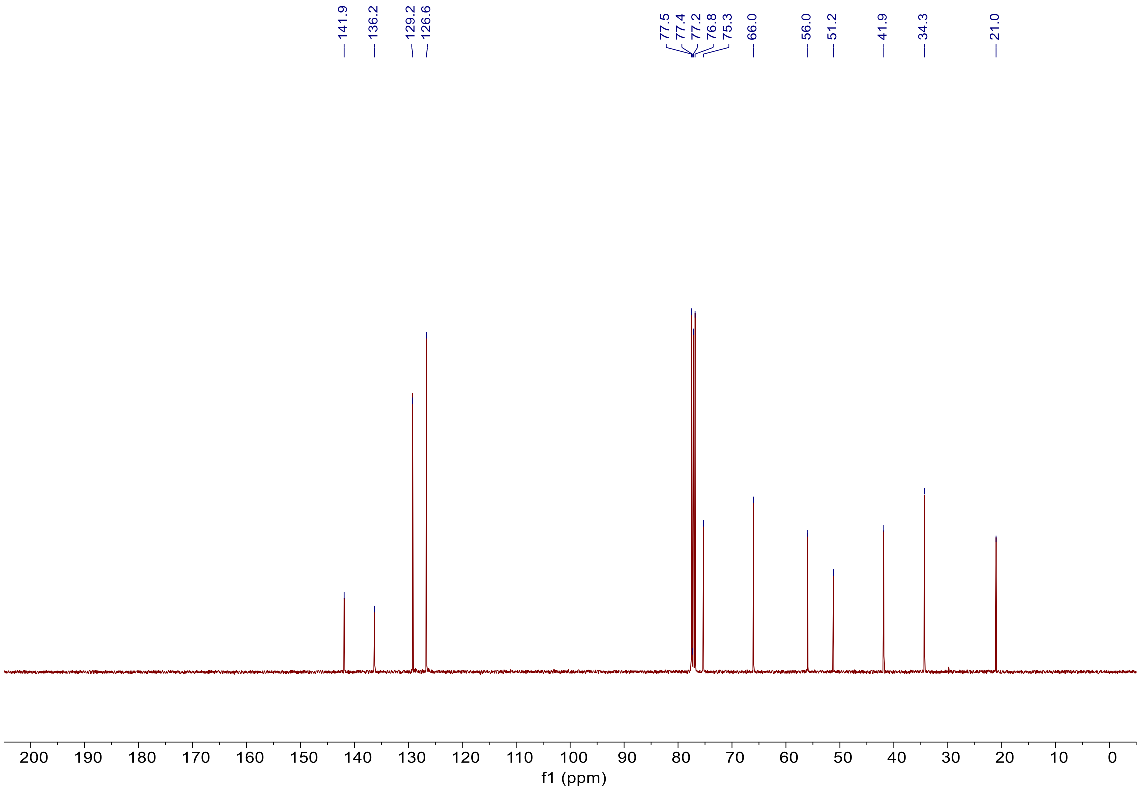

400 MHz ^{1}H NMR spectrum; 100.6 MHz ^{13}C NMR spectrum; $CDCl_3$ of **8w**

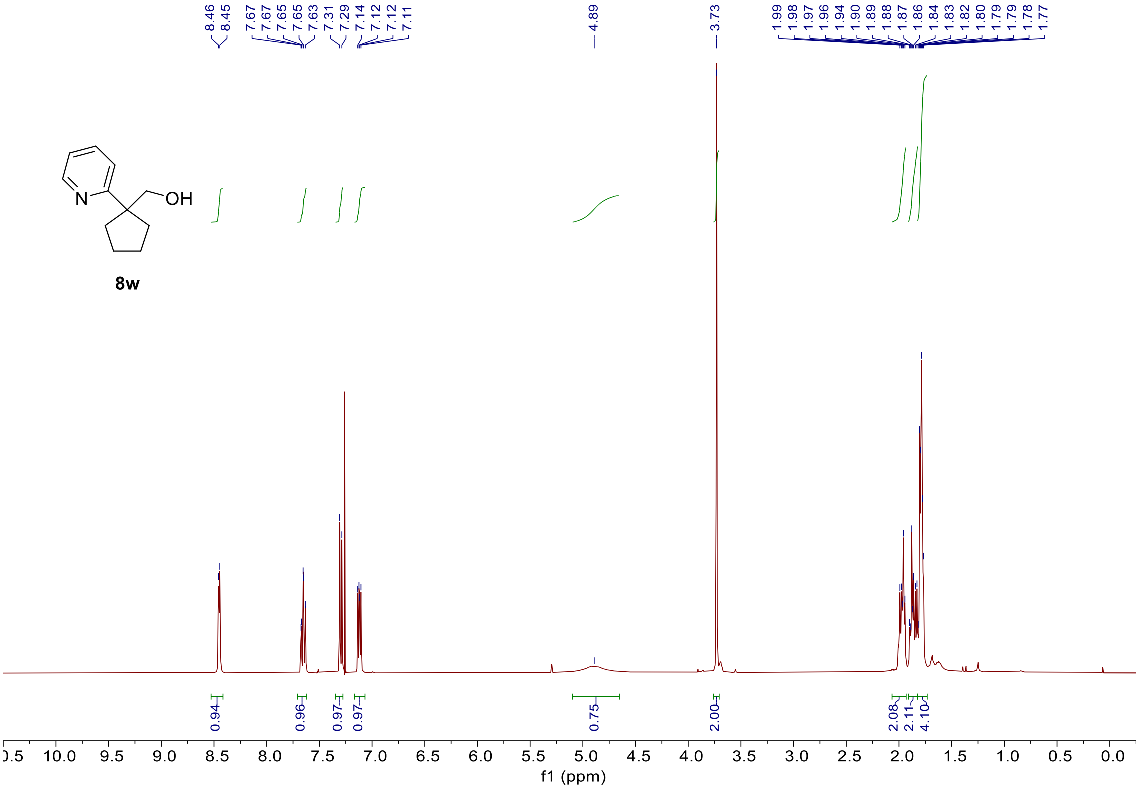

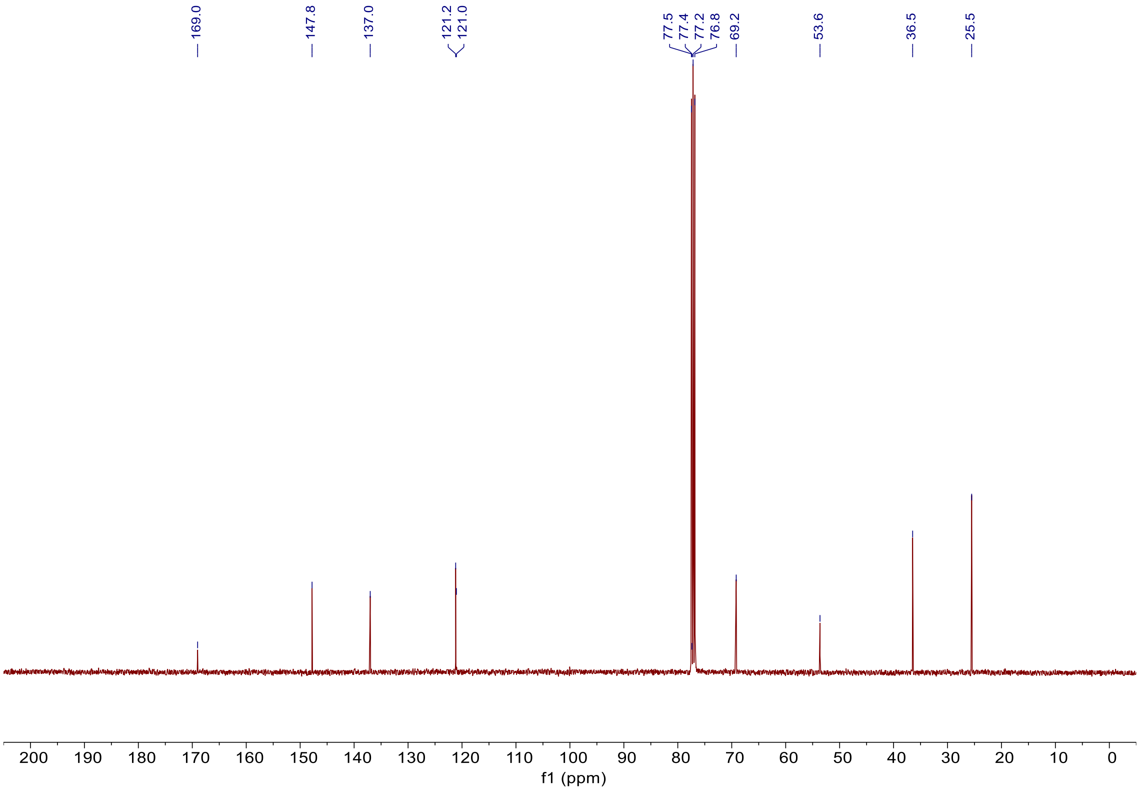

400 MHz ^{1}H NMR spectrum; 100.6 MHz ^{13}C NMR spectrum; $CDCl_3$ of **8x**

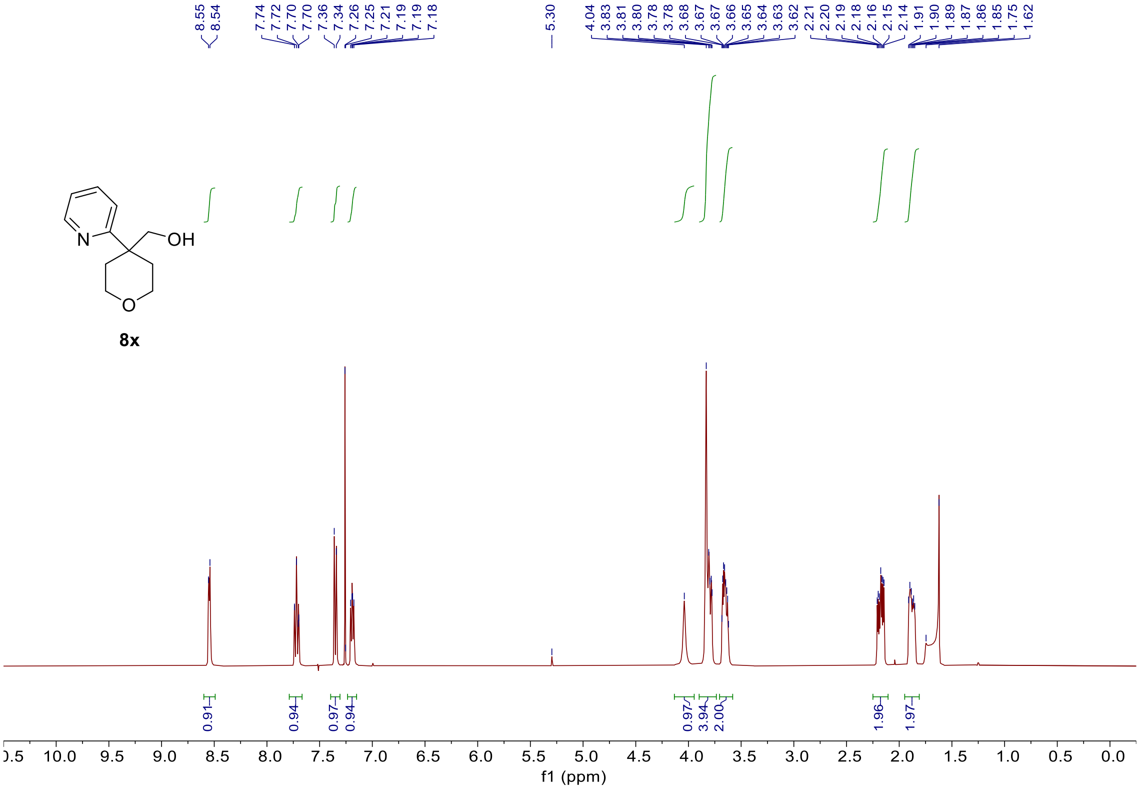

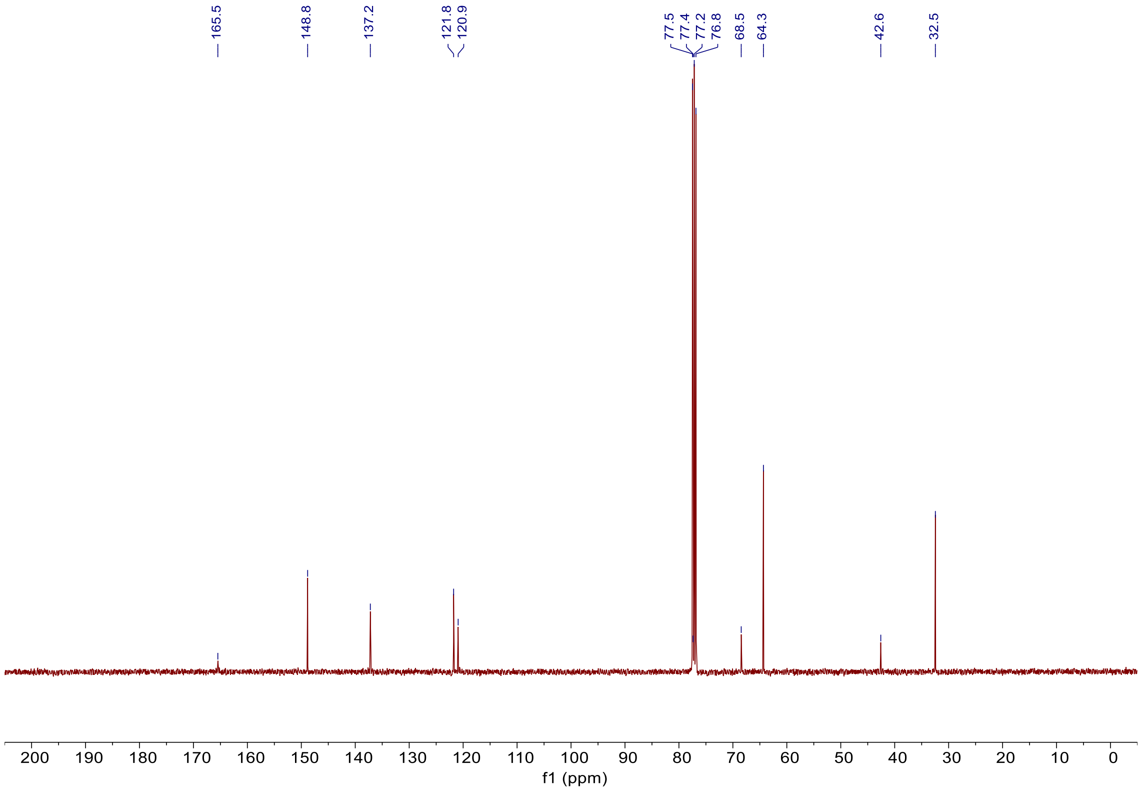

400 MHz ^{1}H NMR spectrum; 100.6 MHz ^{13}C NMR spectrum; $CDCl_3$ of **8y**

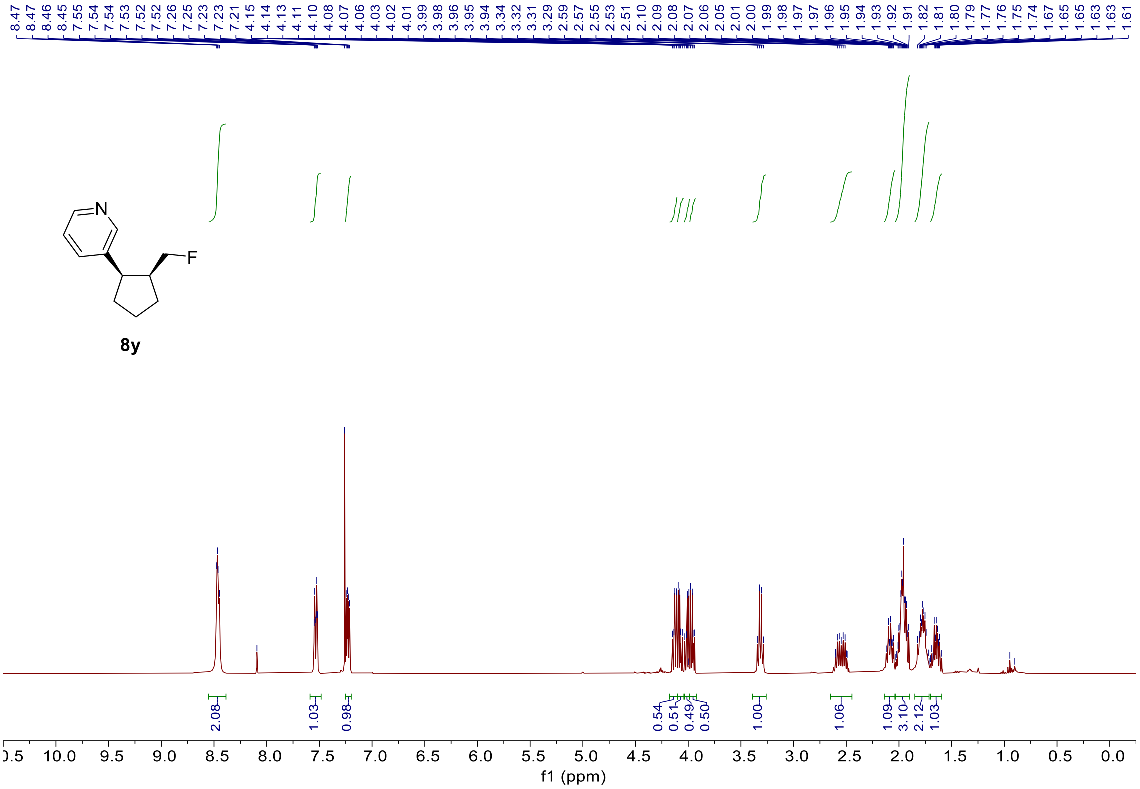

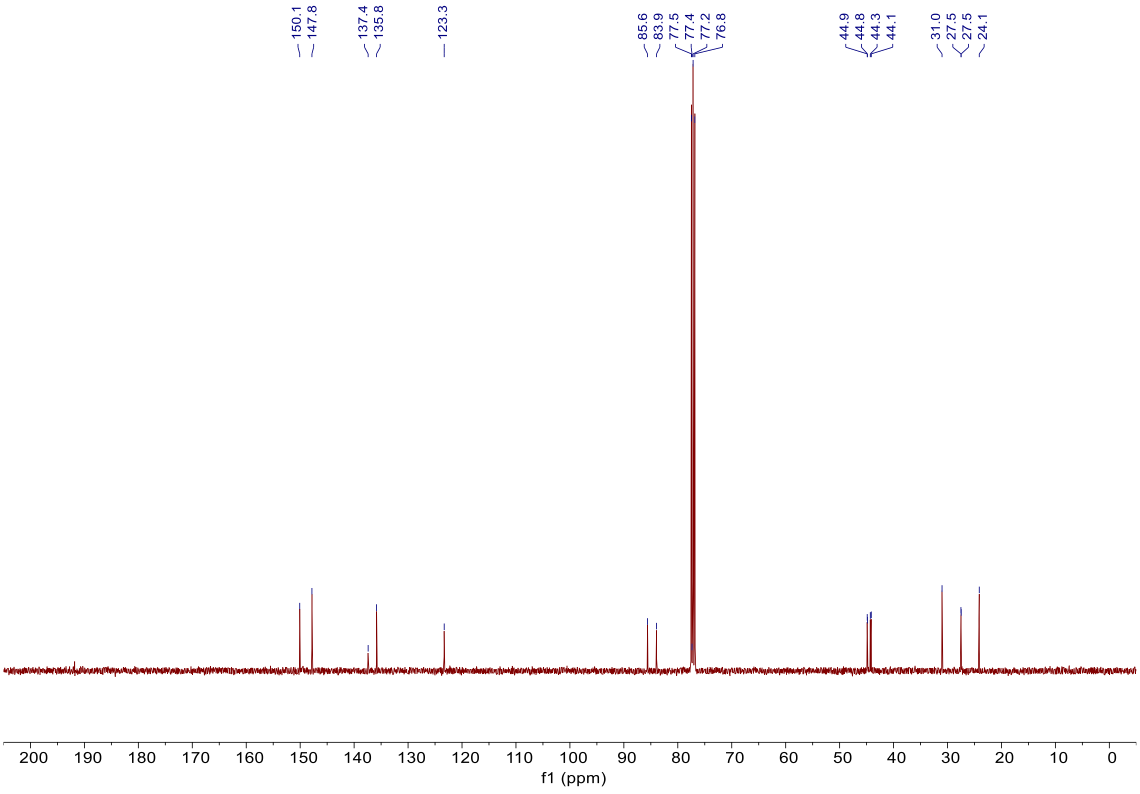

400 MHz ^{1}H NMR spectrum; 100.6 MHz ^{13}C NMR spectrum; $CDCl_3$ of **8z**

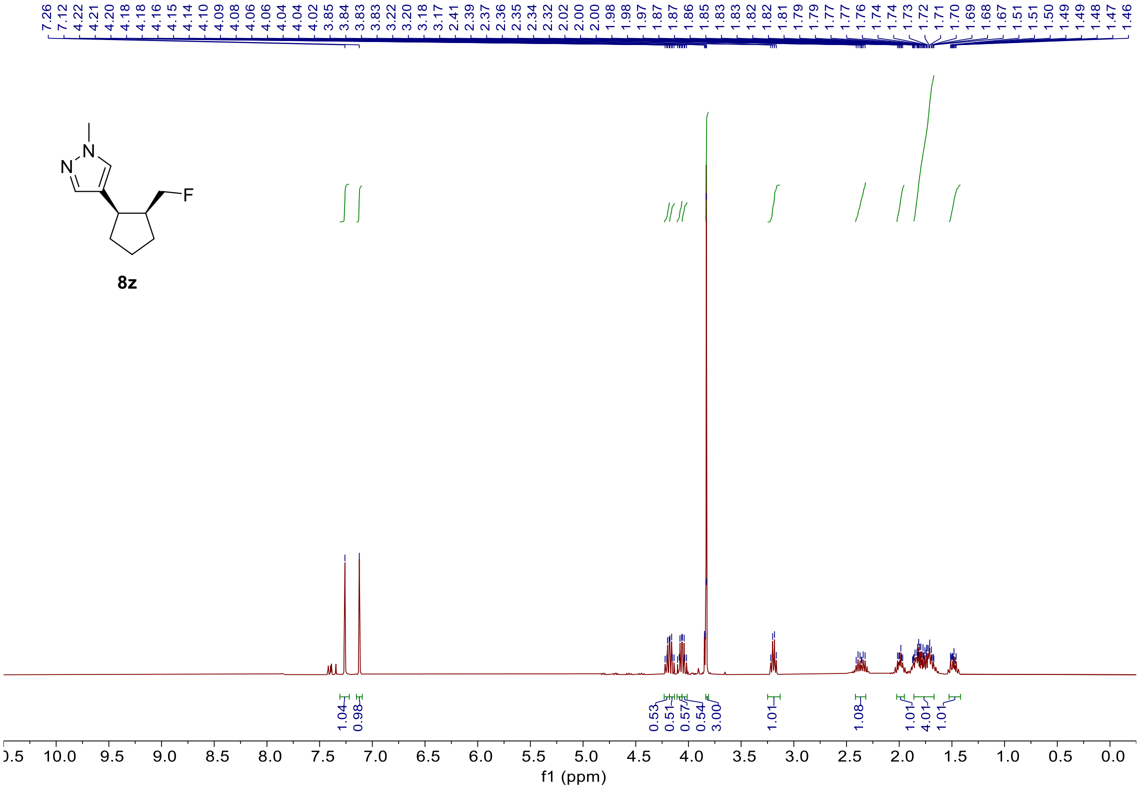

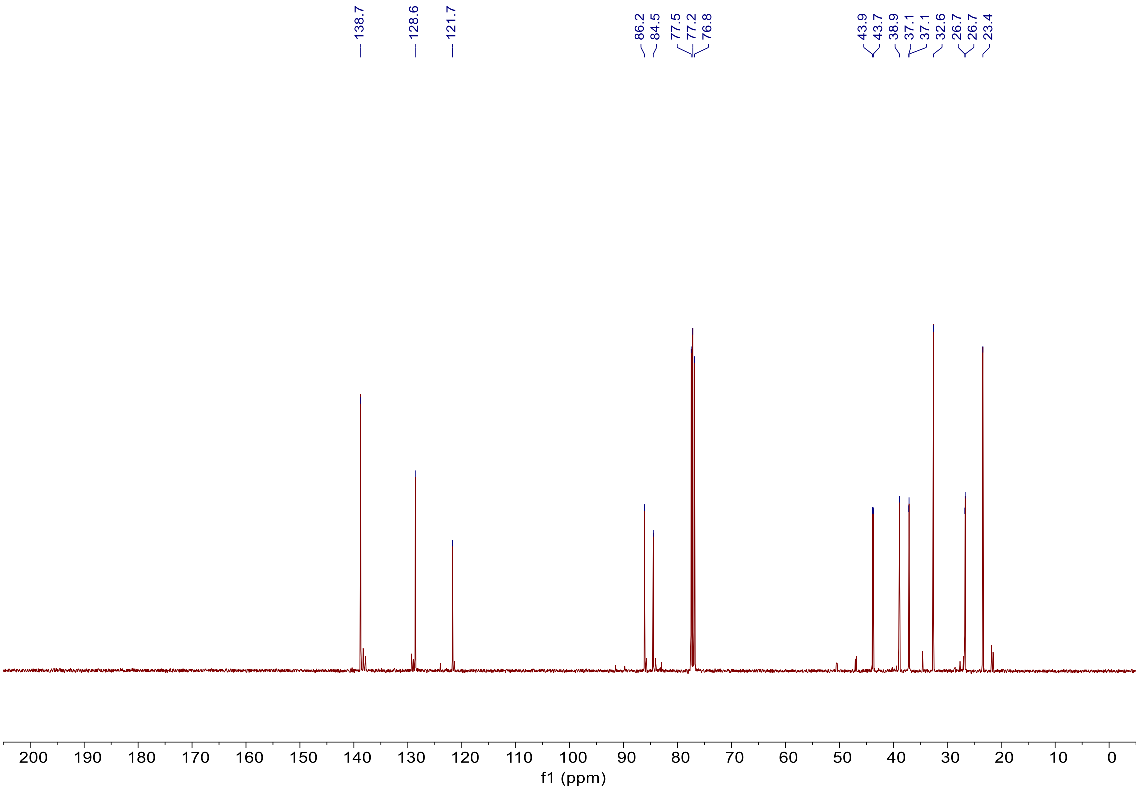

400 MHz ^{1}H NMR spectrum; 100.6 MHz ^{13}C NMR spectrum; $CDCl_3$ of **8aa**

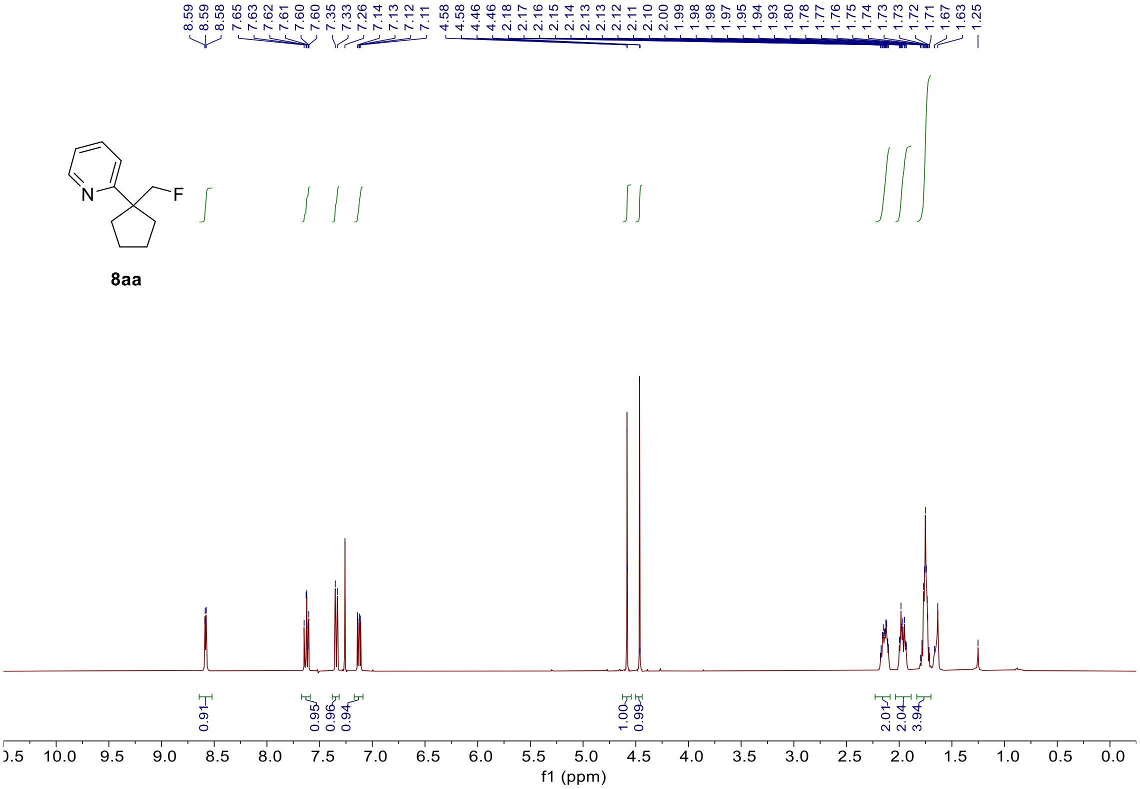

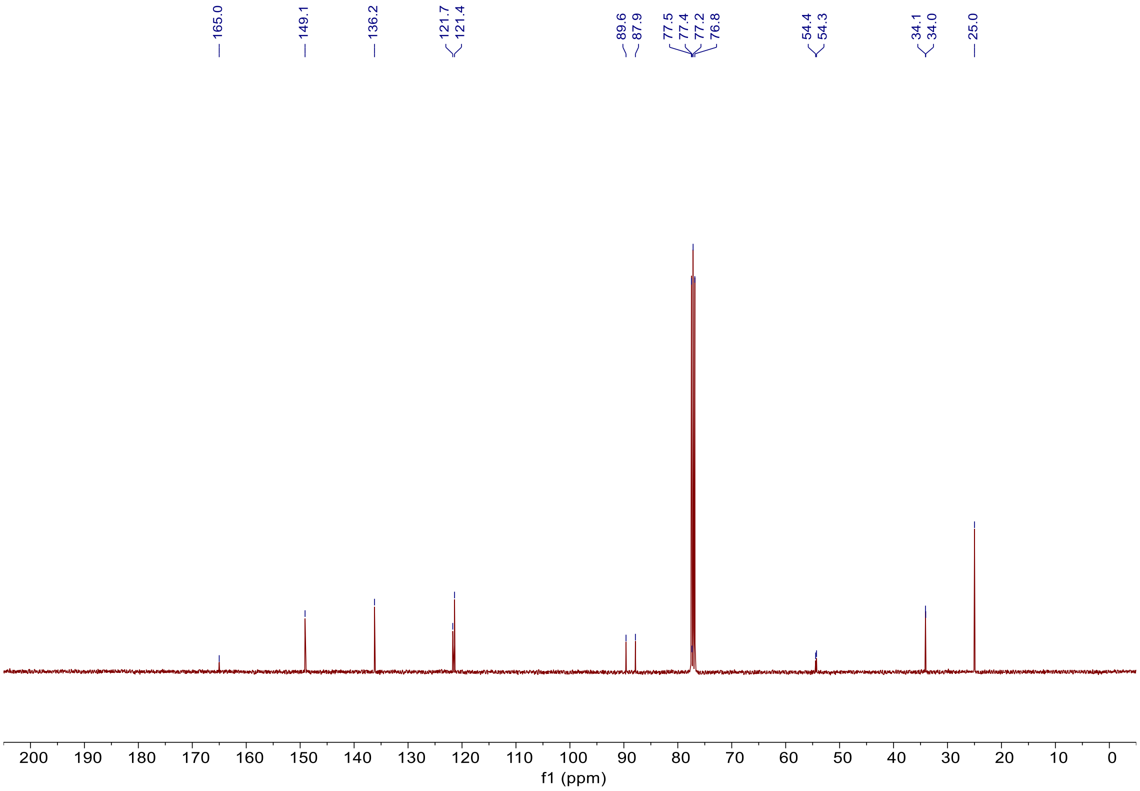

400 MHz ^{1}H NMR spectrum; 100.6 MHz ^{13}C NMR spectrum; $CDCl_3$ of **8ab**

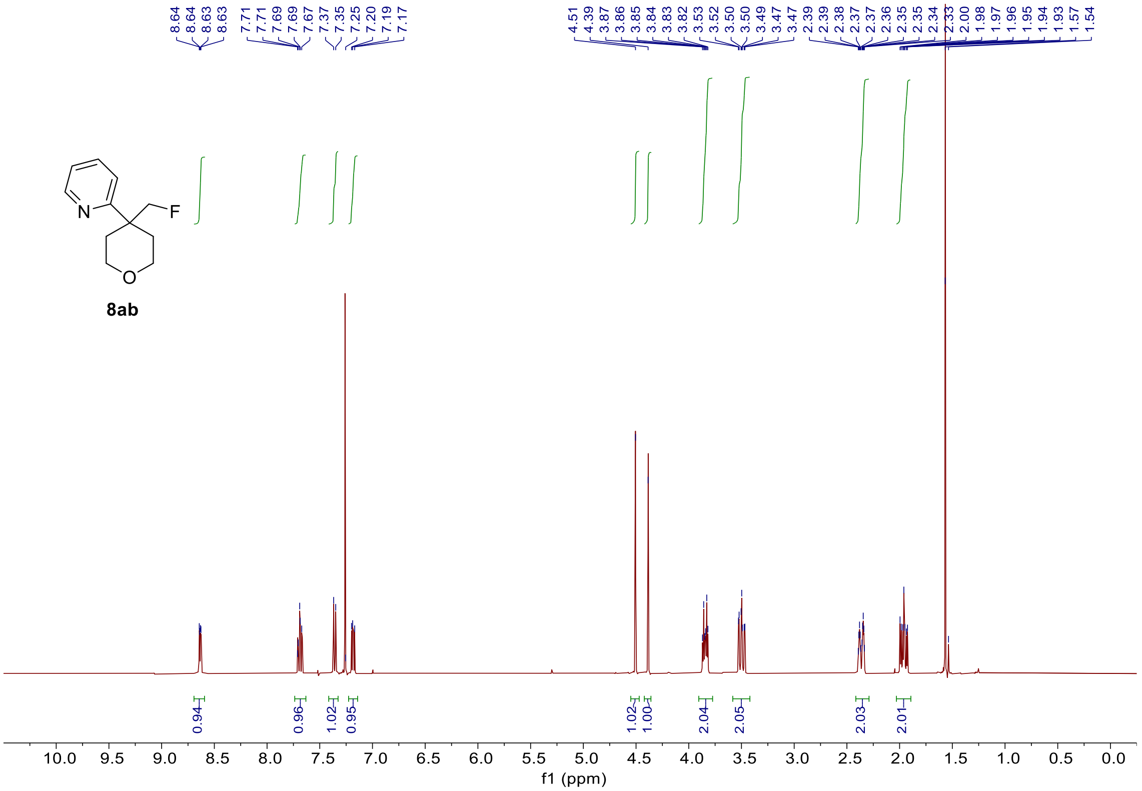

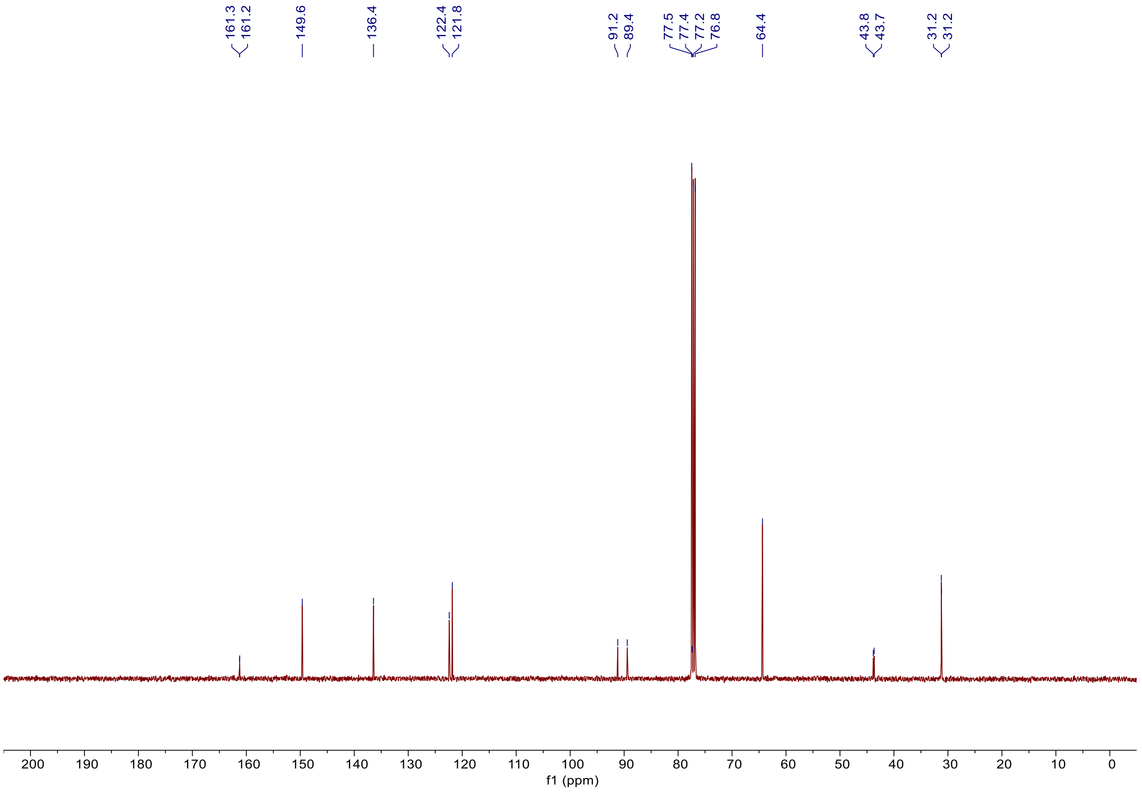

400 MHz ^{1}H NMR spectrum; 100.6 MHz ^{13}C NMR spectrum; $CDCl_3$ of **S11**

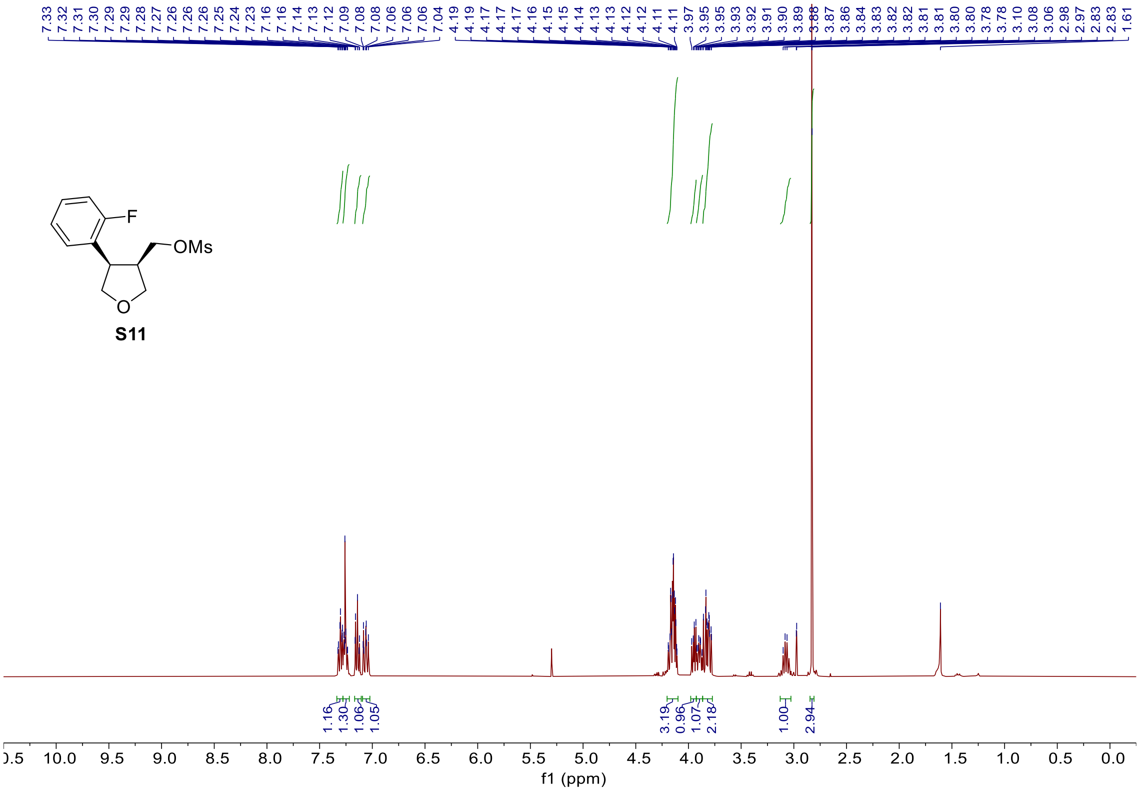

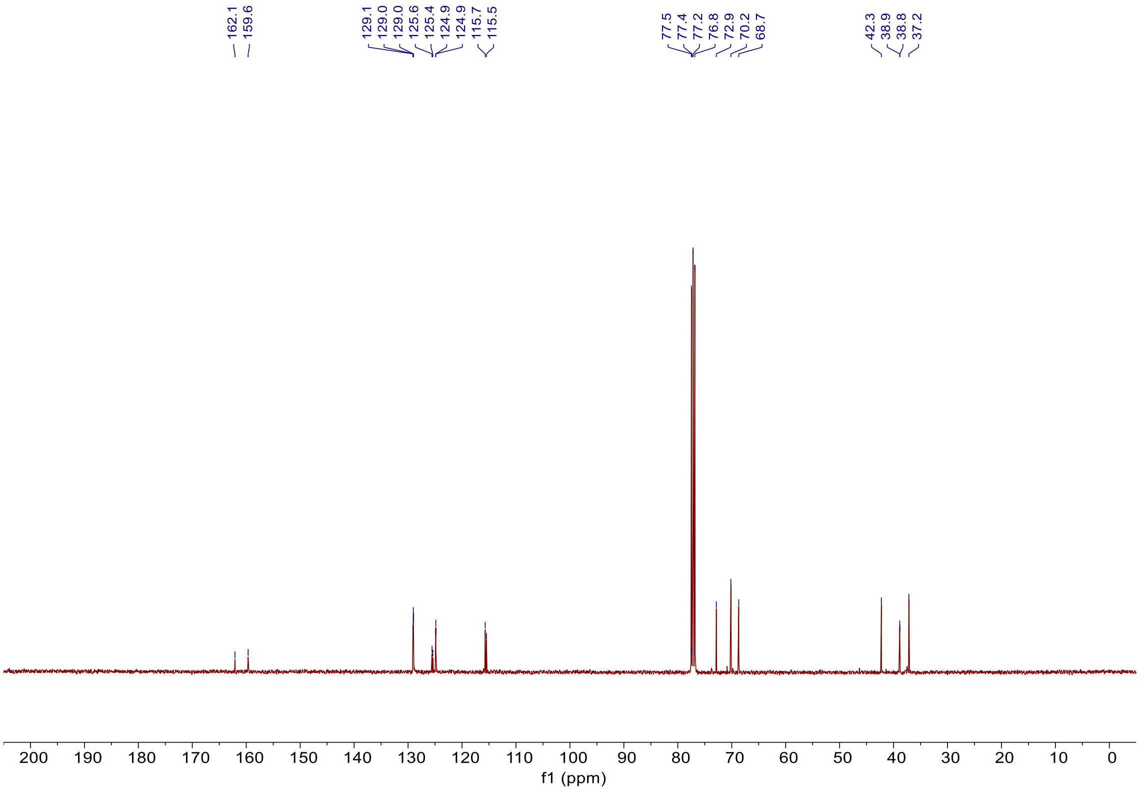

400 MHz ^{1}H NMR spectrum; 100.6 MHz ^{13}C NMR spectrum; $CDCl_3$ of **S12**

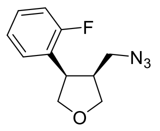

S12

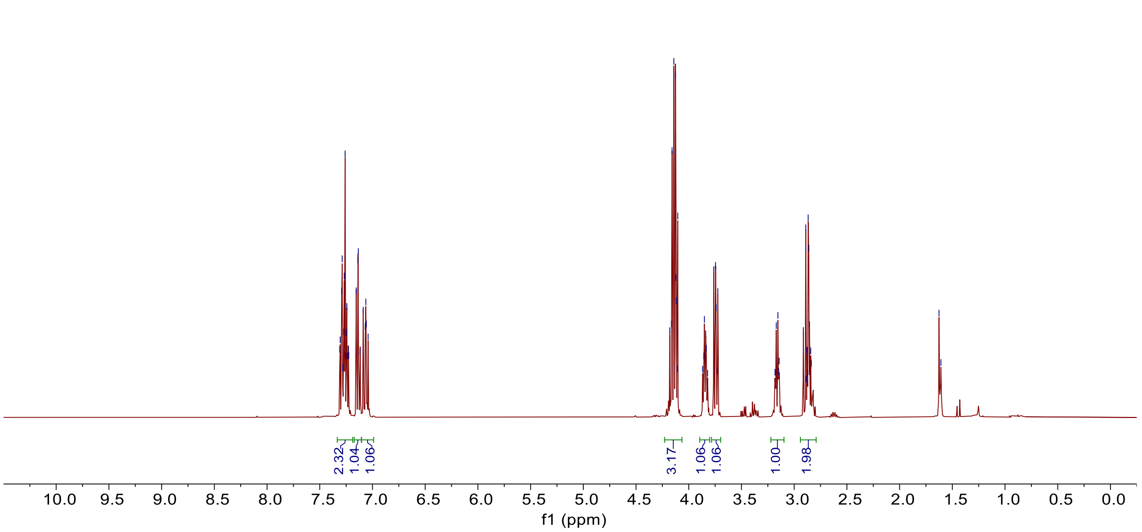

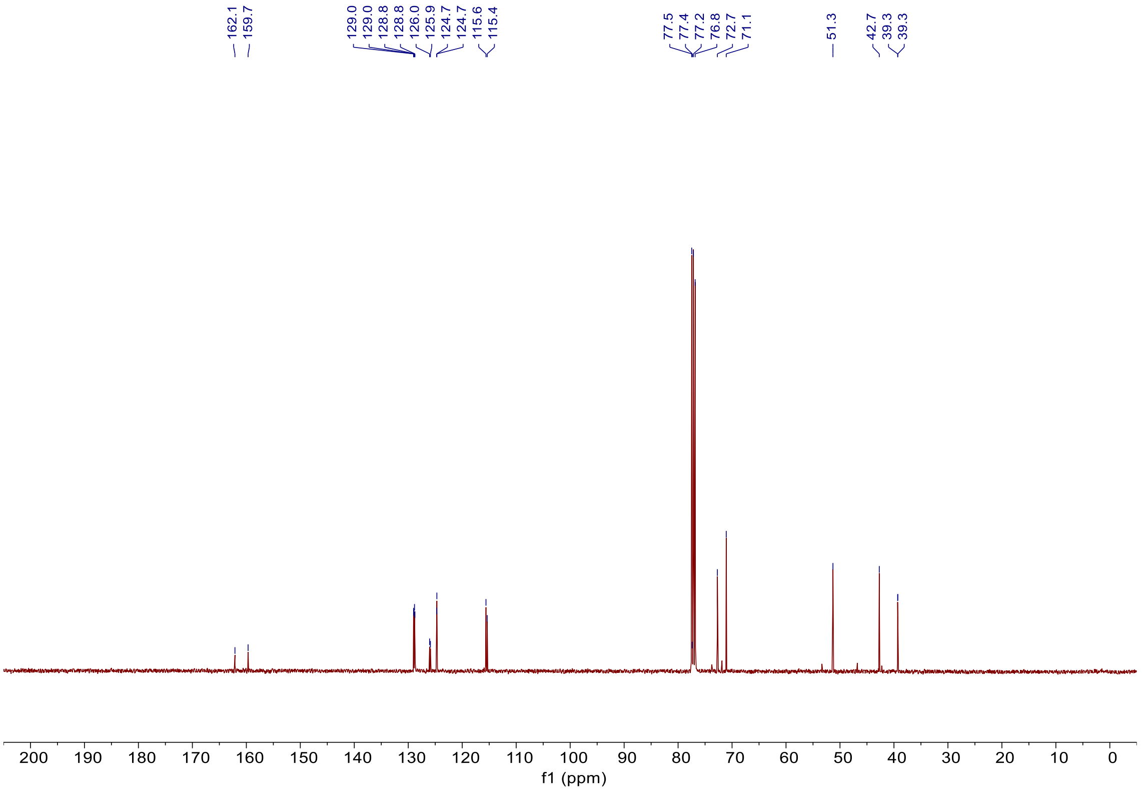

400 MHz ^{1}H NMR spectrum; 100.6 MHz ^{13}C NMR spectrum; $CDCl_3$ of **8ac**

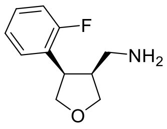

8ac

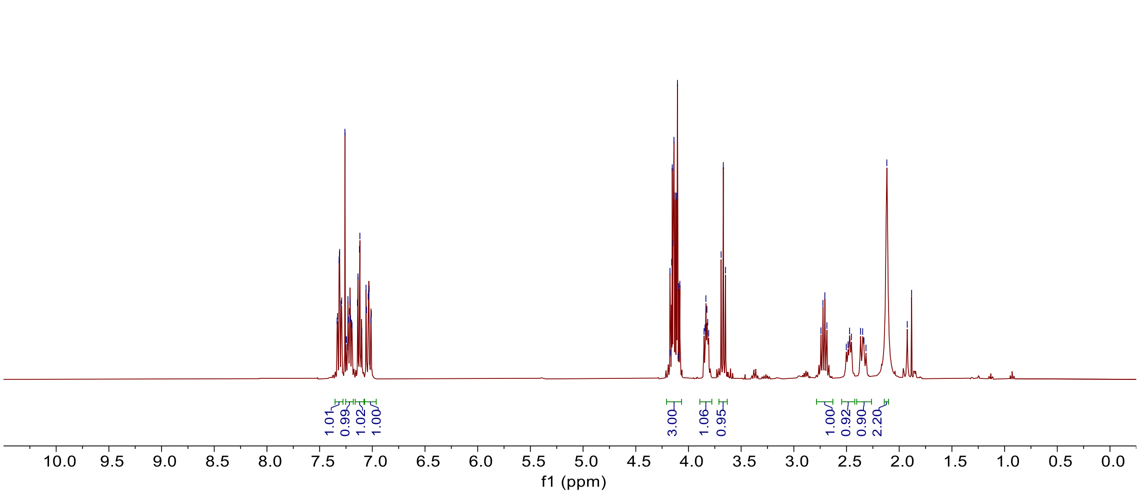

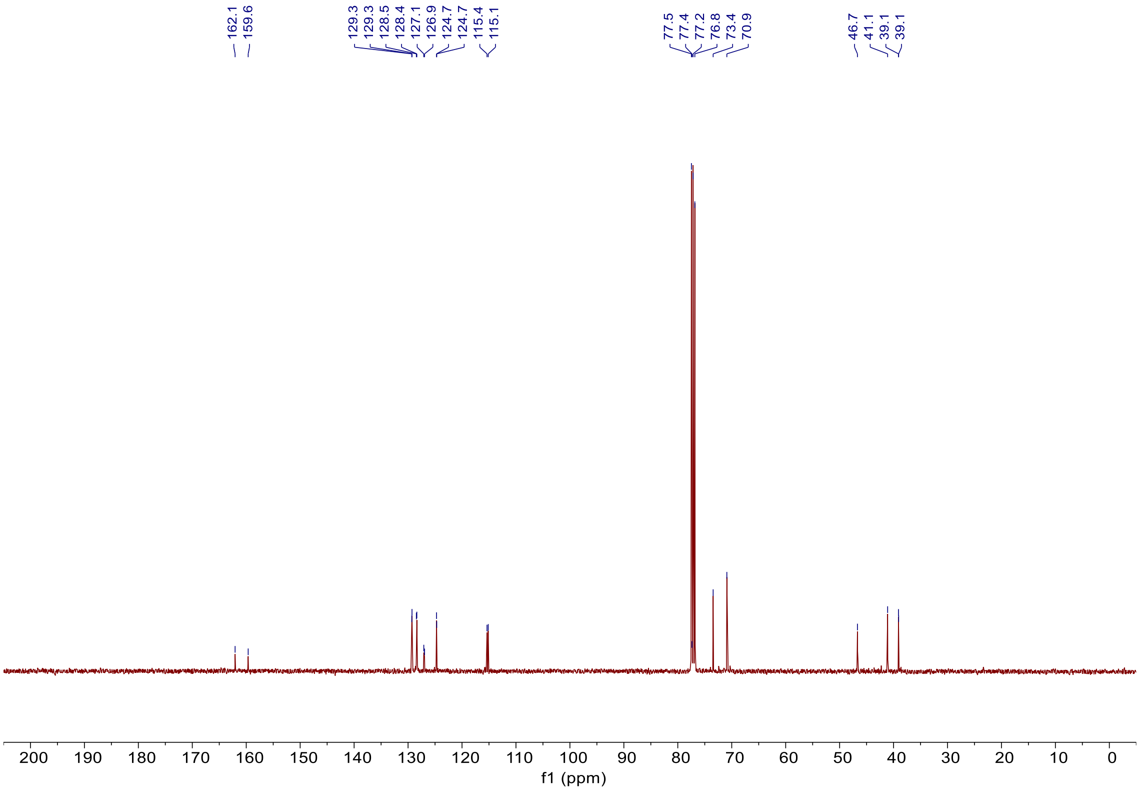

400 MHz ^{1}H NMR spectrum; 100.6 MHz ^{13}C NMR spectrum; $CDCl_3$ of **S13**

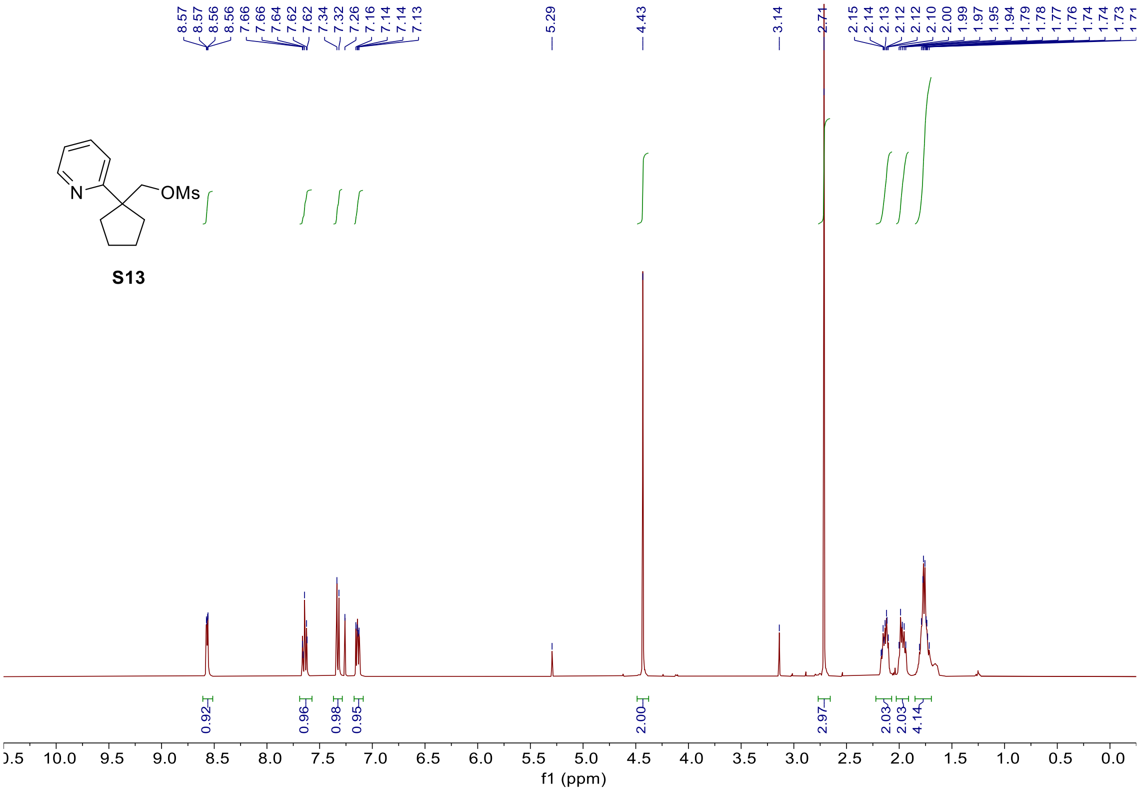

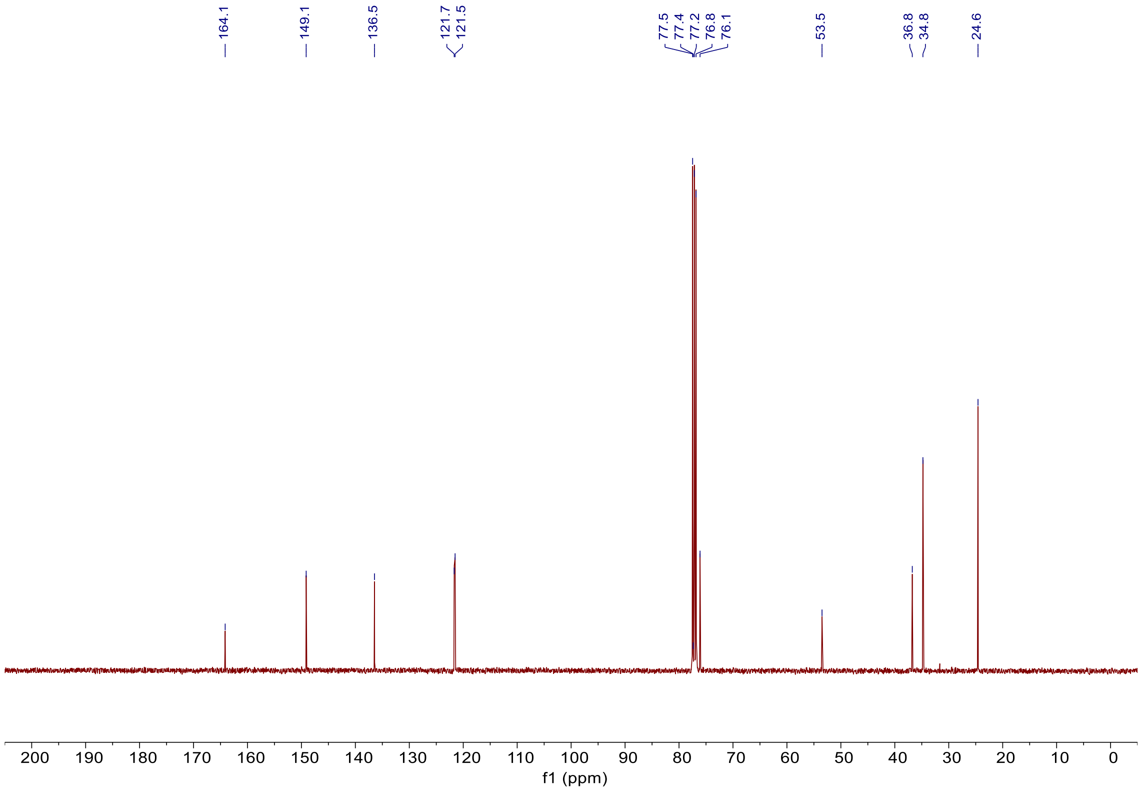

400 MHz ^{1}H NMR spectrum; 100.6 MHz ^{13}C NMR spectrum; $CDCl_3$ of **S14**

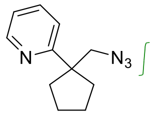

S14

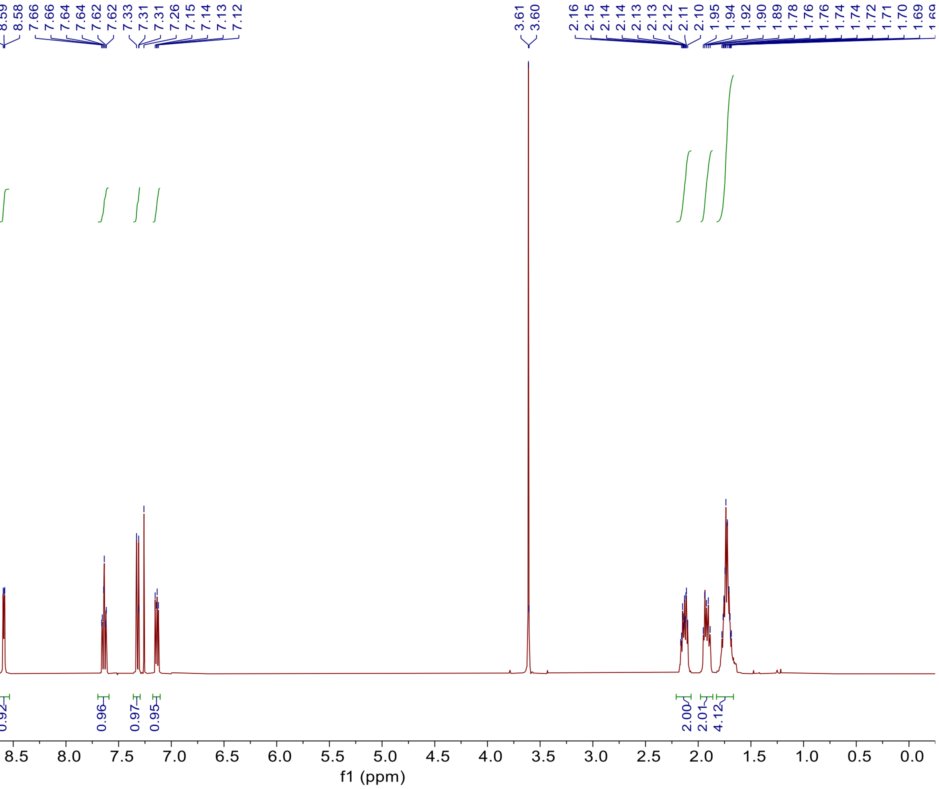

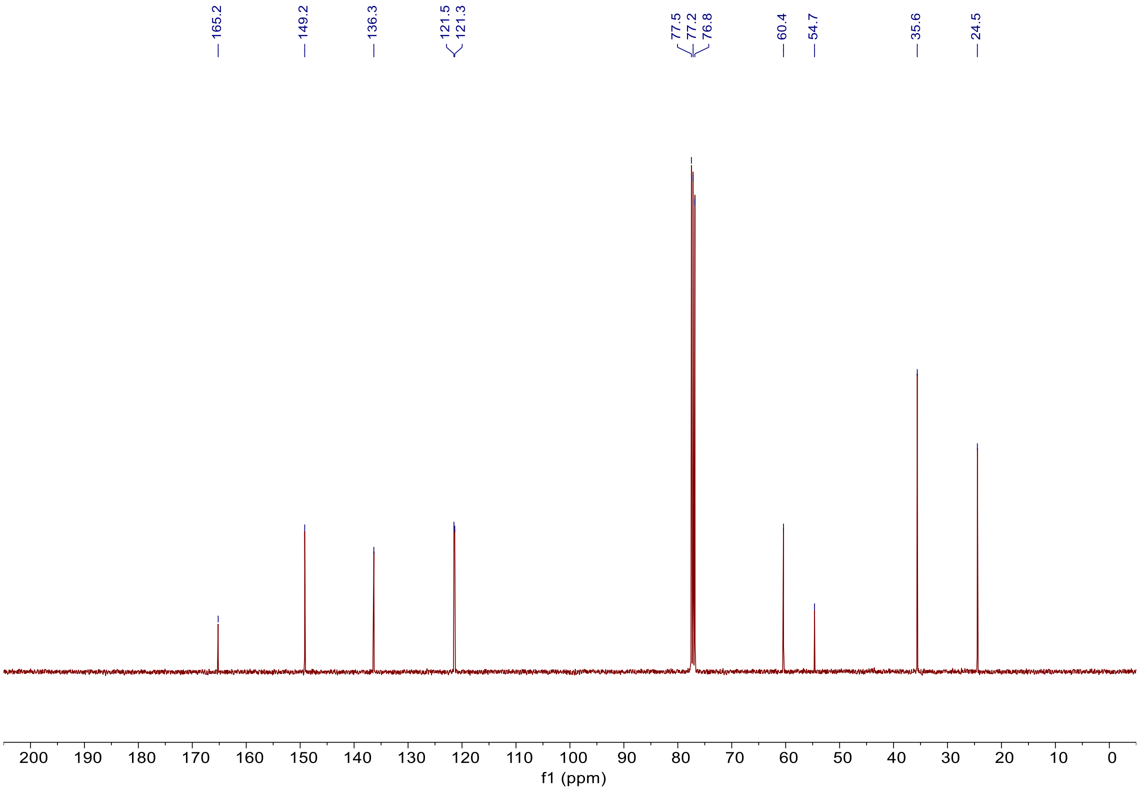

400 MHz ^{1}H NMR spectrum; 100.6 MHz ^{13}C NMR spectrum; $CDCl_3$ of **8ad**

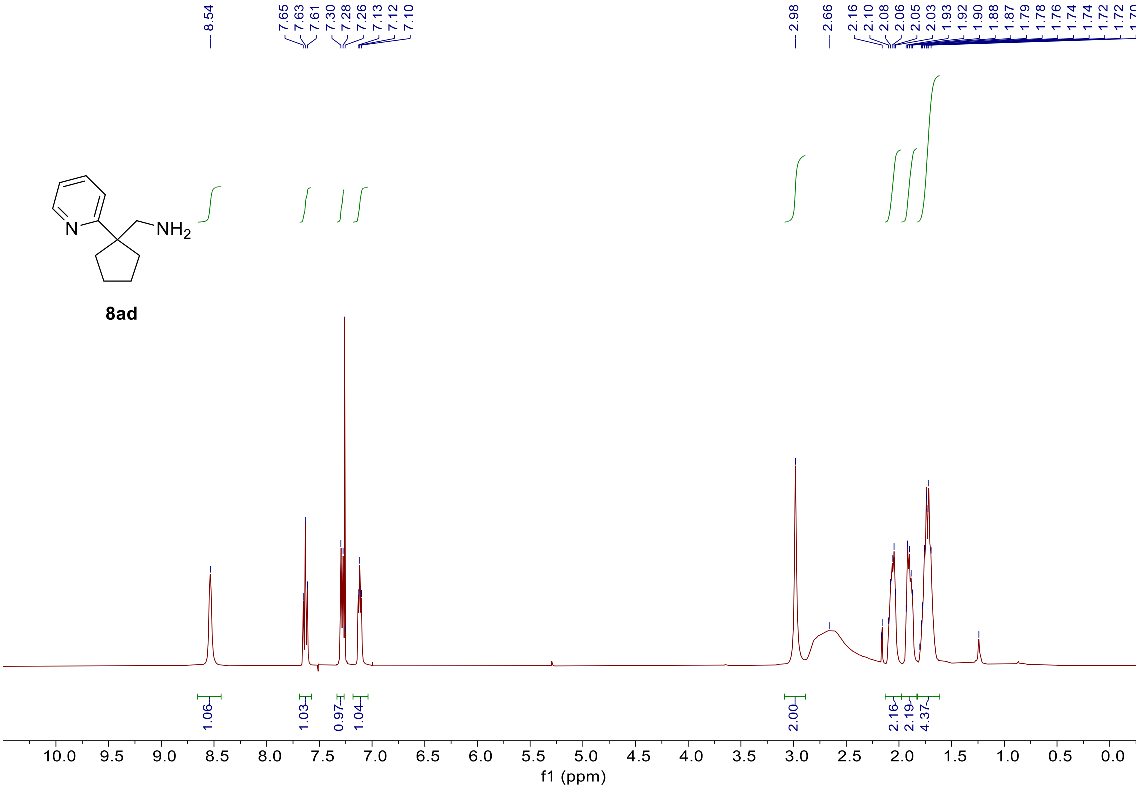

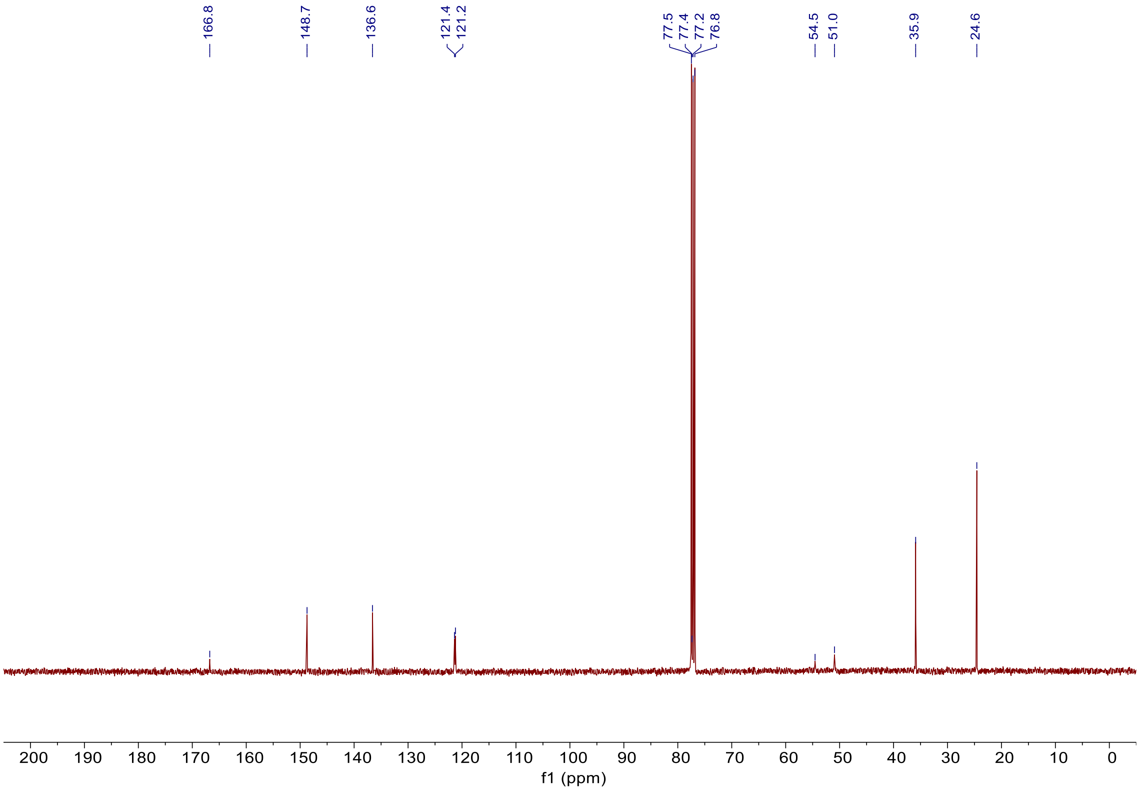

400 MHz ^{1}H NMR spectrum; 100.6 MHz ^{13}C NMR spectrum; $CDCl_3$ of **S15**

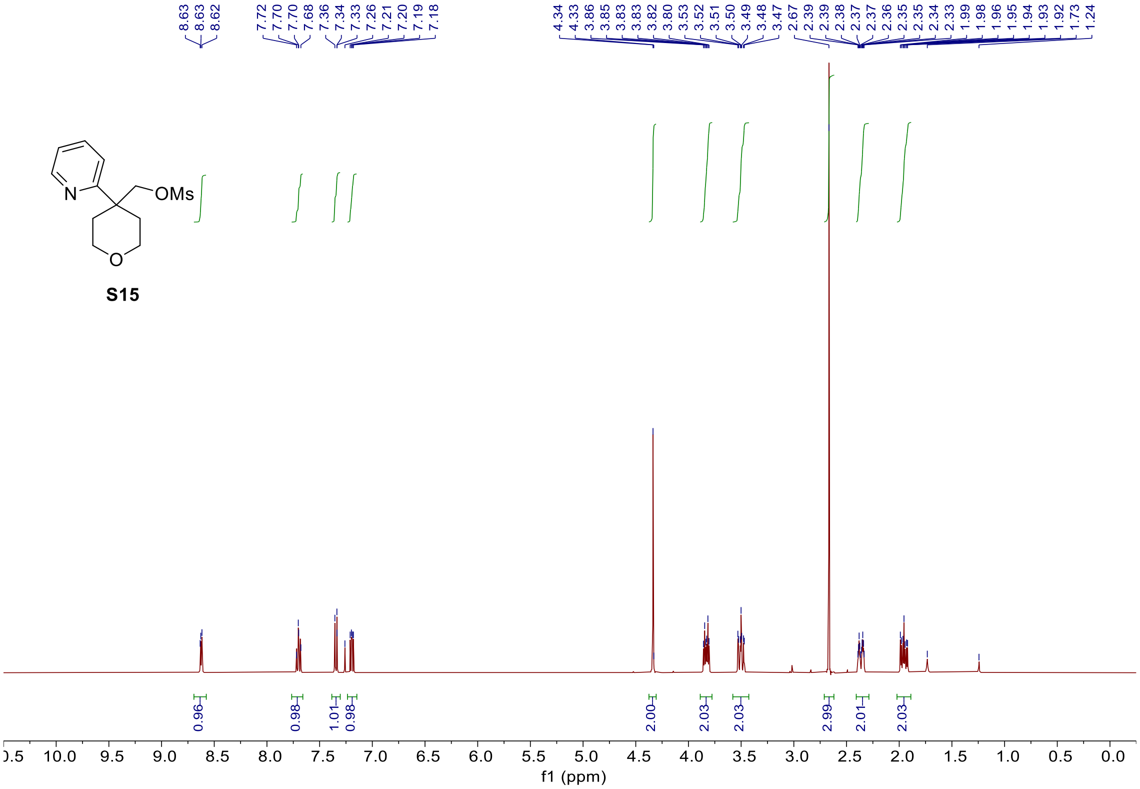

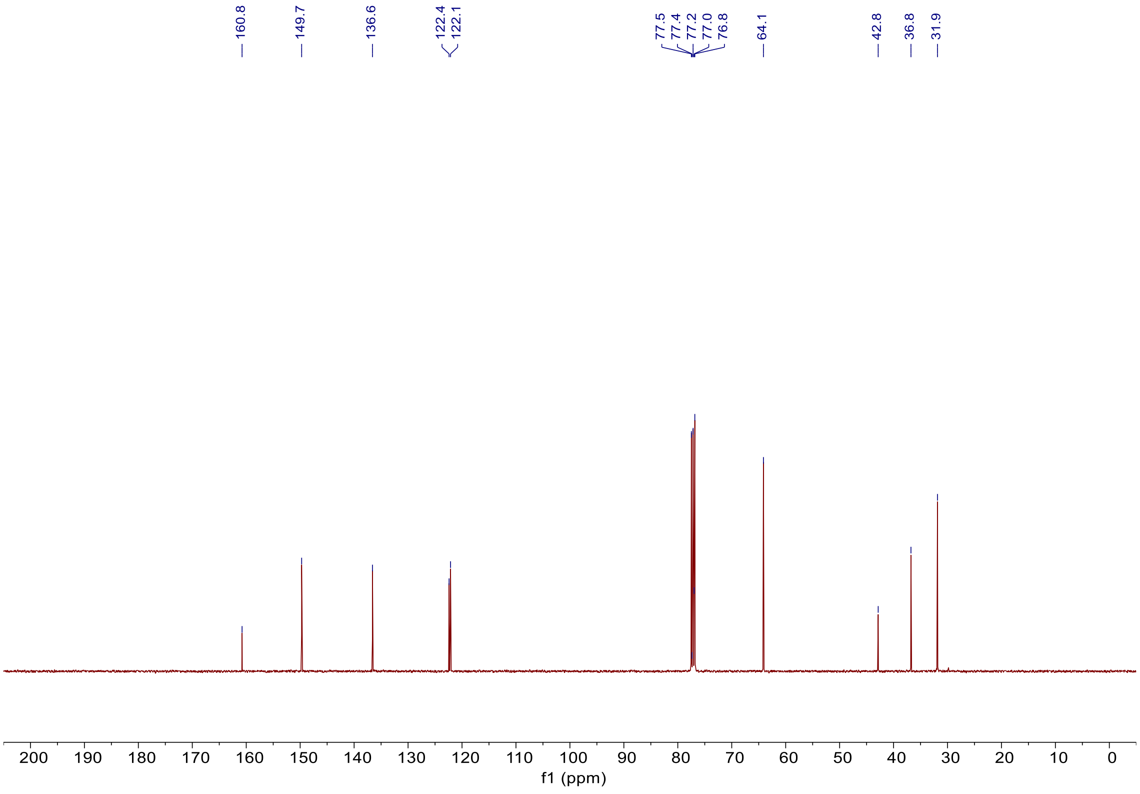

400 MHz ^{1}H NMR spectrum; 100.6 MHz ^{13}C NMR spectrum; $CDCl_3$ of **S16**

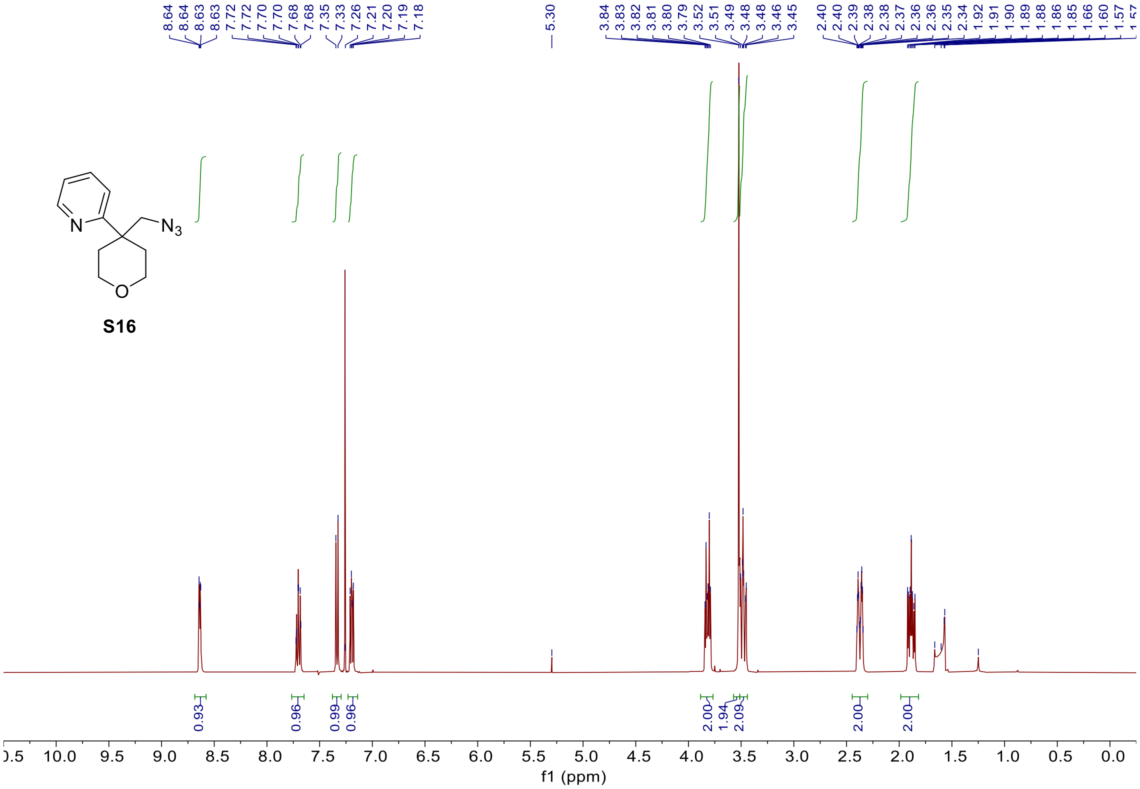

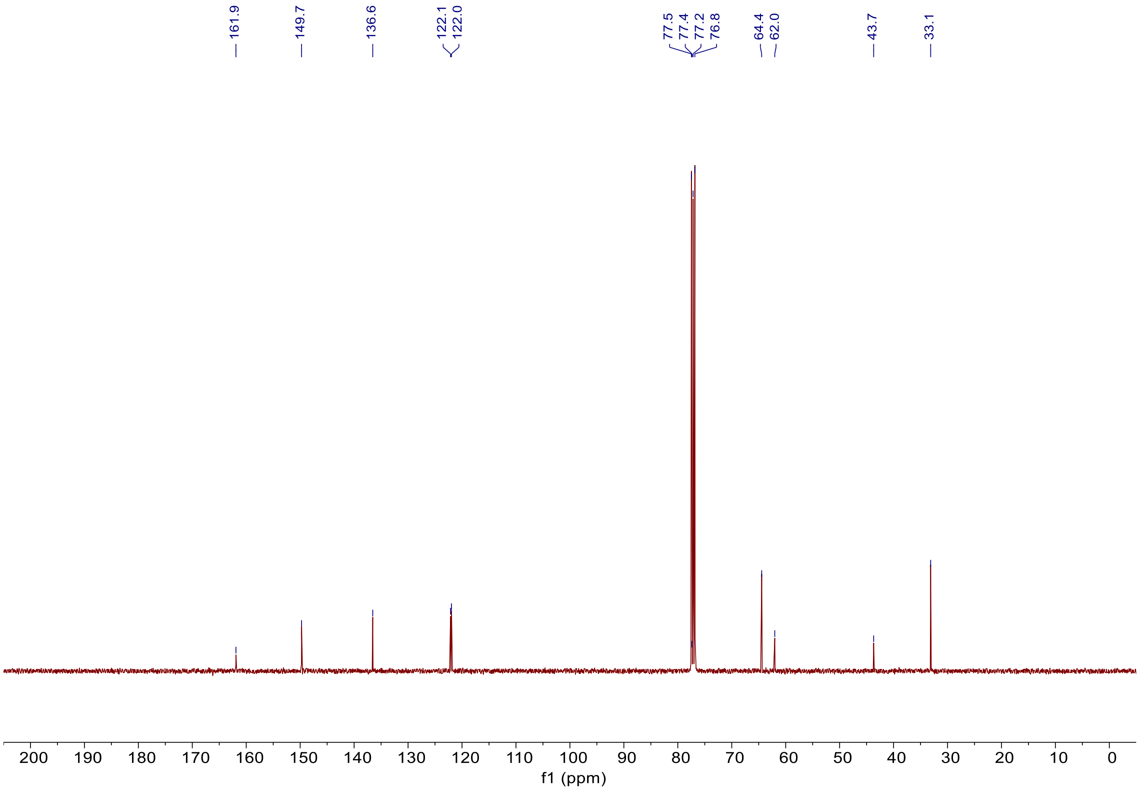

400 MHz ^{1}H NMR spectrum; 100.6 MHz ^{13}C NMR spectrum; $CDCl_3$ of **8ae**

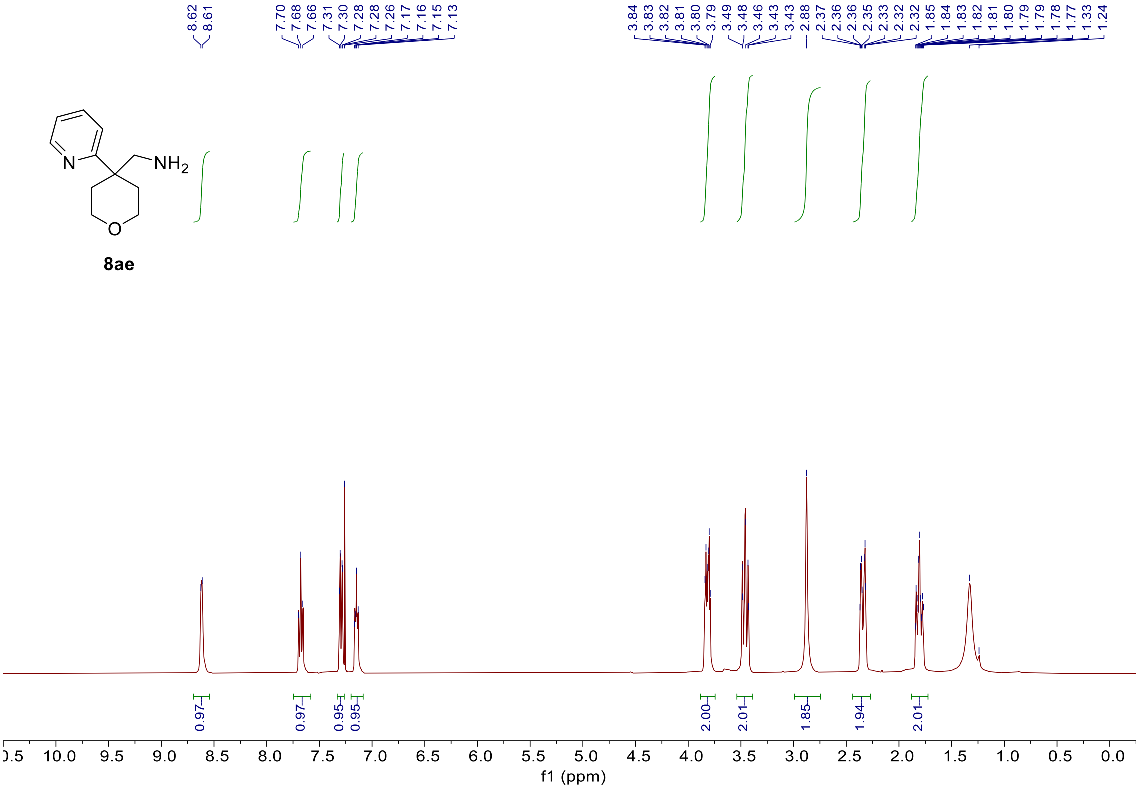

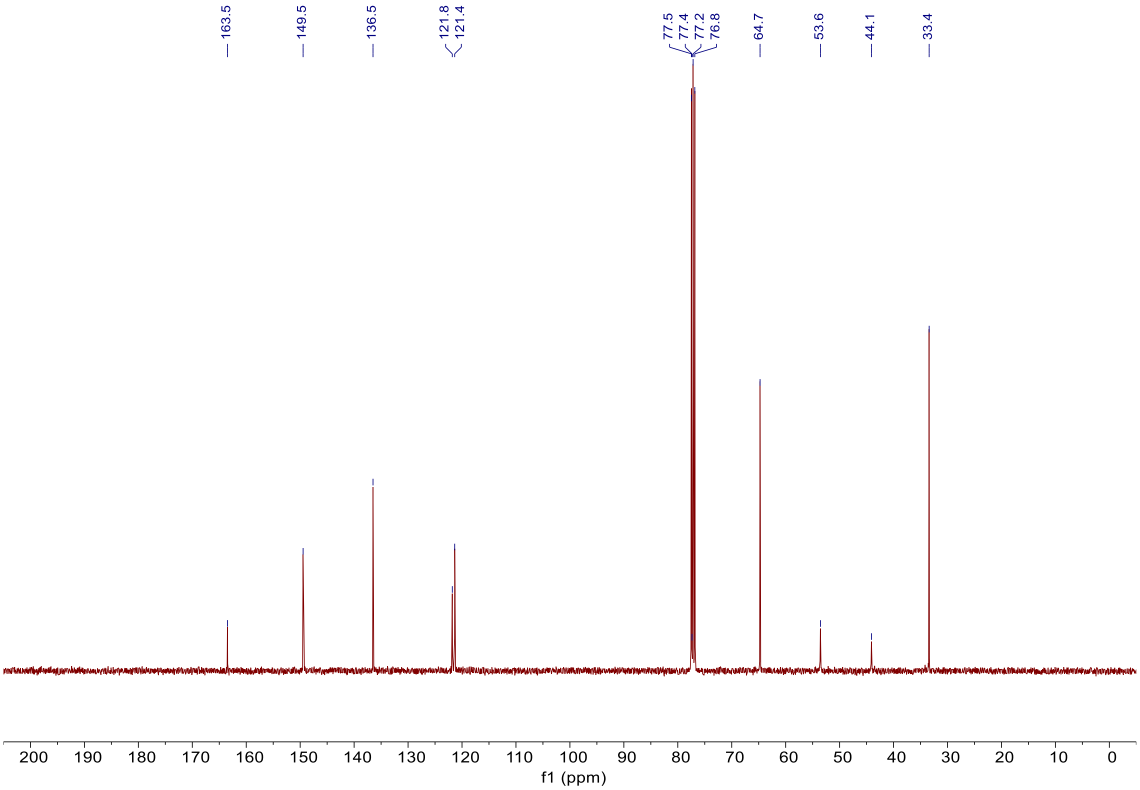

400 MHz ^{1}H NMR spectrum; 100.6 MHz ^{13}C NMR spectrum; $CDCl_3$ of **S17**

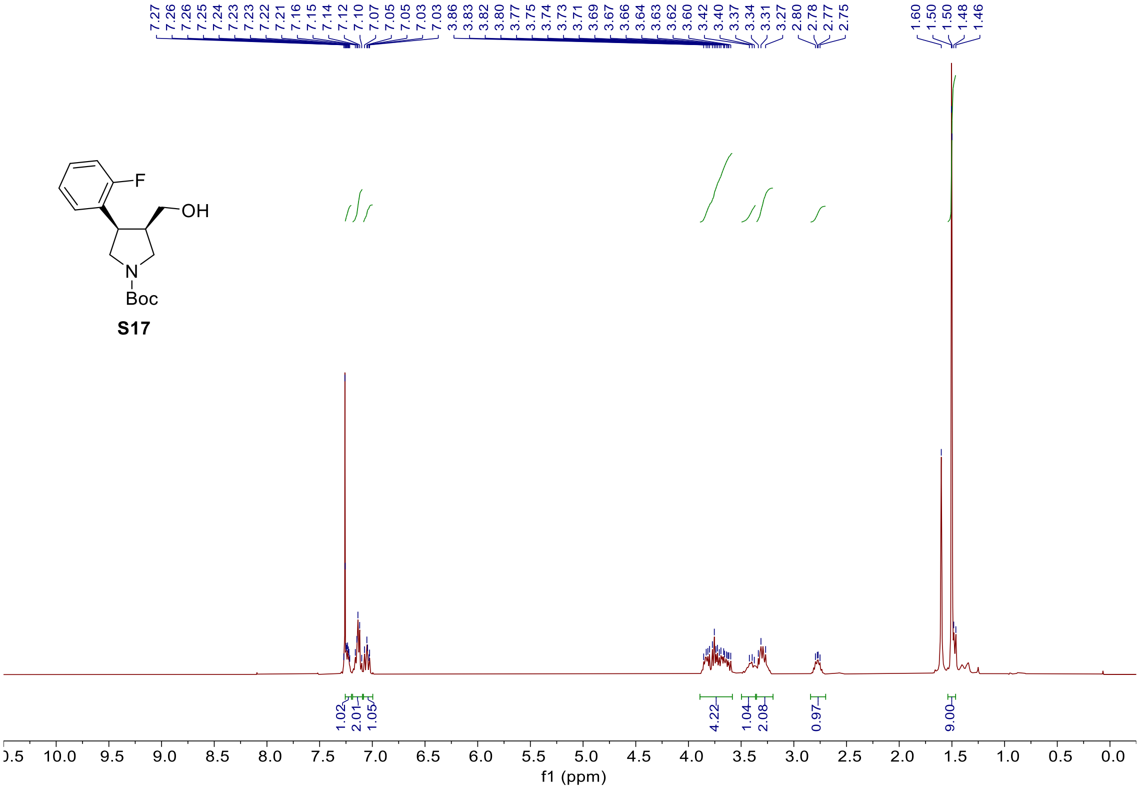

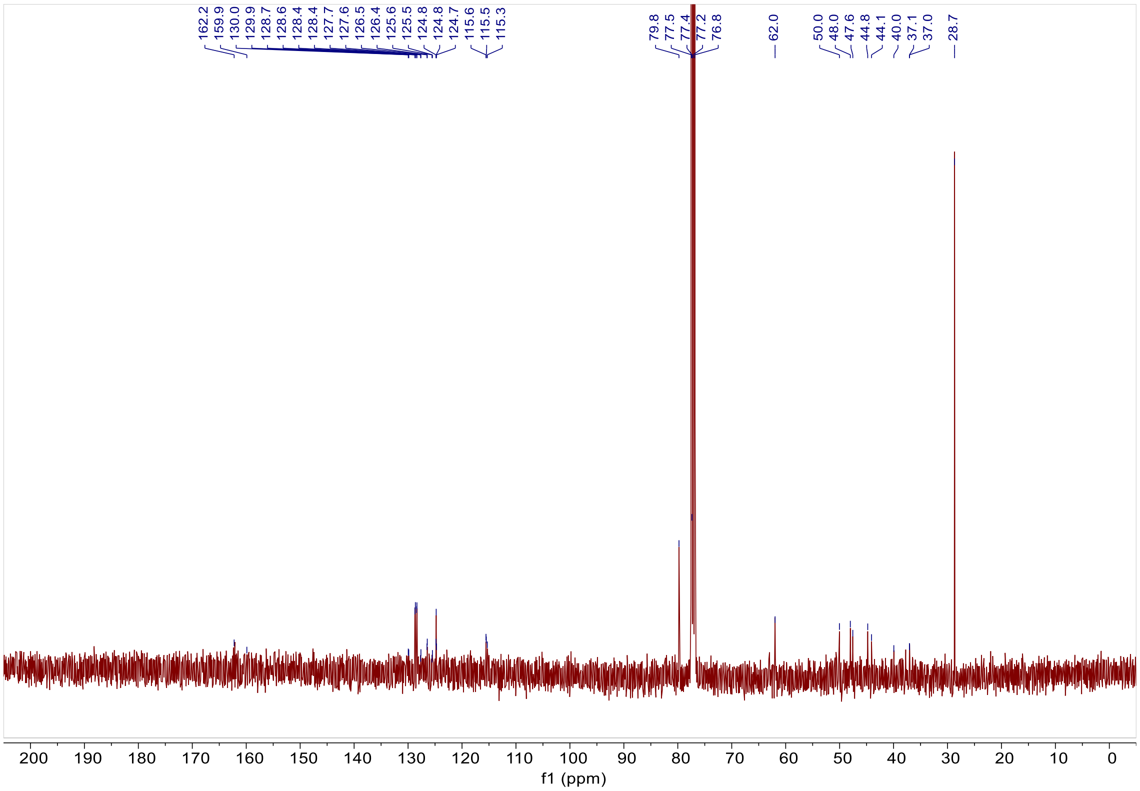

400 MHz ¹H NMR spectrum; 100.6 MHz ¹³C NMR spectrum; MeOD-d_4 of **8af**•HCl

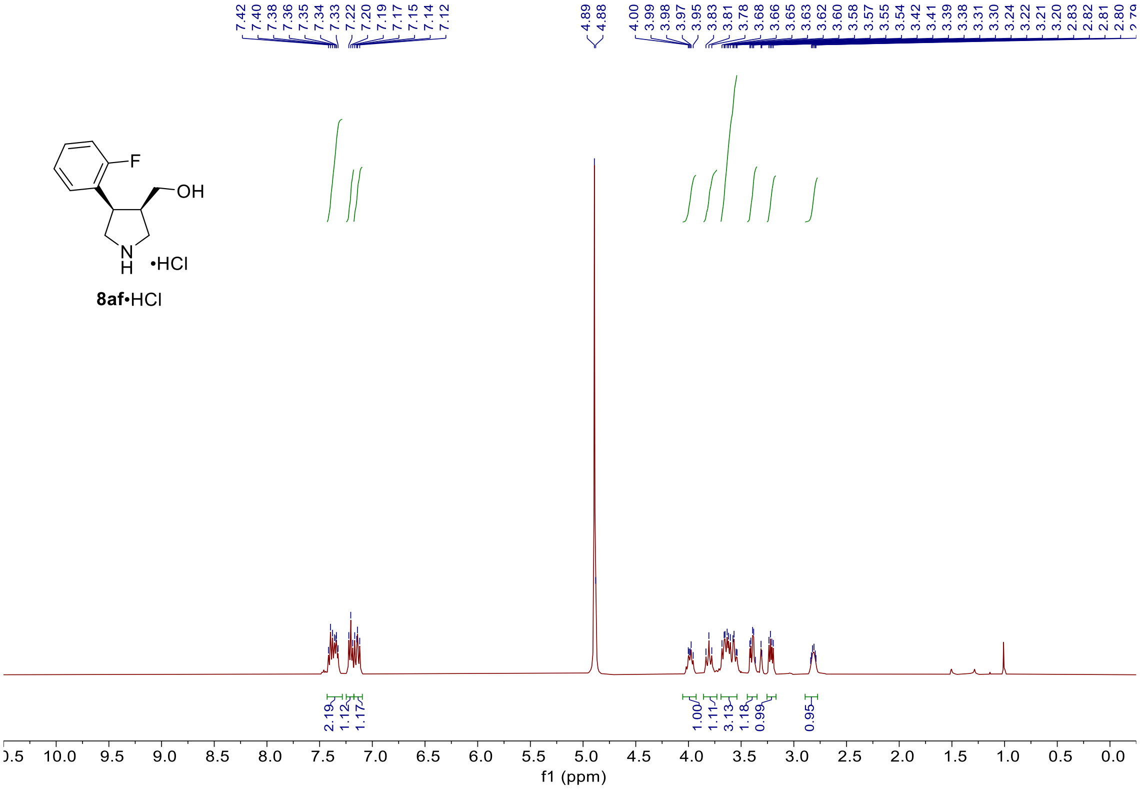

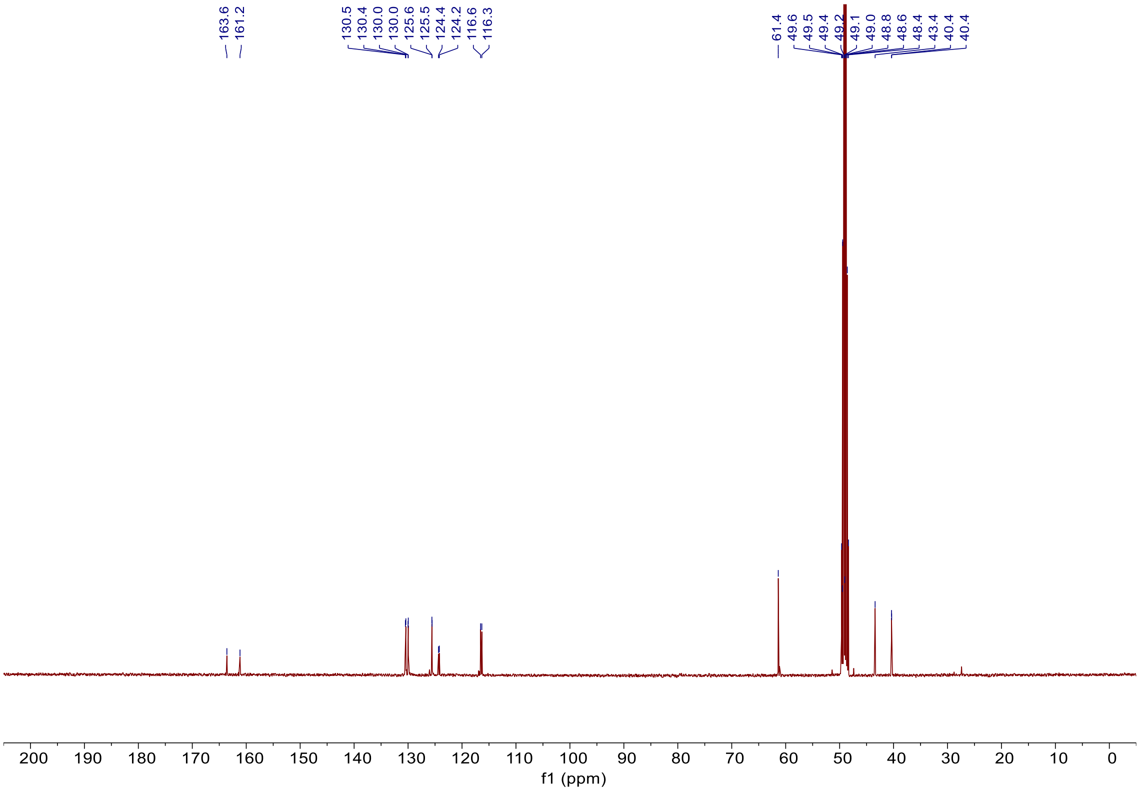

400 MHz ^{1}H NMR spectrum; 100.6 MHz ^{13}C NMR spectrum; MeOD-d_4 of **S18**

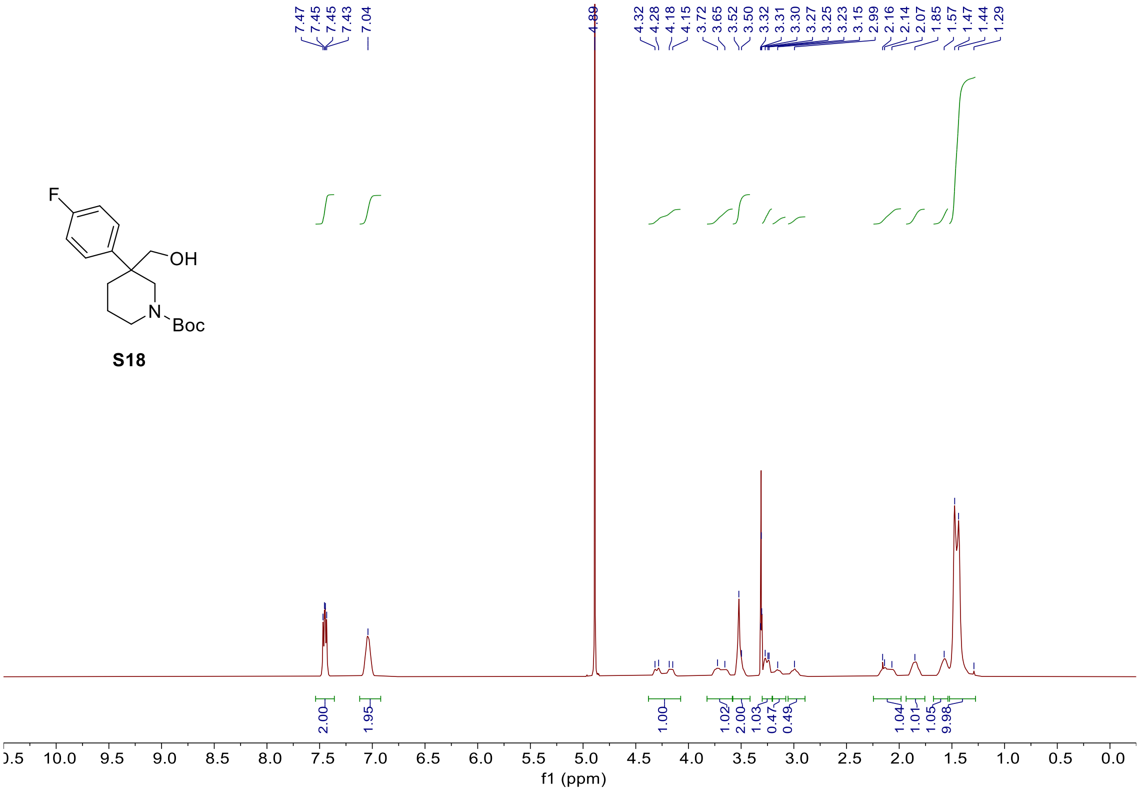

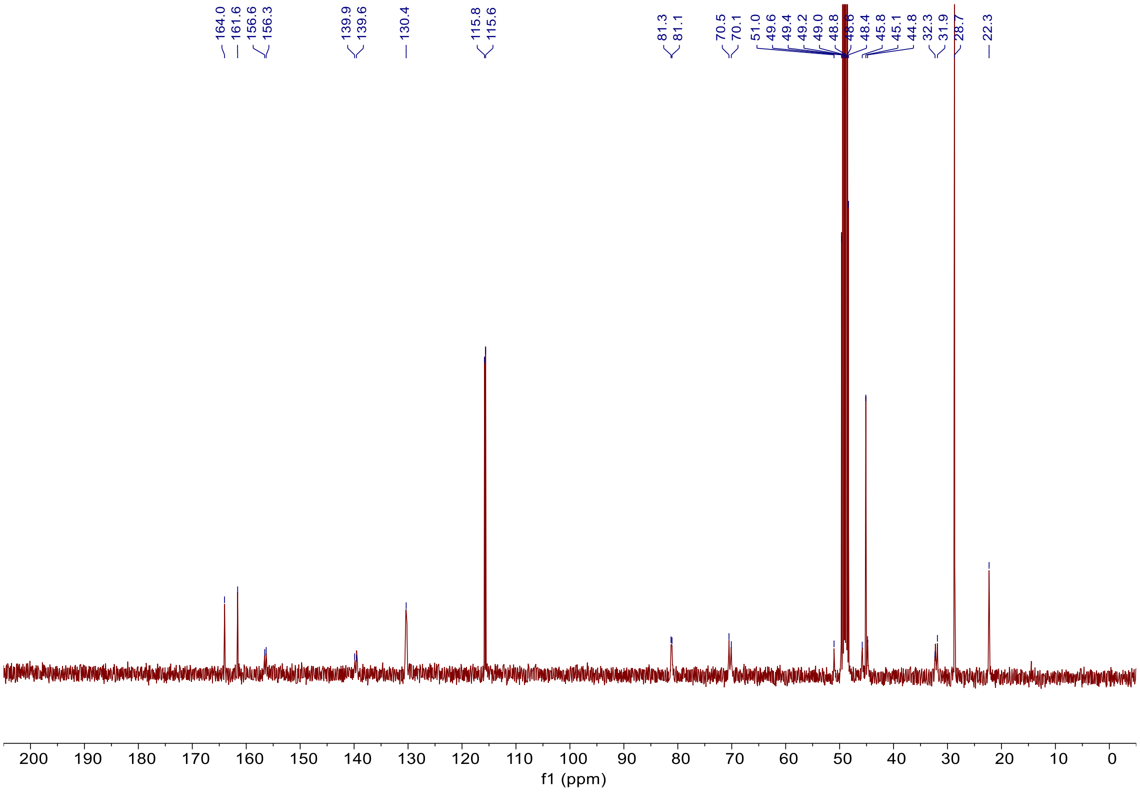

400 MHz ^{1}H NMR spectrum; 100.6 MHz ^{13}C NMR spectrum; $CDCl_3$ of **8ag**•HCl

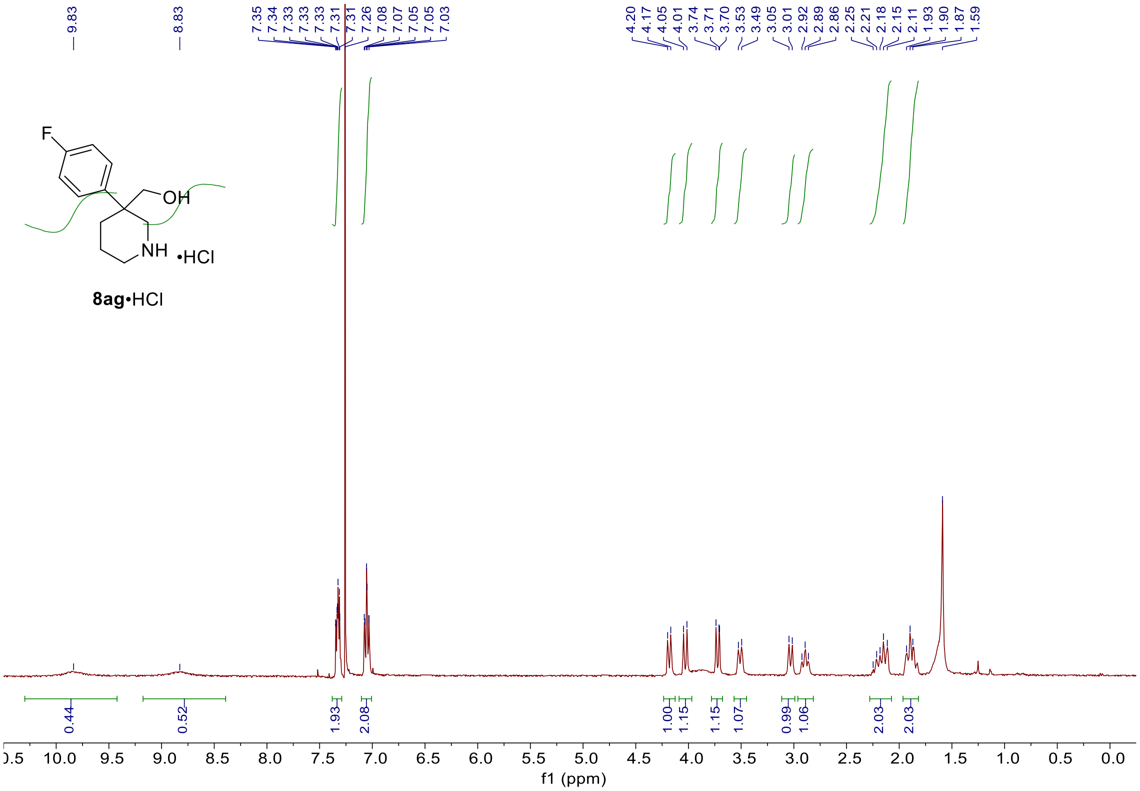

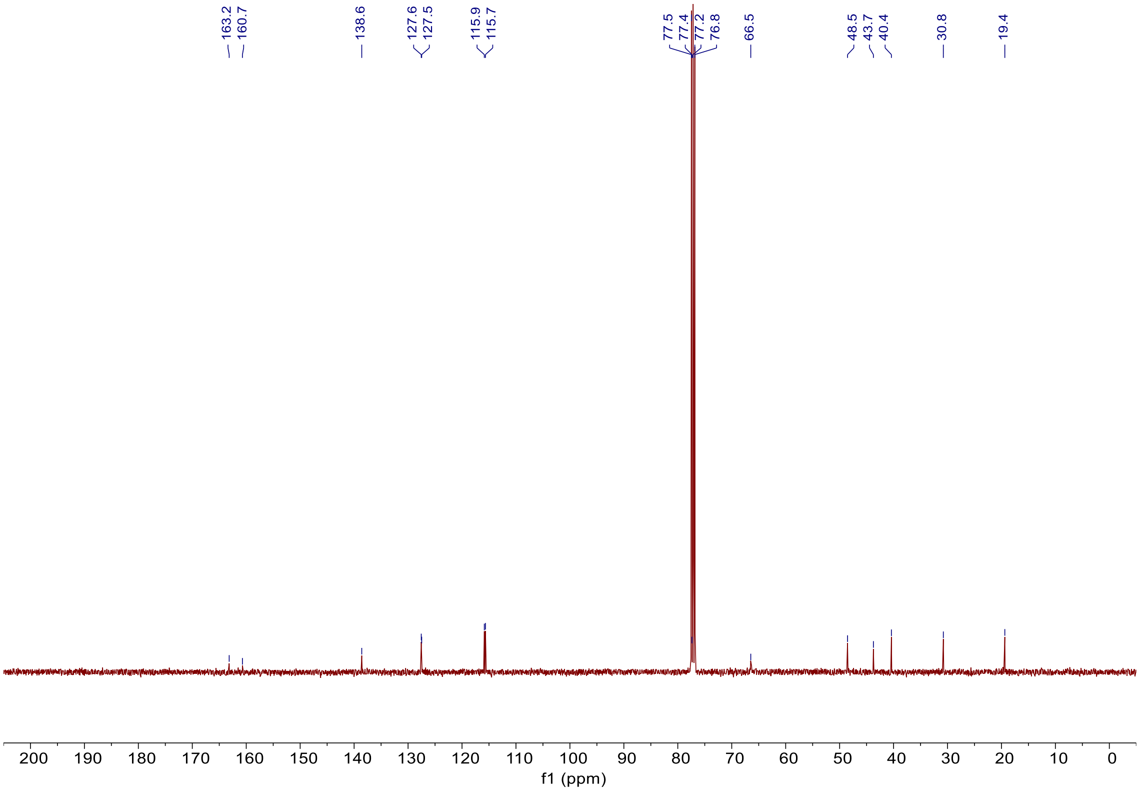

400 MHz ^{1}H NMR spectrum; 100.6 MHz ^{13}C NMR spectrum; $CDCl_3$ of **S19**

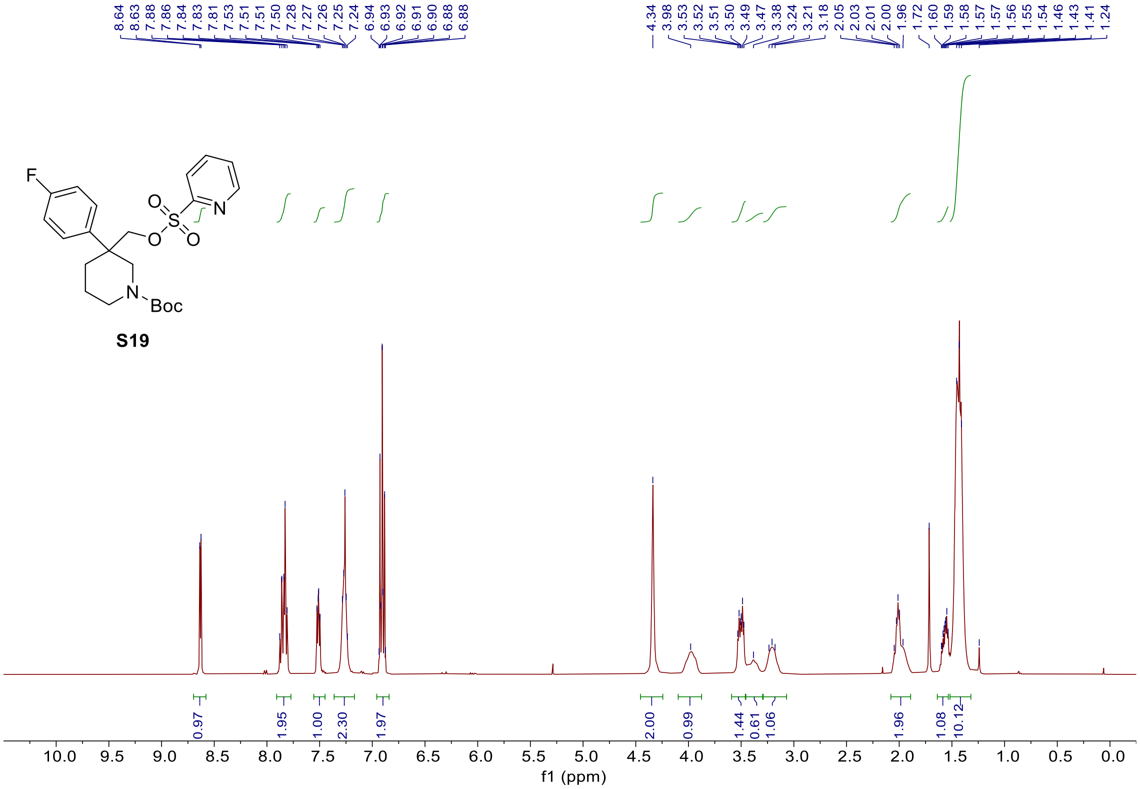

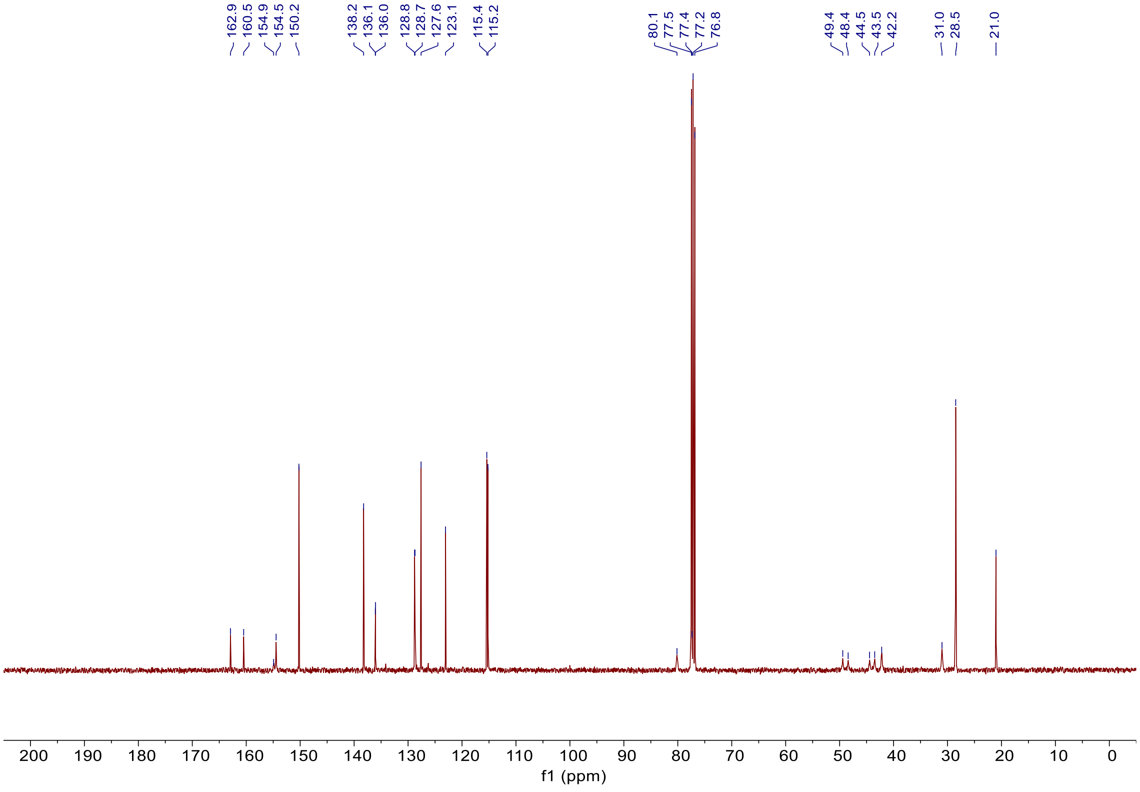

400 MHz ^{1}H NMR spectrum; 100.6 MHz ^{13}C NMR spectrum; $CDCl_3$ of **S20**

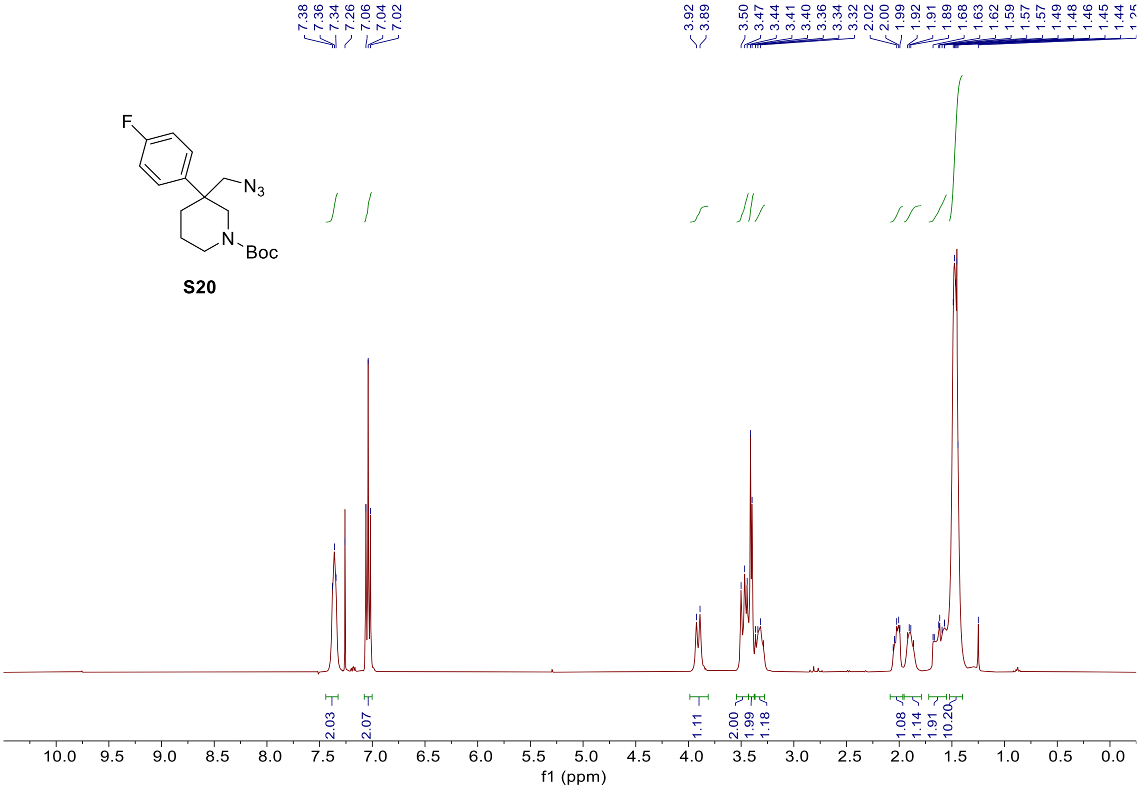

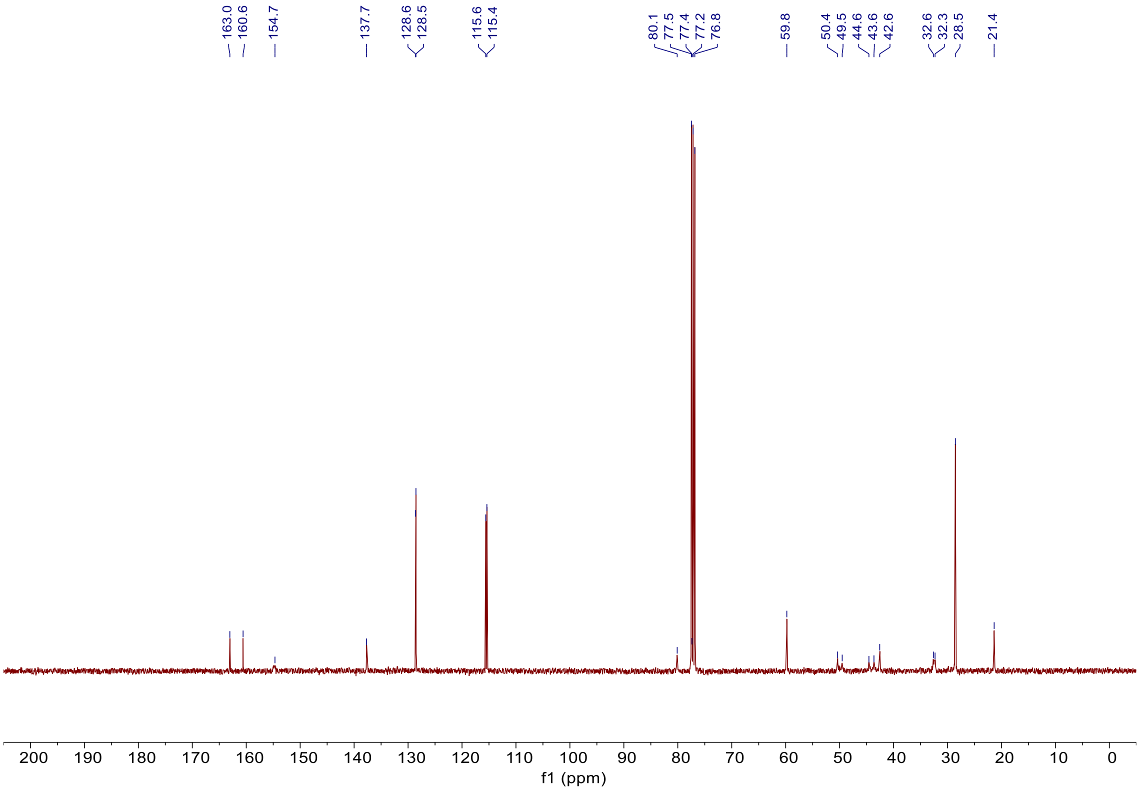

400 MHz ^{1}H NMR spectrum; 100.6 MHz ^{13}C NMR spectrum; $CDCl_3$ of **S21**

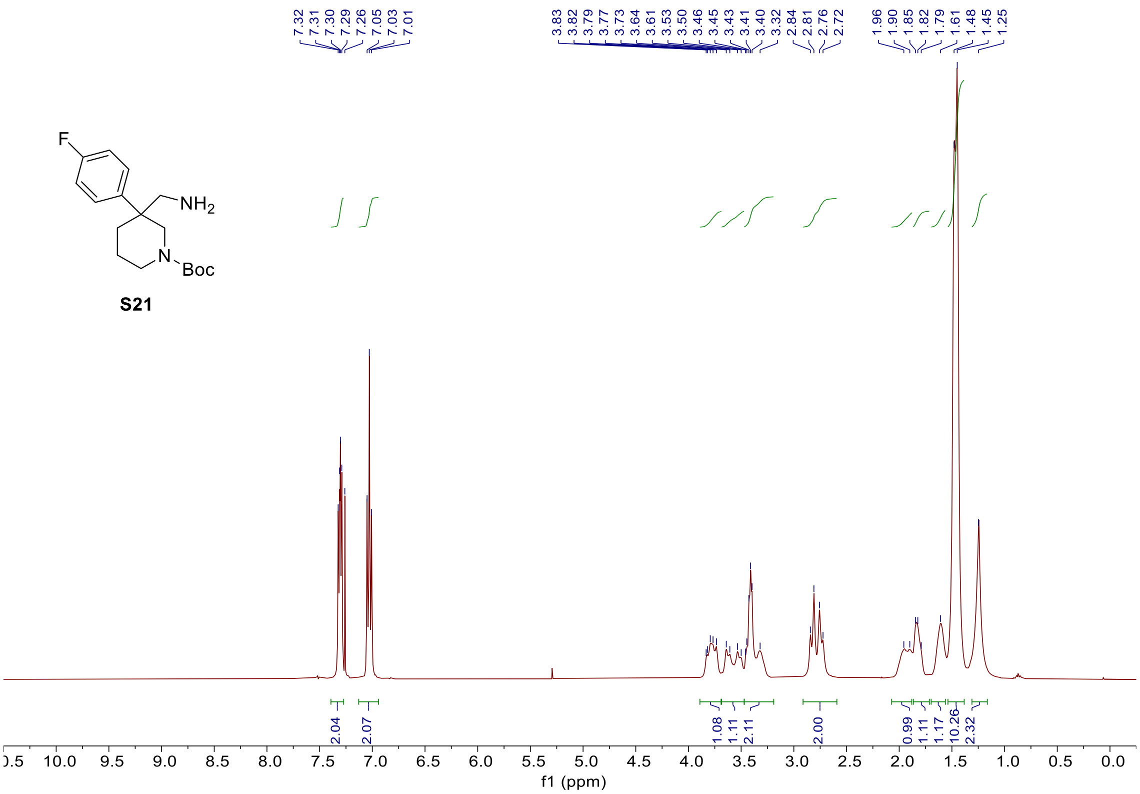

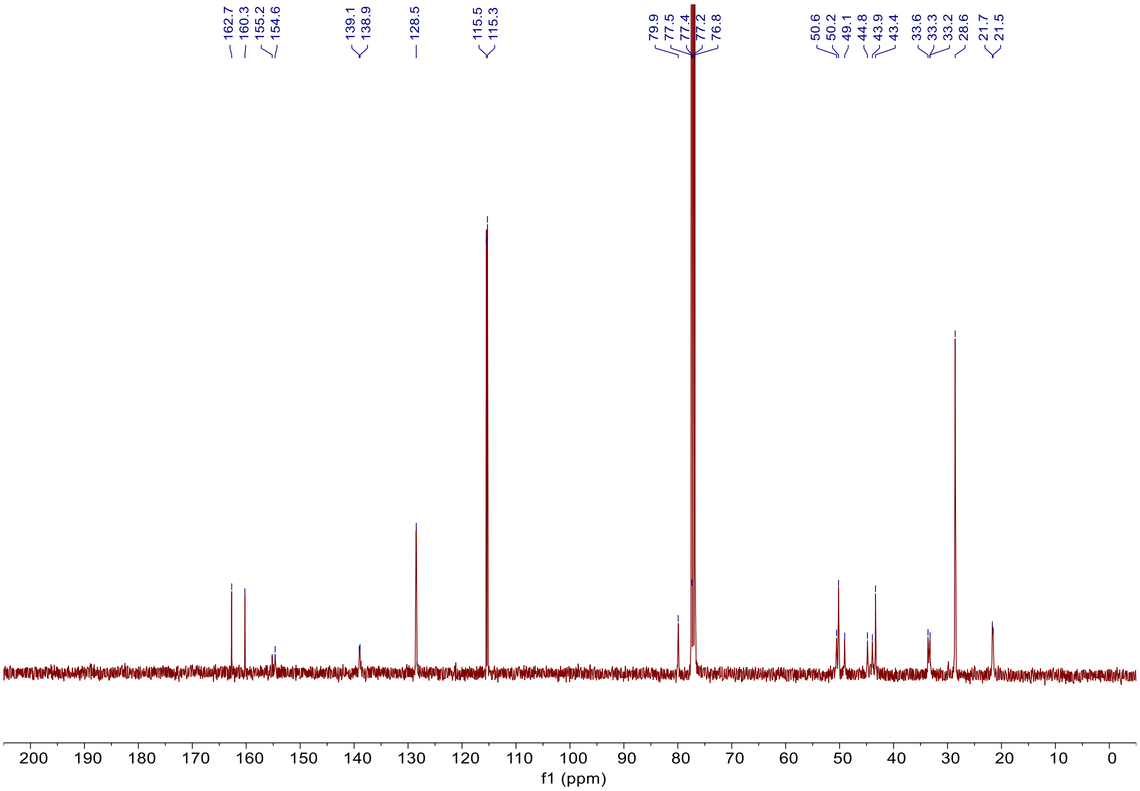

400 MHz ^{1}H NMR spectrum; 100.6 MHz ^{13}C NMR spectrum; MeOD-d_4 of **8ah**•2HCl

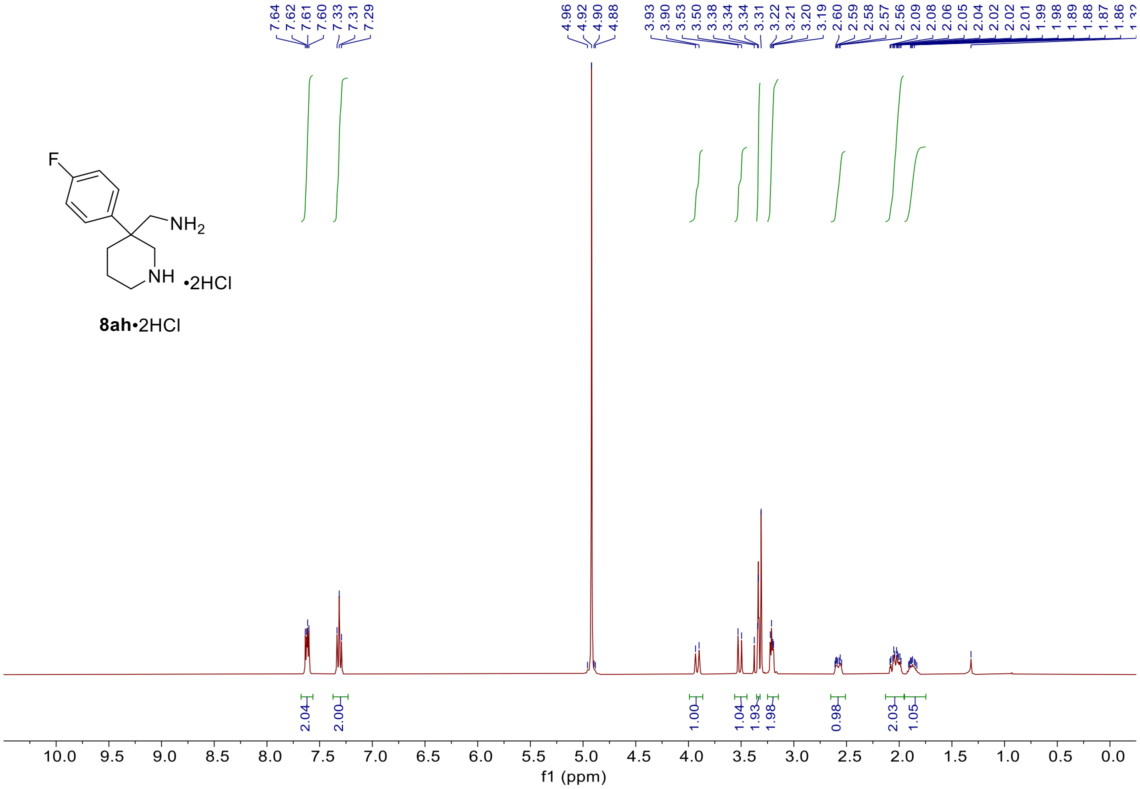

8ah•2HCl

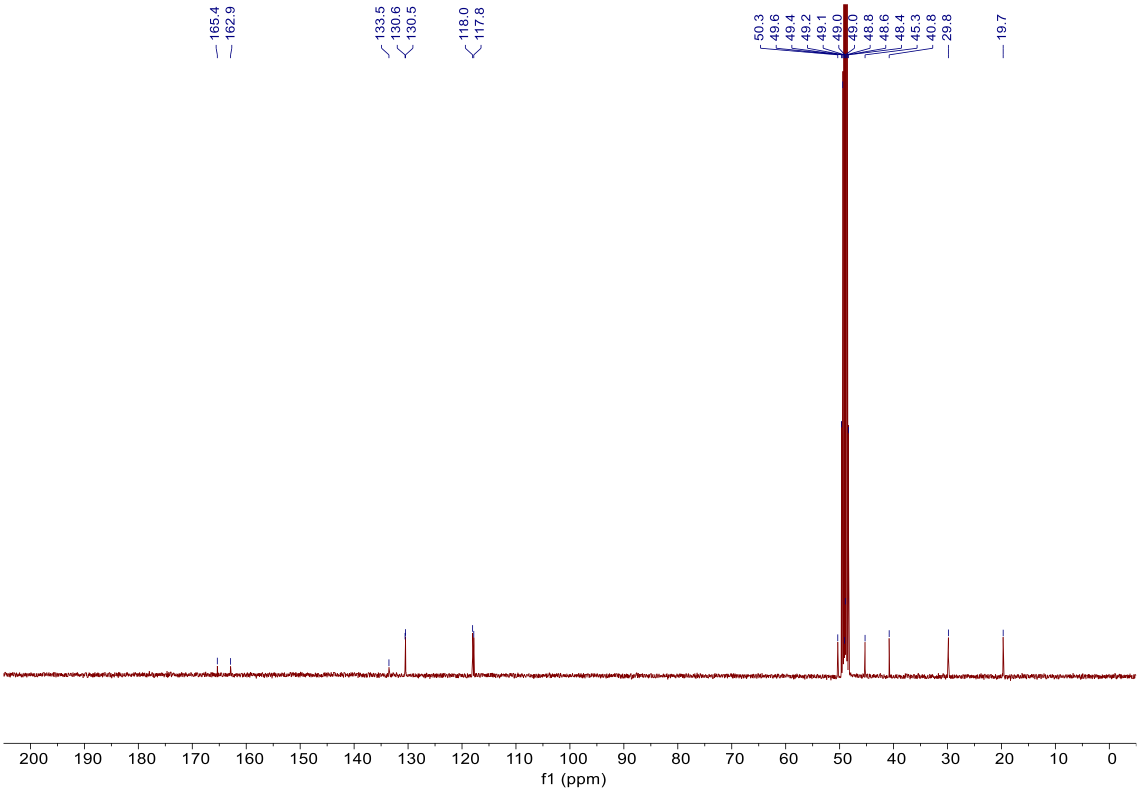

400 MHz ^{1}H NMR spectrum; 100.6 MHz ^{13}C NMR spectrum; $CDCl_3$ of **8ai**

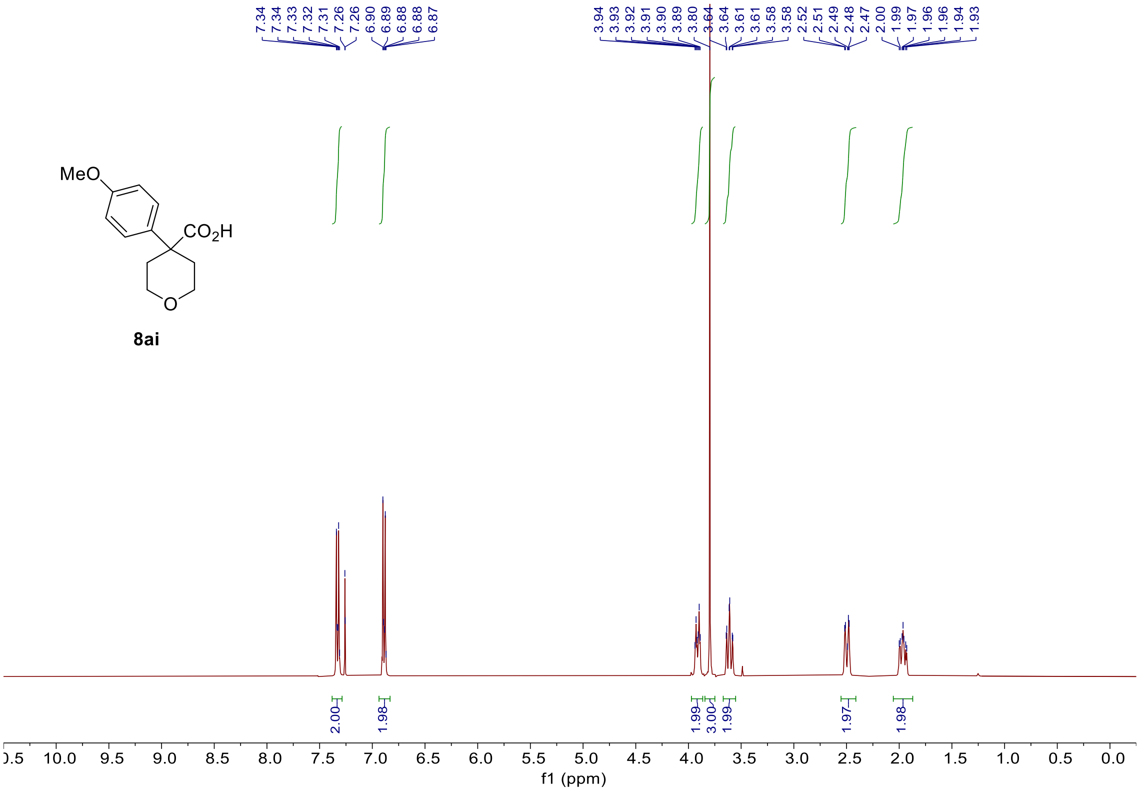

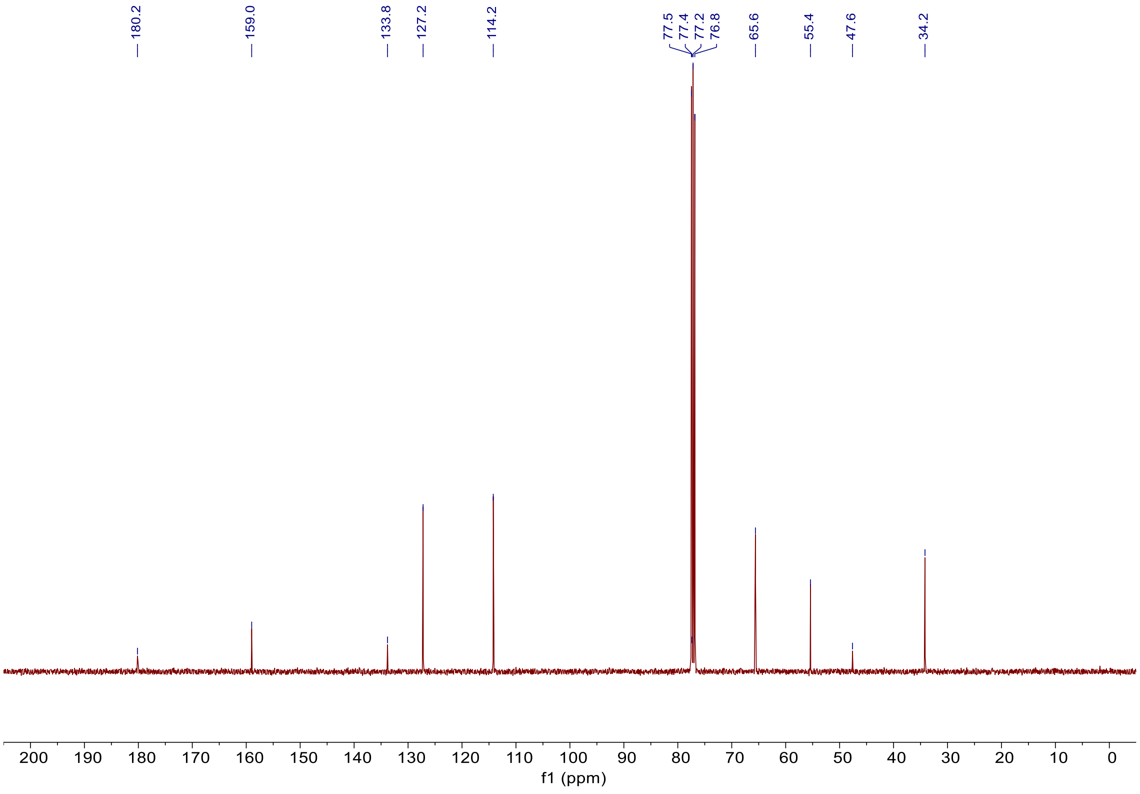

400 MHz ^{1}H NMR spectrum; 100.6 MHz ^{13}C NMR spectrum; $CDCl_3$ of **S22**

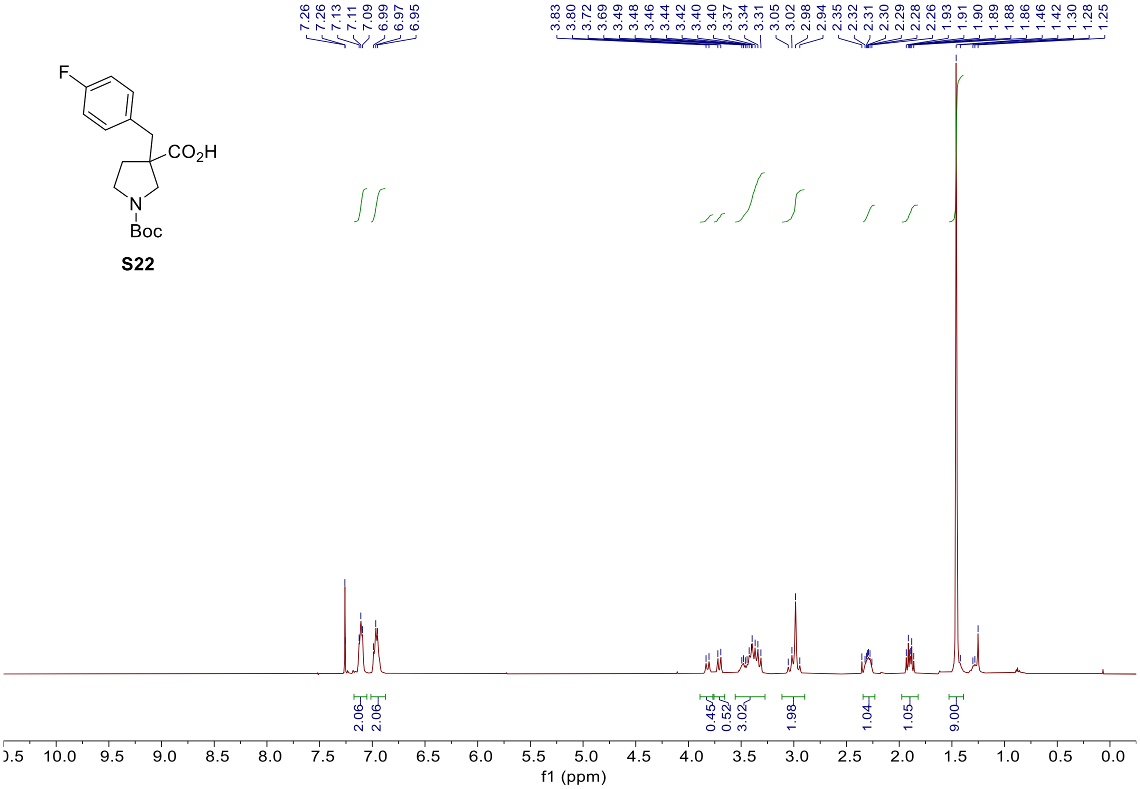

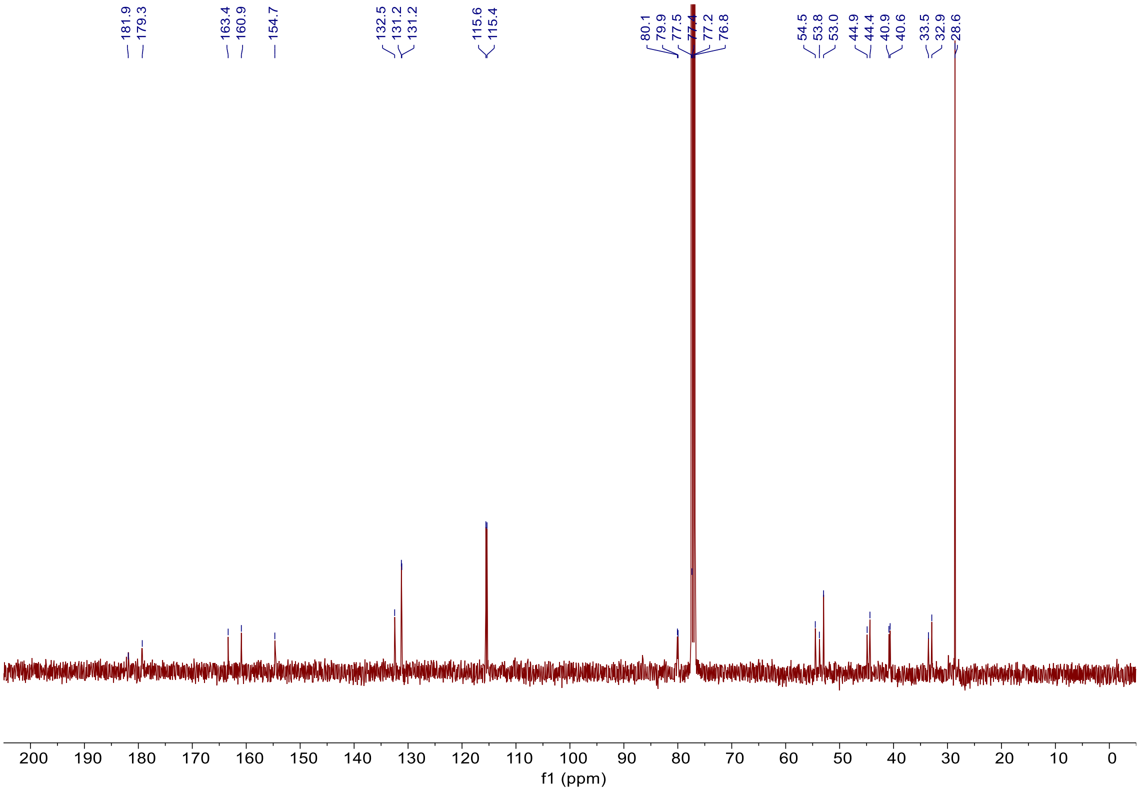

400 MHz ^{1}H NMR spectrum; 100.6 MHz ^{13}C NMR spectrum; MeOD-d_4 of **8aj**•HCl

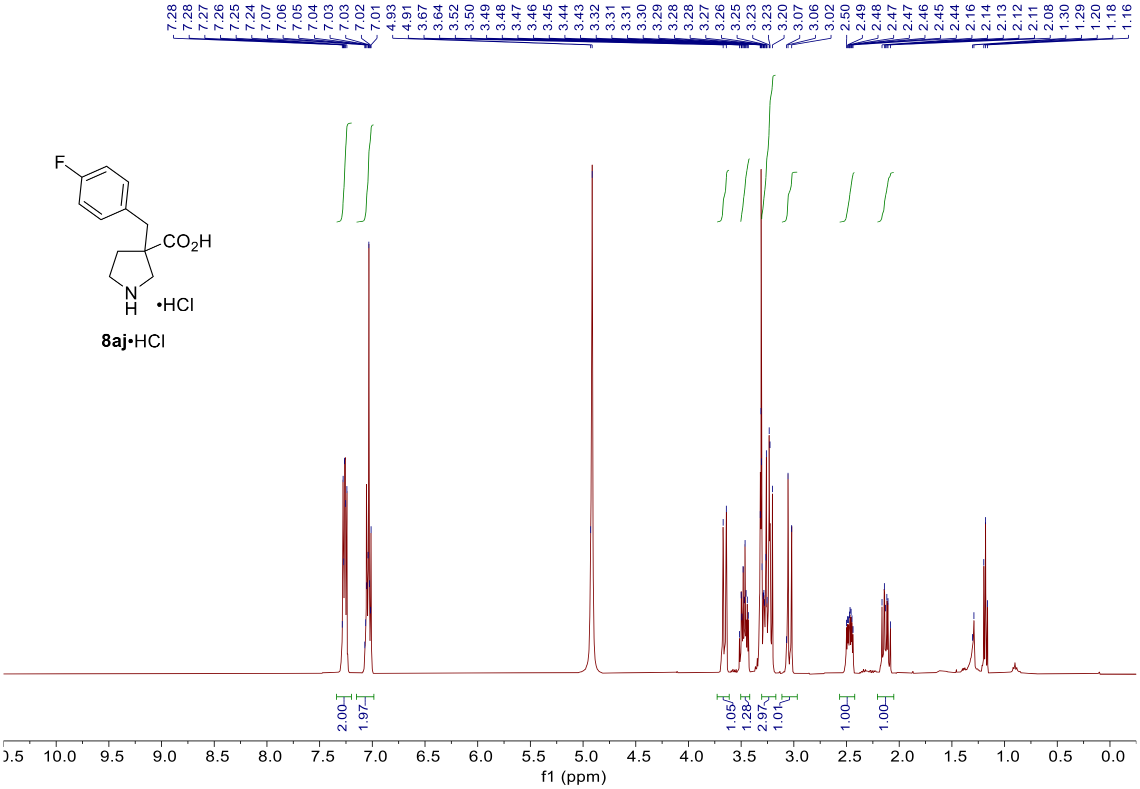

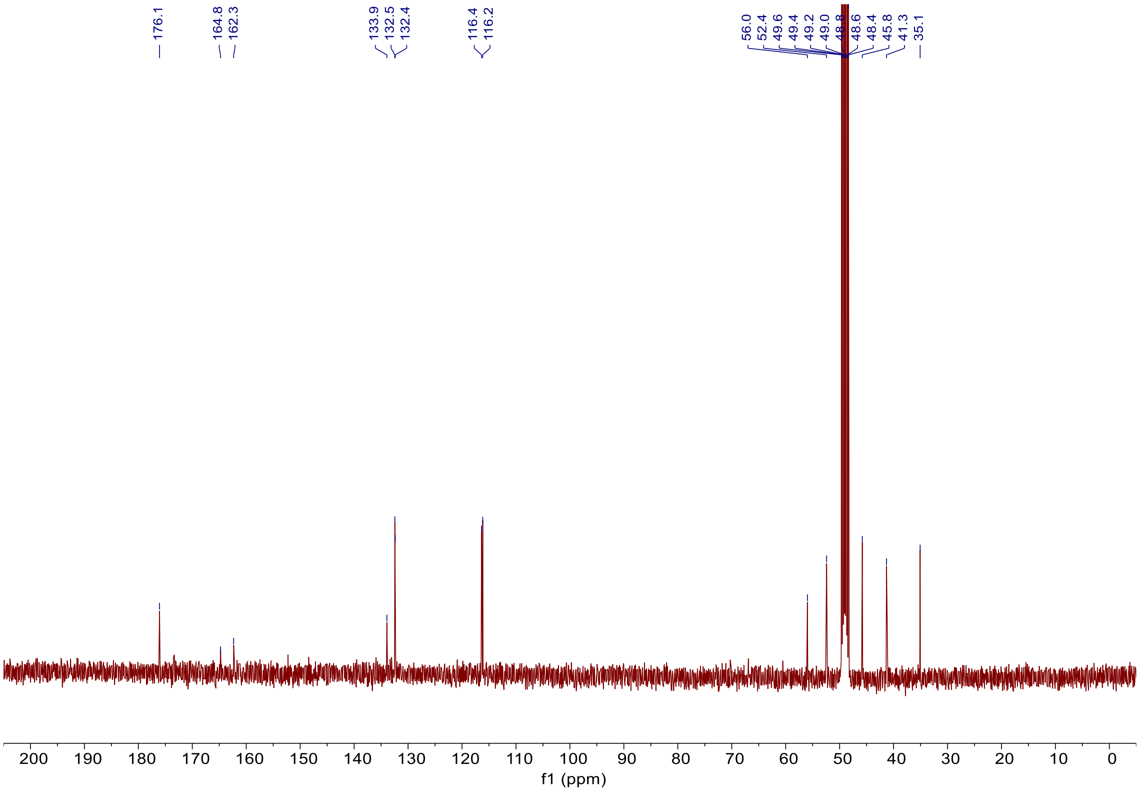

400 MHz ^{1}H NMR spectrum; 100.6 MHz ^{13}C NMR spectrum; $CDCl_3$ of **S23**

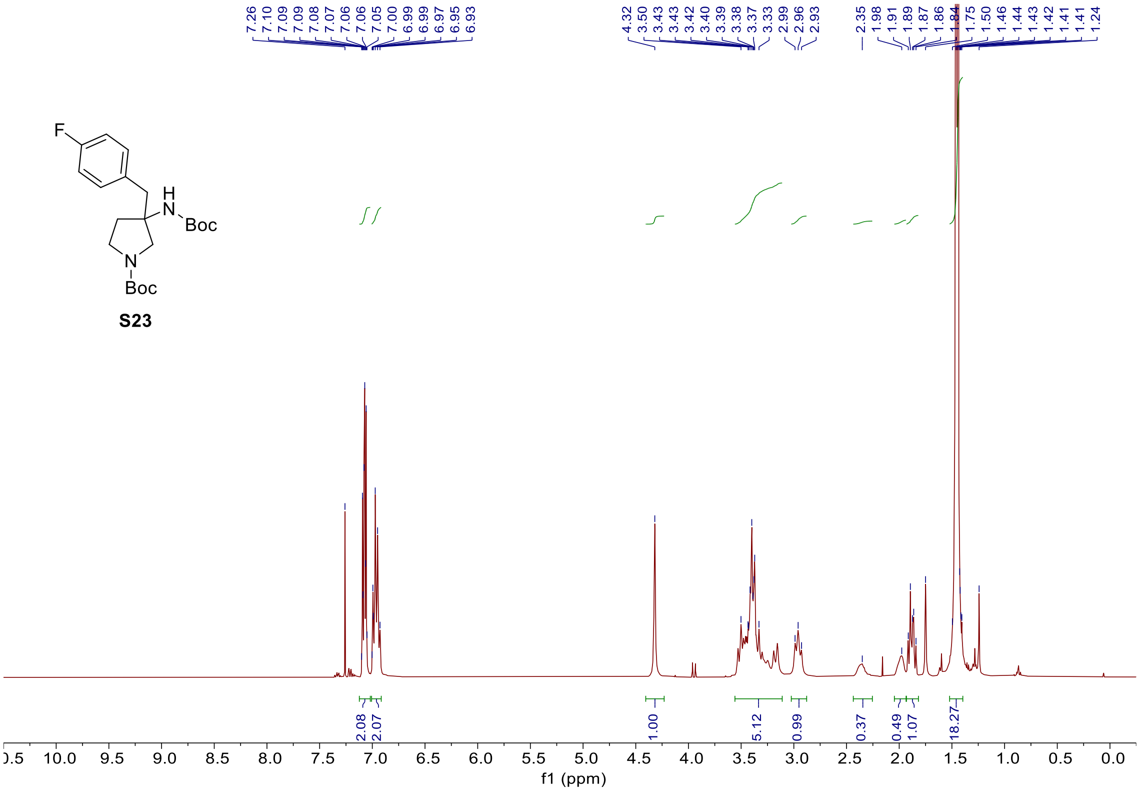

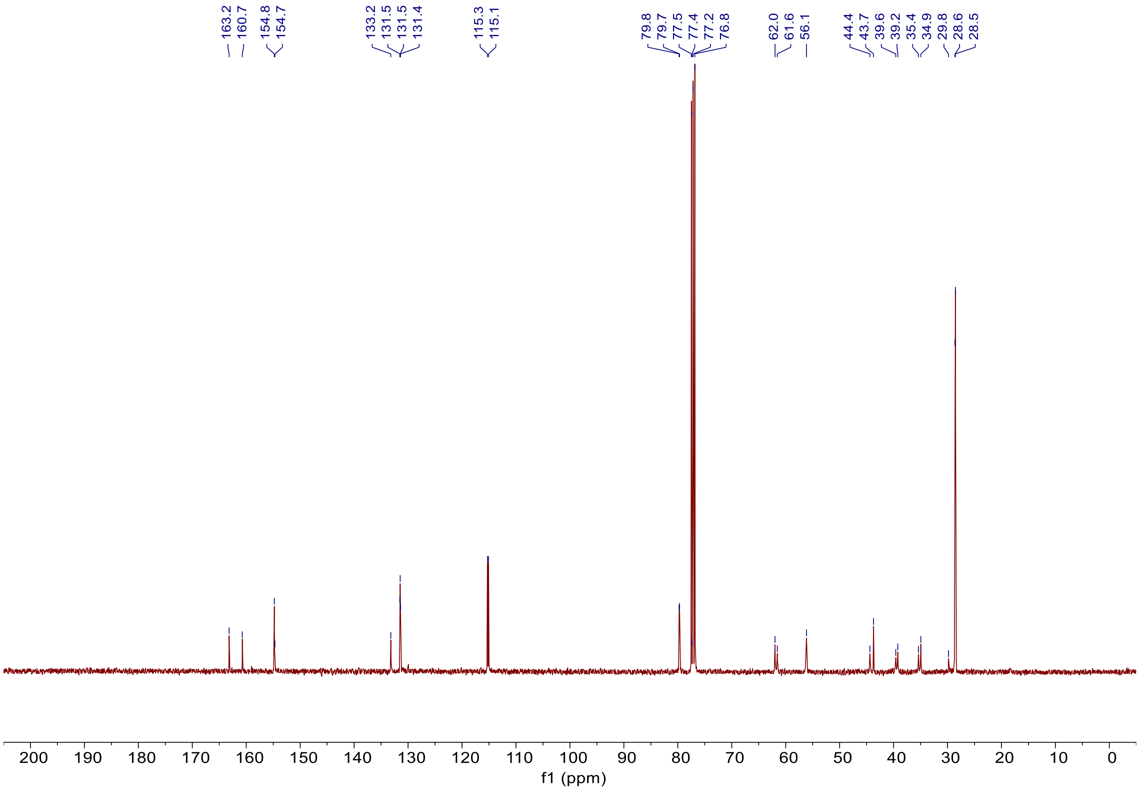

400 MHz ^{1}H NMR spectrum; 100.6 MHz ^{13}C NMR spectrum; MeOD-d_4 of **8ak**•2HCl

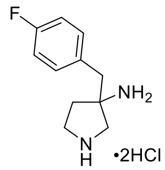

8ak•2HCl

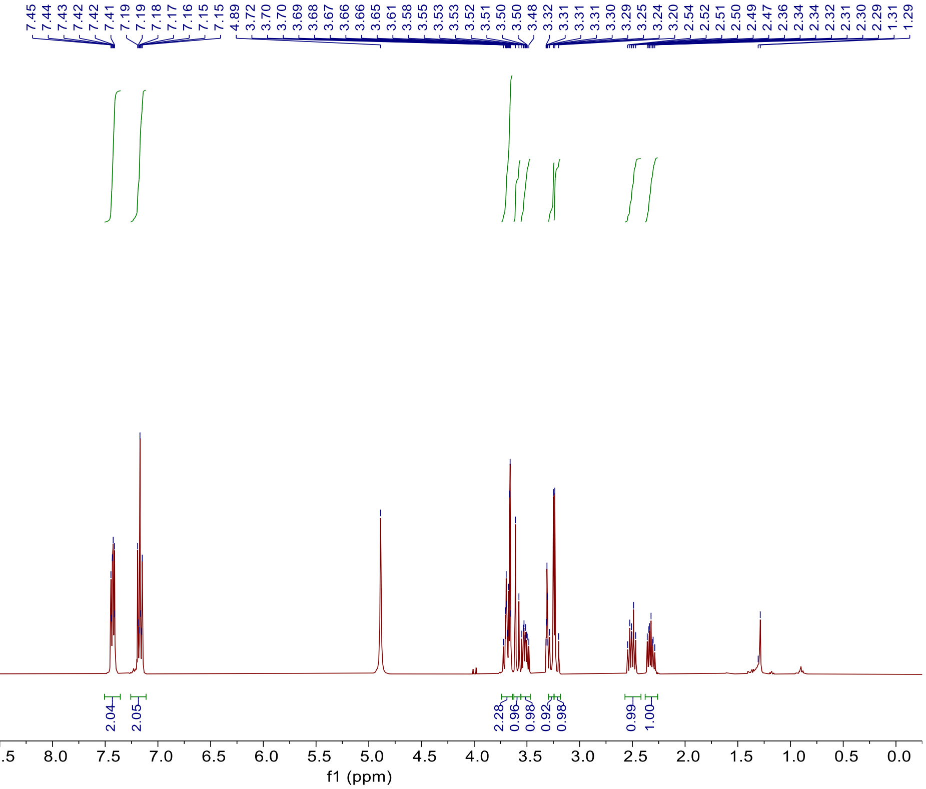

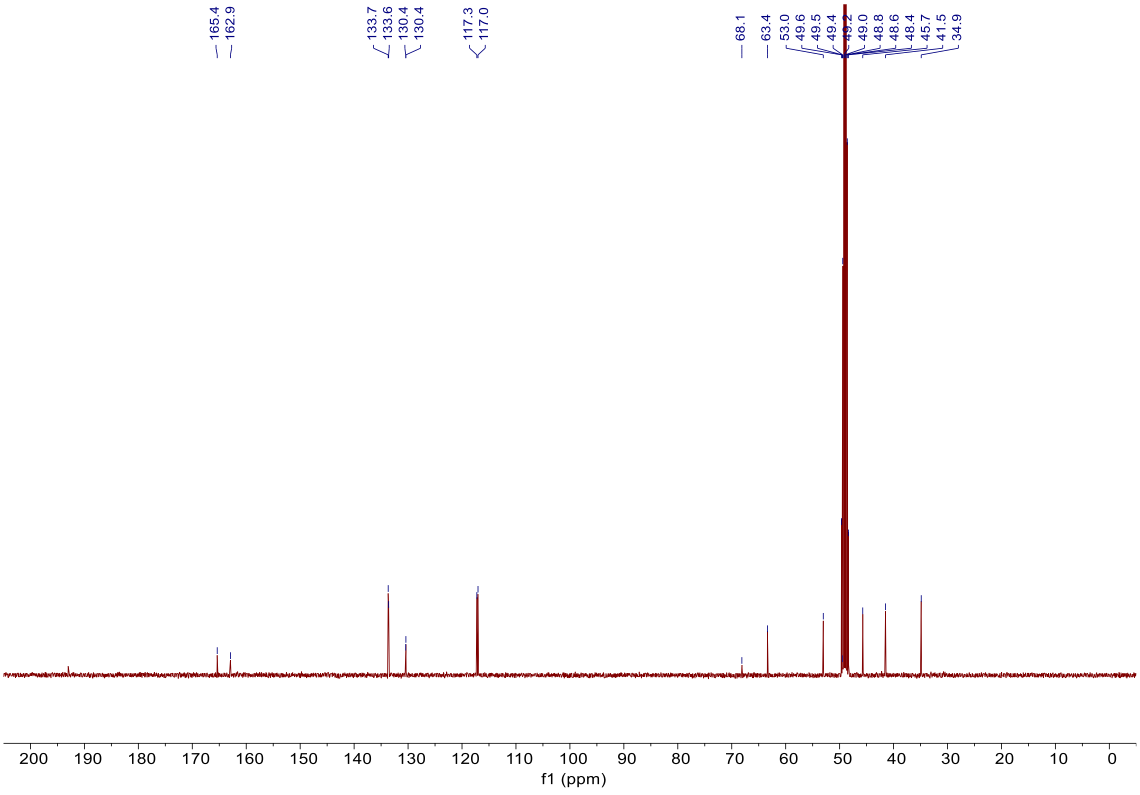

400 MHz ^{1}H NMR spectrum; 100.6 MHz ^{13}C NMR spectrum; $CDCl_3$ of **S24**

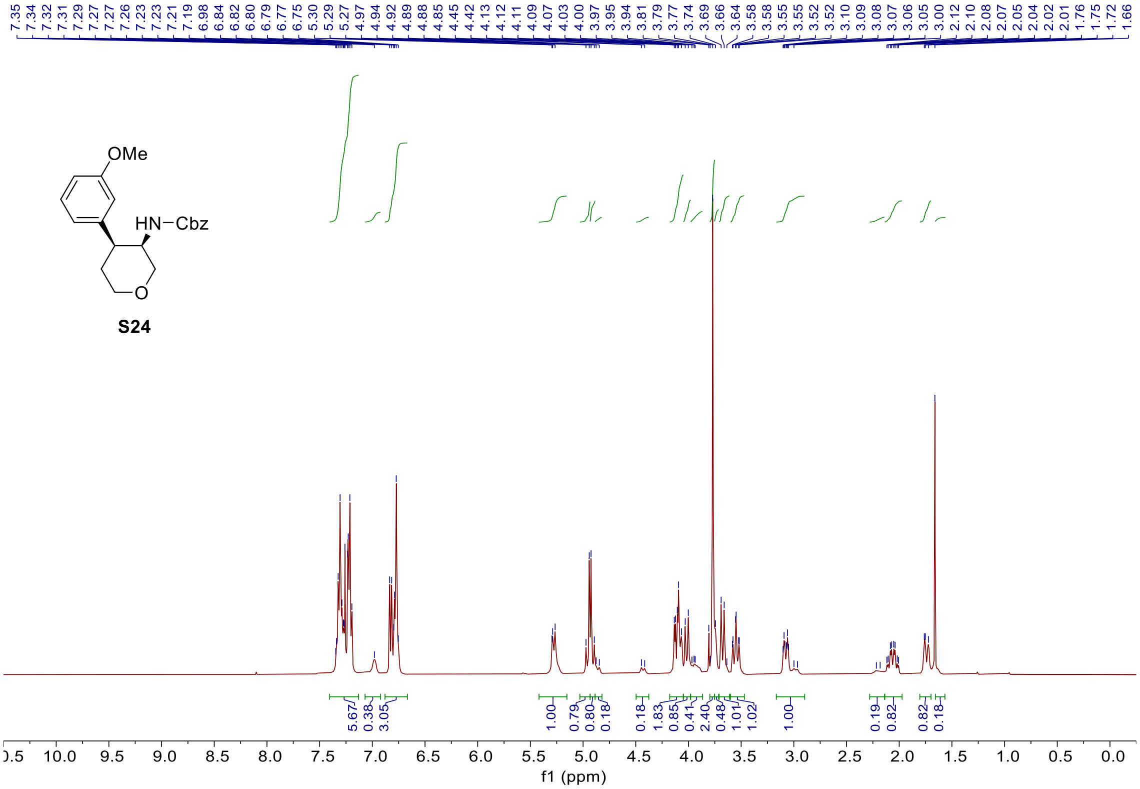

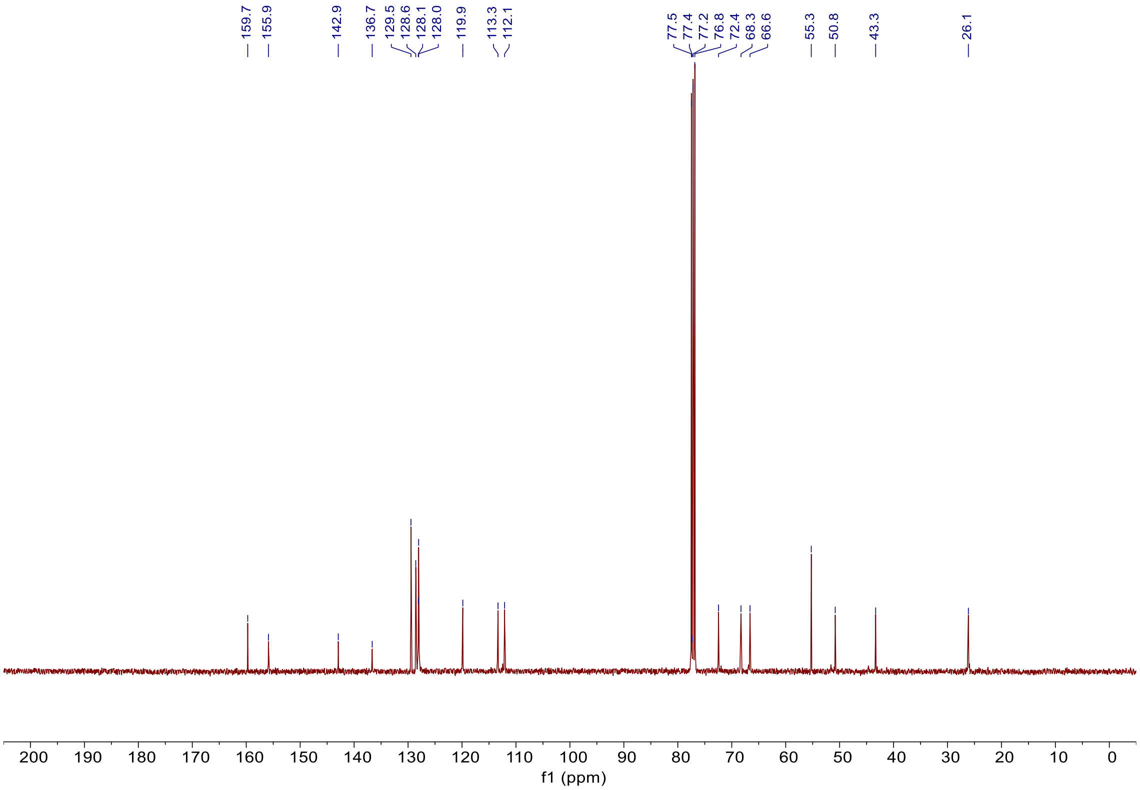

400 MHz ^{1}H NMR spectrum; 100.6 MHz ^{13}C NMR spectrum; $CDCl_3$ of **8al**

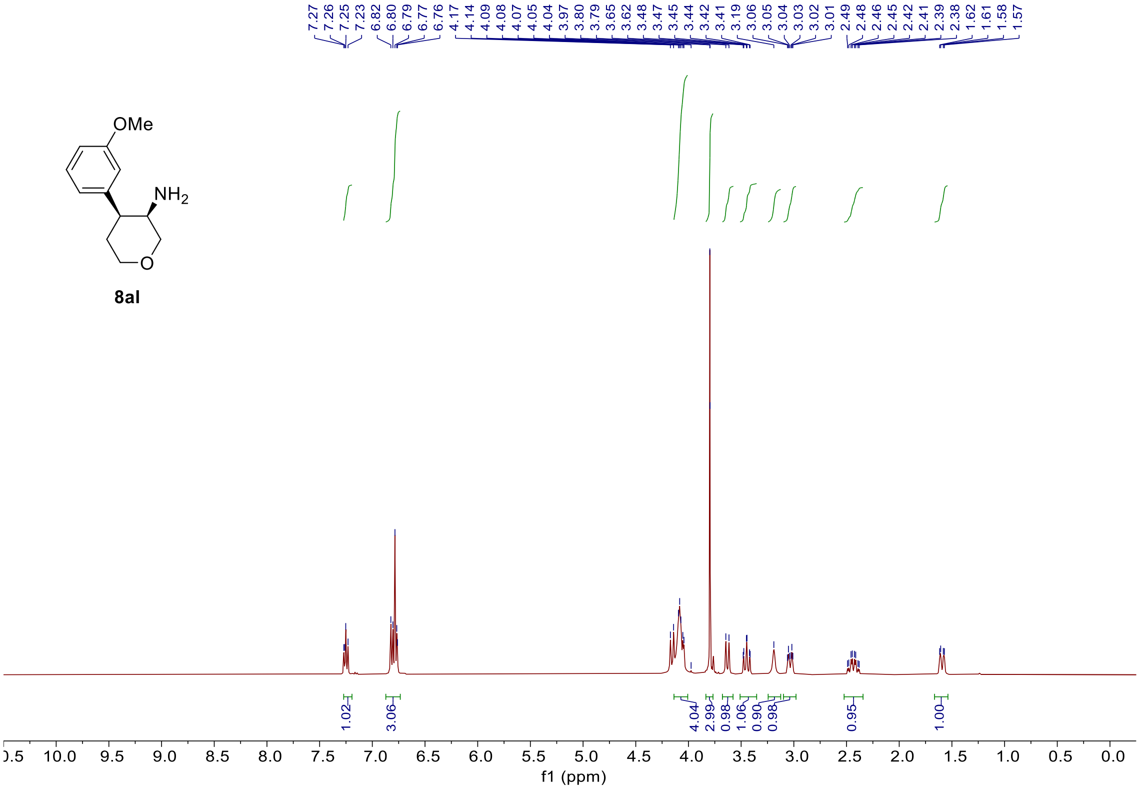

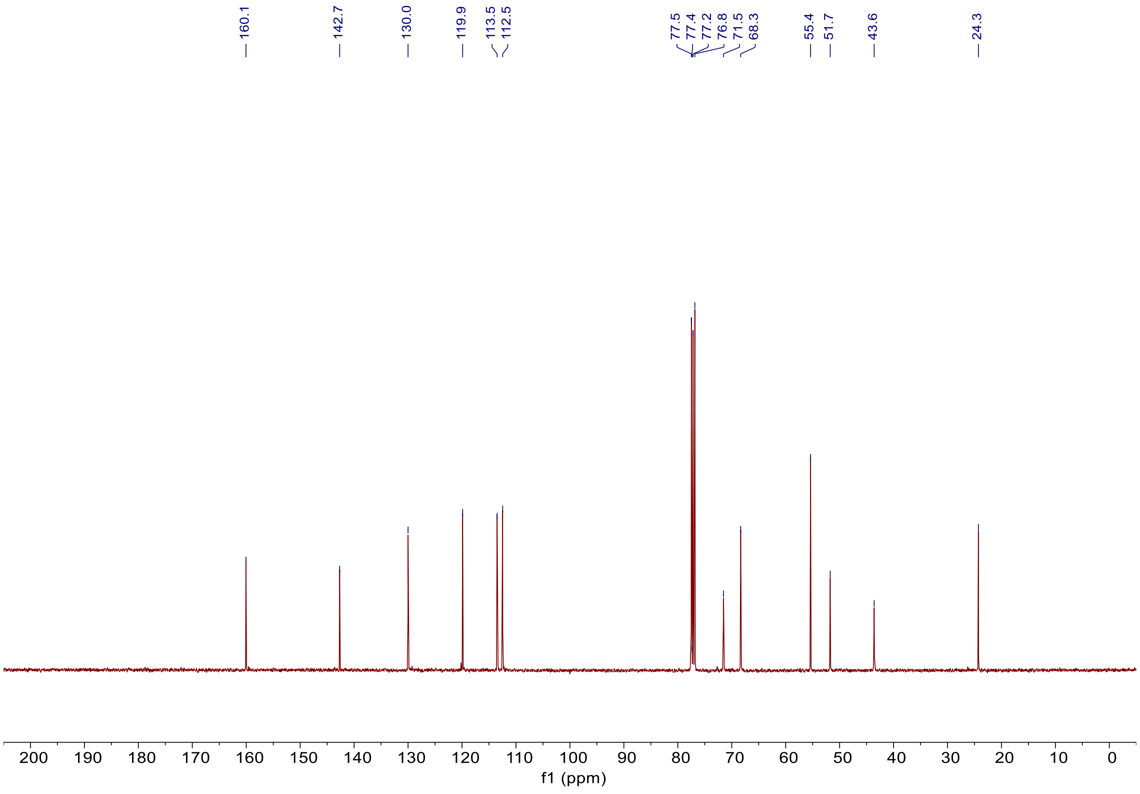

400 MHz ^{1}H NMR spectrum; 100.6 MHz ^{13}C NMR spectrum; $CDCl_3$ of **S25**

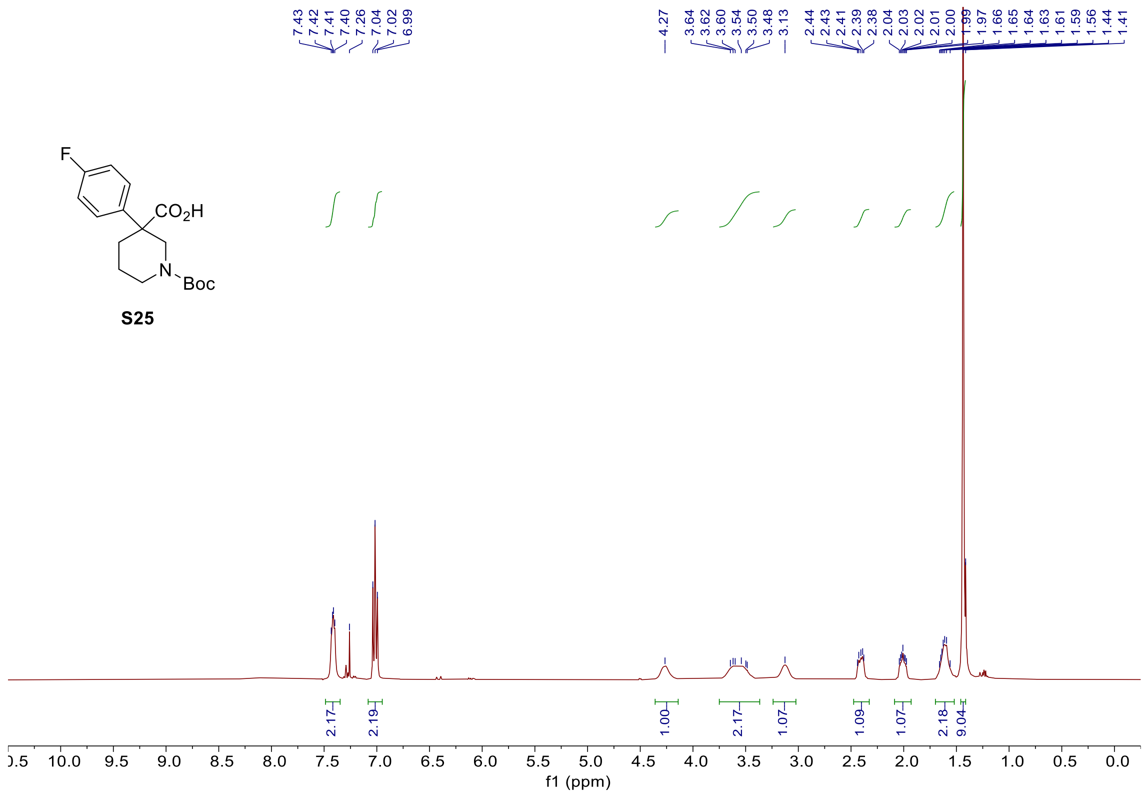

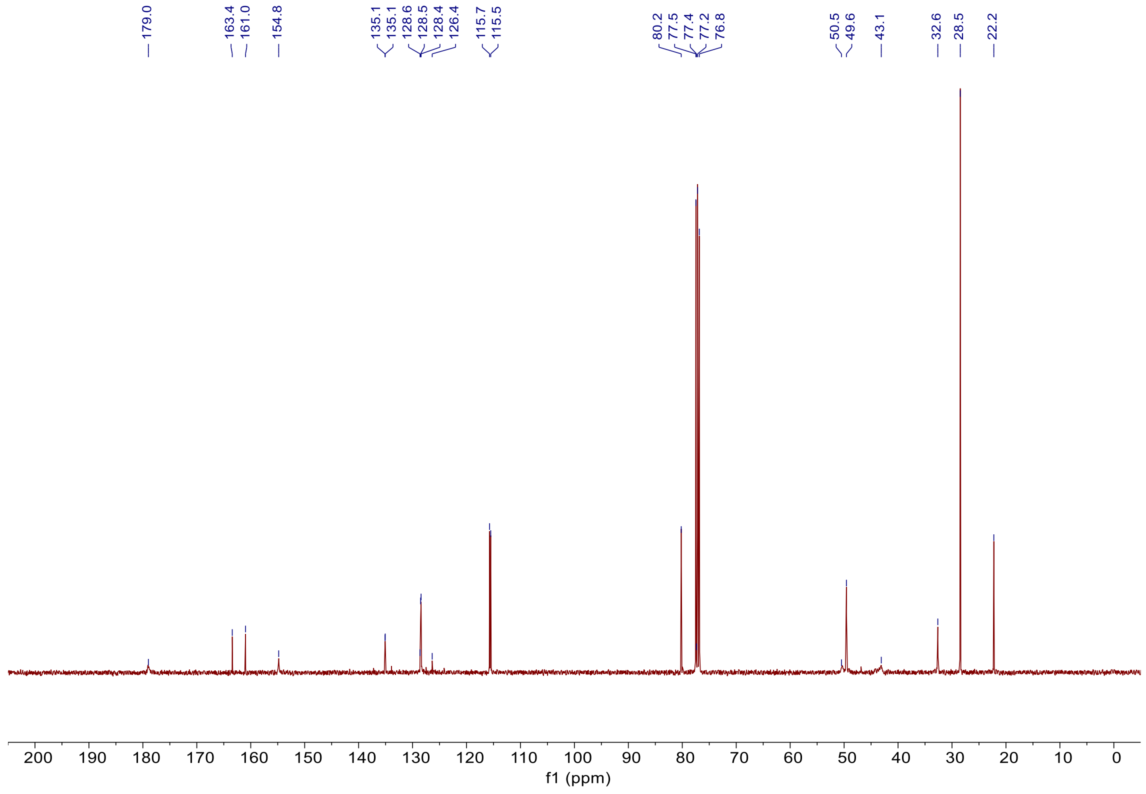

400 MHz ^{1}H NMR spectrum; 100.6 MHz ^{13}C NMR spectrum; $CDCl_3$ of **S26**

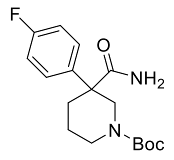

S26

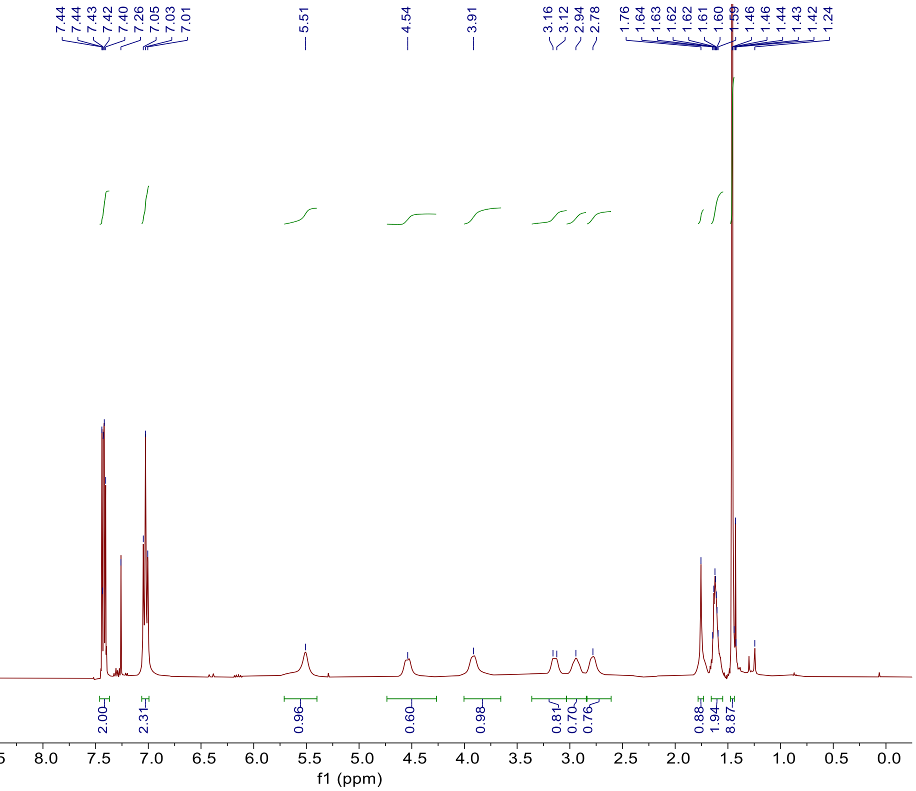

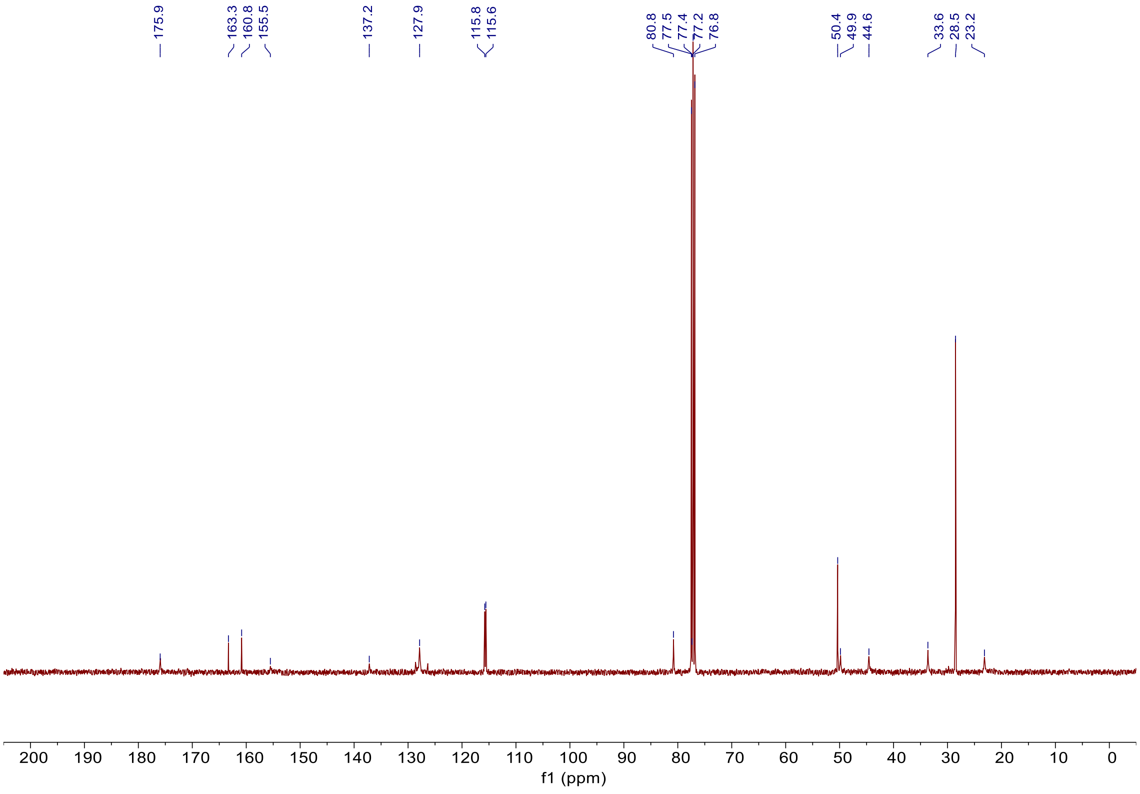

400 MHz ^{1}H NMR spectrum; 100.6 MHz ^{13}C NMR spectrum; MeOD-d_4 of **8am**•HCl

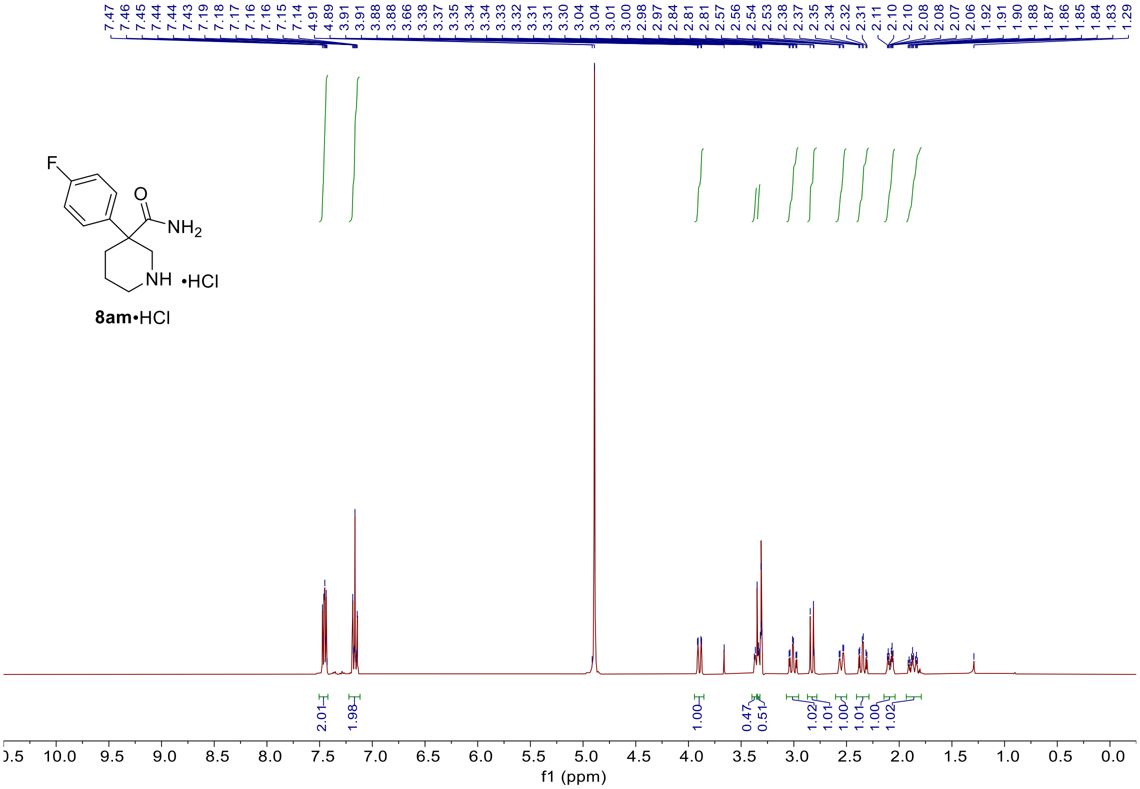

8am•HCl

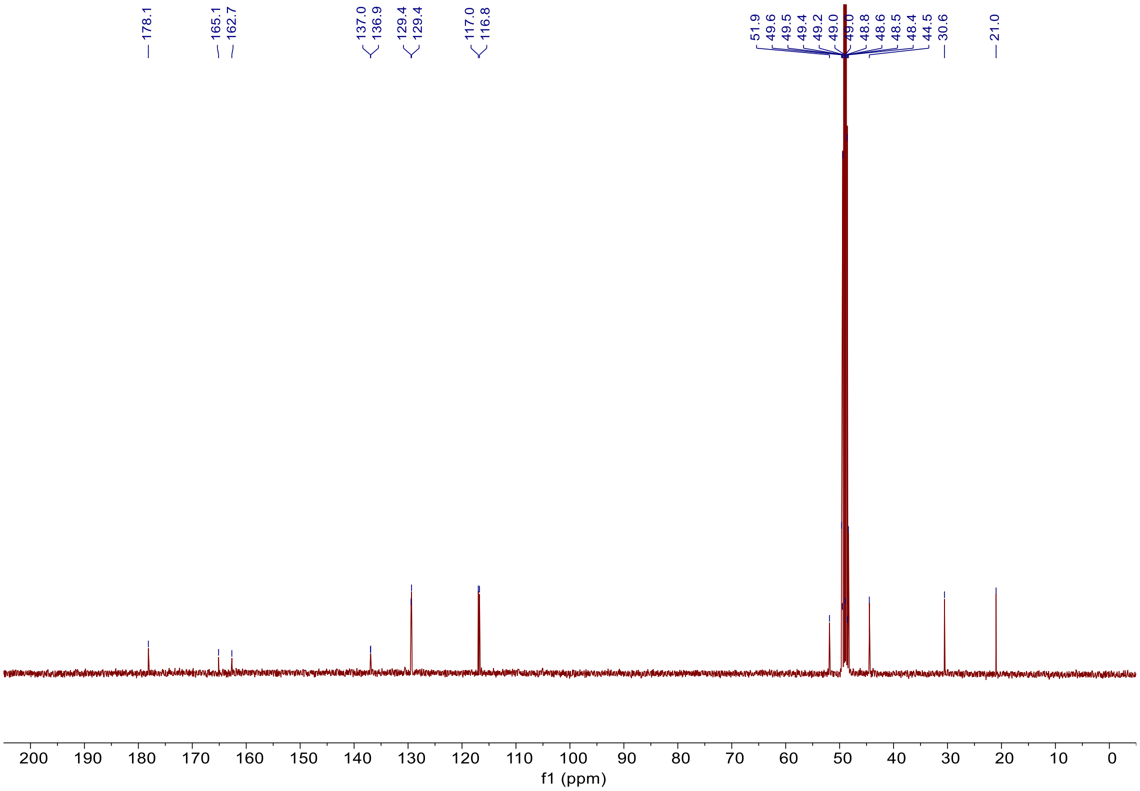

400 MHz ^1H NMR spectrum; 100.6 MHz ^{13}C NMR spectrum; MeOD-d_4 of **S27**

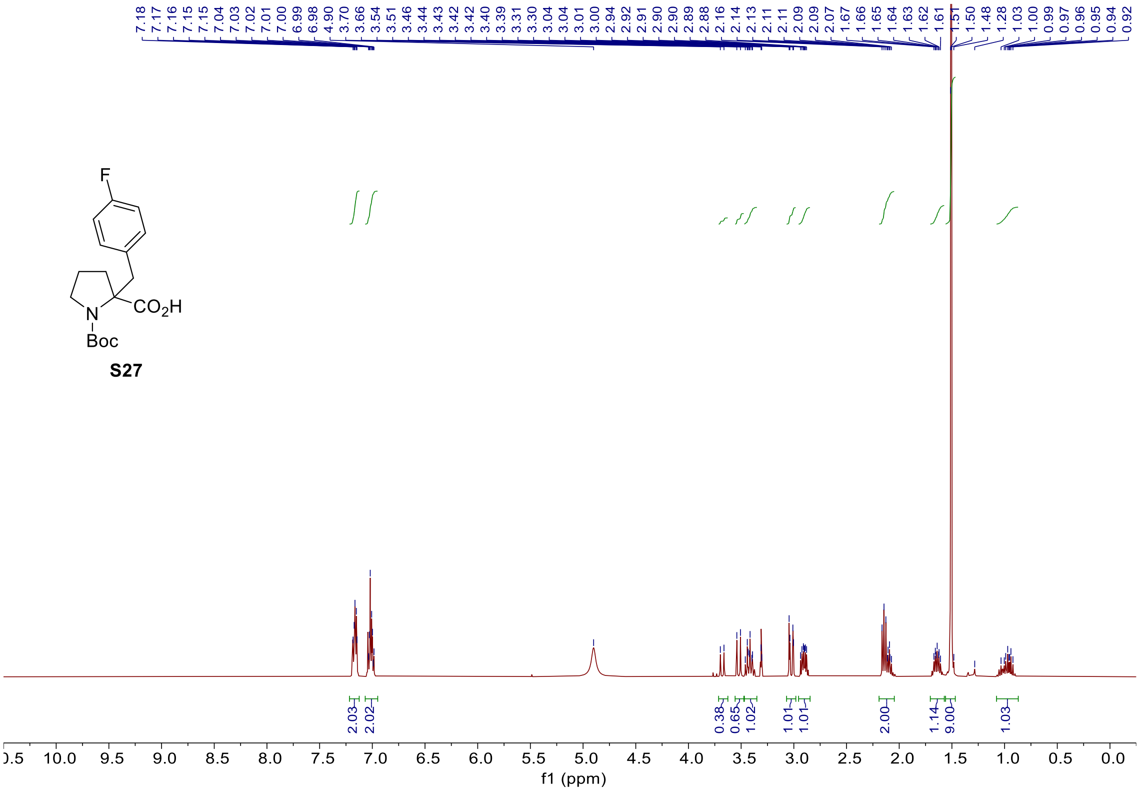

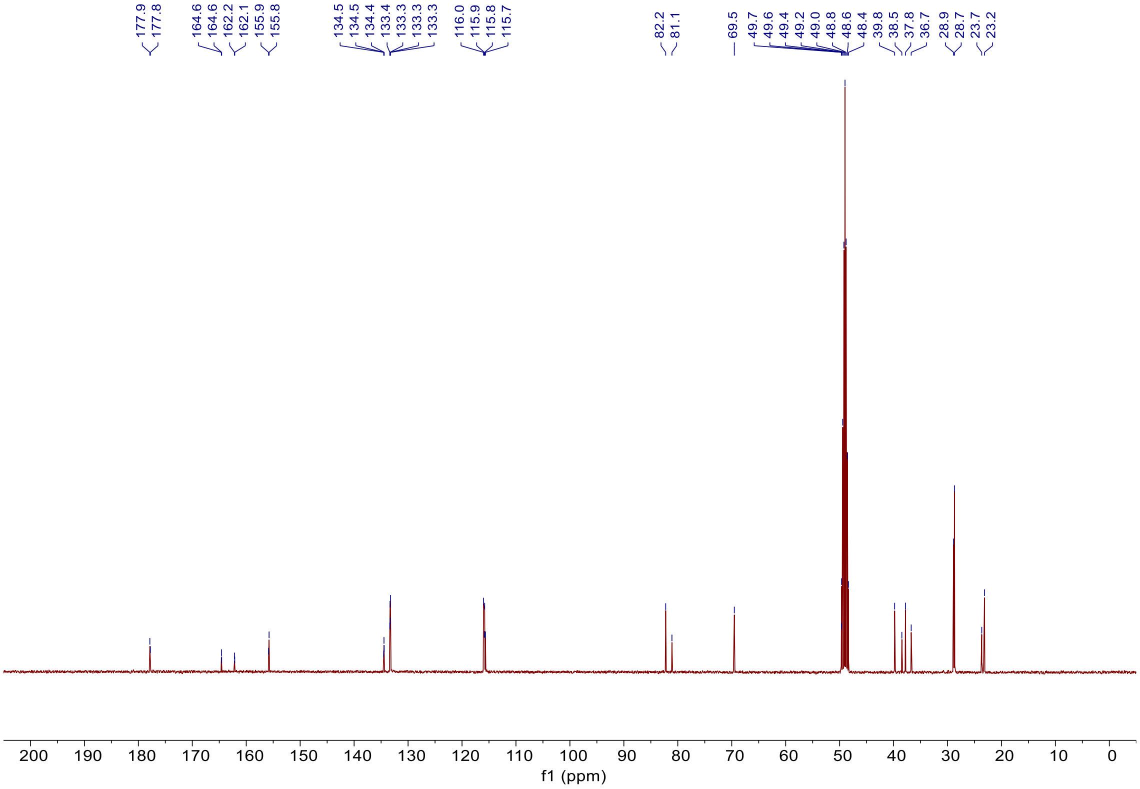

400 MHz ^1H NMR spectrum; 100.6 MHz ^{13}C NMR spectrum; $CDCl_3$ of **S28**

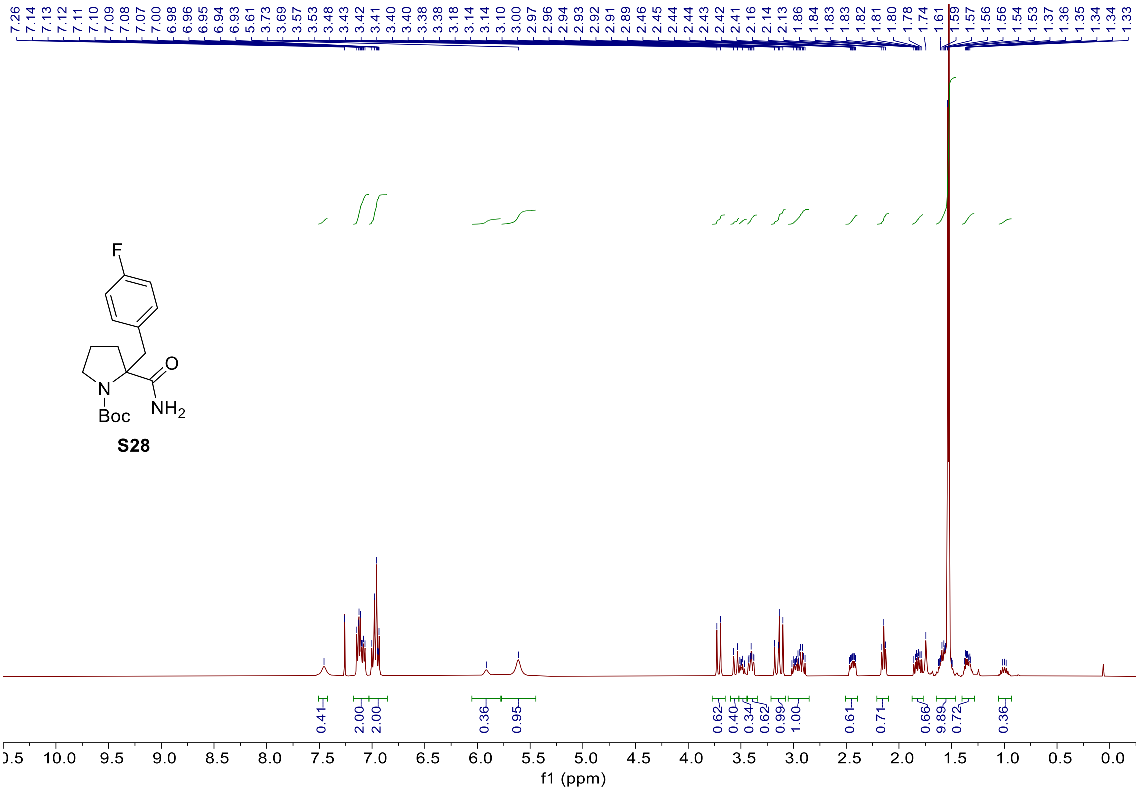

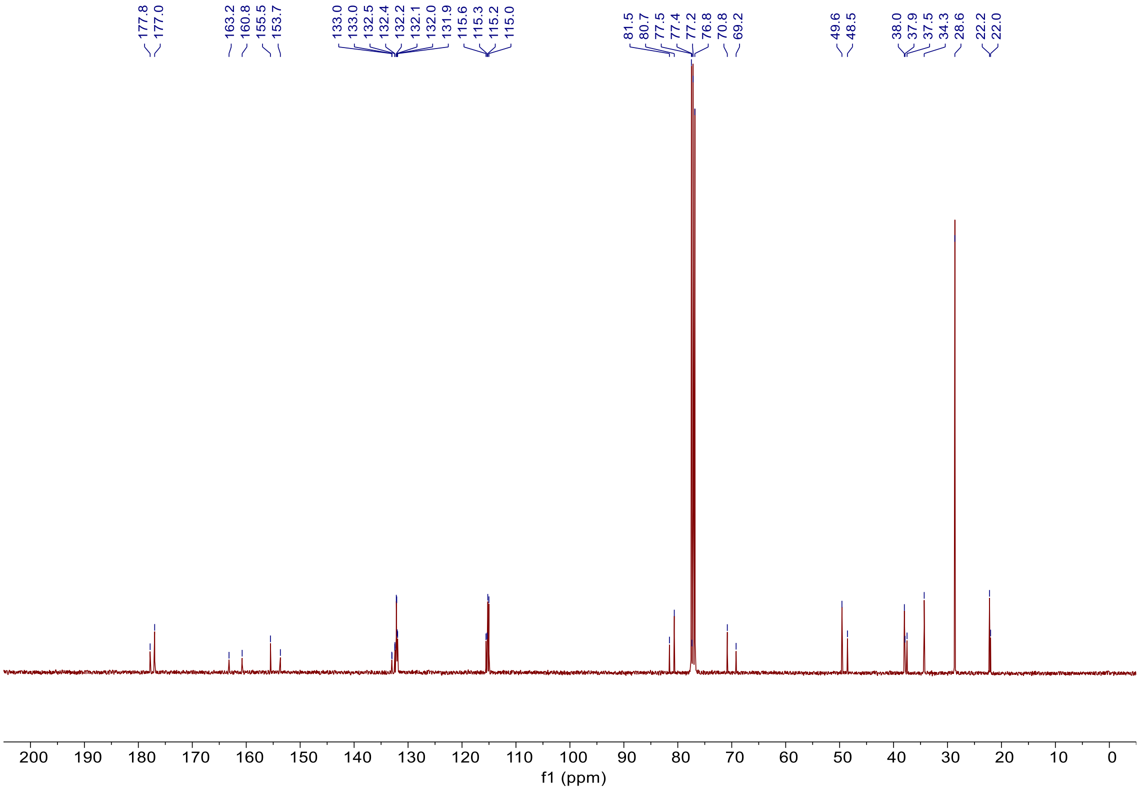

400 MHz ^{1}H NMR spectrum; 100.6 MHz ^{13}C NMR spectrum; MeOD-d_4 of **8an**•HCl

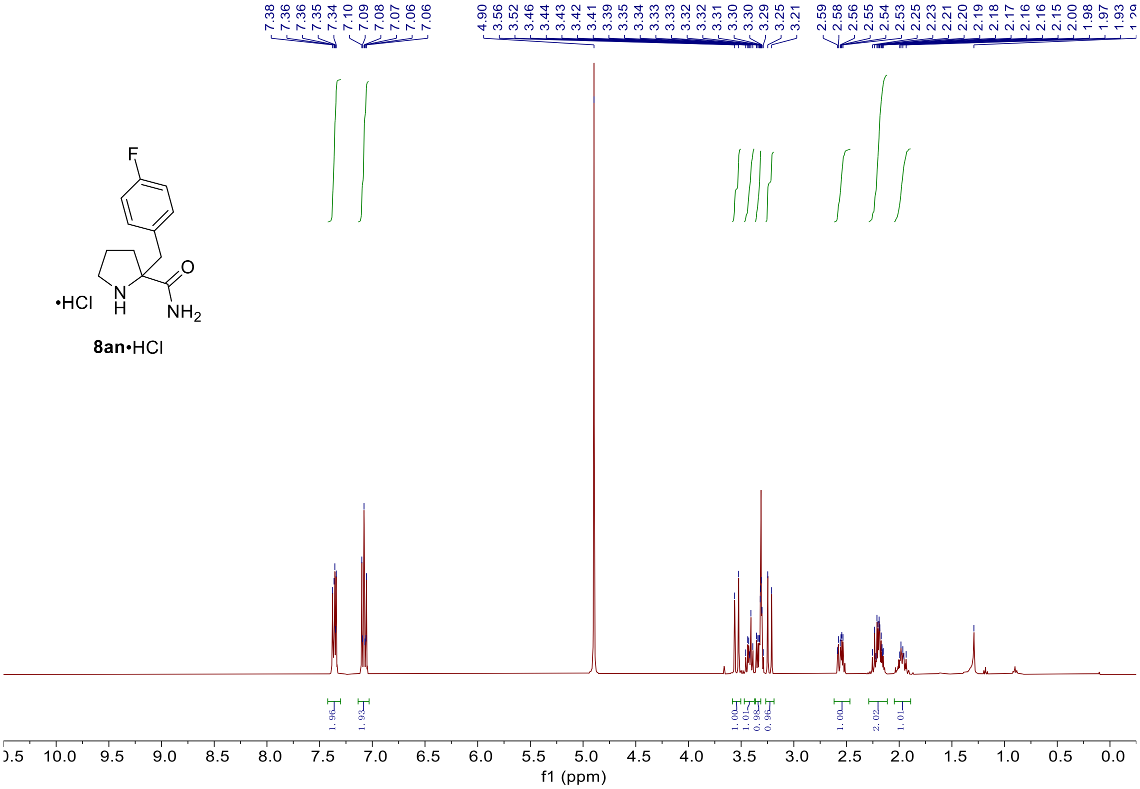

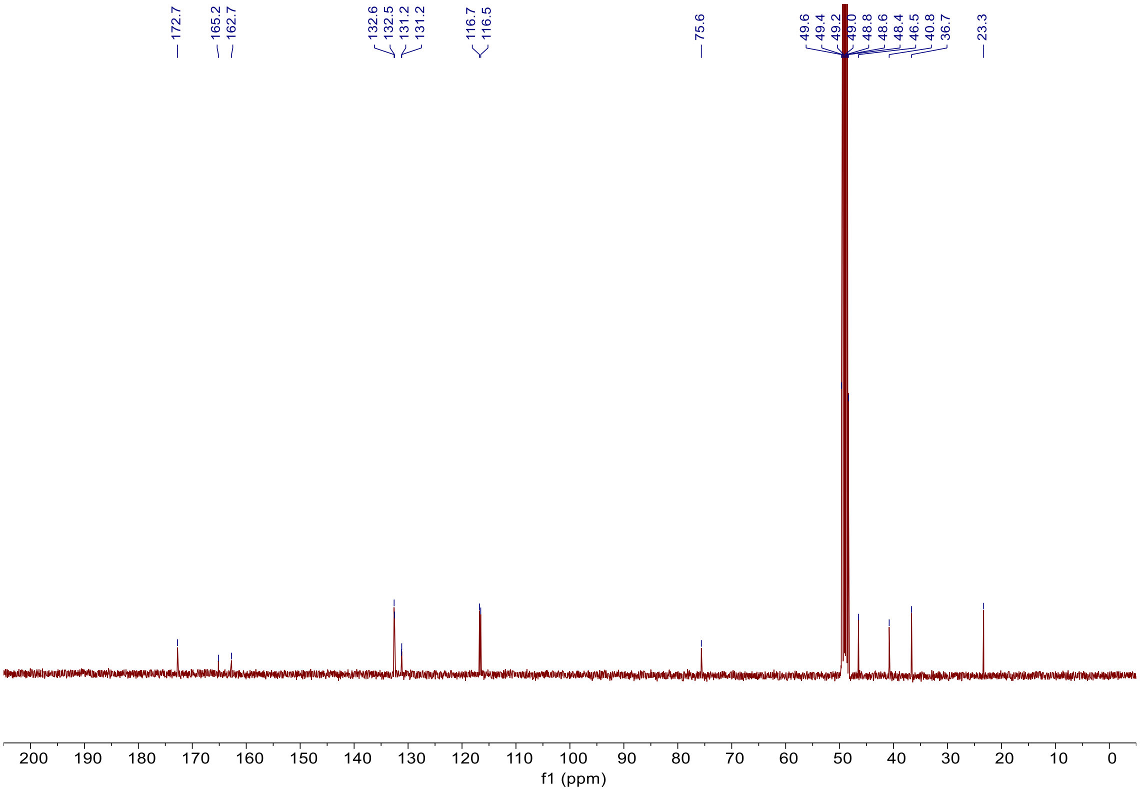

400 MHz ^{1}H NMR spectrum; 100.6 MHz ^{13}C NMR spectrum; $CDCl_3$ of **8ao**

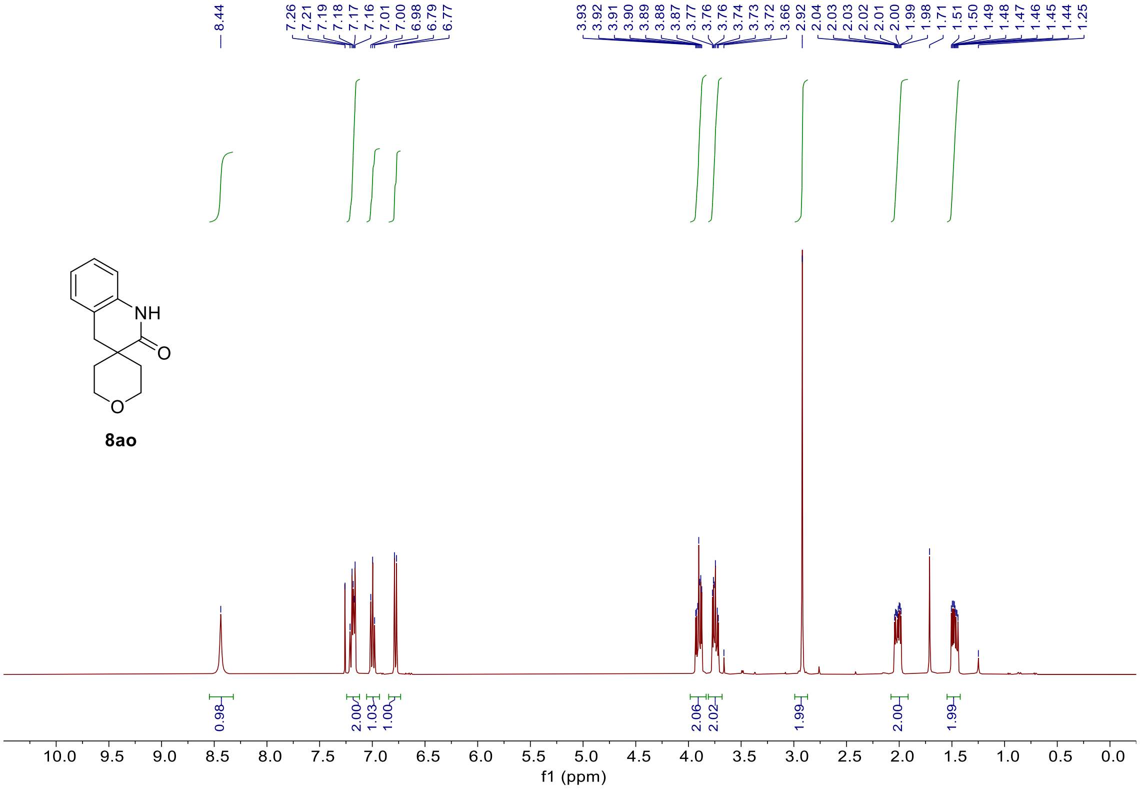

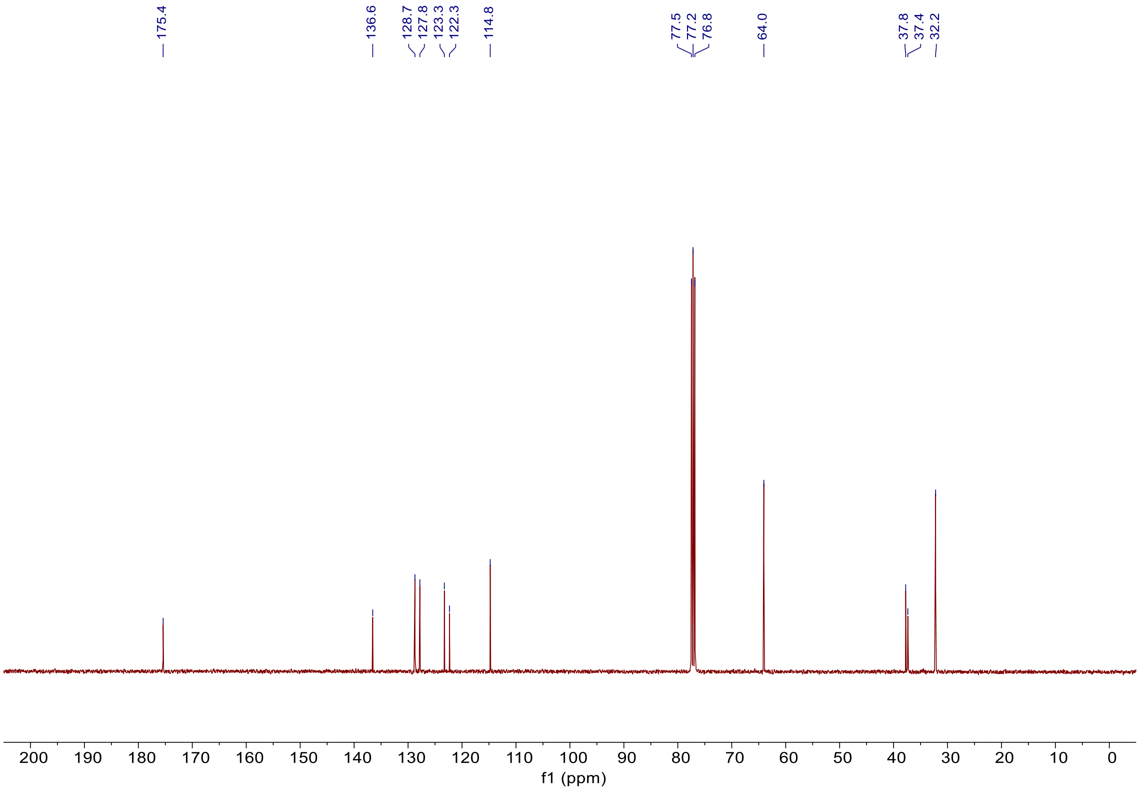

400 MHz ^{1}H NMR spectrum; 100.6 MHz ^{13}C NMR spectrum; $CDCl_3$ of **S30**

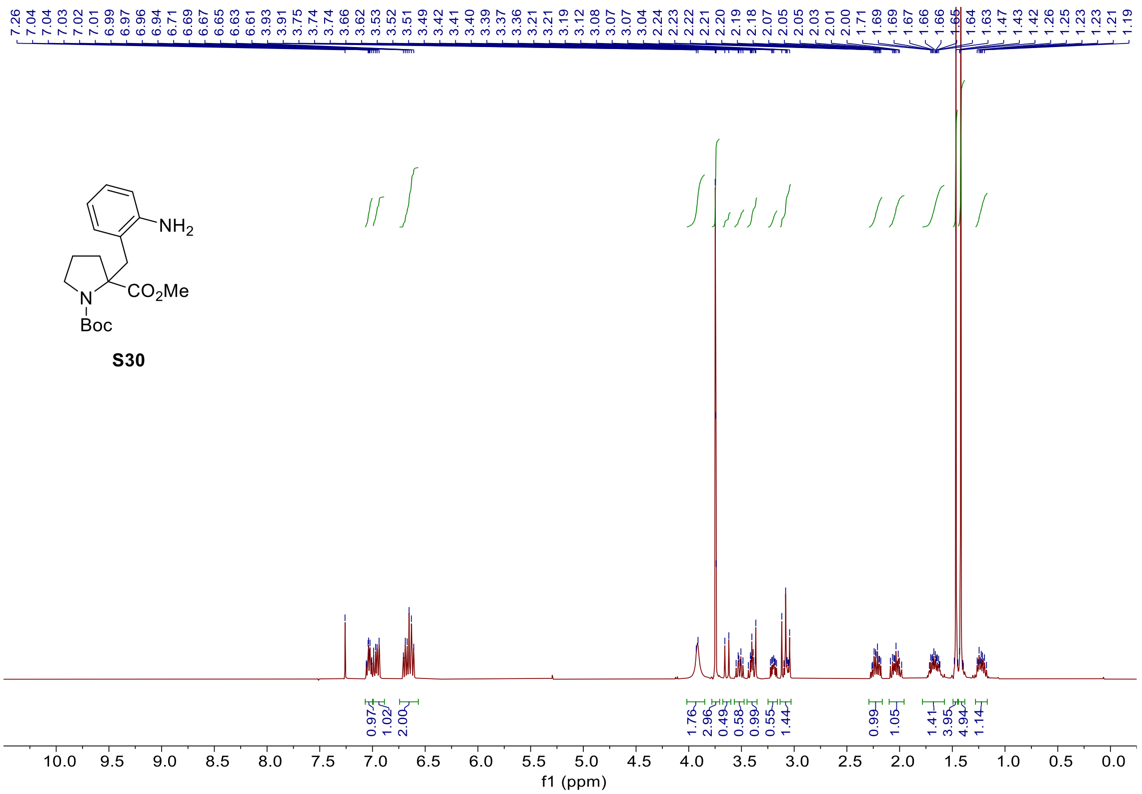

S30

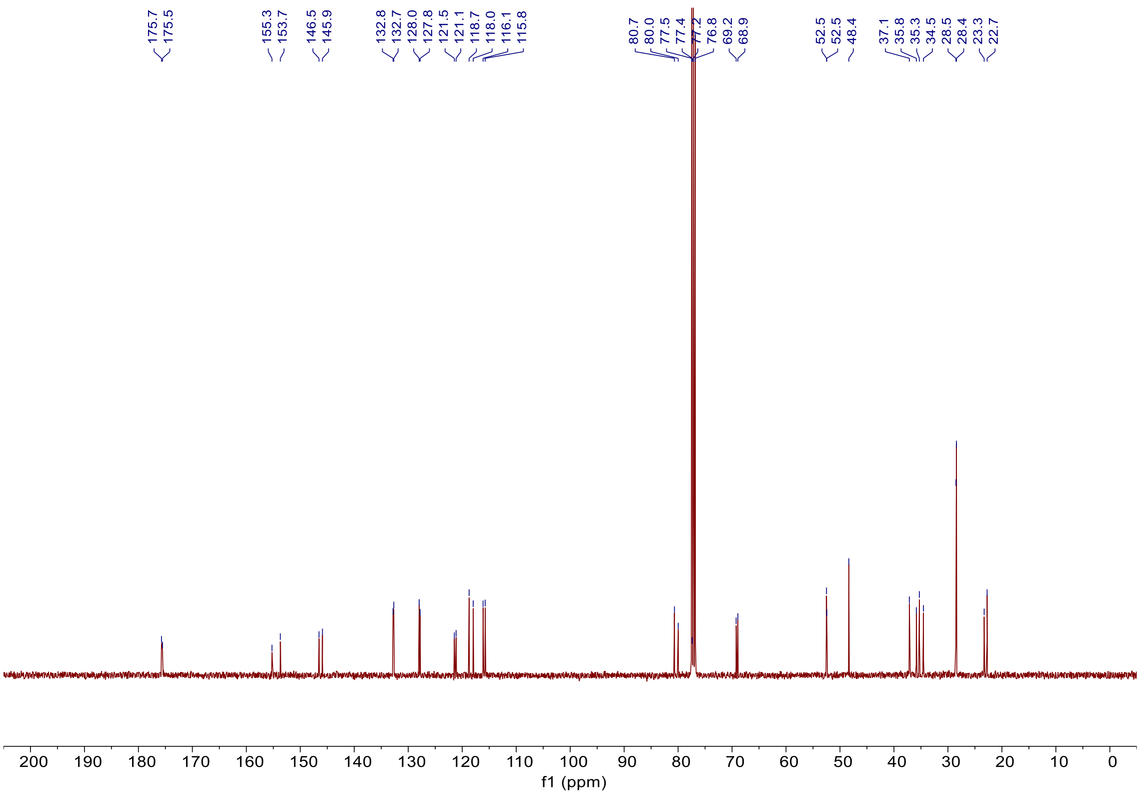

400 MHz ^{1}H NMR spectrum; 100.6 MHz ^{13}C NMR spectrum; $CDCl_3$ of **8ap**

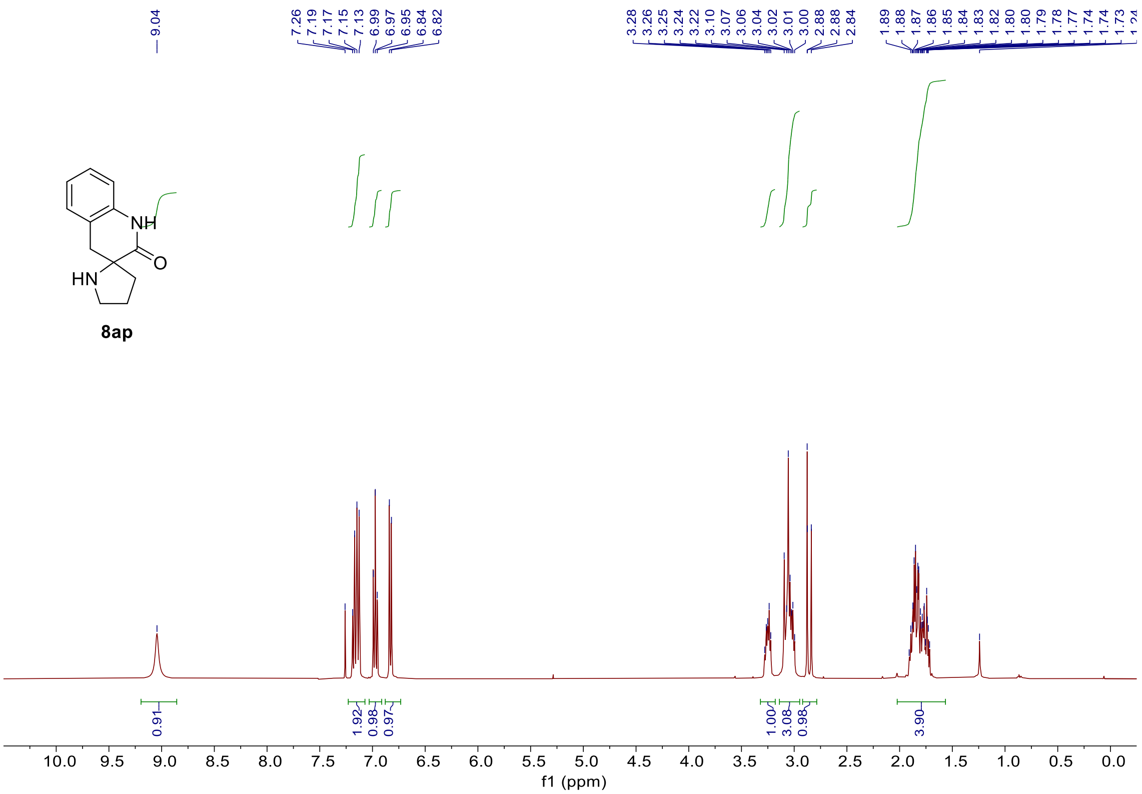

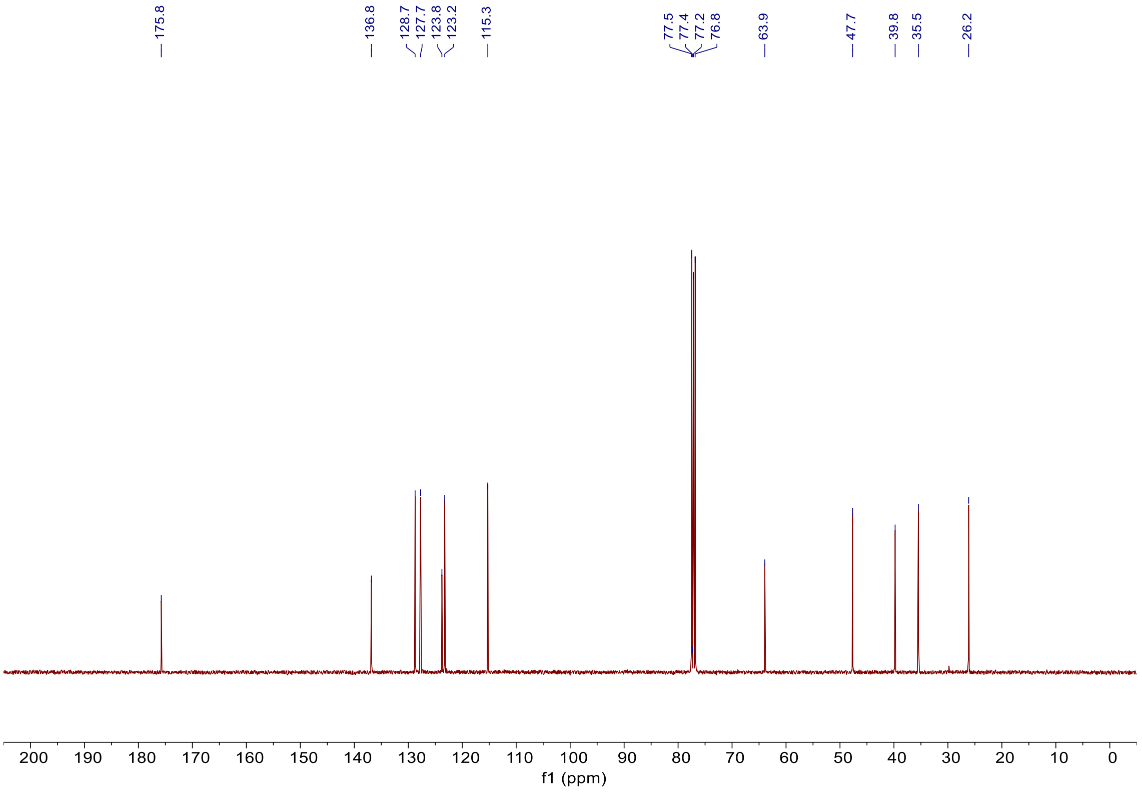

5. References

1 A. W. J. Logan, J. S. Parker, M. S. Hallside and J. W. Burton, *Org. Lett.*, 2012, **14**, 2940–2943.

2 J. Christoffers, J. Sluiter and J. Schmidt, *Synthesis*, 2011, 895–900.

3 WO2008031772A1, 2008.

4 B. M. Trost, Y. Zhang and T. Zhang, *J. Org. Chem.*, 2009, **74**, 5115–5117.

5 A. A. Carr, D. A. Hay, J. M. Kane and M. A. Staeger, *J. Org. Chem.*, 1990, **55**, 1399–1401.

6 A. Liljeblad, H.-M. Kavenius, P. Tähtinen and L. T. Kanerva, *Tetrahedron: Asymm.*, 2007, **18**, 181–191.

7 Q. Shi, M. C. Meehan, M. Galella, H. Park, P. Khandelwal, J. Hynes, T. G. M. Dhar and D. Marcoux, *Org. Lett.*, 2018, **20**, 337–340.

8 F. Vetica, A. Pelosi, A. Gambacorta, M. A. Loreto, M. Miceli and T. Gasperi, *Eur. J. Org. Chem.*, 2014, 1899-1906.

9 Y. Han, B. Tuccio, R. Lauricella, A. Rockenbauer, J. L. Zweier and F. A. Villamena, *J. Org. Chem.*, 2008, **73**, 2533–2541.

10 WO2003103604A2, 2003.

11 S. Pajk, M. Živec, R. Šink, I. Sosič, M. Neu, C. Chung, M. Martínez-Hoyos, E. Pérez-Herrán, D. Álvarez-Gómez, E. Álvarez-Ruíz, A. Mendoza-Losana, J. Castro-Pichel, D. Barros, L. Ballell-Pages, R. J. Young, M. A. Convery, L. Encinas and S. Gobec, *Eur. J. Med. Chem.*, 2016, **112**, 252–257.

12 J. F. Darby, A. K. Gilio, B. Piniello, C. Roth, E. Blagova, R. E. Hubbard, C. Rovira, G. J. Davies and L. Wu, *ACS Catal.*, 2020, **10**, 8590–8596.

13 M. Schuller, J. Correy, Galen, S. Gahbauer, D. Fearon, T. Wu, R. Efraín Díaz, I. D. Young, L. Carvalho Martins, D. H. Smith, U. Schulze-Gahmen, T. W. Owens, I. Deshpande, G. E. Merz, A. C. Thwin, J. T. Biel, J. K. Peters, M. Moritz, N. Herrera, H. T. Kratochvil, A. Aimon, J. M. Bennett, J. Brandão-Neto, A. E. Cohen, A. Dias, A. Douangamath, L. Dunnett, O. Fedorov, M. P. Ferla, M. R. Fuchs, T. J. Gorrie-Stone, J. M. Holton, M. G. Johnson, T. Krojer, G. Meigs, A. J. Powell, J. G. M. Rack, V. L. Rangel, S. Russi, R. E. Skyner, C. A. Smith, A. S. Soares, J. L. Wierman, K. Zhu, P. O'Brien, N. Jura, A. Ashworth, J. J. Irwin, M. C. Thompson, J. E. Gestwicki, F. von Delft, B. K. Shoichet, J. S. Fraser and I. Ahel, *Sci. Adv.*, 2022, **7**, eabf8711.

14 N. M. Pearce, T. Krojer, A. R. Bradley, P. Collins, R. P. Nowak, R. Talon, B. D. Marsden, S. Kelm, J. Shi, C. M. Deane and F. von Delft, *Nat. Commun.*, 2017, **8**, 15123.

15 M. Mayer and B. Meyer, *Angew. Chem. Int. Ed.*, 1999, **38**, 1784–1788.
